# Patterns of chromatin accessibility along the anterior-posterior axis in the early *Drosophila* embryo

**DOI:** 10.1371/journal.pgen.1007367

**Published:** 2018-05-04

**Authors:** Jenna E. Haines, Michael B. Eisen

**Affiliations:** 1 Department of Molecular and Cell Biology, University of California, Berkeley, Berkeley, United States of America; 2 Department of Integrative Biology, University of California, Berkeley, Berkeley, United States of America; 3 Howard Hughes Medical Institute, University of California, Berkeley, Berkeley, United States of America; Max Planck Institute for Molecular Biomedicine, GERMANY

## Abstract

As the *Drosophila* embryo transitions from the use of maternal RNAs to zygotic transcription, domains of open chromatin, with relatively low nucleosome density and specific histone marks, are established at promoters and enhancers involved in patterned embryonic transcription. However it remains unclear how regions of activity are established during early embryogenesis, and if they are the product of spatially restricted or ubiquitous processes. To shed light on this question, we probed chromatin accessibility across the anterior-posterior axis (A-P) of early *Drosophila melanogaster* embryos by applying a transposon based assay for chromatin accessibility (ATAC-seq) to anterior and posterior halves of hand-dissected, cellular blastoderm embryos. We find that genome-wide chromatin accessibility is highly similar between the two halves, with regions that manifest significant accessibility in one half of the embryo almost always accessible in the other half, even for promoters that are active in exclusively one half of the embryo. These data support previous studies that show that chromatin accessibility is not a direct result of activity, and point to a role for ubiquitous factors or processes in establishing chromatin accessibility at promoters in the early embryo. However, in concordance with similar works, we find that at enhancers active exclusively in one half of the embryo, we observe a significant skew towards greater accessibility in the region of their activity, highlighting the role of patterning factors such as Bicoid in this process.

## Author summary

DNA in the nuclei of animals and other eukaryotes is not floating around freely. Rather it is wrapped around proteins called histones that are in turn compacted into higher order structures called chromatin. Highly compact chromatin can restrict which genes and regulatory regions are accessible to the machinery that turns DNA into RNA, effectively shutting genes off. In contrast, chromatin at active sequences tends to be more open and accessible. However, it is still unknown whether open chromatin enables genes to be active, or if open chromatin is a byproduct of activity. Here we use a genomic technique to compare chromatin in the anterior and posterior halves of young fly (*Drosophila melanogaster*) embryos and show that two important classes of regulatory sequences—promoters and enhancers—are accessible in parts of the embryo where they are transcriptionally active and, often, in parts where they are not. This suggests that chromatin accessibility is not a direct consequence of activity per se, and supports a model in which chromatin around active genes and enhancers is systematically opened by specific regulator proteins and is later refined by activity.

## Introduction

During early embryogenesis all animal genomes undergo a transition from a largely quiescent to a highly active state with widespread zygotic transcription [1]. This process, known as the maternal-to-zygotic transition (MZT), involves a major reorganization of chromatin, during which active and inactive regions are established which are distinguished by nucleosome composition, density and post-translational modifications [2–6]. It is generally thought that active—or “open”—chromatin facilitates the binding of polymerases, transcription factors and other proteins to target sequences, while inactive—or “closed”—chromatin limits the scope of their activity, although the degree to which chromatin state is instructive remains controversial [7,8]. Two important open questions are how genomic locations of active and inactive chromatin are encoded in the genome and how their active state is established, especially during the MZT, which follows early embryonic mitotic divisions where little or no differentiation into open and closed chromatin has been observed [2].

In *Drosophila melanogaster*, zygotic transcription largely begins at the seventh syncytial mitotic cycle (although there is evidence for low levels of transcription from the beginning of embryogenesis [9]) and gradually increases until the end of mitotic cycle 13, when the embryo has several thousand nuclei and widespread zygotic transcription is observed [10,11]. Many of the genes activated during the MZT produce mRNAs that have spatially restricted distributions. These patterns are established through the activity of transcriptional enhancers, *cis*-regulatory sequences that integrate activating and repressive inputs from well-characterized, patterning transcription factors to produce novel, increasingly precise transcriptional outputs [12–15].

It is widely assumed that the interactions among patterning factors and the DNA to which they bind plays a central role in determining which sequences will function as enhancers, and that their competition with nucleosomes and recruitment of chromatin remodeling factors establish chromatin accessibility at selected sites [16–19]. The anterior morphogen Bicoid, for example, has been shown to create open chromatin at a subset of its targets [19] in the early embryo.

However, we and others have shown that a parallel system involving the ubiquitously expressed, maternally-deposited pioneer factor Zelda also plays a role in this process [2,20–24]. Zelda binds prior to the MZT to a large fraction of the enhancers and promoters that become active once widespread zygotic transcription begins [20,25]. Most MZT enhancers and promoters contain conserved Zelda binding sites that are highly predictive of both transcription factor activity and chromatin accessibility [20]. Furthermore, Zelda binding is associated with changes to chromatin, including nucleosome depletion and specific post-translational modifications of histones [2,20–24].

Although abundant genetic, genomic and biochemical data support the importance of Zelda in establishing enhancer and promoter activity, many aspects of Zelda activity remain unresolved. While embryos lacking Zelda show severe defects prior to gastrulation, patterned, enhancer-driven transcription is not eliminated in Zelda^-^ embryos [22,25–28]. This could reflect the activity of additional pioneer factors [21] such as the ubiquitously expressed trithorax-like/GAGA Factor (or GAF) which plays an important role in establishing accessibility at promoters [21,29–31] and is likely associated with changes in the nucleo-cytoplasmic ratio [32,33].

Because Zelda and GAF are ubiquitously expressed, while patterning factors have spatially restricted expression, we reasoned that we could probe their relative contributions to the establishment of chromatin accessibility by measuring spatial patterns of chromatin accessibility in the early embryo. As a first step towards this end, here we compare chromatin accessibility in anterior and posterior regions of the *D*. *melanogaster* embryo.

## Results

### Spatially resolved ATAC-seq is robust and consistent with whole embryo measurements of chromatin accessibility

**Fig 1:**
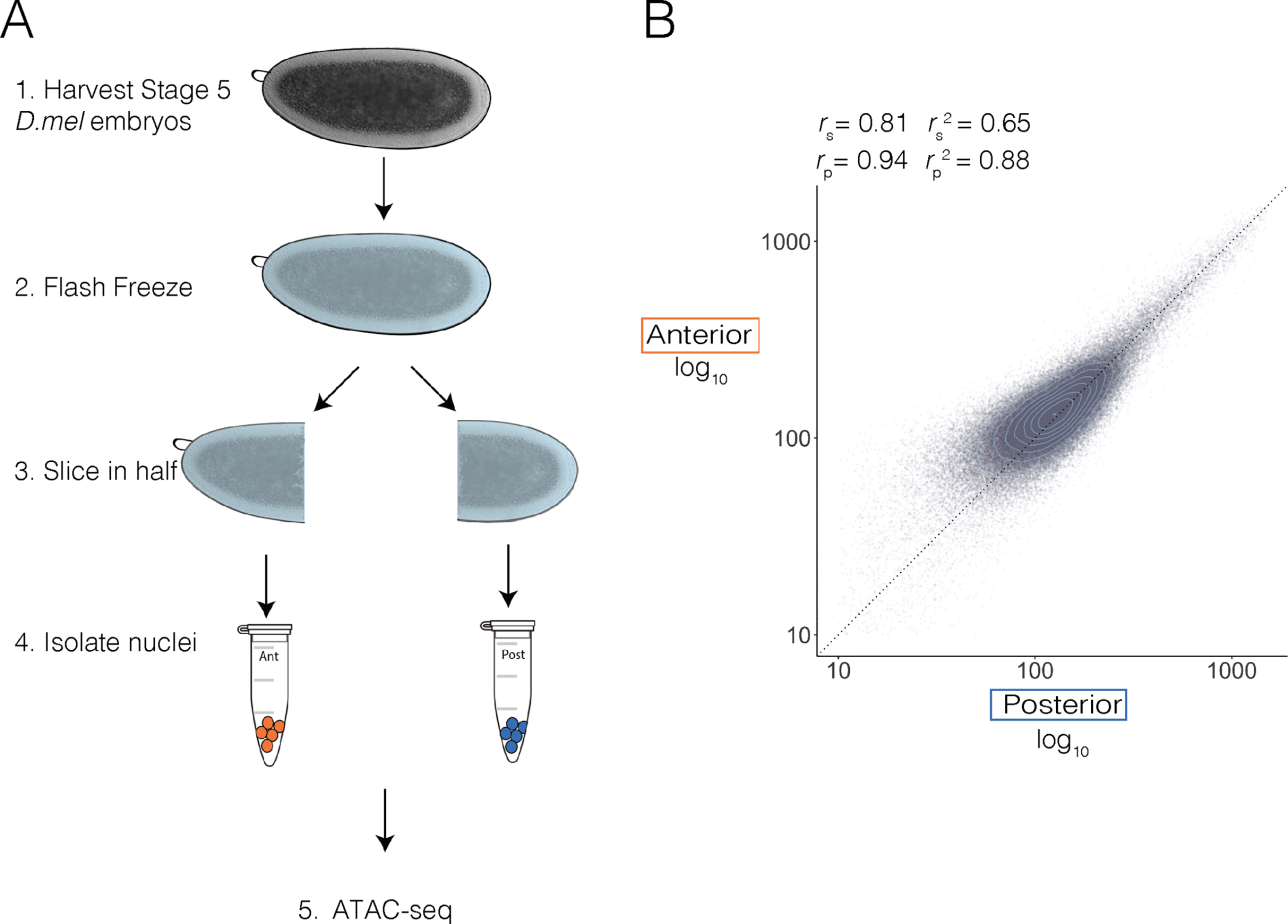
ATAC-seq on dissected, frozen, embryo halves. (A) Stage 5, hand sorted *Drosophila* embryos were flash frozen over dry ice in a buffer containing 5% glycerol and manually sliced in half with a scalpel. Twenty anterior and posterior halves were collected, homogenized, and the nuclei were isolated. ATAC-seq was then performed as described in [34] with three times Tn5 transposase. (B) Scatter plot of normalized ATAC-seq signal over 1kb adjacent windows that tile the *Drosophila* genome in posterior (x) and anterior (y) samples shows high degree of correlation between the anterior and posterior halves. The Spearman correlation coefficient (denoted by *r*_S_) is 0.81. The Pearson correlation coefficient (denoted by *r*_p_) is 0.94. X and Y are log transformed. Light blue circles denote point density.

To determine the extent to which chromatin accessibility is spatially patterned along the A-P axis in the early embryo, we manually separated anterior and posterior embryo halves and performed a modified ATAC-seq [34] protocol on each half separately. Briefly, we collected cellular blastoderm embryos (mitotic cycle 14, embryonic stage 5), flash froze them in liquid nitrogen, and then sliced each embryo at approximately 50% egg length (ascertained by eye) with a chilled scalpel, separating anterior and posterior halves into separate pools (Fig 1A). We isolated nuclei from 20 anterior halves (in duplicate), 20 posterior halves (in duplicate), 10 frozen unsliced embryos, and a mixed sample containing a subset of nuclei from anterior and posterior samples and applied the ATAC-seq “tagmentation” process to each sample. We sequenced the resulting libraries, mapped reads to the *D*. *melanogaster* genome and normalized the data using standard methods (S1 Fig).

ATAC-seq accessibility profiles generated from sliced and unsliced whole embryo samples correlated highly, demonstrating that the slicing process does not introduce any biases (*r*_p_ = 0.95, S2C Fig). Both halves correlate with published DNaseI hypersensitivity data from similar embryo stages [35], demonstrating that our embryo preparation protocol coupled with ATAC-seq can accurately map accessibility in the equivalent of 10 whole frozen embryos (*r*_p_ > 0.78, S2A Fig). Biological replicates of anterior and posterior halves that were collected, sliced, and tagmented independently moderately correlate with each other (anterior replicates *r*_p_ = 0.88, posterior replicates *r*_p_ = 0.80, S2B Fig). To call peaks using MACS2, we first merged replicates to increase the total read number and decrease spurious peaks that arise from low coverage regions [32,36]. We then filtered our peaks for those that were found in both replicates (methods).

### Globally similar chromatin accessibility patterns in anterior and posterior embryo halves

**Fig 2:**
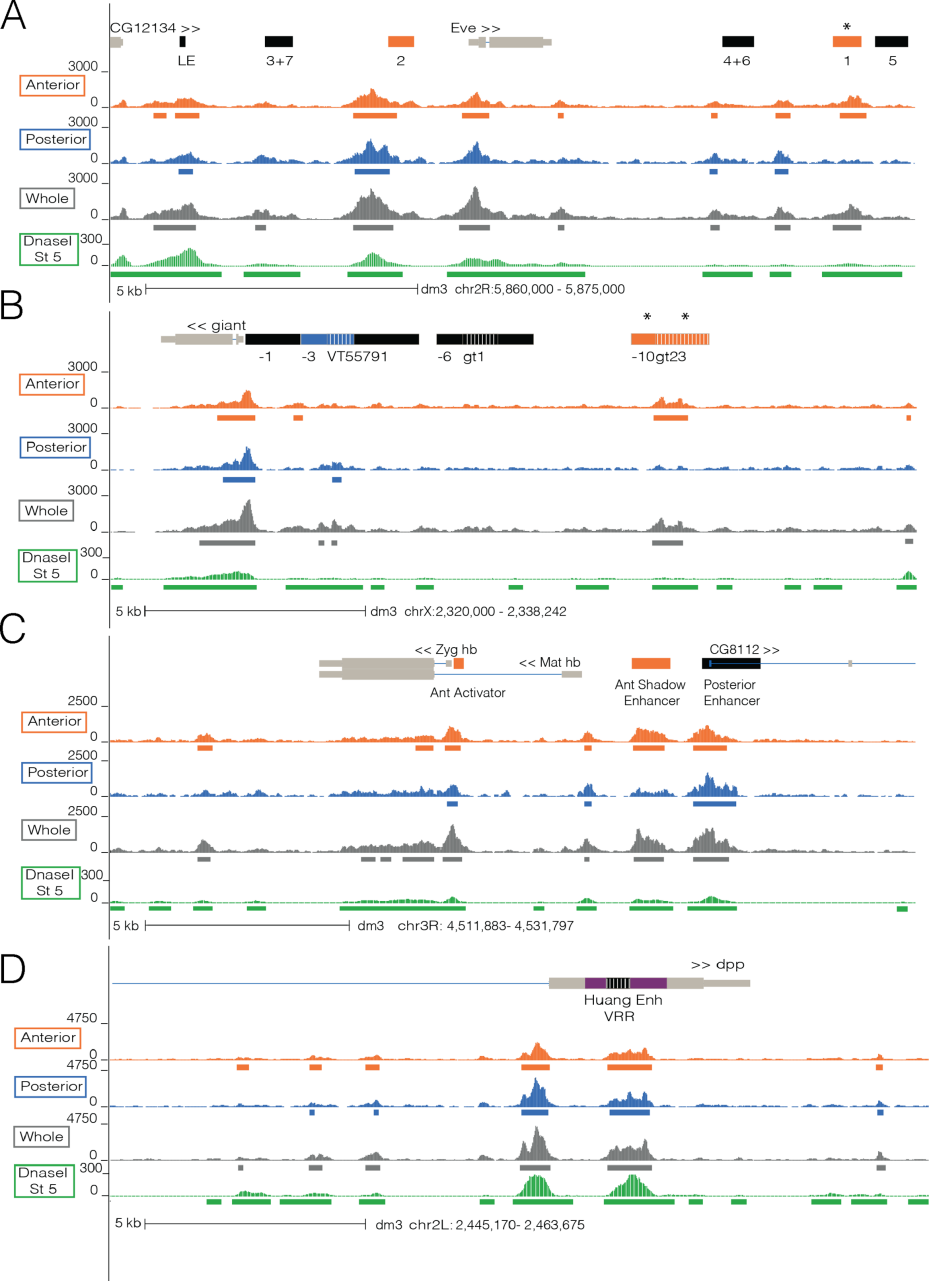
Chromatin accessibility differences and similarities at A-P and D-V patterning loci. Normalized ATAC-seq signal of anterior (orange), posterior (blue), whole embryo (gray) is depicted at *even-skipped* (A), *giant* (B), and *hunchback* (C) loci which contain A-P patterning enhancers and promoters and at *decapentaplegic* (D), a D-V patterning gene. Chromatin accessibility signal derived from DNaseI data for stage 5 *Drosophila* embryos is depicted in green [35]. Colored bars represent peaks called in anterior (orange), posterior (blue), whole (gray), and in DNaseI data (green). Asterisks denote annotated features that show significant changes in accessibility between the anterior and posterior halves. Light gray bars denote the gene annotation while the black bars denote annotated enhancers. Colored annotation bars represent enhancers analyzed in Fig 3. Dashed lines in the enhancer bars signify overlapping enhancers.

Genome-wide, chromatin accessibility in the anterior and posterior halves is remarkably similar (Fig 1B; *r*_p_ = 0.94 on data binned into 1kb windows and *r*_p_ = 0.90 for all whole embryo peaks S3 Fig). Dramatic changes in chromatin accessibility have been observed between early (stage 5) and later stage (stage 14) *Drosophila* embryos [17,35]. Expectedly, A-P halves are more similar to each other than embryos from stage 5 and 14 (*r*_p_ = 0.66, S4 Fig). The conservation of chromatin accessibility patterns between halves is detailed in Fig 2, which shows the results of our ATAC-seq experiments near loci of three A-P patterning genes (*even-skipped*, *giant*, and *hunchback*) and one dorsoventral patterning gene (*dpp*).

Each of these A-P loci contains enhancers that are active exclusively in the anterior or posterior half of the embryo (denoted by colored in Fig 2). For some, the peaks are of similar heights in both halves, such as at *eve* stripe 2 (anterior ATAC-seq signal / posterior ATAC-seq Signal—755/686). However there are some examples where accessibility is clearly reduced in the inactive half, such as at *eve* stripe 1 (1083/336), the *gt* anterior enhancers 23 (648/211) and -10 (513/175) (Fig 2, marked by asterisks).

### Most A-P enhancers are open in both halves of embryo but tend to be more accessible where they are active

To get a more systematic view of the relationship between transcriptional activity and spatial patterns of chromatin accessibility, we used available genome annotation and published *in situ* hybridization experiments to systematically identify A-P and dorsal-ventral (D-V) (as a control) patterning enhancers whose transcriptional outputs are restricted to one half of the embryo [37–48] (S1 File). We excluded enhancers and promoters of genes expressed only around 50% egg length because the precision of manual slicing is most likely variable. We also excluded enhancers that did not overlap peaks called in any of the anterior, posterior, or whole samples, leaving 85 A-P and D-V patterning enhancers.

**Fig 3:**
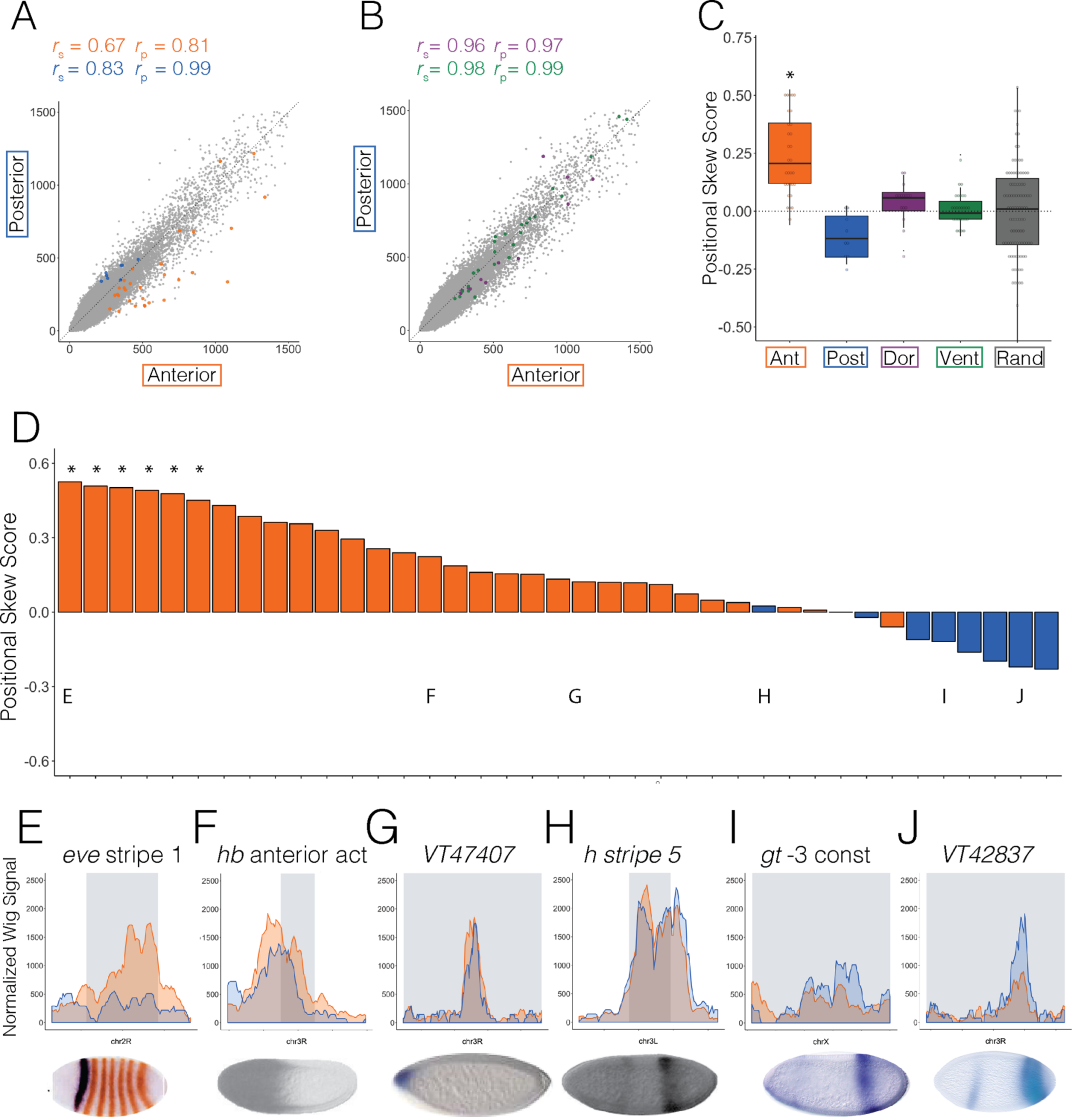
A-P patterning enhancers tend to be more accessible where they are active. Scatter plots showing normalized ATAC-seq signal in anterior (x axis) and posterior (y axis) halves at (A) anterior (orange) and posterior (blue) and (B) dorsal (purple) and ventral (green) patterning enhancers active in Stage 5 embryos and at 1kb adjacent windows tiling the genome (A and B, gray). (C) Box plots showing the difference in mean and variation between average positional skew scores (methods) for anterior (orange), posterior (blue), dorsal (purple), and ventral (green) enhancers. Positional skew scores at random genomic regions (excluding genes and enhancers) that are selected so that their total ATAC-seq signal is of the same magnitude and distribution as the patterning enhancer and promoter set depicted here and in Fig 4 (methods). Pairwise t.tests confirm that the means of anterior patterning enhancers are significantly different than dorsal and ventral patterning enhancers and selected random regions. The mean positional skew score of posterior enhancers is not significantly different than dorsal, ventral, or random regions. (D) Bar graph shows the positional skew scores (methods) calculated for all anterior (orange) and posterior (blue) patterning enhancers in the dataset (S1 File). Asterisks denote enhancers whose accessibility skew scores show statistical significance over random regions (p < 0.05). (E-J) Normalized ATAC-seq signal across 1kb windows centered around *eve* stripe 1 (E), the *hunchback* anterior activator (F), Kvon region VT47407 (G), *hairy* stripe 5 (H), *giant* -3 construct (I), and Kvon region VT42837 (J) with anterior signal in orange and posterior signal in blue. Gray rectangles denote the location and size of the enhancer. Published *in situ* hybridization images depicting gene expression patterns driven by each enhancer are below each graph [39,86–88].

Patterning enhancers clearly trend towards greater accessibility in the embryo half where they are active (Fig 3). Normalized ATAC-seq signal at anterior enhancers (anterior *r*_p_ = 0.81) is less correlated than all 1kb regions genome-wide (gray; *r*_p_ = 0.94) or D-V patterning enhancers (*r*_p_ = 0.97) while posterior patterning enhancers (*r*_p_ = 0.99) correlate similar to the genome-wide measurements (anterior n = 30, orange; posterior n = 9, blue; dorsal n = 16, purple; ventral n = 27, green; Fig 3A and 3B). From this, it is clear that at anterior patterning enhancers, chromatin accessibility is greater in the anterior half.

We computed a measure of differential accessibility (positional skew score) for each A-P enhancer by dividing the difference in accessibility in the anterior and posterior half by total accessibility, such that positive scores denote loci that are more accessible in the anterior half, negative scores signify loci that are more accessible in the posterior half, and loci with a score of zero have no difference in accessibility. We found that only anterior enhancers have a significantly greater mean positional skew score than D-V enhancers (p_ant_ < 0.006 verses dorsal; 5.57e-05 versus ventral) or random genomic regions with similar total accessibility (p_ant_ < 6.68e-08, Fig 3C and S1 Table).

**Fig 4:**
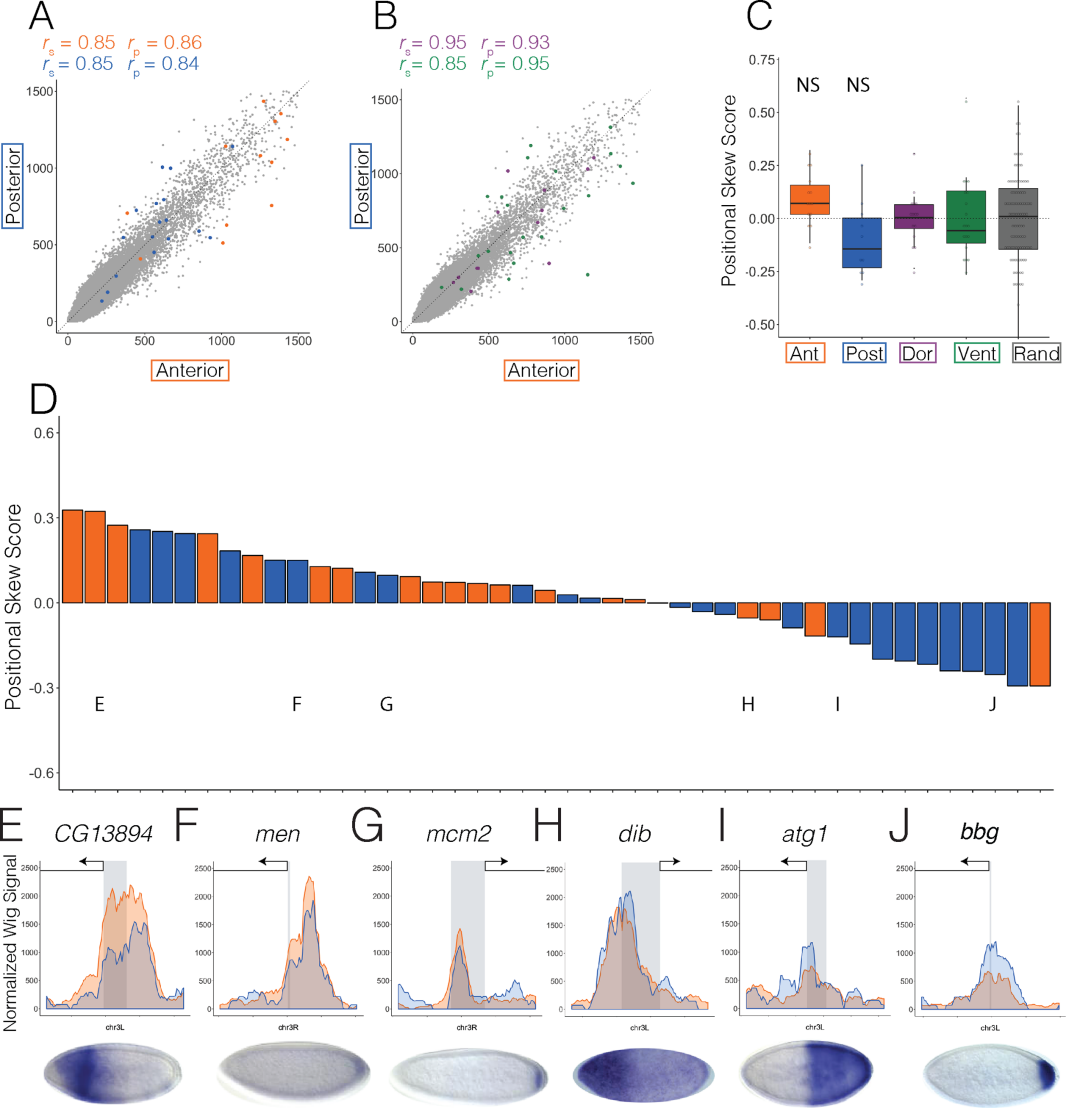
A-P patterning gene promoter accessibility does not correlate with activity. Scatter plots showing normalized ATAC-seq signal in anterior (x axis) and posterior (y axis) halves at (A) anterior (orange) and posterior (blue) and (B) dorsal (purple) and ventral (green) patterning promoters active in Stage 5 embryos and at 1kb adjacent windows tiling the genome (A and B, gray). (C) Box plots showing the difference in mean and variation between overall positional skew scores (methods) for anterior (orange), posterior (blue), dorsal (purple), and ventral (green) promoters of zygotically expressed genes only. Zygotic genes were classified as such from previously published transcriptome data [11]. Positional skew scores at random genomic regions (excluding genes and enhancers) with the same number and distribution of total ATAC-seq signal as the patterning enhancer and promoter set are in gray (methods). NS (not significant) refers to pairwise t.tests which confirm that the mean positional skew score of anterior and posterior patterning promoters is not significantly different than dorsal and ventral patterning promoters or random regions. (D) Bar graph shows positional skew scores calculated for all anterior (orange) and posterior (blue) patterning promoters in the dataset (S1 File). Asterisks denote promoters whose accessibility skew scores show statistical significance over random regions (p < 0.05). (E-J) Normalized ATAC-seq signal across 1kb windows centered around *CG13894* (E), *men* (F), *mcm2* (G), *dib* (H), *atg1* (I), and *bbg* (J) with anterior signal in orange and posterior signal in blue. Gray denotes the location and size of the promoter. Arrow denotes the direction of the gene. Published *in situ* hybridization images depicting gene expression patterns driven by each promoter are below each graph [46–48].

Accessibility at almost all anterior enhancers is skewed towards the anterior while that of posterior enhancers is skewed towards the posterior (Fig 3D). This pattern is in contrast to D-V patterning enhancers and promoters (S5 Fig) and A-P patterning promoters (Fig 4D). Although individually only six anterior enhancers had significant skews relative to random regions, it is remarkable that almost all of these enhancers are skewed towards the active half regardless of the degree of skew. Similar trends were seen when positional skew scores calculated from replicates were examined (S6 Fig) as well as in accessibility profiles derived from single embryo halves (S7 Fig).

In order to understand if this phenomena extends beyond annotated A-P patterning enhancers, we evaluated whether whole peaks that have chromatin accessibility skewed towards the anterior or posterior show specific patterning activity in the embryo. We overlapped significantly skewed peaks with regions identified in a genome-wide screen for enhancer activity [45]. We then utilized available *in situ* hybridization experiments to evaluate whether these fragments have spatially patterned activity (http://enhancers.starklab.org/). Indeed nine out of twelve significantly skewed enhancers showed spatially patterned activity (8 anterior and 1 posterior, S8D Fig).

### Promoters of A-P patterning genes are similarly accessible both when active and inactive

We next examined the promoters of A-P patterning genes using expression data from sections of embryos cryosliced along the A-P axis to curate lists of A-P patterning gene promoters [28]. Similar to our enhancer set, we only included promoters that overlapped accessibility peaks called in whole, anterior, or posterior samples and that are also associated with patterned expression confirmed by *in situ* hybridization assays (n = 19 anterior promoters, n = 25 posterior promoters, S1 File).

Though accessibility in the active and inactive halves is only slightly more similar at anterior promoters (*r*_p_ = 0.86) than at anterior enhancers (*r*_p_ = 0.81), it is comparable to posterior promoters(*r*_p_ = 0.84) and D-V enhancers and promoters (Fig 4A and 4B, dorsal promoters, *r*_p_ = 0.93; ventral promoters, *r*_p_ = 0.95). We confirmed these trends by showing that the mean positional skew score of anterior and posterior patterning promoters is not significantly different than D-V patterning promoters or random regions (Fig 4C and S1 Table). Though accessibility at promoters of A-P expressed, zygotic genes seems to show a very slight trend in the direction of activity, their means are not significantly different than that of random regions (Fig 4C). What is more telling is that there is no distinct skew of accessibility in the expected direction of activity, in contrast to what we observed for A-P enhancers (Fig 4D). From this we conclude that accessibility at promoters is not as correlated with transcriptional activity as enhancers.

### Anterior accessibility is associated with Bicoid binding while similarly accessible regions are enriched for Zelda binding

**Fig 5:**
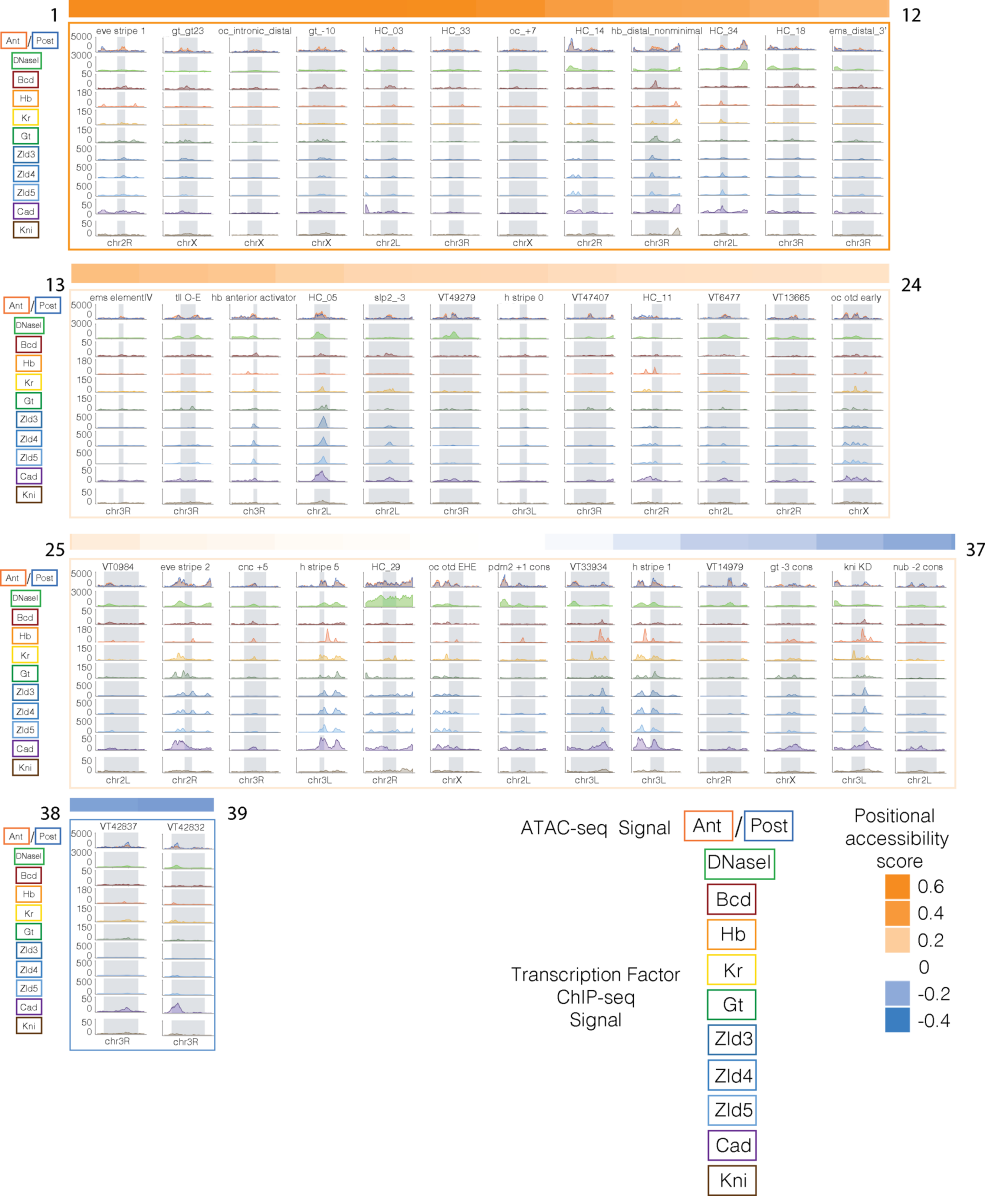
A-P patterning transcription factor binding at similarly and differentially accessible A-P patterning enhancers. A-P patterning enhancers from Fig 3 are ordered by positional skew score. Positional skew score is indicated by the colored bar above each panel–orange indicates more accessible in the anterior, blue indicates more accessible in the posterior, and white is similarly accessible in both halves. Each panel consists of normalized wig signal in a 3kb window around each enhancer (the actual enhancer region is denoted by a gray rectangle). The first panel shows normalized, merged, ATAC-seq signal in the anterior (orange) and posterior (blue) halves. The second panel shows DNaseI signal from stage 5 embryos [35] in green. The third through eleventh panels are normalized wig signal from ChIP-seq experiments of the following proteins: Bicoid (red), Hunchback (orange), Kruppel (yellow), Giant (green), Zelda from stage 3,4,and 5 embryos (dark, medium, and light blue), Caudal (purple), and Knirps (brown). The name of the enhancer is above each panel.

We then used published ChIP-seq data of A-P patterning factors from stage 5 *Drosophila* embryos [49] to examine binding patterns at similarly and differentially accessible A-P enhancers. We analyzed Bicoid, Caudal, Knirps, Giant, Hunchback, Kruppel, and Zelda binding data, normalized by the mean signal for each factor. A-P patterning enhancers that are more accessible in the anterior (shades of orange) generally are dominated by Bicoid binding, with strikingly little binding from other transcription factors, although there are some exceptions (Fig 5, S9 Fig). Enhancers more accessible in the posterior (shades of blue) generally have high Caudal, Knirps, Giant, and Kruppel binding, with more diversity in factors bound than at anteriorly accessible enhancers. Interestingly, enhancers with similar accessibility in both halves (shades of white) have a high diversity of factors binding—including Zelda (Fig 5, S9B Fig). These patterns reveal that while transcription factor binding clearly does not completely explain differential chromatin accessibility, there are clear differences in factor composition and density between differentially and similarly accessible enhancers.

We next examined transcription factor binding at peaks called in whole embryo ATAC-seq samples. Using a stringent statistical cutoff, we found 107 anteriorly skewed peaks, 9 posteriorly skewed peaks, and 6640 peaks that were not significantly skewed. Anteriorly skewed peaks were enriched for Bicoid, GAF, CF2 and to a lesser extent Zelda binding sites while the unskewed peaks were enriched for Zelda, pnr (GATA factor homolog), Dref (associated with insulators), and CF2. Due to so few peaks being skewed towards the posterior, none of the posterior peaks had significant motifs called, although GAF and Hunchback were enriched (S8 Fig). We then overlapped transcription factor peaks with anteriorly skewed, posteriorly skewed, and unskewed peaks and found that there is a significant depletion in Hunchback, Kruppel, Caudal, Knirps, and Zelda peaks in the anteriorly skewed peaks (p = 0.03, 0.005, 5.14E-10, 0.01, and 0.00001735 respectively, S8 Fig). These data further demonstrate the observation that, while transcription factor binding does not completely explain the differences between skewed and unskewed peaks, Bicoid, Zelda, and GAF are likely playing a role in shaping chromatin accessibility, as has already been reported by several recent studies [19,21,32].

## Discussion

We designed this experiment to ask if the open chromatin observed at active enhancers and promoters is found in every nucleus, suggesting a dominant role for ubiquitous factors in establishing regions of genomic activity in the early embryo, or if open chromatin is spatially restricted, suggesting a dominant role for patterning factors.

The data presented here offer several clarifying observations about the early *Drosophila* embryo. First, we find that the vast majority of regions observed to be accessible in whole embryos are equally accessible in anterior and posterior halves, including the promoters of many genes active in only one half. Second, for a curated set of enhancers driving patterns along the anterior-posterior axis, we find that chromatin is more accessible in nuclei where the enhancer is active. This is especially true of anterior patterning enhancers regulated by the primary anterior morphogen Bicoid, but is also observed for several posterior patterning enhancers. Third, in most cases where we see accessibility biased towards the embryo half where an element is active we also see clear chromatin in the “inactive” half.

Each of these observations comes with the caveat that the signal measured in each pool of halves is an average from approximately 60,000 nuclei that clearly limits the conclusions that we can draw. For example, for a given, region equal accessibility in both halves could indicate uniform accessibility in nuclei across the embryo, but could also arise from similar fractions of nuclei active in both halves. Similarly, it is impossible to resolve whether quantitative differences between the halves are the result of different numbers of nuclei with open chromatin, differences in the levels of accessibility, or both. And, finally, we likely cannot detect cases where only a small fraction of nuclei are active, although with our current methods it is difficult to accurately estimate what our resolving power is.

Nonetheless, a study published while this paper was under review that applied a single-nucleus chromatin accessibility assay, which is largely immune to these caveats, to a variety of embryonic stages largely confirms our finding [50]. They observe that early (2–4 hour) chromatin is more homogeneous than at later embryonic stages, and that the majority of regions of open chromatin in blastoderm embryos show no clear spatial pattern. Furthermore, when they applied an unbiased clustering method to single nuclei ATAC-seq data, nuclei from blastoderm embryos separate into two broad populations, each with increased accessibility in enhancers with anterior and posterior biases respectively.

**Fig 6:**
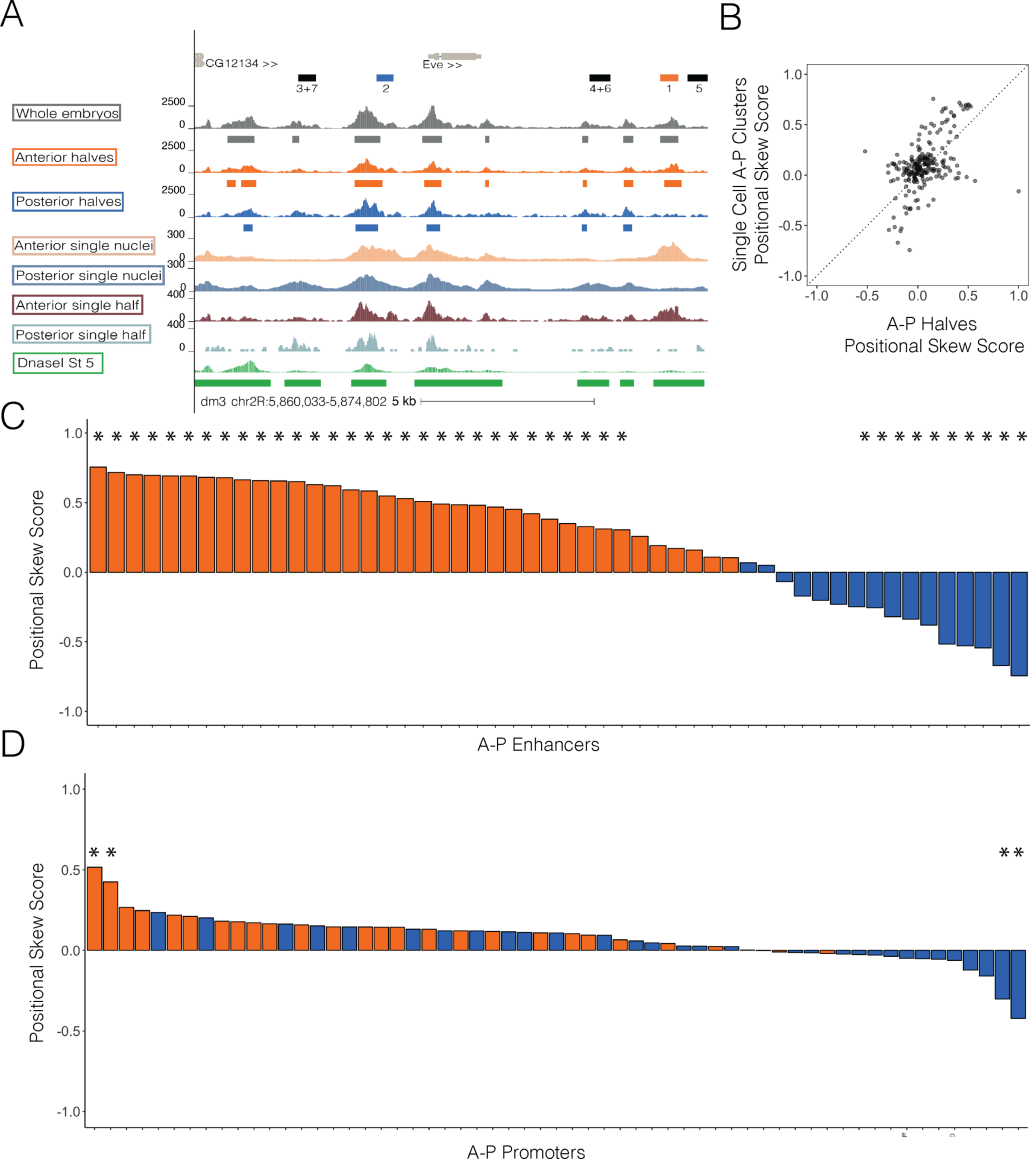
Single nuclei ATAC-seq from cellular blastoderm embryos largely agrees with data from embryo halves. Recently published single nuclei ATAC-seq data [50] corresponding to the cellular blastoderm were separated into anterior and posterior groups following designations determined by [50]. (A) Genome browser trace at the eve locus. Whole embryos, anterior halves, and posterior halves are in grey, orange, and blue respectively. Merged anterior single nuclei and merged posterior single nuclei from [50] are shown in peach and grey blue or cesious. Finally, single anterior and single posterior halves from our study are shown in rusty red and light blue. DnaseI hypersensitivity data is in green. Peaks are depicted in bars below the pooled halves and whole data. Light gray bars denote the gene annotation while the black bars denote annotated enhancers. Colored annotation bars represent enhancers analyzed in Fig 3. (B) Positional skew scores calculated for single nuclei and halves ATAC-seq data at all A-P and D-V patterning regions (enhancers and promoters) are plotted with halves on X and single nuclei on Y. Dotted line indicates X = Y. (C) Bar graph shows the positional skew scores calculated from single nuclei ATAC-seq data at all anterior (orange) and posterior (blue) patterning enhancers in the dataset. Asterisks denote enhancers whose accessibility skew scores show statistical significance over random regions (p < 0.05). (D) Bar graph shows the positional skew scores calculated from single nuclei ATAC-seq data at all anterior (orange) and posterior (blue) patterning promoters in the dataset. Asterisks denote promoters whose accessibility skew scores show statistical significance over random regions (p < 0.05).

We mapped their data to the regions we analyze above and find general agreement with ours (Fig 6). Skew scores computed with their data are correlated with our skew scores, although the skews from single-cell data are more extreme than those from the hand-dissected embryos (Fig 6B). Notably, when these skew scores are plotted around A-P patterning regions, we detect a similar overall skew in the direction of activity at enhancers but not promoters (Fig 6C and 6D). It is interesting that single cell data show greater magnitude of skew score at these regions. This likely reflects the increased spatial precision of single cell methods which are able to subdivide the embryo into anterior and posterior domains by accessibility profile instead of by approximately 50% egg length as we have done. Though there are numerous advantages to single-nucleus experiments, one of the benefits of the experiment reported here is that spatial information about pools of nuclei is determined independently from their accessibility profiles. As such, both experiments taken together reveal that though most genomic regions show similar patterns of accessibility, most A-P patterning enhancers are more accessible in their active half.

The strong anterior skew we and others observe for Bicoid targets is consistent with a recent study showing that chromatin accessibility at a set of around 100 early embryonic enhancers is primarily dependent on Bicoid [19]. Given the strong anterior bias in Bicoid protein levels, it would have been surprising not to find an anterior bias in chromatin accessibility for these regions, although we note that there is significant chromatin accessibility in the posterior for many of these regions in both our data and that of [50], perhaps reflecting activity by the low levels of Bicoid in the posterior [51].

A more comprehensive understanding of how chromatin accessibility is established requires better resolved data on how closely chromatin accessibility tracks with activity in these regions, as neither our data nor that of [50] currently provides greater spatial precision than anterior vs. posterior. There is a large body of literature showing, for example, that tissue specific enhancers often have open chromatin in tissues in which they are not active [52,53], and that this is often a result of enhancer priming by pioneer factors. Furthermore, the transcriptional output of enhancer in the early *Drosophila* embryo [52], and in many other systems, is determined by a balance between the binding of activators and repressors (reviewed in [54]). We expect activators to be bound in parts of the embryo where the enhancer is active, but repressors will bind, by definition, in parts of the embryo where they are repressing enhancer activity. Assuming that open chromatin is associated both with activator and repressor binding implies that the nuclei where chromatin is open for a given enhancer will always be a superset of the nuclei where it is transcriptionally active—the question for the future is how wide these regions are and what their specific patterns tell us about the mechanisms of how they were established.

It will also be interesting to look at the emergence of spatial patterns and biases over time. We have previously shown that nucleosome depletion at enhancers and other aspects of their chromatin state is established prior to the expression of most patterning factors [2]. It has also recently been shown that most enhancers and promoters for patterning genes are accessible by nuclear cycle 11, that this state is maintained through DNA replication and mitosis, and that this open state is associated with ubiquitous factors Zelda and GAF [32]. This leaves open the possibility that the binding of ubiquitous pioneer factors plays a particularly important role in determining chromatin accessibility at early cycles.

In conclusion, our data, as well as that of [19] and [50], establish that though most genomic regions do not show any difference in accessibility, there is significant spatial patterning of chromatin along the anterior-posterior axis in blastoderm embryos, with a clear coupling of activity and accessibility at spatially patterned enhancers. Whether these patterns of chromatin accessibility are instructive for patterning transcription, or merely reflect patterns of activity, remains to be determined.

## Methods and materials

### Fly lines

*Drosophila melanogaster* OregonR embryos were collected for 2 hours and aged for 90 minutes on molasses agar plates. Embryos were then dechorionated with 30%-50% bleach solution for three minutes. Embryos were hand staged at 20x magnification at 14°C to be mitotic cycle 14 (NC14) using previously established methods [2].

### Slicing frozen embryos

NC14 embryos were placed in a custom freezing buffer consisting of ATAC-seq lysis buffer [34] without detergent, 5% glycerol, and 1ul of bromoblue dye. Embryos were then taken out of the freezing buffer and placed onto a glass slide which was then put on dry ice for 2–5 minutes. Once embryos were completely frozen, the glass slide was removed and embryos were sliced with a chilled razorblade. Sliced embryo halves were moved to tubes containing ATAC-seq lysis buffer with 0.15mM spermine added to help stabilize chromatin.

### ATAC-seq on frozen embryo halves

Embryo halves were then homogenized using Kimble Kontes Pellet Pestle (cat no. K749521-1590). IGEPal CA-630 was added to a final concentration of 0.1%. After a 10 minute incubation, nuclei were spun down and resuspended in water. Twenty halves were added to the transposition reaction containing 25ul of 2x TD buffer (Illumina), and 7.5ul of Tn5 enzyme (Illumina). The reaction was incubated at 37°C for 30 minutes. Transposed DNA was purified using Qiagen Minelute kit. Libraries were then amplified using Phusion (NEB cat no. F531S) and Illumina Nextera index kit (cat no. FC-121-1011). Libraries were then purified with Ampure Beads at a 1.2: 1 beads to sample ratio and sequenced on the Hiseq4000 using 100bp paired end reads. Fragments over 500bp were removed from libraries using a Pippen prep to reduce sequencing bias with the Hiseq4000.

### ATAC-seq data preprocessing and normalization

Fastq files were aligned to *Drosophila* dm3 genome with Bowtie2 using the following parameters -5 5–3 5 -N 1 -X 2000—local—very-sensitive-local. Mapping metrics are provided in supplementary S2 Table. Sam files were then sorted and converted to Bam files using Samtools, only keeping uniquely mapped reads with a MAPQ score of 30 or higher using -q 30, proper pairs with -f 2, and removing unmapped, not primary alignment, reads that fail platform vendor quality checks, and optical duplicates with sam flag -F 1804. Duplicates were removed with Picard (http://broadinstitute.github.io/picard/). Bams were then converted to bed files with bedtools [55] and shifted using a custom shell script to reflect a 4bp increase on the plus strand and a 5bp decrease on the minus strand as recommended by [34]. Replicate bed files were merged. Finally shifted bed files were converted into wig files using custom scripts (S3 File) and wig files which were uploaded to the UCSC genome browser. Merged wig files were normalized to reflect 10 million mapped *Drosophila melanogaster* reads. Anterior and posterior samples were normalized by linear regression to the whole embryo sample not including the y intercept (S1 Fig).

### A-P and D-V patterning enhancer and promoter annotations

A-P and D-V patterning enhancer and gene annotations were compiled from many sources (S1 File) [28,37–48,56]. In order to provide the most accurate promoter annotations possible for our analysis we used RACE, CAGE, and EST data performed in *Drosophila melanogaster* embryos [57] to identify promoters preferentially used by the fly embryo. When there were multiple promoters per gene (as was frequently seen), we chose the promoter that was verified by all three methods, denoted by a “V” in Hoskins et. al. (2011) supplementary file 3. There were three genes that did not have annotated promoters in the Hoskins et al. (2011) dataset that were used in our analysis. Instead, these promoter annotations came from the Eukaryotic Promoter Database converted to dm3 annotations [58,59].

In order to further validate our A-P and D-V patterning enhancer and promoter annotations we manually curated *in situ* hybridization images corresponding to 678 genomic regions from multiple sources [38,42,46–48,60–83]. Each region was manually inspected such that only regions with both an *in situ* hybridization image that shows spatially restricted expression as well as moderate accessibility signal (wig signal > 200) were kept for further analysis leaving 253 enhancers and promoters with spatially restricted expression. Anterior and posterior patterning enhancers and promoters were categorized as either completely spatially restricted or mostly spatially restricted. A report PDF for each region, including *in situ* hybridization images, accessibility browser traces, and Z-score and p-value, are found in S2 File.

Promoters used in our analysis were categorized as maternal, maternal-zygotic, or zygotic using previously published RNA-seq data from single embryos [11]. Only zygotically expressed gene promoters were used in Fig 4C. However, all classes of promoters were used in the rest of Fig 4.

### Differential ATAC-seq analysis

All graphs were made with R scripts (S3 File) and Deeptools [84]. Accessibility skew score was calculated by the following equation:

$$\frac{X_{active}-X_{inactive}}{X_{active}+X_{inactive}}$$ (1)

where X_active_ is the wig signal in the half where the region is activating gene expression and X_inactive_ is wig signal in the half where the region is not supposed to activate gene expression. Accessibility skew score measures whether a region is differentially accessible in the expected direction. This score is useful when comparing differential accessibility regardless of which half is favored (for example when comparing accessibility skew at anterior to posteriorly patterned regions).

Positional skew score provides information about the direction of the skew such that regions that are more accessible in the anterior have a positive positional skew score while those that are more accessible in the posterior have a negative positional skew score. Positional skew score is calculated by the following equation:

$$\frac{X_{anterior}-X_{posterior}}{X_{anterior}+X_{posterior}}$$ (2)

Where X_anterior_ is the wig signal in the anterior sample and X_posterior_ is the wig signal in the posterior sample. Significance for each region was determined by computationally matching each region to a random region that has the same total normalized wig score (S10 Fig). Positional skew score was calculated for each random region (termed RandSkewScore). These scores were distributed normally and were used to determine a Z-score for each region of interest (Z_ROI_) by the following equation:

z_ROI_ = $\frac{AccessibilitySkewScore-\mu }{\sigma }$ where μ is the mean of the random region distribution and σ is the standard deviation of the random region distribution. Two-tailed p-values were then calculated from the Z score.

### Peaks

Replicates were merged and peaks were called on the merged bed file using MACS2 with the following parameters:—nomodel—call-summits—bdg -p 1e-3. Reproducible peaks were selected using bedtools to intersect peaks called in both replicates and the merged samples. Anterior and posterior accessibility signal was averaged using custom scripts around the set of reproducible whole peaks and positional skew scores were calculated for each peak region as described above. Significantly skewed peaks were determined using random regions as with A-P patterning regions. Significantly skewed anterior and posterior peaks as well as unskewed peaks were intersected with experimentally derived promoters, Kvon predicted enhancers [45], and transcription factor ChIP-seq binding data [15,49,85]. One-tailed fisher exact tests were performed to determine whether there was a significant depletion or enrichment of transcription factor peaks in significantly skewed accessibility peaks. Regulatory sequence analysis tools (RSAT) peak motif tool was used to find motifs in each peak set.

### ChIP-seq data analysis

Wig files from previously published ChIP-seq data were obtained for Kruppel, Hunchback, Giant, Knirps, Caudal, Bicoid [15,49,85], and Zelda data from stage 3, 4, and 5 embryos [20]. Wig files were normalized by the mean signal for each sample, assuming that the mean signal over the entire genome is similar to that of background. This normalization essentially transforms the data into deviations from the mean such that signal from different experiments can be compared to each other. Wig signal around regions of interest was determined and graphed in R (S3 File). For the heat maps, normalized wig signal was averaged over 3kb windows around regions of interest for each factor before being scaled such that the region with the highest value is equal to 1 and the lowest to 0 for each factor.

### DNaseI data analysis

For all DnaseI comparisons to ATAC-seq (S2 Fig and Fig 5), previously published DnaseI data from stage 5 embryos (replicate 1) normalized to 10 million reads was used. For S4 Fig, DNaseI data was downloaded from the following SRA datasets: SRA:SRP002474.1, SRA:SRX020691.4, SRA:SRX020692.1, SRA:SRX020693.1, SRA:SRX020694.1, SRA:SRX020695.1, SRA:SRX020696.1, SRA:SRX020697.1, SRA:SRX020698.1, SRA:SRX020699.1, SRA:SRX020700.1, SRA:SRX041410. Reads were processed similarly to ATAC-seq data. Reads were aligned to the dm3 genome with Bowtie2 with the following parameters -p 10–5 5–3 5 -N 1 -X 2000—local—very-sensitive-local. Sam files were then sorted and converted to Bam files using Samtools using the same filters as ATAC-seq samples. Duplicates were removed with Picard. Bams were then converted to bed files using Bedtools and converted into wig files using custom scripts (S1 Code). All replicates were merged and were normalized to 10 million mapped reads.

### Single nuclei ATAC-seq analysis

Bam files labeled to correspond with published clusters were shared by [50] with the authors. Bam files corresponding to anterior and posterior clusters were merged respectively. Merged bam files were then converted into wig files and then normalized to 1 million reads. The anterior wig file was then normalized by linear regression to the posterior wig file. Positional skew score was measured for all A-P and D-V patterning regions in the same manner as was done for halves ATAC-seq data (S4 File).

## Supporting information

S1 Fig Linear regression normalization. Scatter plots showing 1kb genomic bins (gray) with 2D density plots (light blue) indicating areas of increased point density. The X = Y line is indicated by a dashed line. Linear regression model is indicated by a solid dark blue line. Anterior and posterior halves were normalized to whole samples. (A,C) Scatter plots show data before normalization. (B,D) Scatter plots show data after normalization. Spearman correlation coefficients (r_s_), Pearson correlation coefficients (r_p_), and r squared values are shown above each plot.
(TIF)

S2 Fig ATAC-seq on embryo halves correlates highly with DNaseI and whole embryos. **(**A) Scatter plots showing merged normalized wig signal values from ATAC-seq experiments performed on anterior halves, posterior halves, and whole embryos compared with DNase-I hypersensitivity from stage 5 *Drosophila melanogaster* embryos [35] binned into 1kb regions. Spearman correlation coefficients (r_s_), Pearson correlation coefficients (r_p_), and r squared values are shown above each plot. The line X = Y is shown as a dotted line. (B) Scatter plots showing wig signal values for anterior, posterior, and whole replicates normalized to 1 million reads. (C) Normalized wig signal from ATAC-seq data for the combined anterior and posterior halves sample compared to the merged whole embryo sample.
(TIF)

S3 Fig Chromatin accessibility in anterior and posterior halves is highly correlated at whole embryo peaks. (A) Scatter plots showing normalized ATAC-seq signal in anterior (x axis) and posterior (y axis) pooled halves at whole embryo accessibility peaks (gray). Spearman correlation coefficients (r_s_), Pearson correlation coefficients (r_p_), and r squared values are shown above each plot. The line X = Y is shown as a dotted line. Light blue circles denote point density.
(TIF)

S4 Fig Chromatin accessibility changes during development. Scatter plots showing merged normalized wig signal values from DNaseI hypersensitivity experiments on stage 5,9,11, and 14 *Drosophila melanogaster* embryos [35] binned into 1kb regions. Spearman correlation coefficients (r_s_), Pearson correlation coefficients (r_p_), and r squared values are shown above each plot. The line X = Y is shown as a dotted line. 2D density plot (light blue) indicates areas of increased point density.
(TIF)

S5 Fig D-V patterning enhancers and promoters are similarly accessible in both halves. Bar graphs showing positional skew score calculated for dorsal (purple) and ventral (green) patterning enhancers and promoters. The enhancer or promoter names are below the graph. (A) D-V enhancers (B) D-V promoters.
(TIF)

S6 Fig Replicate and merged positional skew scores at A-P enhancers. The bar graph represents positional skew scores calculated from the merged replicate samples for all anterior (orange) and posterior (blue) patterning enhancers in the dataset (S1 File). Dots show the positional skew score calculated for both biological replicates.
(TIF)

S7 Fig Single anterior and posterior halves show similar trends as pooled embryo halves. (A) Scatter plots showing normalized ATAC-seq signal in anterior (x axis) and posterior (y axis) single halves at 1kb adjacent windows tiling the genome (gray). Spearman correlation coefficients (r_s_), Pearson correlation coefficients (r_p_), and r squared values are shown above each plot. The line X = Y is shown as a dotted line. (B) Bar graph shows the positional skew scores calculated for all anterior (orange) and posterior (blue) patterning enhancers in the dataset (File S1) and for dorsal (purple) and ventral (green) patterning enhancers. (C) Bar graph shows the positional skew scores calculated for all anterior (orange) and posterior (blue) patterning promoters in the dataset (File S1) and for dorsal (purple) and ventral (green) patterning promoters. The region names are below each graph.
(TIF)

S8 Fig Significantly skewed peaks show similar differences in transcription factor binding and are predictive of enhancer activity. (A) ChIP-seq data for Bicoid, Caudal, Knirps, Giant, Hunchback, Kruppel, and Zelda from three stages (stage 3,4, and 5) from [20,49] normalized to the mean of each factor and scaled between 0 and 1 summed over a 3kb window around each reproducible whole peak. White represents the minimum signal and black represents the maximum ChIP signal for that transcription factor. Above each heat map is a colored bar that represents the positional skew score for each reproducible whole peak with orange representing peaks that are more accessible in the anterior, blue representing those that are more accessible in the posterior, and white representing those that are not differentially accessible between the two halves. Peaks are ordered by positional skew score. (B) Positional skew scores were calculated for reproducible whole peaks and z-scores and p-values were determined using random region distributions like for patterning regions. Peaks were then divided into three categories based on skew score and p-value. AntSig peaks are those that are significantly more accessible in the anterior (positive positional skew score and p-value < 0.05). PostSig peaks are those that are significantly more accessible in the posterior (negative positional skew score and p-value < 0.05). NotSig peaks are those that are not significantly skewed in any direction (p-value > 0.05). These three groups of peaks were then intersected using Bedtools with peaks called on ChIP-seq data from (A). One-tailed Fisher’s exact tests were performed on these overlaps and the resulting odds ratio are depicted on the logarithmically transformed Y axis such that negative values represent depletions of transcription factor binding in whole peaks and positive values represent enrichments. Asterisks indicate odds ratios that are significant. (C) Transcription factor motifs were searched for in AntSig, PostSig, and NotSig peaksets (methods). The top five predicted motifs are shown with associated number of sites, e-values, and predicted factor match. (D) Replicated whole peaks were intersected with known Kvon enhancers [45]. Matches were then filtered for ones that had annotated expression in stage 4–6 embryos. 12 matches remained. For each of these 12, published slides from *in situ* hybridization experiments were analyzed for stage 5 embryos which are depicted below the bar graph. Using these *in situ* images, the location of activity was determined (represented by bar color—orange represents those active in the anterior, blue represents those active in the posterior, purple represents activity in both halves, gray represents no visible expression). Y-axis depicts positional skew scores calculated for each matching peak region.
(TIF)

S9 Fig Transcription factor binding and differential accessibility at A-P patterning enhancers. ChIP-seq data for Bicoid, Caudal, Knirps, Giant, Hunchback, Kruppel, and Zelda from three stages (stage 3,4, and 5) from [20,49] normalized to the mean of each factor and scaled between 0 and 1 summed over a 3kb window around each A-P enhancer. White represents the minimum signal and black represents the maximum ChIP signal for that transcription factor. Above each heat map is a colored bar that represents the positional skew score for each A-P enhancer with orange representing enhancers that are more accessible in the anterior, blue representing those that are more accessible in the posterior, and white representing those that are not differentially accessible between the two halves. (A) Enhancers are ordered by positional skew score. (B) Enhancers are hierarchically clustered using complex heat map package in R [89].
(TIF)

S10 Fig Positional skew scores of enhancers and promoters compared to random regions. Histograms showing the distribution of positional skew scores of random regions compared to (A) A-P patterning enhancers (B) D-V patterning enhancers (C) A-P patterning promoters (D) D-V patterning promoters. Anterior is orange, posterior is blue, dorsal is purple, ventral is green. (E) Histogram showing the distribution of random regions with the fitted normal curve in black. Mu and std from the normal curve are shown above the graph.
(TIF)

S1 Table Comparisons between A-P and D-V enhancer and promoter mean positional skew scores using Tukey HSD (multiple testing correction). Pairwise comparisons of mean positional skew score across the different predicted locations (anterior, posterior, dorsal, ventral) as well as sets of random genomic regions chosen to mirror the same total signal distribution of the tested regions. The table is derived from the output of the functions below that can also be found in the supplemental code file (S4 File).
Anova_Enhancers_lm = lm(as.numeric(Anova_Enhancers_dataframe$ATACSkewScore) ~
Anova_Enhancers_dataframe$Location)
Anova_Enhancer = aov(formula = as.numeric(Anova_Enhancers_dataframe$ATACSkewScore) ~
Anova_Enhancers_dataframe$Location)
Tukey_Enhancers = TukeyHSD(Anova_Enhancer)
write.table(Tukey_Enhancers$`Anova_Enhancers_dataframe$Location`,
"020118_Anova_ALLLocations_Enhancers.txt")
The output of the Tukey HSD function supplies the identity of the pair-wise comparison, “diff” which is the difference in the observed means, “lwr” which is the lower end point for the interval, “upper” is the upper end point for the interval, and “p adj” is the p-value after adjustments are made for multiple comparisons.
(XLSX)

S2 Table ATAC-seq halves mapping metrics. Cells A3 to D1l show the number of reads that were aligned by Bowtie2 (methods). B3 to B9 contain the total number of reads for each sample. C3 to C9 show the number of concordant read pairs that aligned. D3 to D11 shows the overall alignment rate (including multiple and discordant reads) to represent what percent of the sample came from our expected genome (*Drosophila melanogaster*). Rows 13 to 34 shows the read number changes after filtering out unmapped, not primary alignment, duplicates, failed platform checks, and improper pairs. Rows 23 to 31 show read numbers of each sample that are less than 130 bp. C14 to C34 shows the normalization factor used to normalize each sample to the number in column D (1 million reads for all replicate samples and 10 million reads for samples where the replicates are merged).
(XLSX)

S1 File Average signal and ATAC-seq analysis for A-P and D-V patterning enhancers and promoters—all replicates. This file contains all of the patterning regions used to make Figs 2,3,4 and 5. These are regions that were filtered to overlap a peak and have spatial localization of activity confirmed by *in situ* hybridization experiments. The values in each column is described in the table included in S1 File.
(XLSX)

S2 File Random region file. Random genomic regions chosen to correspond to A-P and D-V combined patterning enhancers and promoters. File columns are as included in the file.
(XLSX)

S3 File Report file for all patterning regions used in Figs 3 and 4. Reports consist of *in situ hybridization* images, ATAC-seq traces, and calculated p-value and Z Score for each region used in the final analysis.
(ZIP)

S4 File Scripts and code. HTML file of all scripts used in the final analysis.
(HTML)

S1 Text Supplementary references. (DOCX)
