# Supplementary figures and images for: Patterns of chromatin accessibility along the anterior-posterior axis in the early Drosophila embryo (part 1 of 2)
Source: PLoS Genet. 2018 May 4;14(5):e1007367. doi: 10.1371/journal.pgen.1007367 (PMC5955596; doi:10.1371/journal.pgen.1007367)

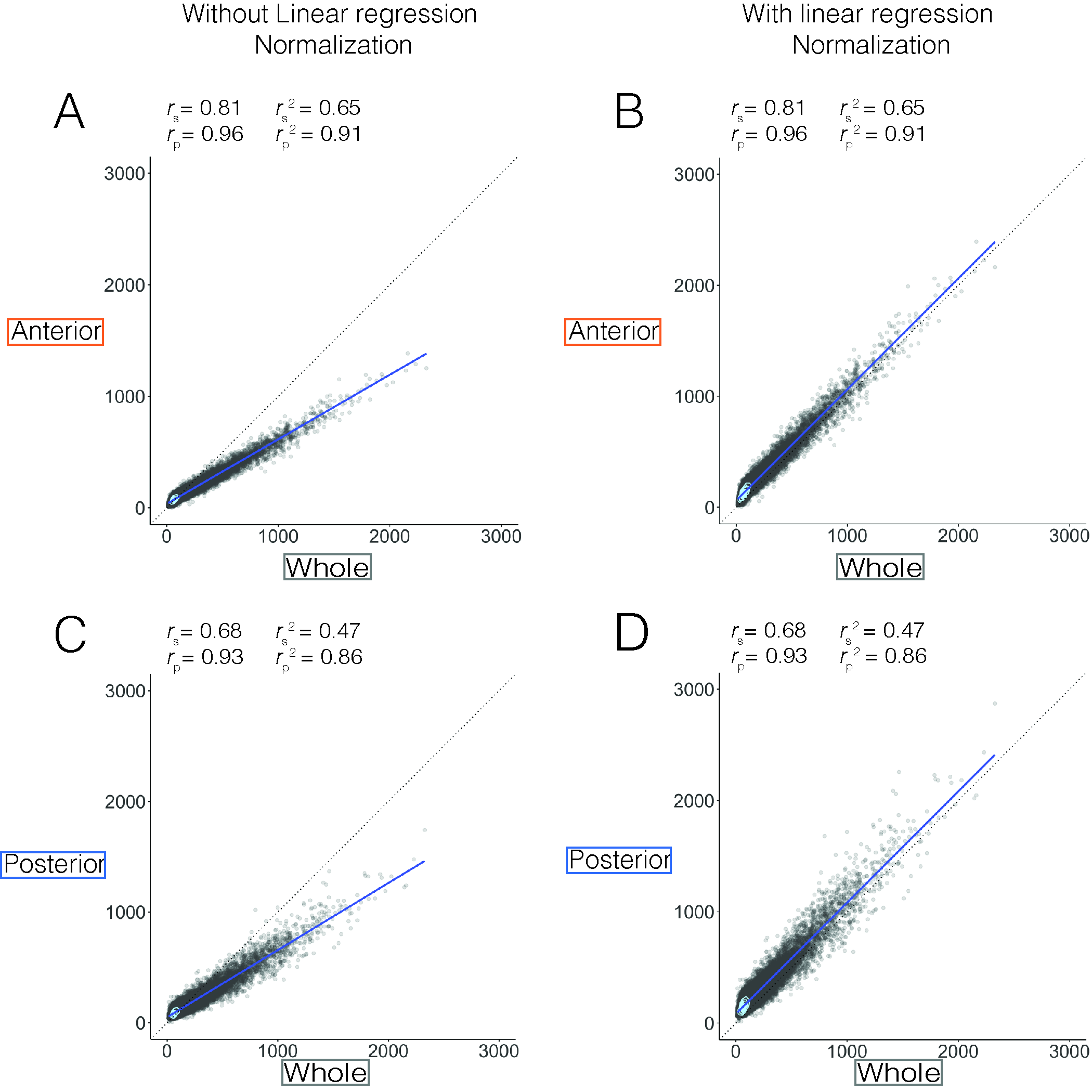

Supplement: S1 Fig — Scatter plots showing 1kb genomic bins (gray) with 2D density plots (light blue) indicating areas of increased point density. The X = Y line is indicated by a dashed line. Linear regression model is indicated by a solid dark blue line. Anterior and posterior halves were normalized to whole samples. (A,C) Scatter plots show data before normalization. (B,D) Scatter plots show data after normalization. Spearman correlation coefficients (rs), Pearson correlation coefficients (rp), and r squared values are shown above each plot. (TIF) [file pgen.1007367.s001.tif]

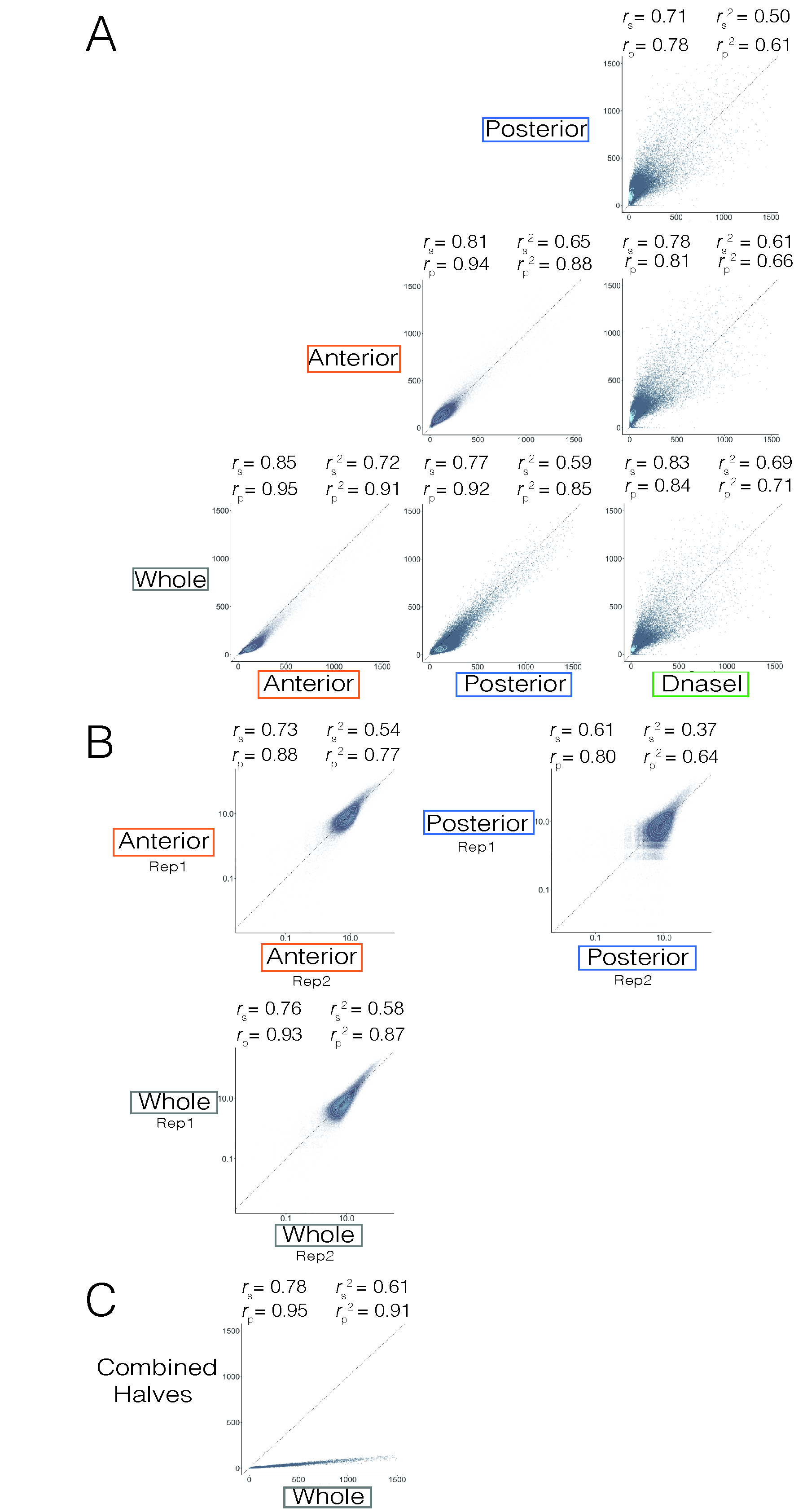

Supplement: S2 Fig — (A) Scatter plots showing merged normalized wig signal values from ATAC-seq experiments performed on anterior halves, posterior halves, and whole embryos compared with DNase-I hypersensitivity from stage 5 Drosophila melanogaster embryos [35] binned into 1kb regions. Spearman correlation coefficients (rs), Pearson correlation coefficients (rp), and r squared values are shown above each plot. The line X = Y is shown as a dotted line. (B) Scatter plots showing wig signal values for anterior, posterior, and whole replicates normalized to 1 million reads. (C) Normalized wig signal from ATAC-seq data for the combined anterior and posterior halves sample compared to the merged whole embryo sample. (TIF) [file pgen.1007367.s002.tif]

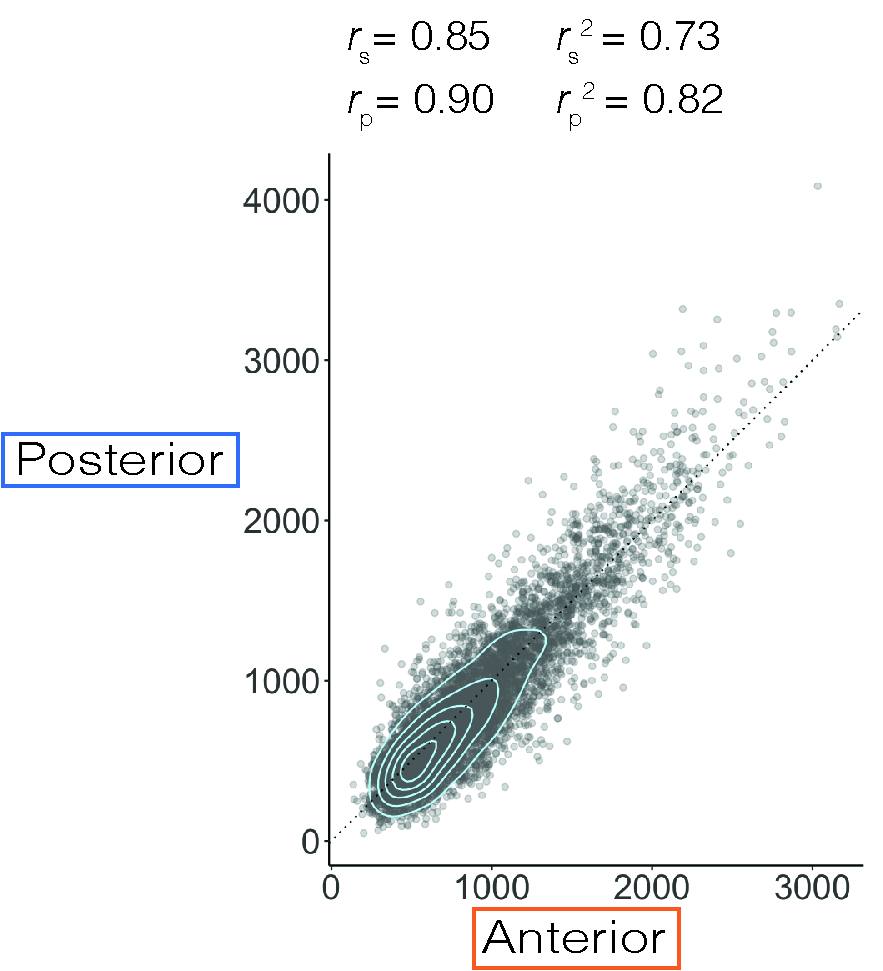

Supplement: S3 Fig — (A) Scatter plots showing normalized ATAC-seq signal in anterior (x axis) and posterior (y axis) pooled halves at whole embryo accessibility peaks (gray). Spearman correlation coefficients (rs), Pearson correlation coefficients (rp), and r squared values are shown above each plot. The line X = Y is shown as a dotted line. Light blue circles denote point density. (TIF) [file pgen.1007367.s003.tif]

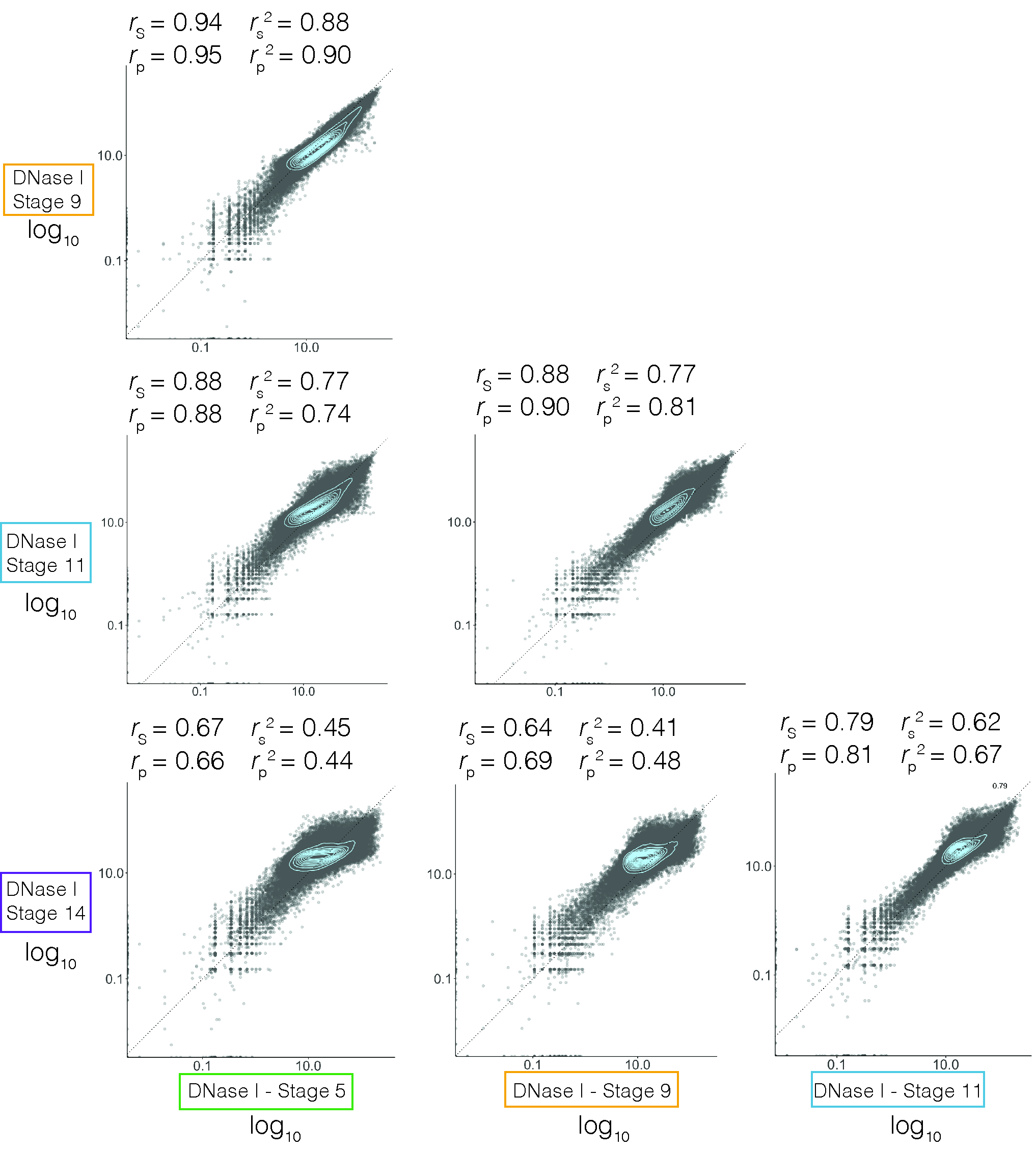

Supplement: S4 Fig — Scatter plots showing merged normalized wig signal values from DNaseI hypersensitivity experiments on stage 5,9,11, and 14 Drosophila melanogaster embryos [35] binned into 1kb regions. Spearman correlation coefficients (rs), Pearson correlation coefficients (rp), and r squared values are shown above each plot. The line X = Y is shown as a dotted line. 2D density plot (light blue) indicates areas of increased point density. (TIF) [file pgen.1007367.s004.tif]

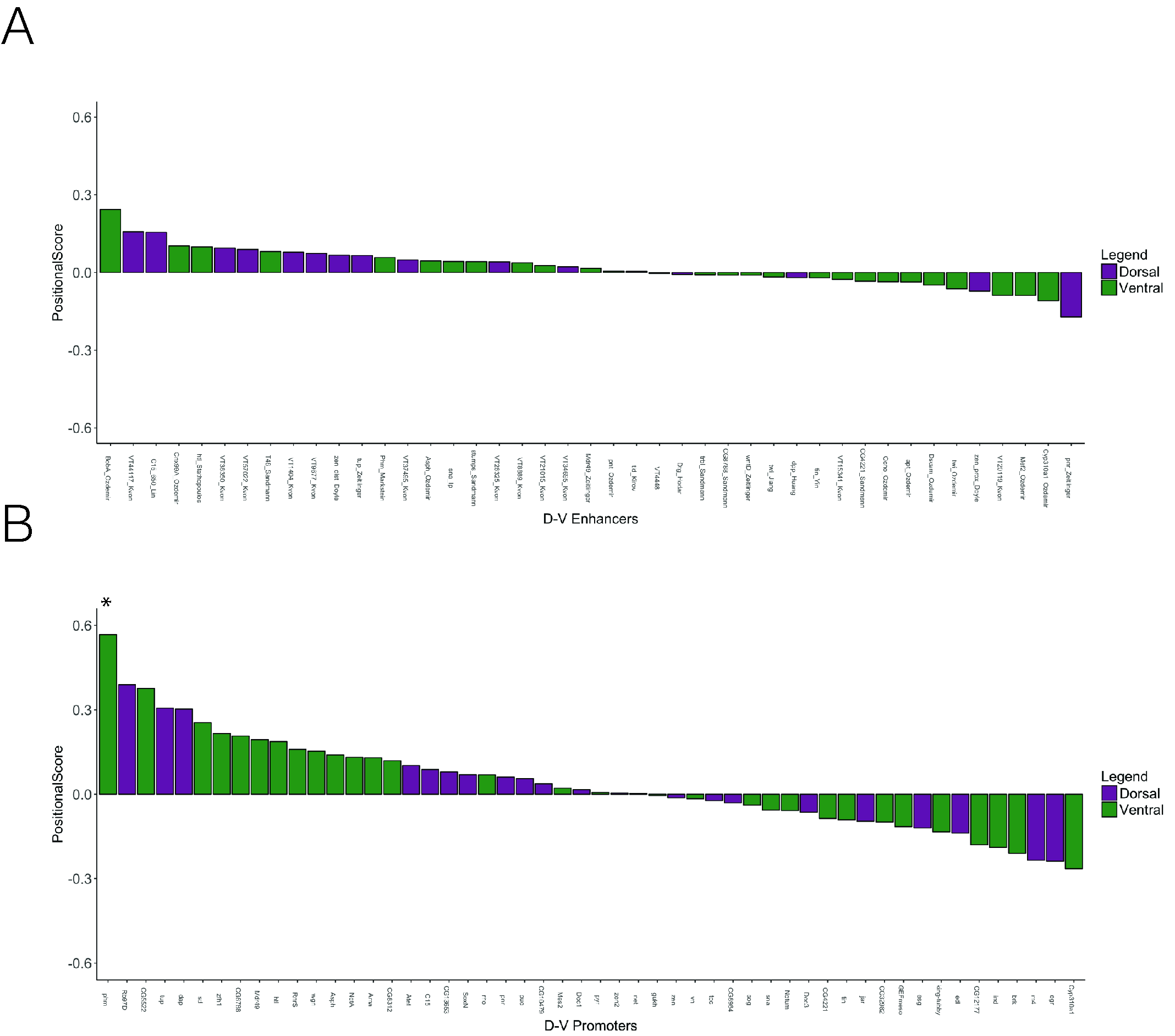

Supplement: S5 Fig — Bar graphs showing positional skew score calculated for dorsal (purple) and ventral (green) patterning enhancers and promoters. The enhancer or promoter names are below the graph. (A) D-V enhancers (B) D-V promoters. (TIF) [file pgen.1007367.s005.tif]

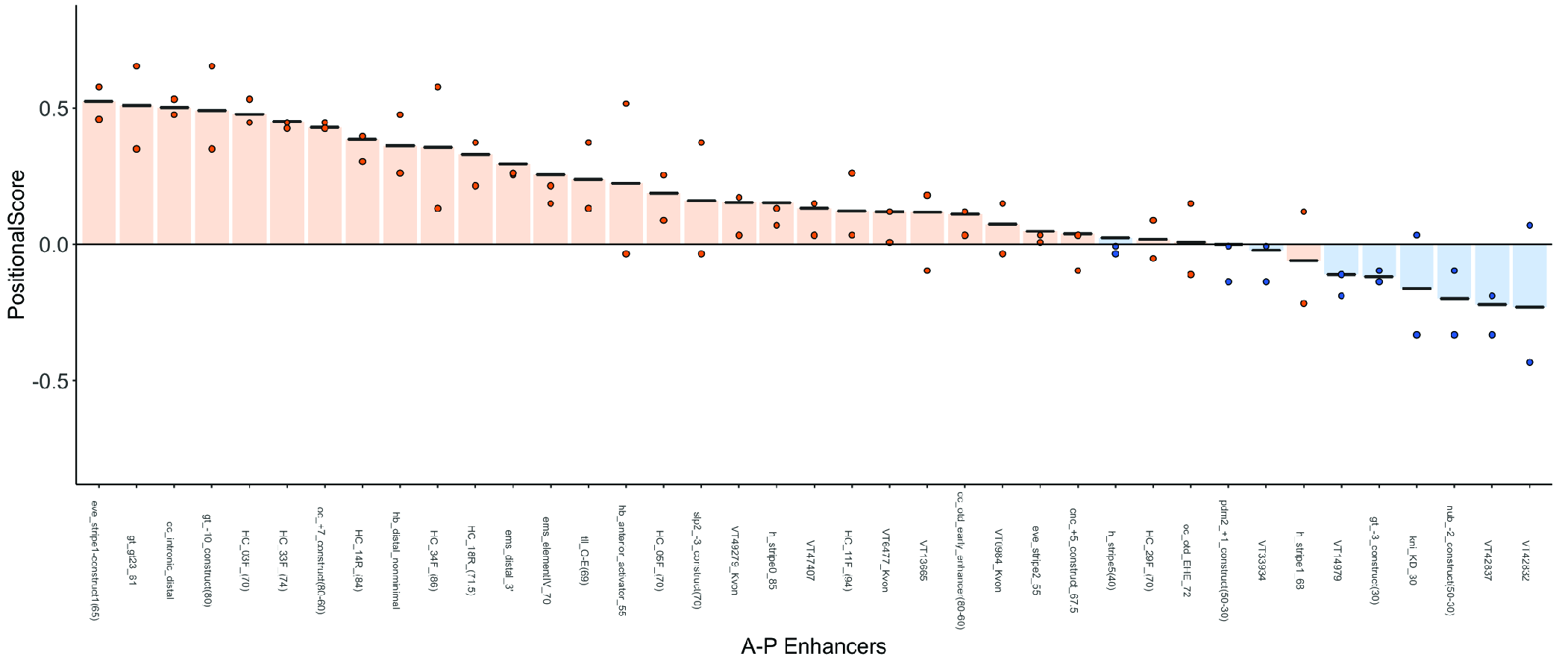

Supplement: S6 Fig — The bar graph represents positional skew scores calculated from the merged replicate samples for all anterior (orange) and posterior (blue) patterning enhancers in the dataset (S1 File). Dots show the positional skew score calculated for both biological replicates. (TIF) [file pgen.1007367.s006.tif]

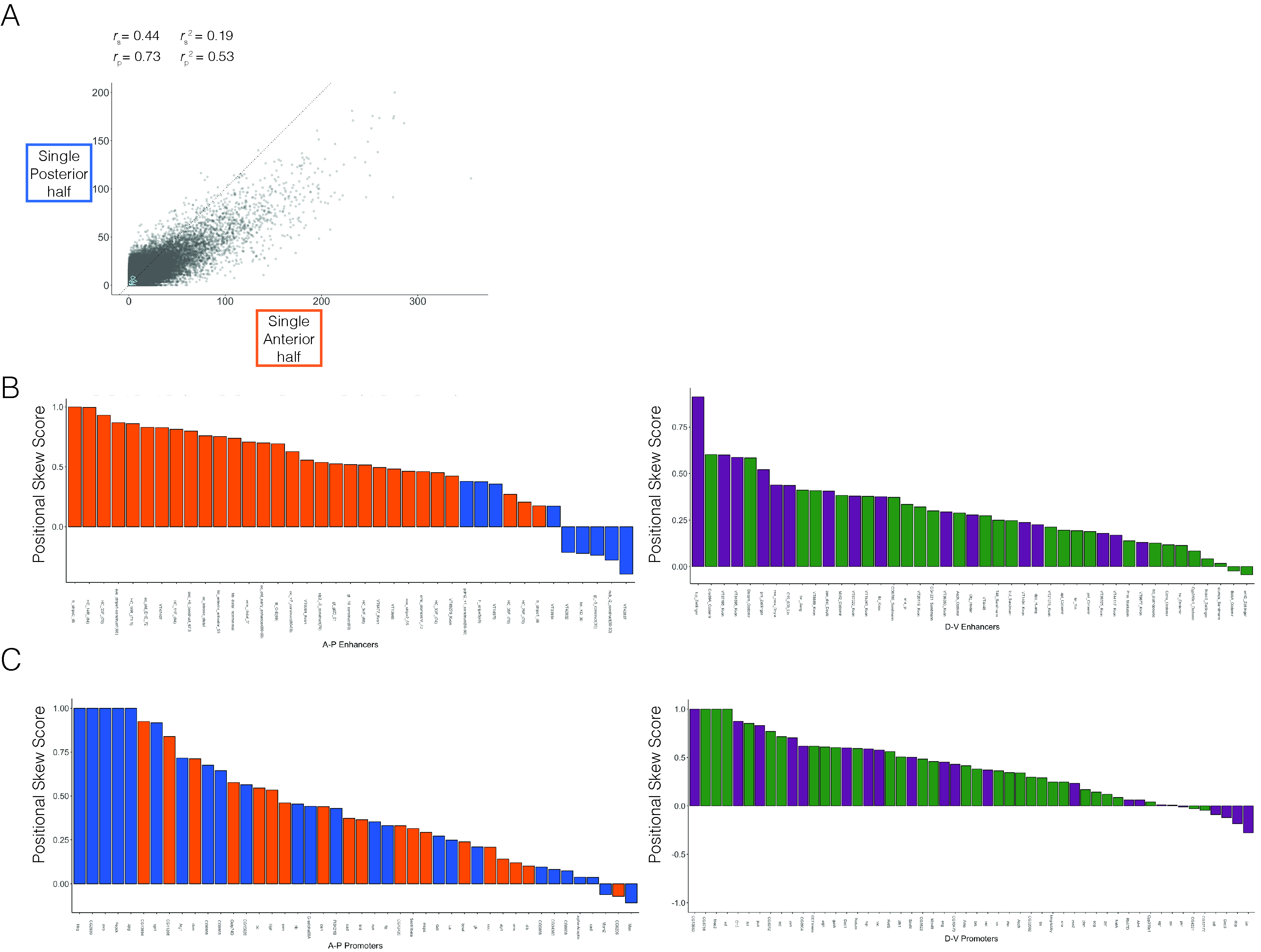

Supplement: S7 Fig — (A) Scatter plots showing normalized ATAC-seq signal in anterior (x axis) and posterior (y axis) single halves at 1kb adjacent windows tiling the genome (gray). Spearman correlation coefficients (rs), Pearson correlation coefficients (rp), and r squared values are shown above each plot. The line X = Y is shown as a dotted line. (B) Bar graph shows the positional skew scores calculated for all anterior (orange) and posterior (blue) patterning enhancers in the dataset (File S1) and for dorsal (purple) and ventral (green) patterning enhancers. (C) Bar graph shows the positional skew scores calculated for all anterior (orange) and posterior (blue) patterning promoters in the dataset (File S1) and for dorsal (purple) and ventral (green) patterning promoters. The region names are below each graph. (TIF) [file pgen.1007367.s007.tif]

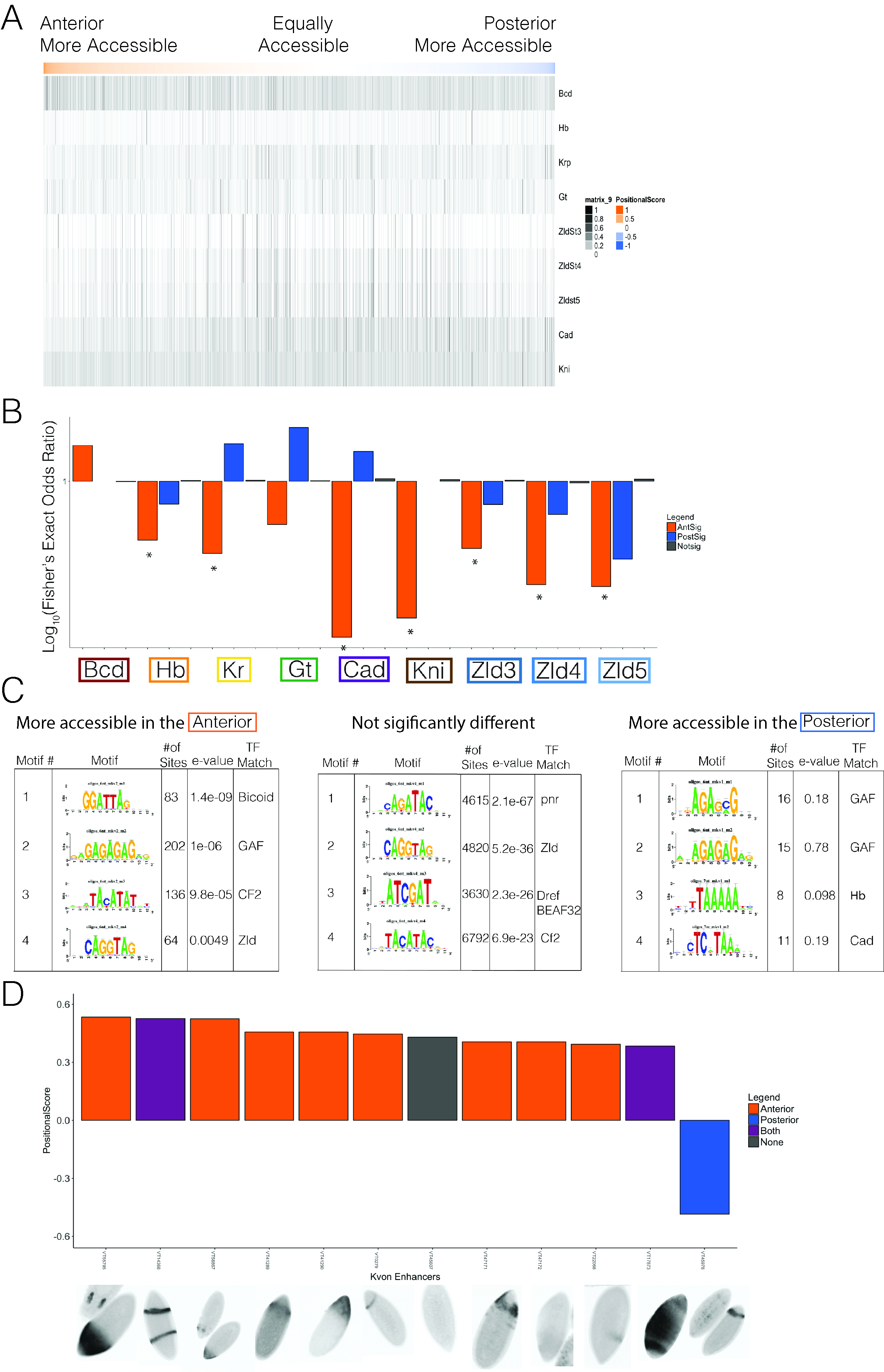

Supplement: S8 Fig — (A) ChIP-seq data for Bicoid, Caudal, Knirps, Giant, Hunchback, Kruppel, and Zelda from three stages (stage 3,4, and 5) from [20,49] normalized to the mean of each factor and scaled between 0 and 1 summed over a 3kb window around each reproducible whole peak. White represents the minimum signal and black represents the maximum ChIP signal for that transcription factor. Above each heat map is a colored bar that represents the positional skew score for each reproducible whole peak with orange representing peaks that are more accessible in the anterior, blue representing those that are more accessible in the posterior, and white representing those that are not differentially accessible between the two halves. Peaks are ordered by positional skew score. (B) Positional skew scores were calculated for reproducible whole peaks and z-scores and p-values were determined using random region distributions like for patterning regions. Peaks were then divided into three categories based on skew score and p-value. AntSig peaks are those that are significantly more accessible in the anterior (positive positional skew score and p-value < 0.05). PostSig peaks are those that are significantly more accessible in the posterior (negative positional skew score and p-value < 0.05). NotSig peaks are those that are not significantly skewed in any direction (p-value > 0.05). These three groups of peaks were then intersected using Bedtools with peaks called on ChIP-seq data from (A). One-tailed Fisher’s exact tests were performed on these overlaps and the resulting odds ratio are depicted on the logarithmically transformed Y axis such that negative values represent depletions of transcription factor binding in whole peaks and positive values represent enrichments. Asterisks indicate odds ratios that are significant. (C) Transcription factor motifs were searched for in AntSig, PostSig, and NotSig peaksets (methods). The top five predicted motifs are shown with associated number of sites, e-val [file pgen.1007367.s008.tif]

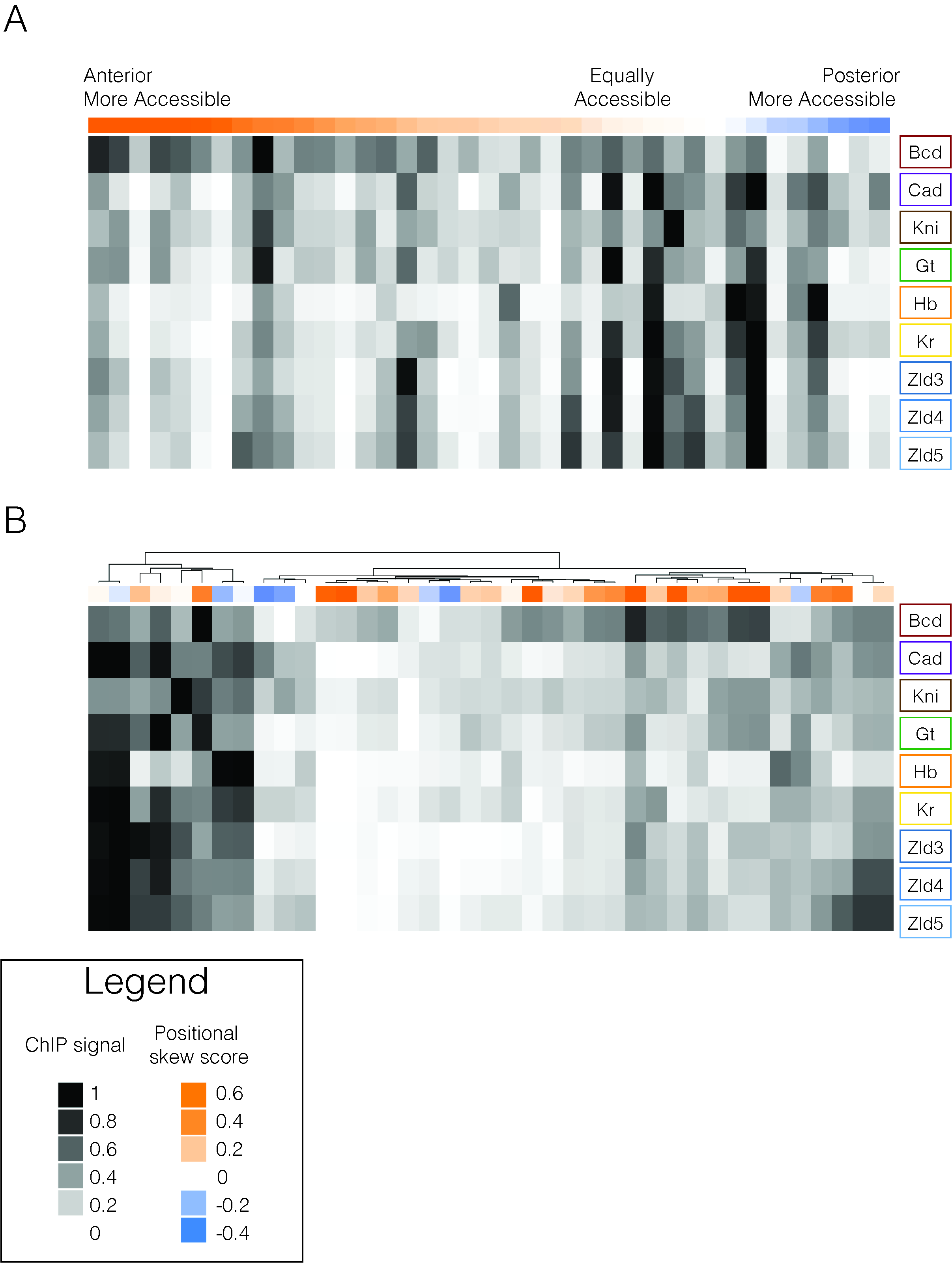

Supplement: S9 Fig — ChIP-seq data for Bicoid, Caudal, Knirps, Giant, Hunchback, Kruppel, and Zelda from three stages (stage 3,4, and 5) from [20,49] normalized to the mean of each factor and scaled between 0 and 1 summed over a 3kb window around each A-P enhancer. White represents the minimum signal and black represents the maximum ChIP signal for that transcription factor. Above each heat map is a colored bar that represents the positional skew score for each A-P enhancer with orange representing enhancers that are more accessible in the anterior, blue representing those that are more accessible in the posterior, and white representing those that are not differentially accessible between the two halves. (A) Enhancers are ordered by positional skew score. (B) Enhancers are hierarchically clustered using complex heat map package in R [89]. (TIF) [file pgen.1007367.s009.tif]

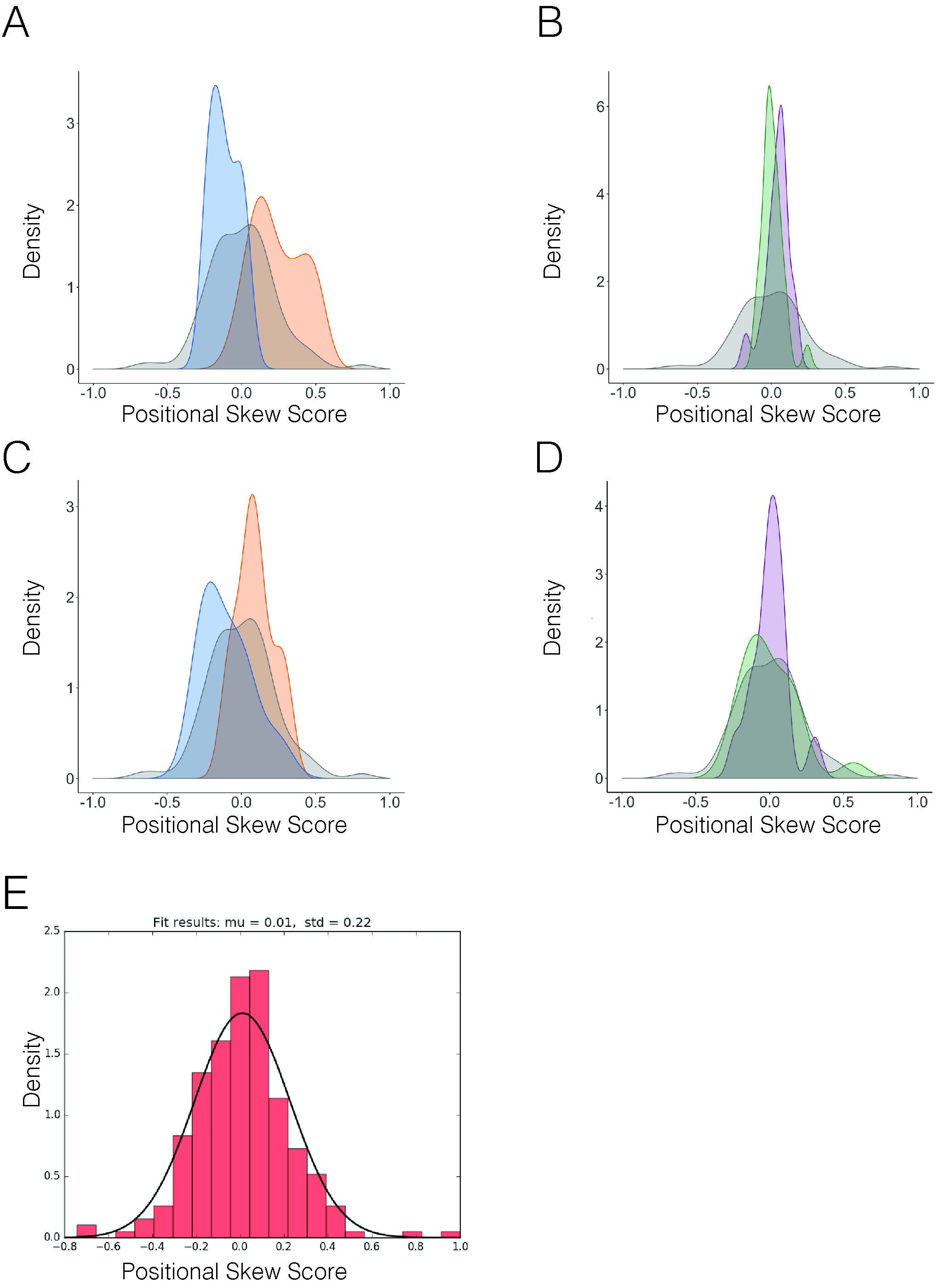

Supplement: S10 Fig — Histograms showing the distribution of positional skew scores of random regions compared to (A) A-P patterning enhancers (B) D-V patterning enhancers (C) A-P patterning promoters (D) D-V patterning promoters. Anterior is orange, posterior is blue, dorsal is purple, ventral is green. (E) Histogram showing the distribution of random regions with the fitted normal curve in black. Mu and std from the normal curve are shown above the graph. (TIF) [file pgen.1007367.s010.tif]

# alpha-Adaptin

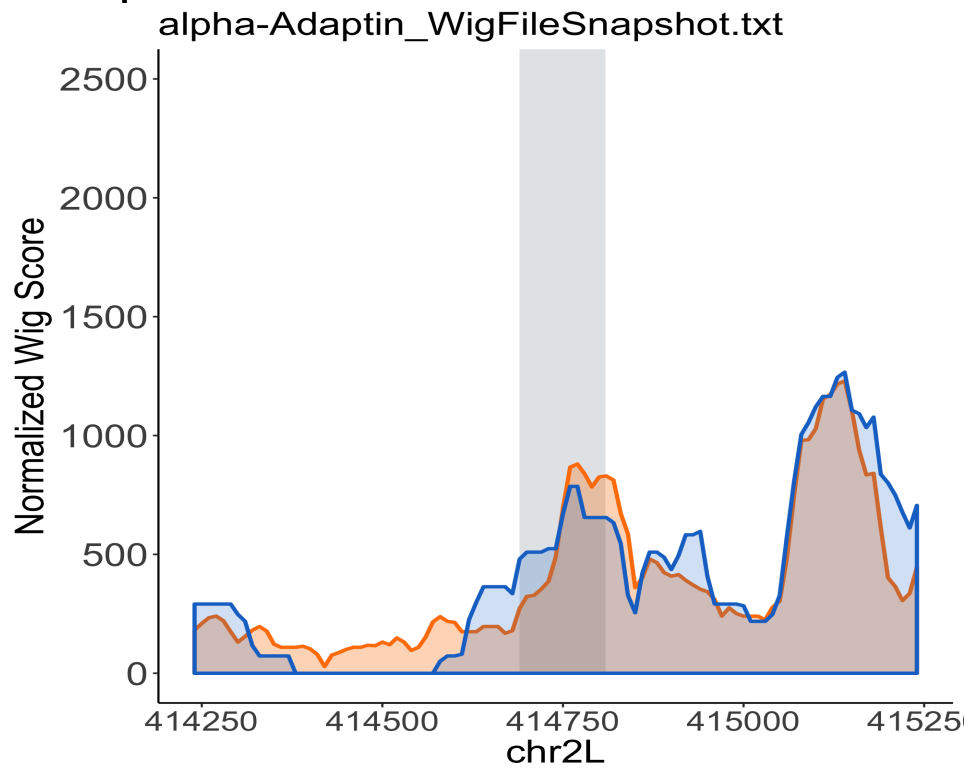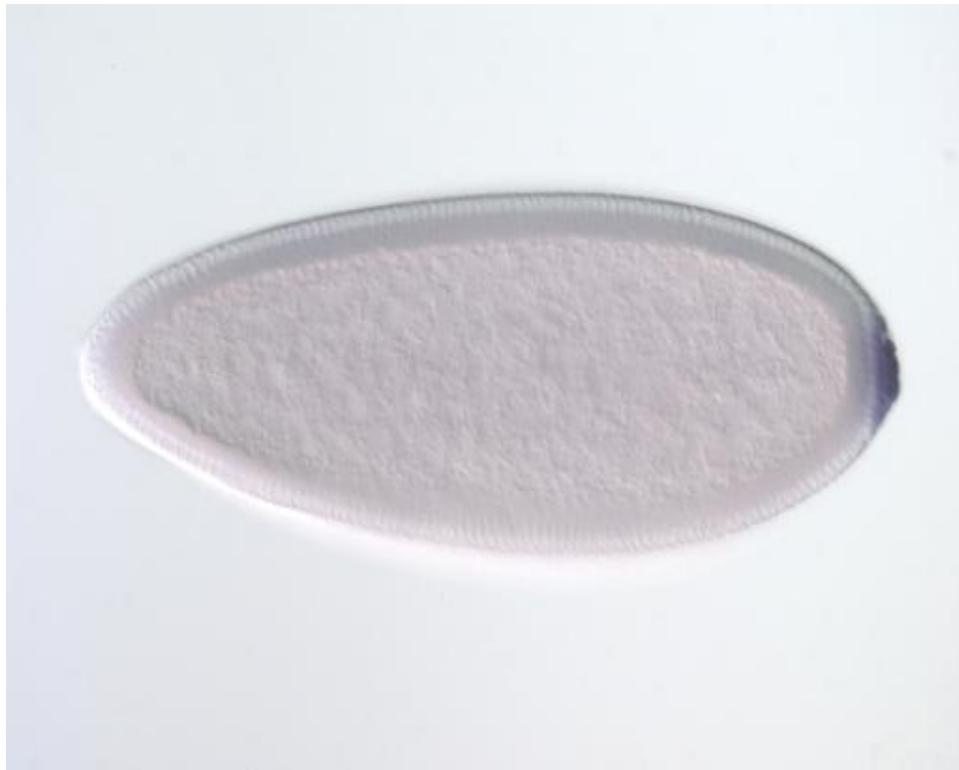

Location: Posterior Type: Promoter ZScore: 0.025710339 PValue: 0.979488377

Supplement: S3 File — Reports consist of in situ hybridization images, ATAC-seq traces, and calculated p-value and Z Score for each region used in the final analysis. (ZIP) [file pgen.1007367.s015.zip › S3_File/alpha-Adaptin_Report.pdf]

Ama

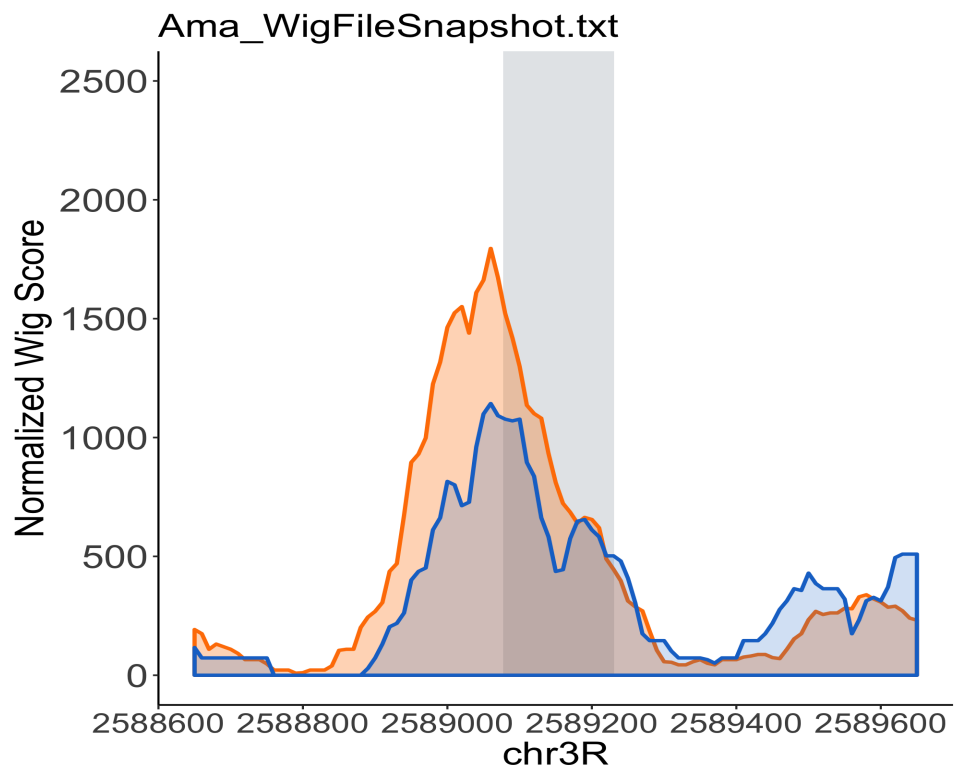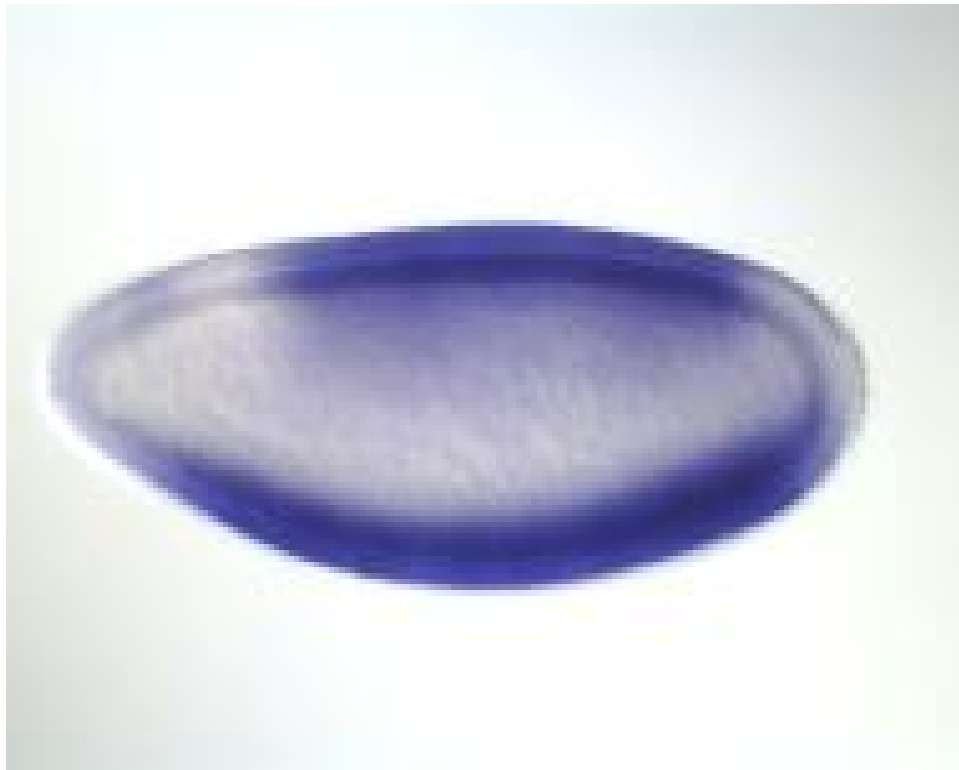

Location: Ventral Type: Promoter ZScore: 0.549952771 PValue: 0.582351768

Supplement: S3 File — Reports consist of in situ hybridization images, ATAC-seq traces, and calculated p-value and Z Score for each region used in the final analysis. (ZIP) [file pgen.1007367.s015.zip › S3_File/Ama_Report.pdf]

apt\_Ozdemir

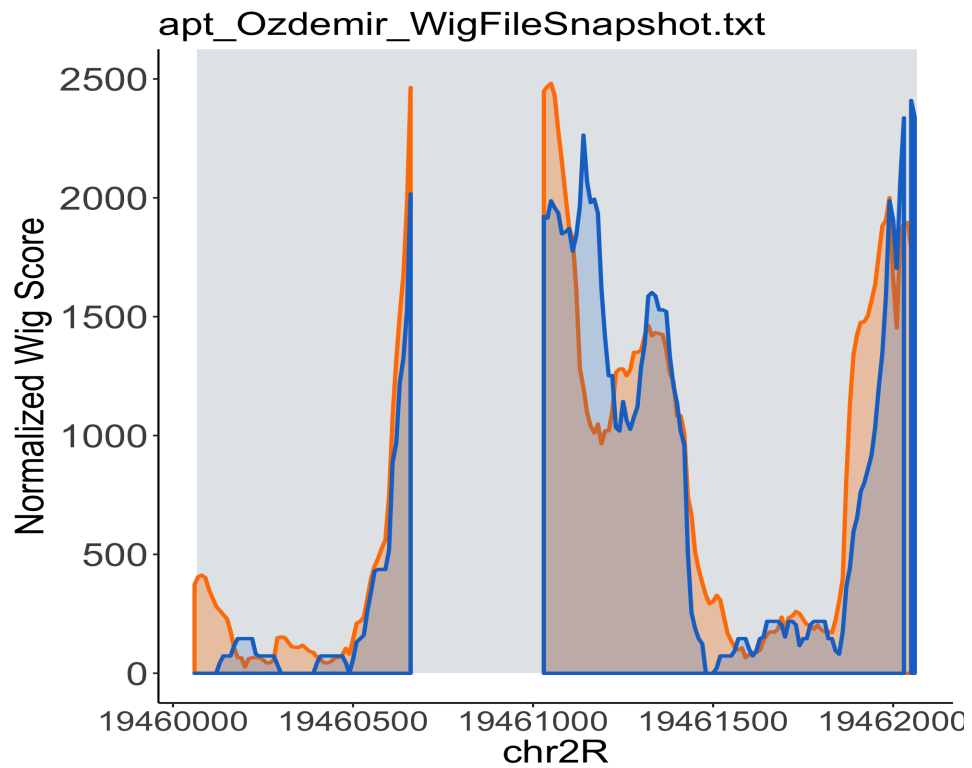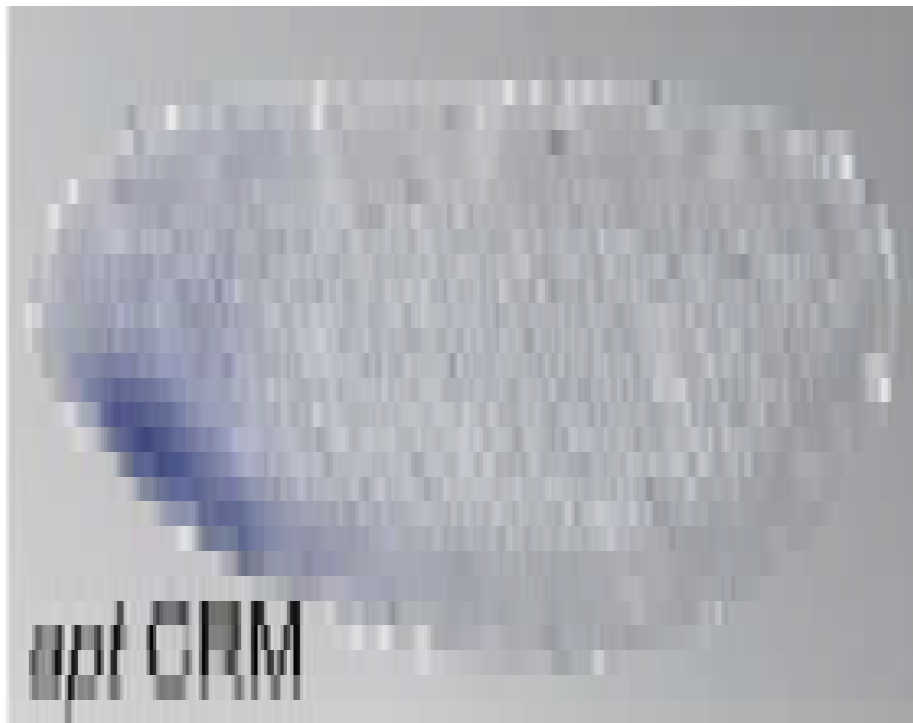

Location: Ventral Type: Enhancer ZScore: -0.211743584 PValue: 0.832307084

Supplement: S3 File — Reports consist of in situ hybridization images, ATAC-seq traces, and calculated p-value and Z Score for each region used in the final analysis. (ZIP) [file pgen.1007367.s015.zip › S3_File/apt_Ozdemir_Report.pdf]

# Asph\_Ozdemir

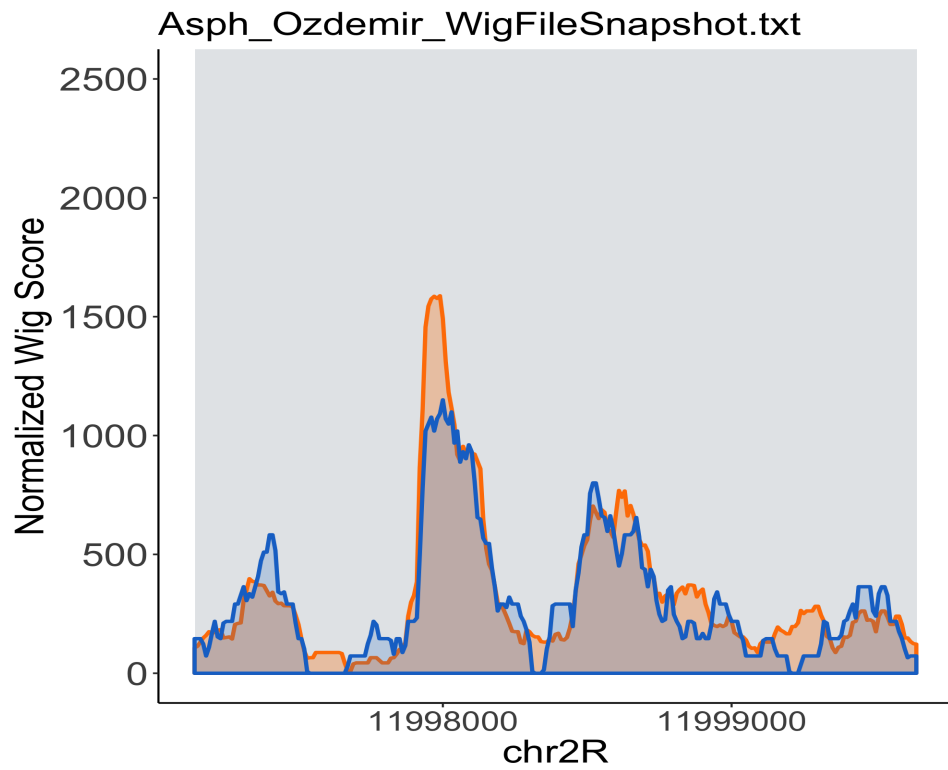

Location: Ventral Type: Enhancer ZScore: 0.159429077 PValue: 0.873330832

Supplement: S3 File — Reports consist of in situ hybridization images, ATAC-seq traces, and calculated p-value and Z Score for each region used in the final analysis. (ZIP) [file pgen.1007367.s015.zip › S3_File/Asph_Ozdemir_Report.pdf]

Asph

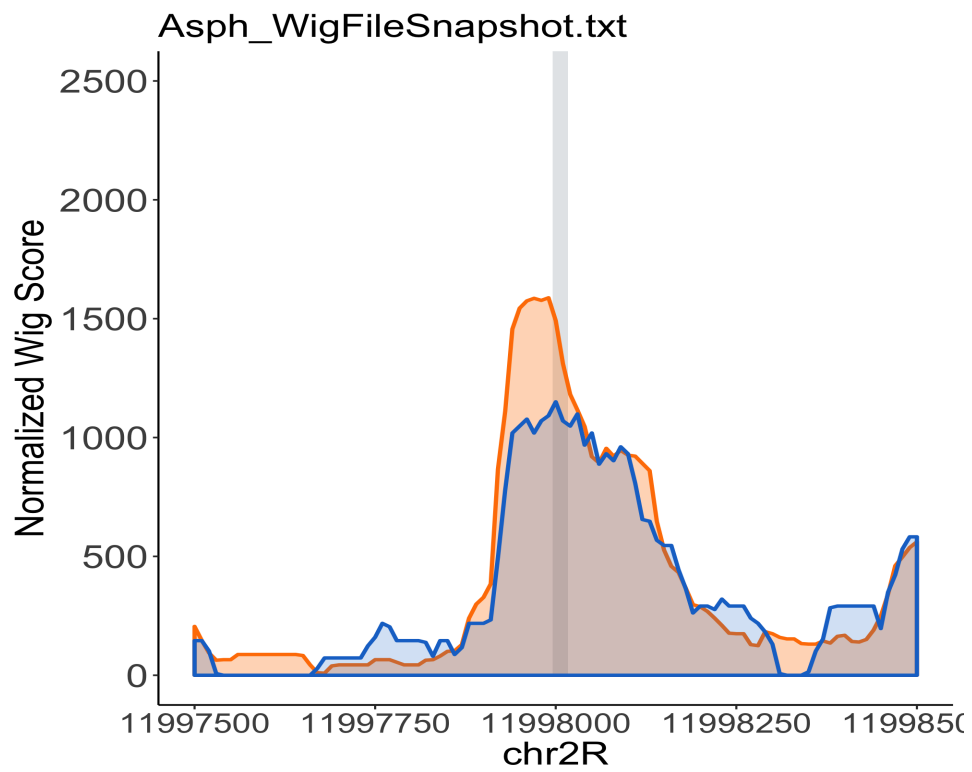

Location: Ventral Type: Promoter ZScore: 0.597691739 PValue: 0.550045639

Supplement: S3 File — Reports consist of in situ hybridization images, ATAC-seq traces, and calculated p-value and Z Score for each region used in the final analysis. (ZIP) [file pgen.1007367.s015.zip › S3_File/Asph_Report.pdf]

Atet

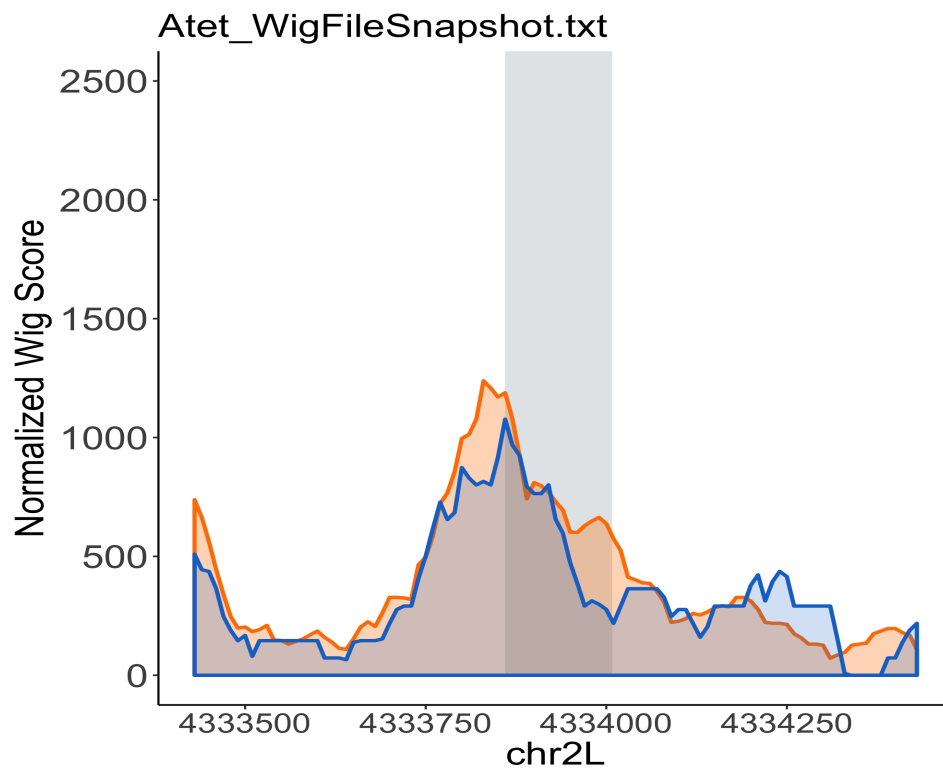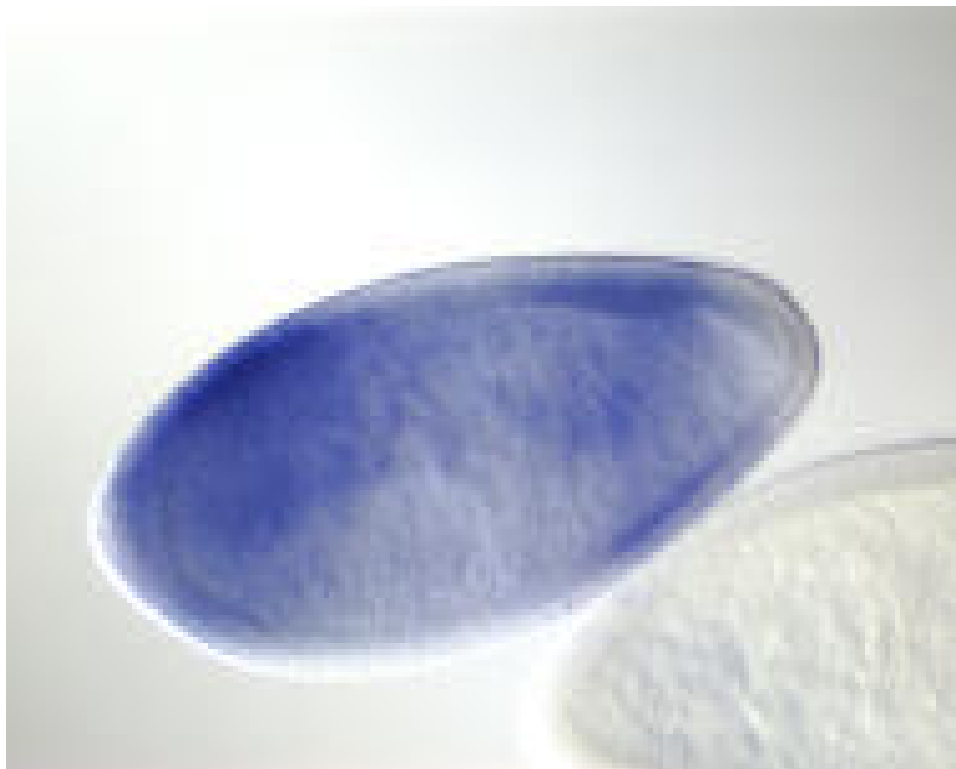

Location: Dorsal Type: Promoter ZScore: 0.423828522 PValue: 0.671690875

Supplement: S3 File — Reports consist of in situ hybridization images, ATAC-seq traces, and calculated p-value and Z Score for each region used in the final analysis. (ZIP) [file pgen.1007367.s015.zip › S3_File/Atet_Report.pdf]

Atg1

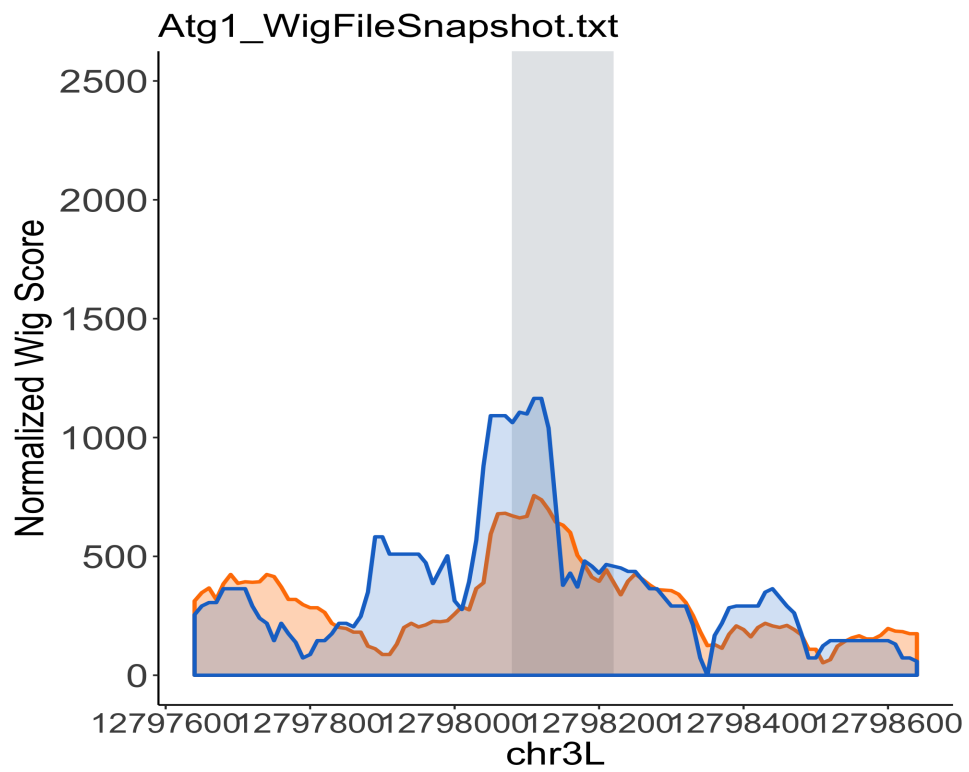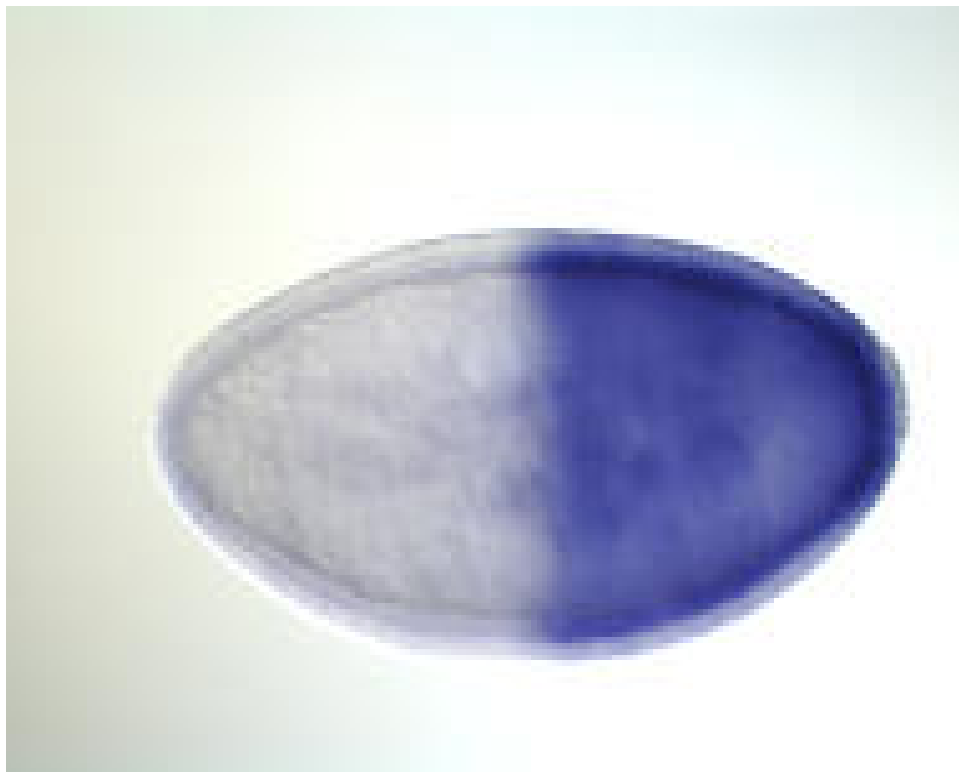

Location: Posterior Type: Promoter ZScore: 0.506759722 PValue: 0.612323421

Supplement: S3 File — Reports consist of in situ hybridization images, ATAC-seq traces, and calculated p-value and Z Score for each region used in the final analysis. (ZIP) [file pgen.1007367.s015.zip › S3_File/Atg1_Report.pdf]

bbg

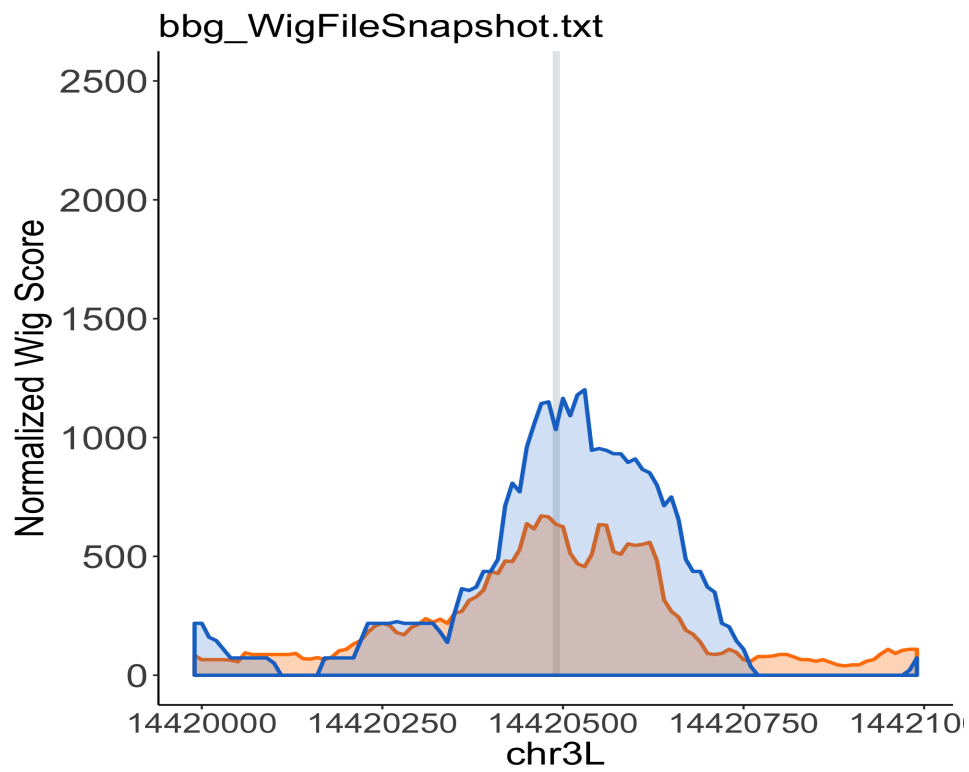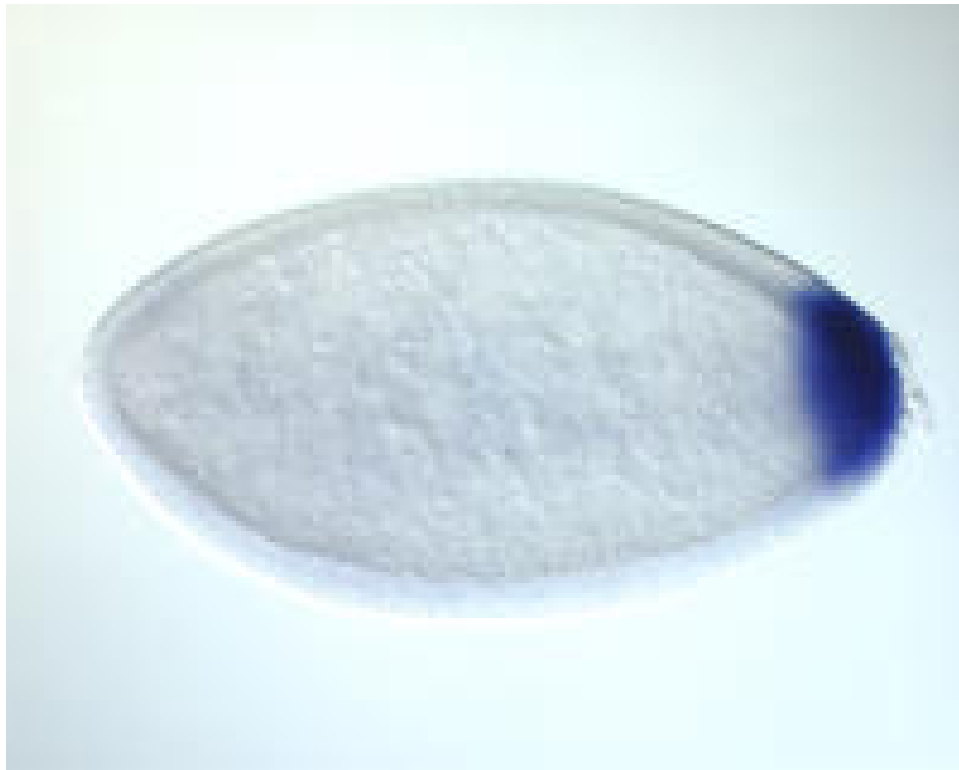

Location: Posterior Type: Promoter ZScore: 1.116874769 PValue: 0.264047874

Supplement: S3 File — Reports consist of in situ hybridization images, ATAC-seq traces, and calculated p-value and Z Score for each region used in the final analysis. (ZIP) [file pgen.1007367.s015.zip › S3_File/bbg_Report.pdf]

## BobA\_Ozdemir

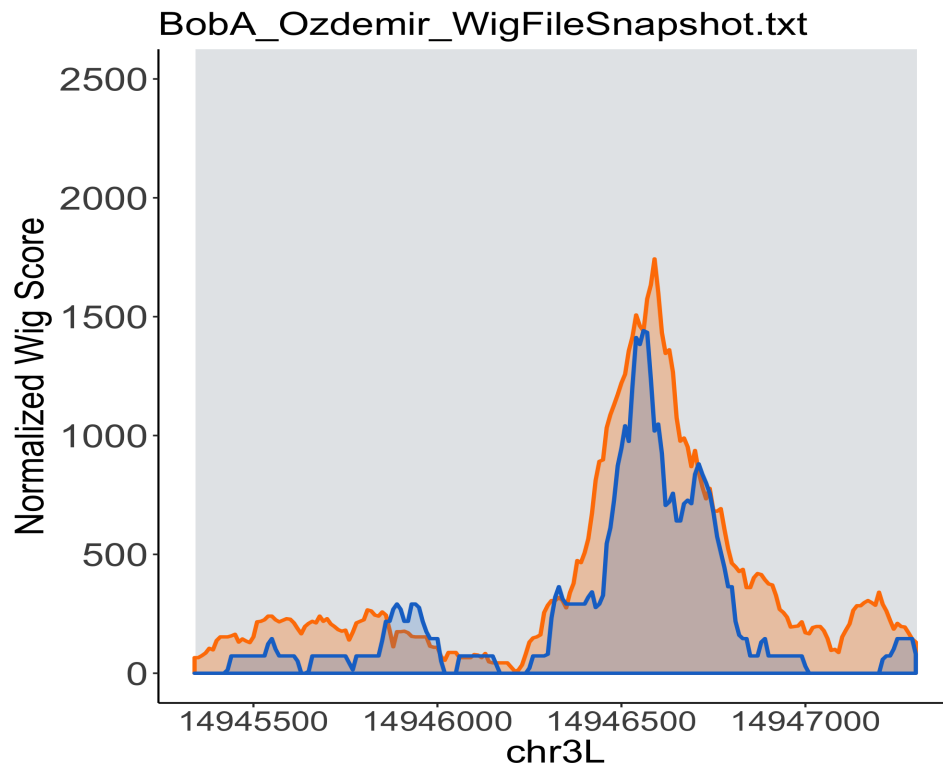

ASPM CRM

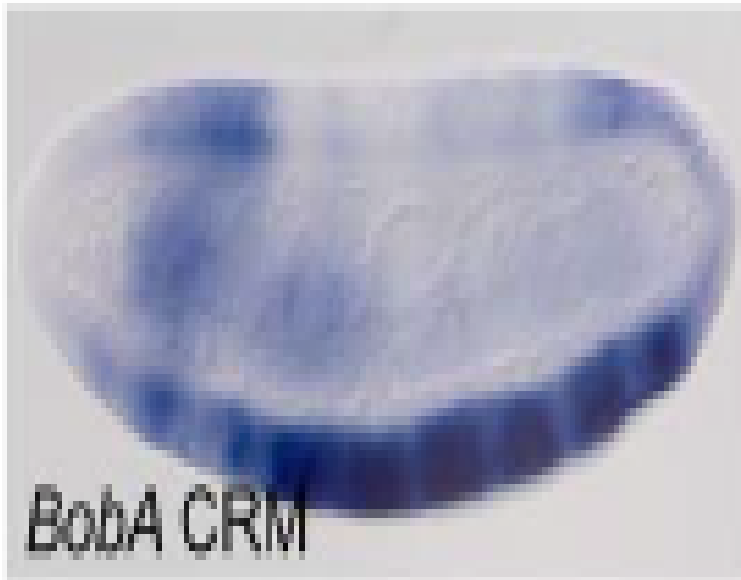

Location: Ventral Type: Enhancer ZScore: 1.073320176 PValue: 0.283127486

Supplement: S3 File — Reports consist of in situ hybridization images, ATAC-seq traces, and calculated p-value and Z Score for each region used in the final analysis. (ZIP) [file pgen.1007367.s015.zip › S3_File/BobA_Ozdemir_Report.pdf]

bowl

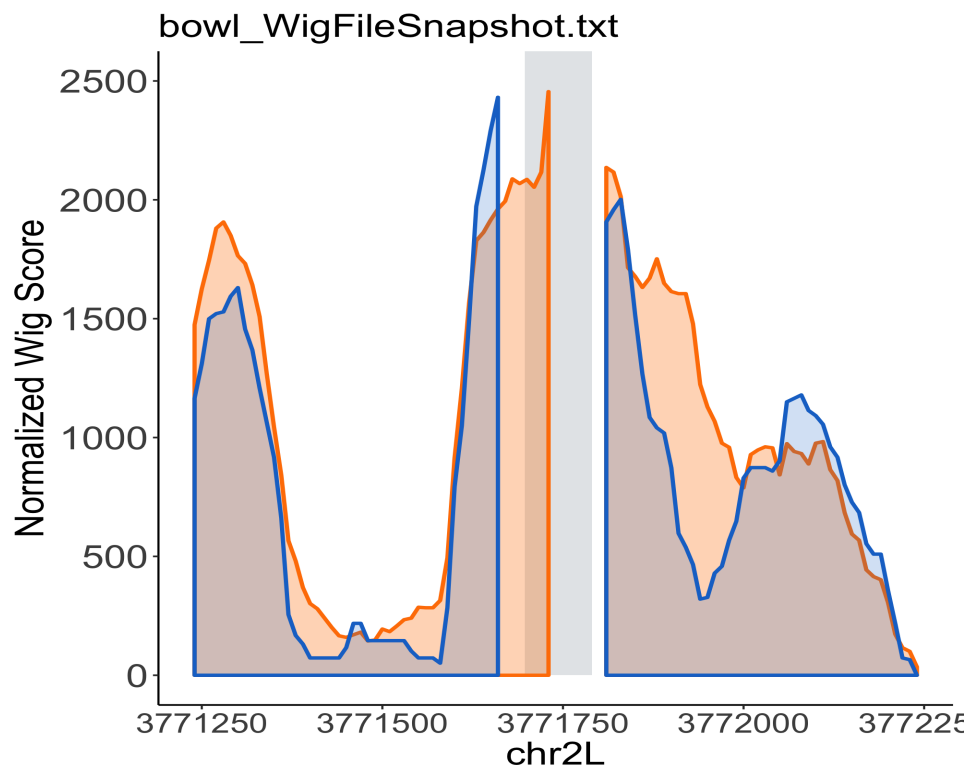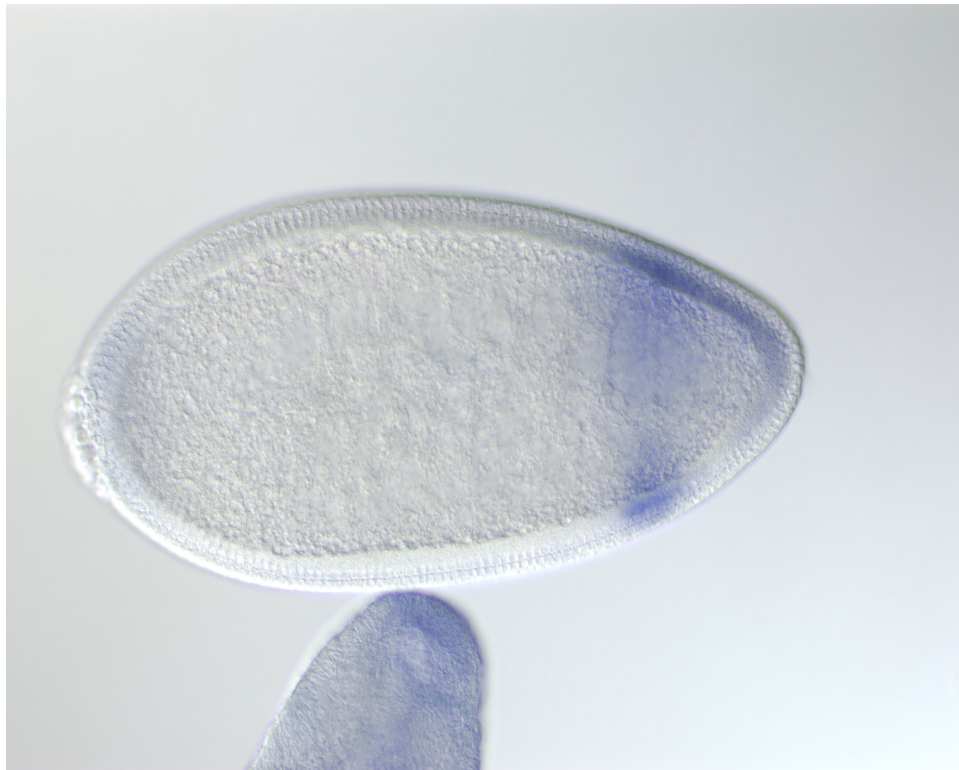

Location: Anterior Type: Promoter ZScore: -0.58166876 PValue: 0.560789818

Supplement: S3 File — Reports consist of in situ hybridization images, ATAC-seq traces, and calculated p-value and Z Score for each region used in the final analysis. (ZIP) [file pgen.1007367.s015.zip › S3_File/bowl_Report.pdf]

brk

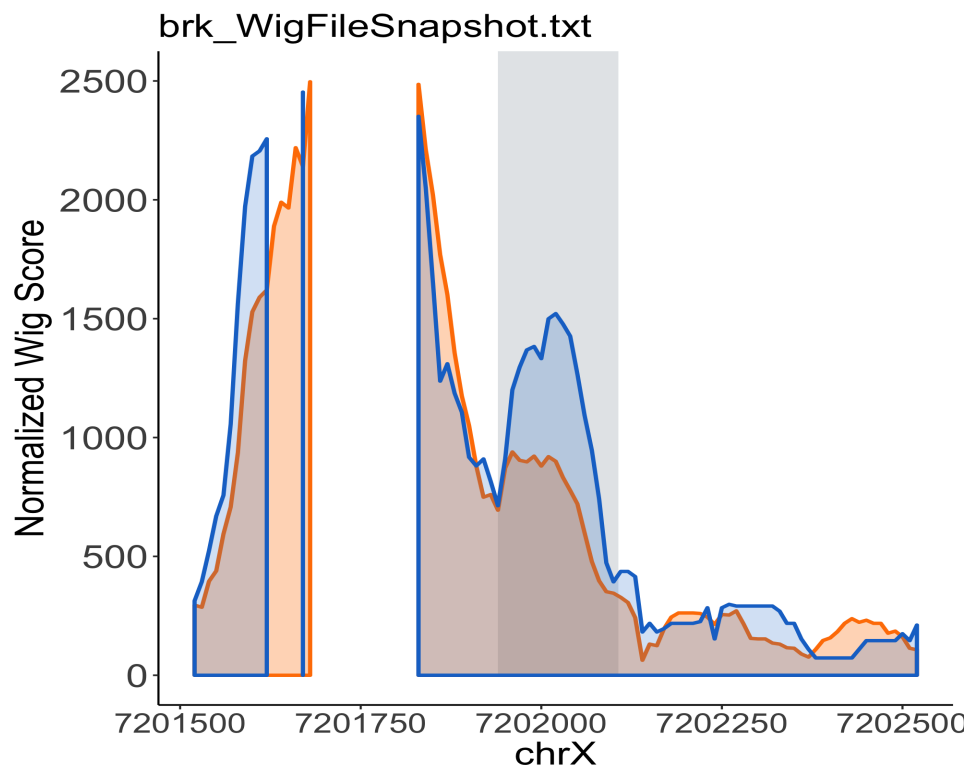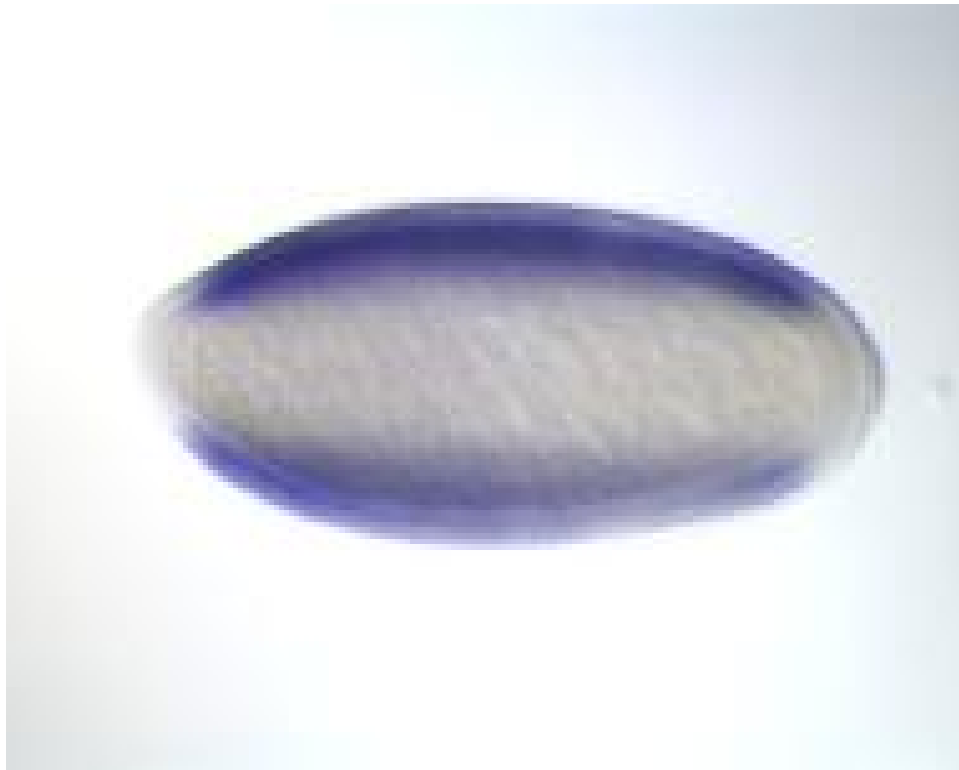

Location: Ventral Type: Promoter ZScore: -1.009866409 PValue: 0.312559298

Supplement: S3 File — Reports consist of in situ hybridization images, ATAC-seq traces, and calculated p-value and Z Score for each region used in the final analysis. (ZIP) [file pgen.1007367.s015.zip › S3_File/brk_Report.pdf]

btd

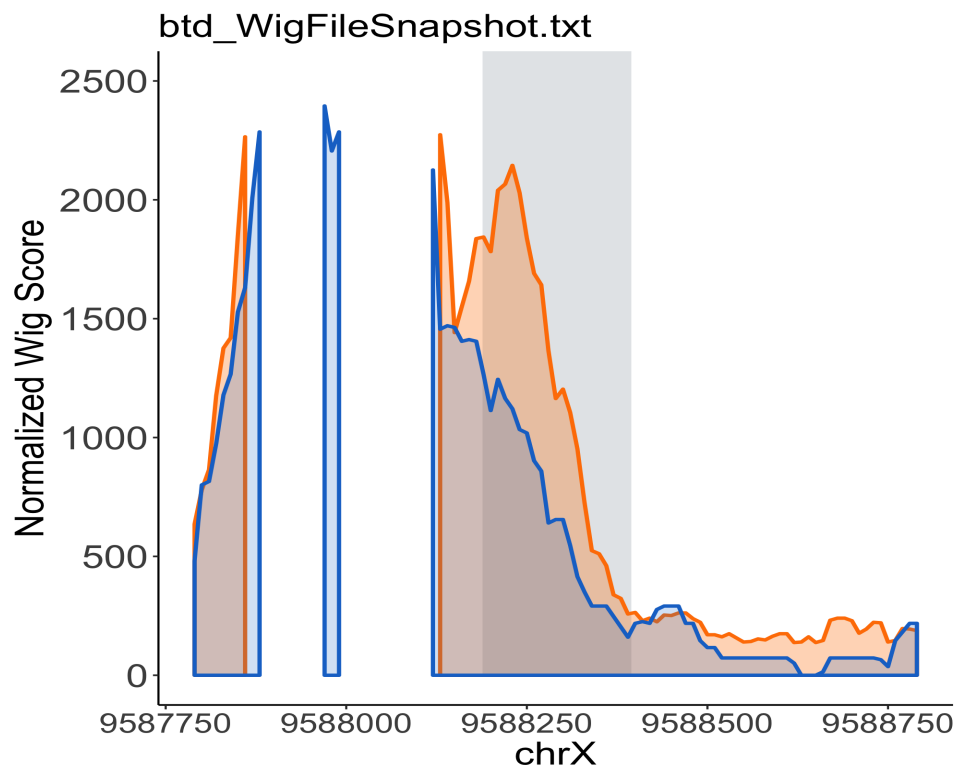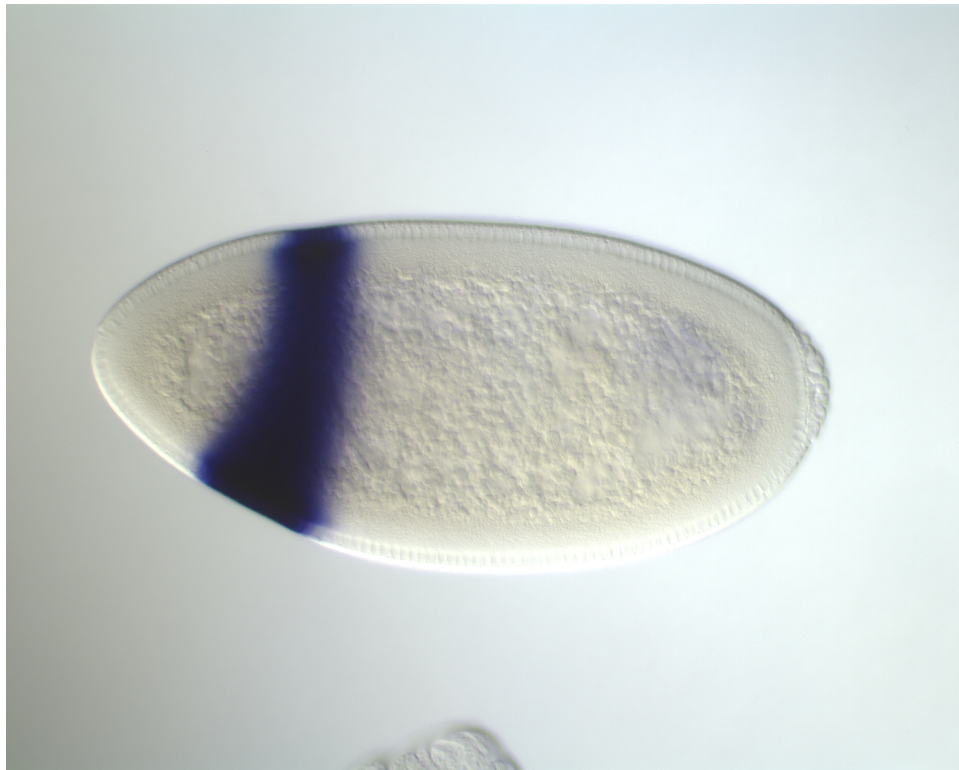

Location: Anterior Type: Promoter ZScore: 1.211763518 PValue: 0.225602917

Supplement: S3 File — Reports consist of in situ hybridization images, ATAC-seq traces, and calculated p-value and Z Score for each region used in the final analysis. (ZIP) [file pgen.1007367.s015.zip › S3_File/btd_Report.pdf]

bwa

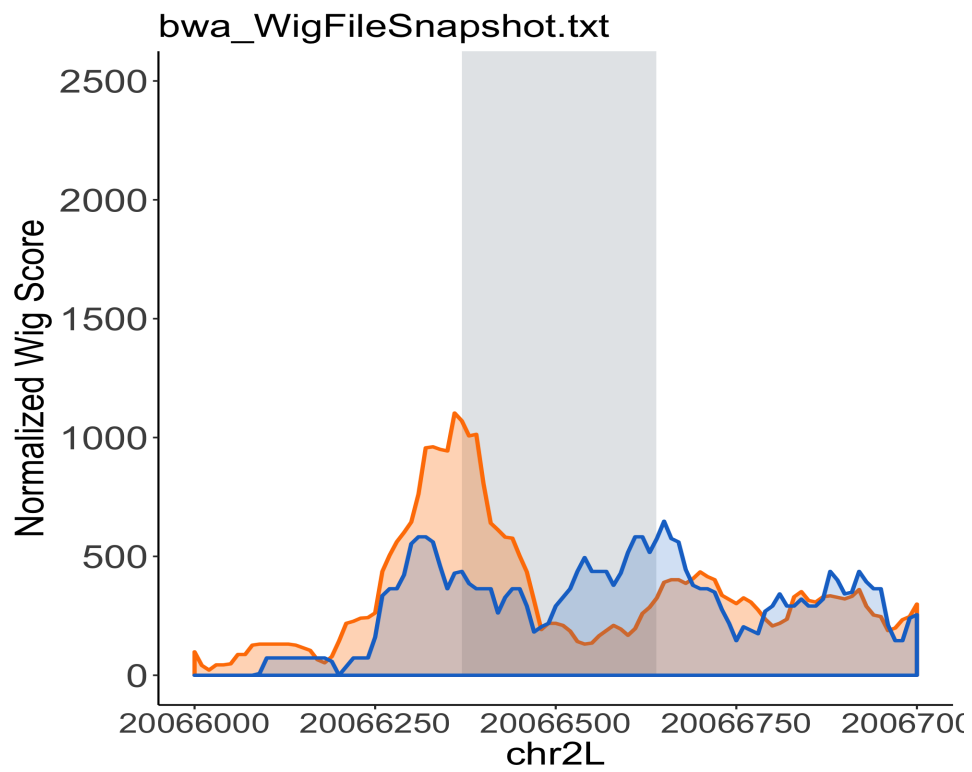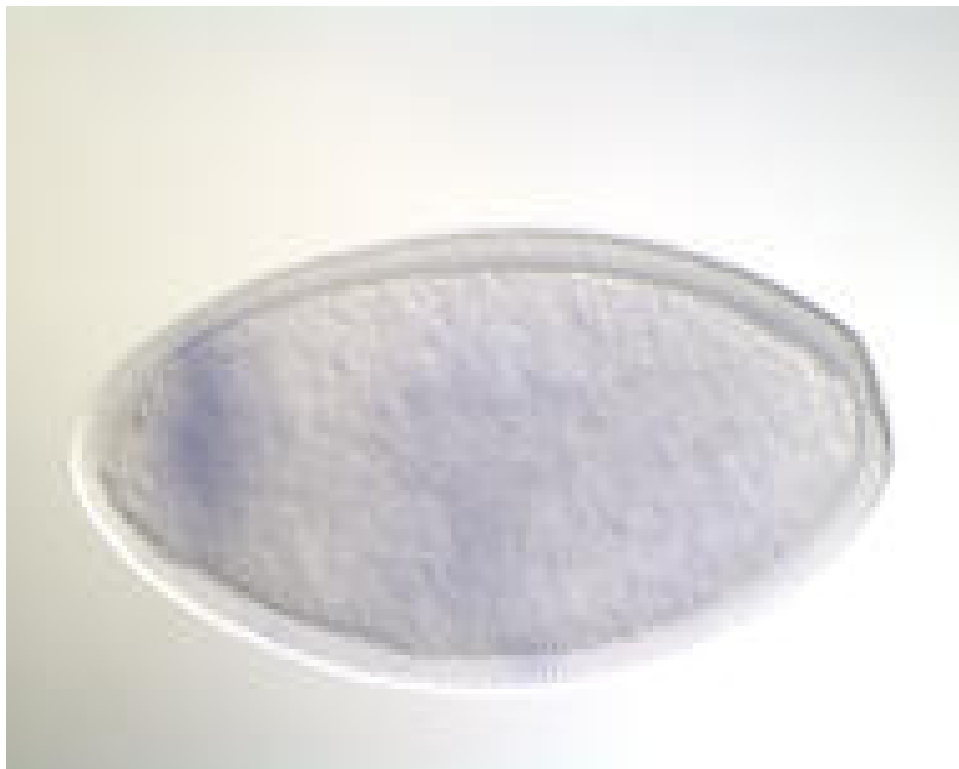

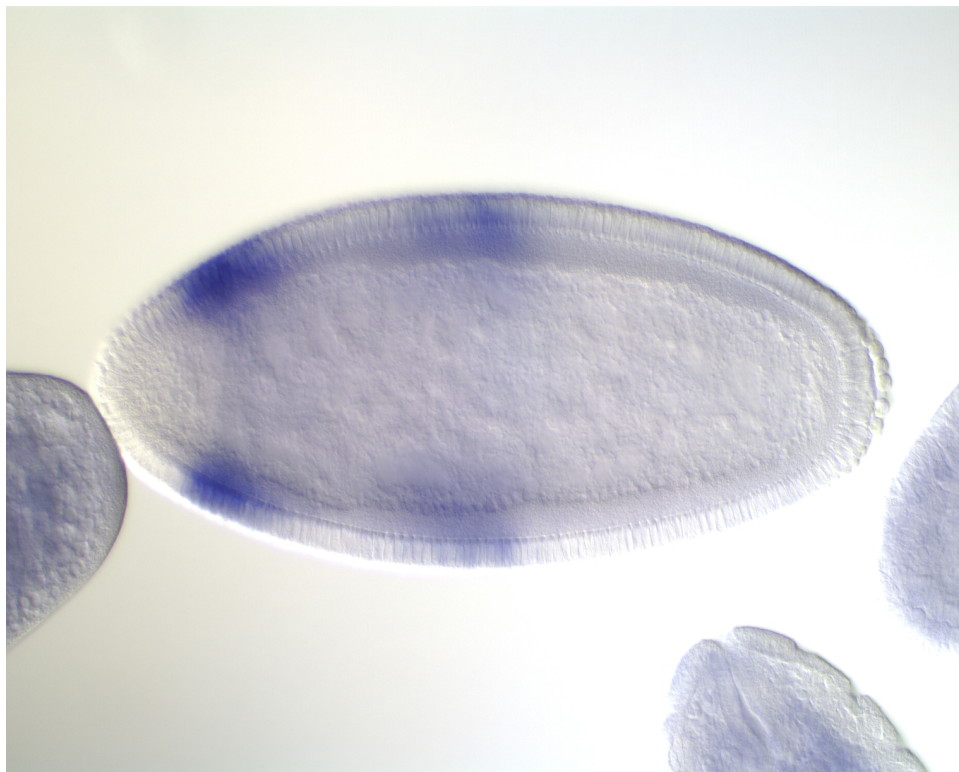

Location: Mostly Ant Type: Promoter ZScore: 0.020550921 PValue: 0.983603891

Supplement: S3 File — Reports consist of in situ hybridization images, ATAC-seq traces, and calculated p-value and Z Score for each region used in the final analysis. (ZIP) [file pgen.1007367.s015.zip › S3_File/bwa_Report.pdf]

## C15\_650\_Lin

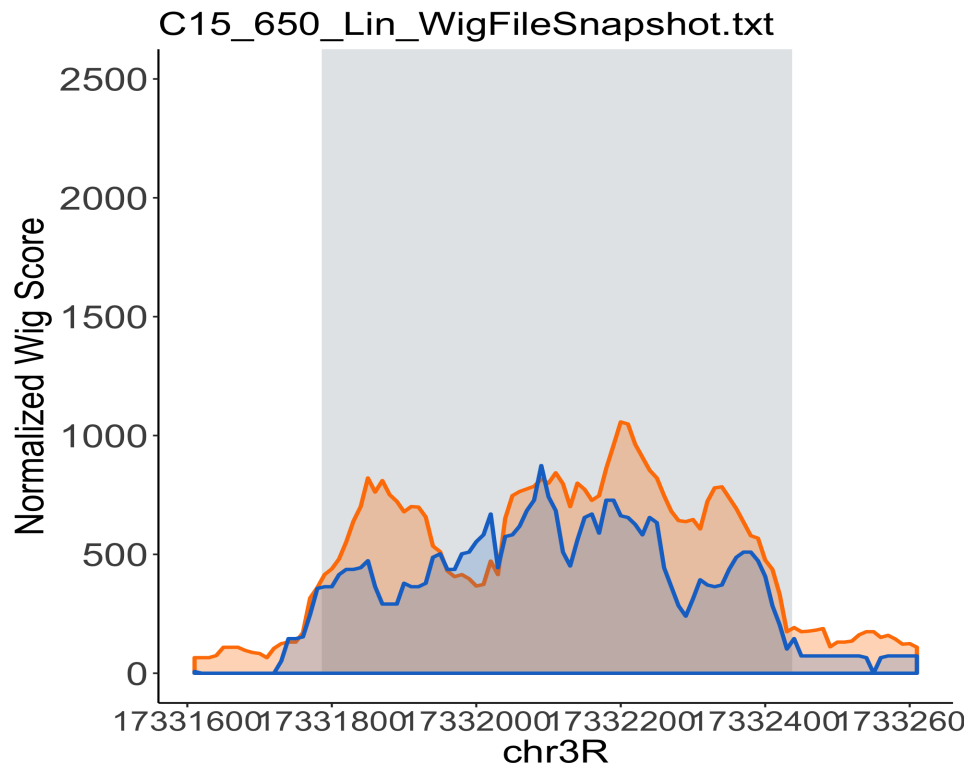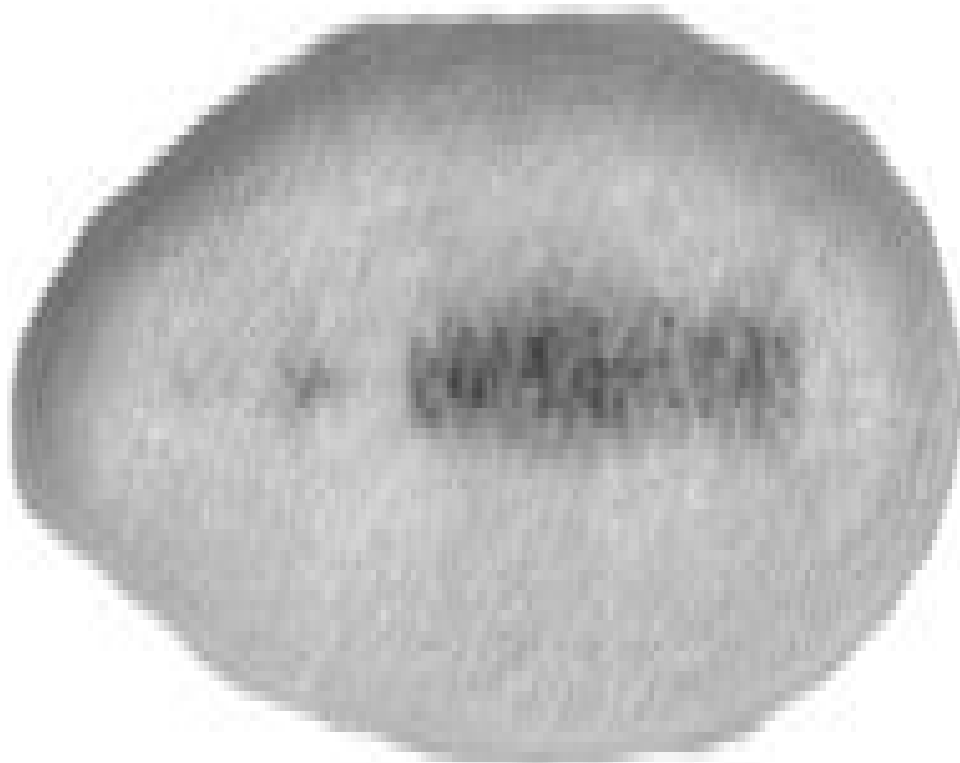

Location: Dorsal Type: Enhancer ZScore: 0.667229233 PValue: 0.504625721

Supplement: S3 File — Reports consist of in situ hybridization images, ATAC-seq traces, and calculated p-value and Z Score for each region used in the final analysis. (ZIP) [file pgen.1007367.s015.zip › S3_File/C15_650_Lin_Report.pdf]

C15

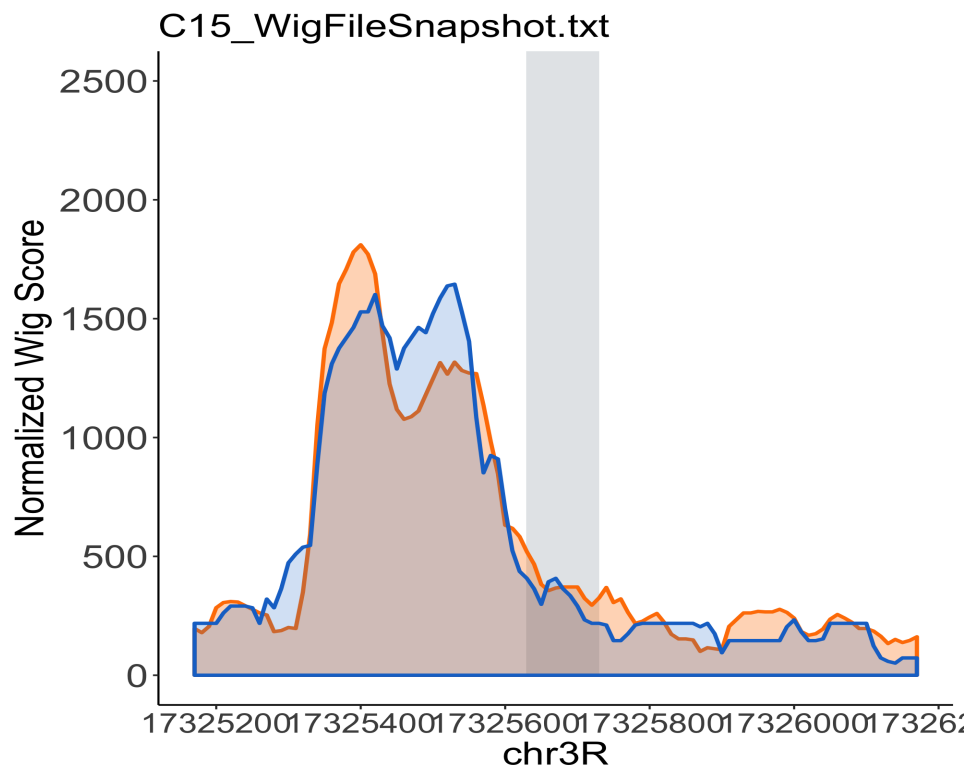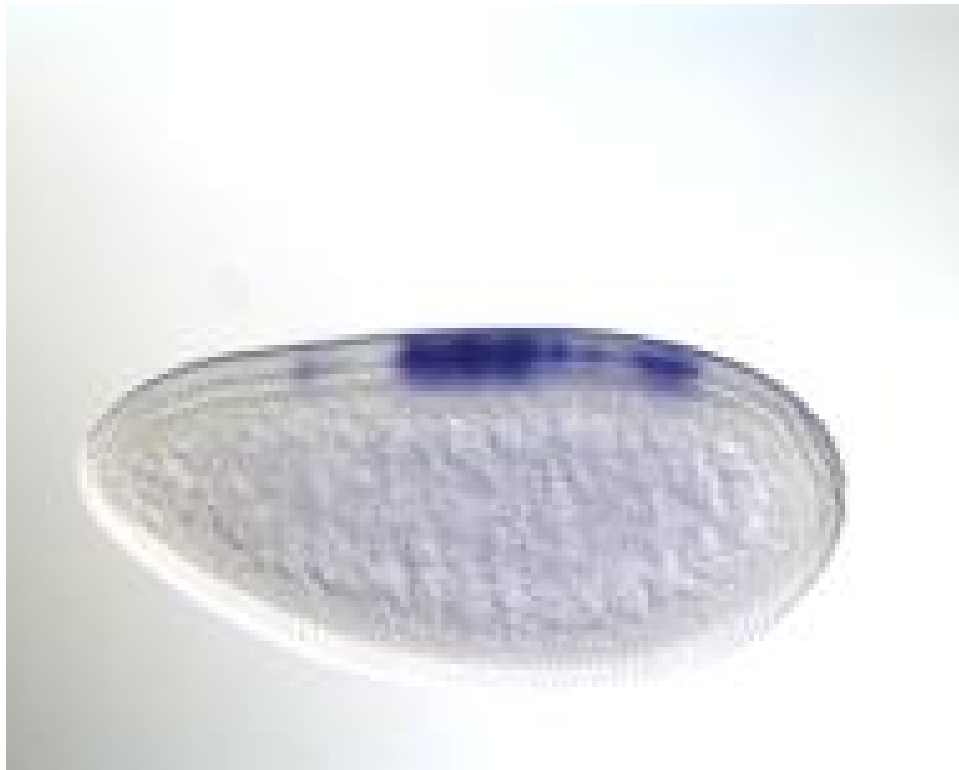

Location: Dorsal Type: Promoter ZScore: 0.359167825 PValue: 0.719469545

Supplement: S3 File — Reports consist of in situ hybridization images, ATAC-seq traces, and calculated p-value and Z Score for each region used in the final analysis. (ZIP) [file pgen.1007367.s015.zip › S3_File/C15_Report.pdf]

cad

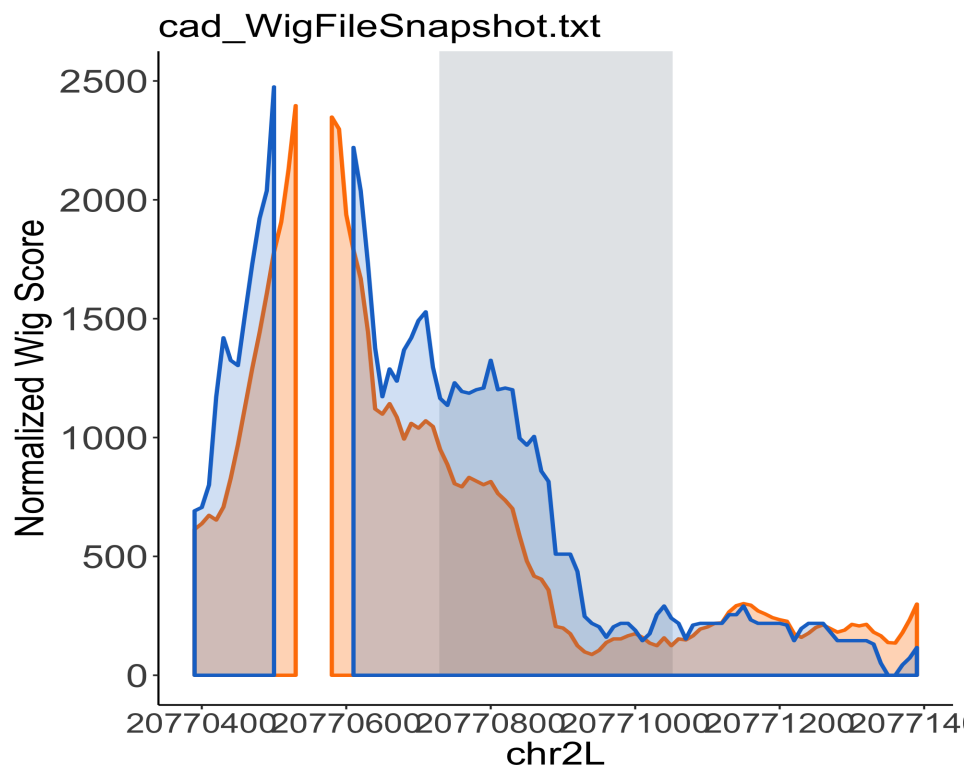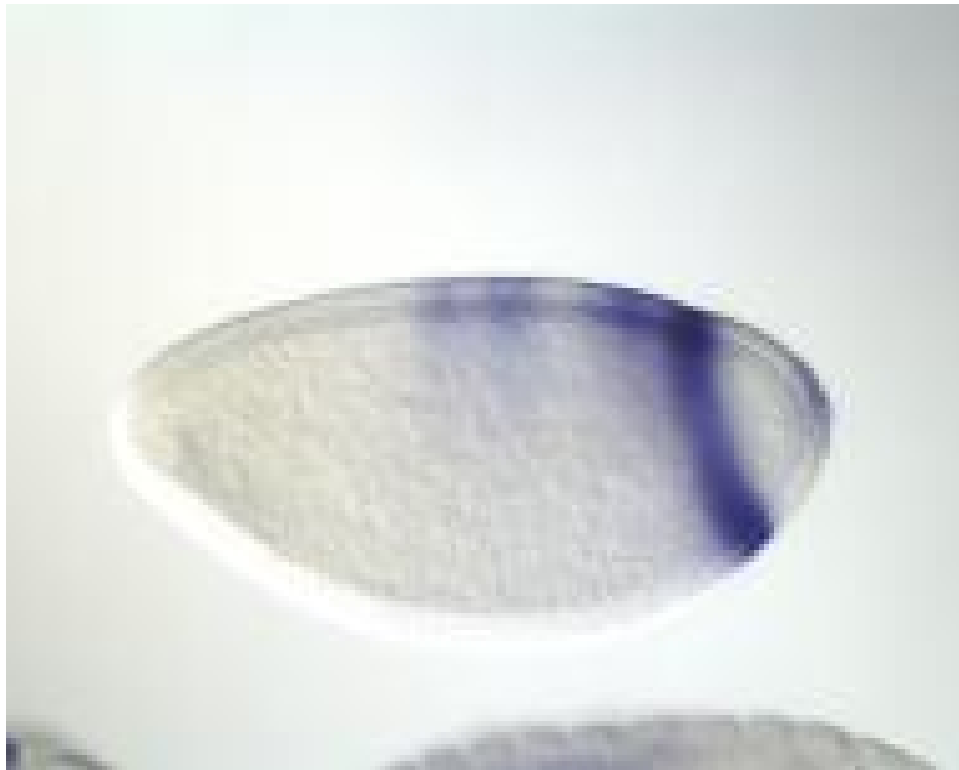

Location: Posterior Type: Promoter ZScore: 1.055720247 PValue: 0.291096044

Supplement: S3 File — Reports consist of in situ hybridization images, ATAC-seq traces, and calculated p-value and Z Score for each region used in the final analysis. (ZIP) [file pgen.1007367.s015.zip › S3_File/cad_Report.pdf]

# CG10479

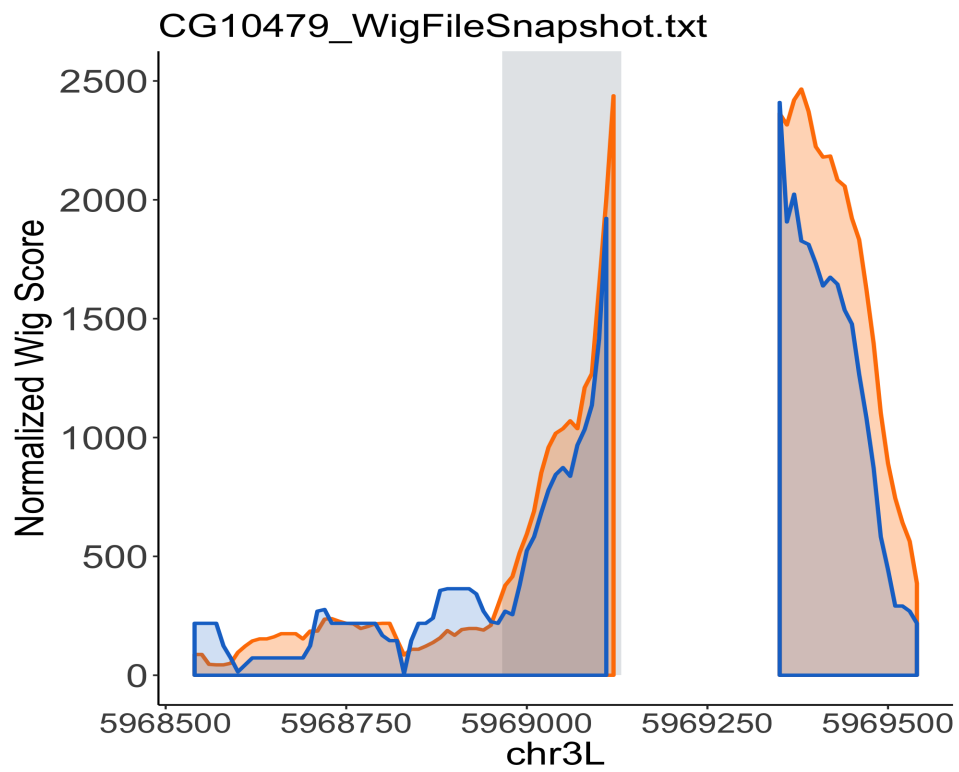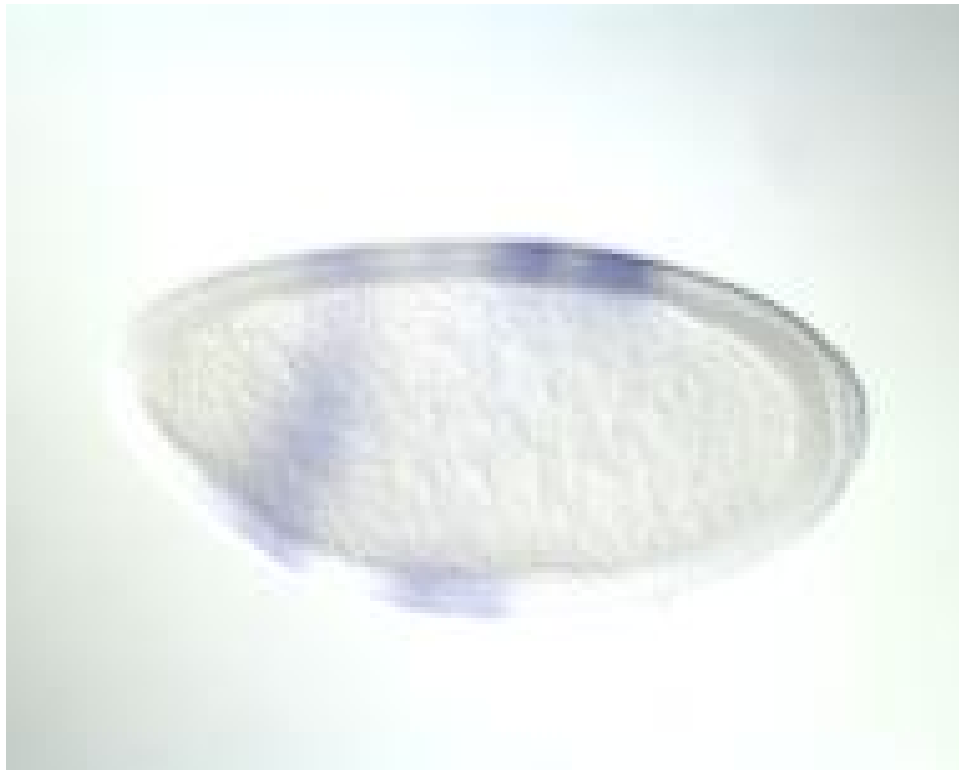

Location: Dorsal Type: Promoter ZScore: 0.12401195 PValue: 0.901305814

Supplement: S3 File — Reports consist of in situ hybridization images, ATAC-seq traces, and calculated p-value and Z Score for each region used in the final analysis. (ZIP) [file pgen.1007367.s015.zip › S3_File/CG10479_Report.pdf]

# CG11208

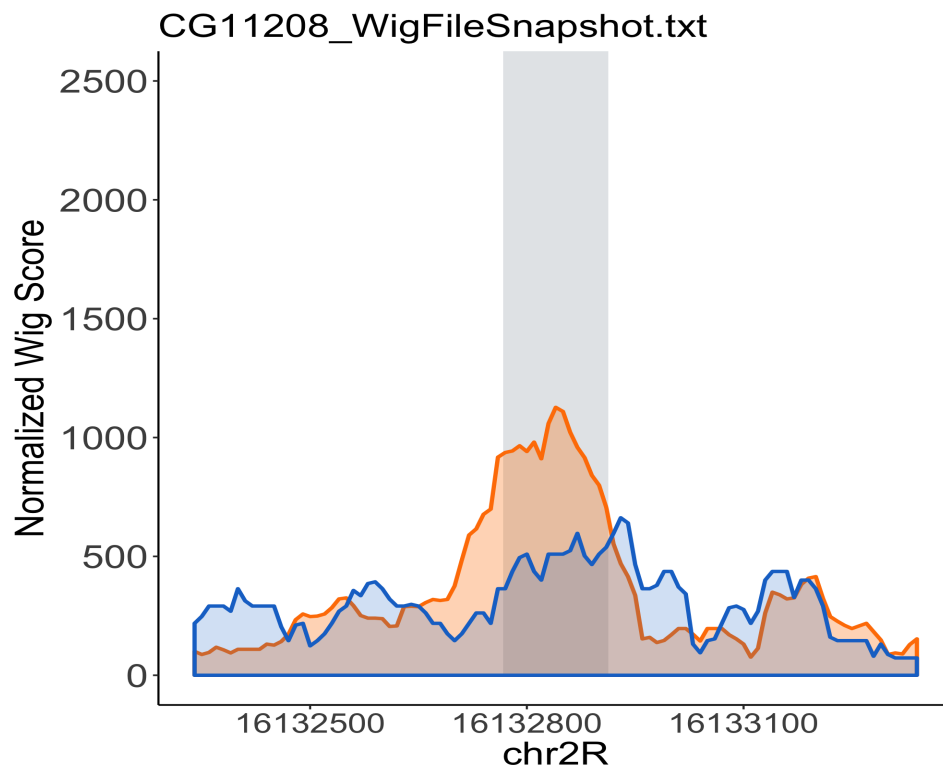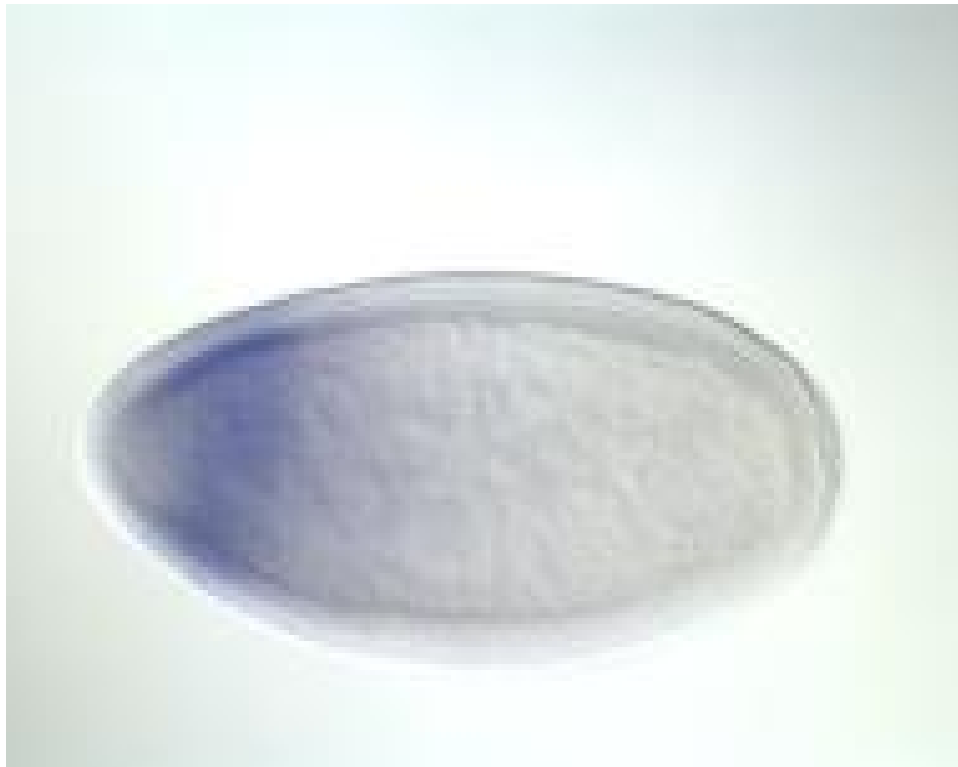

Location: Anterior Type: Promoter ZScore: 1.457333925 PValue: 0.145024227

Supplement: S3 File — Reports consist of in situ hybridization images, ATAC-seq traces, and calculated p-value and Z Score for each region used in the final analysis. (ZIP) [file pgen.1007367.s015.zip › S3_File/CG11208_Report.pdf]

# CG12177

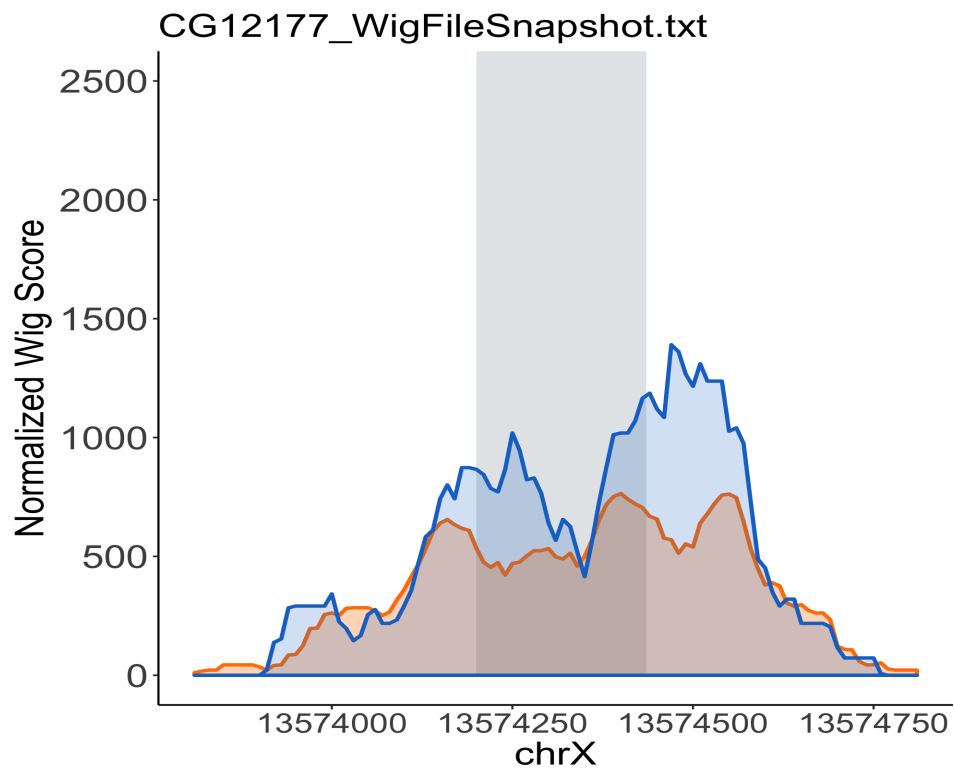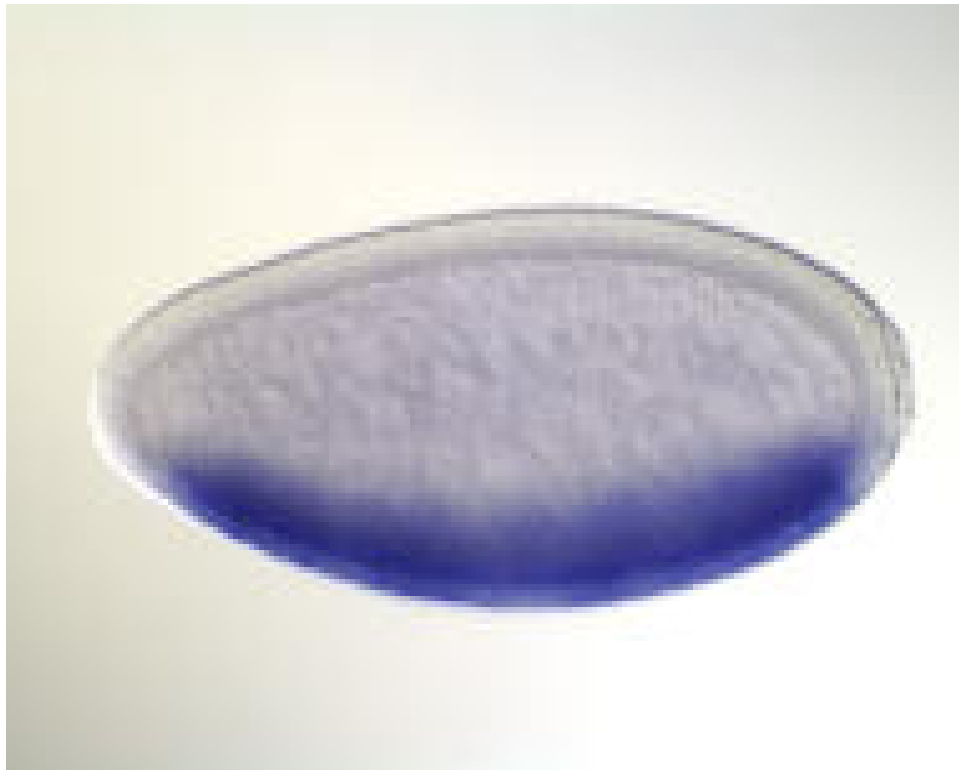

Location: Ventral Type: Promoter ZScore: -0.871434384 PValue: 0.383517019

Supplement: S3 File — Reports consist of in situ hybridization images, ATAC-seq traces, and calculated p-value and Z Score for each region used in the final analysis. (ZIP) [file pgen.1007367.s015.zip › S3_File/CG12177_Report.pdf]

# CG12420

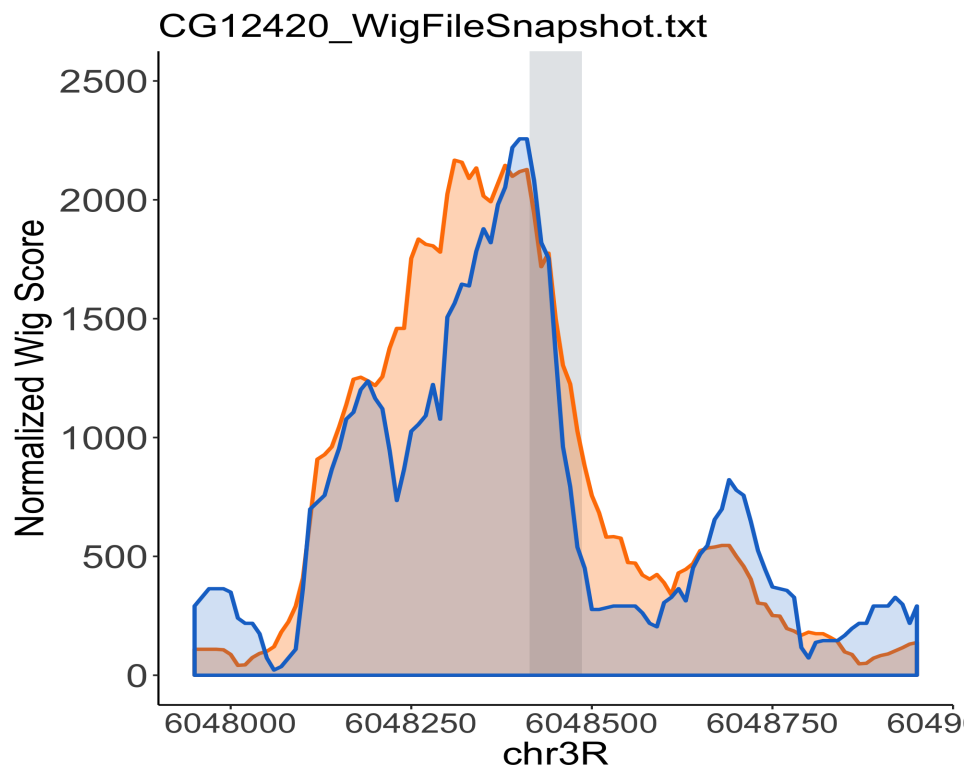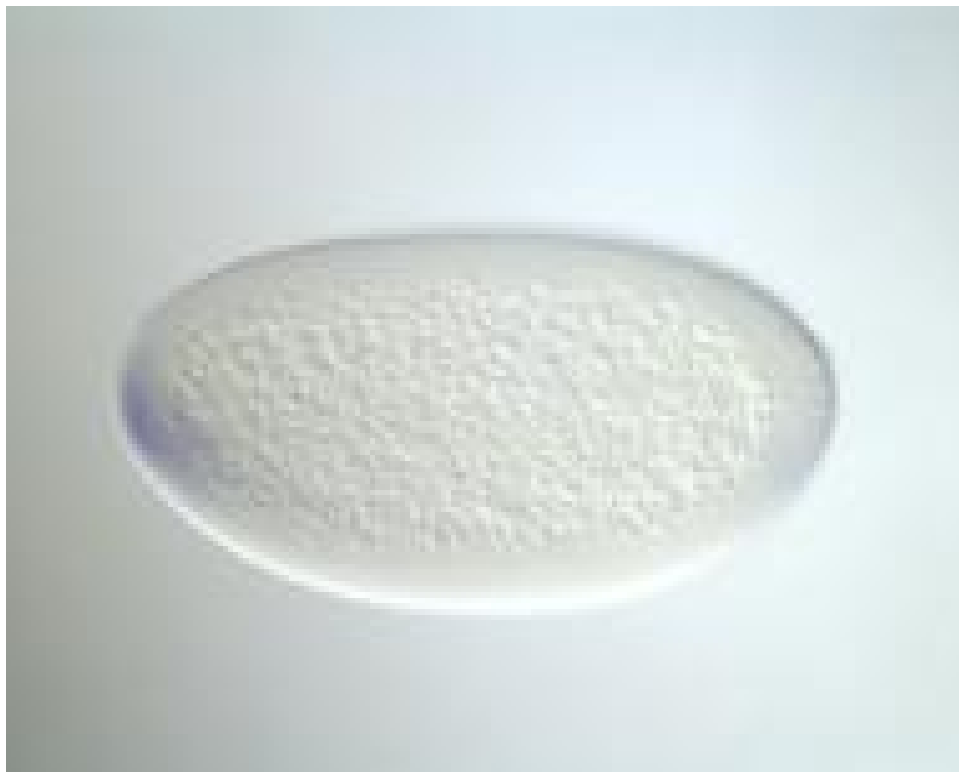

Location: Anterior Type: Promoter ZScore: 0.157803577 PValue: 0.87461158

Supplement: S3 File — Reports consist of in situ hybridization images, ATAC-seq traces, and calculated p-value and Z Score for each region used in the final analysis. (ZIP) [file pgen.1007367.s015.zip › S3_File/CG12420_Report.pdf]

# CG13653

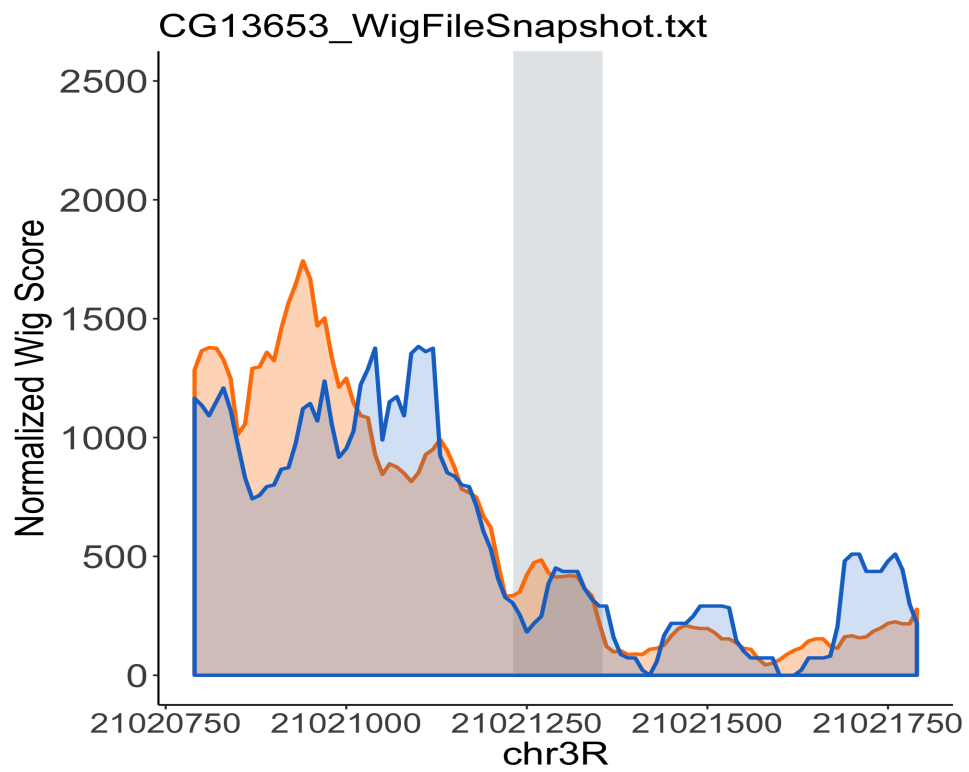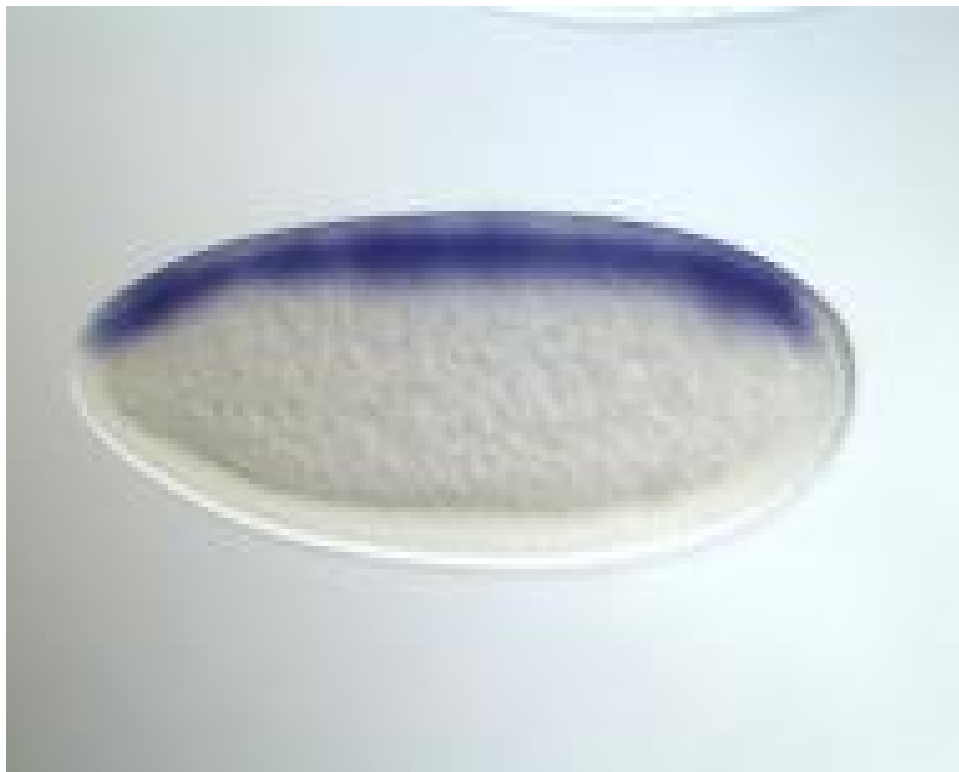

Location: Dorsal Type: Promoter ZScore: 0.319499024 PValue: 0.749348131

Supplement: S3 File — Reports consist of in situ hybridization images, ATAC-seq traces, and calculated p-value and Z Score for each region used in the final analysis. (ZIP) [file pgen.1007367.s015.zip › S3_File/CG13653_Report.pdf]

# CG13894

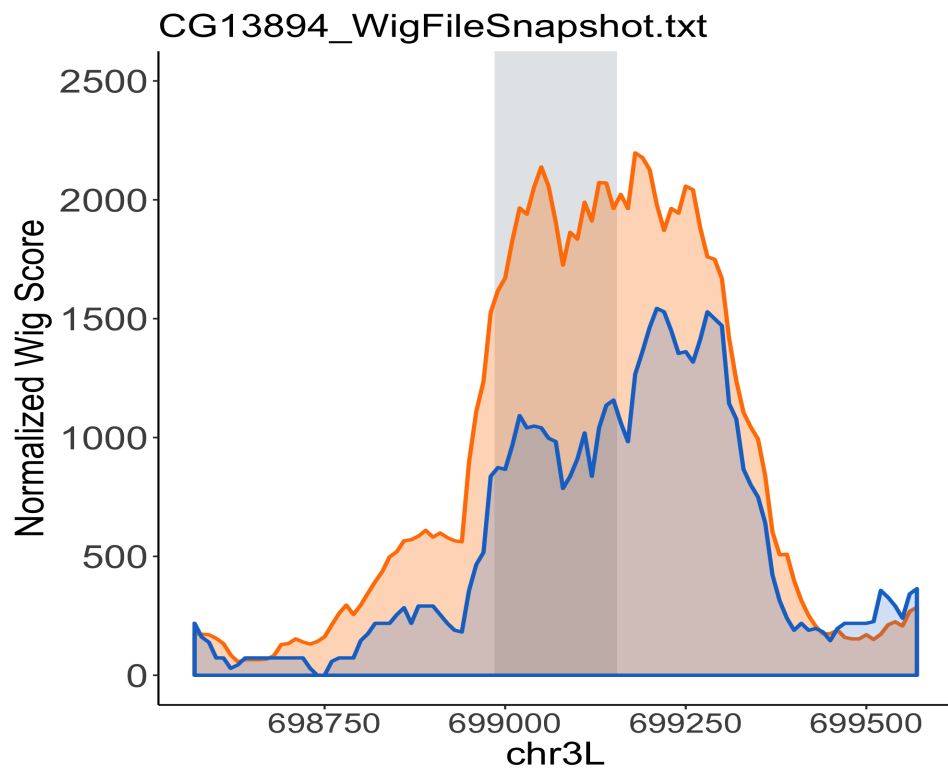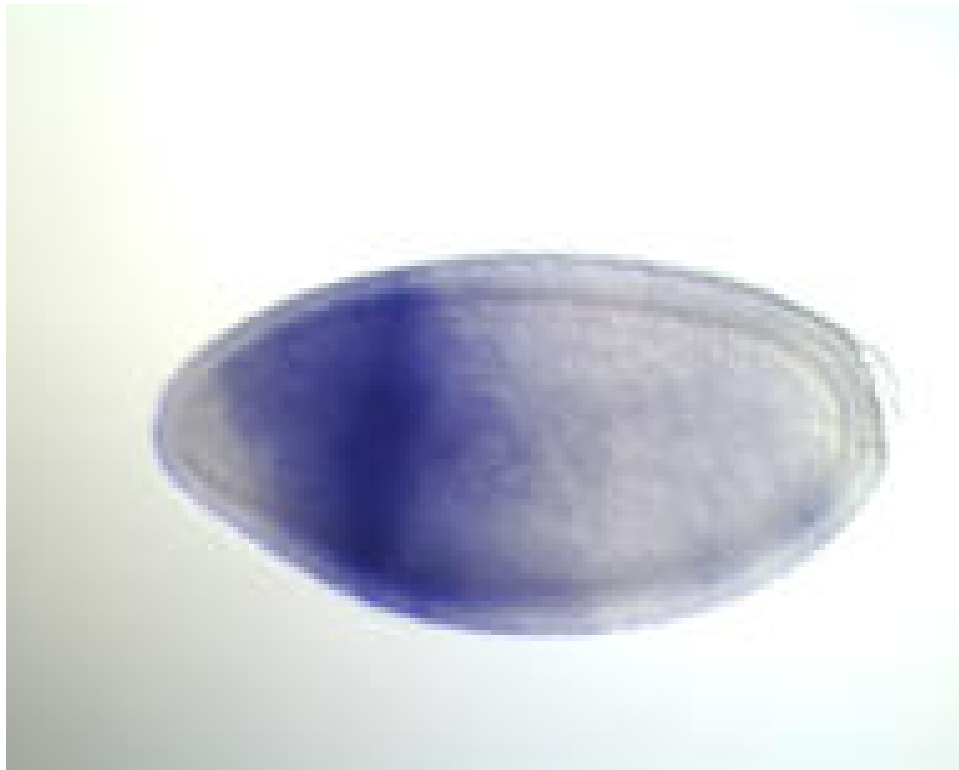

Location: Mostly Ant Type: Promoter ZScore: 1.438032965 PValue: 0.150424701

Supplement: S3 File — Reports consist of in situ hybridization images, ATAC-seq traces, and calculated p-value and Z Score for each region used in the final analysis. (ZIP) [file pgen.1007367.s015.zip › S3_File/CG13894_Report.pdf]

# CG15628

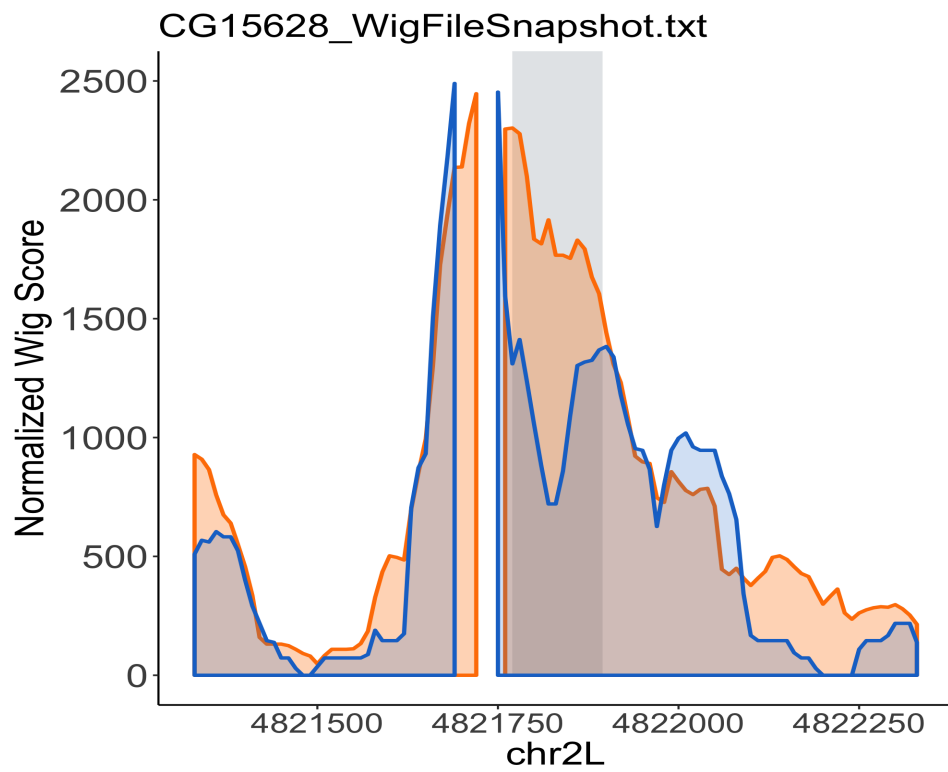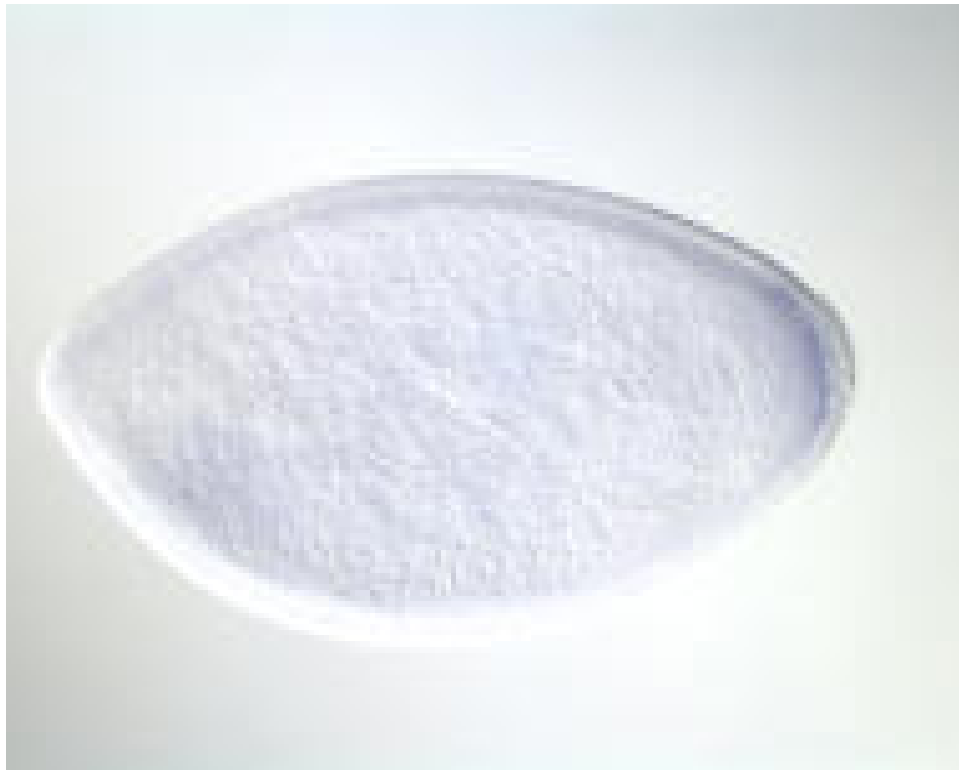

Location: Posterior Type: Promoter ZScore: -1.201919335 PValue: 0.229394783

Supplement: S3 File — Reports consist of in situ hybridization images, ATAC-seq traces, and calculated p-value and Z Score for each region used in the final analysis. (ZIP) [file pgen.1007367.s015.zip › S3_File/CG15628_Report.pdf]

## CG2930

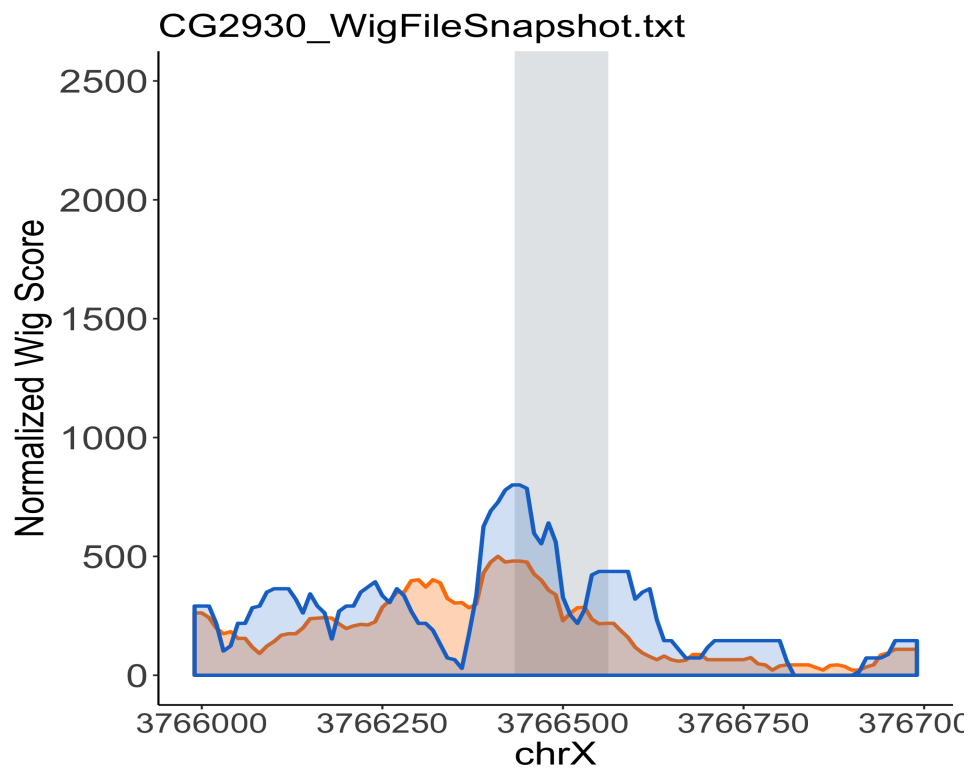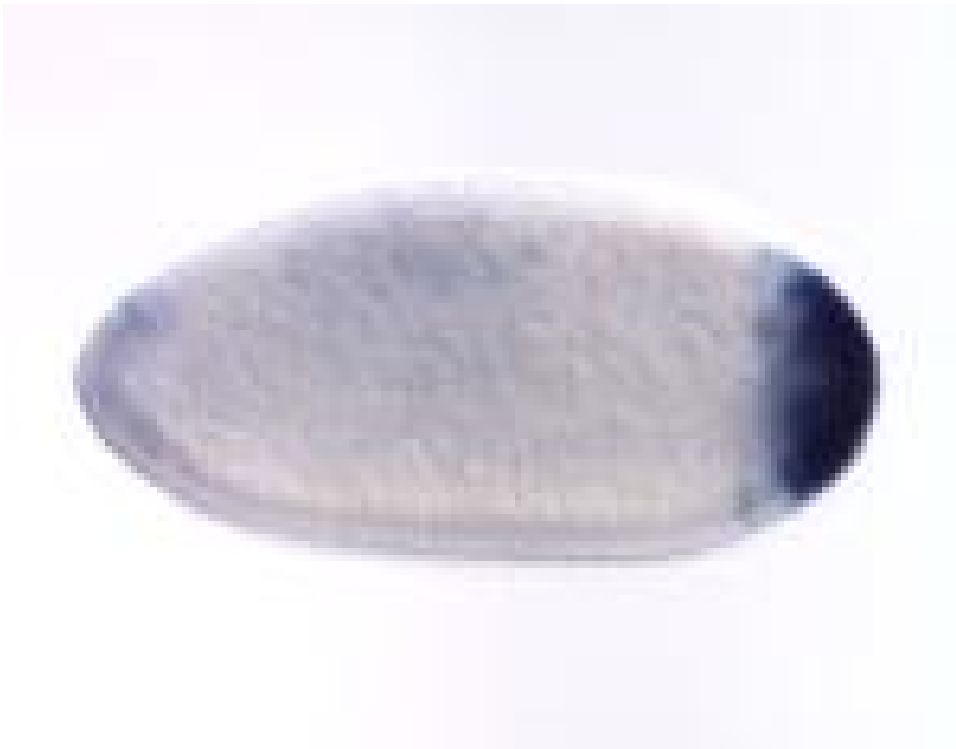

Location: Mostly Post Type: Promoter ZScore: 0.897464979 PValue: 0.369470853

Supplement: S3 File — Reports consist of in situ hybridization images, ATAC-seq traces, and calculated p-value and Z Score for each region used in the final analysis. (ZIP) [file pgen.1007367.s015.zip › S3_File/CG2930_Report.pdf]

CG32982

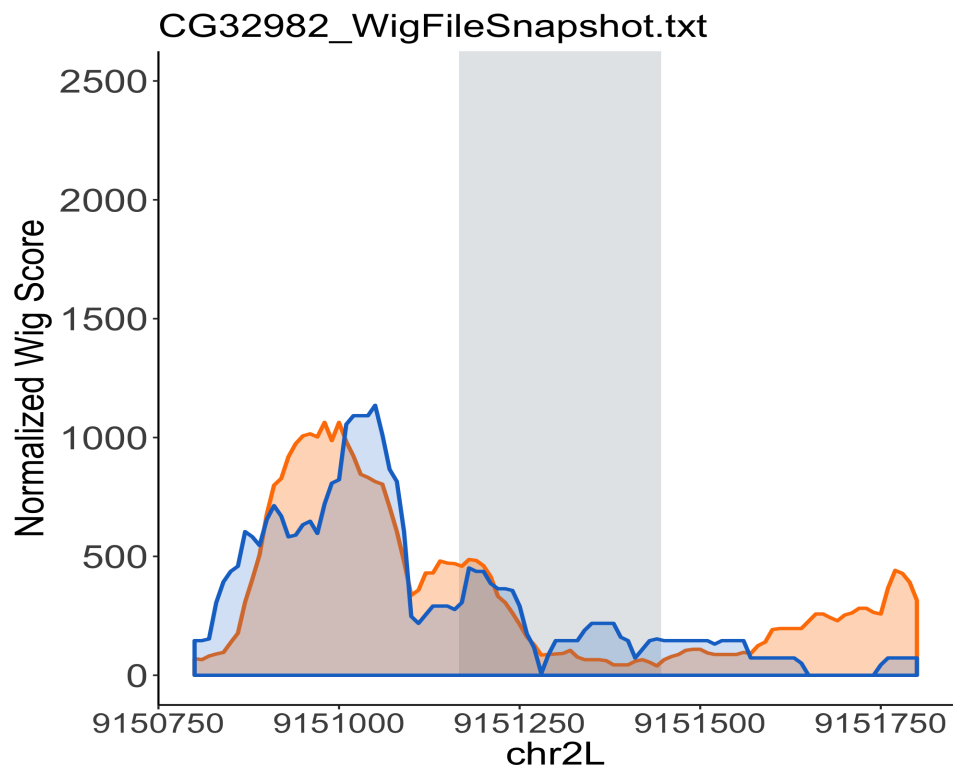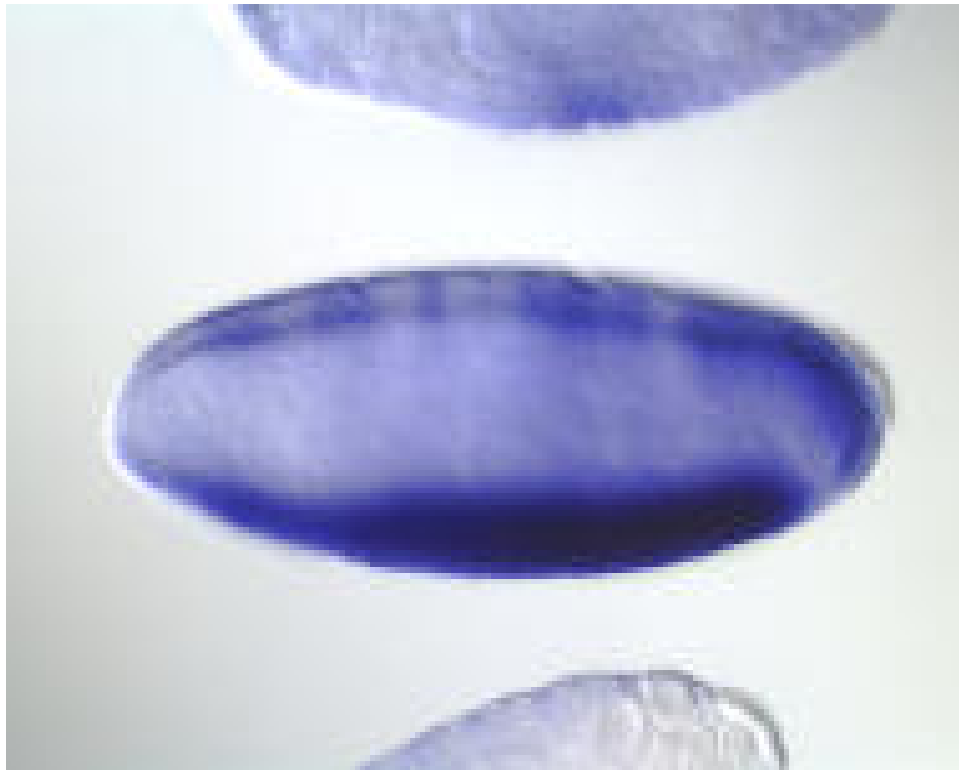

Location: Ventral Type: Promoter ZScore: -0.498264336 PValue: 0.618297741

Supplement: S3 File — Reports consist of in situ hybridization images, ATAC-seq traces, and calculated p-value and Z Score for each region used in the final analysis. (ZIP) [file pgen.1007367.s015.zip › S3_File/CG32982_Report.pdf]

# CG34383

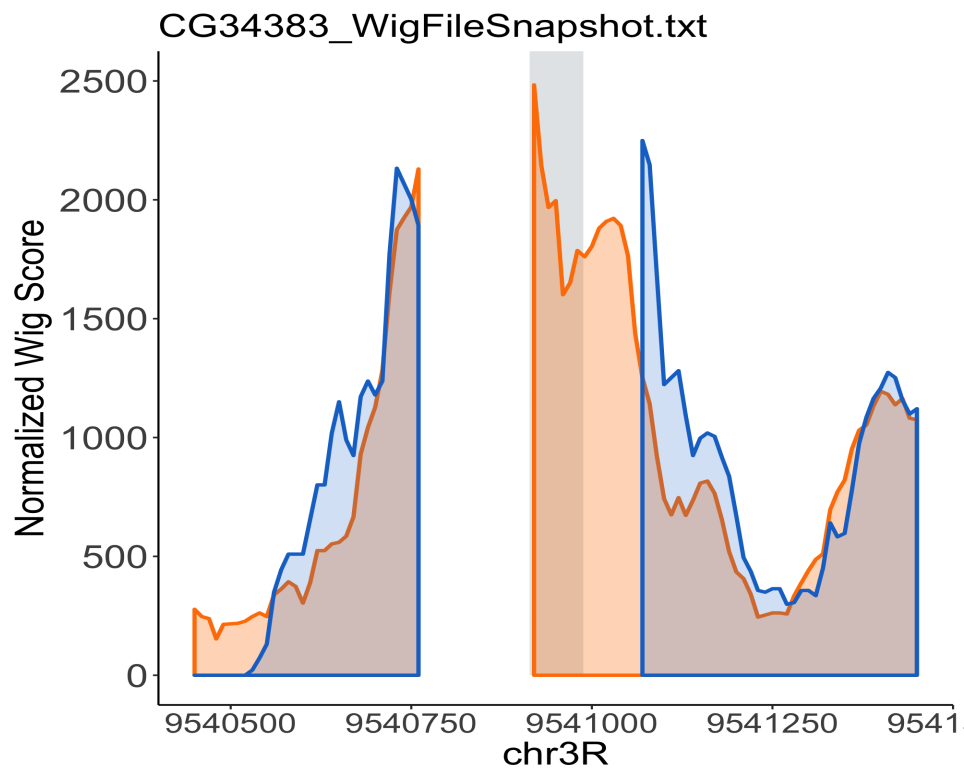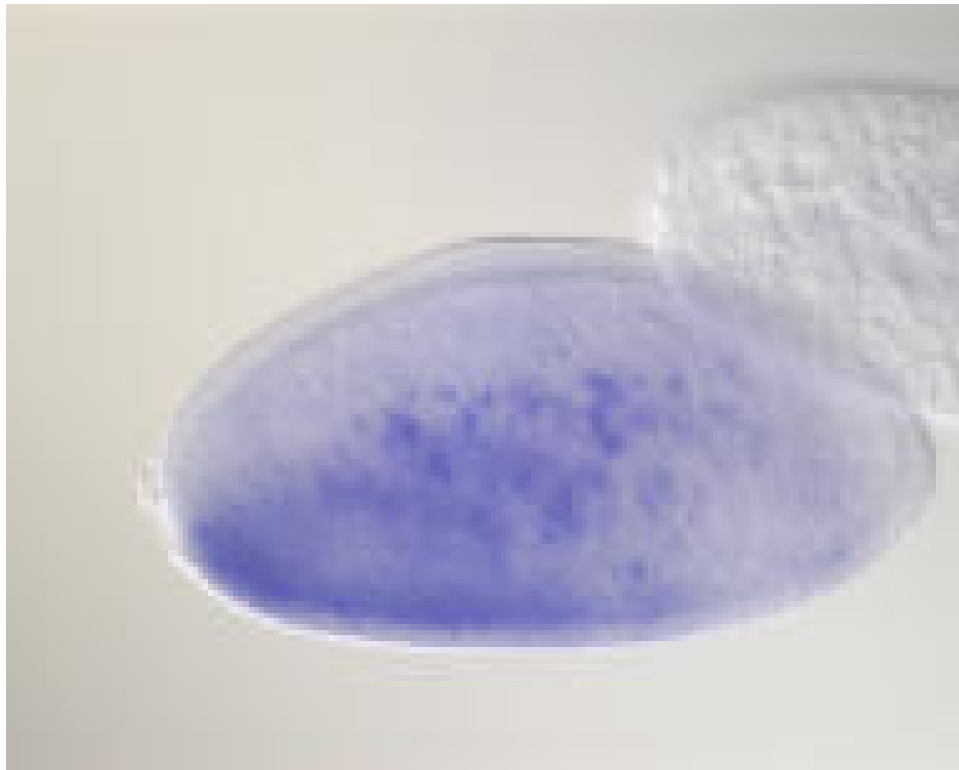

Location: Mostly Post Type: Promoter ZScore: 1.299074964 PValue: 0.193918204

Supplement: S3 File — Reports consist of in situ hybridization images, ATAC-seq traces, and calculated p-value and Z Score for each region used in the final analysis. (ZIP) [file pgen.1007367.s015.zip › S3_File/CG34383_Report.pdf]

CG3502

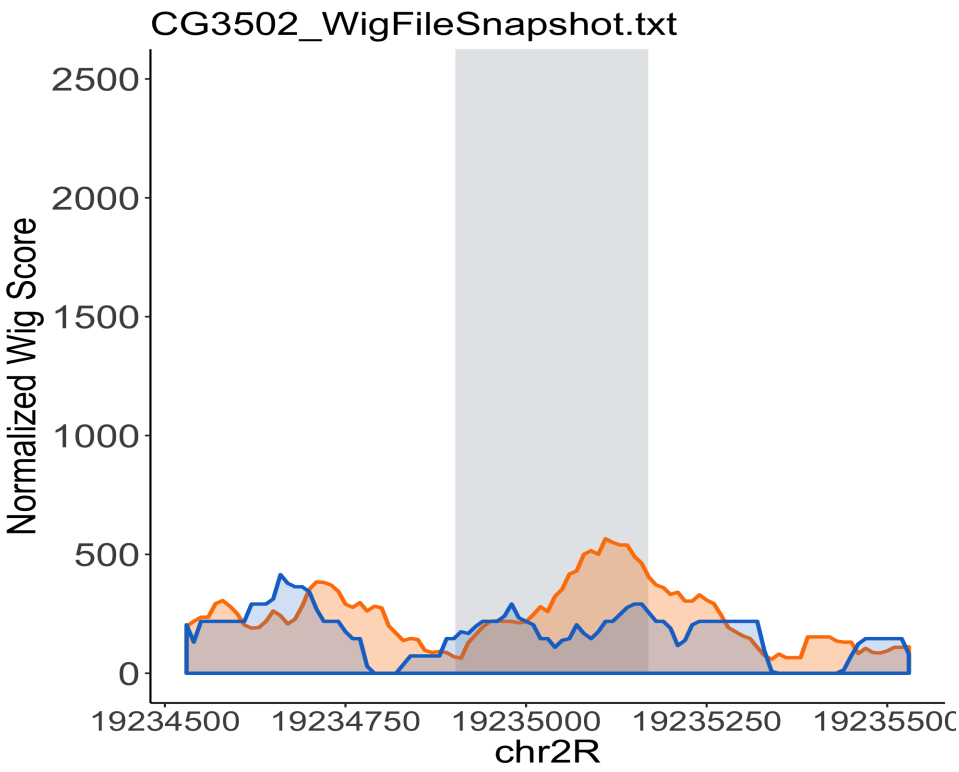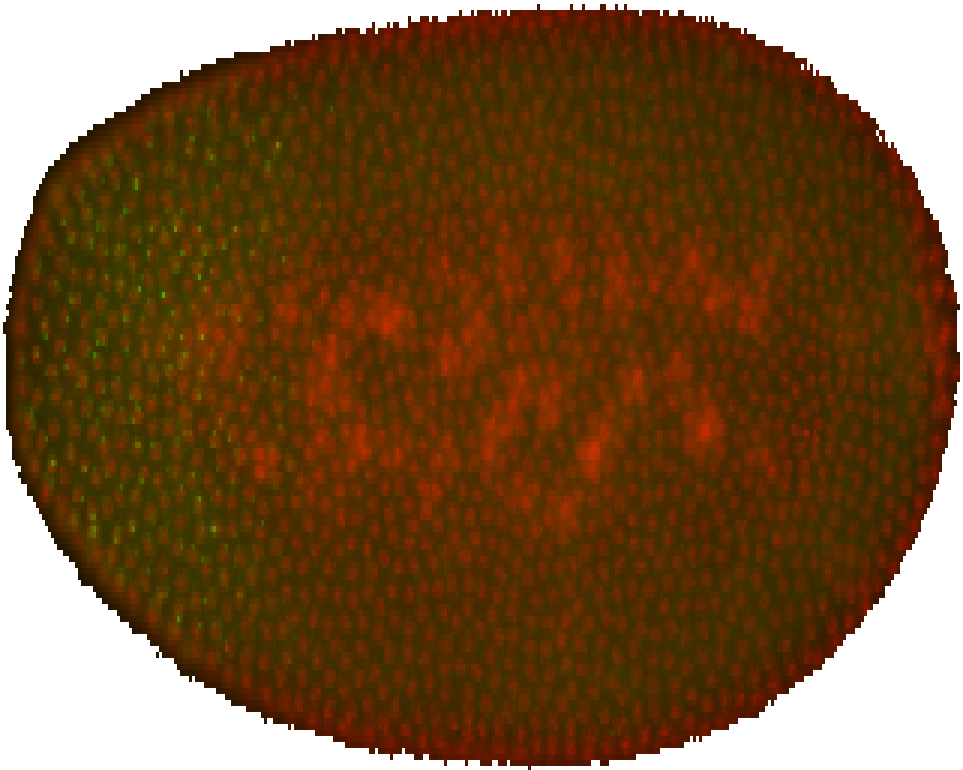

Location: Anterior Type: Promoter ZScore: 1.060549904 PValue: 0.288894499

Supplement: S3 File — Reports consist of in situ hybridization images, ATAC-seq traces, and calculated p-value and Z Score for each region used in the final analysis. (ZIP) [file pgen.1007367.s015.zip › S3_File/CG3502_Report.pdf]

CG4221

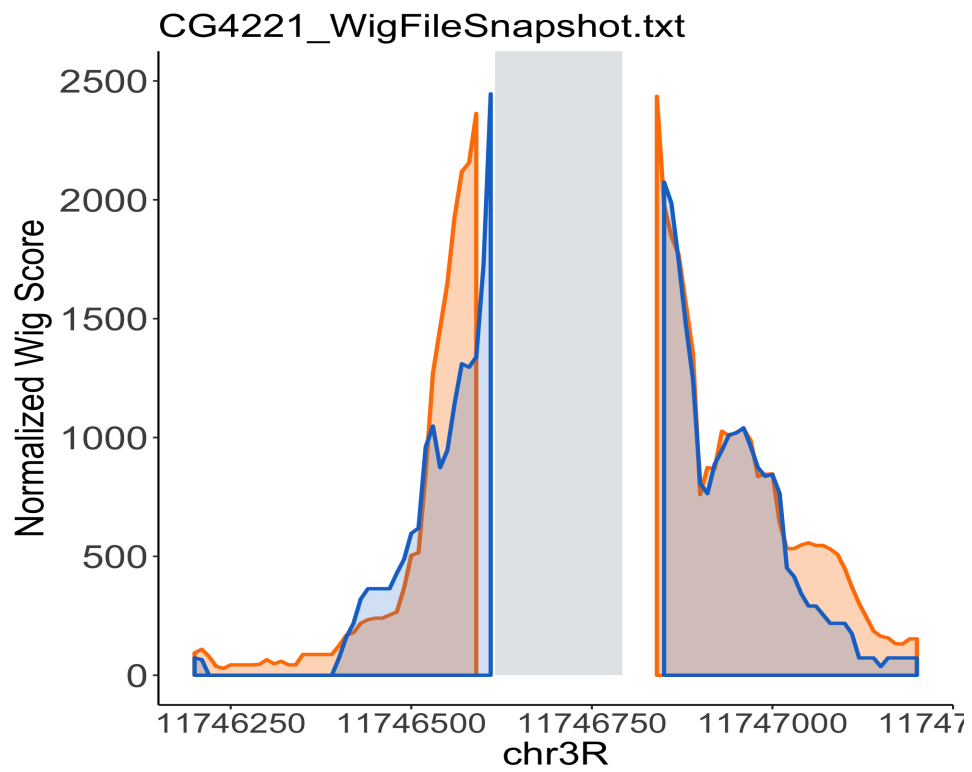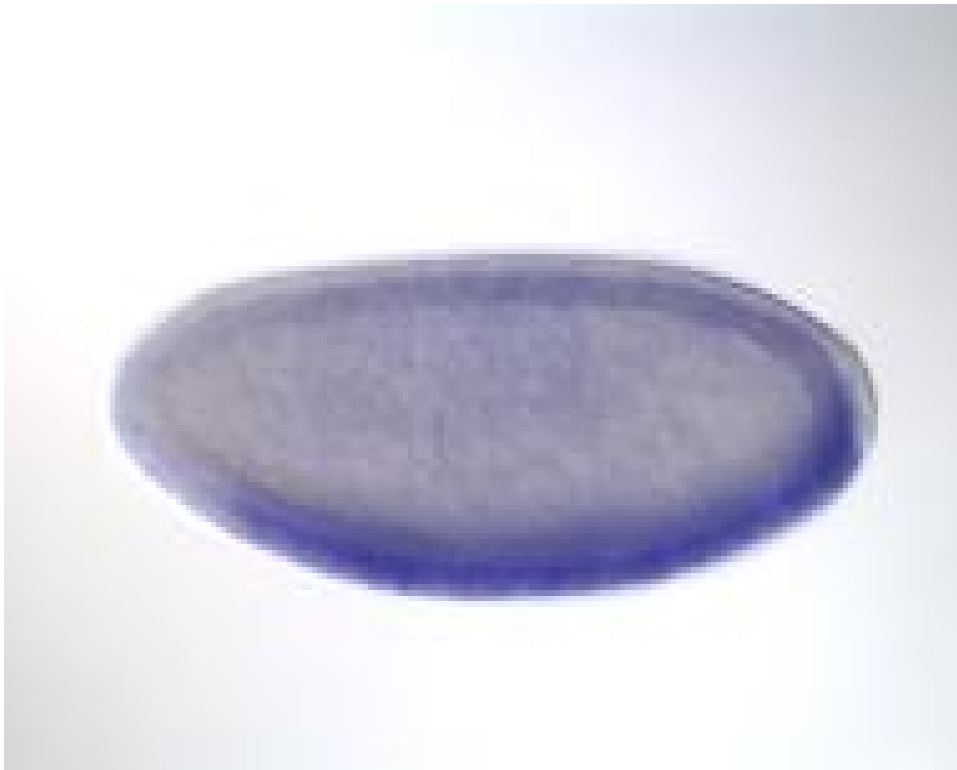

Location: Ventral Type: Promoter ZScore: -0.442339451 PValue: 0.658243587

Supplement: S3 File — Reports consist of in situ hybridization images, ATAC-seq traces, and calculated p-value and Z Score for each region used in the final analysis. (ZIP) [file pgen.1007367.s015.zip › S3_File/CG4221_Report.pdf]

## CG4221\_Sandmann

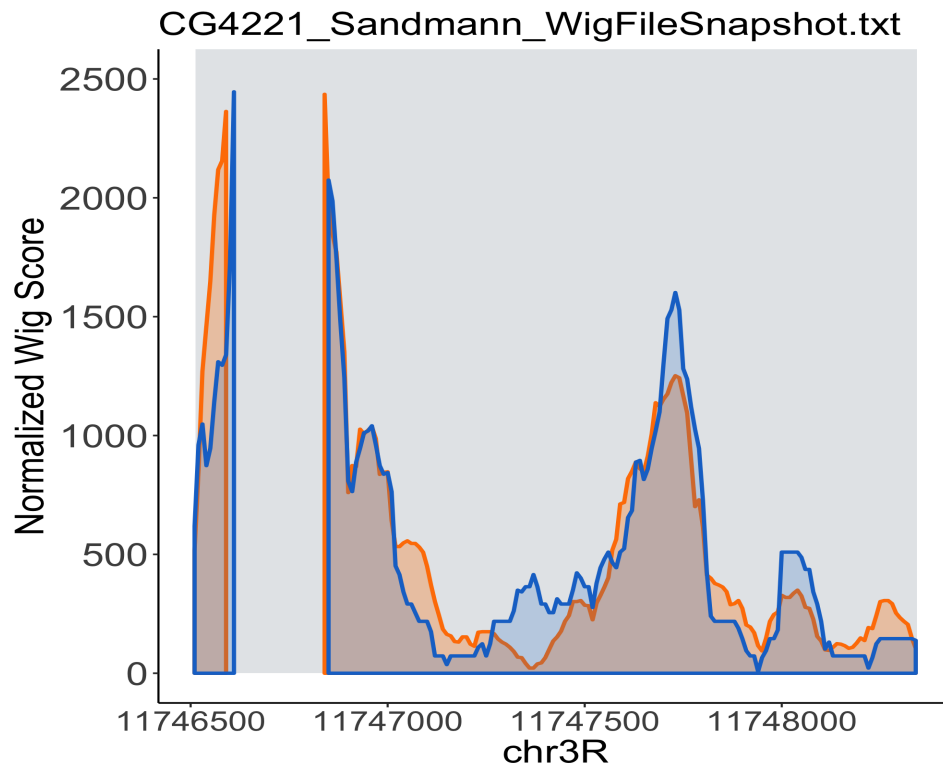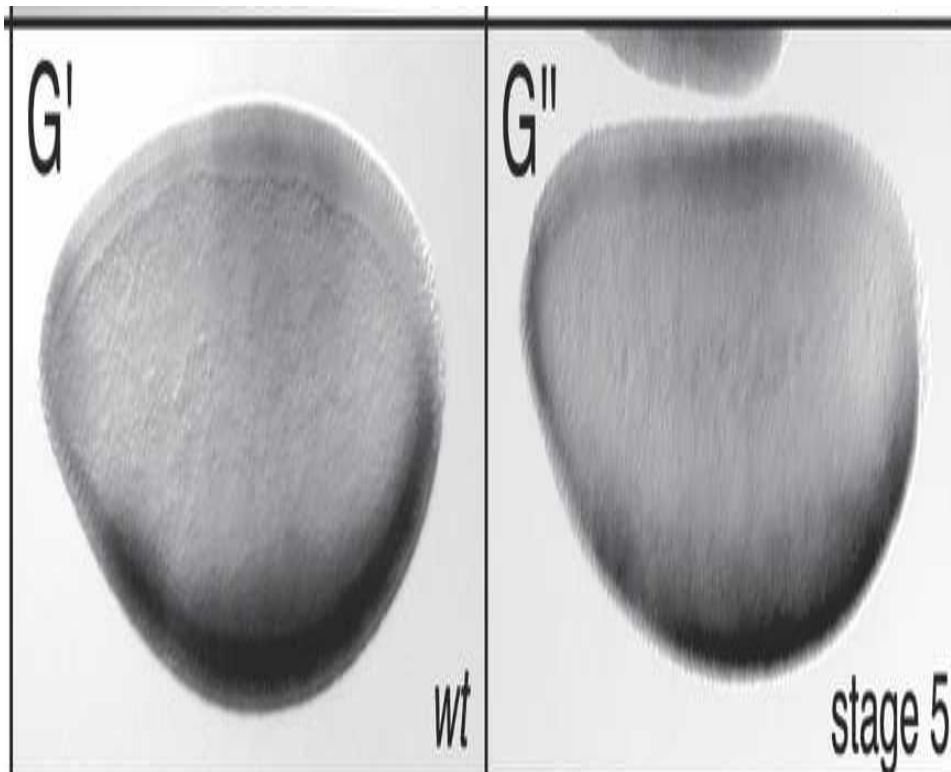

Location: Ventral Type: Enhancer ZScore: -0.199792857 PValue: 0.841642588

Supplement: S3 File — Reports consist of in situ hybridization images, ATAC-seq traces, and calculated p-value and Z Score for each region used in the final analysis. (ZIP) [file pgen.1007367.s015.zip › S3_File/CG4221_Sandmann_Report.pdf]

CG5522

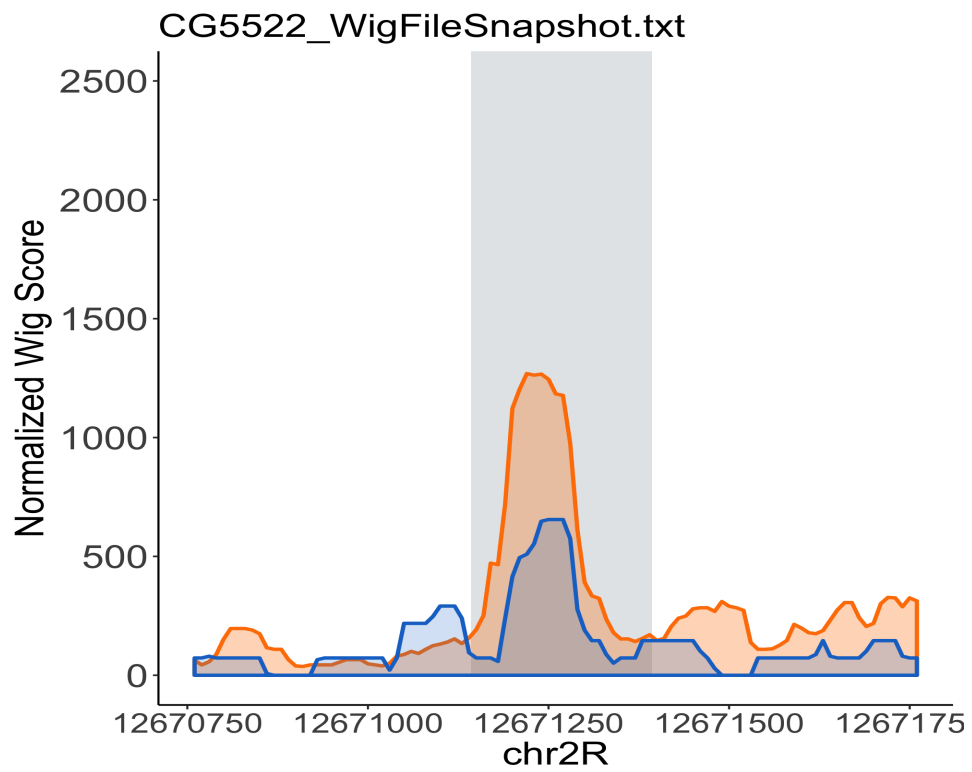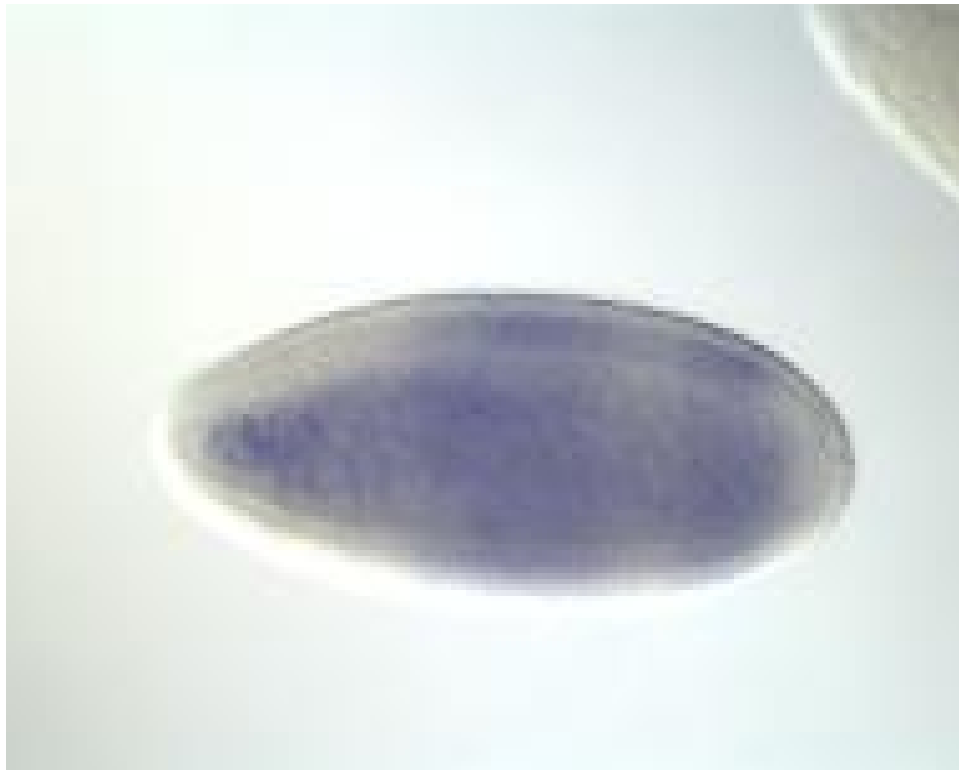

Location: Ventral Type: Promoter ZScore: 1.679830878 PValue: 0.092990225

Supplement: S3 File — Reports consist of in situ hybridization images, ATAC-seq traces, and calculated p-value and Z Score for each region used in the final analysis. (ZIP) [file pgen.1007367.s015.zip › S3_File/CG5522_Report.pdf]

# CG5888

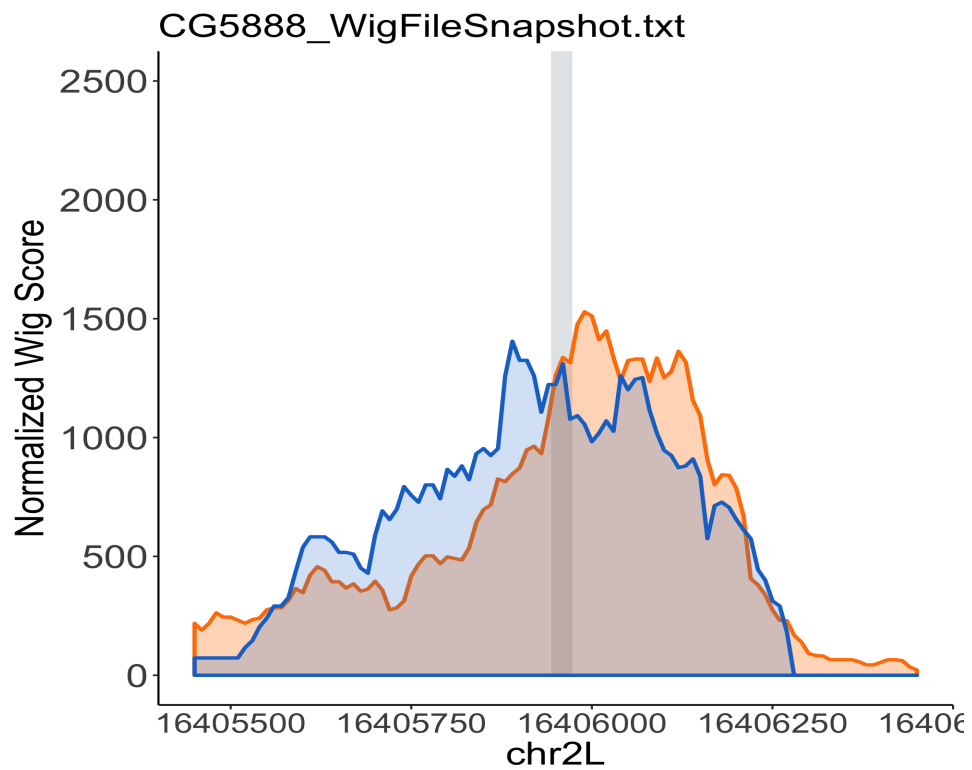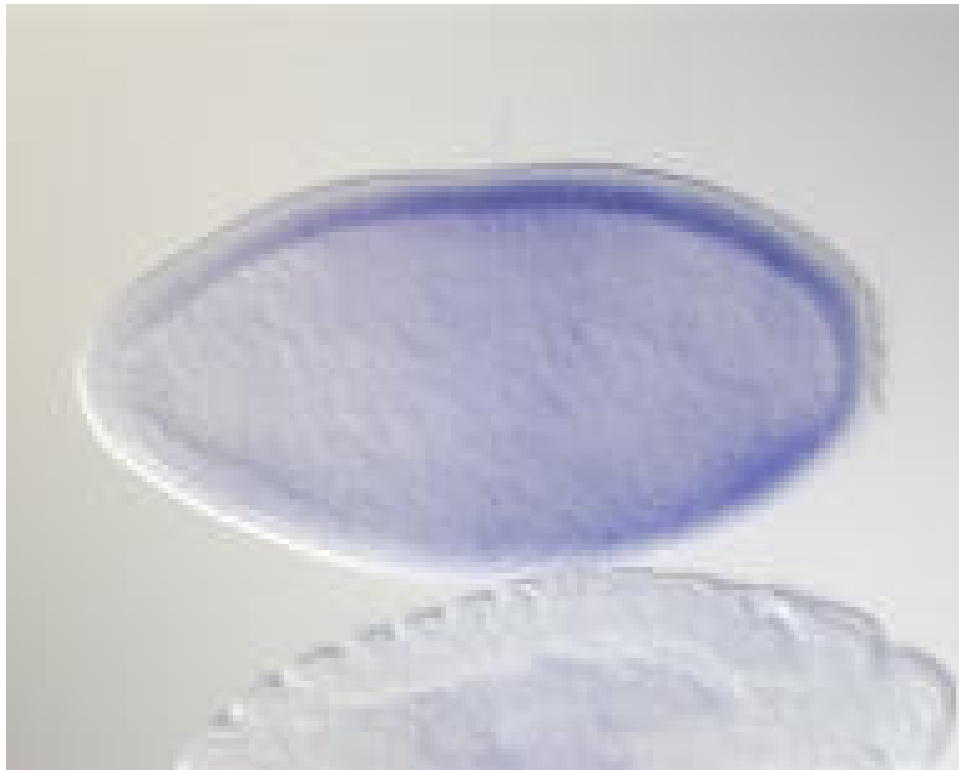

Location: Mostly Post Type: Promoter ZScore: -0.121718452 PValue: 0.903121999

Supplement: S3 File — Reports consist of in situ hybridization images, ATAC-seq traces, and calculated p-value and Z Score for each region used in the final analysis. (ZIP) [file pgen.1007367.s015.zip › S3_File/CG5888_Report.pdf]

## CG6206

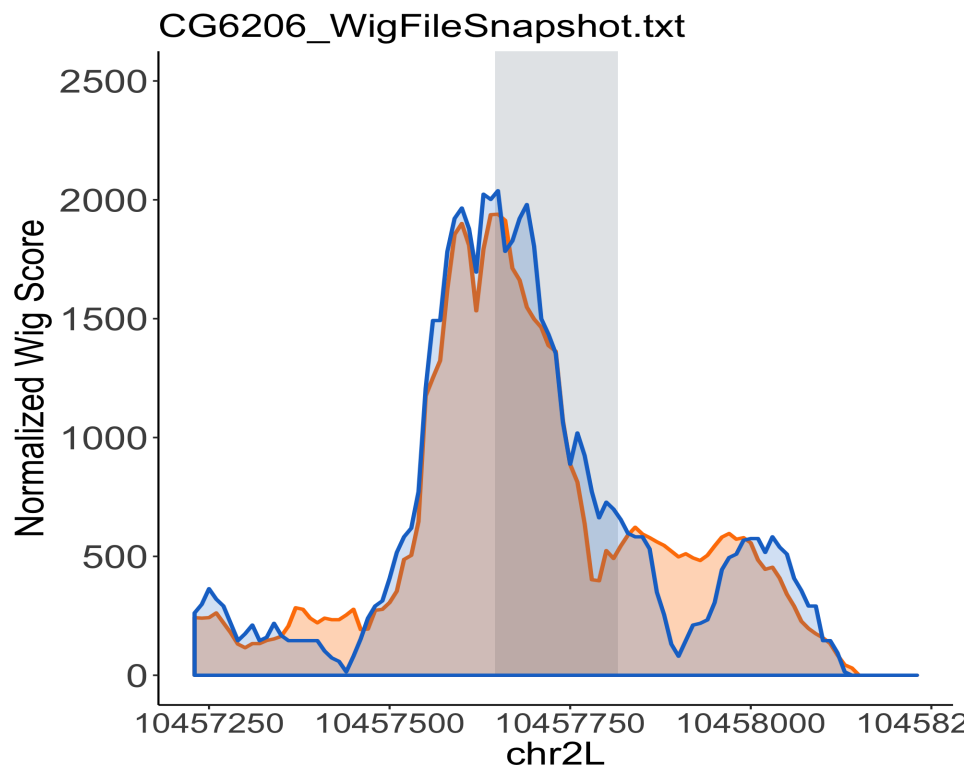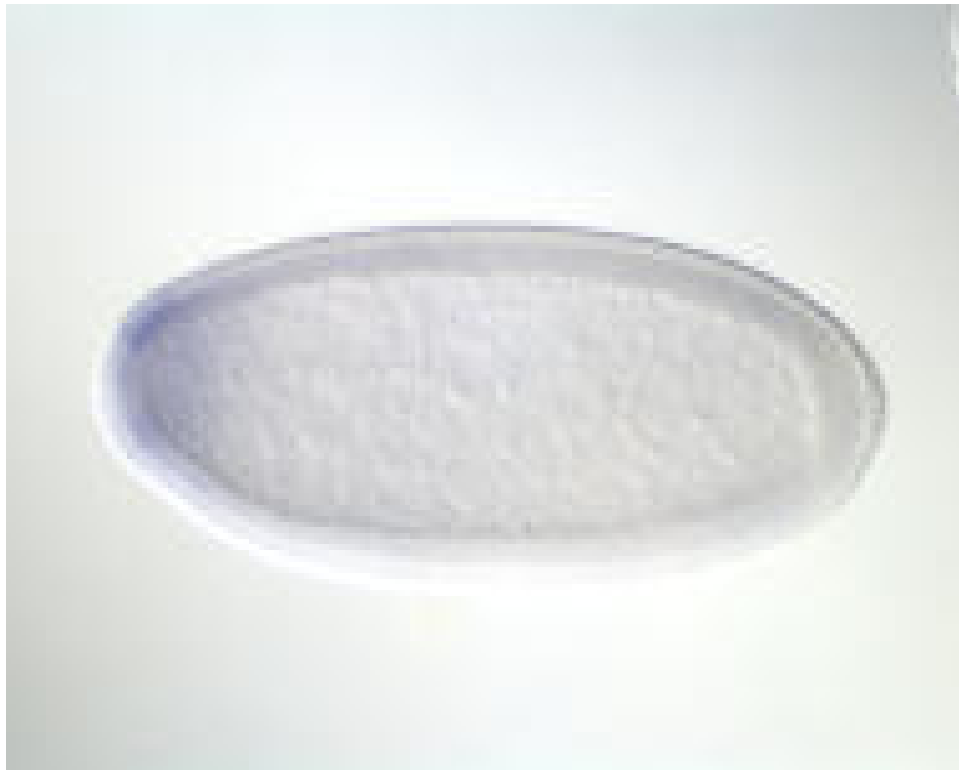

Location: Anterior Type: Promoter ZScore: -0.31959276 PValue: 0.749277064

Supplement: S3 File — Reports consist of in situ hybridization images, ATAC-seq traces, and calculated p-value and Z Score for each region used in the final analysis. (ZIP) [file pgen.1007367.s015.zip › S3_File/CG6206_Report.pdf]

## CG6966

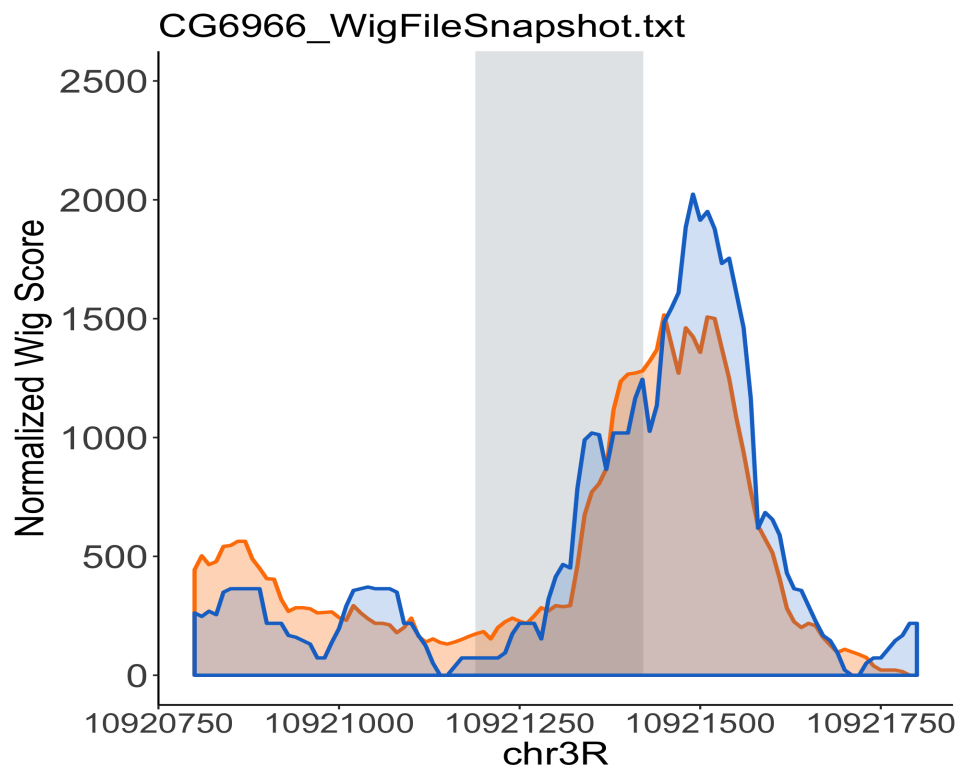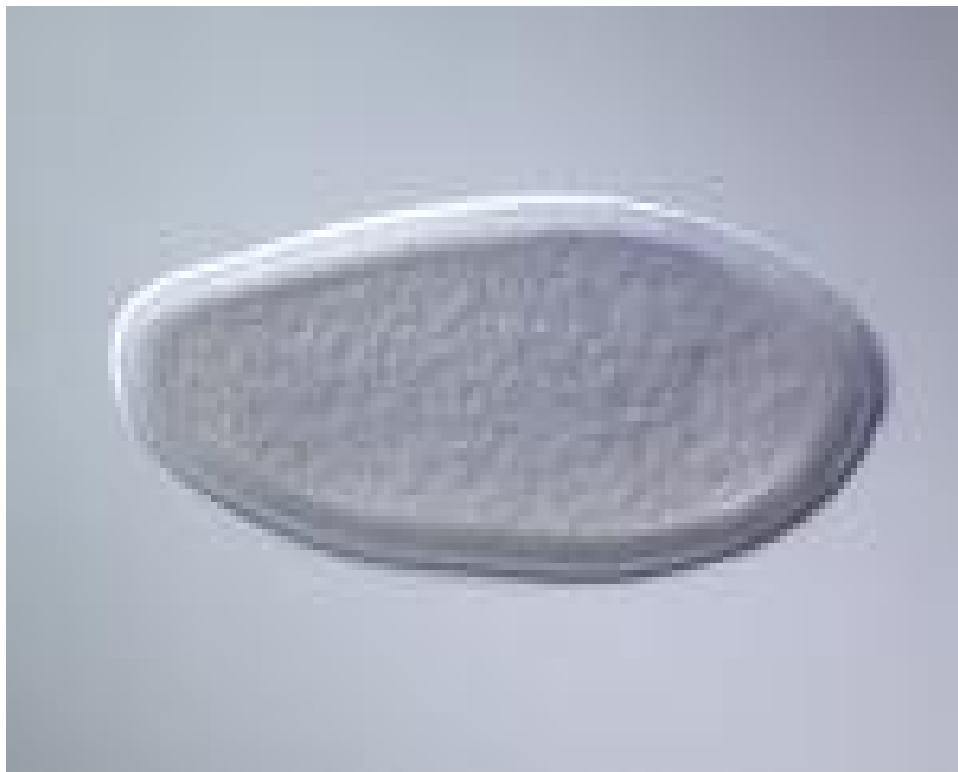

Location: Mostly Post Type: Promoter ZScore: -0.042134981 PValue: 0.966391094

Supplement: S3 File — Reports consist of in situ hybridization images, ATAC-seq traces, and calculated p-value and Z Score for each region used in the final analysis. (ZIP) [file pgen.1007367.s015.zip › S3_File/CG6966_Report.pdf]

CG8312

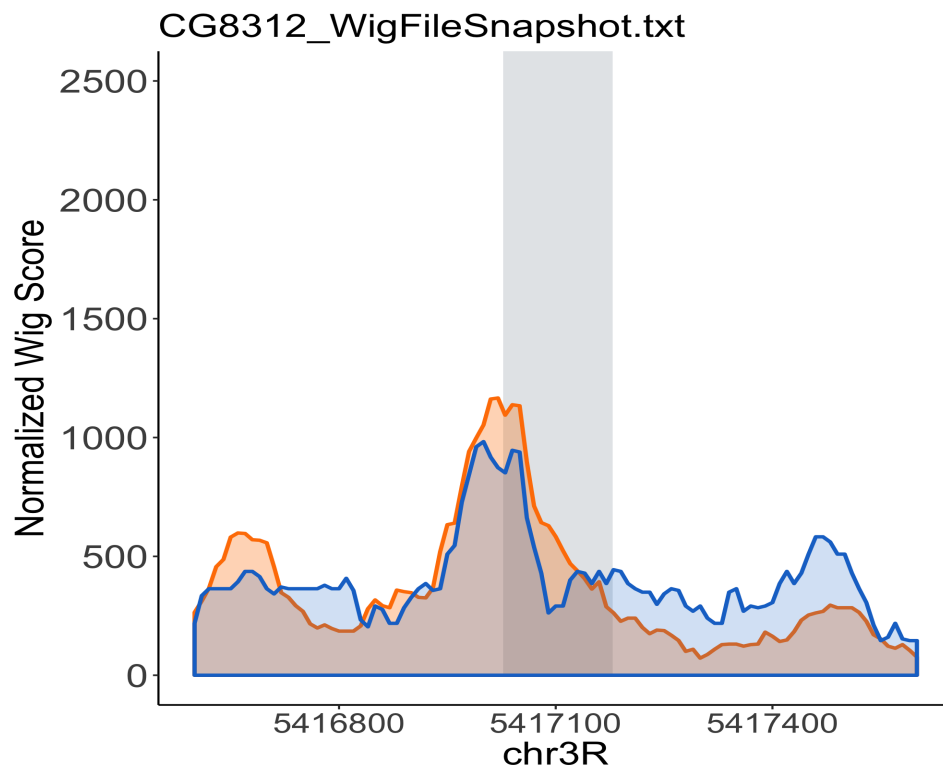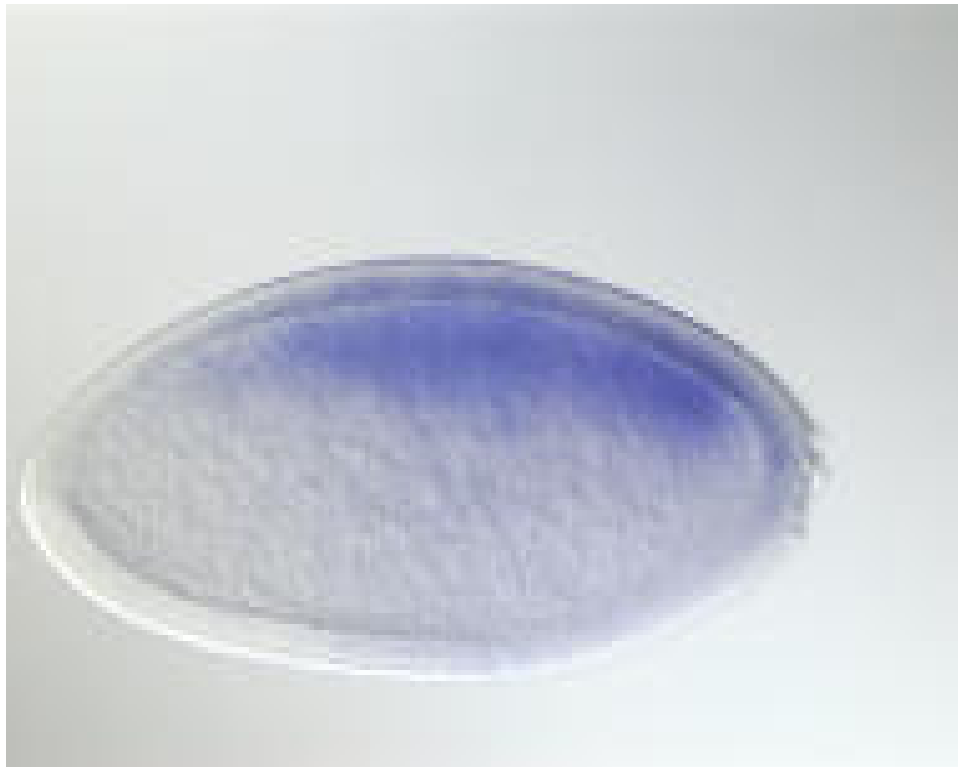

Location: Ventral Type: Promoter ZScore: 0.502701106 PValue: 0.615174432

Supplement: S3 File — Reports consist of in situ hybridization images, ATAC-seq traces, and calculated p-value and Z Score for each region used in the final analysis. (ZIP) [file pgen.1007367.s015.zip › S3_File/CG8312_Report.pdf]

## CG8668

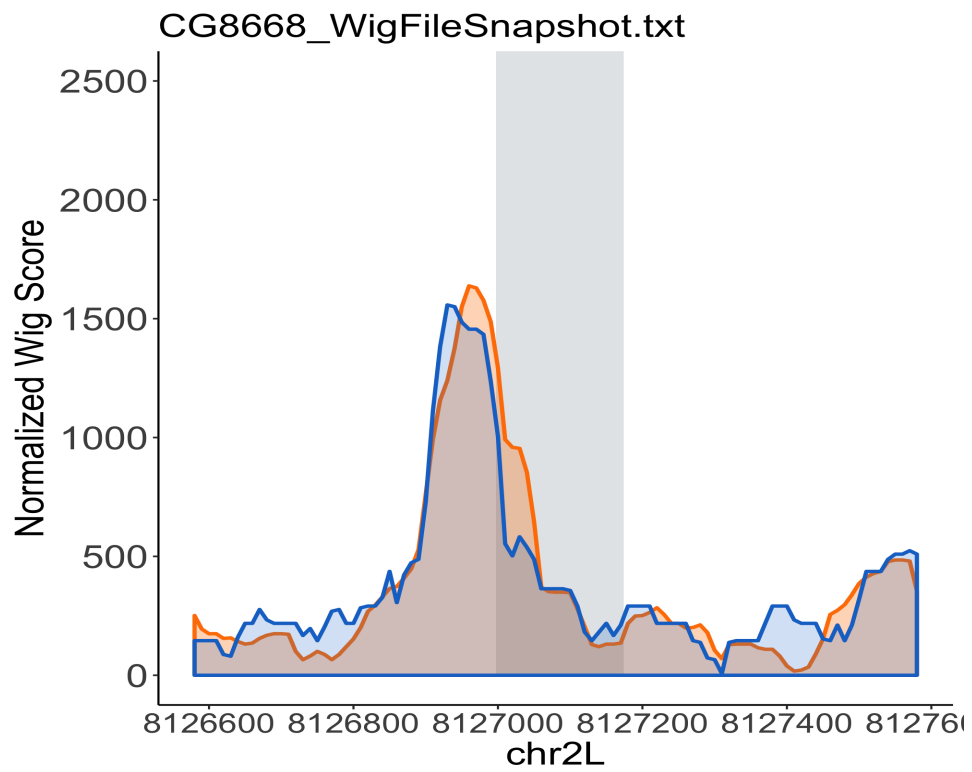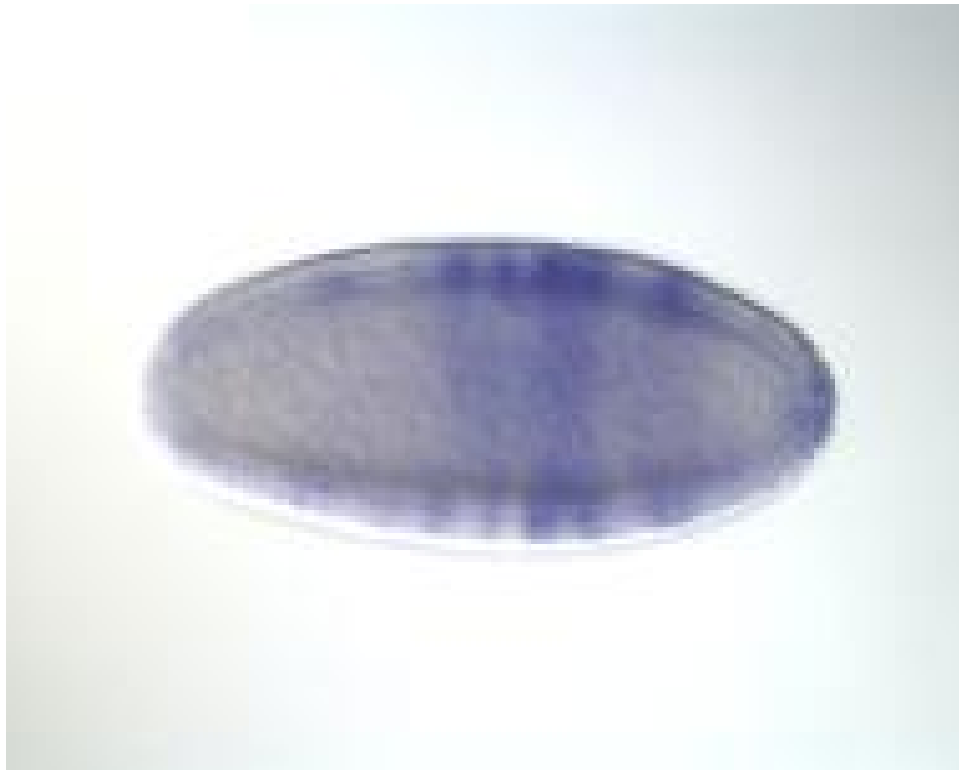

Location: Mostly Post Type: Promoter ZScore: -0.542136914 PValue: 0.587724191

Supplement: S3 File — Reports consist of in situ hybridization images, ATAC-seq traces, and calculated p-value and Z Score for each region used in the final analysis. (ZIP) [file pgen.1007367.s015.zip › S3_File/CG8668_Report.pdf]

# CG8788

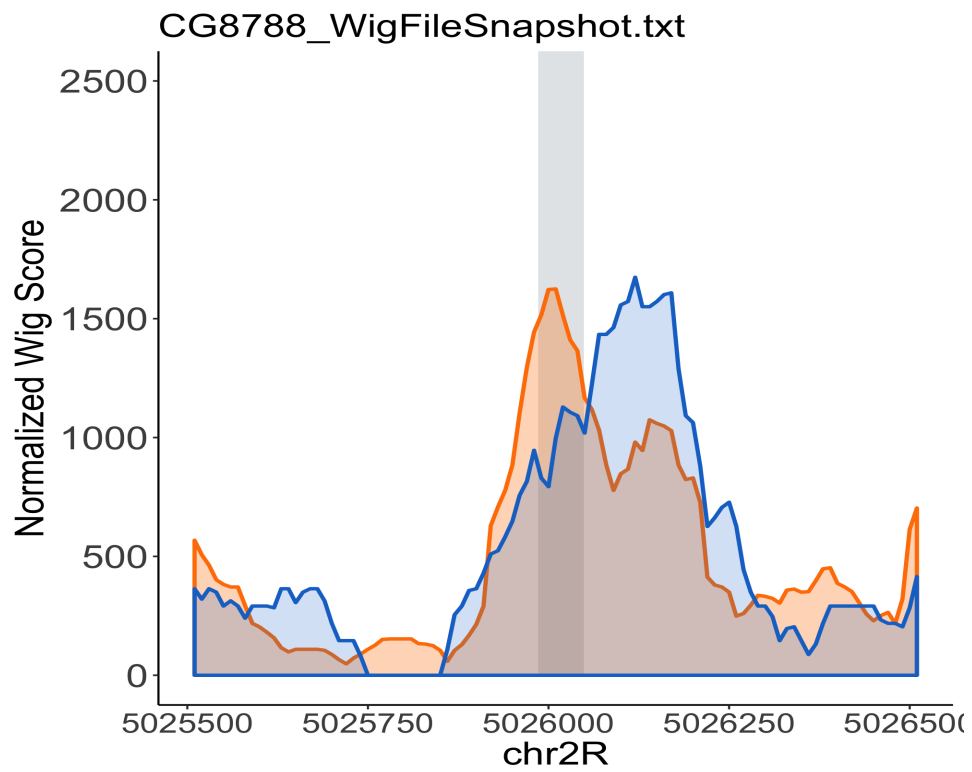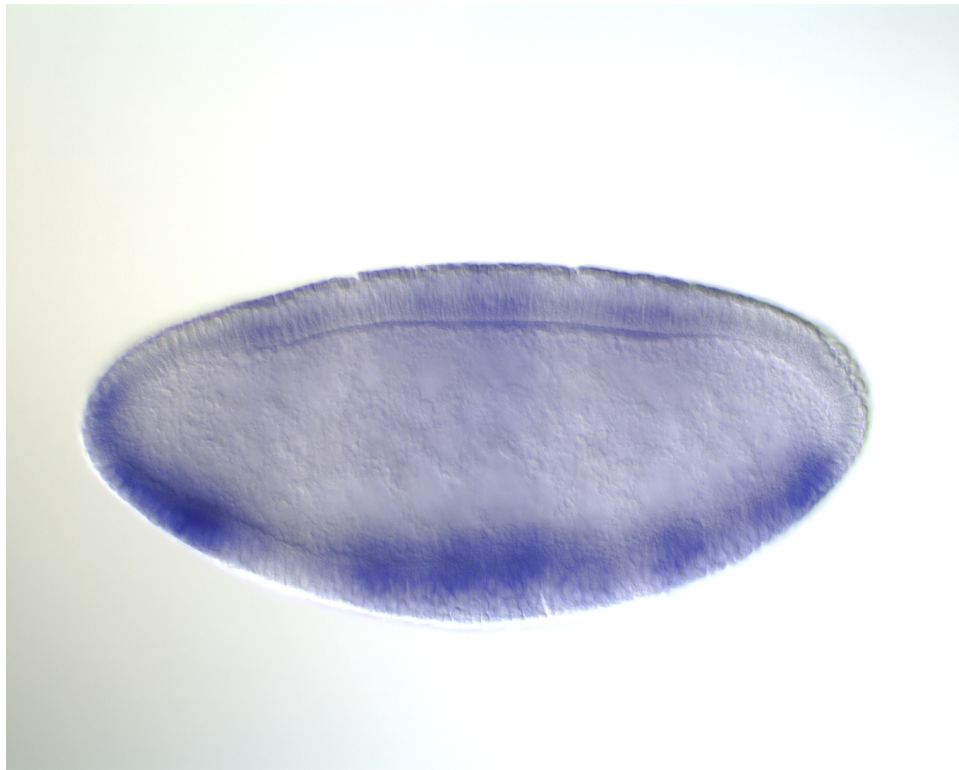

Location: Ventral Type: Promoter ZScore: 0.906480416 PValue: 0.364681626

Supplement: S3 File — Reports consist of in situ hybridization images, ATAC-seq traces, and calculated p-value and Z Score for each region used in the final analysis. (ZIP) [file pgen.1007367.s015.zip › S3_File/CG8788_Report.pdf]

# CG8788\_Sandmann

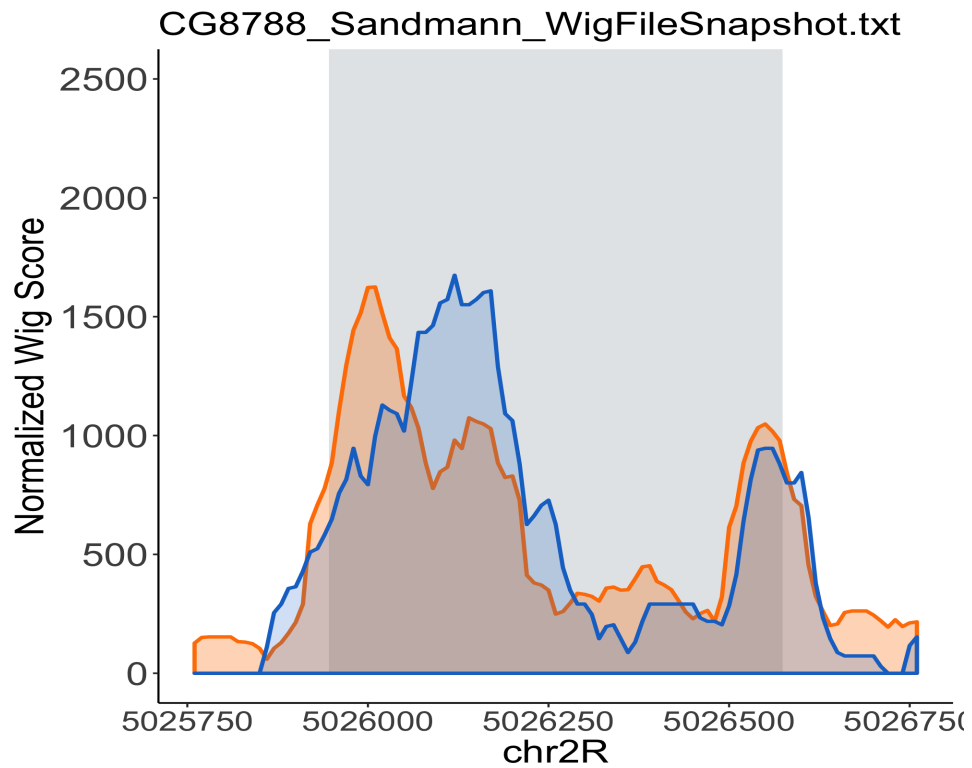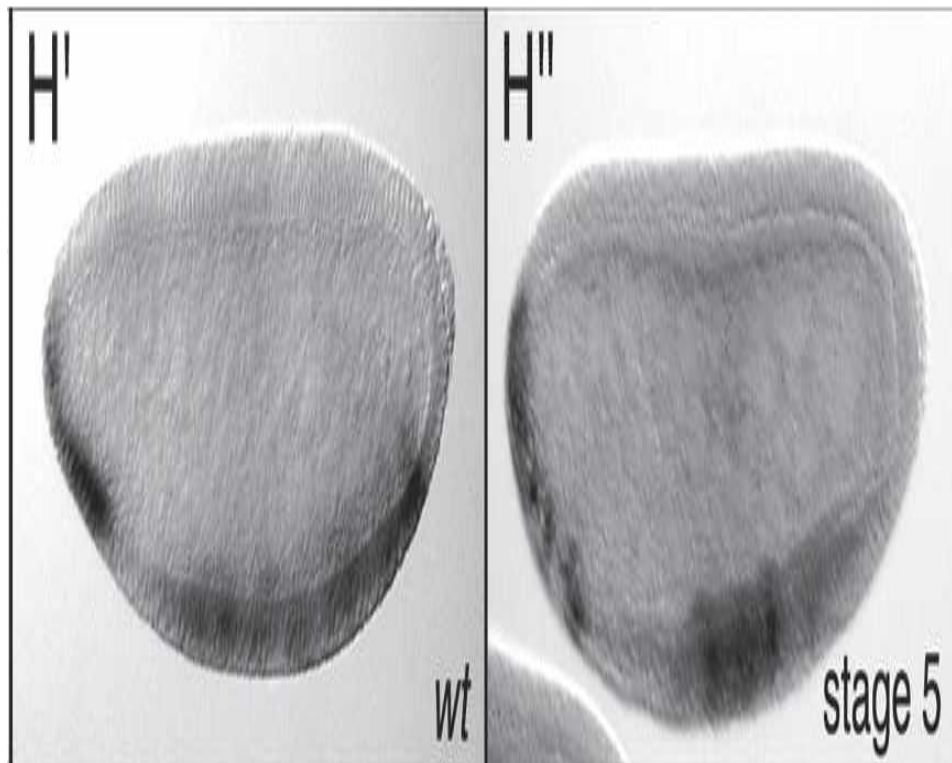

Location: Ventral Type: Enhancer ZScore: -0.09139438 PValue: 0.927179227

Supplement: S3 File — Reports consist of in situ hybridization images, ATAC-seq traces, and calculated p-value and Z Score for each region used in the final analysis. (ZIP) [file pgen.1007367.s015.zip › S3_File/CG8788_Sandmann_Report.pdf]

CG8964

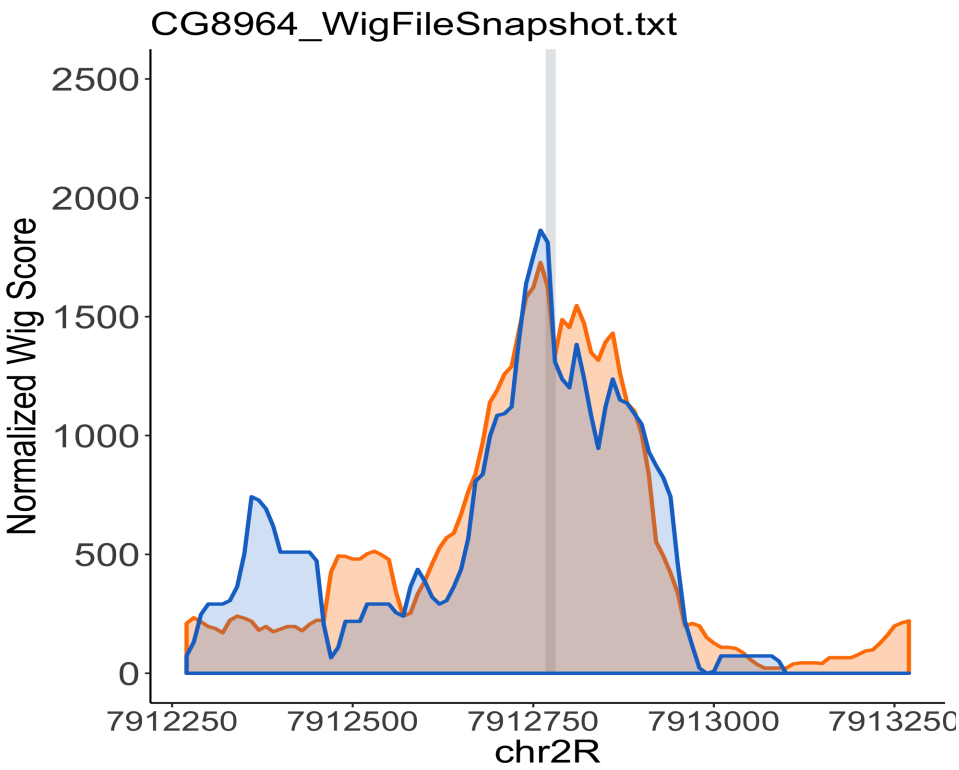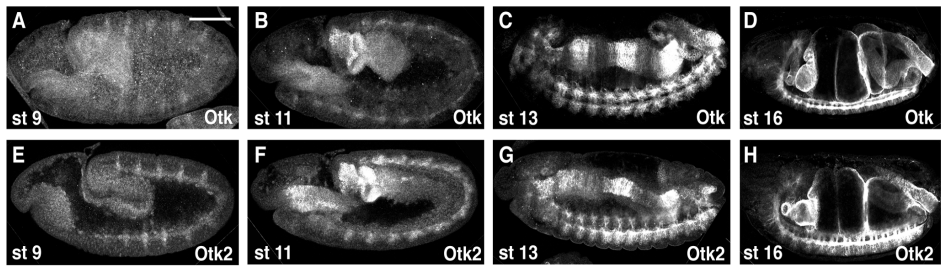

Location: Dorsal Type: Promoter ZScore: -0.185972784 PValue: 0.852466104

Supplement: S3 File — Reports consist of in situ hybridization images, ATAC-seq traces, and calculated p-value and Z Score for each region used in the final analysis. (ZIP) [file pgen.1007367.s015.zip › S3_File/CG8964_Report.pdf]

CG8965

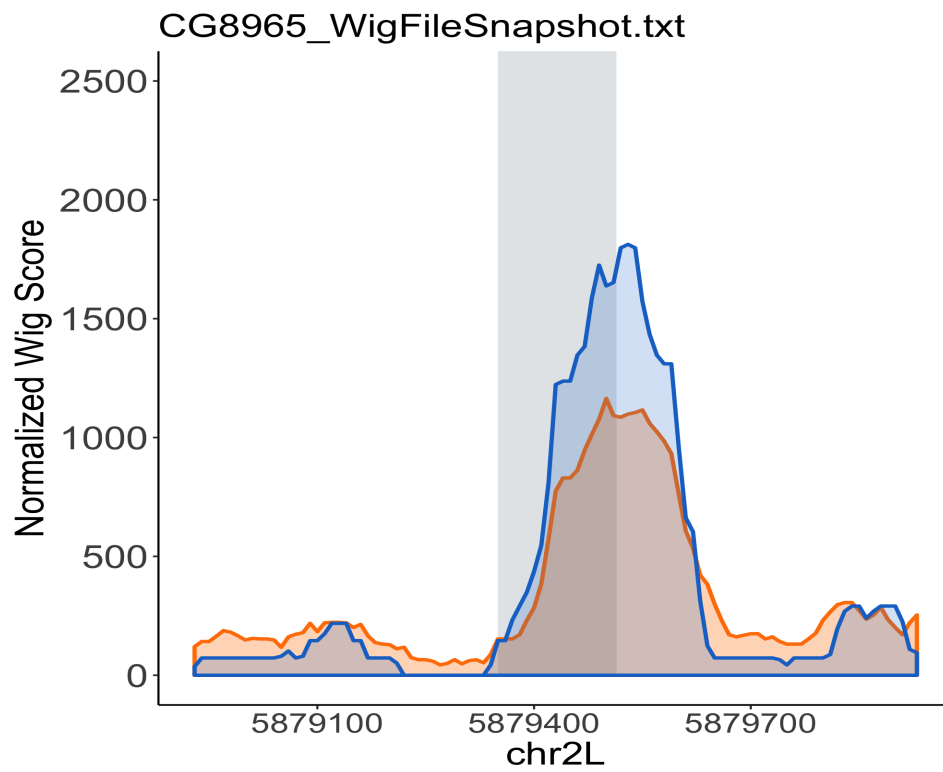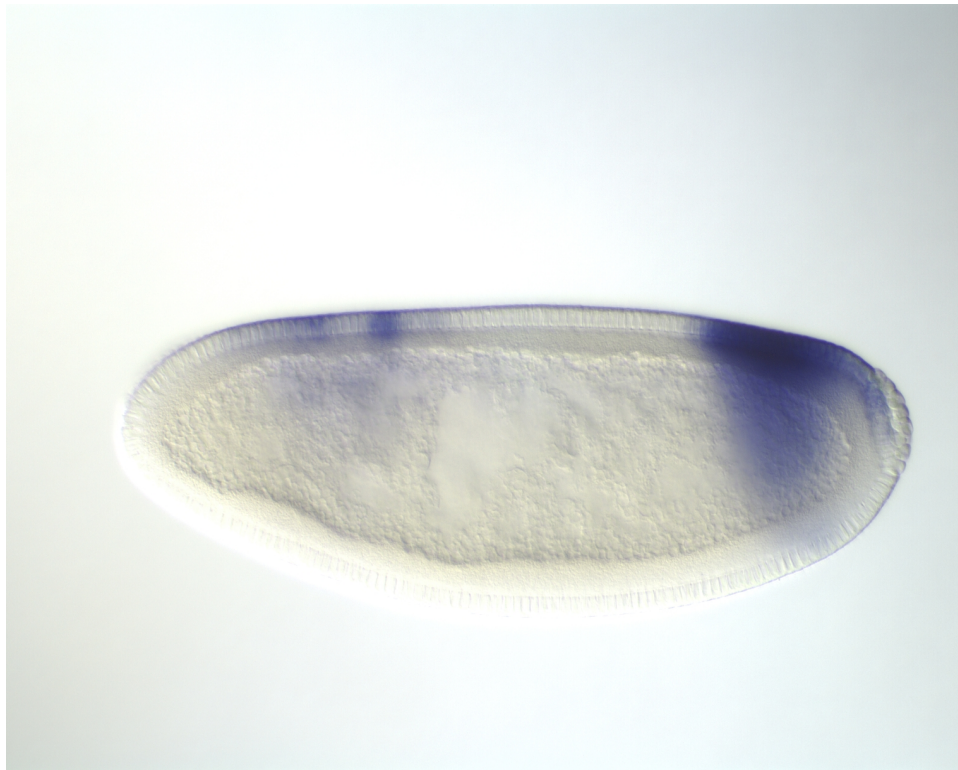

Location: Mostly Post Type: Promoter ZScore: 0.86610943 PValue: 0.386430154

Supplement: S3 File — Reports consist of in situ hybridization images, ATAC-seq traces, and calculated p-value and Z Score for each region used in the final analysis. (ZIP) [file pgen.1007367.s015.zip › S3_File/CG8965_Report.pdf]

## cnc\_+5\_construct\_67.5

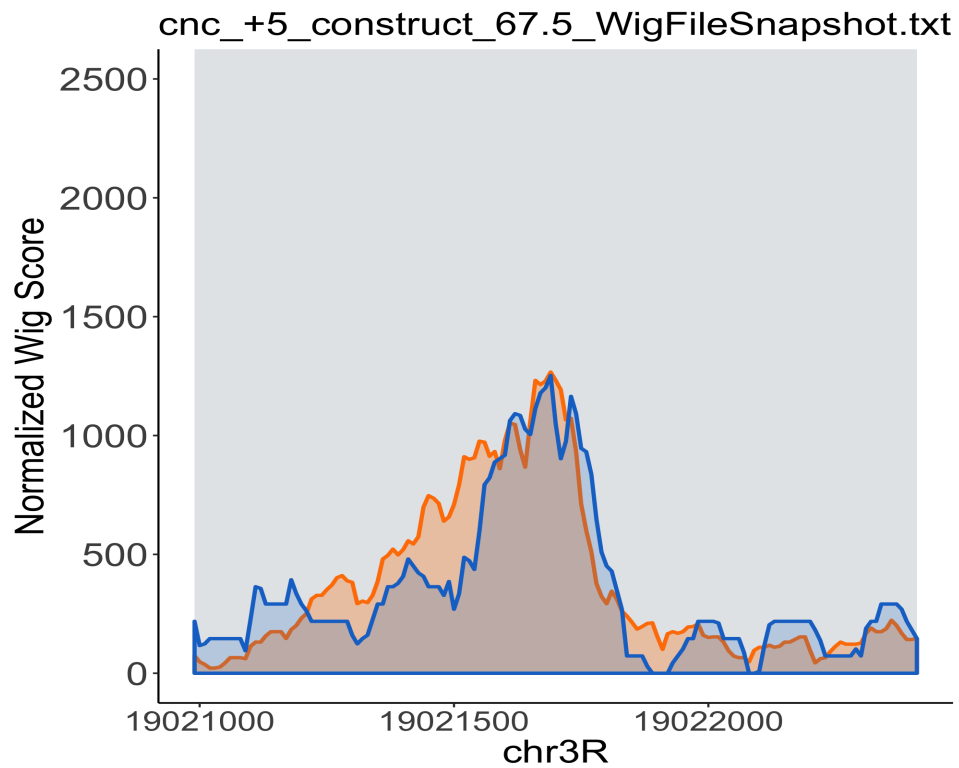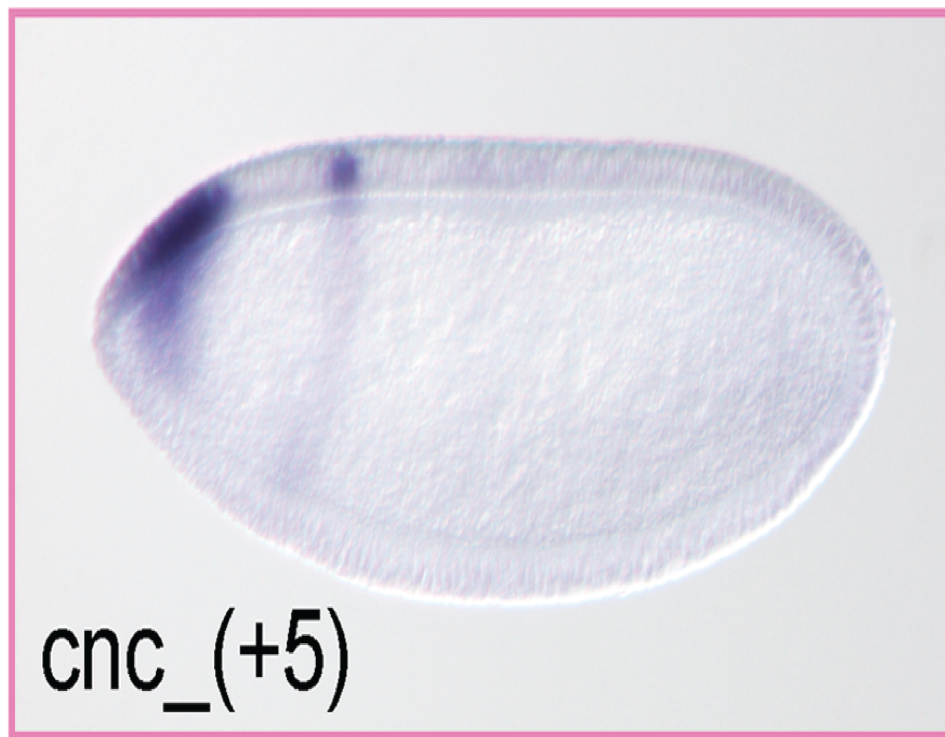

Location: Anterior Type: Enhancer ZScore: 0.132518366 PValue: 0.894574297

Supplement: S3 File — Reports consist of in situ hybridization images, ATAC-seq traces, and calculated p-value and Z Score for each region used in the final analysis. (ZIP) [file pgen.1007367.s015.zip › S3_File/cnc_+5_construct_67.5_Report.pdf]

Cnx99A\_Ozdemir

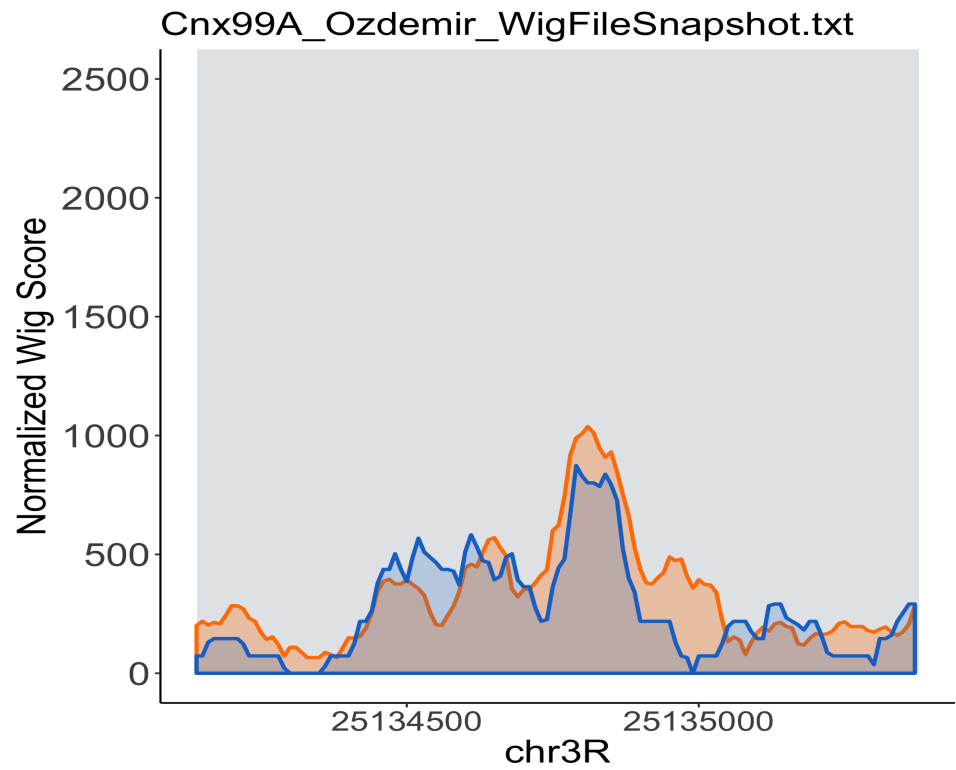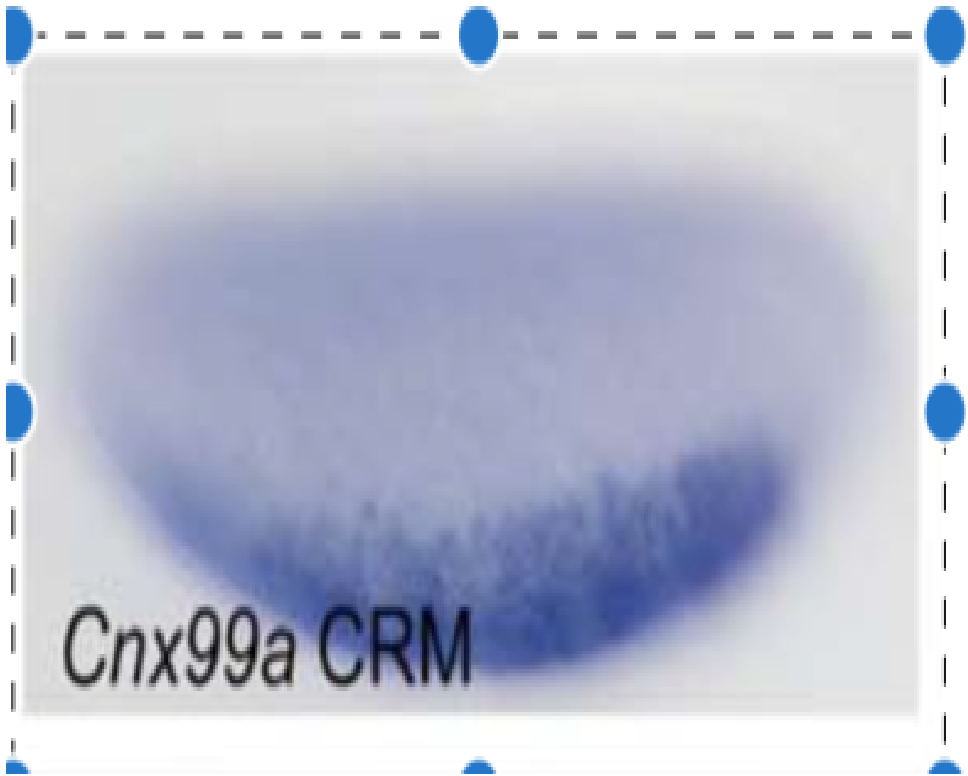

Location: Ventral Type: Enhancer ZScore: 0.425537445 PValue: 0.670444931

Supplement: S3 File — Reports consist of in situ hybridization images, ATAC-seq traces, and calculated p-value and Z Score for each region used in the final analysis. (ZIP) [file pgen.1007367.s015.zip › S3_File/Cnx99A_Ozdemir_Report.pdf]

# Cyp310a1\_Ozdemir

Cyp310a1\_Ozdemir\_WigFileSnapshot.txt

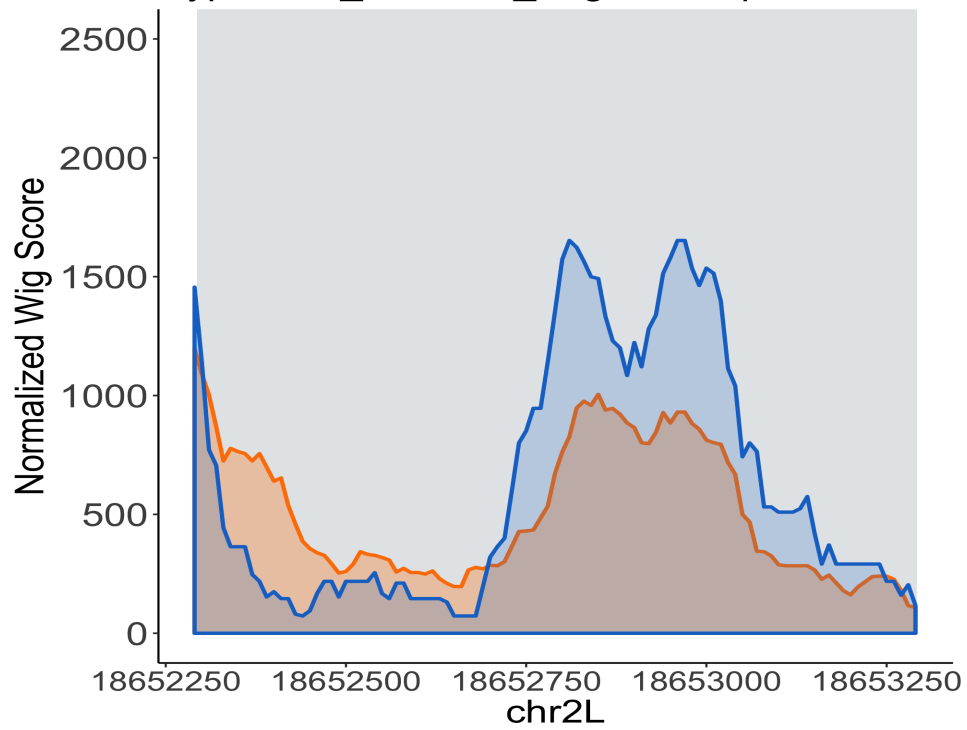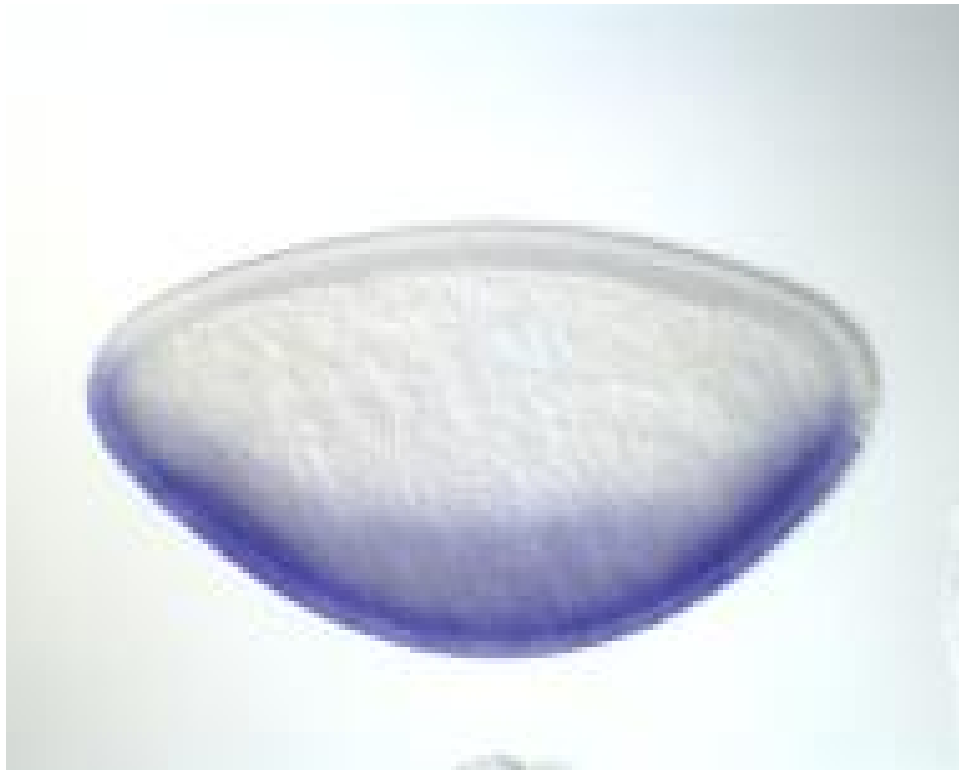

Location: Ventral Type: Enhancer ZScore: -0.544483879 PValue: 0.586108539

Supplement: S3 File — Reports consist of in situ hybridization images, ATAC-seq traces, and calculated p-value and Z Score for each region used in the final analysis. (ZIP) [file pgen.1007367.s015.zip › S3_File/Cyp310a1_Ozdemir_Report.pdf]

# Cyp310a1

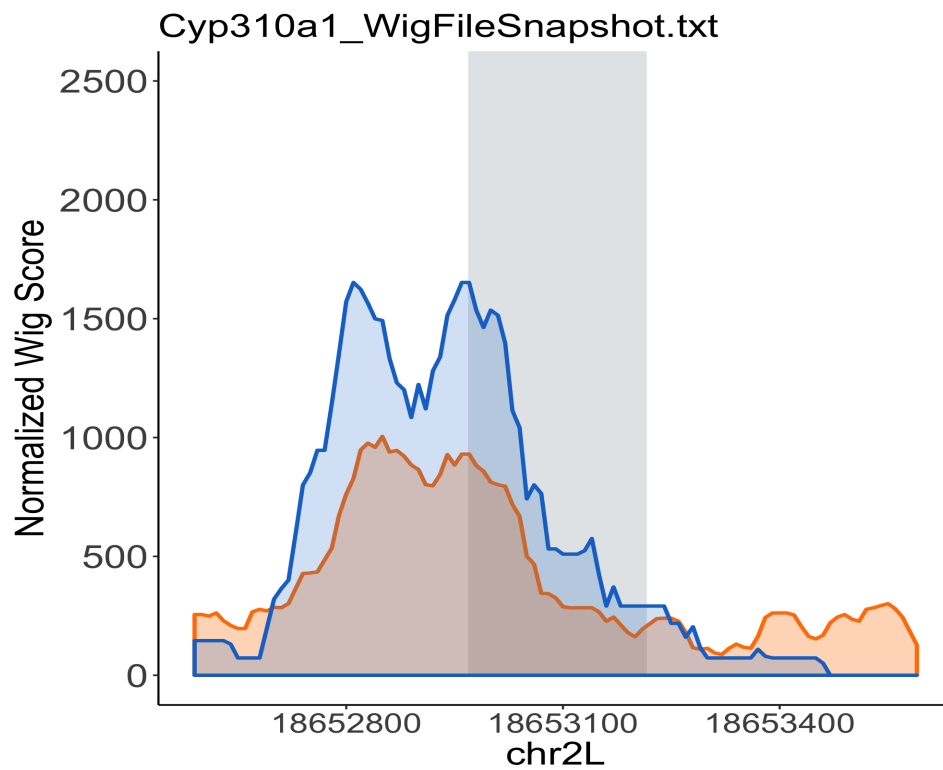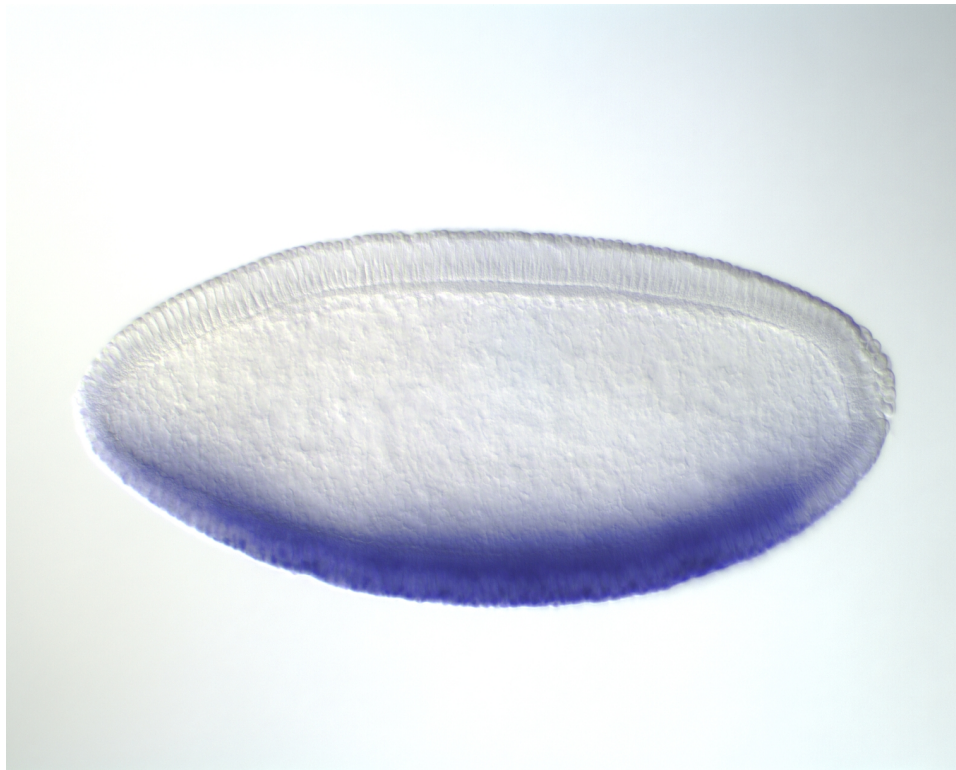

Location: Ventral Type: Promoter ZScore: -1.262425202 PValue: 0.206795826

Supplement: S3 File — Reports consist of in situ hybridization images, ATAC-seq traces, and calculated p-value and Z Score for each region used in the final analysis. (ZIP) [file pgen.1007367.s015.zip › S3_File/Cyp310a1_Report.pdf]

dan

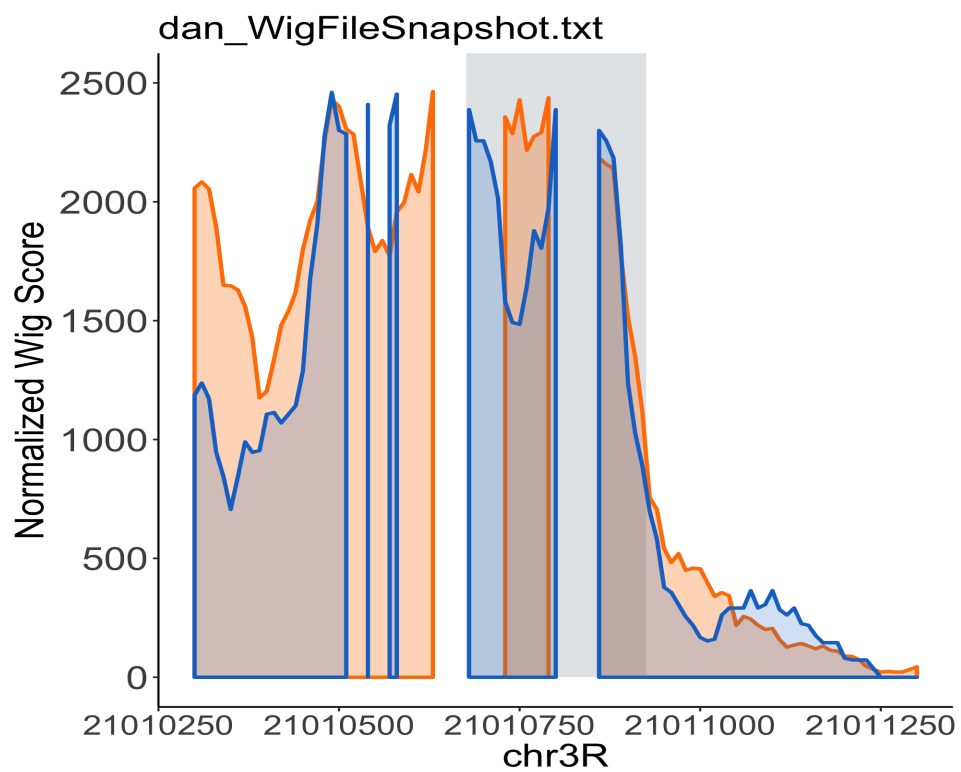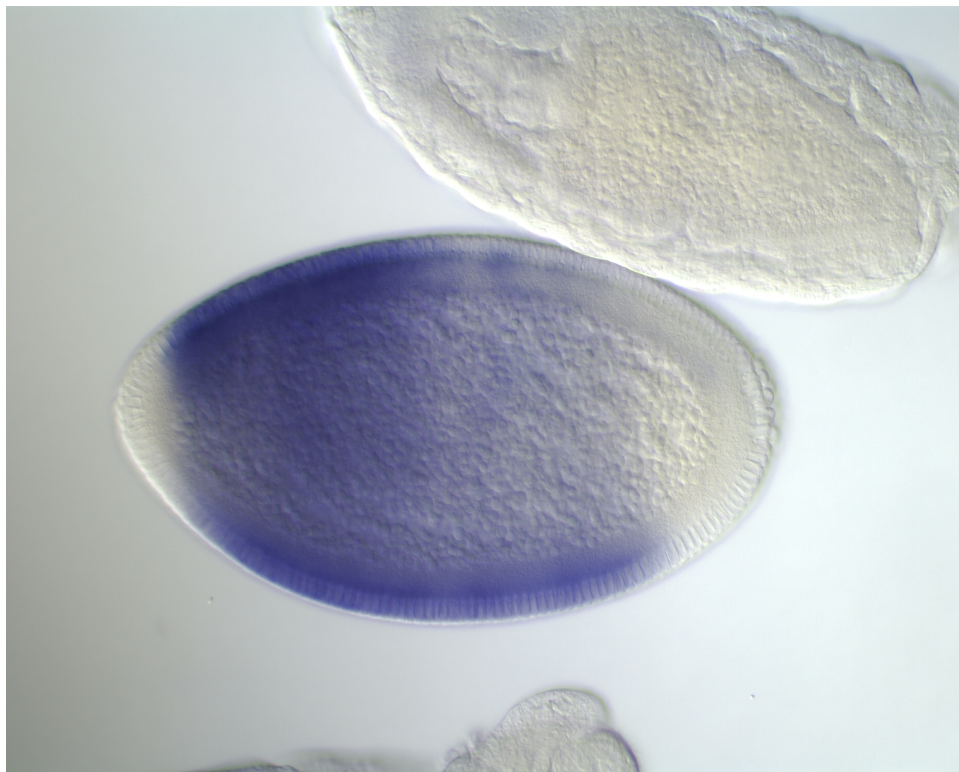

Location: Mostly Ant Type: Promoter ZScore: 0.268463837 PValue: 0.788342307

Supplement: S3 File — Reports consist of in situ hybridization images, ATAC-seq traces, and calculated p-value and Z Score for each region used in the final analysis. (ZIP) [file pgen.1007367.s015.zip › S3_File/dan_Report.pdf]

danr

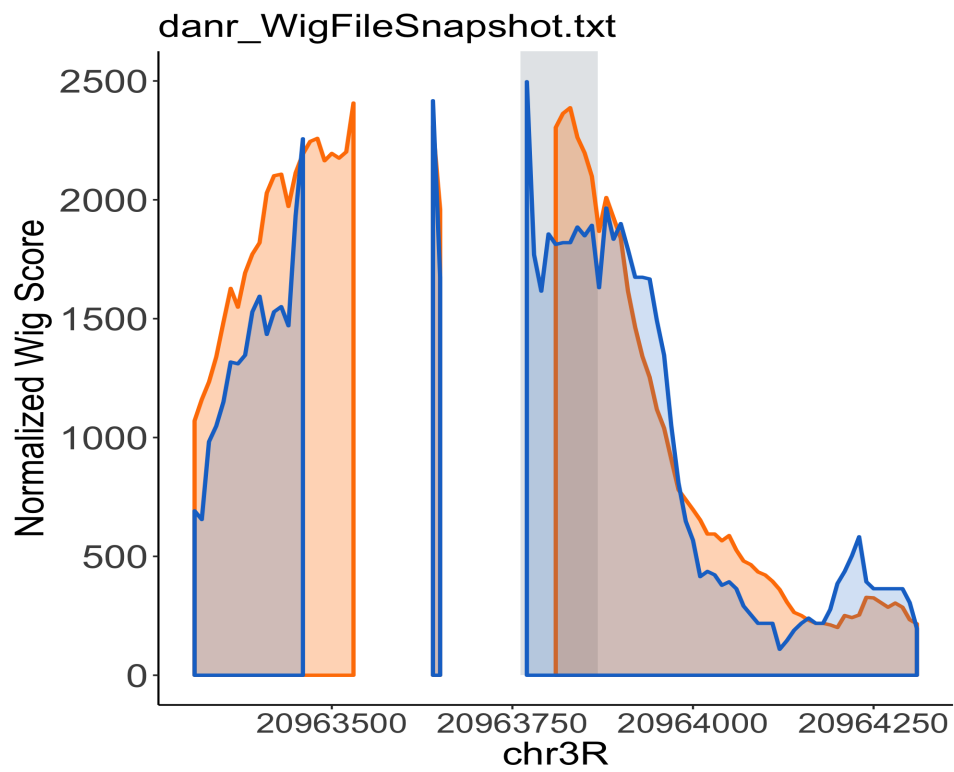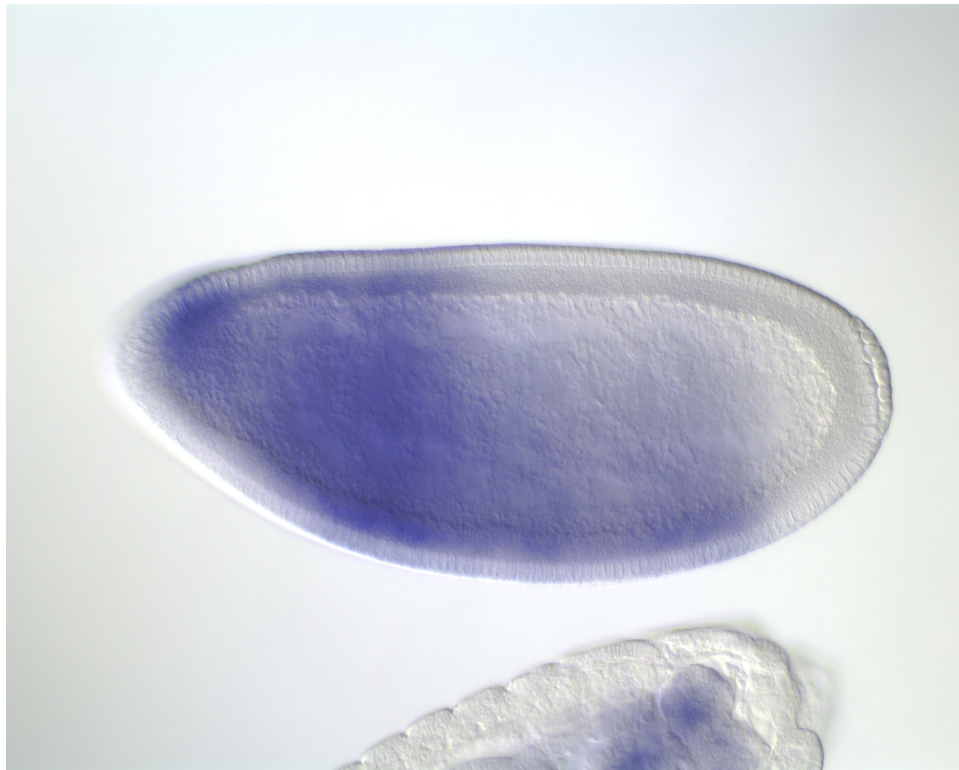

Location: Mostly Ant Type: Promoter ZScore: 0.543345255 PValue: 0.586892111

Supplement: S3 File — Reports consist of in situ hybridization images, ATAC-seq traces, and calculated p-value and Z Score for each region used in the final analysis. (ZIP) [file pgen.1007367.s015.zip › S3_File/danr_Report.pdf]

dap

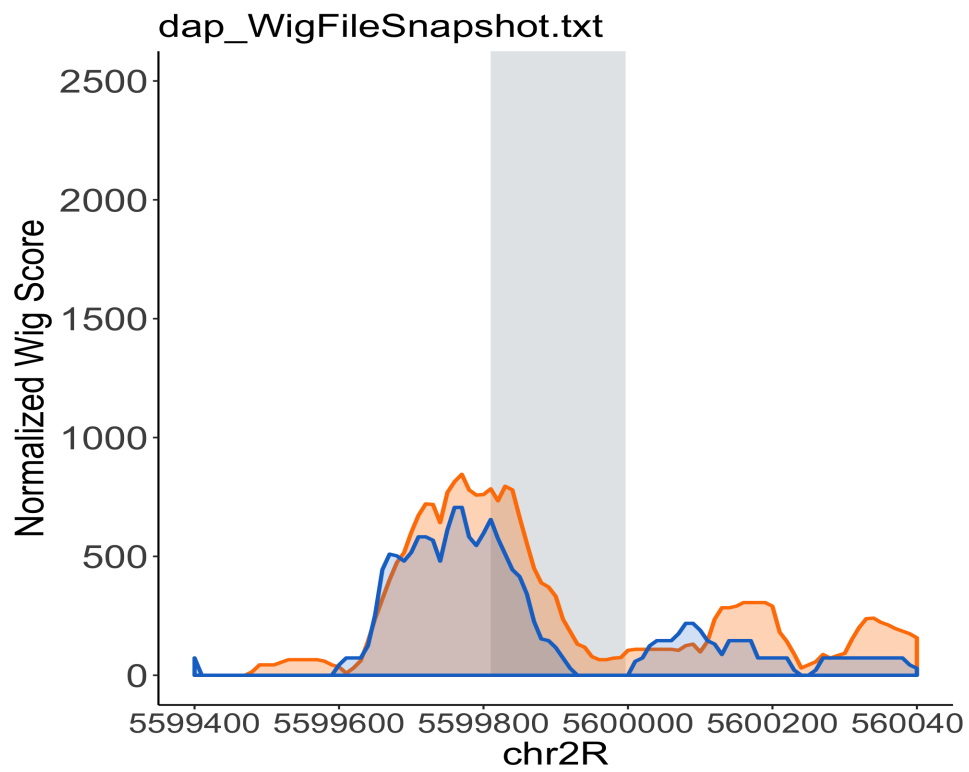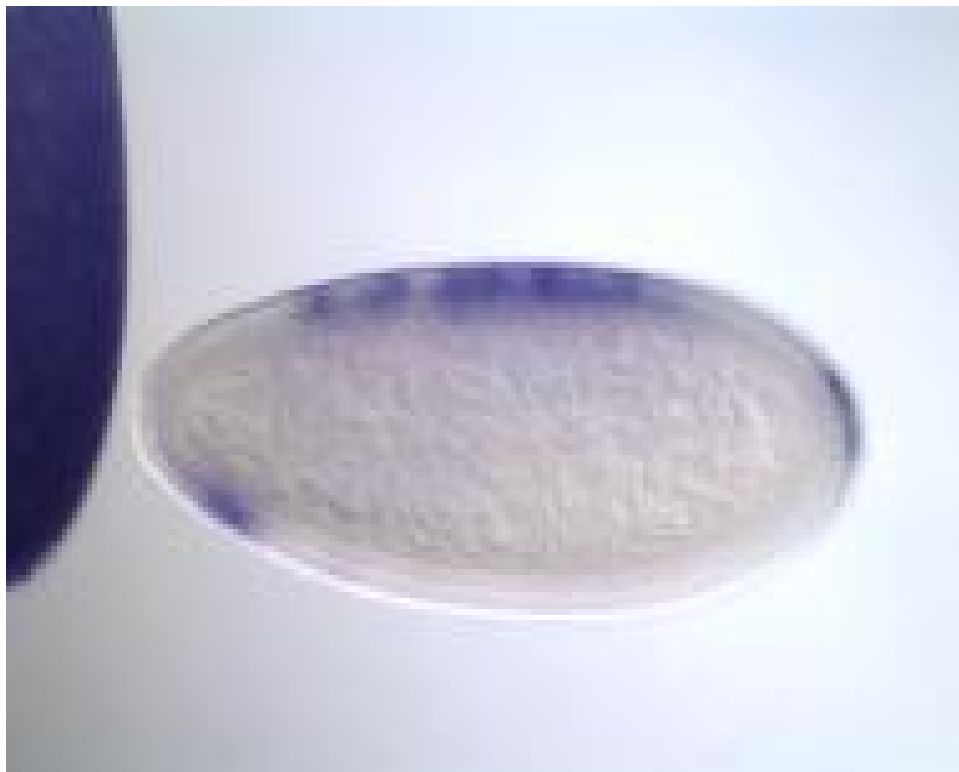

Location: Dorsal Type: Promoter ZScore: 1.343953005 PValue: 0.178963584

Supplement: S3 File — Reports consist of in situ hybridization images, ATAC-seq traces, and calculated p-value and Z Score for each region used in the final analysis. (ZIP) [file pgen.1007367.s015.zip › S3_File/dap_Report.pdf]

dib

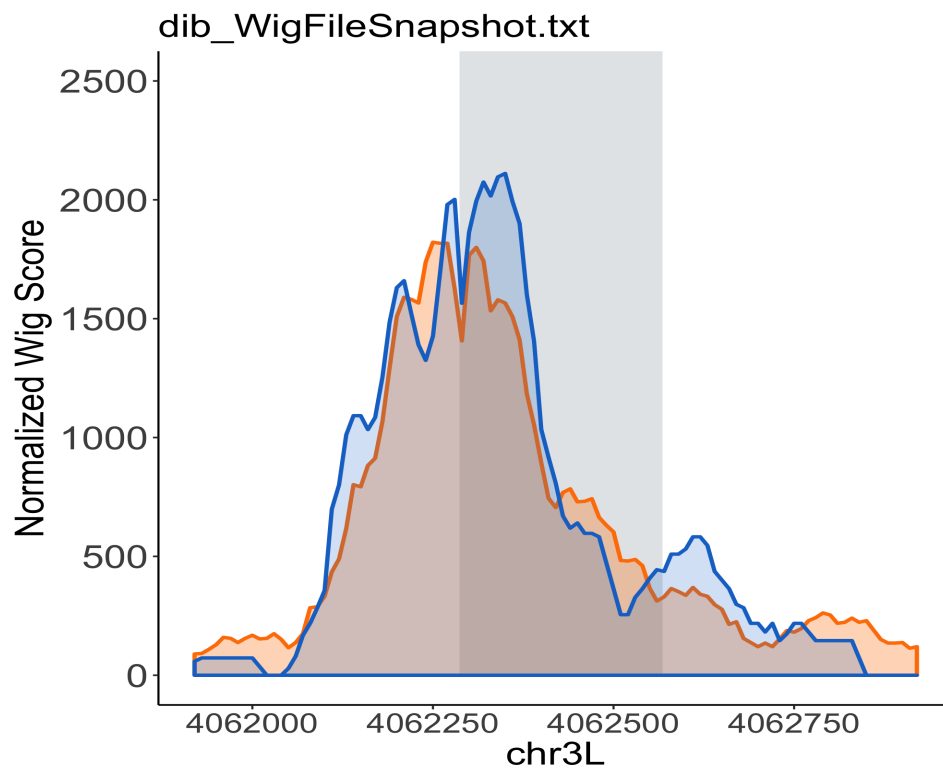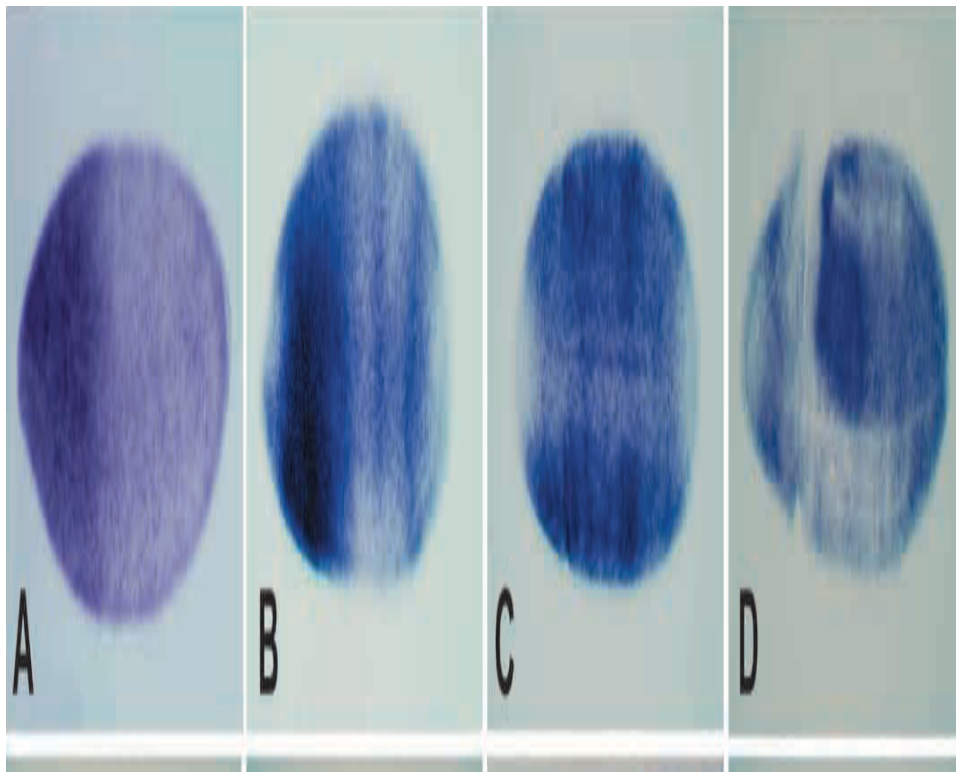

Location: Mostly Ant Type: Promoter ZScore: -0.287740525 PValue: 0.773545367

Supplement: S3 File — Reports consist of in situ hybridization images, ATAC-seq traces, and calculated p-value and Z Score for each region used in the final analysis. (ZIP) [file pgen.1007367.s015.zip › S3_File/dib_Report.pdf]

## Doc1

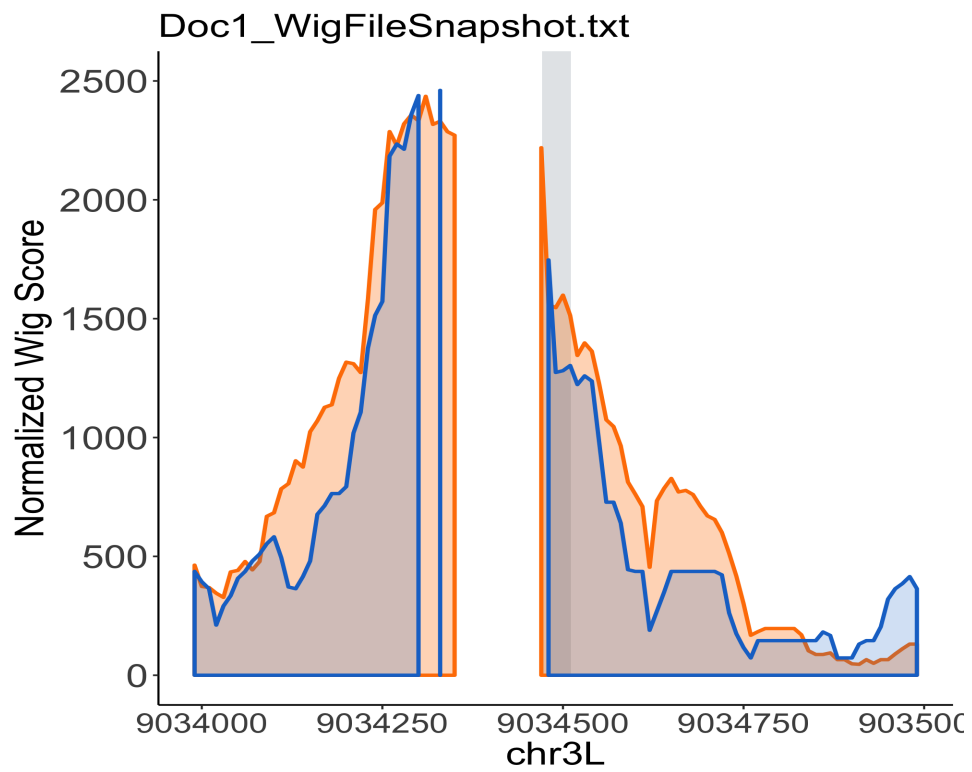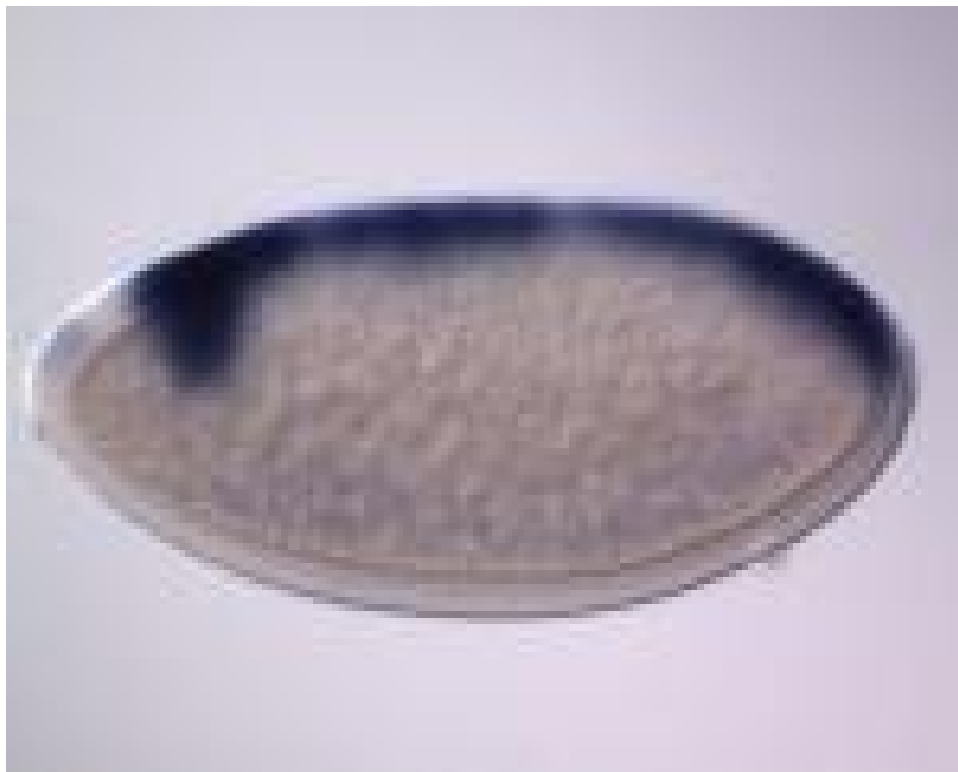

Location: Dorsal Type: Promoter ZScore: 0.02962581 PValue: 0.976365481

Supplement: S3 File — Reports consist of in situ hybridization images, ATAC-seq traces, and calculated p-value and Z Score for each region used in the final analysis. (ZIP) [file pgen.1007367.s015.zip › S3_File/Doc1_Report.pdf]

## Doc3

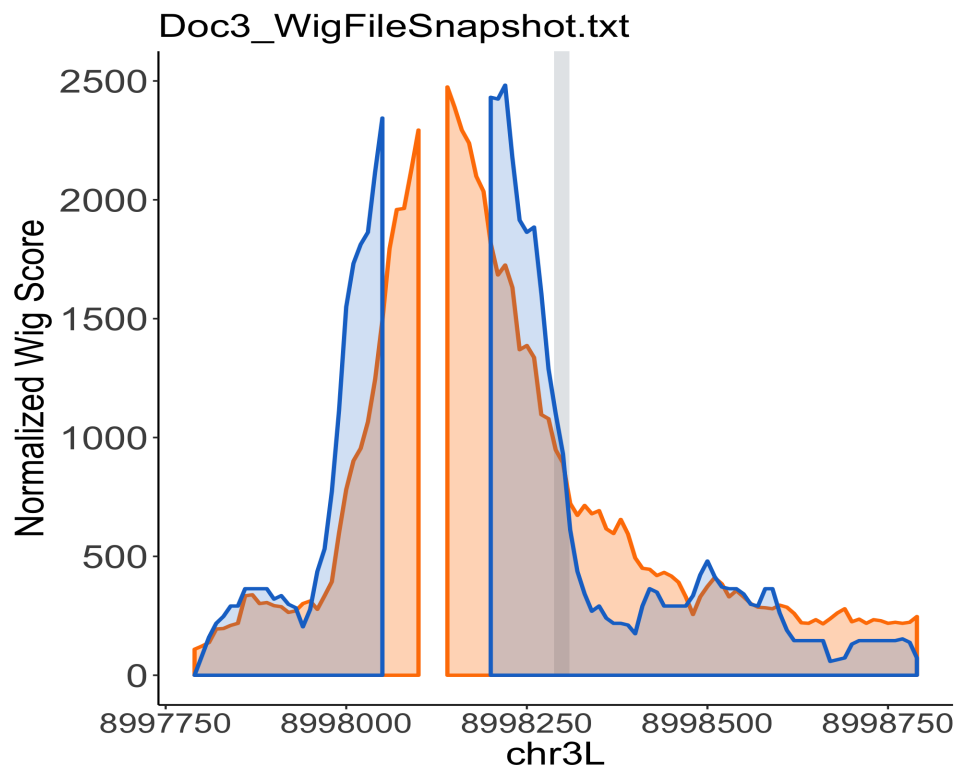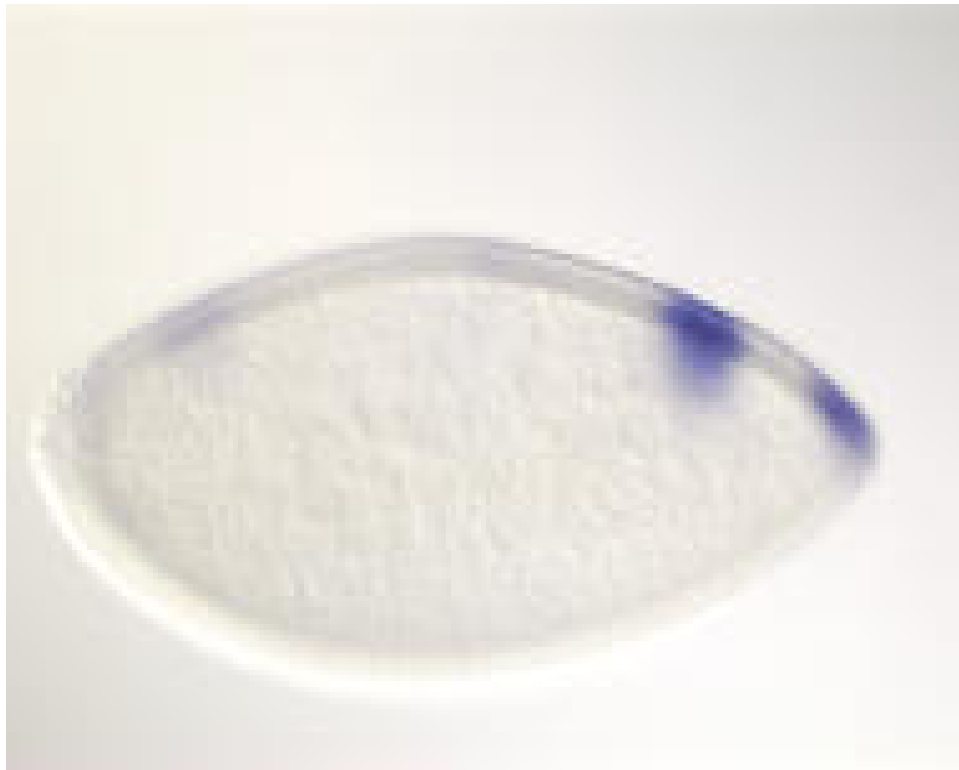

Location: Dorsal Type: Promoter ZScore: -0.338799273 PValue: 0.734760949

Supplement: S3 File — Reports consist of in situ hybridization images, ATAC-seq traces, and calculated p-value and Z Score for each region used in the final analysis. (ZIP) [file pgen.1007367.s015.zip › S3_File/Doc3_Report.pdf]

dpp\_Huang

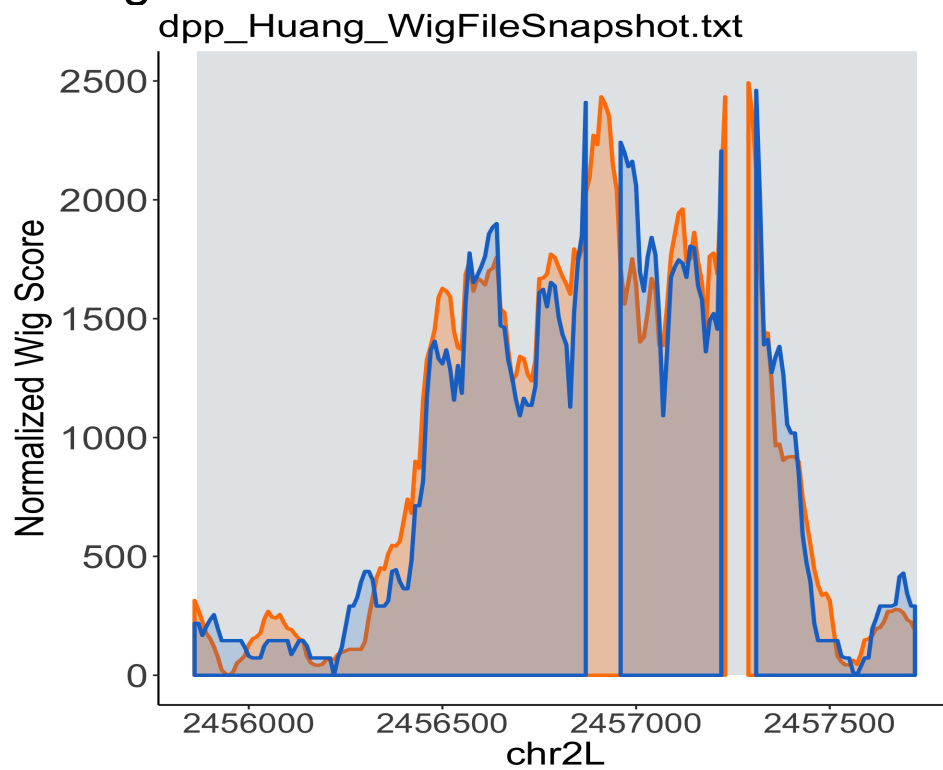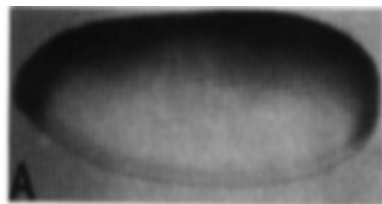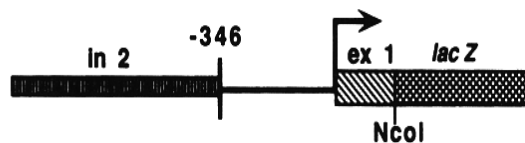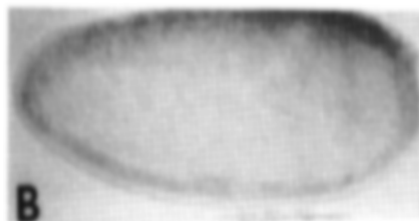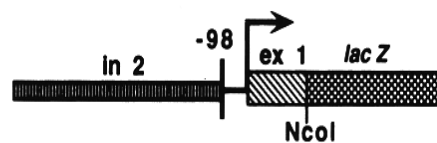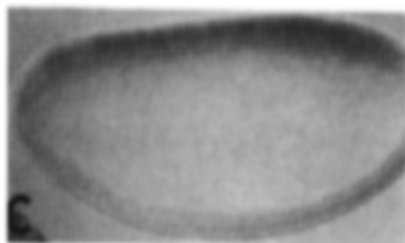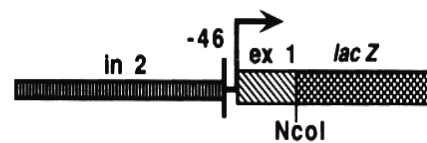

Location: Dorsal Type: Enhancer ZScore: -0.135796451 PValue: 0.891982197

Supplement: S3 File — Reports consist of in situ hybridization images, ATAC-seq traces, and calculated p-value and Z Score for each region used in the final analysis. (ZIP) [file pgen.1007367.s015.zip › S3_File/dpp_Huang_Report.pdf]

Dscam\_Ozdemir

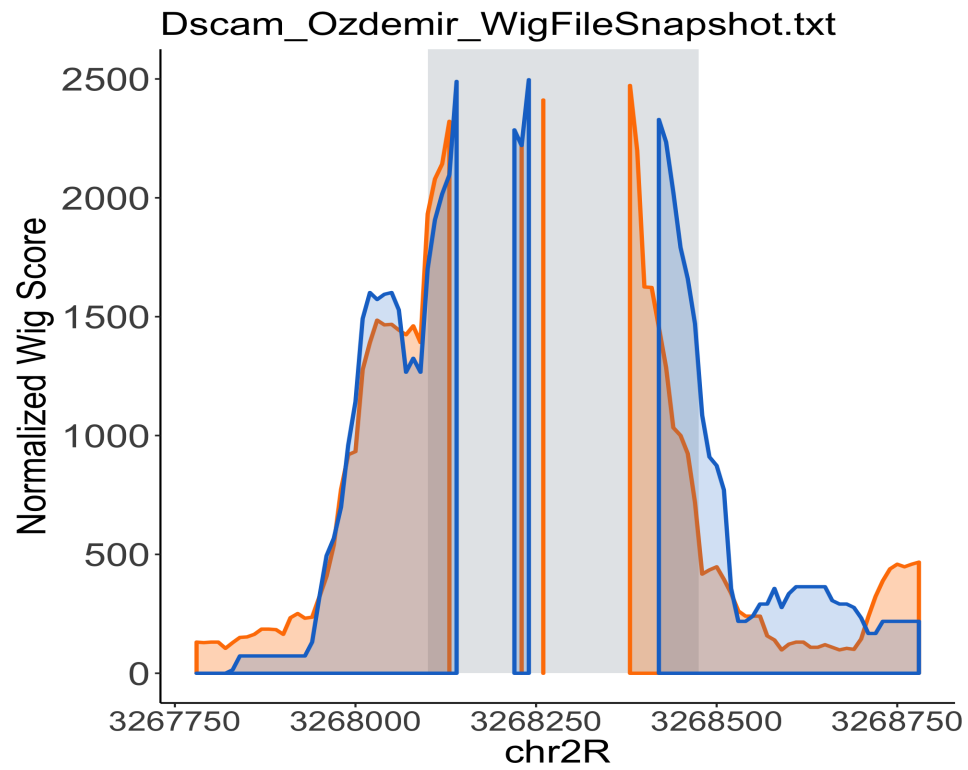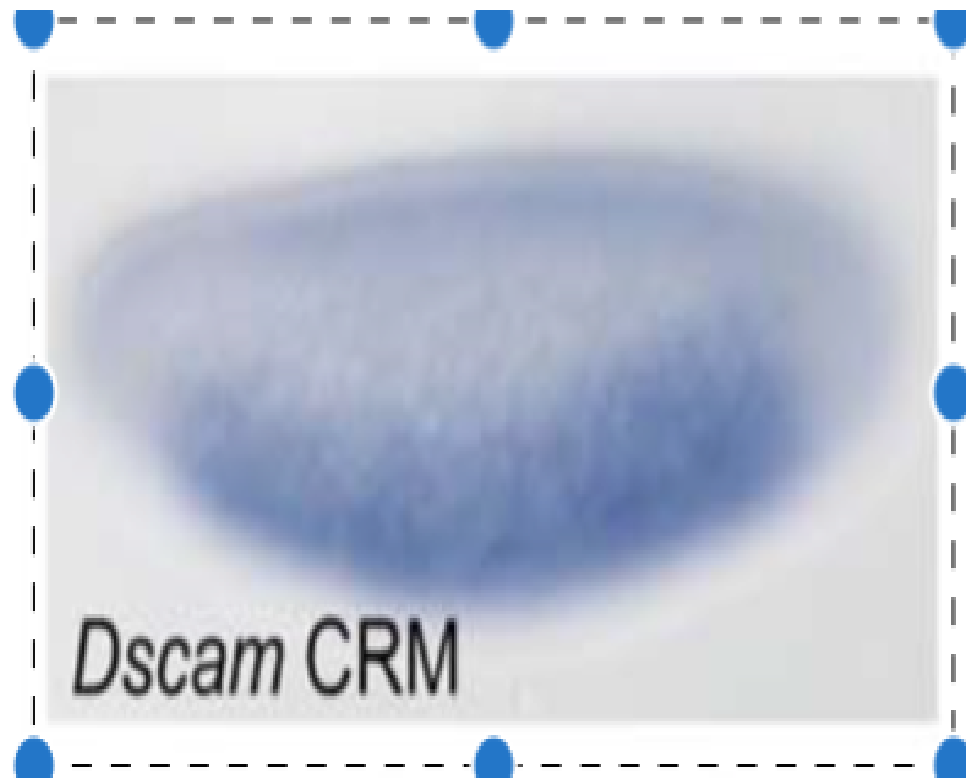

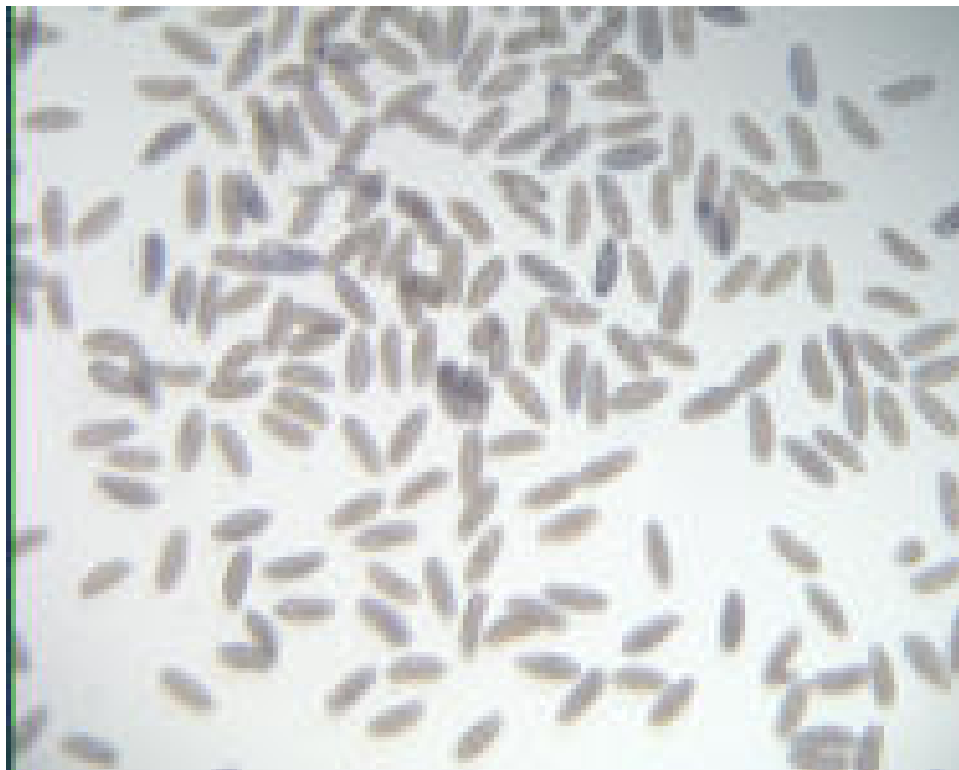

Location: Ventral Type: Enhancer ZScore: -0.268143297 PValue: 0.788589019

Supplement: S3 File — Reports consist of in situ hybridization images, ATAC-seq traces, and calculated p-value and Z Score for each region used in the final analysis. (ZIP) [file pgen.1007367.s015.zip › S3_File/Dscam_Ozdemir_Report.pdf]

# Dtg\_Hodar

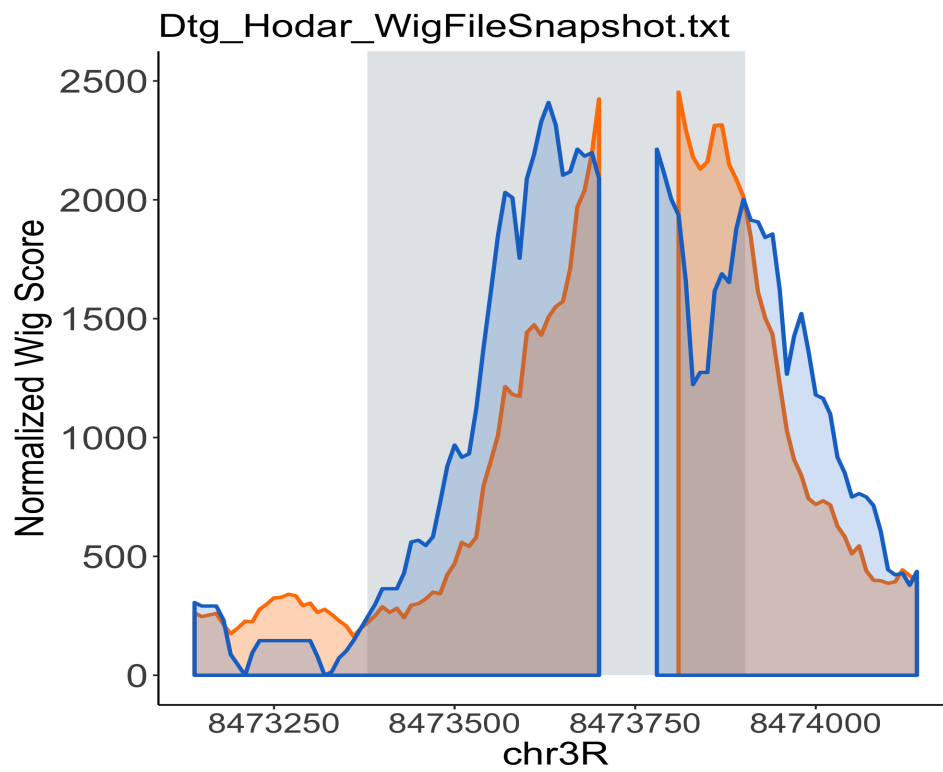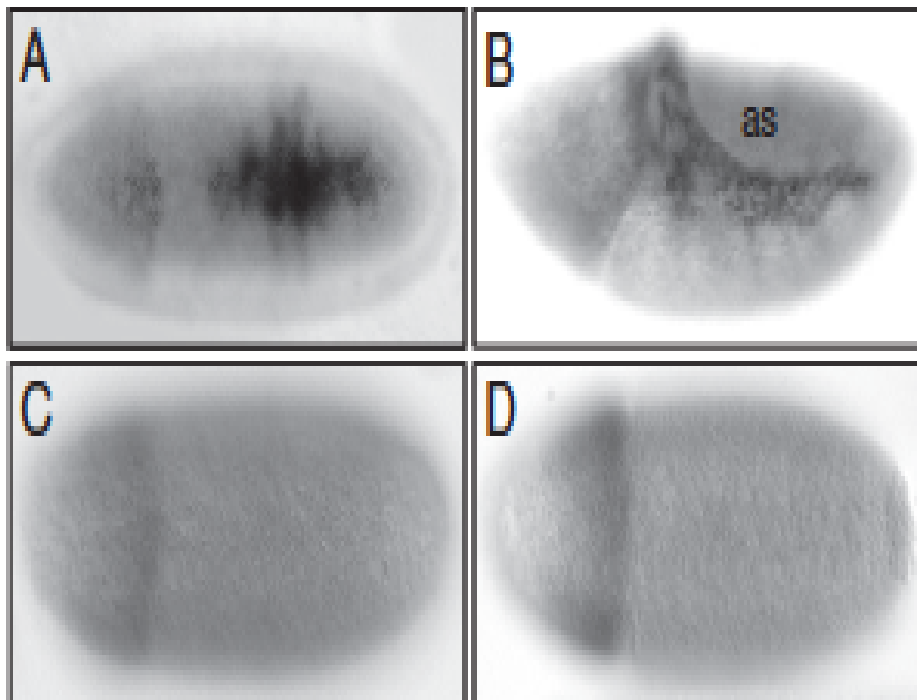

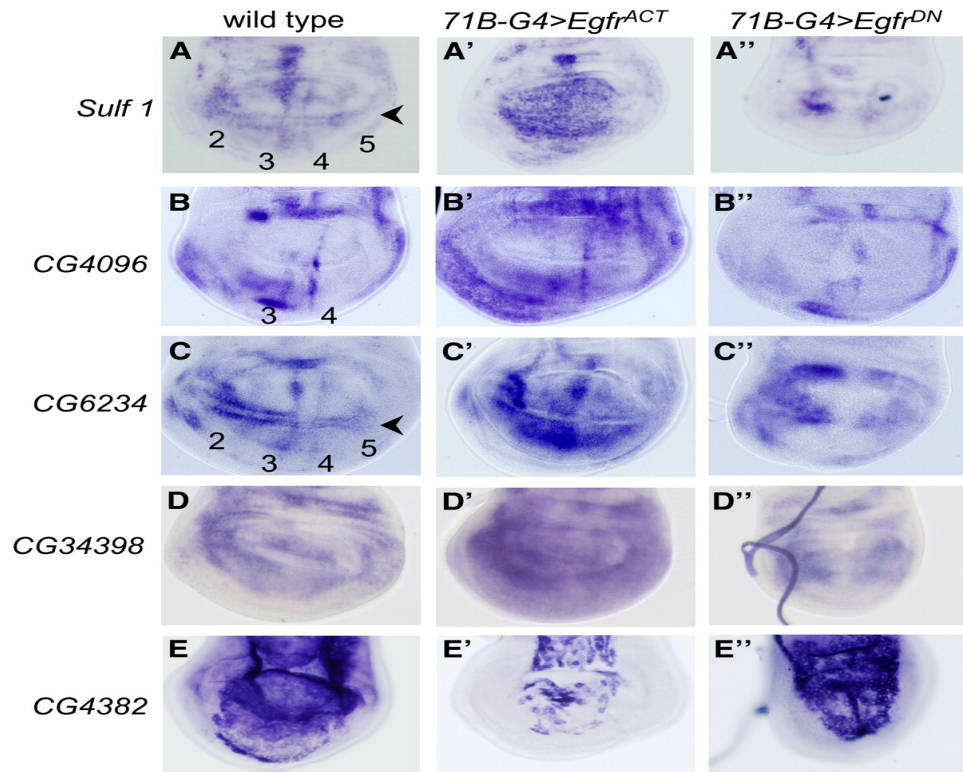

Location: Dorsal Type: Enhancer ZScore: -0.077925676 PValue: 0.937887175

Supplement: S3 File — Reports consist of in situ hybridization images, ATAC-seq traces, and calculated p-value and Z Score for each region used in the final analysis. (ZIP) [file pgen.1007367.s015.zip › S3_File/Dtg_Hodar_Report.pdf]

edl

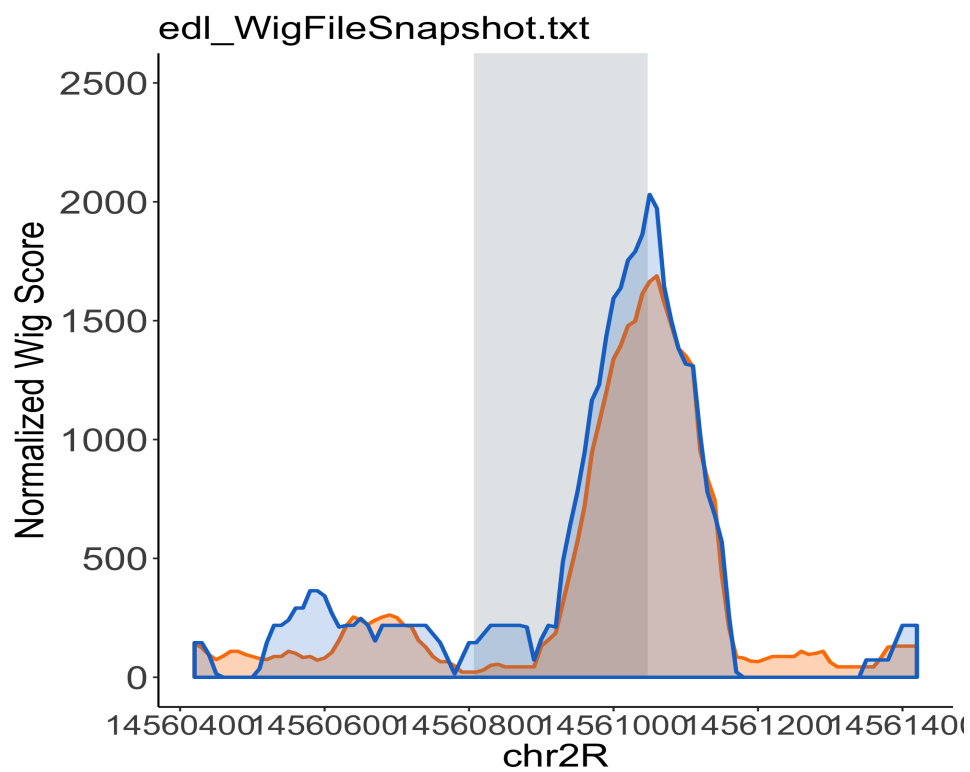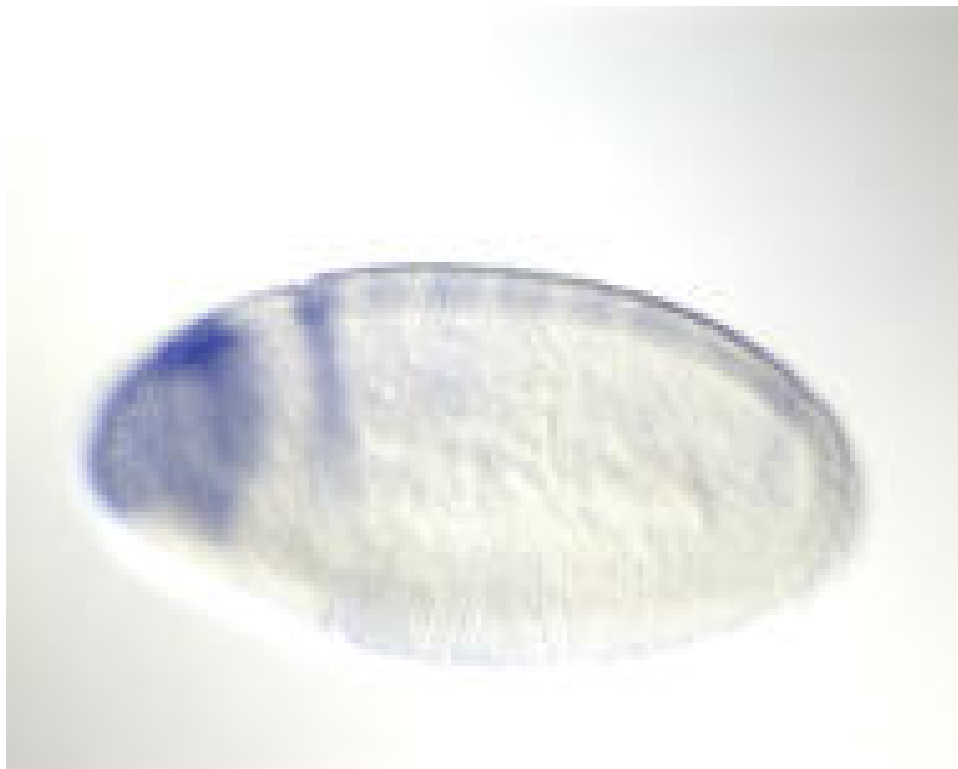

Location: Dorsal Type: Promoter ZScore: -0.678280432 PValue: 0.497593903

Supplement: S3 File — Reports consist of in situ hybridization images, ATAC-seq traces, and calculated p-value and Z Score for each region used in the final analysis. (ZIP) [file pgen.1007367.s015.zip › S3_File/edl_Report.pdf]

egr

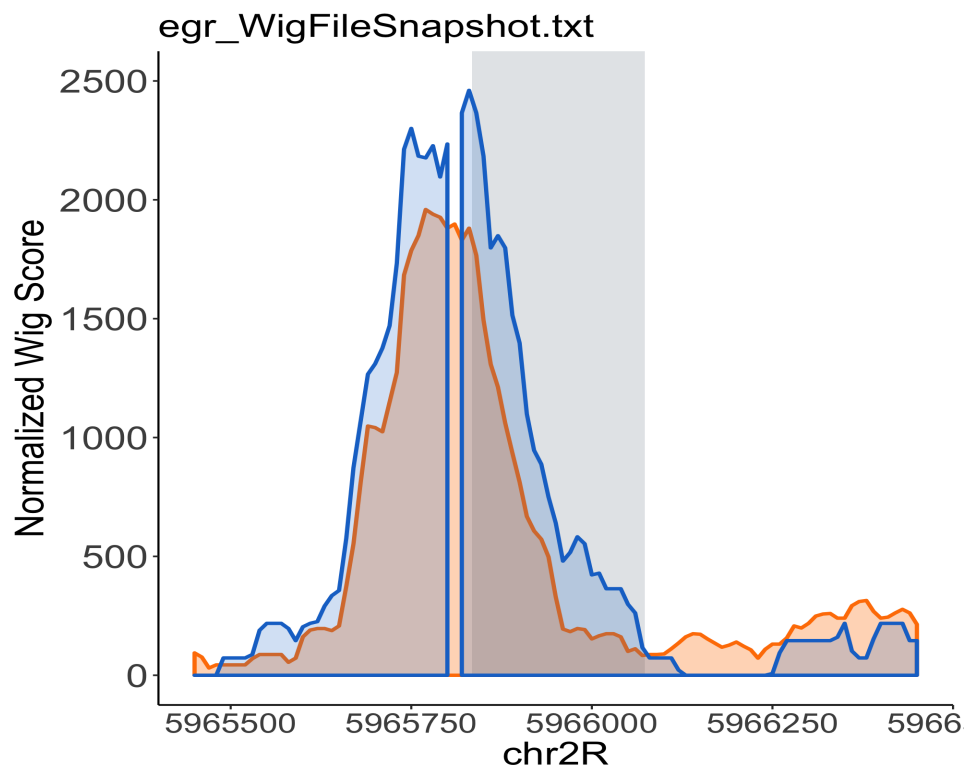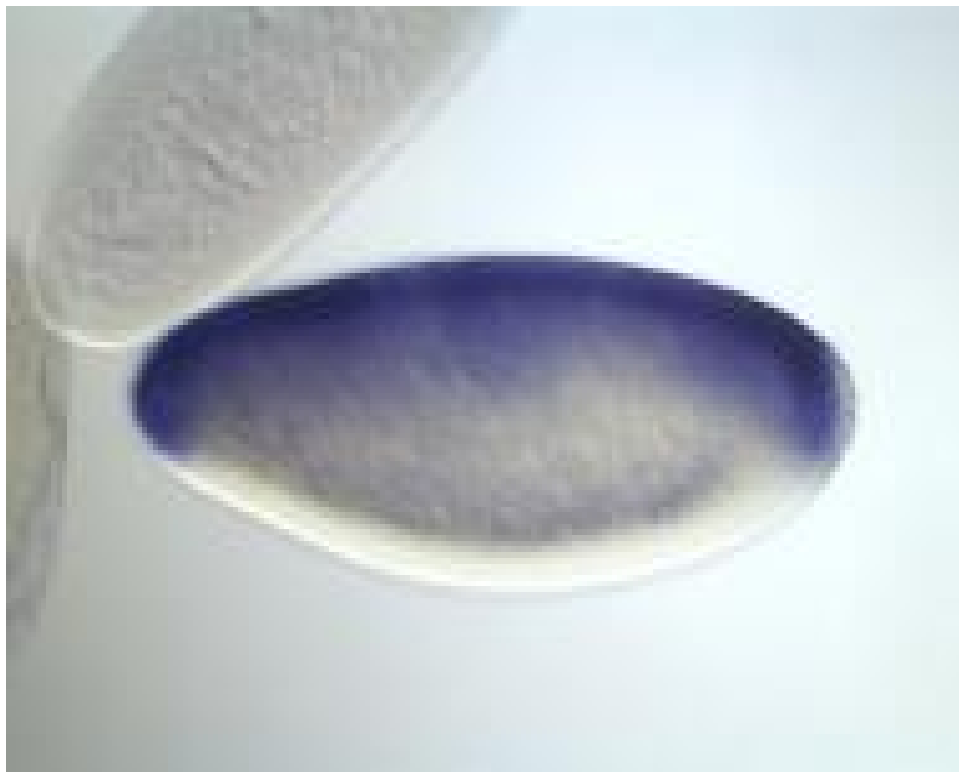

Location: Dorsal Type: Promoter ZScore: -1.139037003 PValue: 0.254687721

Supplement: S3 File — Reports consist of in situ hybridization images, ATAC-seq traces, and calculated p-value and Z Score for each region used in the final analysis. (ZIP) [file pgen.1007367.s015.zip › S3_File/egr_Report.pdf]

ems\_distal\_3'

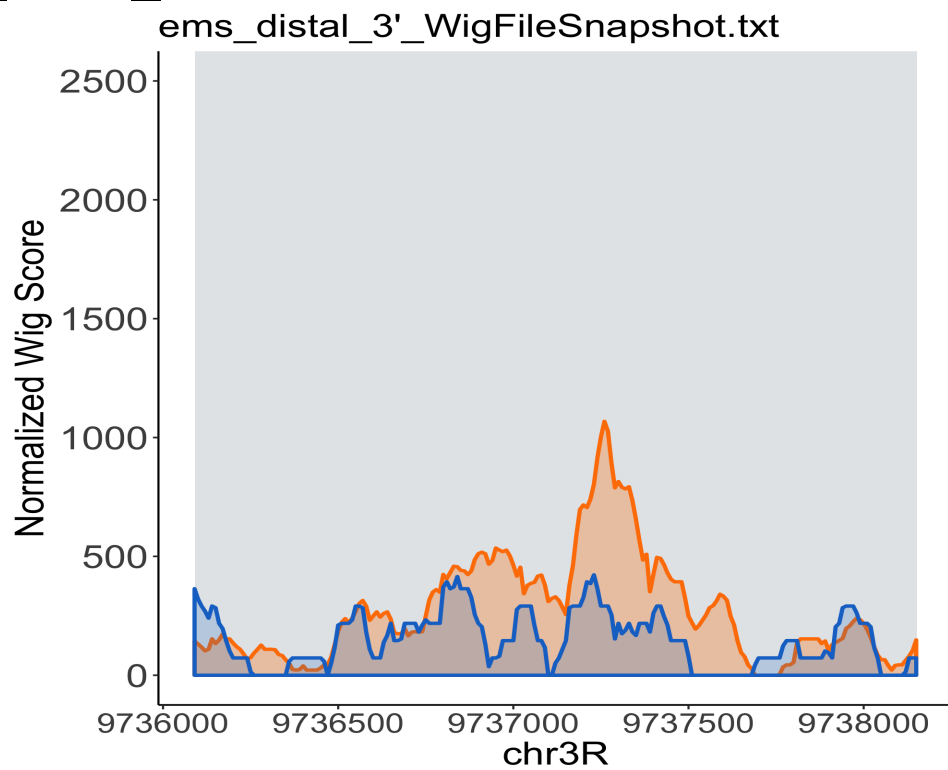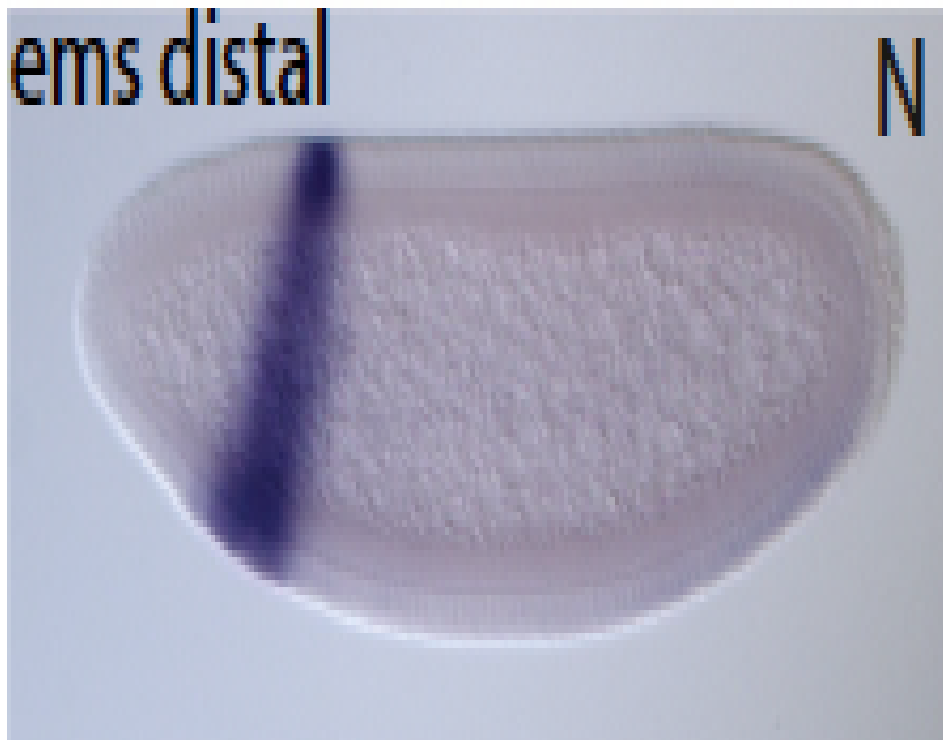

Location: Anterior Type: Enhancer ZScore: 1.309354011 PValue: 0.190414462

Supplement: S3 File — Reports consist of in situ hybridization images, ATAC-seq traces, and calculated p-value and Z Score for each region used in the final analysis. (ZIP) [file pgen.1007367.s015.zip › S3_File/ems_distal_3'_Report.pdf]

ems\_elementIV\_70

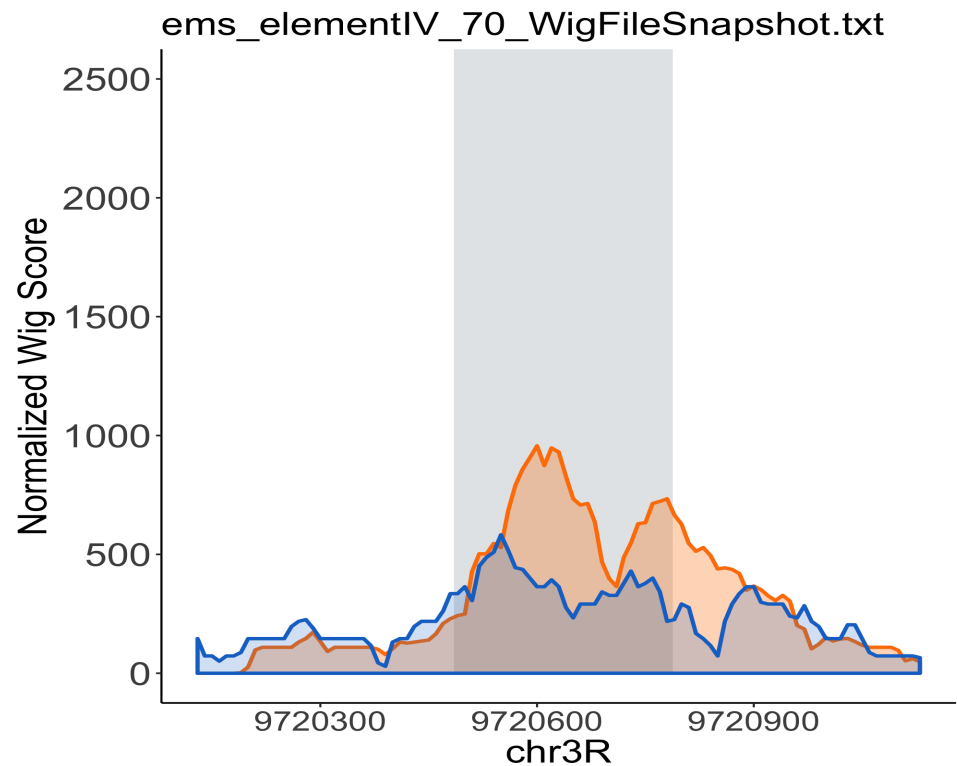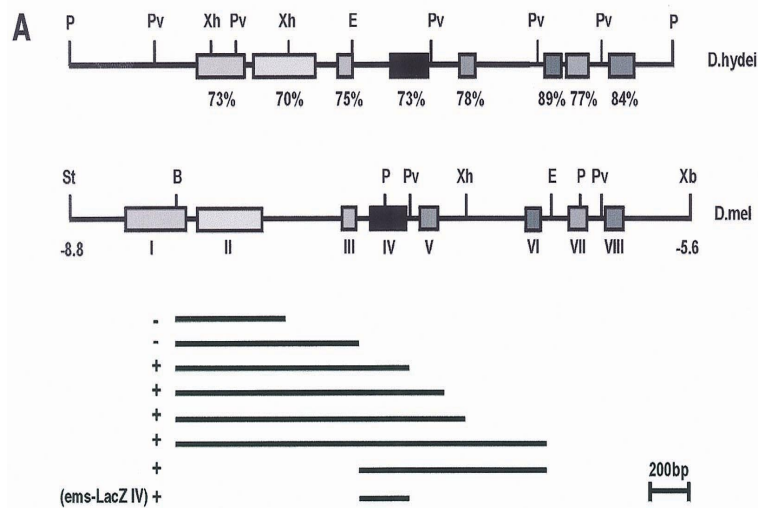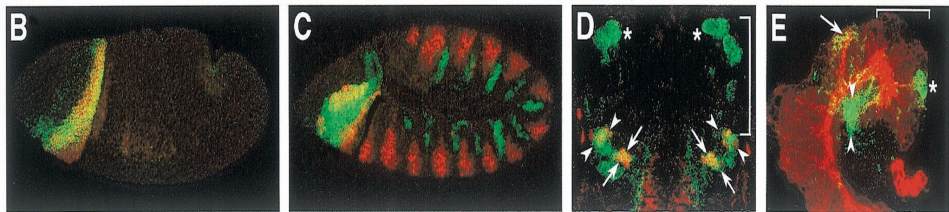

Location: Anterior Type: Enhancer ZScore: 1.130915264 PValue: 0.258090758

Supplement: S3 File — Reports consist of in situ hybridization images, ATAC-seq traces, and calculated p-value and Z Score for each region used in the final analysis. (ZIP) [file pgen.1007367.s015.zip › S3_File/ems_elementIV_70_Report.pdf]

ems

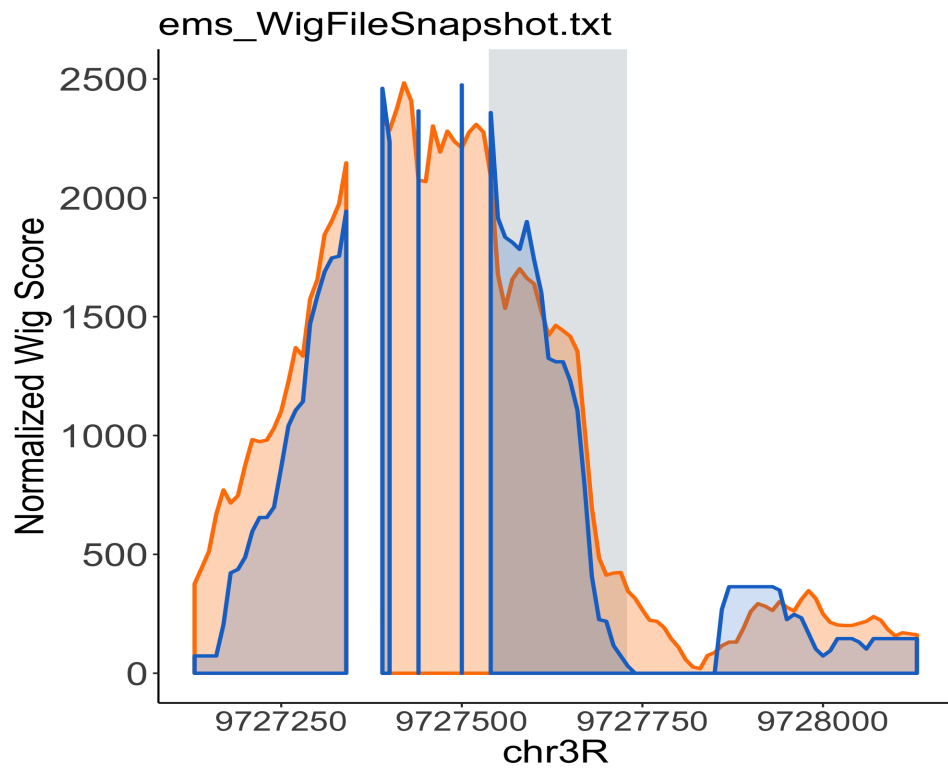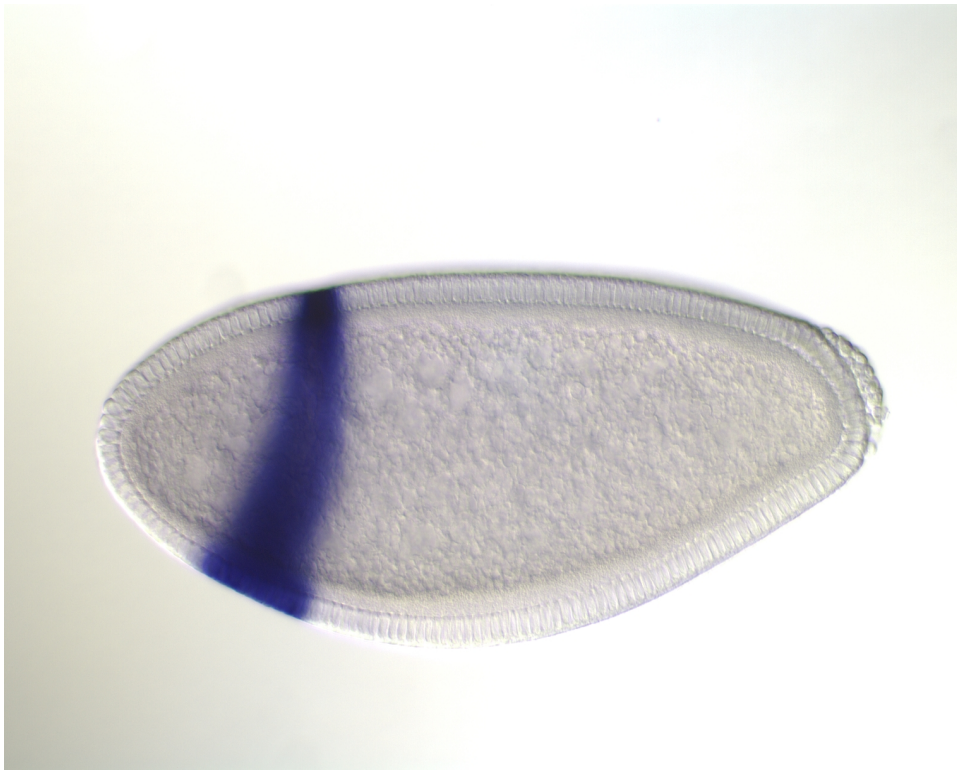

Location: Anterior Type: Promoter ZScore: 0.007209644 PValue: 0.994247586

Supplement: S3 File — Reports consist of in situ hybridization images, ATAC-seq traces, and calculated p-value and Z Score for each region used in the final analysis. (ZIP) [file pgen.1007367.s015.zip › S3_File/ems_Report.pdf]

erm

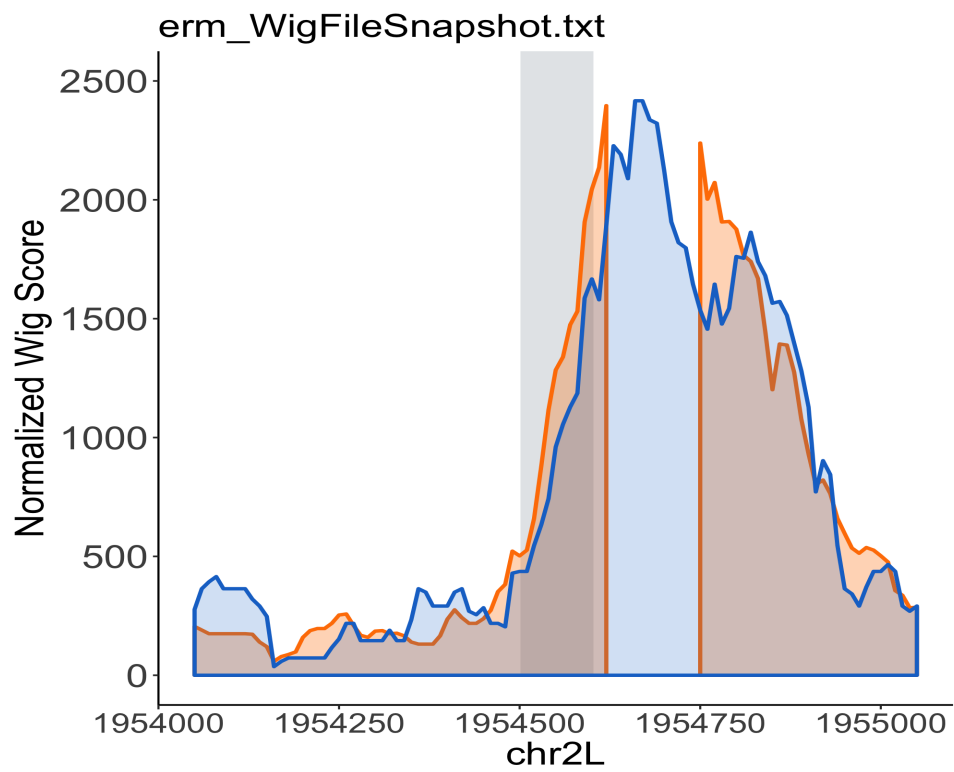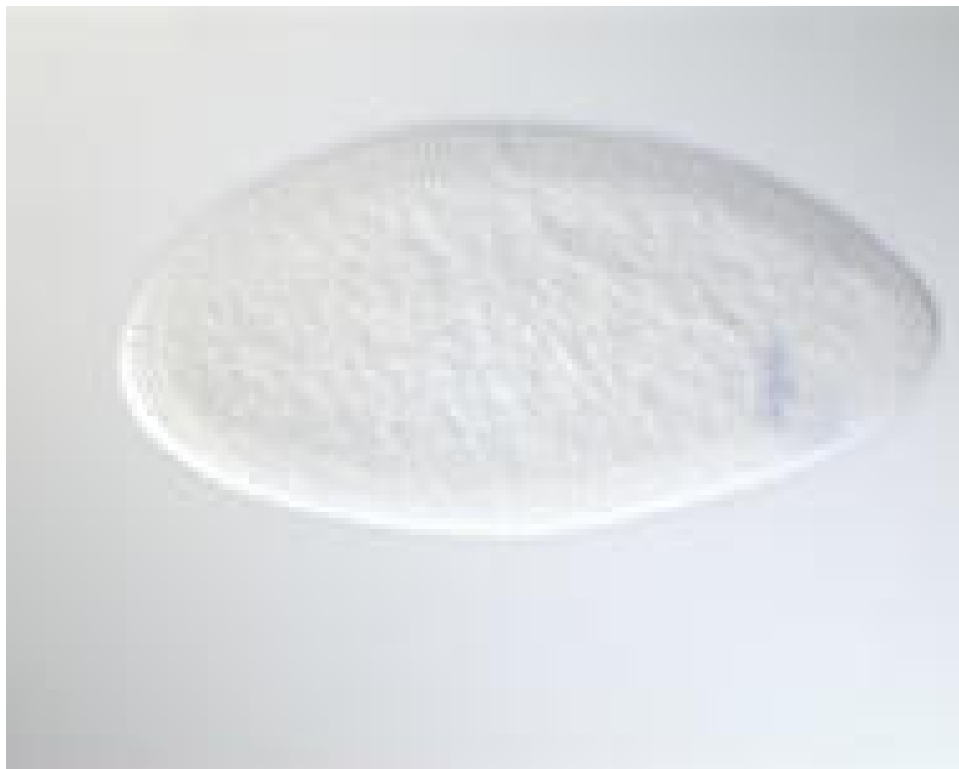

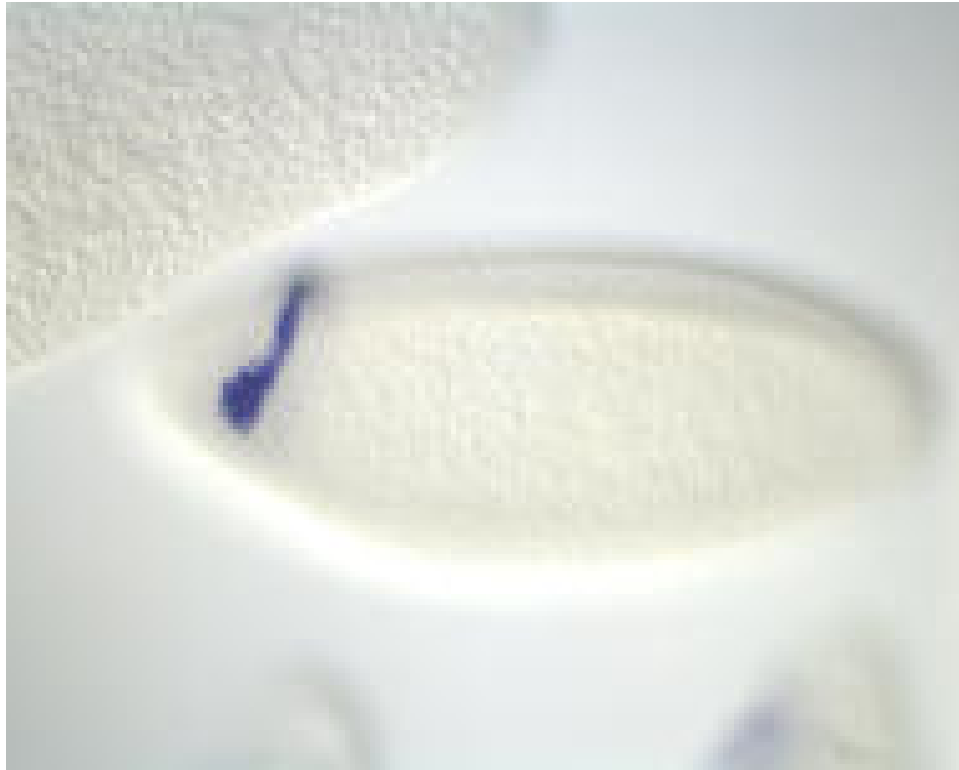

Location: Anterior Type: Promoter ZScore: 0.515072013 PValue: 0.606502699

Supplement: S3 File — Reports consist of in situ hybridization images, ATAC-seq traces, and calculated p-value and Z Score for each region used in the final analysis. (ZIP) [file pgen.1007367.s015.zip › S3_File/erm_Report.pdf]

esg

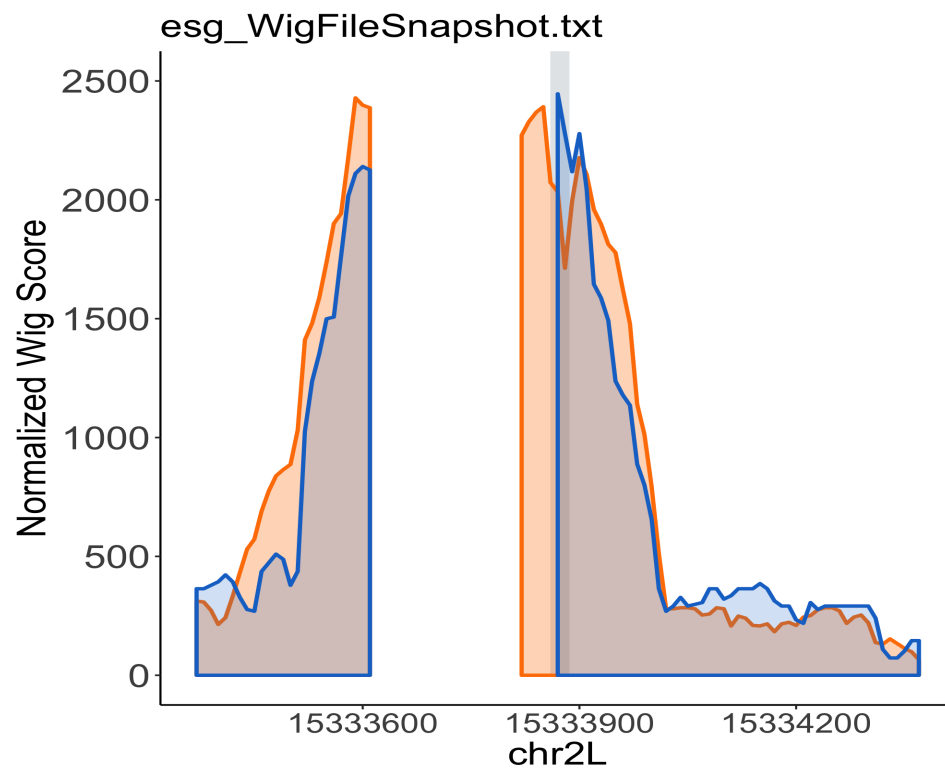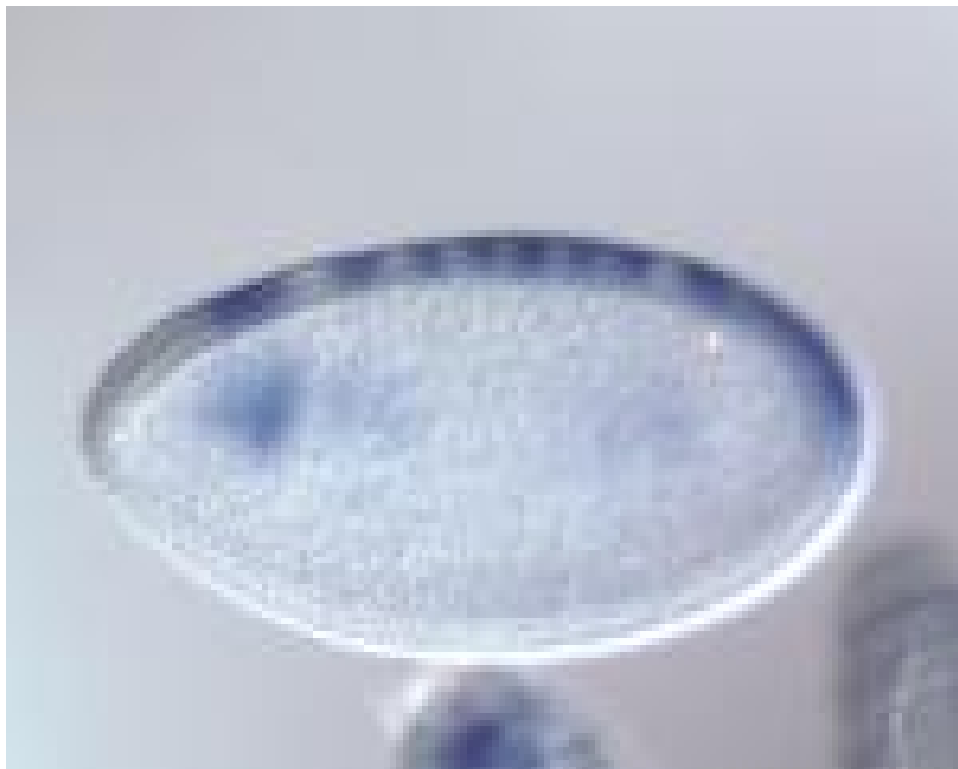

Location: Dorsal Type: Promoter ZScore: -0.595349173 PValue: 0.551610093

Supplement: S3 File — Reports consist of in situ hybridization images, ATAC-seq traces, and calculated p-value and Z Score for each region used in the final analysis. (ZIP) [file pgen.1007367.s015.zip › S3_File/esg_Report.pdf]

eve\_stripe2\_55

eve\_stripe2\_55\_WigFileSnapshot.txt

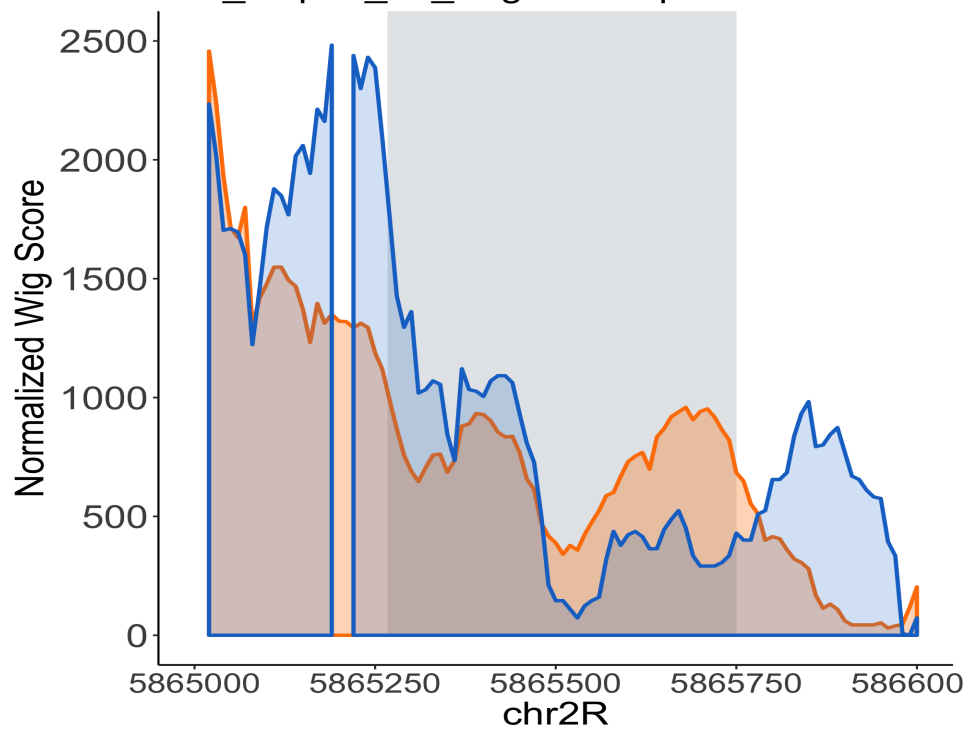

Location: Anterior Type: Enhancer ZScore: 0.176529016 PValue: 0.859878355

Supplement: S3 File — Reports consist of in situ hybridization images, ATAC-seq traces, and calculated p-value and Z Score for each region used in the final analysis. (ZIP) [file pgen.1007367.s015.zip › S3_File/eve_stripe2_55_Report.pdf]

ftz

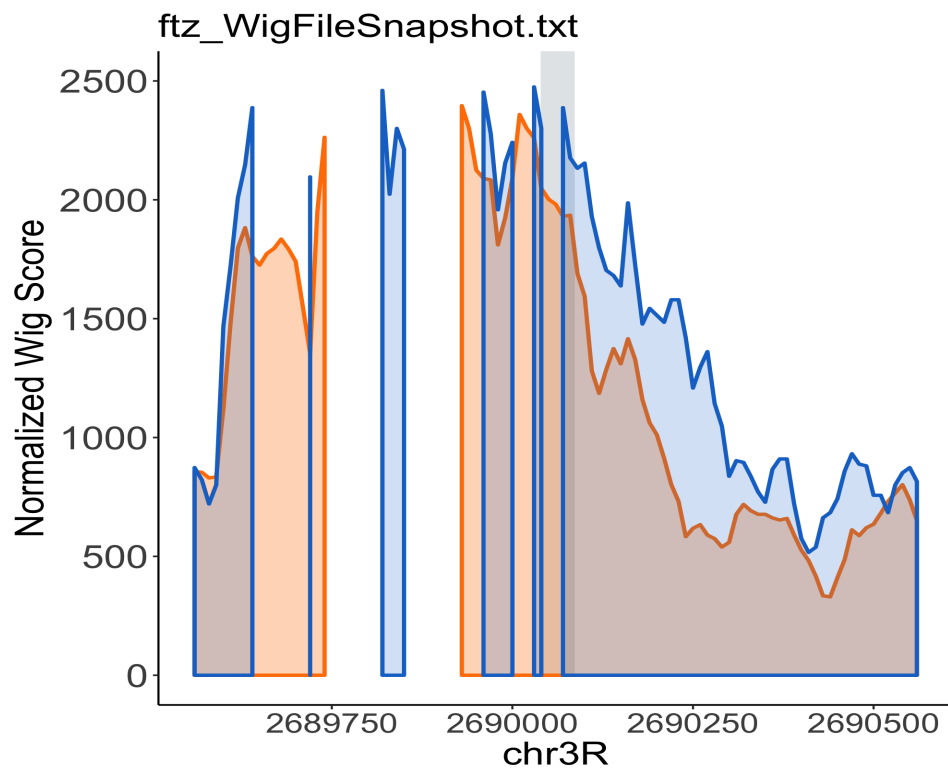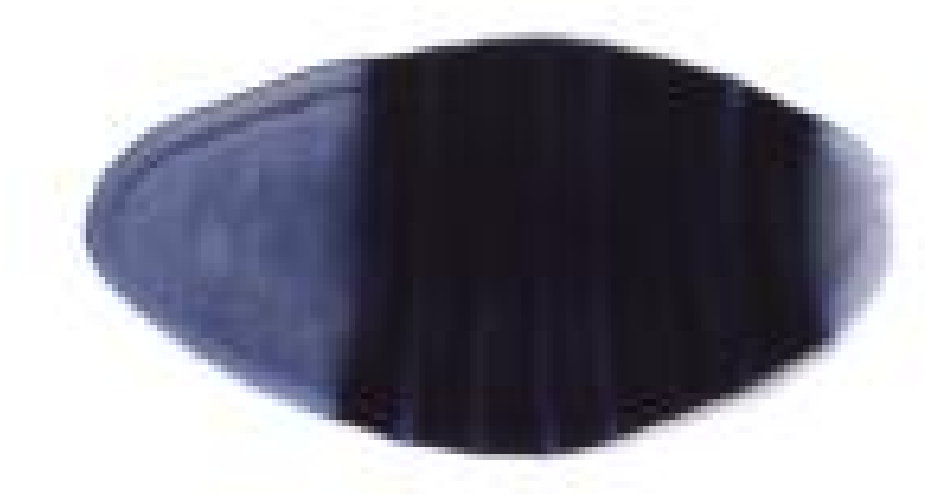

Location: Mostly Post Type: Promoter ZScore: 0.358097293 PValue: 0.720270505

Supplement: S3 File — Reports consist of in situ hybridization images, ATAC-seq traces, and calculated p-value and Z Score for each region used in the final analysis. (ZIP) [file pgen.1007367.s015.zip › S3_File/ftz_Report.pdf]

## G-salpa60A

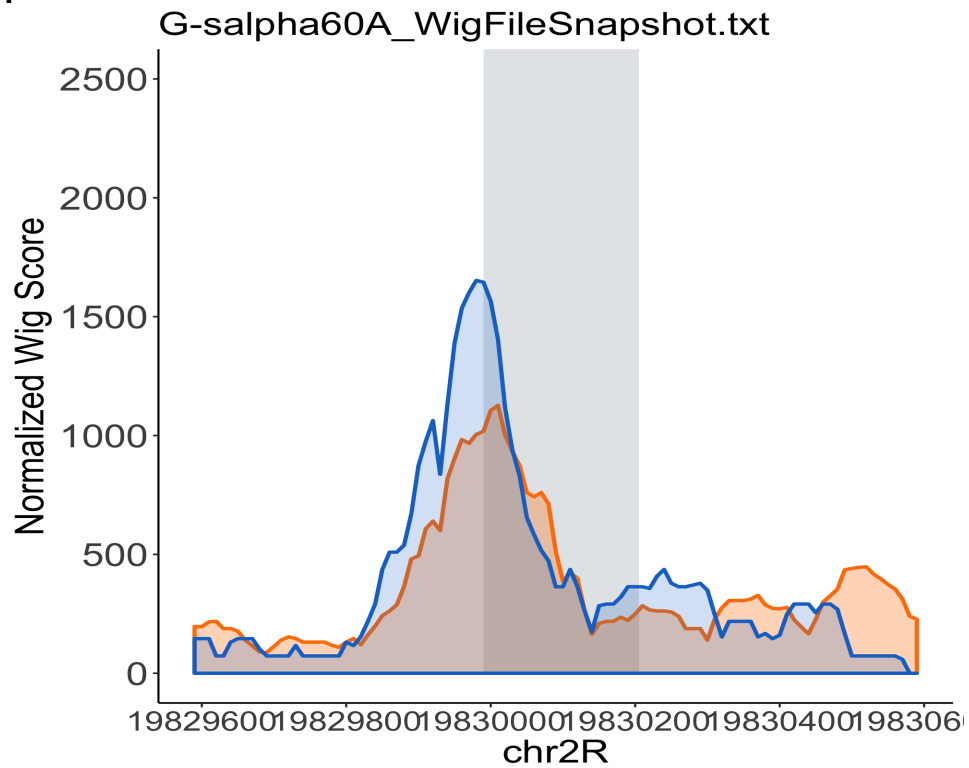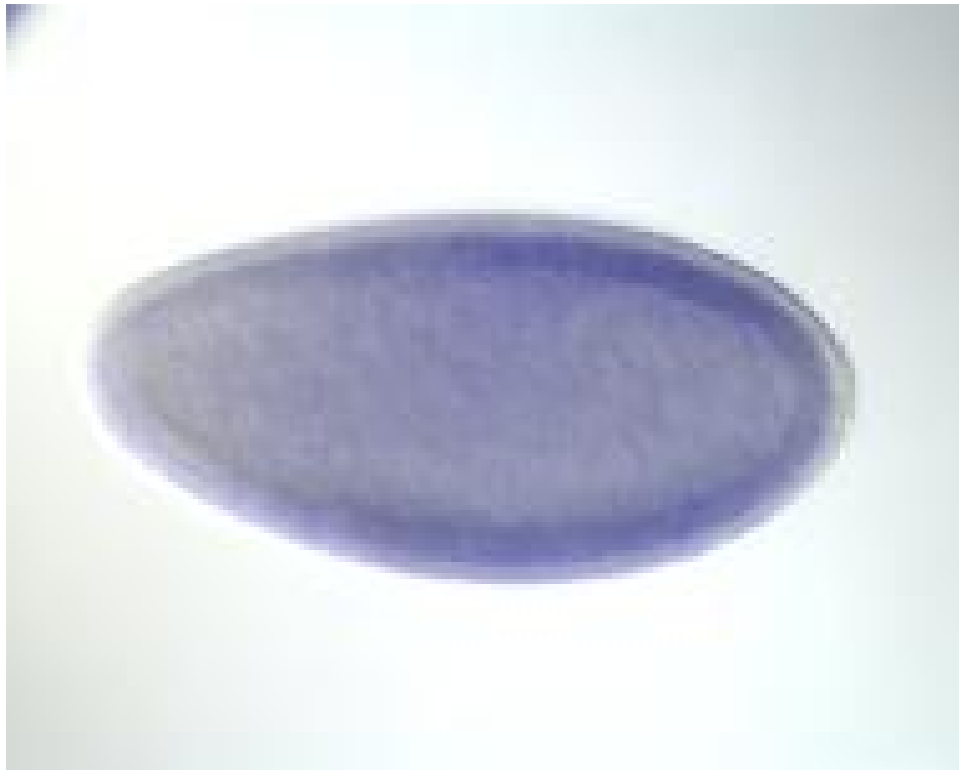

Location: Mostly Post Type: Promoter ZScore: 0.142031887 PValue: 0.887054819

Supplement: S3 File — Reports consist of in situ hybridization images, ATAC-seq traces, and calculated p-value and Z Score for each region used in the final analysis. (ZIP) [file pgen.1007367.s015.zip › S3_File/G-salpha60A_Report.pdf]

gcl

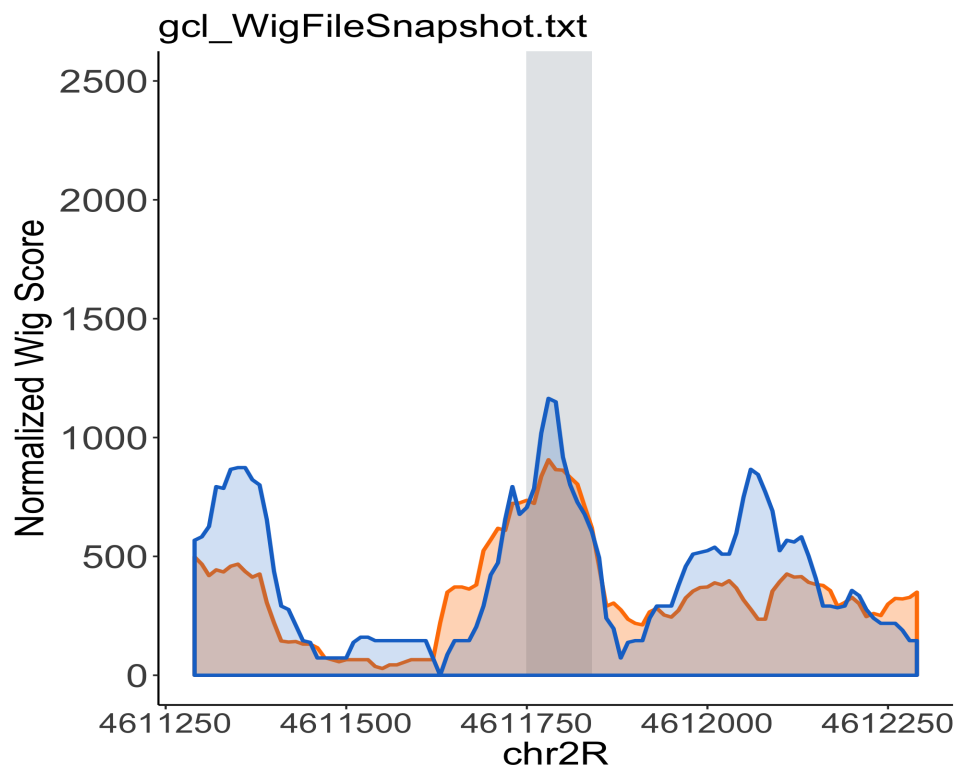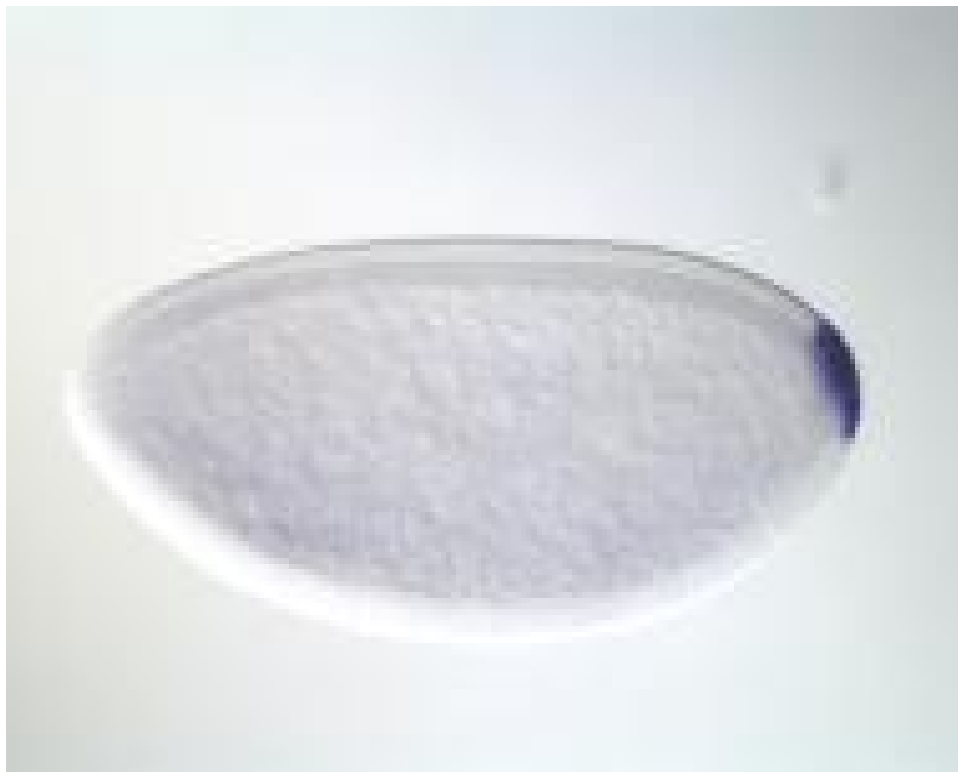

Location: Posterior Type: Promoter ZScore: 0.111572047 PValue: 0.911162737

Supplement: S3 File — Reports consist of in situ hybridization images, ATAC-seq traces, and calculated p-value and Z Score for each region used in the final analysis. (ZIP) [file pgen.1007367.s015.zip › S3_File/gcl_Report.pdf]

Gdi

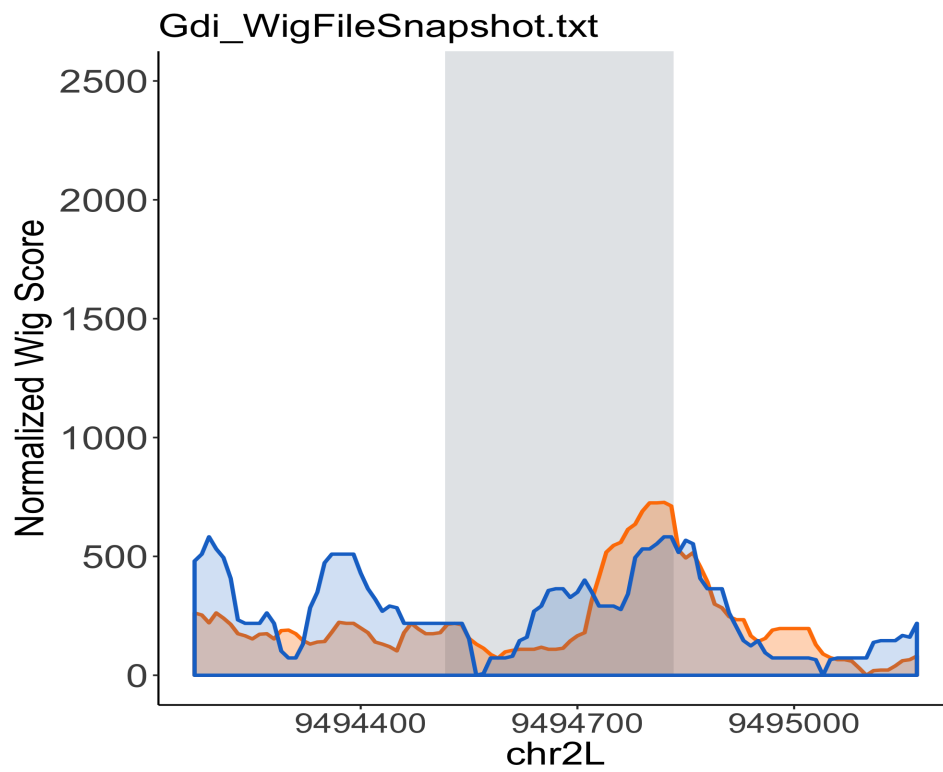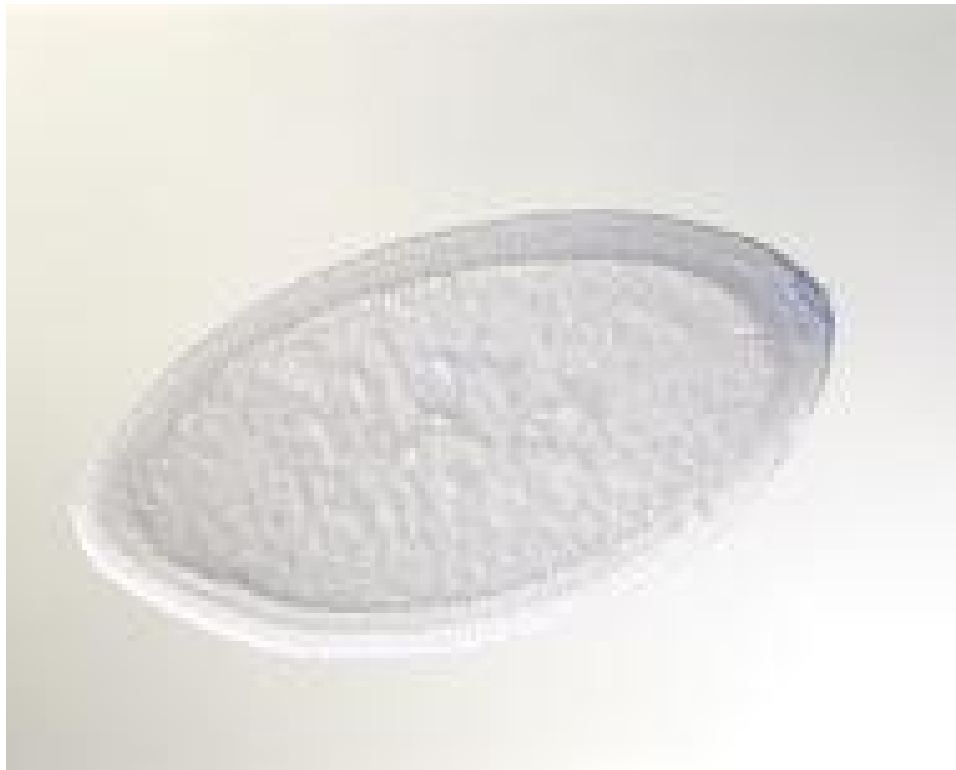

Location: Posterior Type: Promoter ZScore: -0.176601133 PValue: 0.859821704

Supplement: S3 File — Reports consist of in situ hybridization images, ATAC-seq traces, and calculated p-value and Z Score for each region used in the final analysis. (ZIP) [file pgen.1007367.s015.zip › S3_File/Gdi_Report.pdf]

# GEFmeso

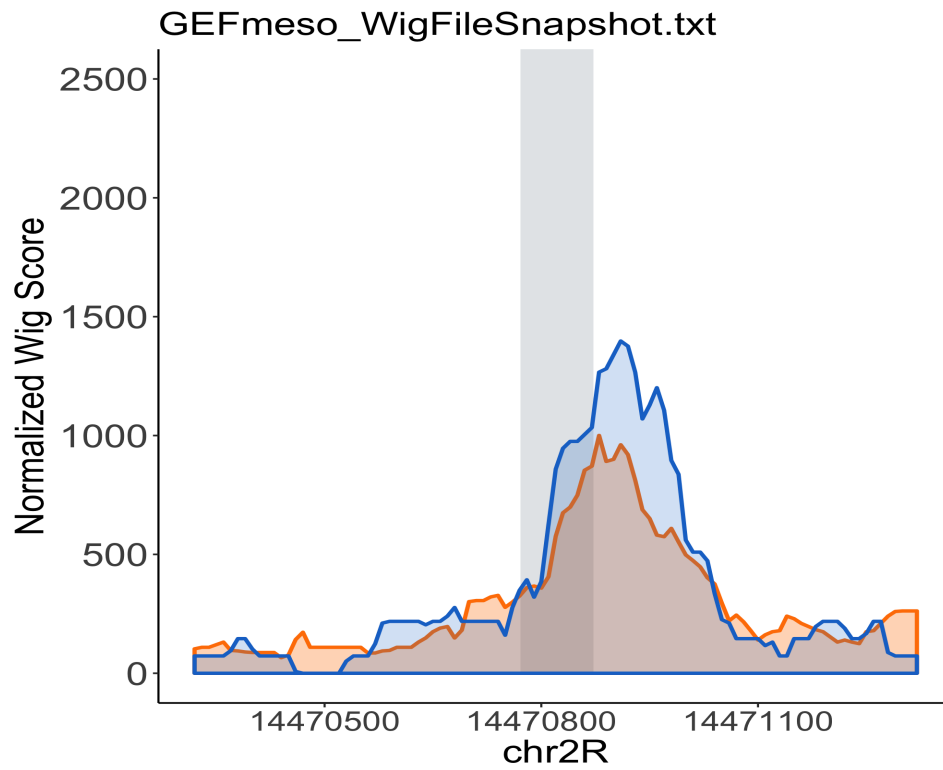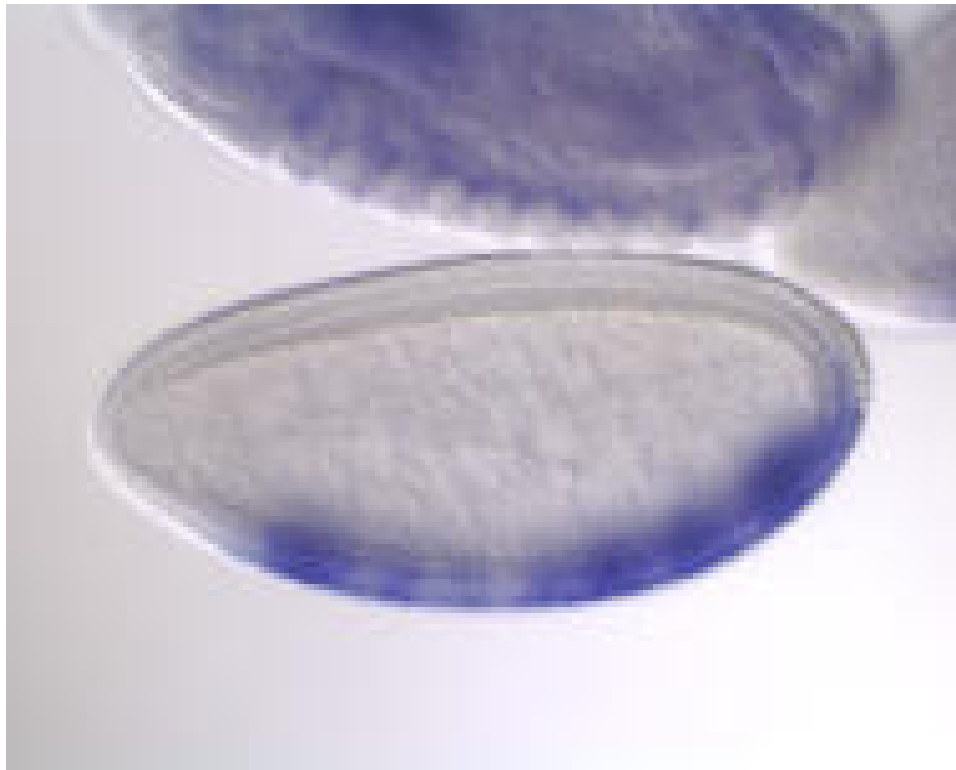

Location: Ventral Type: Promoter ZScore: -0.57437301 PValue: 0.565715413

Supplement: S3 File — Reports consist of in situ hybridization images, ATAC-seq traces, and calculated p-value and Z Score for each region used in the final analysis. (ZIP) [file pgen.1007367.s015.zip › S3_File/GEFmeso_Report.pdf]

gk

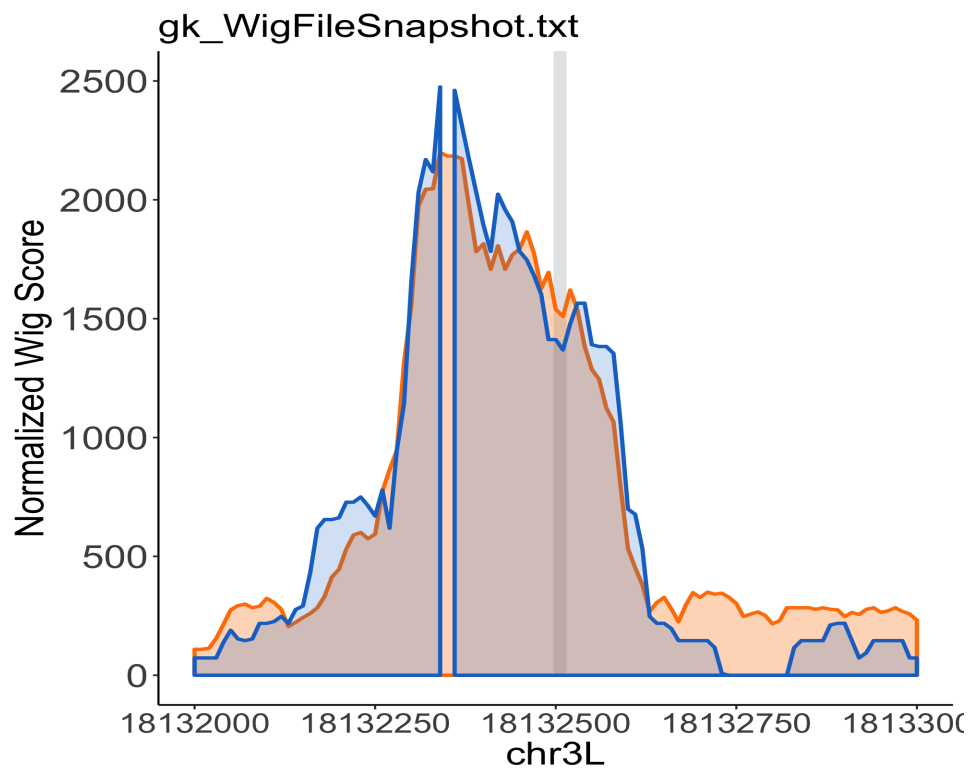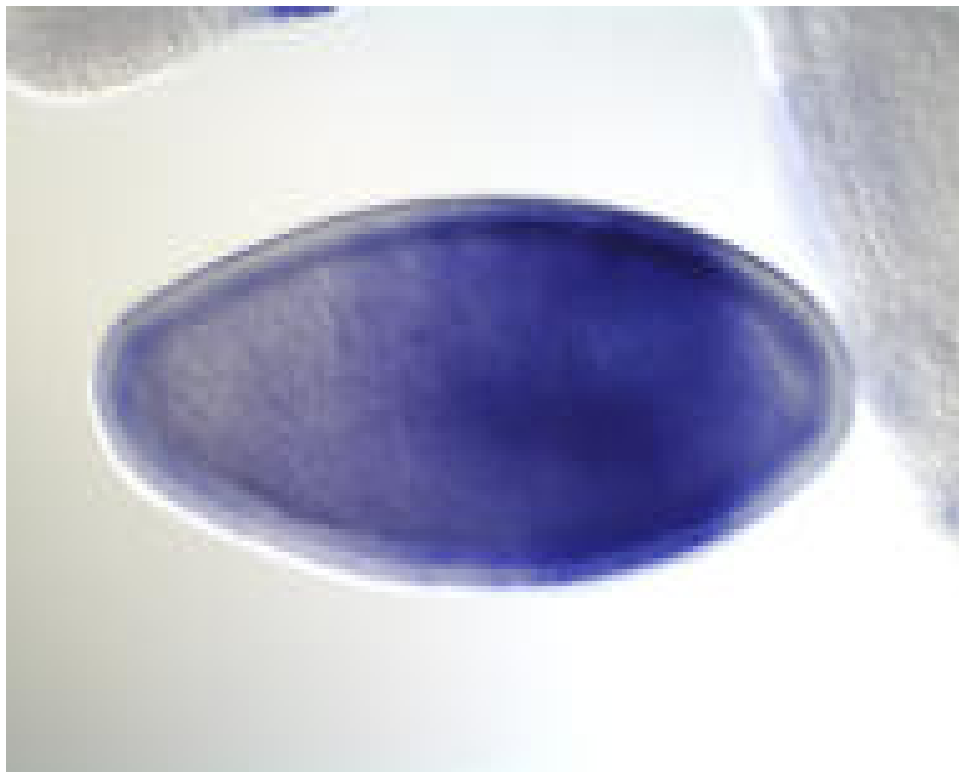

Location: Mostly Post Type: Promoter ZScore: -0.328339476 PValue: 0.742655

Supplement: S3 File — Reports consist of in situ hybridization images, ATAC-seq traces, and calculated p-value and Z Score for each region used in the final analysis. (ZIP) [file pgen.1007367.s015.zip › S3_File/gk_Report.pdf]

gt\_-10\_construct(80)

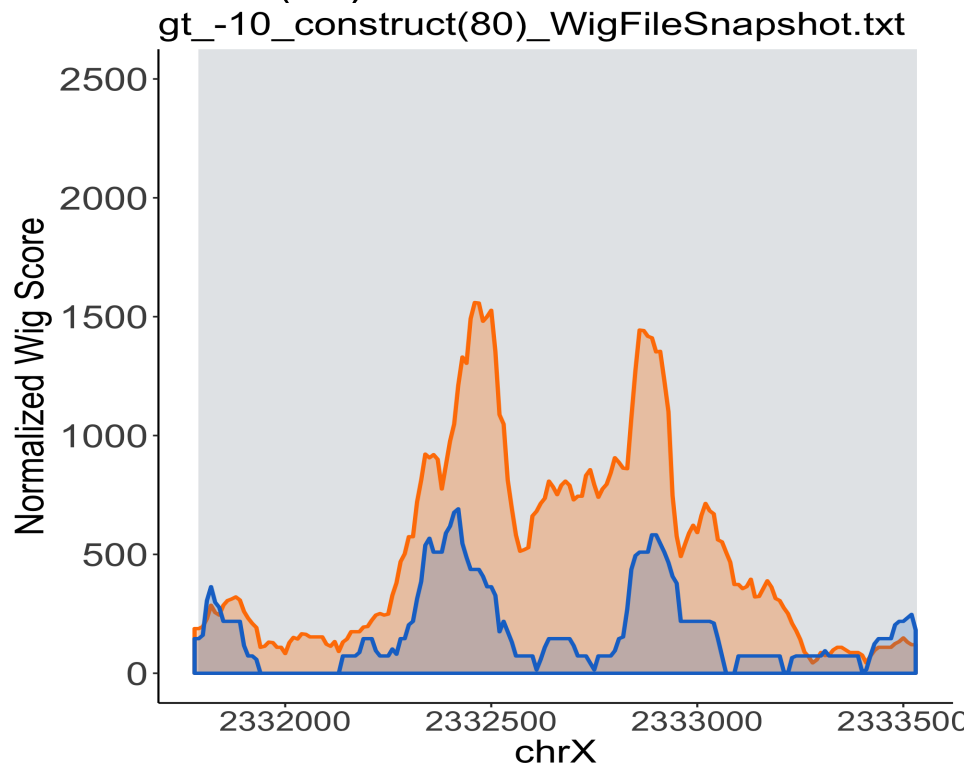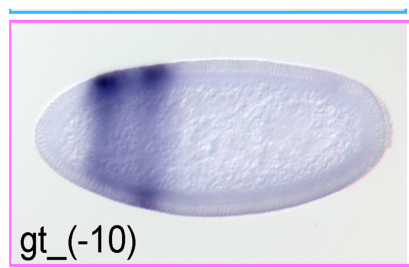

Location: Anterior Type: Enhancer ZScore: 2.21287151 PValue: 0.026906505

Supplement: S3 File — Reports consist of in situ hybridization images, ATAC-seq traces, and calculated p-value and Z Score for each region used in the final analysis. (ZIP) [file pgen.1007367.s015.zip › S3_File/gt_-10_construct(80)_Report.pdf]

gt\_-3\_construct(30)

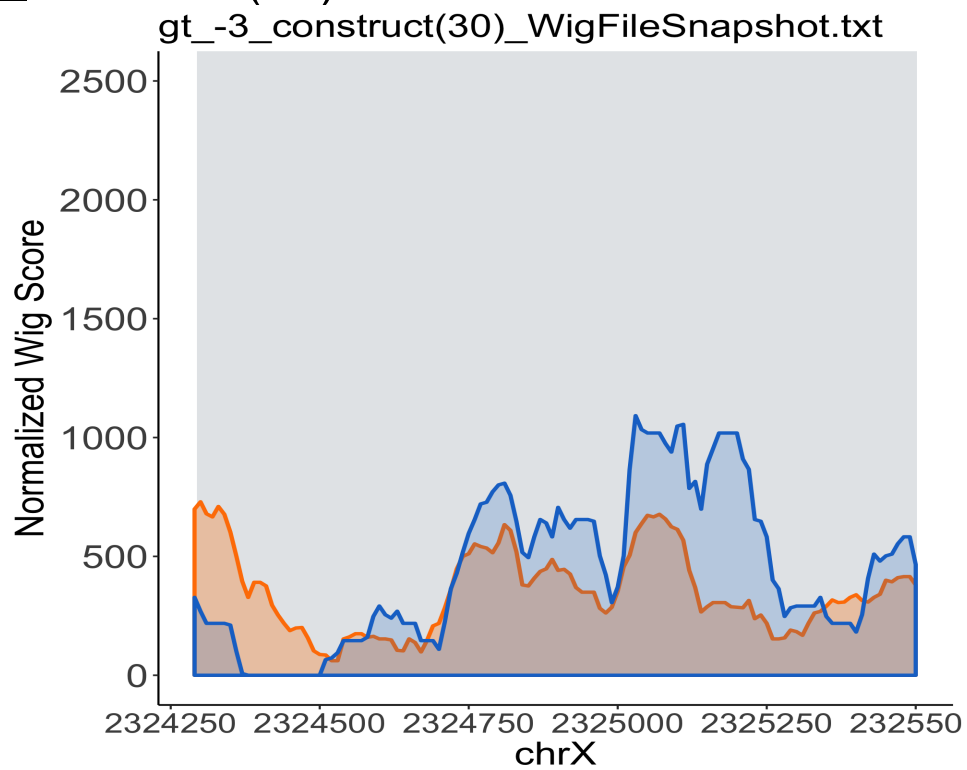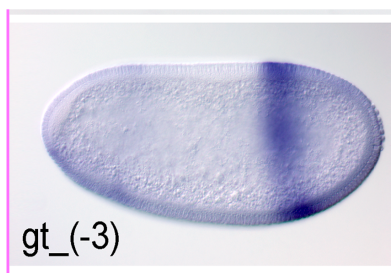

Location: Posterior Type: Enhancer ZScore: 0.49681542 PValue: 0.619319221

Supplement: S3 File — Reports consist of in situ hybridization images, ATAC-seq traces, and calculated p-value and Z Score for each region used in the final analysis. (ZIP) [file pgen.1007367.s015.zip › S3_File/gt_-3_construct(30)_Report.pdf]

gt\_gt23\_61

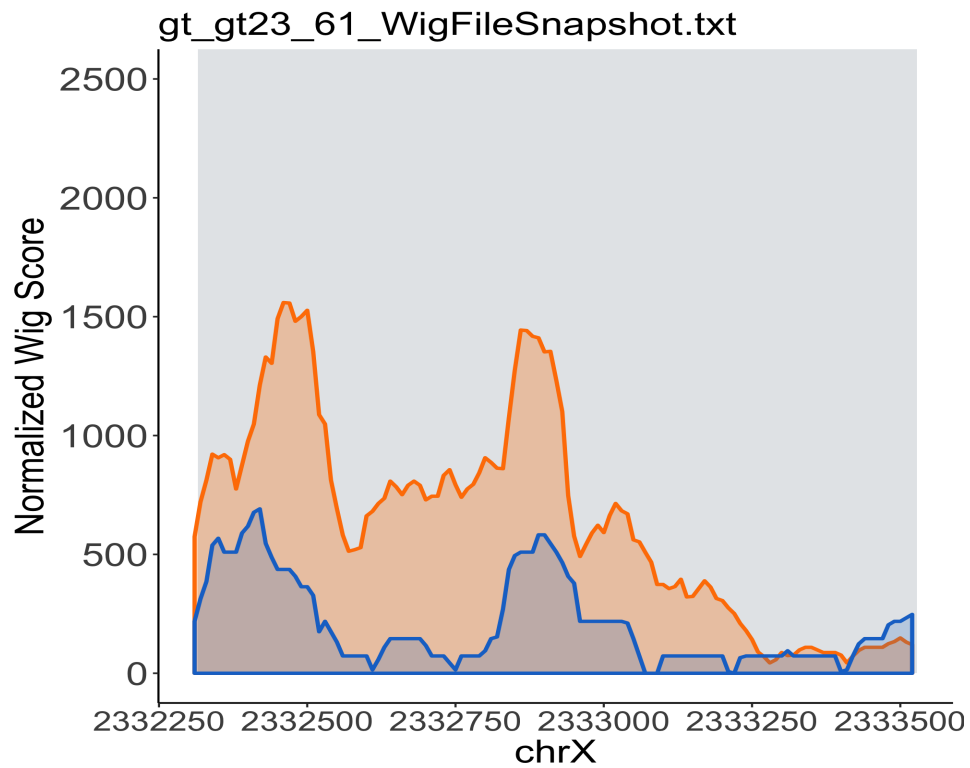

B  
gt23

Location: Anterior Type: Enhancer ZScore: 2.292713851 PValue: 0.021864485

Supplement: S3 File — Reports consist of in situ hybridization images, ATAC-seq traces, and calculated p-value and Z Score for each region used in the final analysis. (ZIP) [file pgen.1007367.s015.zip › S3_File/gt_gt23_61_Report.pdf]

gukh

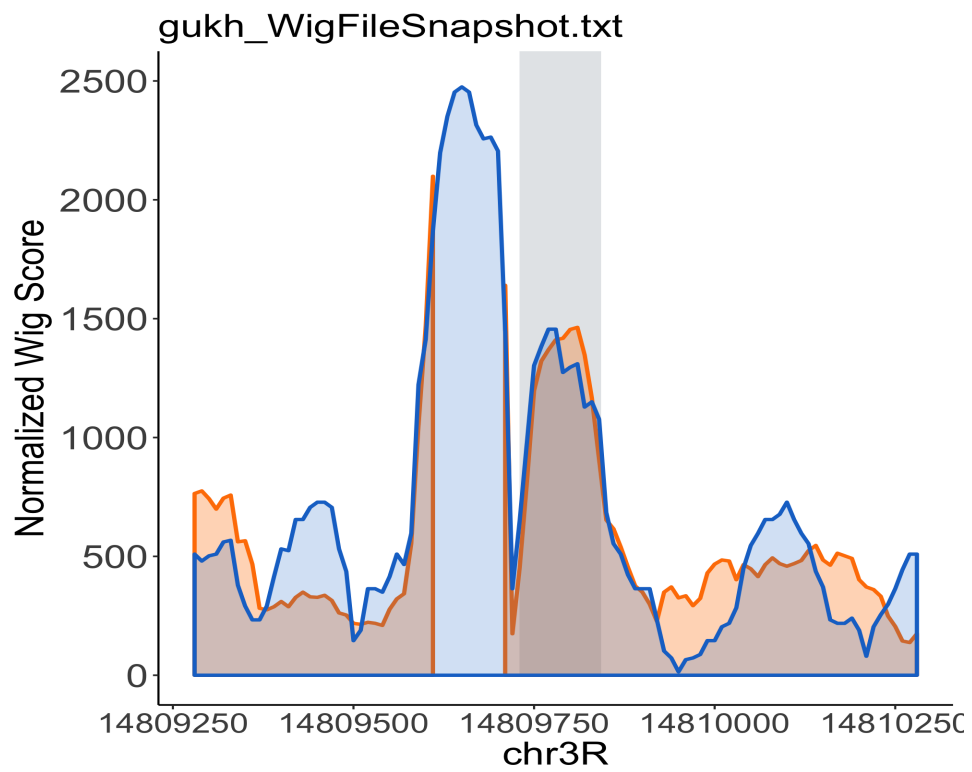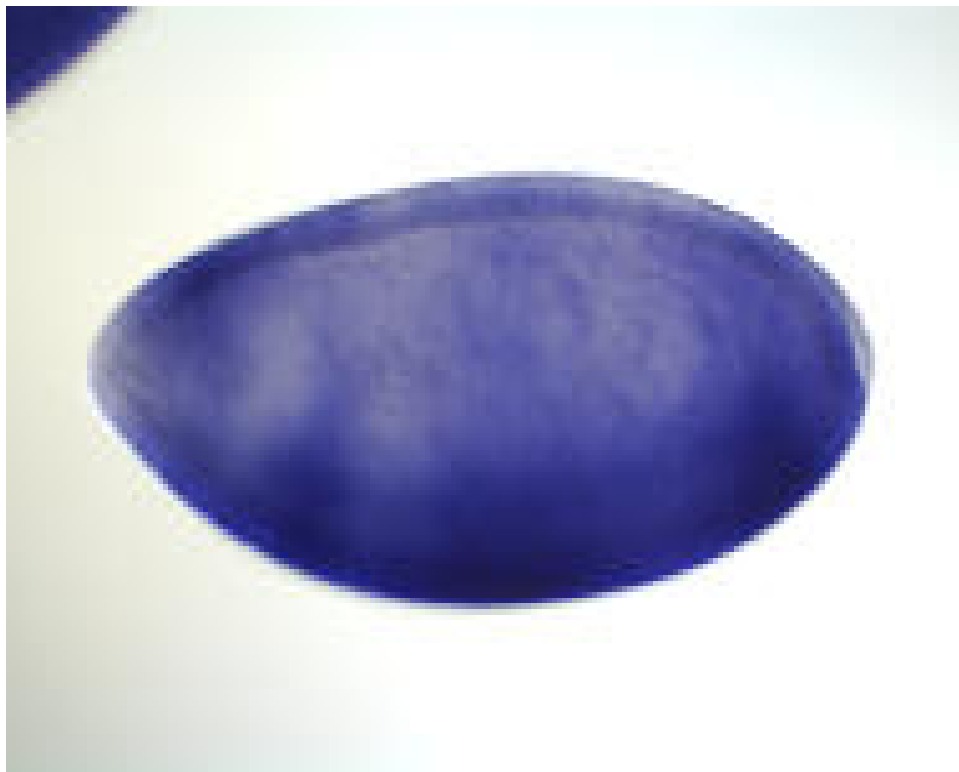

Location: Ventral Type: Promoter ZScore: -0.067318343 PValue: 0.946328274

Supplement: S3 File — Reports consist of in situ hybridization images, ATAC-seq traces, and calculated p-value and Z Score for each region used in the final analysis. (ZIP) [file pgen.1007367.s015.zip › S3_File/gukh_Report.pdf]

h\_stripe0\_85

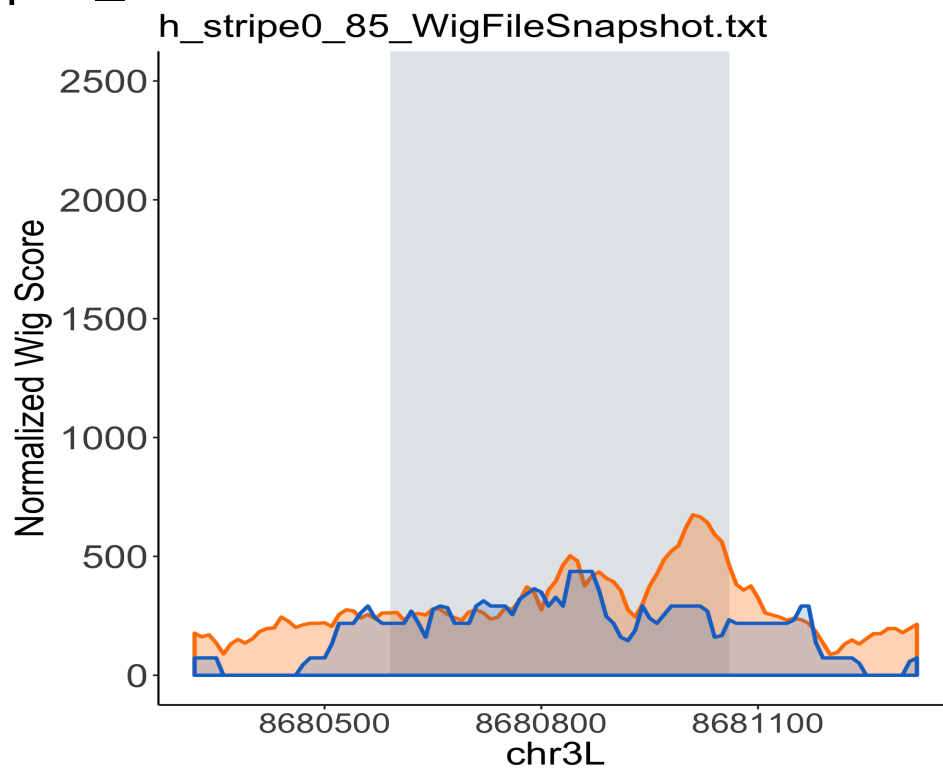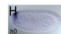

Location: Anterior Type: Enhancer ZScore: 0.658026256 PValue: 0.51052126

Supplement: S3 File — Reports consist of in situ hybridization images, ATAC-seq traces, and calculated p-value and Z Score for each region used in the final analysis. (ZIP) [file pgen.1007367.s015.zip › S3_File/h_stripe0_85_Report.pdf]

h\_stripe1\_68

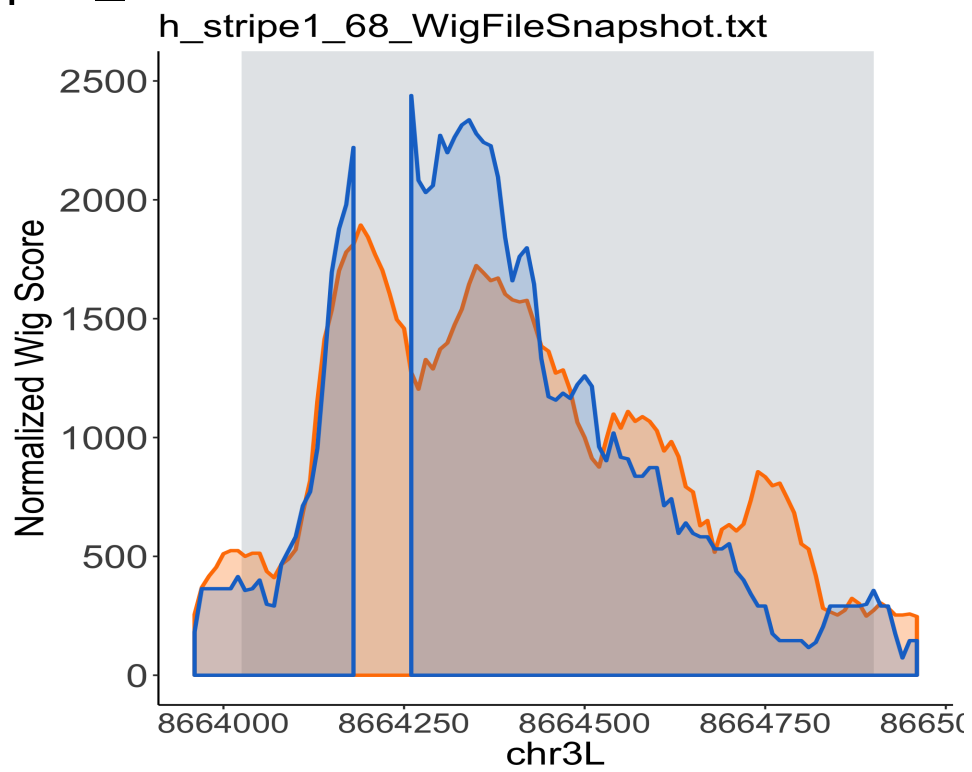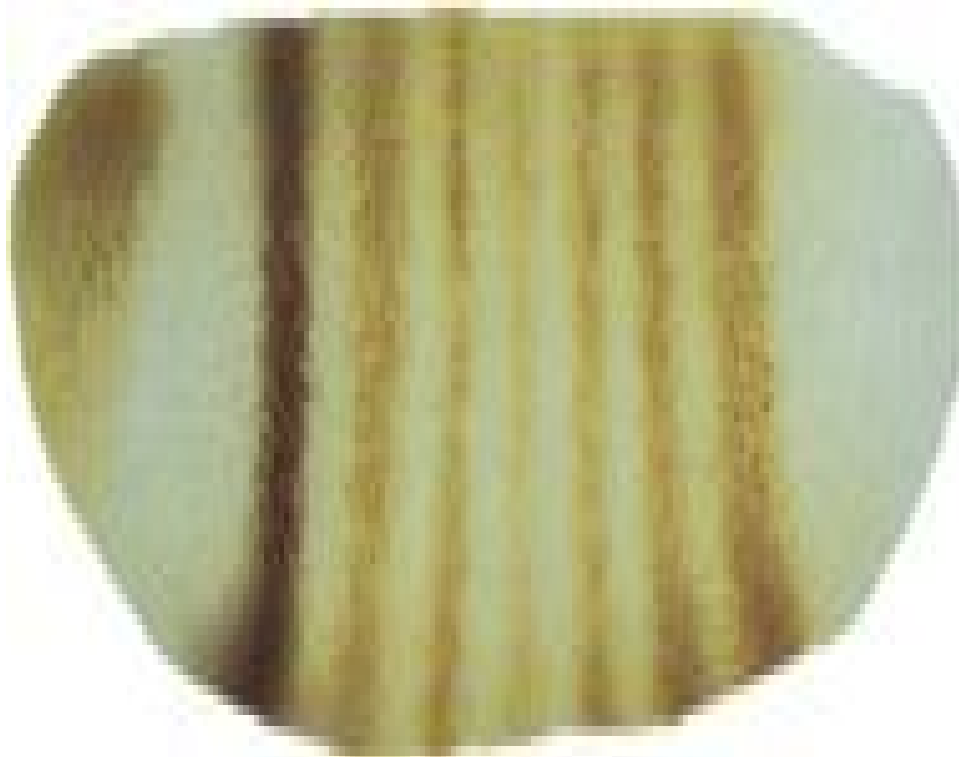

Location: Anterior Type: Enhancer ZScore: -0.317332895 PValue: 0.75099102

Supplement: S3 File — Reports consist of in situ hybridization images, ATAC-seq traces, and calculated p-value and Z Score for each region used in the final analysis. (ZIP) [file pgen.1007367.s015.zip › S3_File/h_stripe1_68_Report.pdf]

h\_stripe5(40)

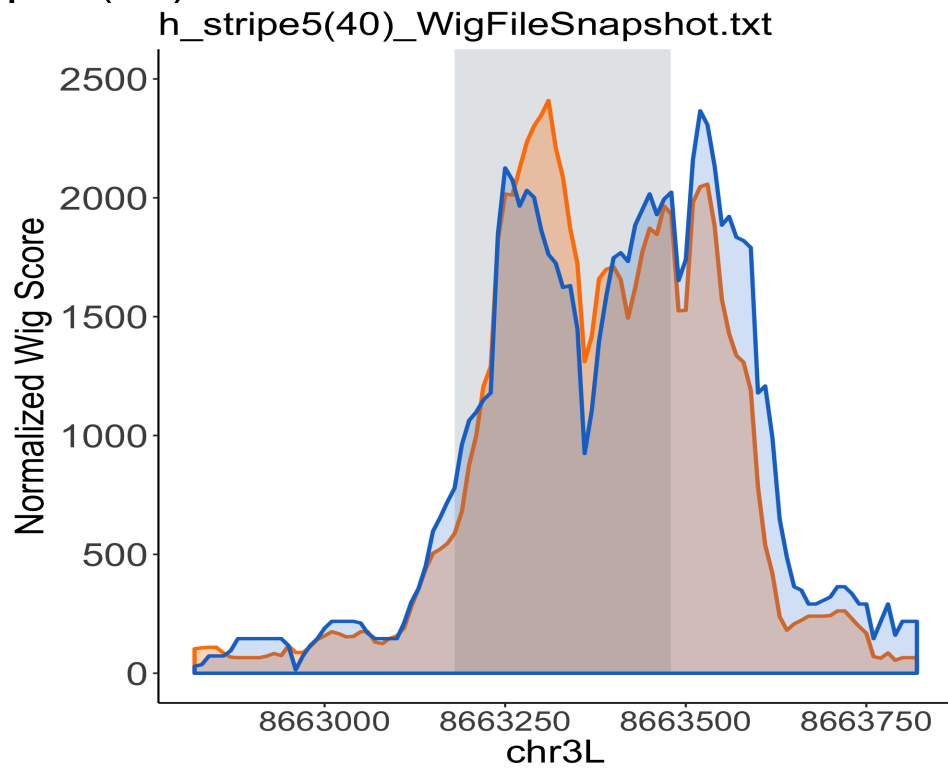

Location: Posterior Type: Enhancer ZScore: -0.158879484 PValue: 0.873763825

Supplement: S3 File — Reports consist of in situ hybridization images, ATAC-seq traces, and calculated p-value and Z Score for each region used in the final analysis. (ZIP) [file pgen.1007367.s015.zip › S3_File/h_stripe5(40)_Report.pdf]

## hb\_anterior\_activator\_55

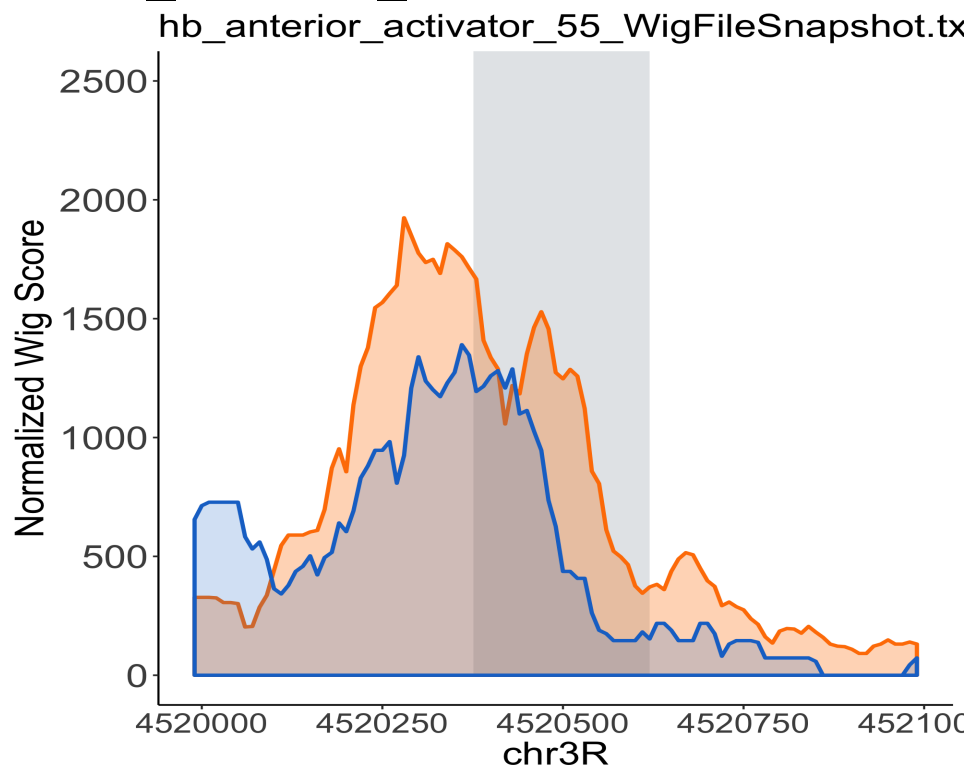

Location: Anterior Type: Enhancer ZScore: 0.983385795 PValue: 0.325417599

Supplement: S3 File — Reports consist of in situ hybridization images, ATAC-seq traces, and calculated p-value and Z Score for each region used in the final analysis. (ZIP) [file pgen.1007367.s015.zip › S3_File/hb_anterior_activator_55_Report.pdf]

## hb\_distal\_nonminimal

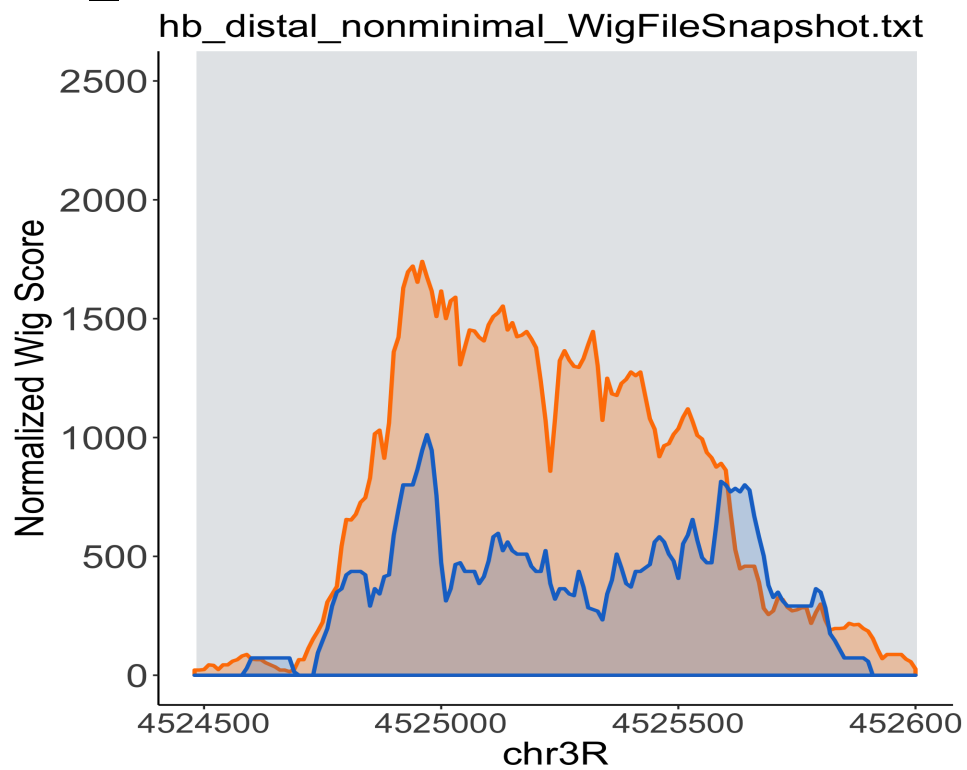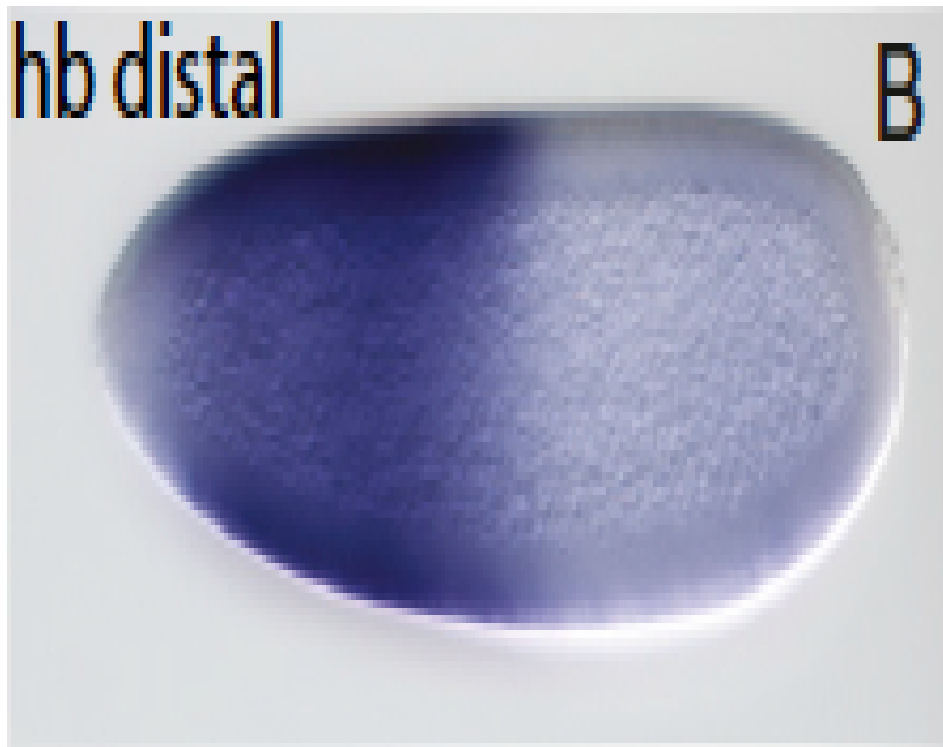

Location: Anterior Type: Enhancer ZScore: 1.618054918 PValue: 0.105650763

Supplement: S3 File — Reports consist of in situ hybridization images, ATAC-seq traces, and calculated p-value and Z Score for each region used in the final analysis. (ZIP) [file pgen.1007367.s015.zip › S3_File/hb_distal_nonminimal_Report.pdf]

HC\_03F\_(70)

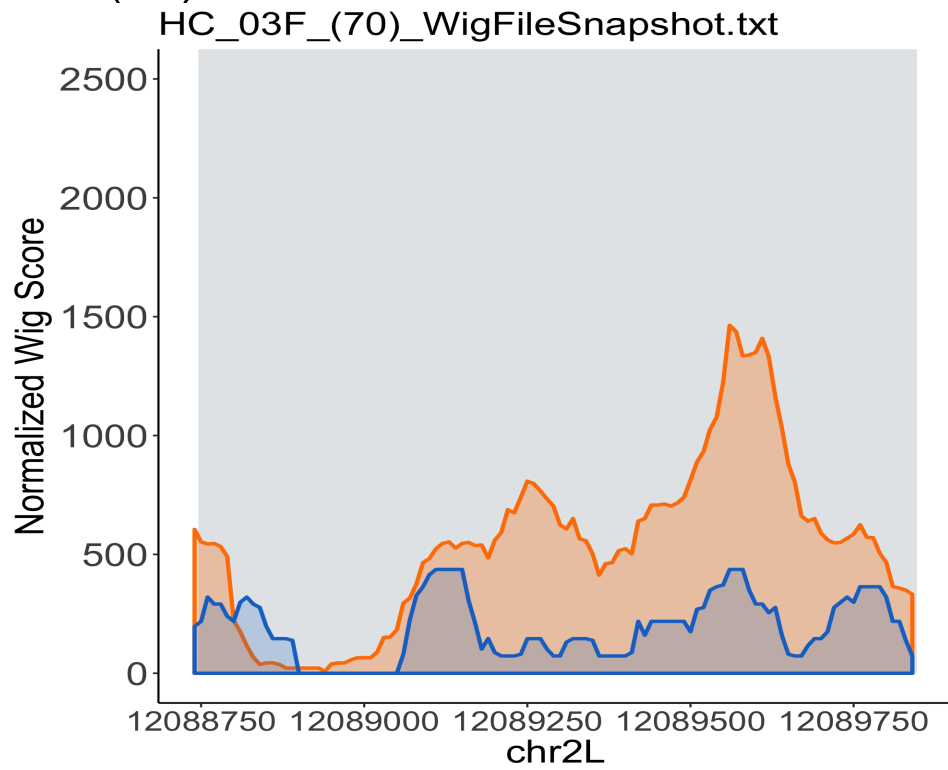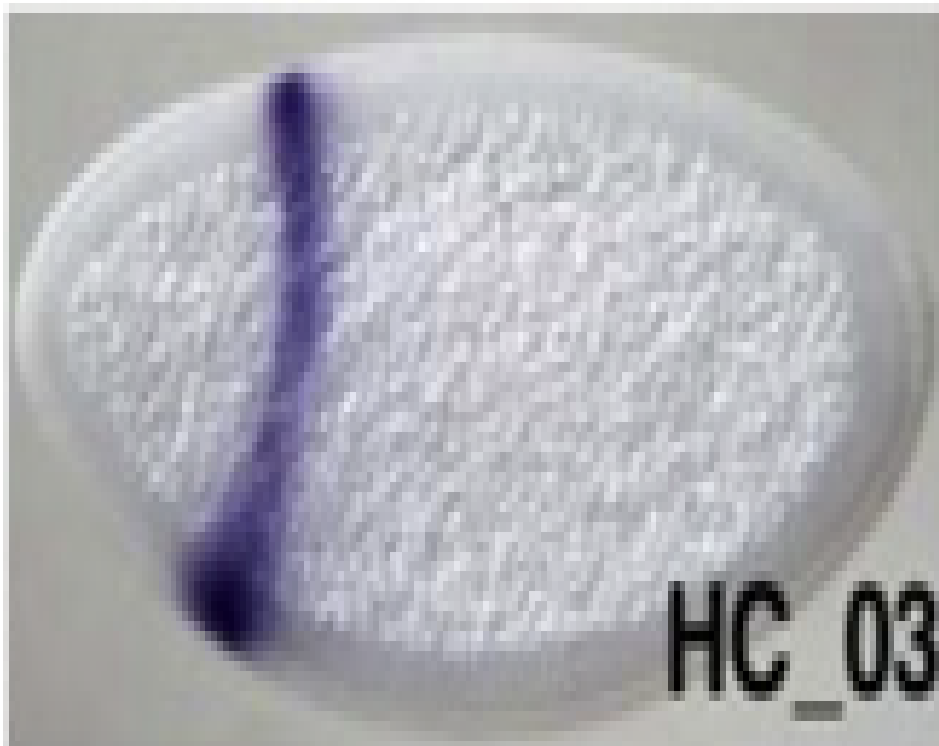

Location: Anterior Type: Enhancer ZScore: 2.147946431 PValue: 0.031718011

Supplement: S3 File — Reports consist of in situ hybridization images, ATAC-seq traces, and calculated p-value and Z Score for each region used in the final analysis. (ZIP) [file pgen.1007367.s015.zip › S3_File/HC_03F_(70)_Report.pdf]

HC\_05F\_(70)

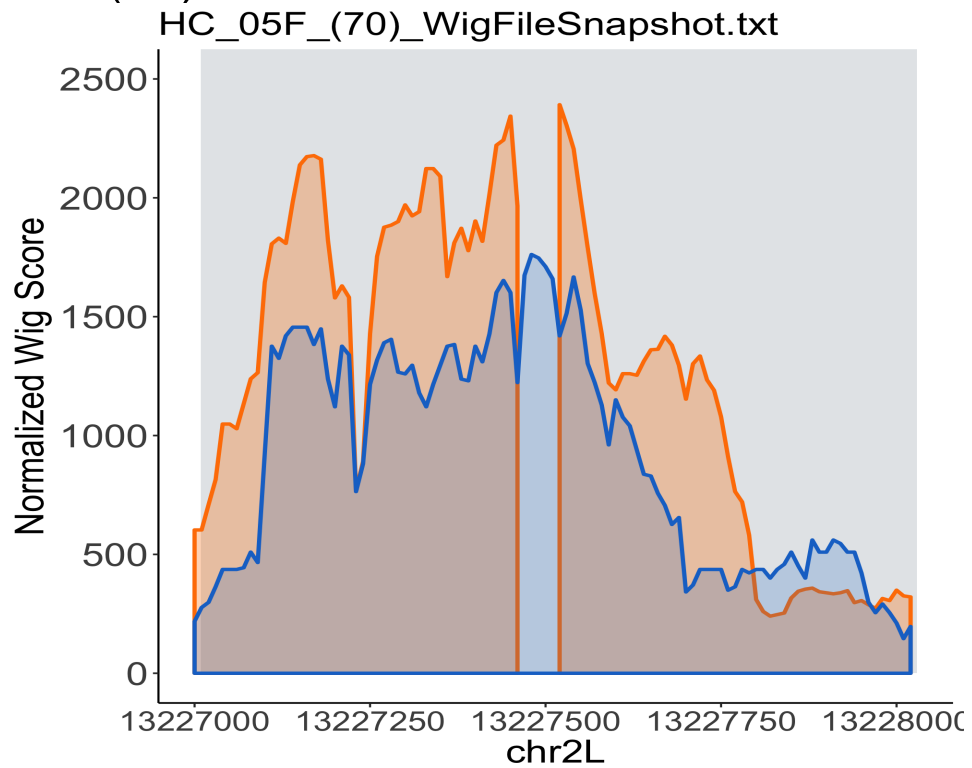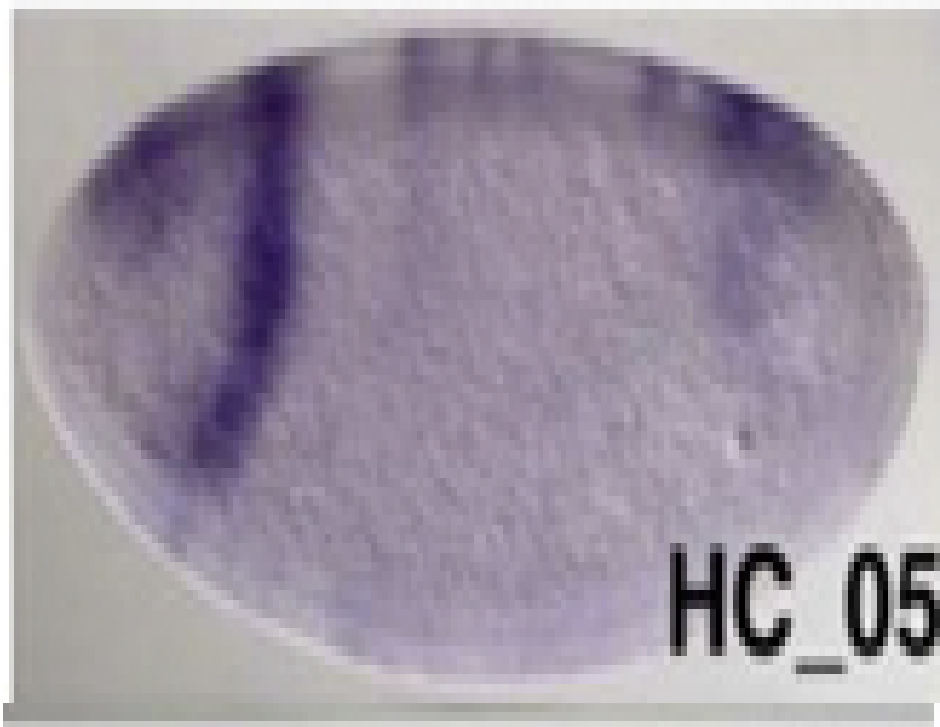

Location: Anterior Type: Enhancer ZScore: 0.814185316 PValue: 0.415538796

Supplement: S3 File — Reports consist of in situ hybridization images, ATAC-seq traces, and calculated p-value and Z Score for each region used in the final analysis. (ZIP) [file pgen.1007367.s015.zip › S3_File/HC_05F_(70)_Report.pdf]

HC\_11F\_(94)

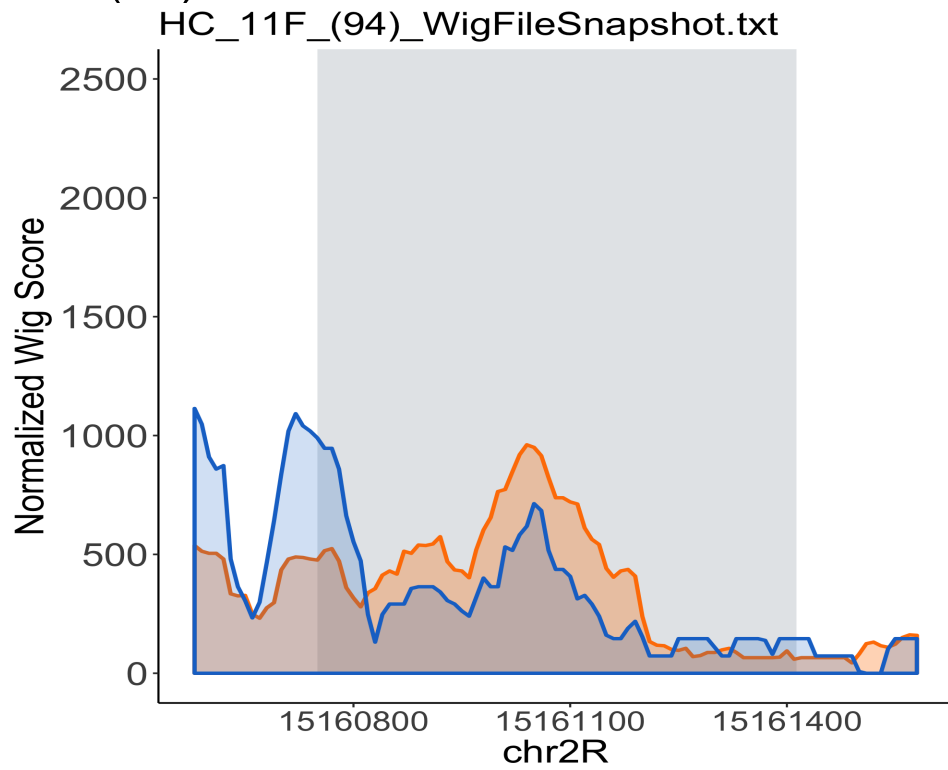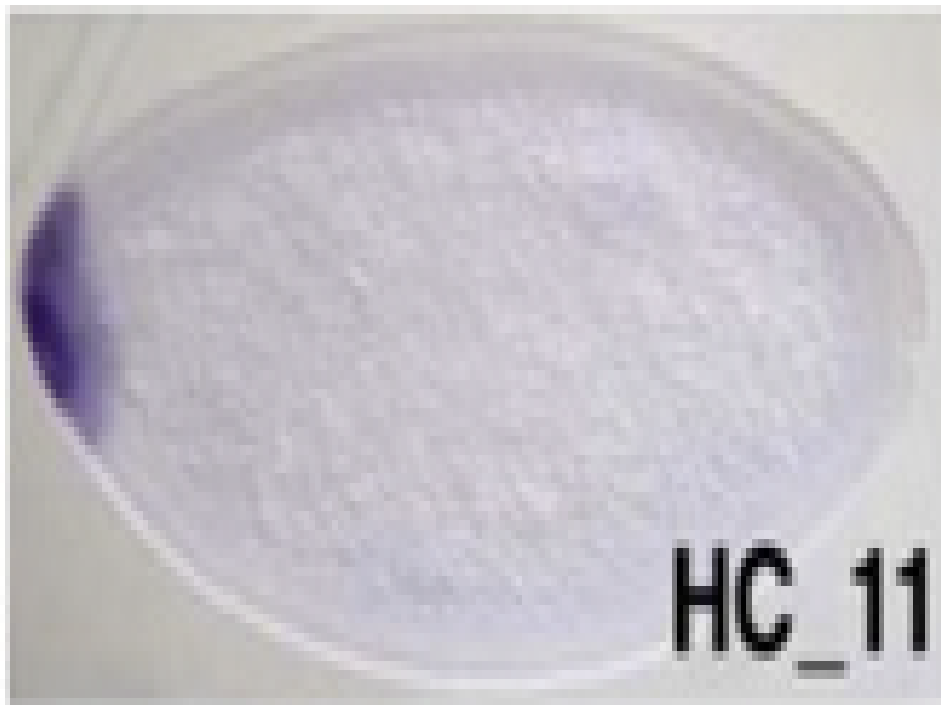

Location: Anterior Type: Enhancer ZScore: 0.517953886 PValue: 0.604490444

Supplement: S3 File — Reports consist of in situ hybridization images, ATAC-seq traces, and calculated p-value and Z Score for each region used in the final analysis. (ZIP) [file pgen.1007367.s015.zip › S3_File/HC_11F_(94)_Report.pdf]

HC\_14R\_(84)

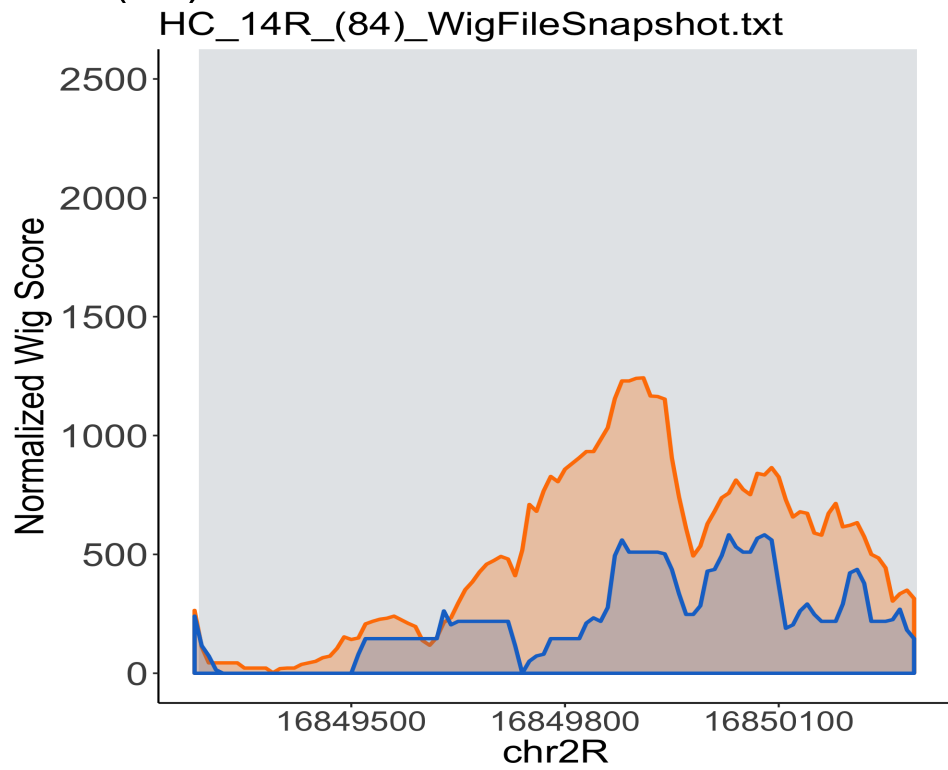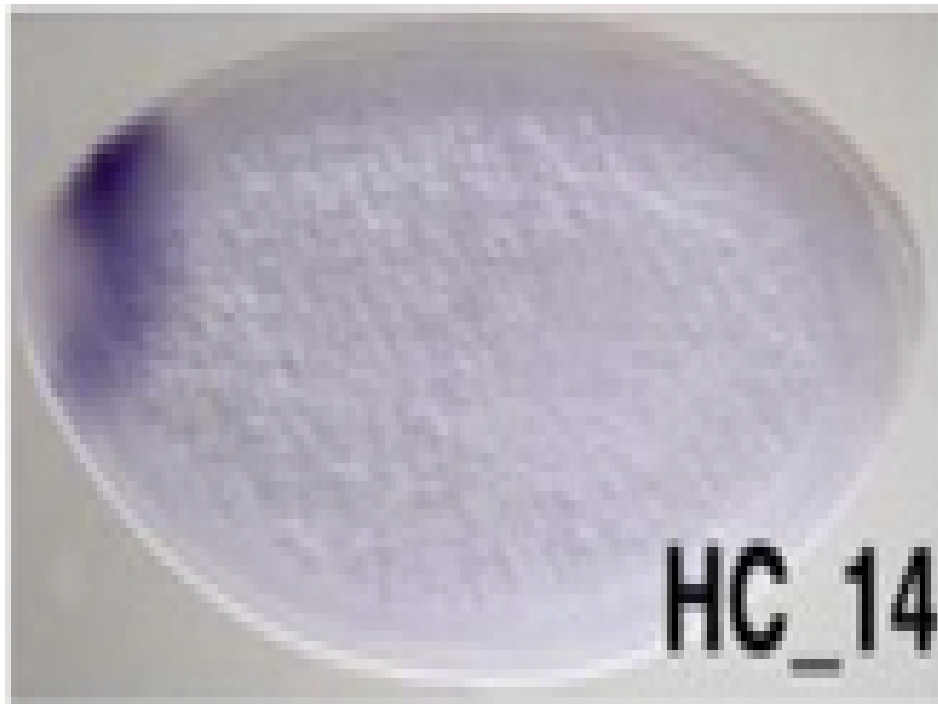

Location: Anterior Type: Enhancer ZScore: 1.72908305 PValue: 0.083794232

Supplement: S3 File — Reports consist of in situ hybridization images, ATAC-seq traces, and calculated p-value and Z Score for each region used in the final analysis. (ZIP) [file pgen.1007367.s015.zip › S3_File/HC_14R_(84)_Report.pdf]

HC\_18R\_(71.5)

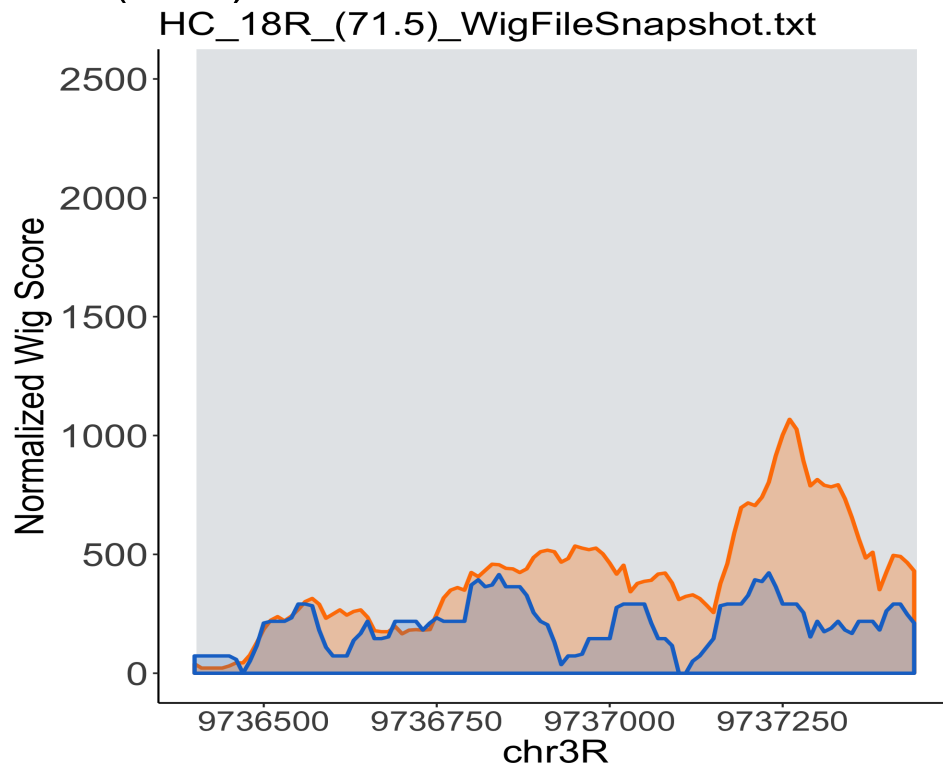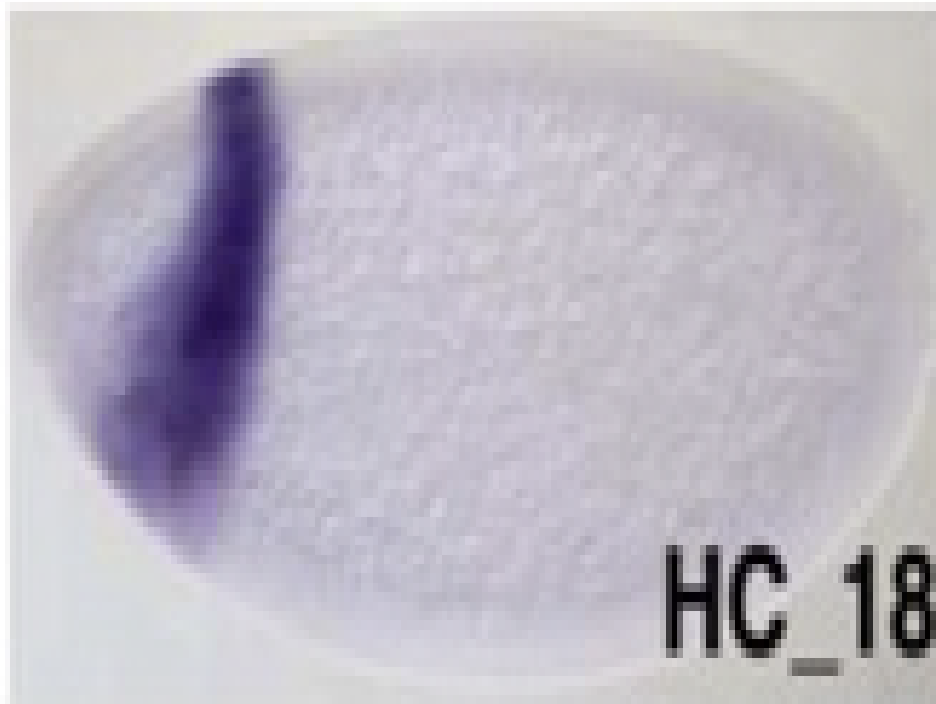

Location: Anterior Type: Enhancer ZScore: 1.468334045 PValue: 0.142013507

Supplement: S3 File — Reports consist of in situ hybridization images, ATAC-seq traces, and calculated p-value and Z Score for each region used in the final analysis. (ZIP) [file pgen.1007367.s015.zip › S3_File/HC_18R_(71.5)_Report.pdf]

HC\_29F\_(70)

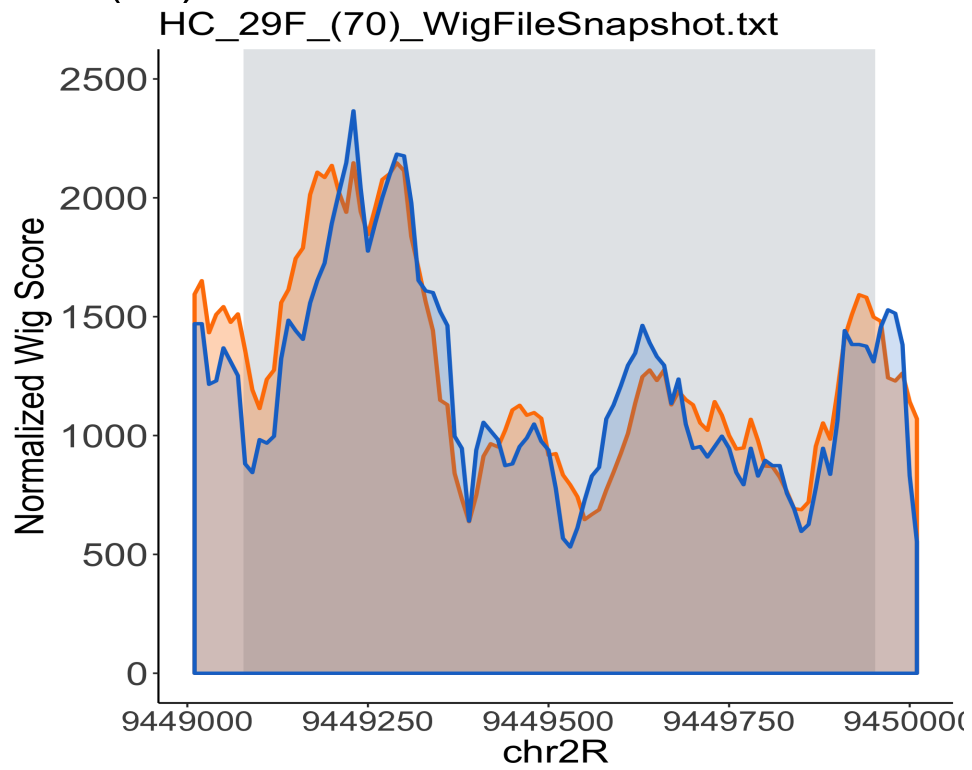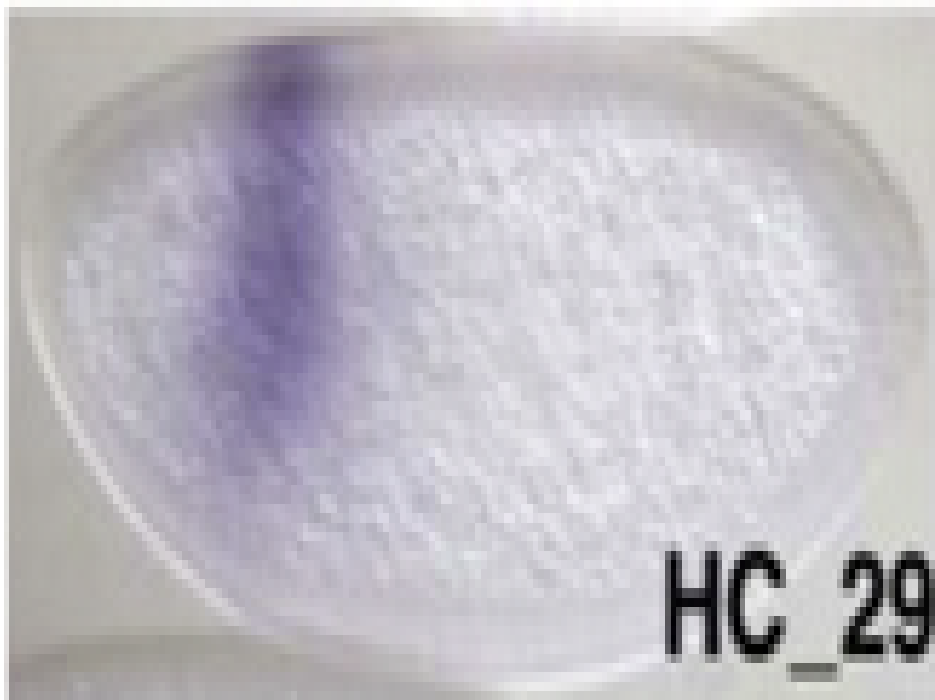

Location: Anterior Type: Enhancer ZScore: 0.041881632 PValue: 0.966593059

Supplement: S3 File — Reports consist of in situ hybridization images, ATAC-seq traces, and calculated p-value and Z Score for each region used in the final analysis. (ZIP) [file pgen.1007367.s015.zip › S3_File/HC_29F_(70)_Report.pdf]

HC\_33F\_(74)

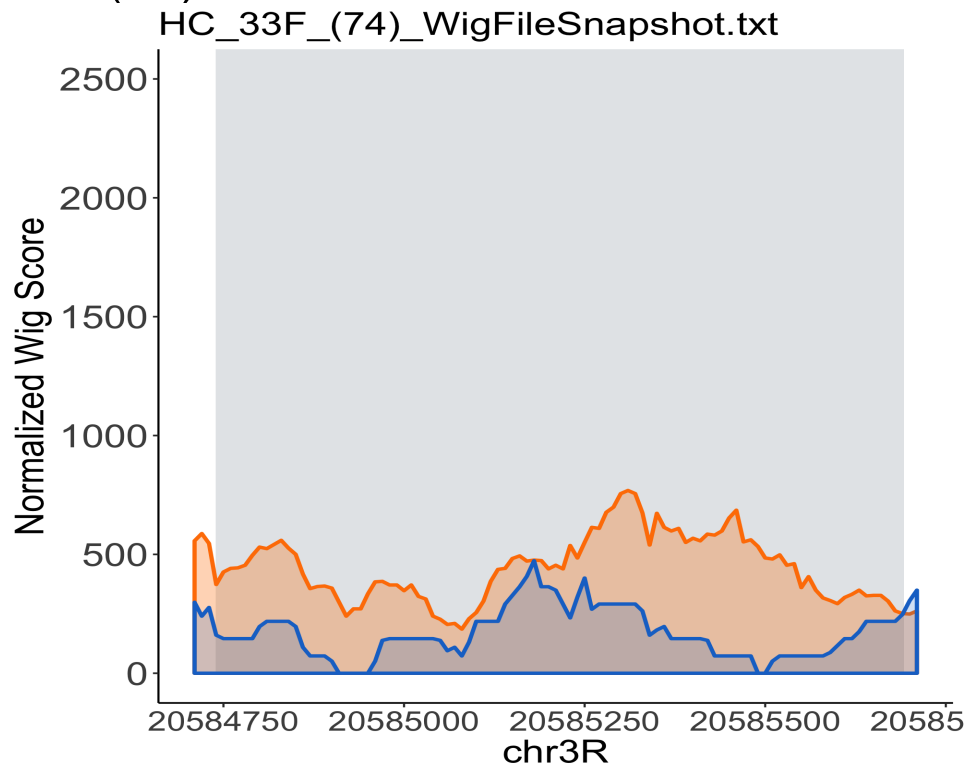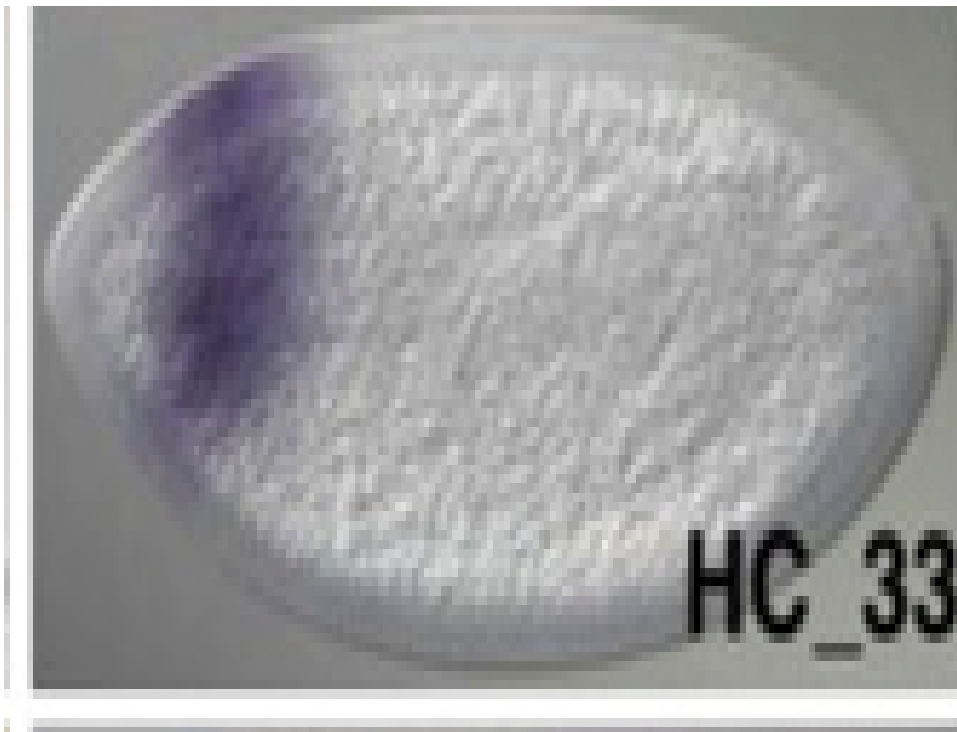

Location: Anterior Type: Enhancer ZScore: 2.027631114 PValue: 0.042597911

Supplement: S3 File — Reports consist of in situ hybridization images, ATAC-seq traces, and calculated p-value and Z Score for each region used in the final analysis. (ZIP) [file pgen.1007367.s015.zip › S3_File/HC_33F_(74)_Report.pdf]

HC\_34F\_(86)

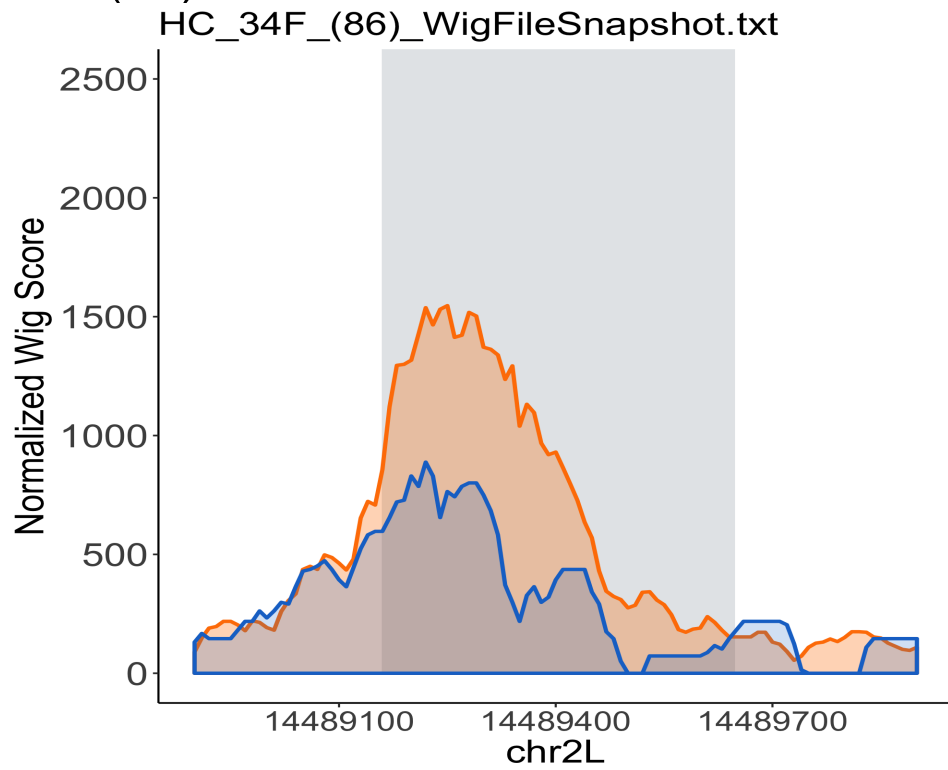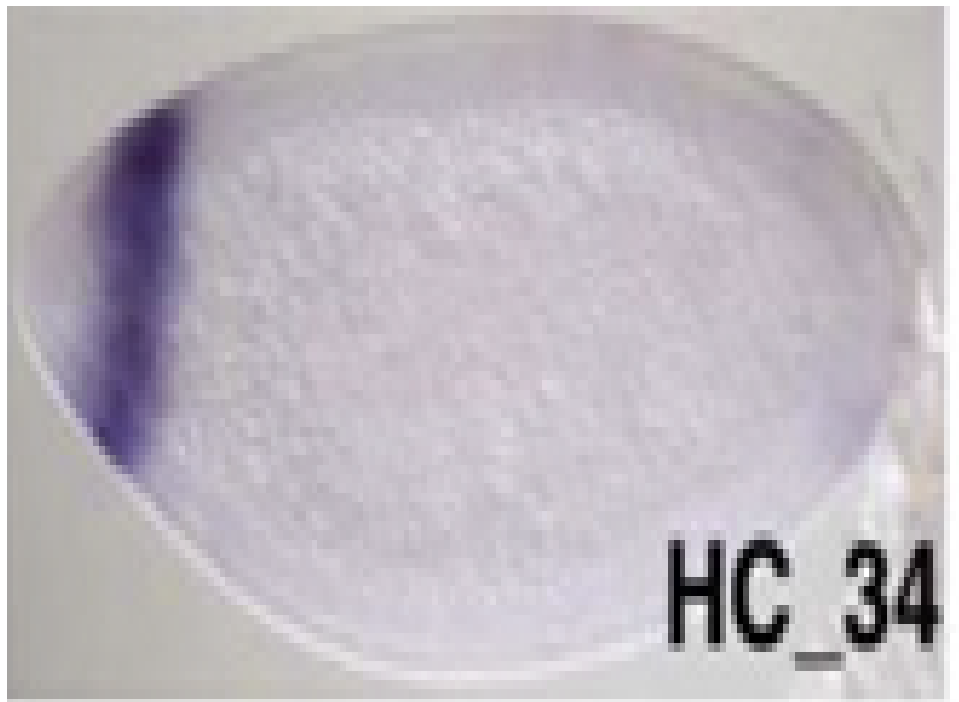

Location: Anterior Type: Enhancer ZScore: 1.591837548 PValue: 0.111421211

Supplement: S3 File — Reports consist of in situ hybridization images, ATAC-seq traces, and calculated p-value and Z Score for each region used in the final analysis. (ZIP) [file pgen.1007367.s015.zip › S3_File/HC_34F_(86)_Report.pdf]

Hph

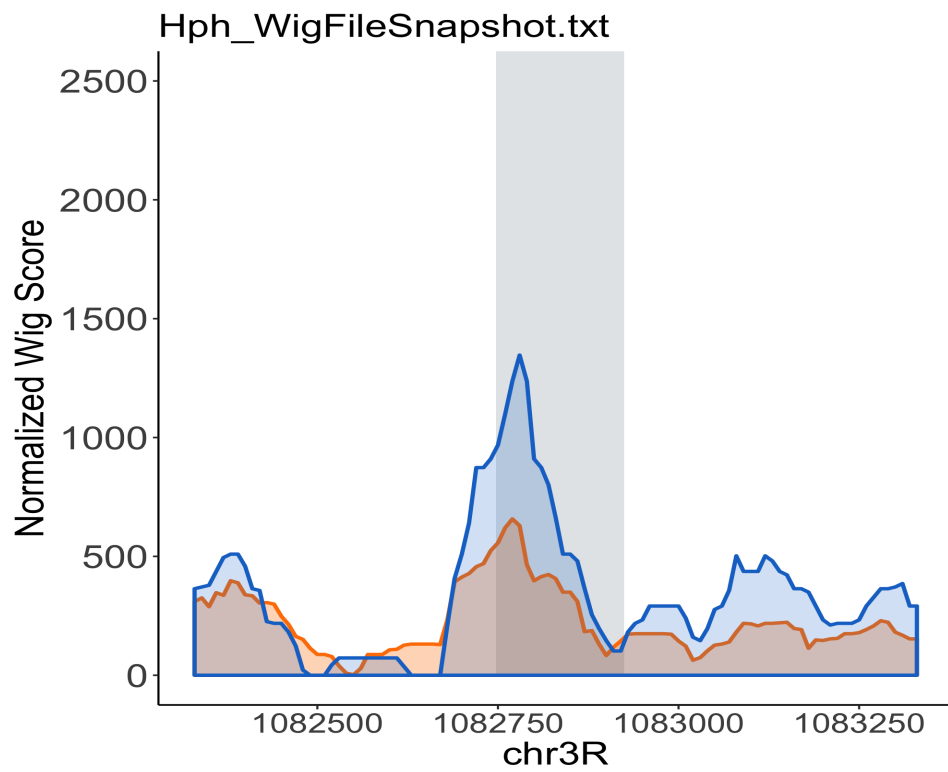

Location: Mostly Ant Type: Promoter ZScore: -1.389717902 PValue: 0.164614556

Supplement: S3 File — Reports consist of in situ hybridization images, ATAC-seq traces, and calculated p-value and Z Score for each region used in the final analysis. (ZIP) [file pgen.1007367.s015.zip › S3_File/Hph_Report.pdf]

htl

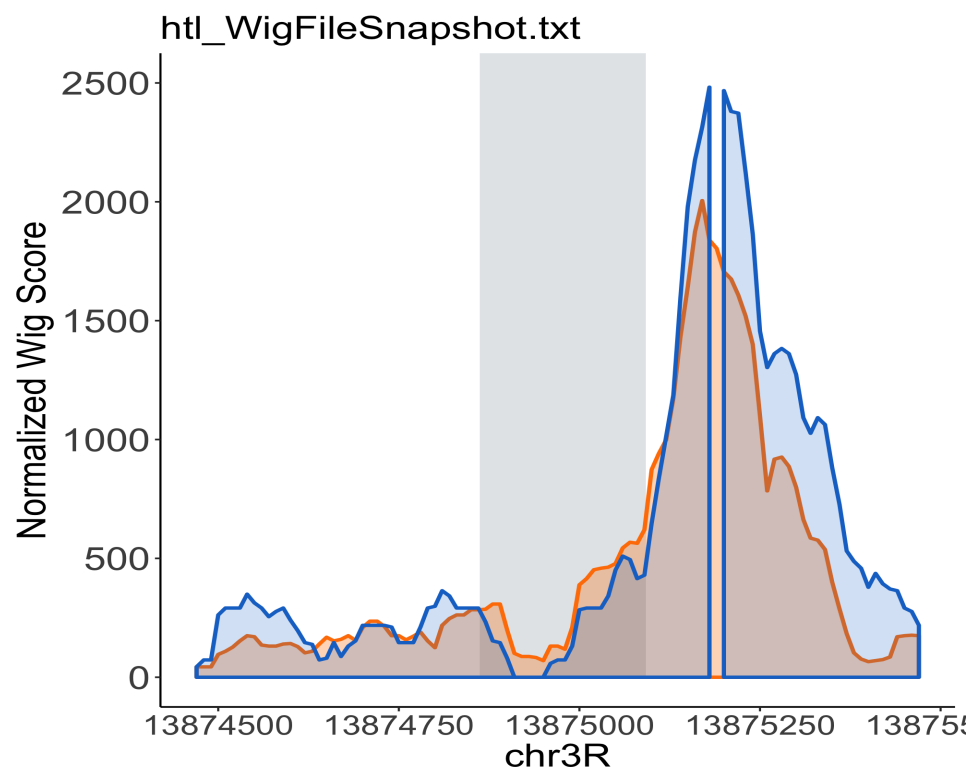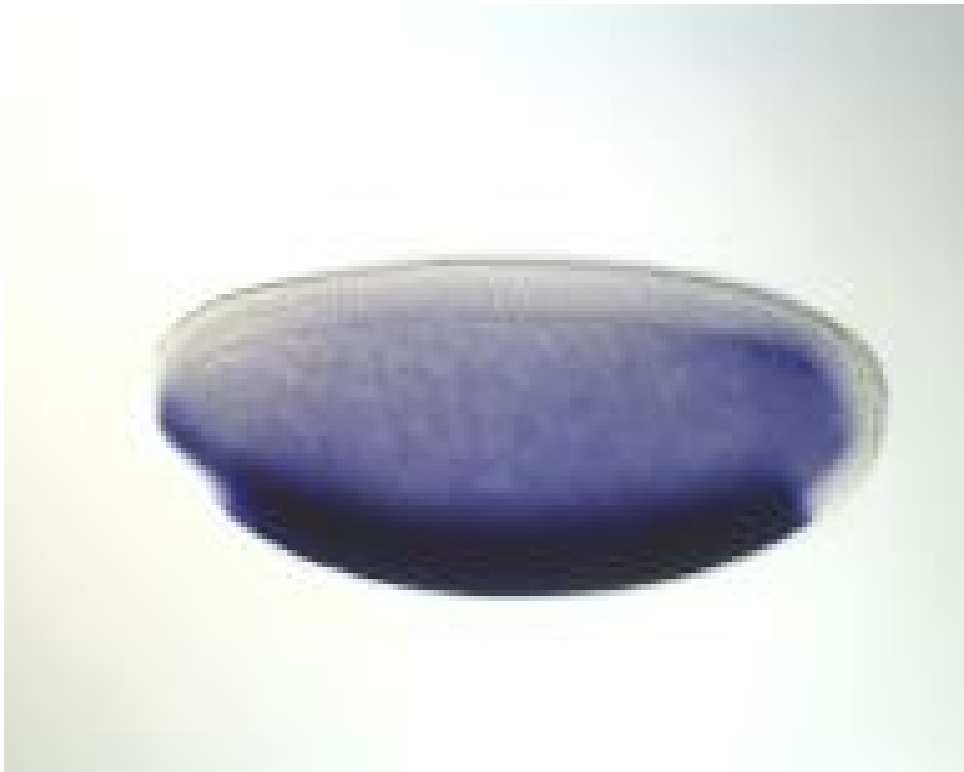

Location: Ventral Type: Promoter ZScore: 0.813511374 PValue: 0.415924928

Supplement: S3 File — Reports consist of in situ hybridization images, ATAC-seq traces, and calculated p-value and Z Score for each region used in the final analysis. (ZIP) [file pgen.1007367.s015.zip › S3_File/htl_Report.pdf]

# htl\_Stathopoulos

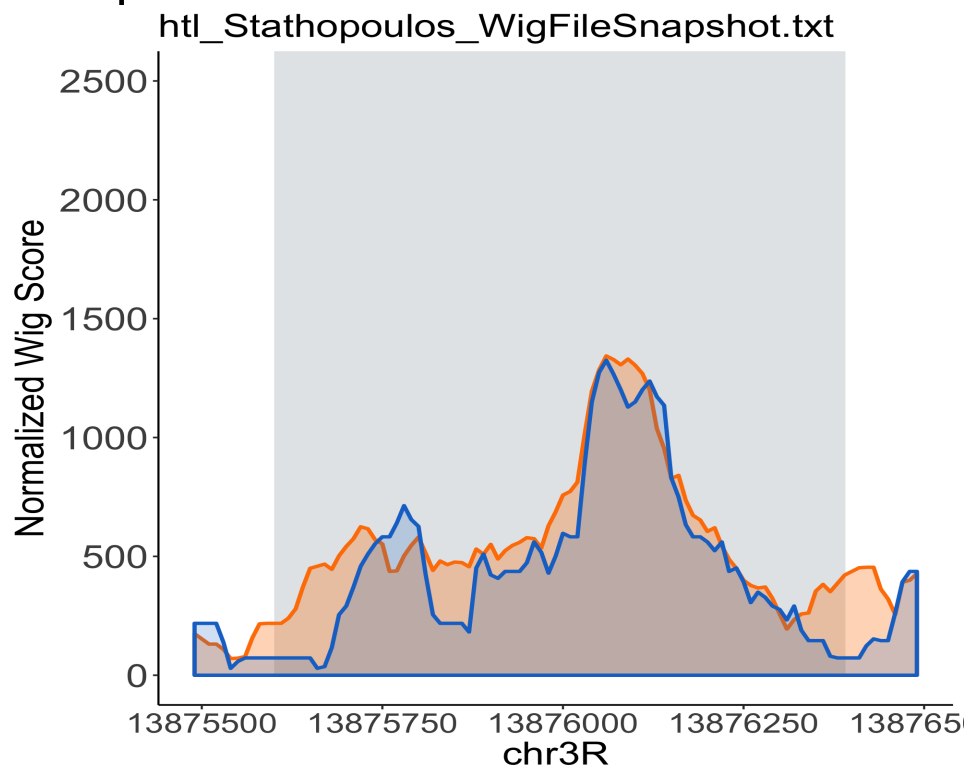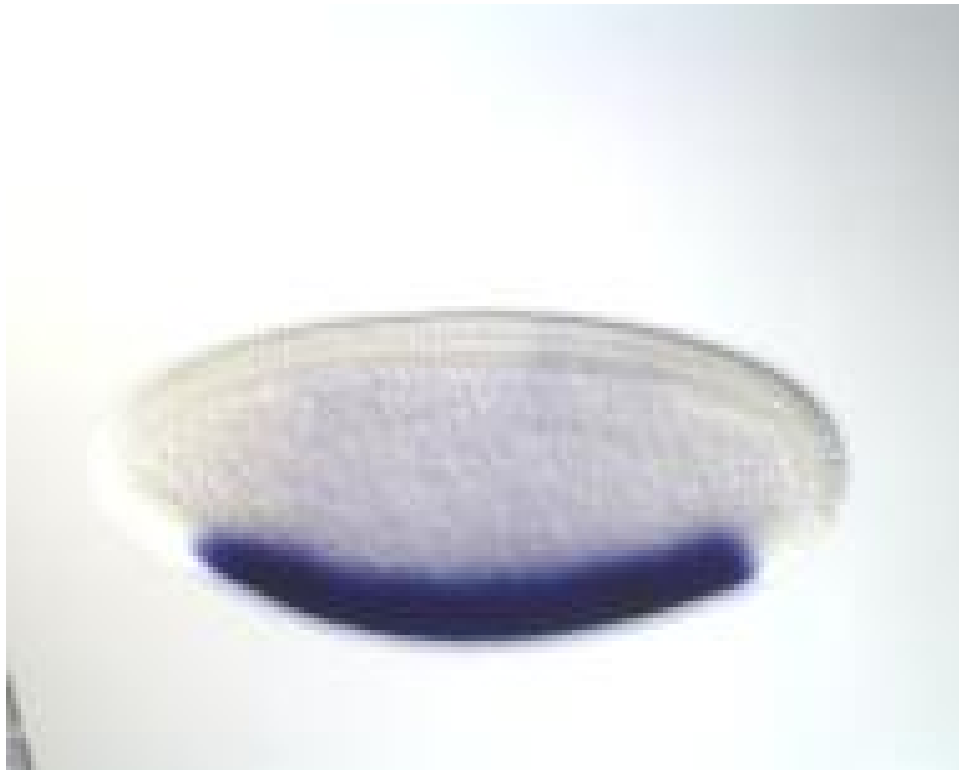

Location: Ventral Type: Enhancer ZScore: 0.410308911 PValue: 0.681579356

Supplement: S3 File — Reports consist of in situ hybridization images, ATAC-seq traces, and calculated p-value and Z Score for each region used in the final analysis. (ZIP) [file pgen.1007367.s015.zip › S3_File/htl_Stathopoulos_Report.pdf]

ind

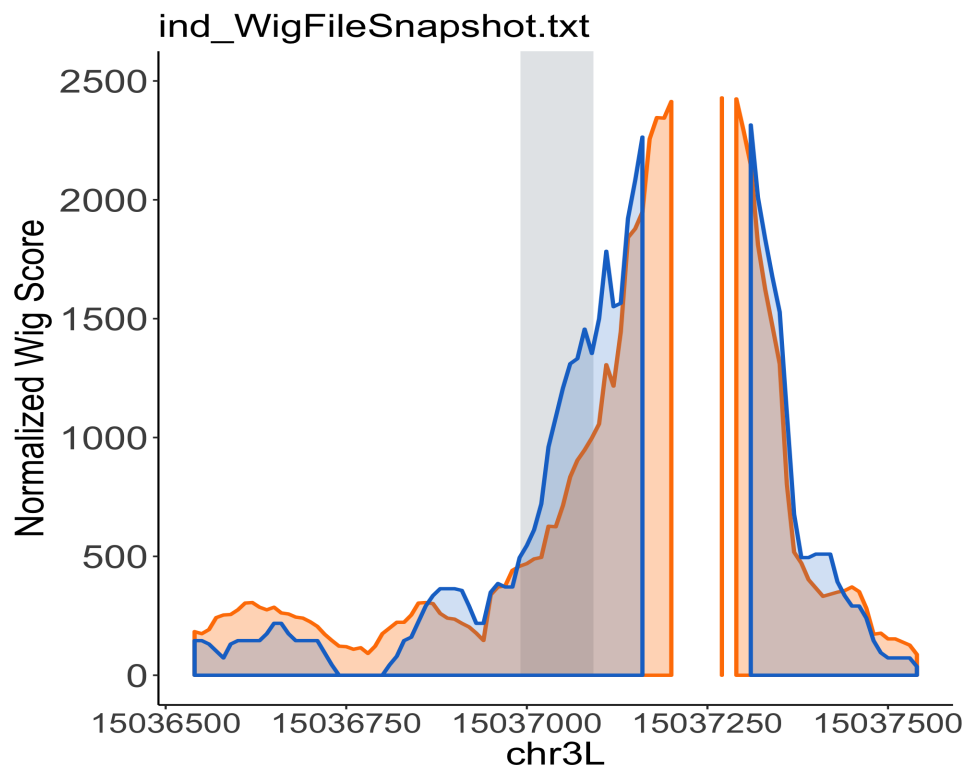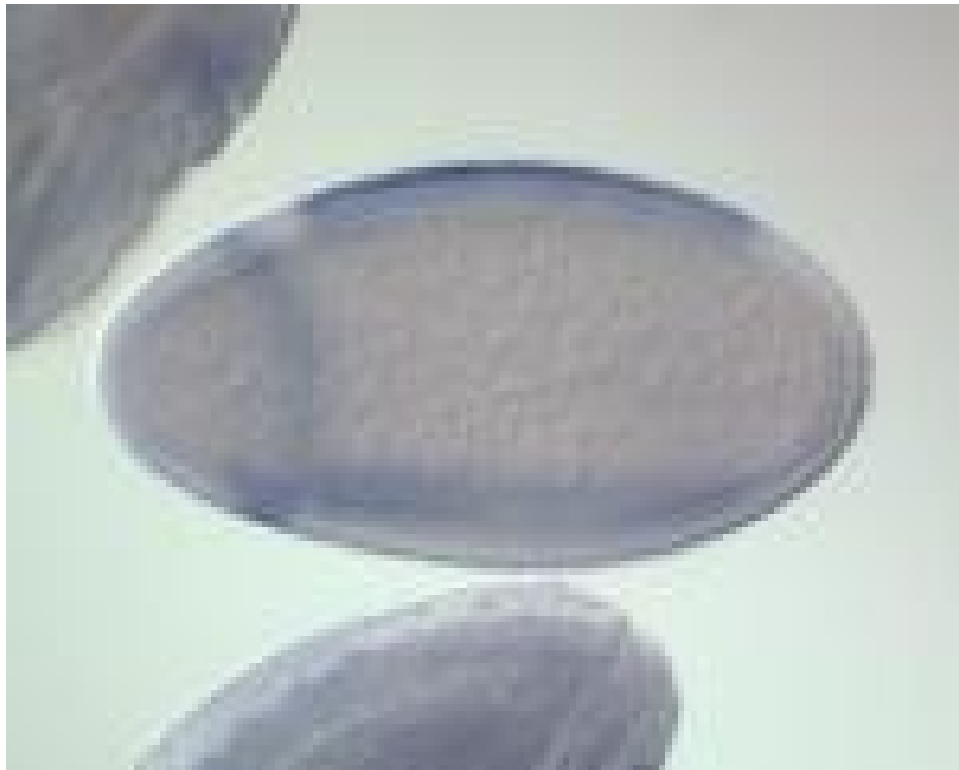

Location: Ventral Type: Promoter ZScore: -0.910940863 PValue: 0.362326534

Supplement: S3 File — Reports consist of in situ hybridization images, ATAC-seq traces, and calculated p-value and Z Score for each region used in the final analysis. (ZIP) [file pgen.1007367.s015.zip › S3_File/ind_Report.pdf]

jar

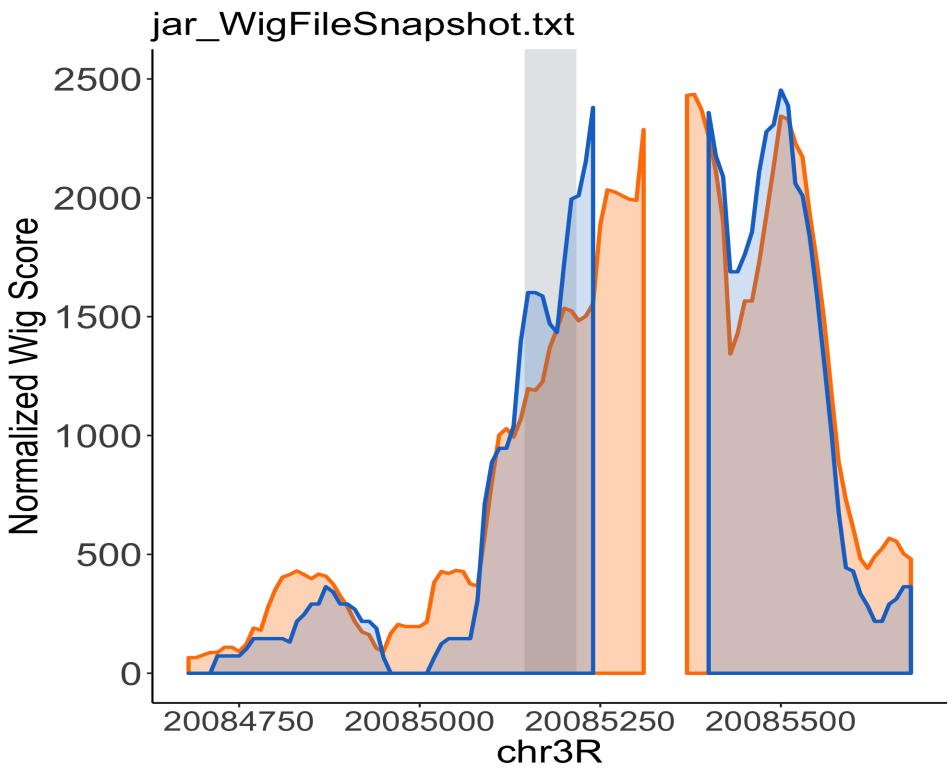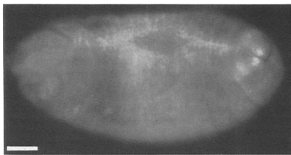

Location: Dorsal Type: Promoter ZScore: -0.487350229 PValue: 0.626010158

Supplement: S3 File — Reports consist of in situ hybridization images, ATAC-seq traces, and calculated p-value and Z Score for each region used in the final analysis. (ZIP) [file pgen.1007367.s015.zip › S3_File/jar_Report.pdf]

king-tubby

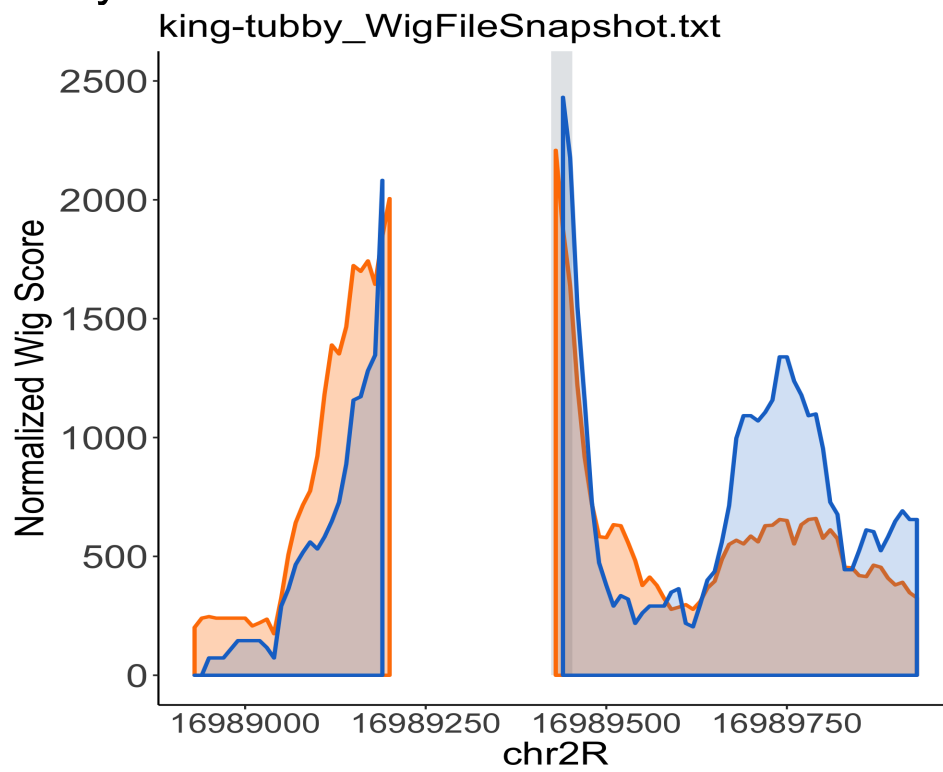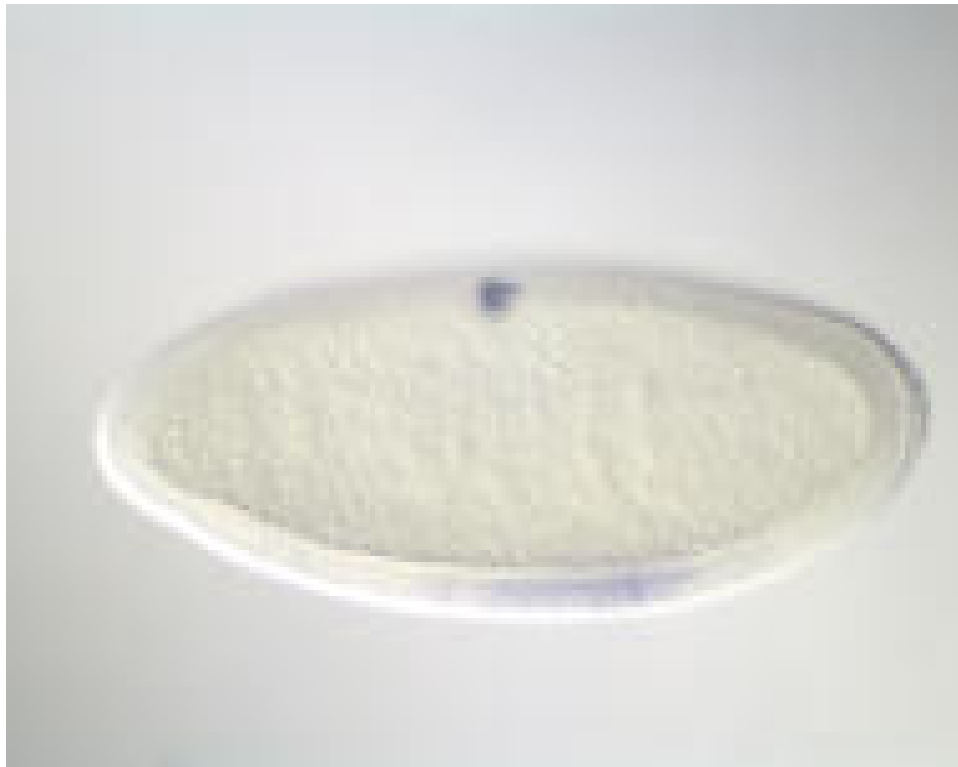

Location: Ventral Type: Promoter ZScore: -0.659352422 PValue: 0.509669487

Supplement: S3 File — Reports consist of in situ hybridization images, ATAC-seq traces, and calculated p-value and Z Score for each region used in the final analysis. (ZIP) [file pgen.1007367.s015.zip › S3_File/king-tubby_Report.pdf]

## kni\_-5\_construct\_71

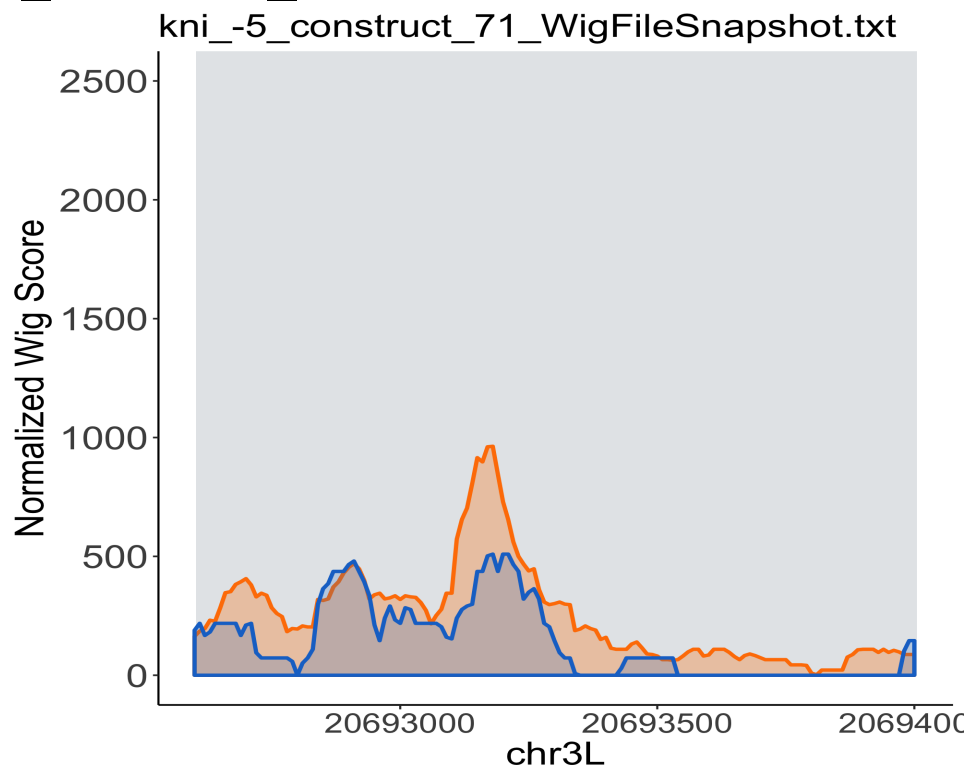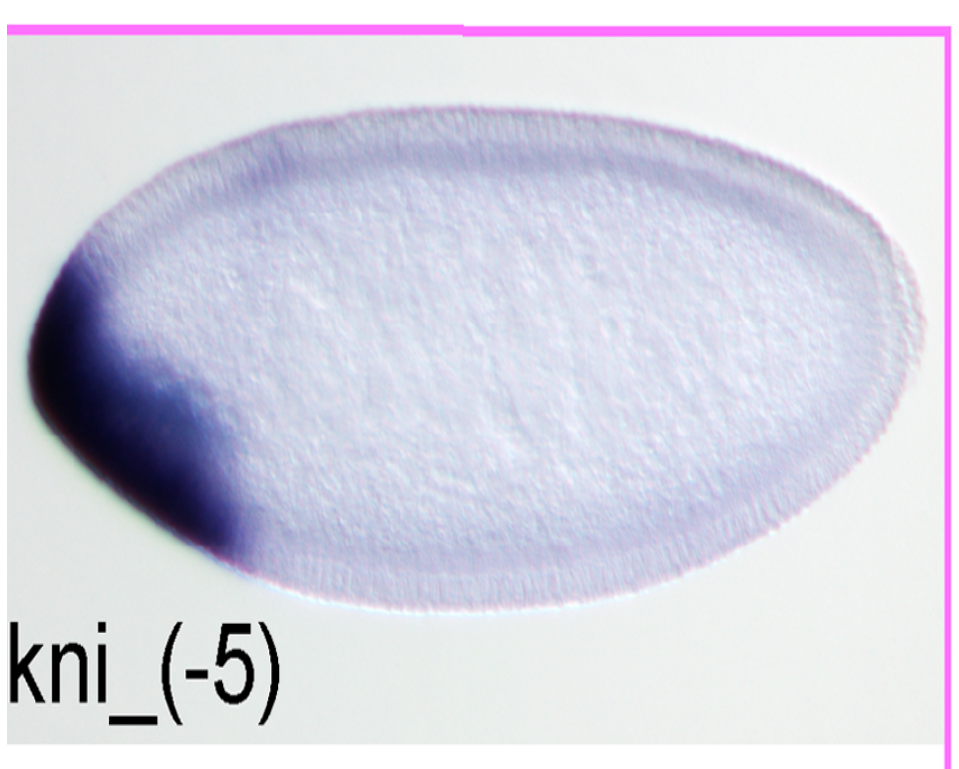

Location: Anterior Type: Enhancer ZScore: 1.224633192 PValue: 0.220713456

Supplement: S3 File — Reports consist of in situ hybridization images, ATAC-seq traces, and calculated p-value and Z Score for each region used in the final analysis. (ZIP) [file pgen.1007367.s015.zip › S3_File/kni_-5_construct_71_Report.pdf]
