# Supplementary material for: Patterns of chromatin accessibility along the anterior-posterior axis in the early Drosophila embryo
Source: PLoS Genet. 2018 May 4;14(5):e1007367. doi: 10.1371/journal.pgen.1007367 (PMC5955596; doi:10.1371/journal.pgen.1007367)
Supplement: S3 File — Reports consist of in situ hybridization images, ATAC-seq traces, and calculated p-value and Z Score for each region used in the final analysis. (ZIP) [file pgen.1007367.s015.zip › S3_File/eve_stripe1-construct1(65)_Report.pdf]

eve_stripe1-construct1(65)

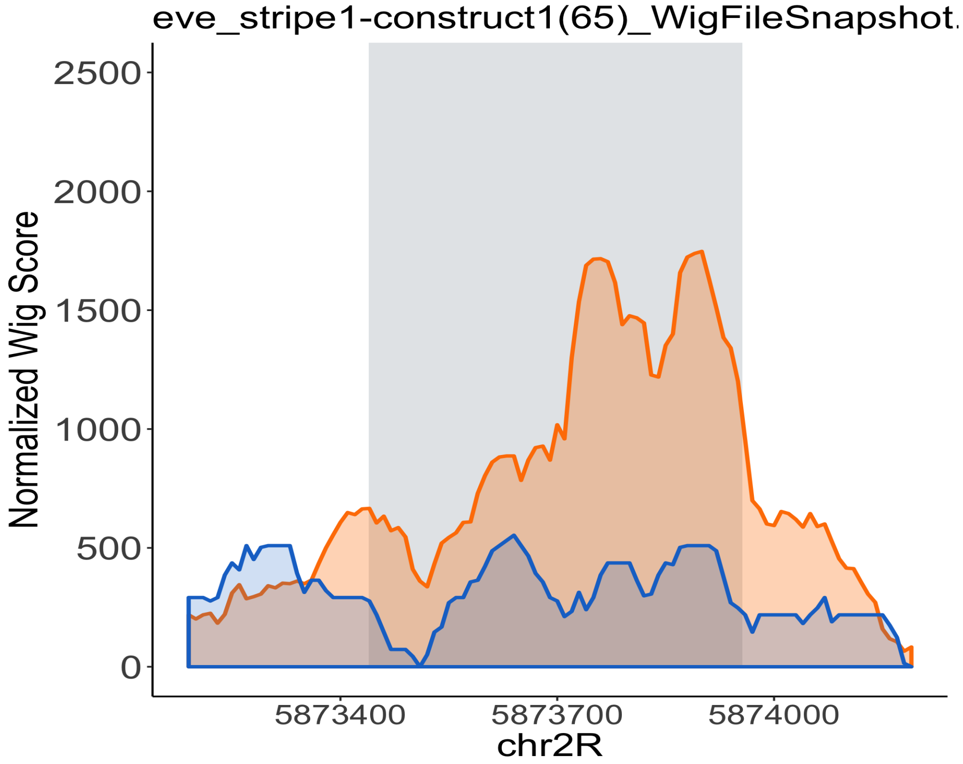

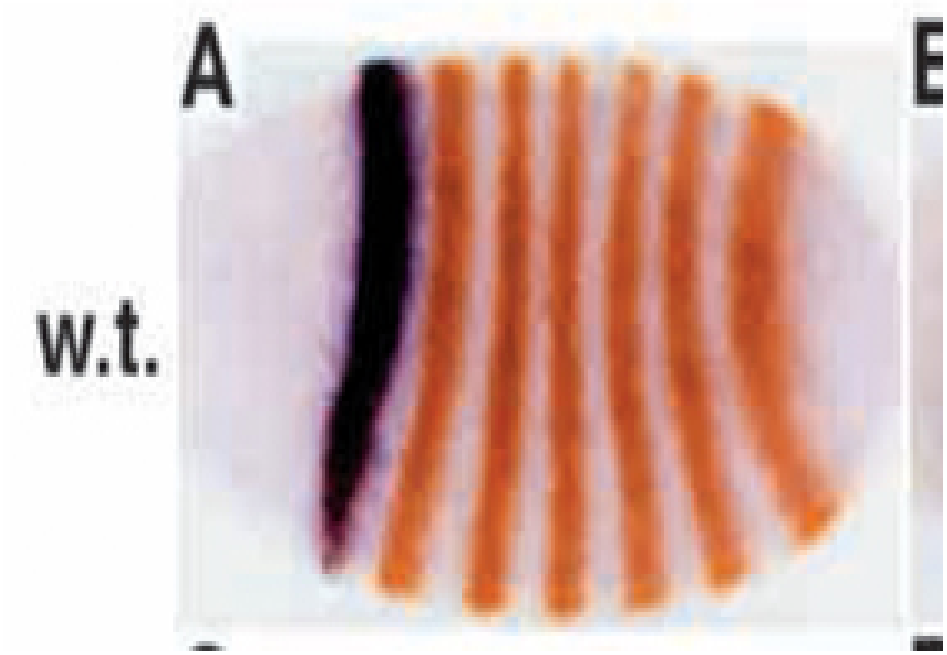

Location: Anterior Type: Enhancer ZScore: 2.36975532 PValue: 0.017799861
