# Supplementary figures and images for: Patterns of chromatin accessibility along the anterior-posterior axis in the early Drosophila embryo (part 2 of 2)
Source: PLoS Genet. 2018 May 4;14(5):e1007367. doi: 10.1371/journal.pgen.1007367 (PMC5955596; doi:10.1371/journal.pgen.1007367)

kni\_KD\_30

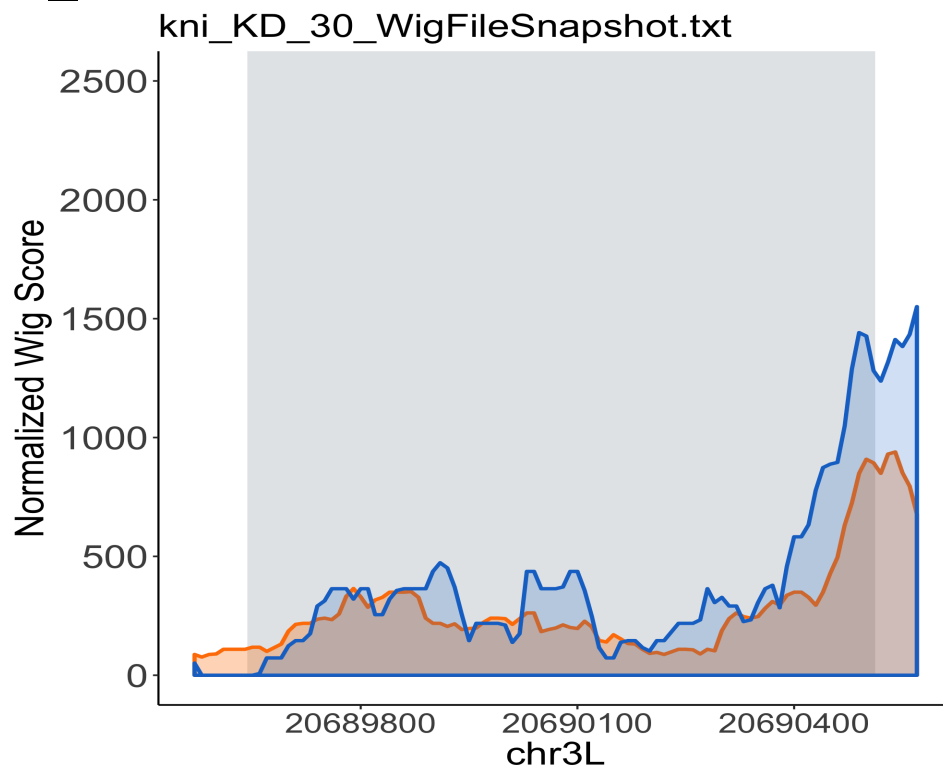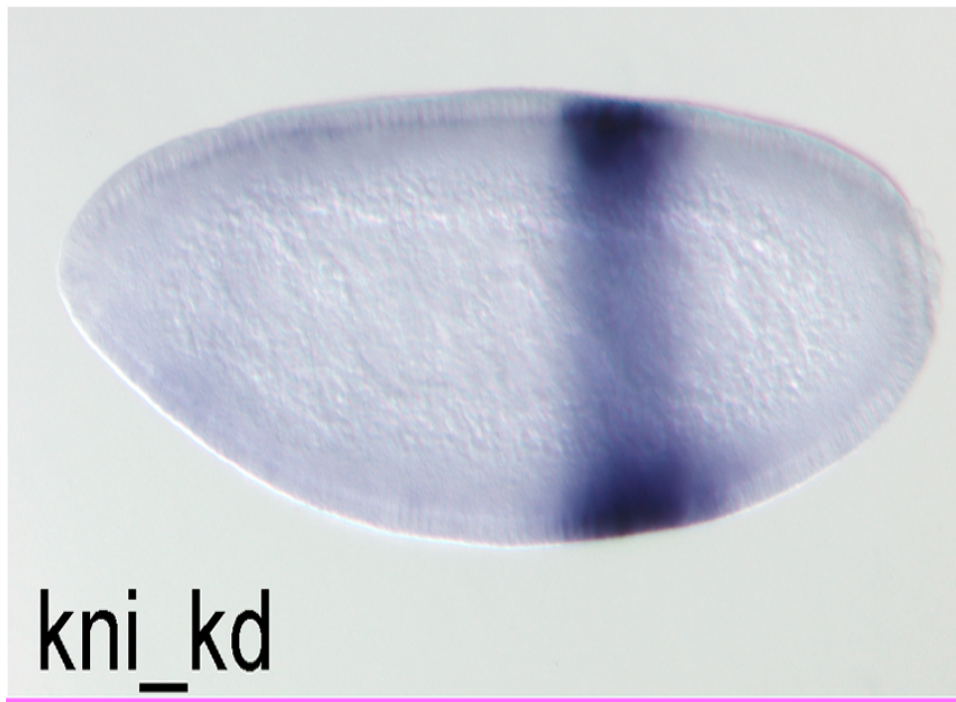

Location: Posterior Type: Enhancer ZScore: 0.697814413 PValue: 0.485293264

Supplement: S3 File — Reports consist of in situ hybridization images, ATAC-seq traces, and calculated p-value and Z Score for each region used in the final analysis. (ZIP) [file pgen.1007367.s015.zip › S3_File/kni_KD_30_Report.pdf]

La

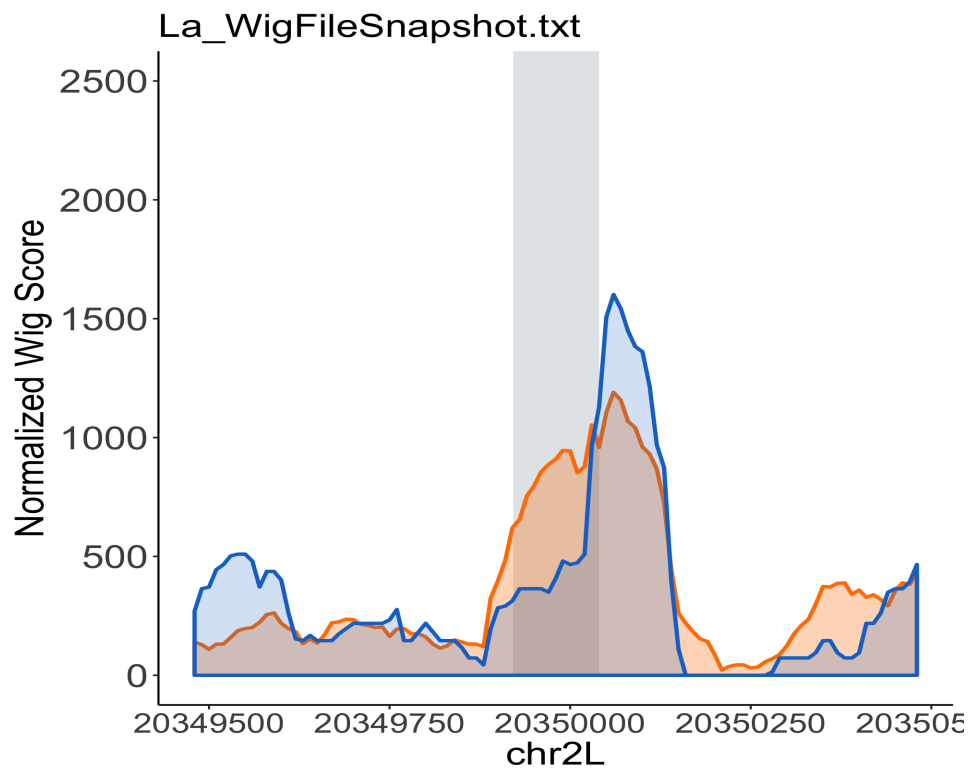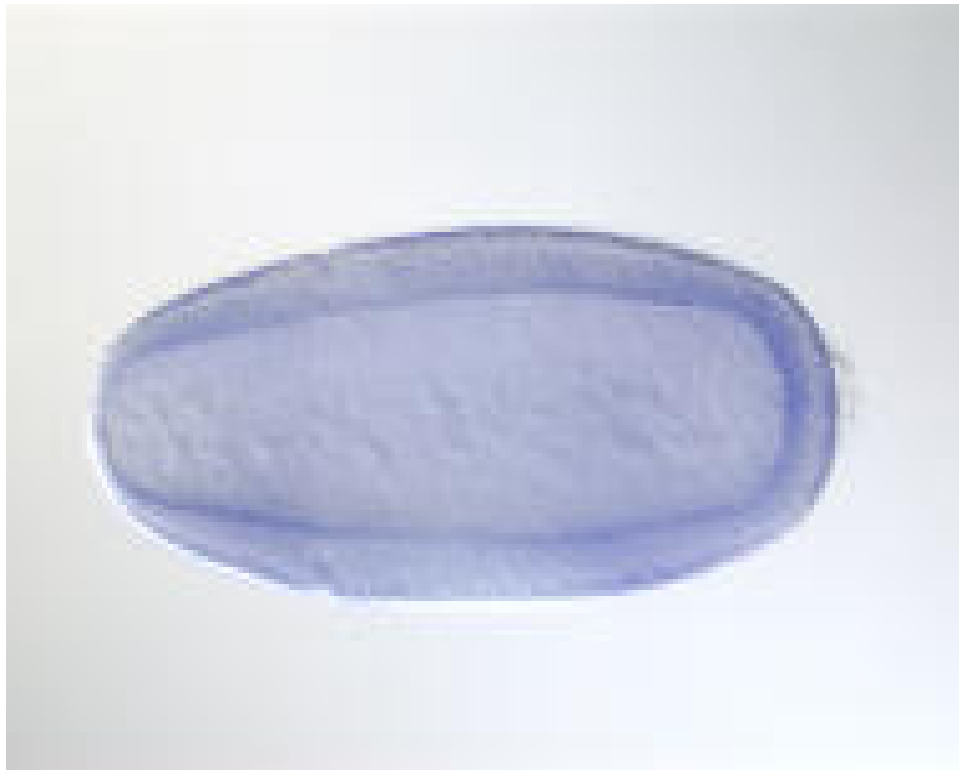

Location: Mostly Post Type: Promoter ZScore: -1.230155313 PValue: 0.21863895

Supplement: S3 File — Reports consist of in situ hybridization images, ATAC-seq traces, and calculated p-value and Z Score for each region used in the final analysis. (ZIP) [file pgen.1007367.s015.zip › S3_File/La_Report.pdf]

m4

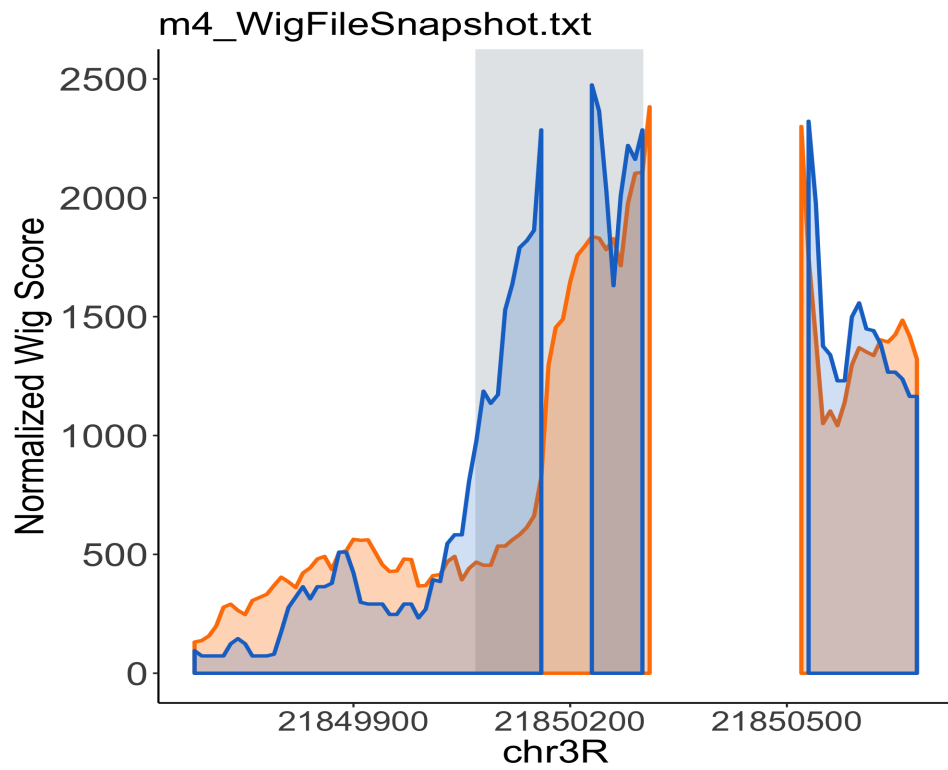

A

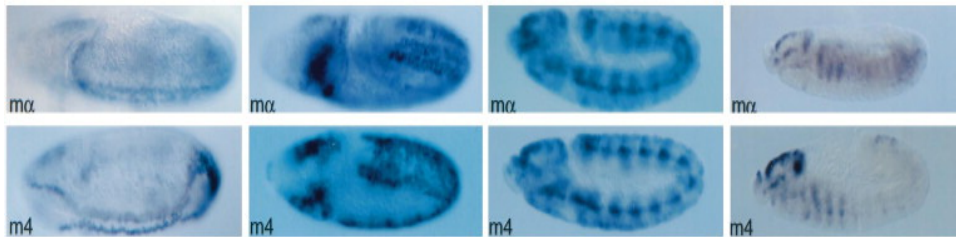

B

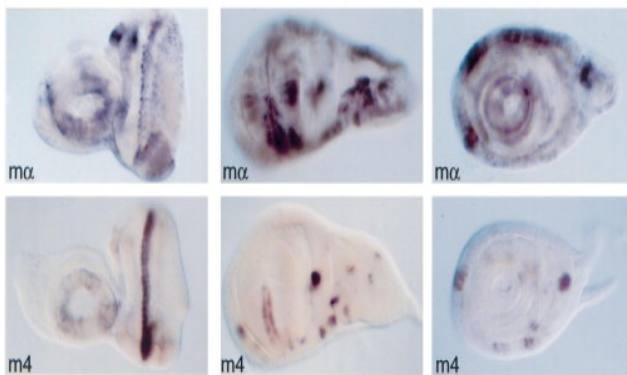

Location: Dorsal Type: Promoter ZScore: -1.12328896 PValue: 0.261314791

Supplement: S3 File — Reports consist of in situ hybridization images, ATAC-seq traces, and calculated p-value and Z Score for each region used in the final analysis. (ZIP) [file pgen.1007367.s015.zip › S3_File/m4_Report.pdf]

## Mcm2

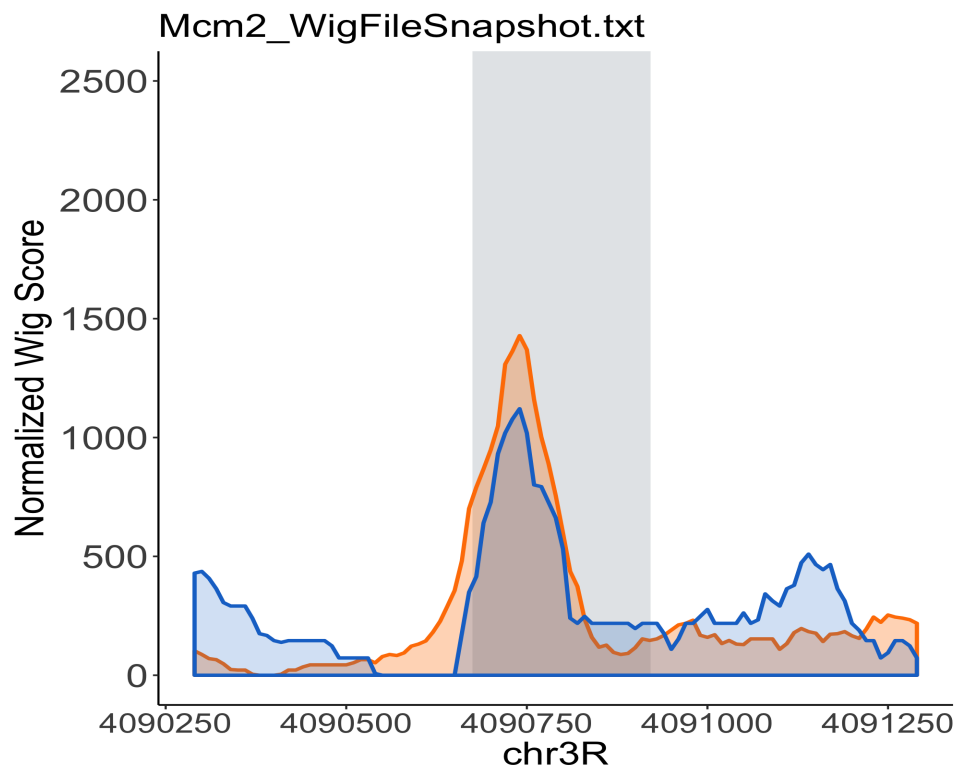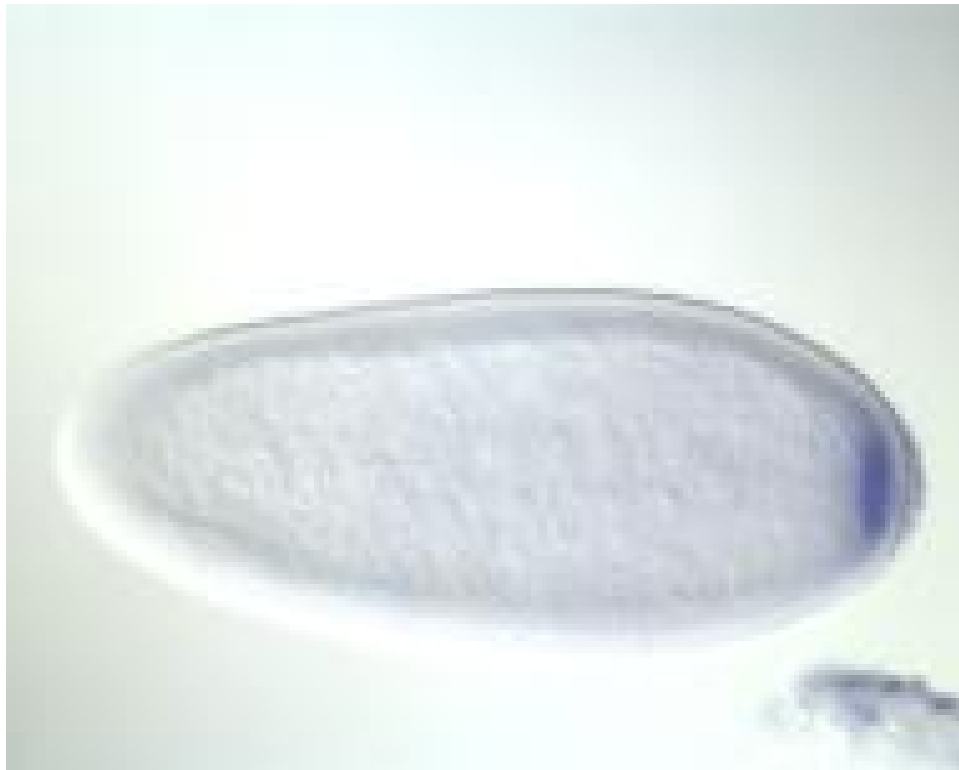

Location: Posterior Type: Promoter ZScore: -0.492333061 PValue: 0.622483912

Supplement: S3 File — Reports consist of in situ hybridization images, ATAC-seq traces, and calculated p-value and Z Score for each region used in the final analysis. (ZIP) [file pgen.1007367.s015.zip › S3_File/Mcm2_Report.pdf]

## Mdr49

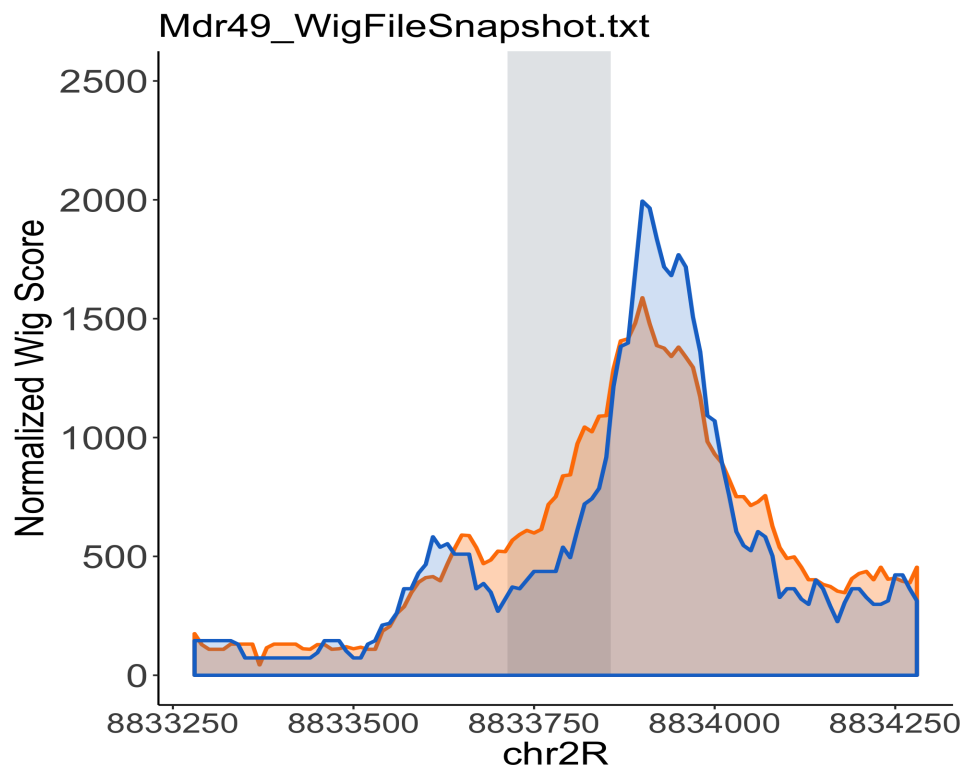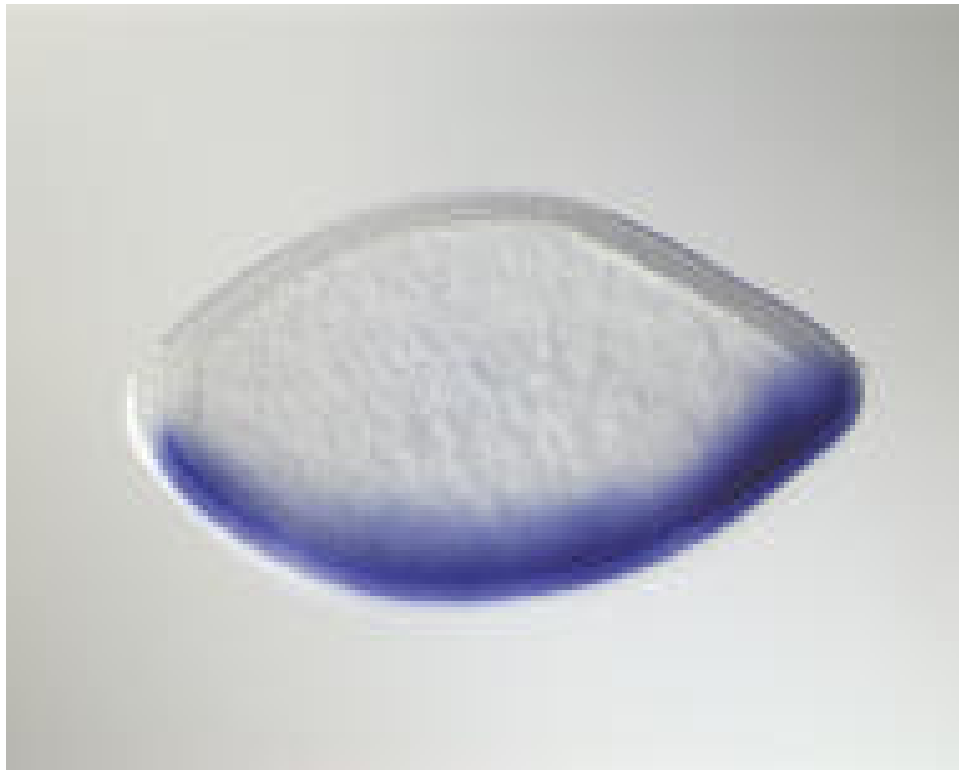

Location: Ventral Type: Promoter ZScore: 0.84670263 PValue: 0.397160893

Supplement: S3 File — Reports consist of in situ hybridization images, ATAC-seq traces, and calculated p-value and Z Score for each region used in the final analysis. (ZIP) [file pgen.1007367.s015.zip › S3_File/Mdr49_Report.pdf]

## Mdr49\_Zeitlinger

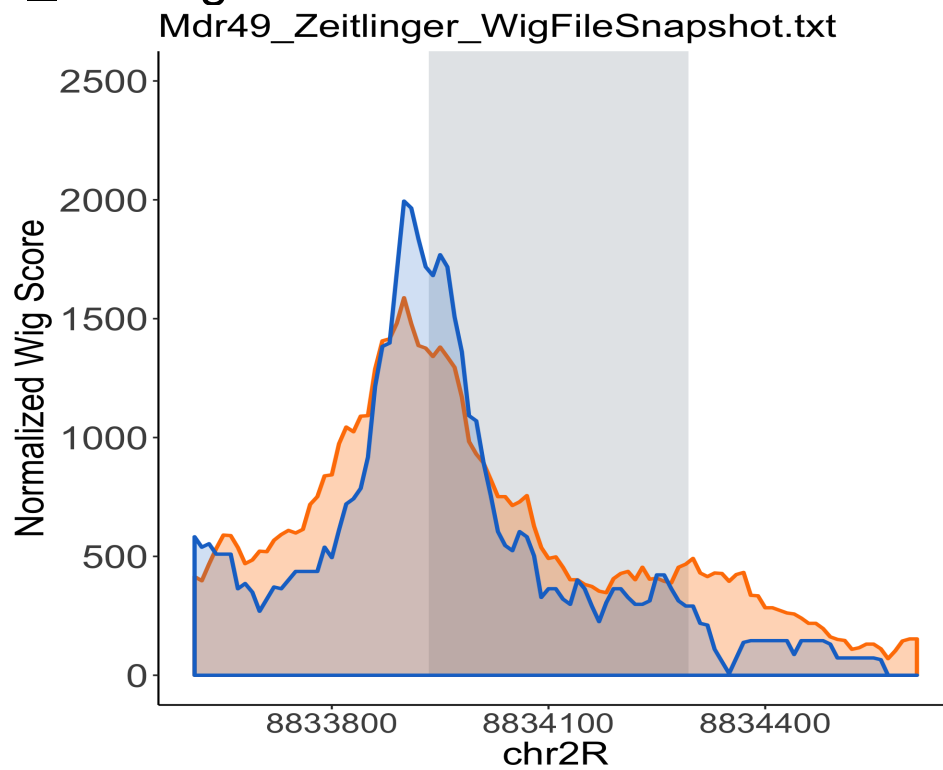

**Fig. S3: *Mdr49* enhancer validation**

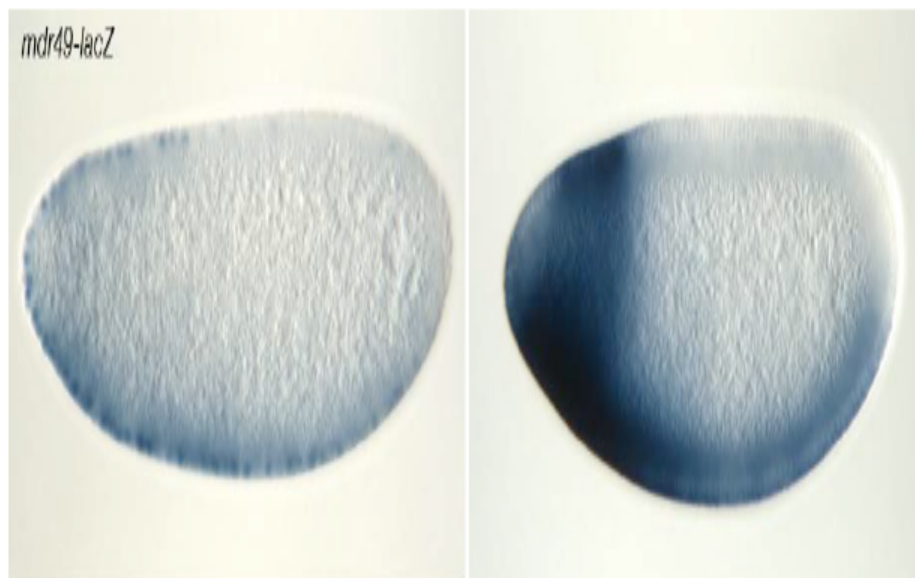

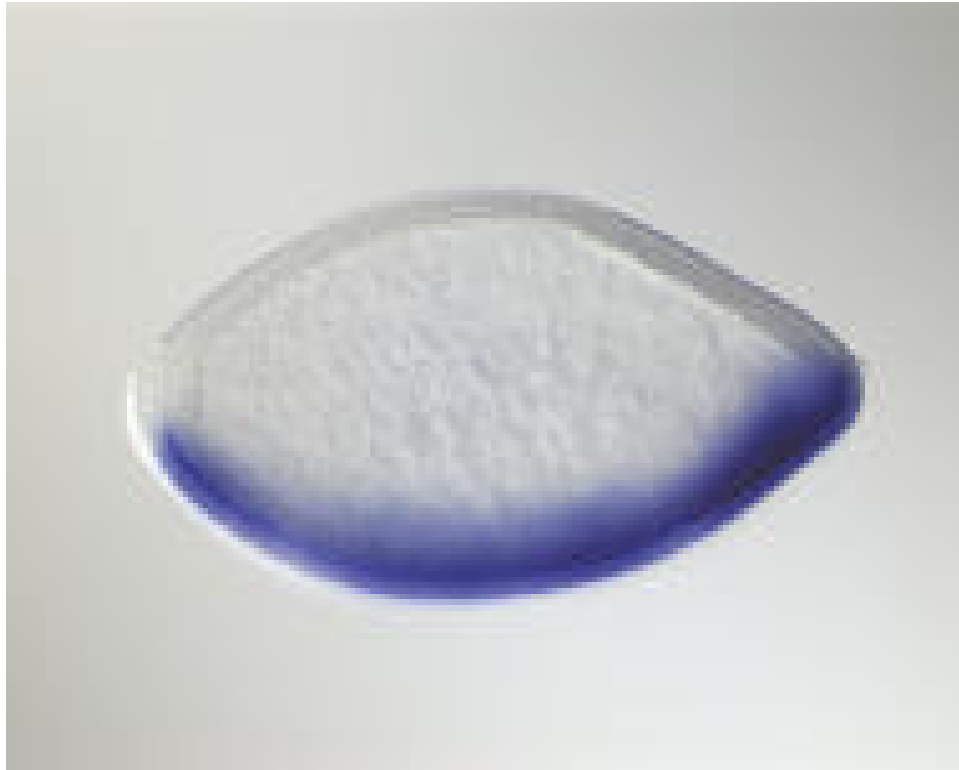

Location: Ventral Type: Enhancer ZScore: 0.028276225 PValue: 0.977441843

Supplement: S3 File — Reports consist of in situ hybridization images, ATAC-seq traces, and calculated p-value and Z Score for each region used in the final analysis. (ZIP) [file pgen.1007367.s015.zip › S3_File/Mdr49_Zeitlinger_Report.pdf]

## Mef2\_Ozdemir

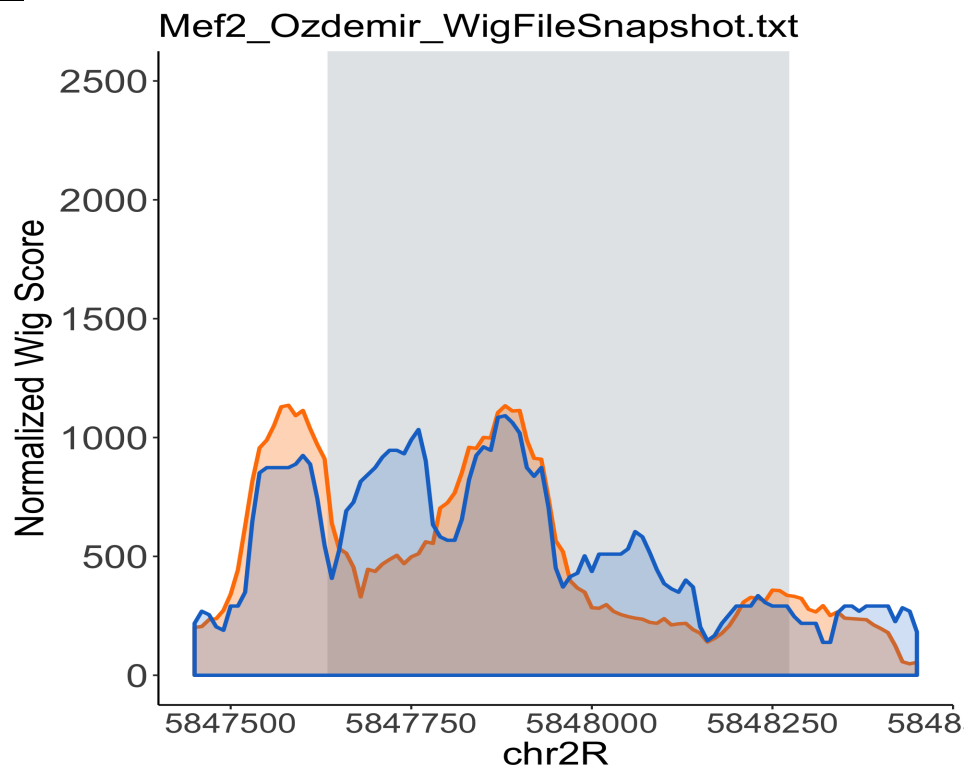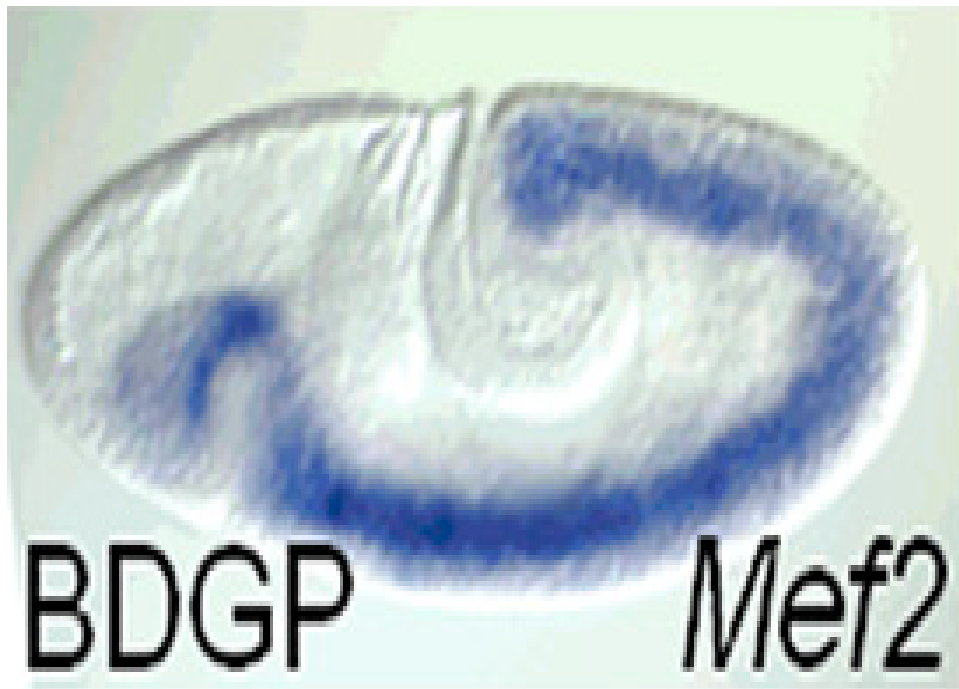

Location: Ventral Type: Enhancer ZScore: -0.451573238 PValue: 0.651576453

Supplement: S3 File — Reports consist of in situ hybridization images, ATAC-seq traces, and calculated p-value and Z Score for each region used in the final analysis. (ZIP) [file pgen.1007367.s015.zip › S3_File/Mef2_Ozdemir_Report.pdf]

Men

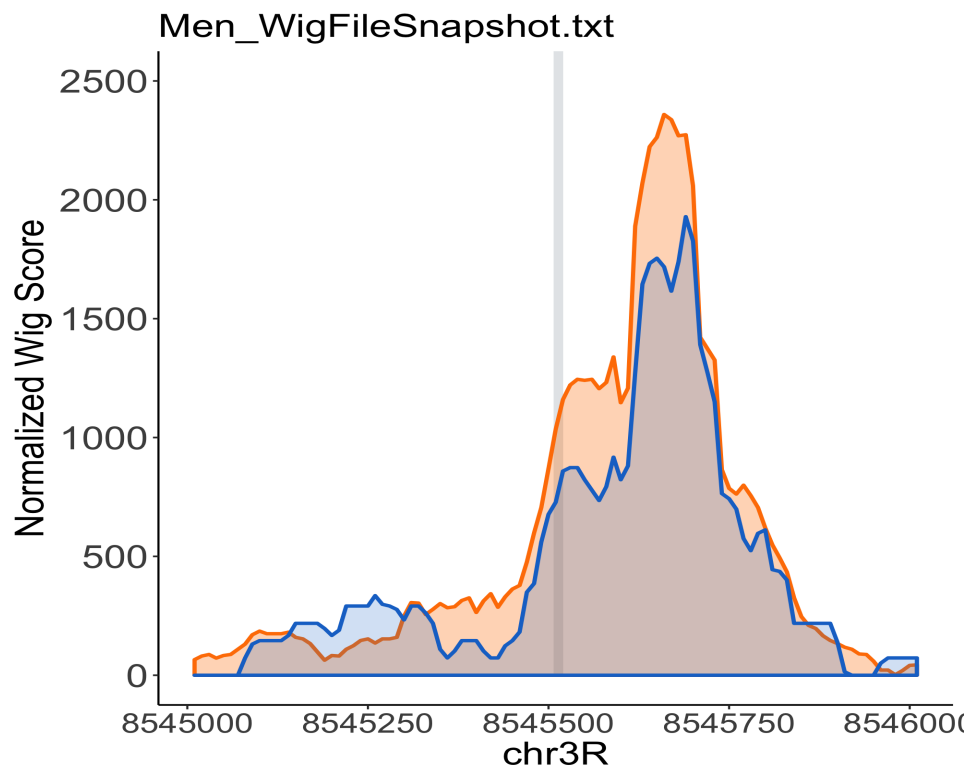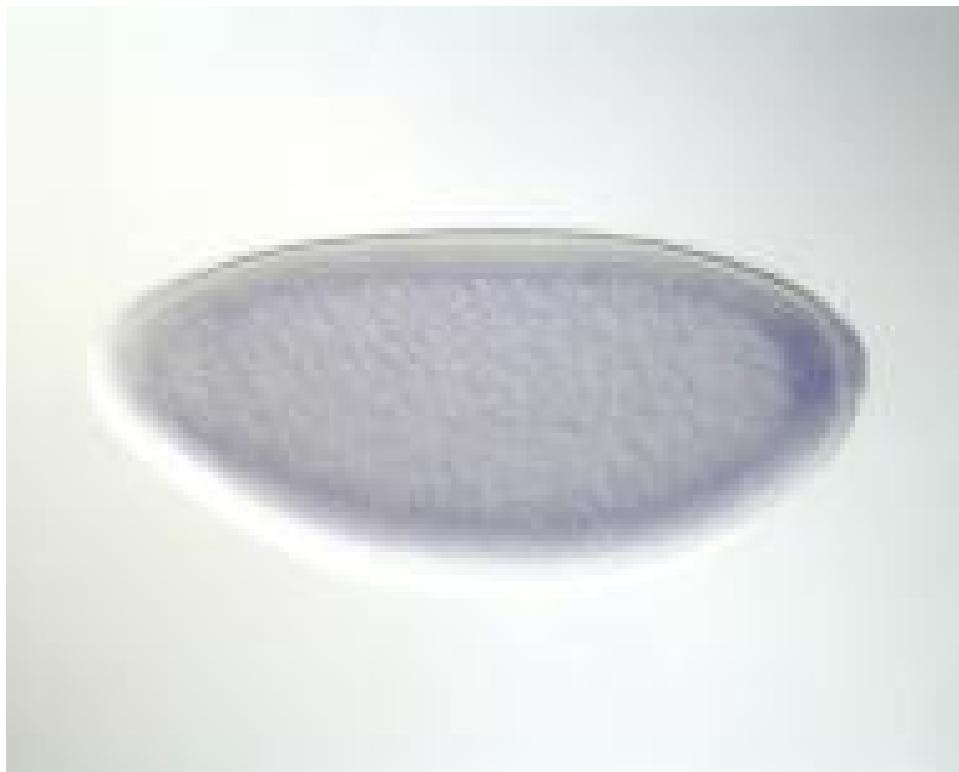

Location: Posterior Type: Promoter ZScore: -0.735063683 PValue: 0.462300711

Supplement: S3 File — Reports consist of in situ hybridization images, ATAC-seq traces, and calculated p-value and Z Score for each region used in the final analysis. (ZIP) [file pgen.1007367.s015.zip › S3_File/Men_Report.pdf]

# Mes2

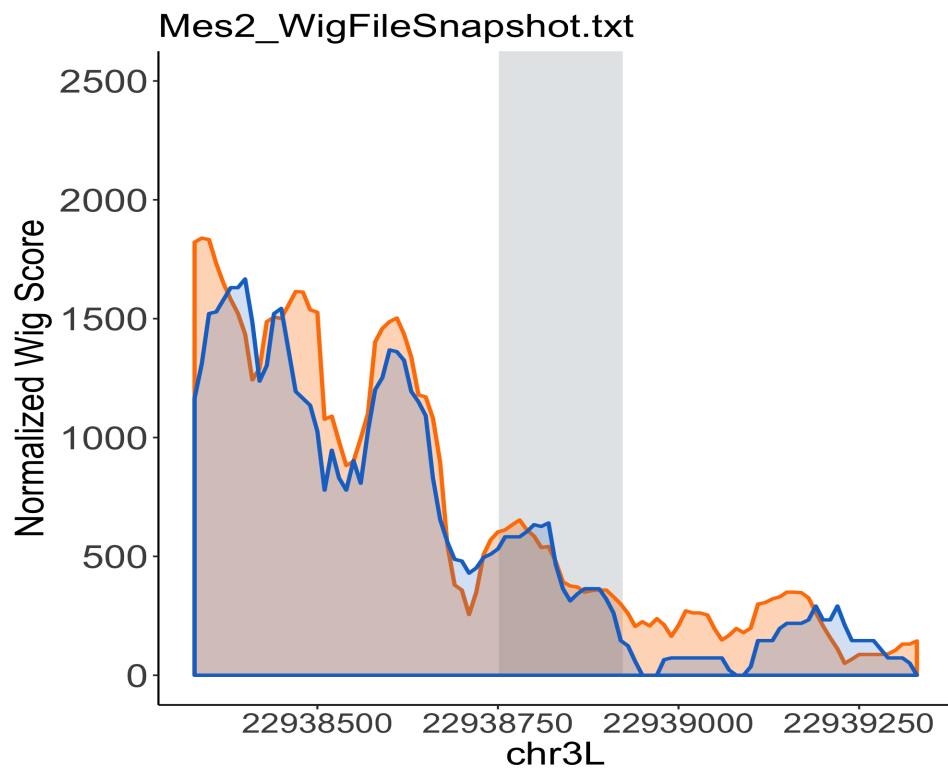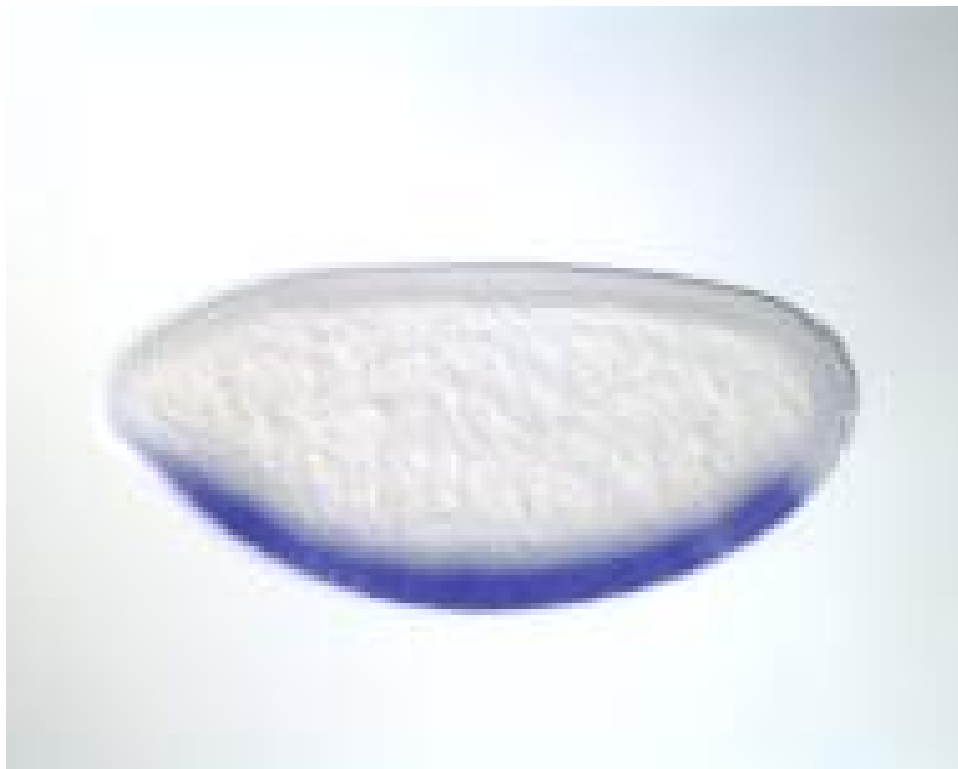

Location: Ventral Type: Promoter ZScore: 0.054613969 PValue: 0.95644601

Supplement: S3 File — Reports consist of in situ hybridization images, ATAC-seq traces, and calculated p-value and Z Score for each region used in the final analysis. (ZIP) [file pgen.1007367.s015.zip › S3_File/Mes2_Report.pdf]

## mir-1\_Biemar

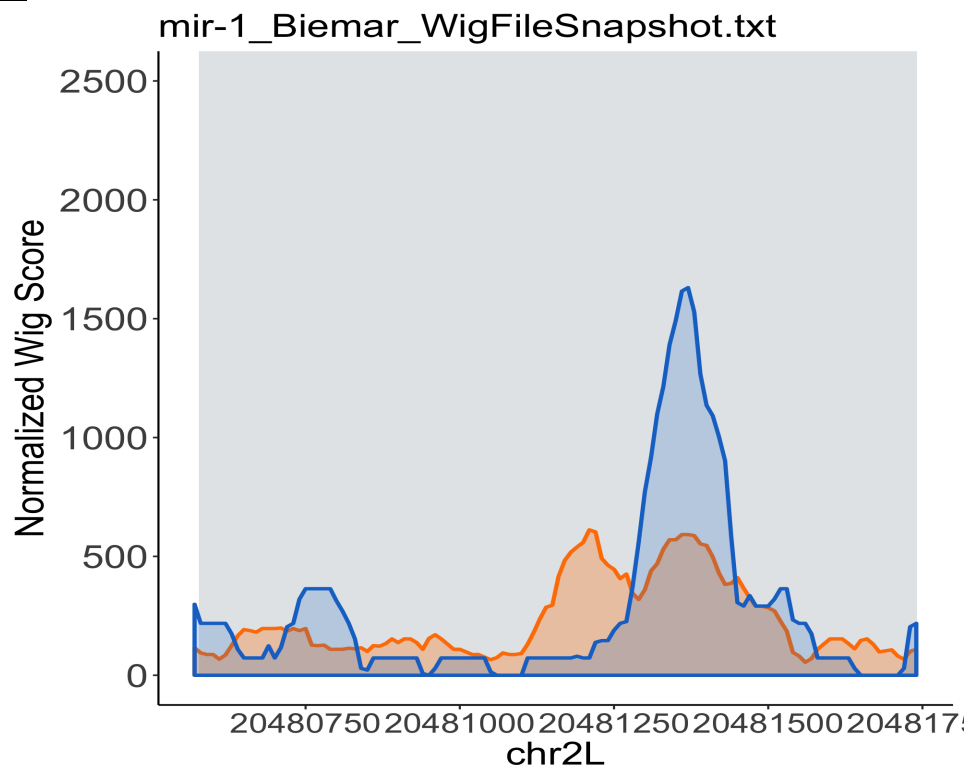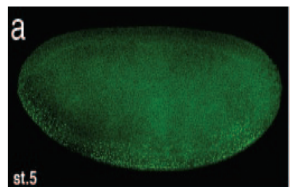

Location: Ventral Type: Enhancer ZScore: -0.422197492 PValue: 0.672880872

Supplement: S3 File — Reports consist of in situ hybridization images, ATAC-seq traces, and calculated p-value and Z Score for each region used in the final analysis. (ZIP) [file pgen.1007367.s015.zip › S3_File/mir-1_Biemar_Report.pdf]

mmps

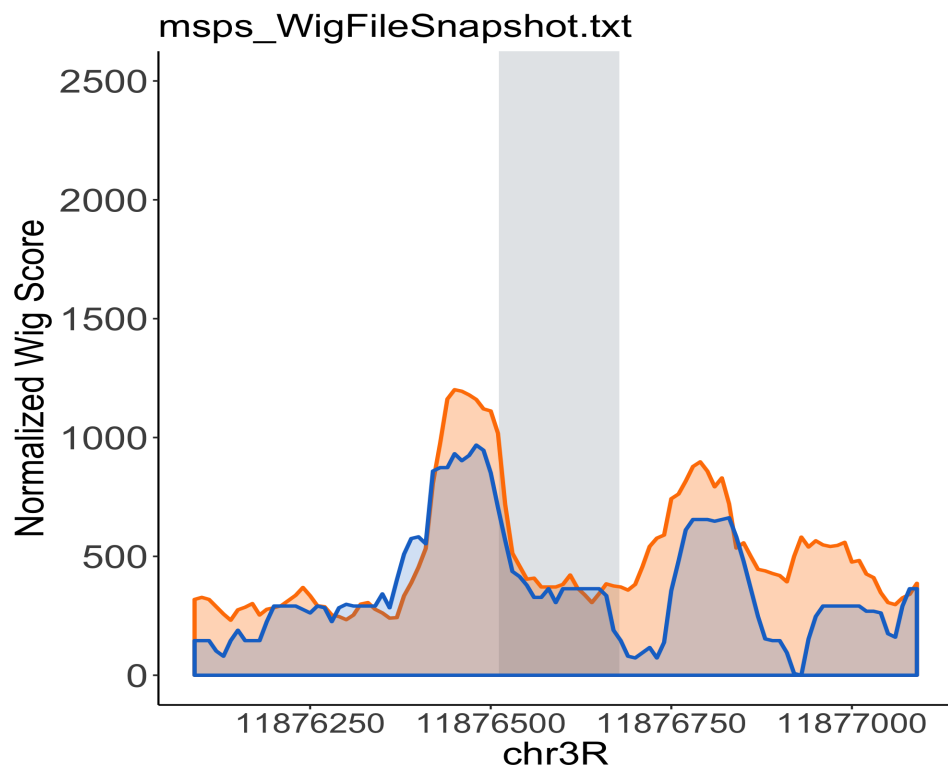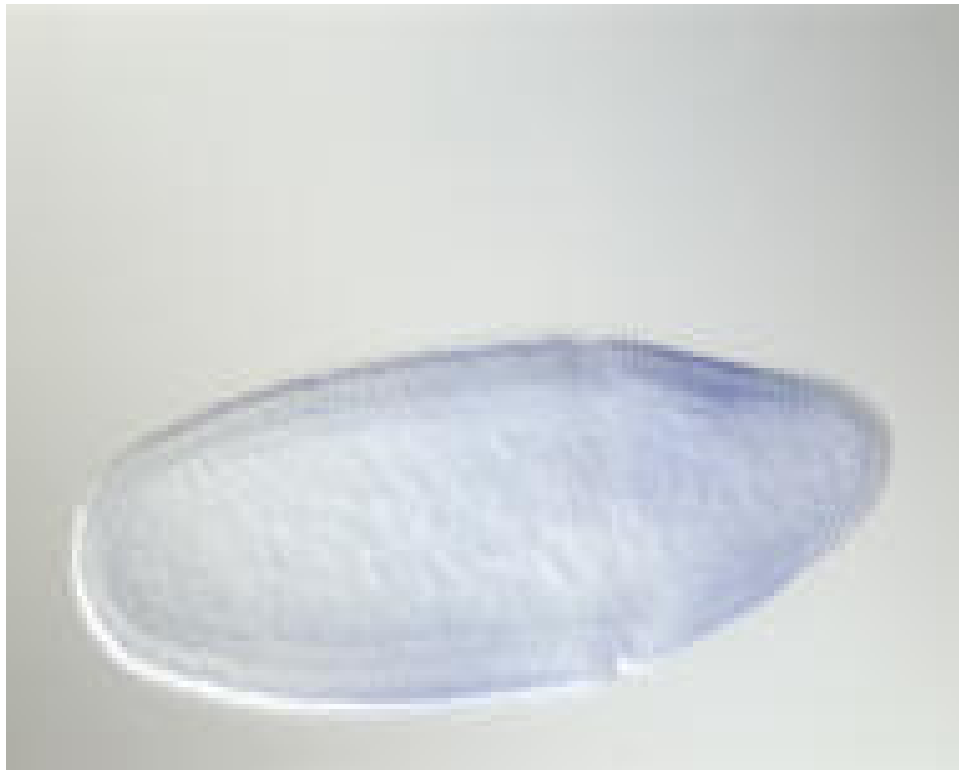

Location: Mostly Ant Type: Promoter ZScore: 0.286082286 PValue: 0.774815098

Supplement: S3 File — Reports consist of in situ hybridization images, ATAC-seq traces, and calculated p-value and Z Score for each region used in the final analysis. (ZIP) [file pgen.1007367.s015.zip › S3_File/msps_Report.pdf]

net

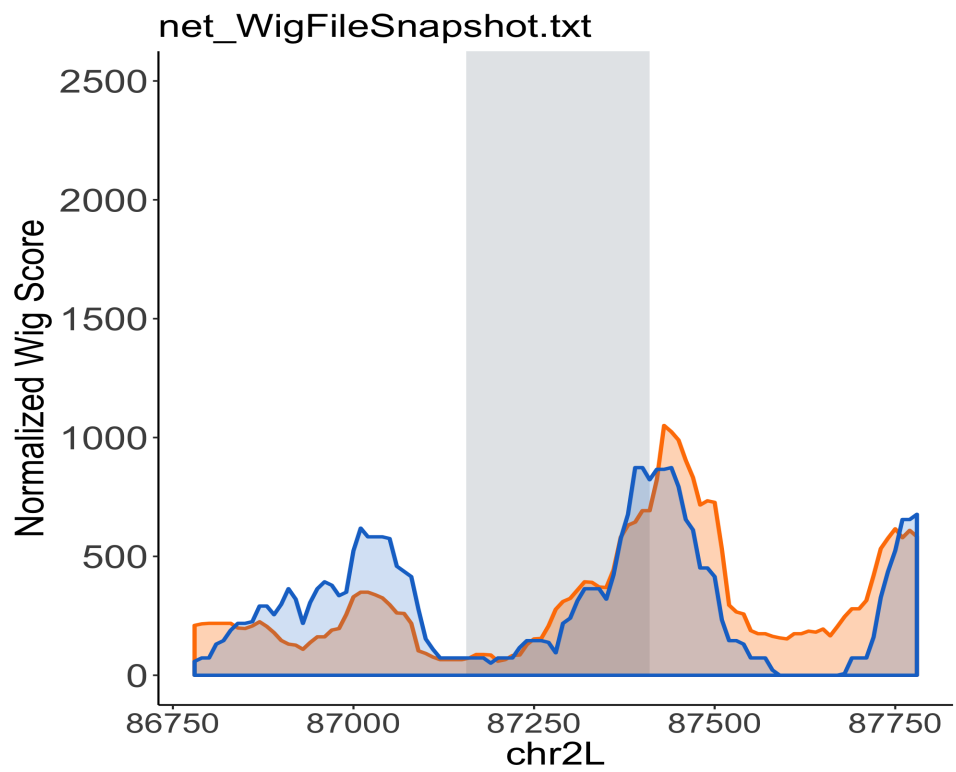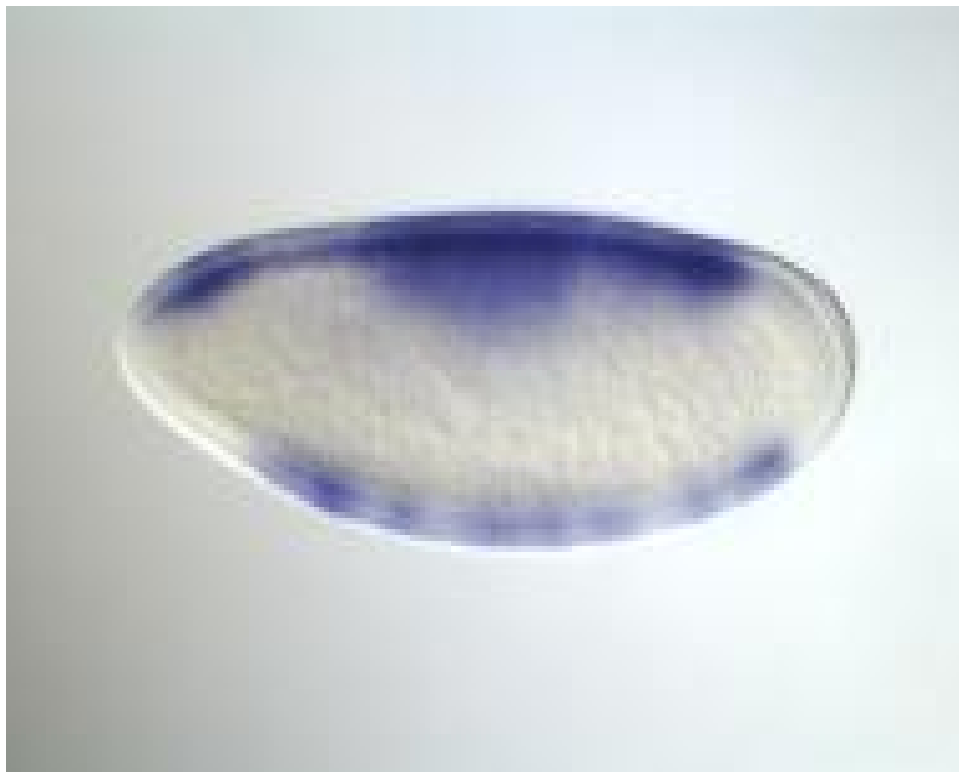

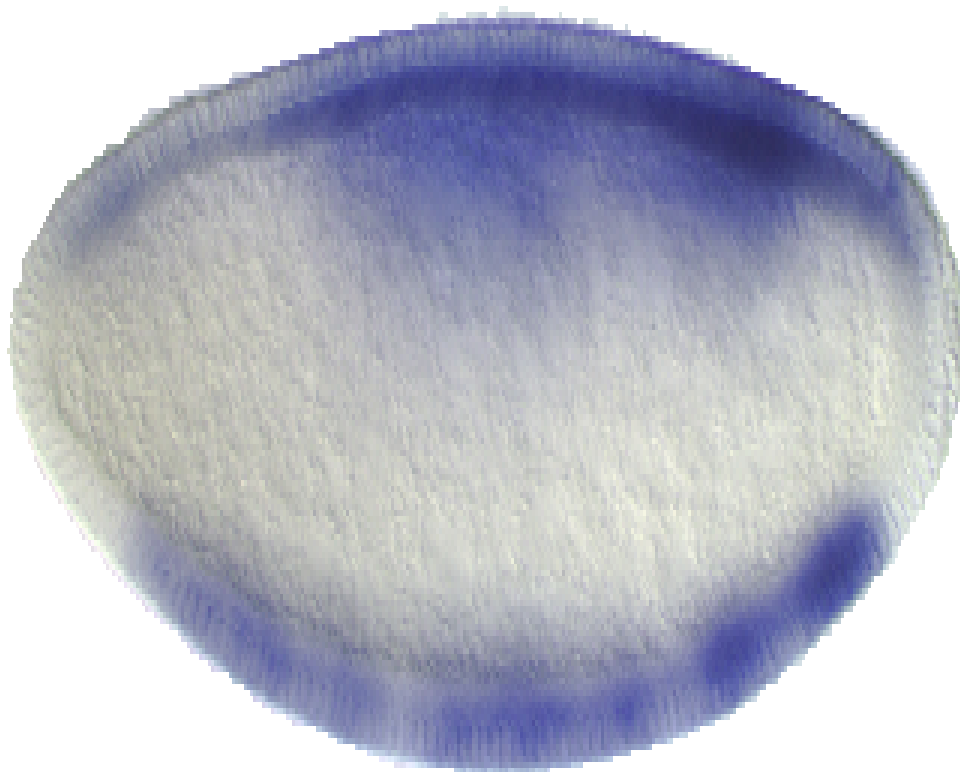

Location: Dorsal Type: Promoter ZScore: -0.033725149 PValue: 0.973096324

Supplement: S3 File — Reports consist of in situ hybridization images, ATAC-seq traces, and calculated p-value and Z Score for each region used in the final analysis. (ZIP) [file pgen.1007367.s015.zip › S3_File/net_Report.pdf]

# NetA

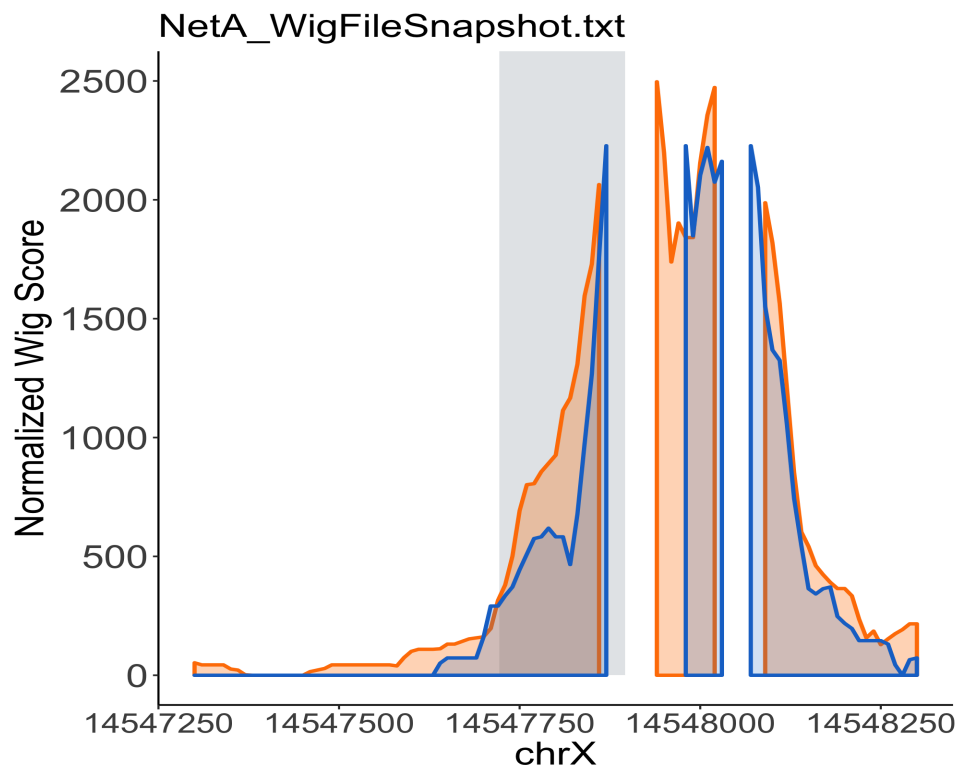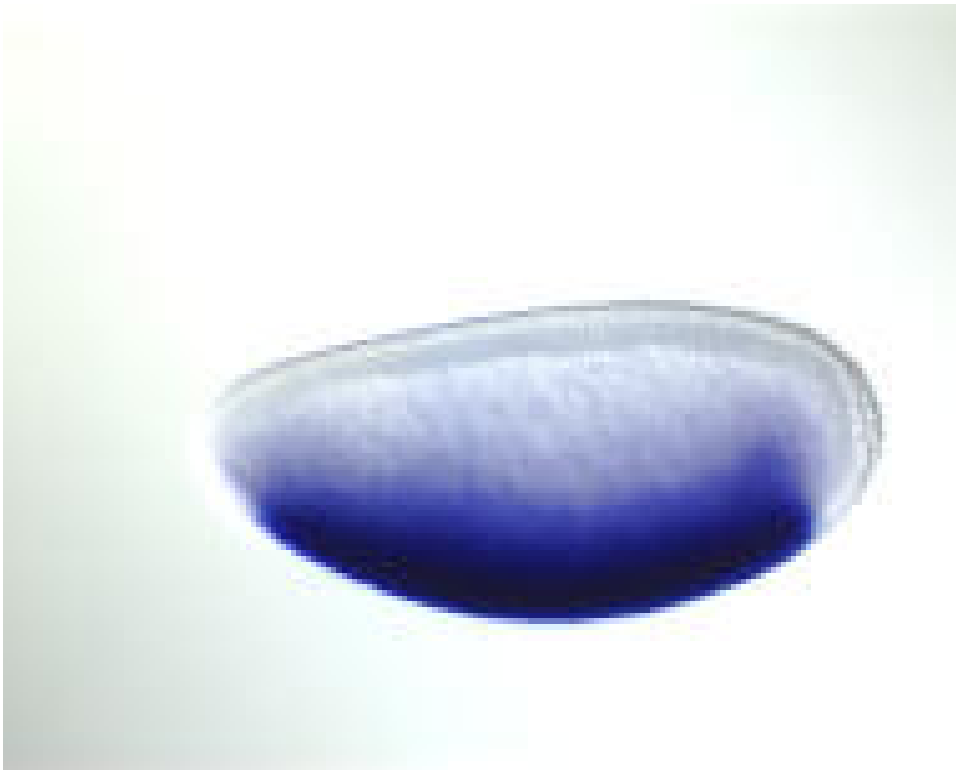

Location: Ventral Type: Promoter ZScore: 0.555421981 PValue: 0.578606061

Supplement: S3 File — Reports consist of in situ hybridization images, ATAC-seq traces, and calculated p-value and Z Score for each region used in the final analysis. (ZIP) [file pgen.1007367.s015.zip › S3_File/NetA_Report.pdf]

noc

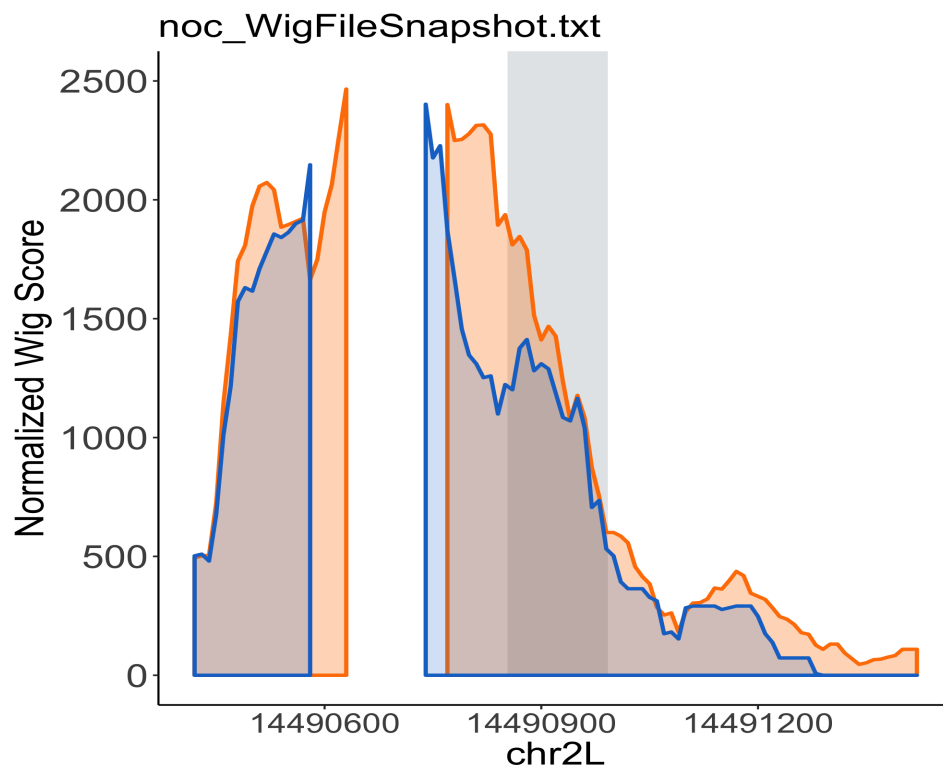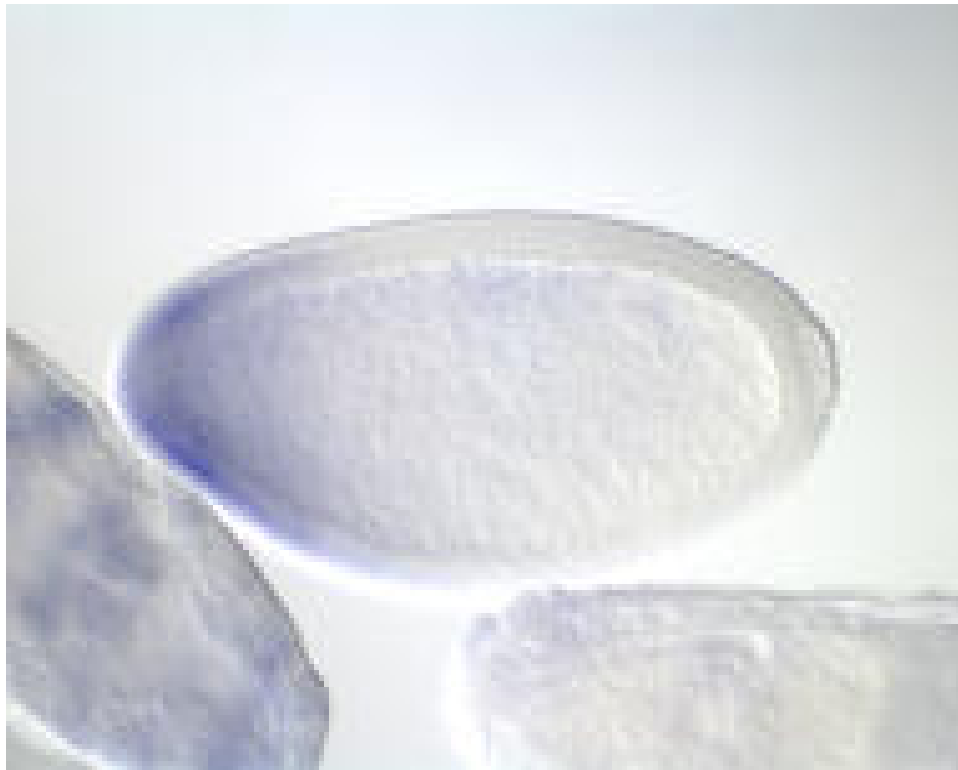

Location: Anterior Type: Promoter ZScore: 0.379763579 PValue: 0.70412092

Supplement: S3 File — Reports consist of in situ hybridization images, ATAC-seq traces, and calculated p-value and Z Score for each region used in the final analysis. (ZIP) [file pgen.1007367.s015.zip › S3_File/noc_Report.pdf]

# Notum

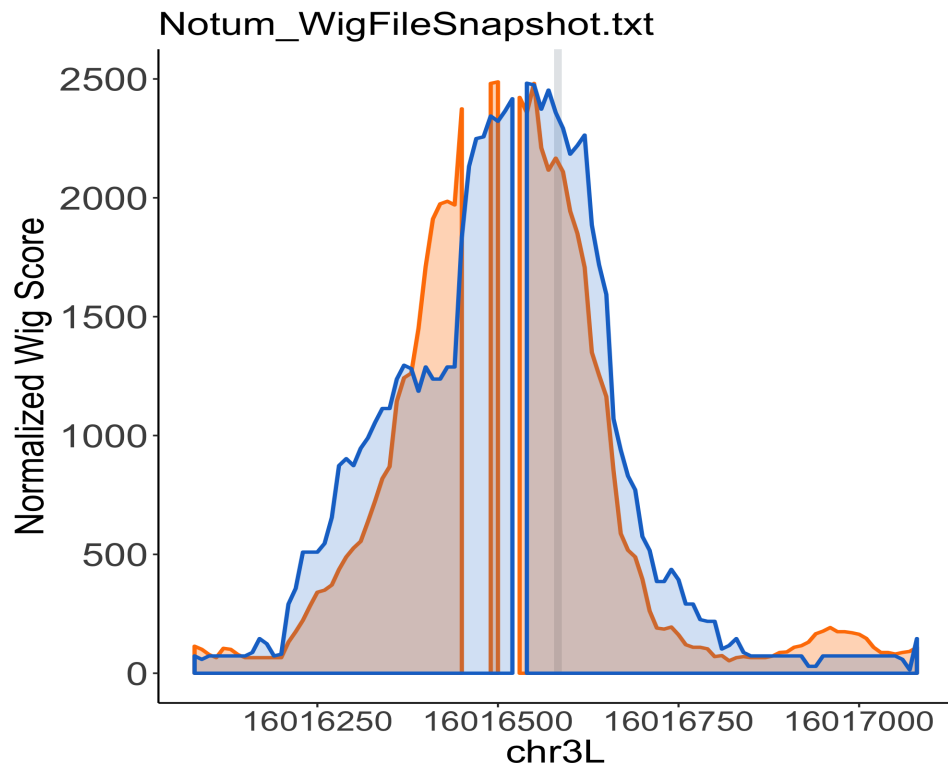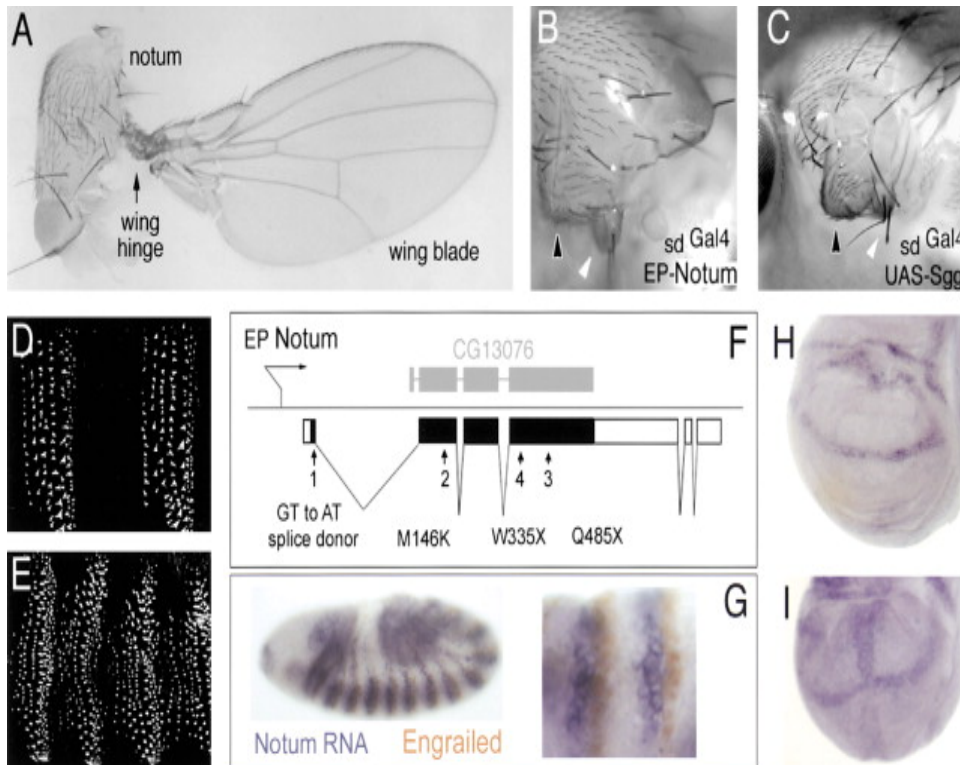

Location: Ventral Type: Promoter ZScore: -0.31188683 PValue: 0.755126532

Supplement: S3 File — Reports consist of in situ hybridization images, ATAC-seq traces, and calculated p-value and Z Score for each region used in the final analysis. (ZIP) [file pgen.1007367.s015.zip › S3_File/Notum_Report.pdf]

nub\_-2\_construct(50-30)

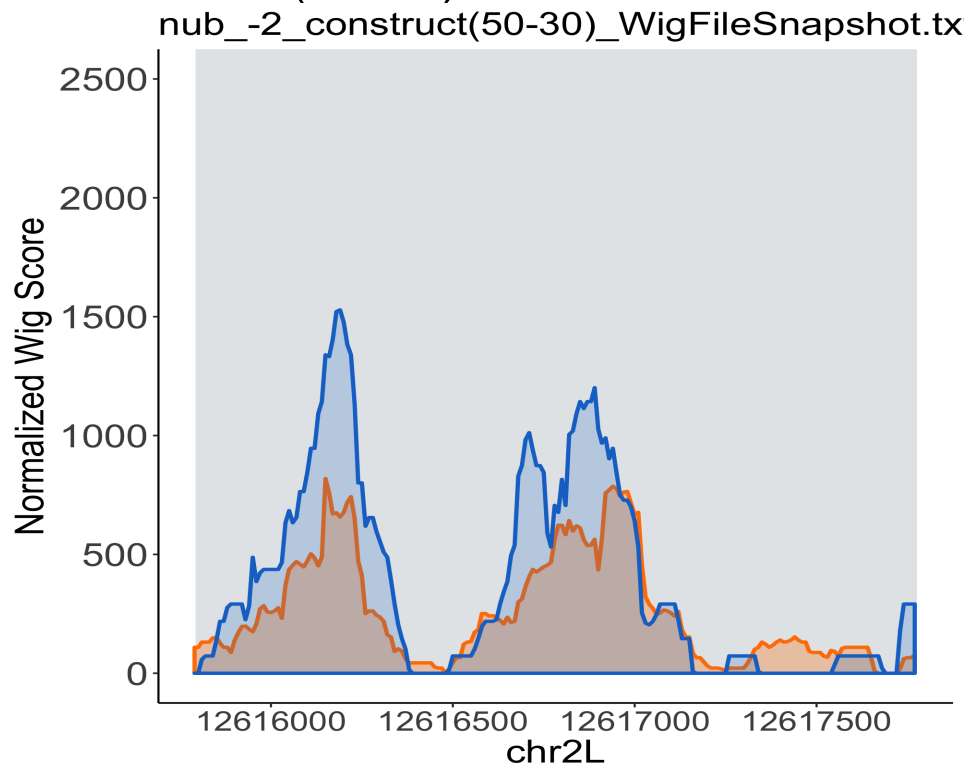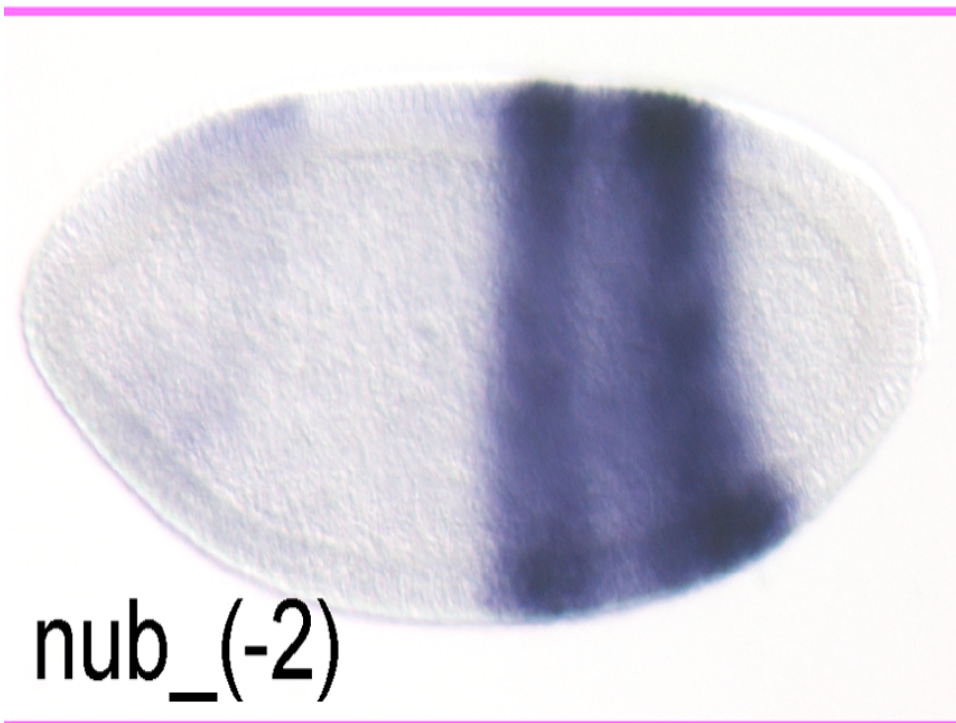

Location: Posterior Type: Enhancer ZScore: 0.864286043 PValue: 0.387430775

Supplement: S3 File — Reports consist of in situ hybridization images, ATAC-seq traces, and calculated p-value and Z Score for each region used in the final analysis. (ZIP) [file pgen.1007367.s015.zip › S3_File/nub_-2_construct(50-30)_Report.pdf]

# Oatp74D

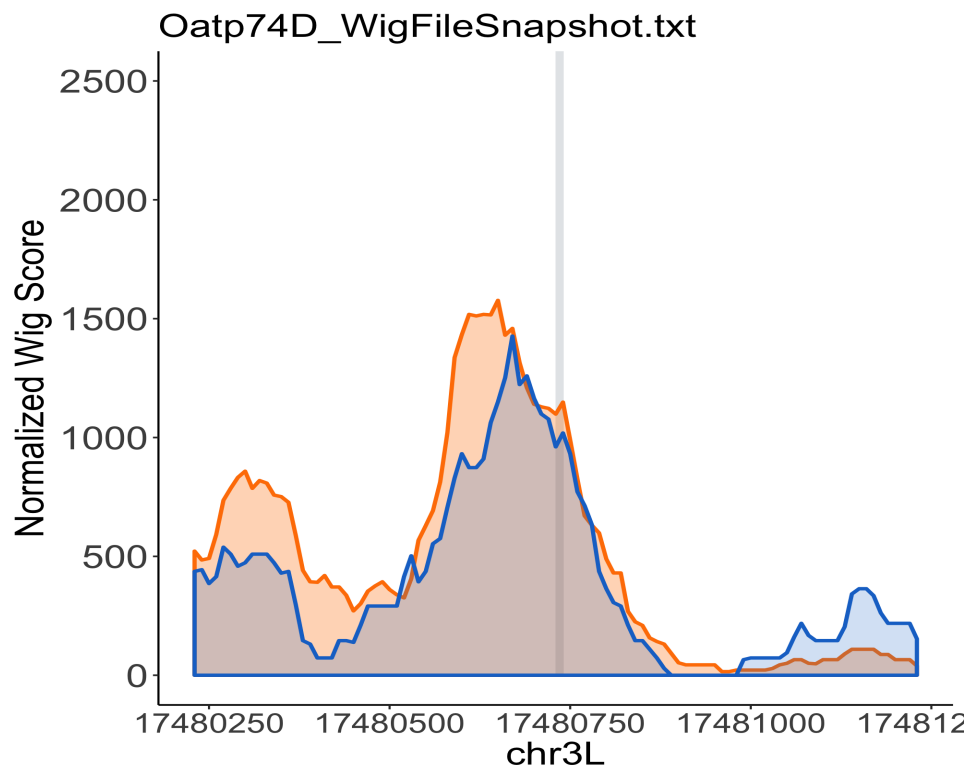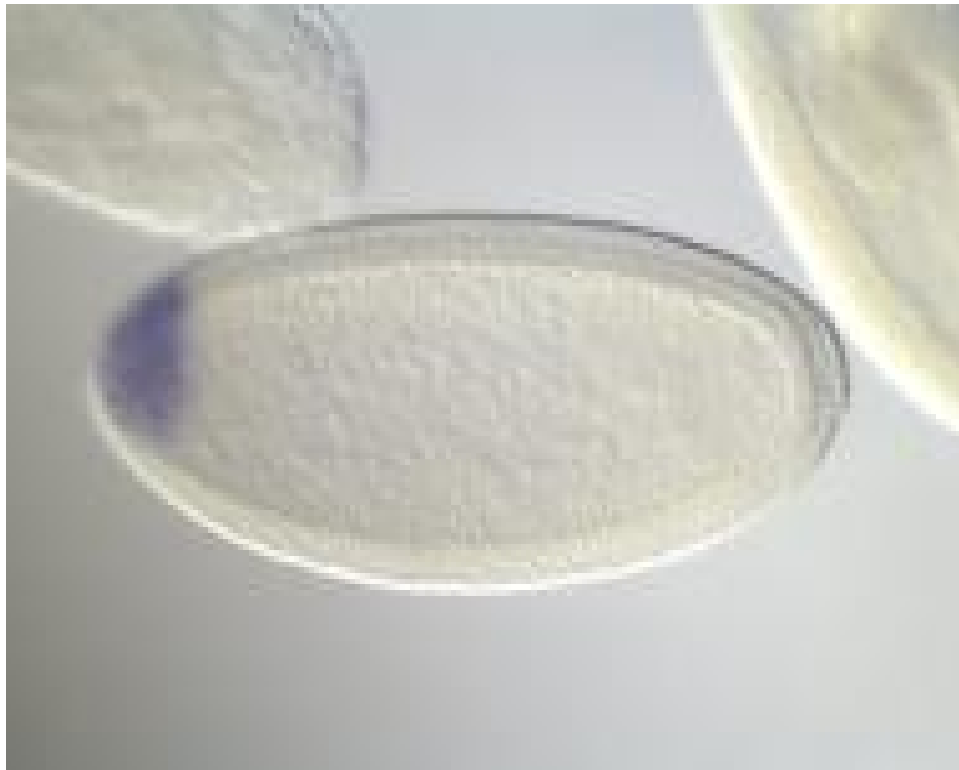

Location: Anterior Type: Promoter ZScore: 0.245549561 PValue: 0.806030938

Supplement: S3 File — Reports consist of in situ hybridization images, ATAC-seq traces, and calculated p-value and Z Score for each region used in the final analysis. (ZIP) [file pgen.1007367.s015.zip › S3_File/Oatp74D_Report.pdf]

## oc\_+7\_construct(80-60)

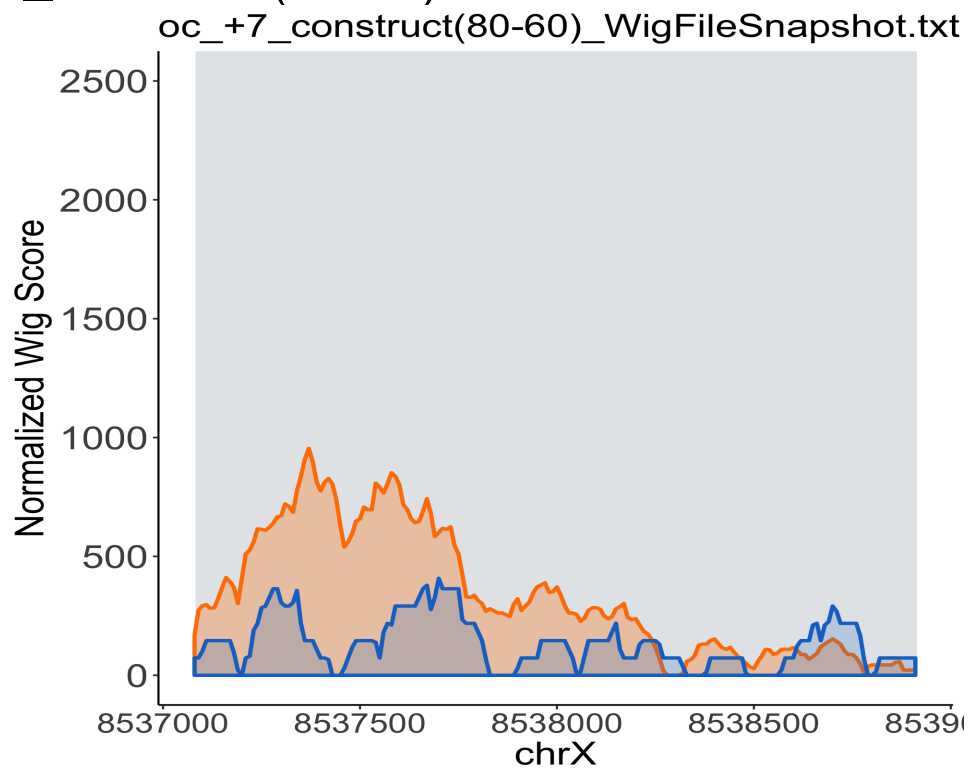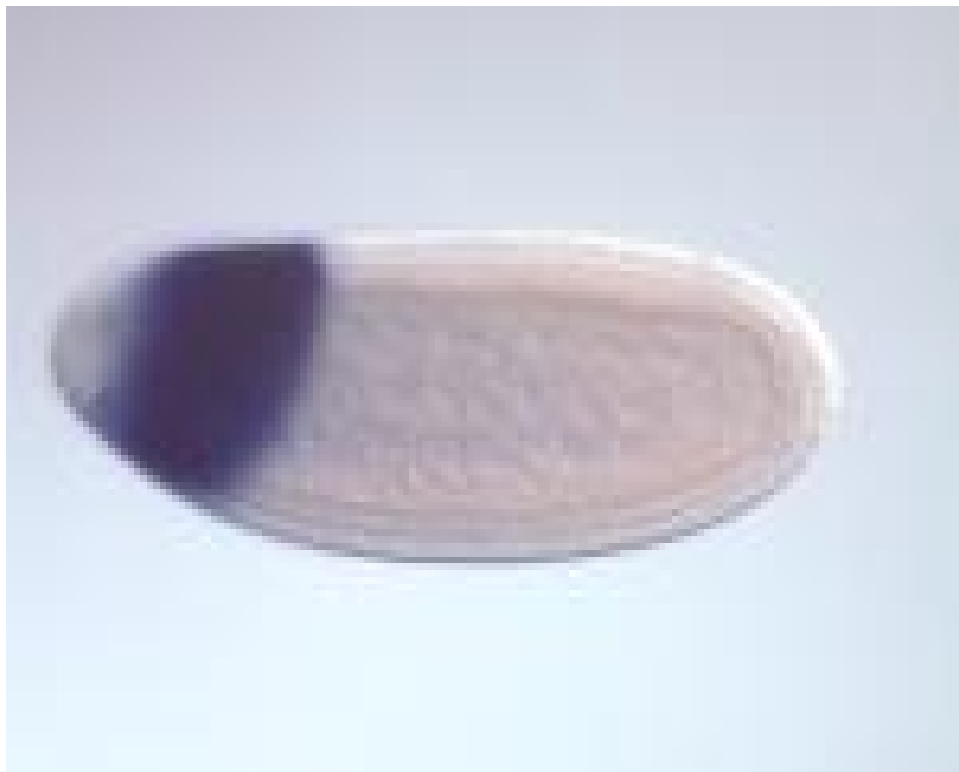

Location: Anterior Type: Enhancer ZScore: 1.930368187 PValue: 0.053561234

Supplement: S3 File — Reports consist of in situ hybridization images, ATAC-seq traces, and calculated p-value and Z Score for each region used in the final analysis. (ZIP) [file pgen.1007367.s015.zip › S3_File/oc_+7_construct(80-60)_Report.pdf]

oc\_intronic\_distal

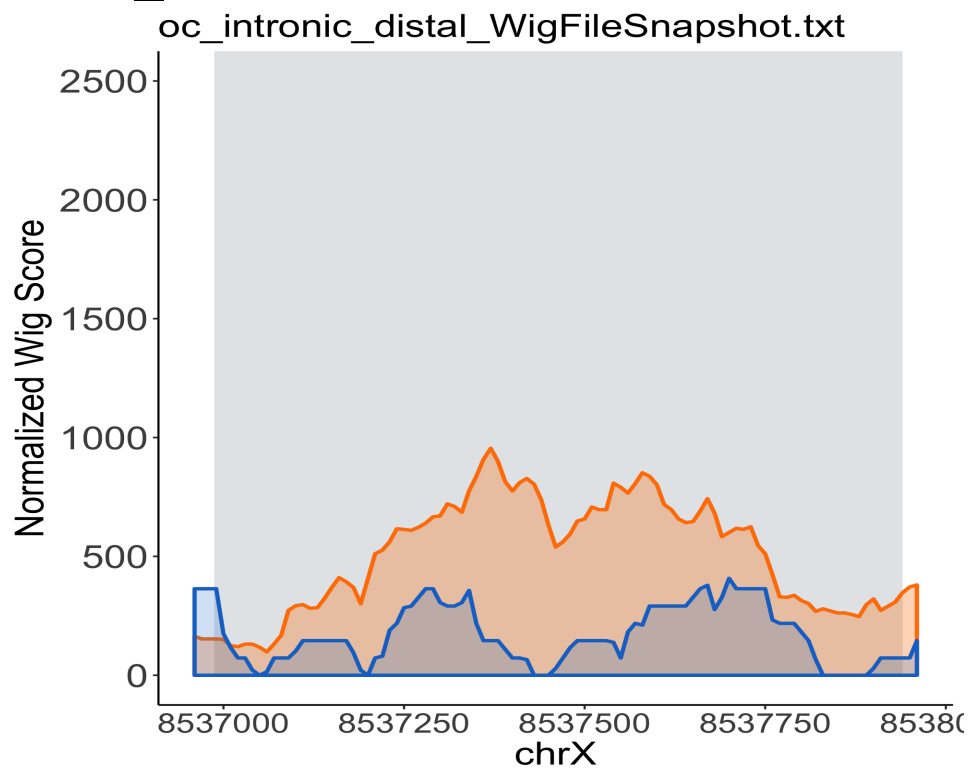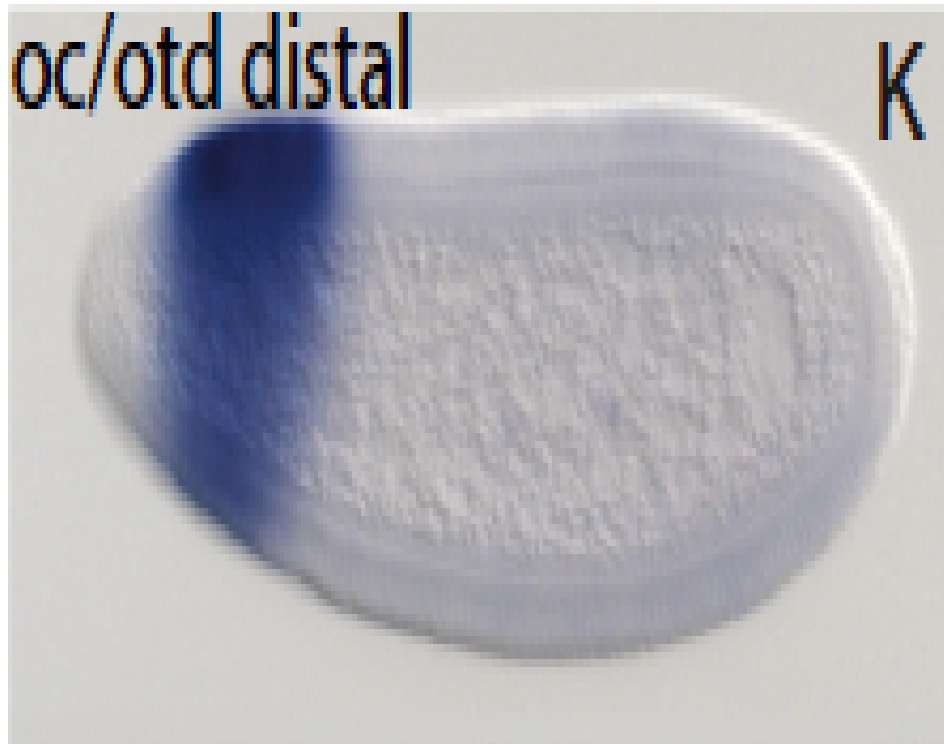

Location: Anterior Type: Enhancer ZScore: 2.260835462 PValue: 0.023769448

Supplement: S3 File — Reports consist of in situ hybridization images, ATAC-seq traces, and calculated p-value and Z Score for each region used in the final analysis. (ZIP) [file pgen.1007367.s015.zip › S3_File/oc_intronic_distal_Report.pdf]

oc\_otd\_early\_enhancer(80-60)

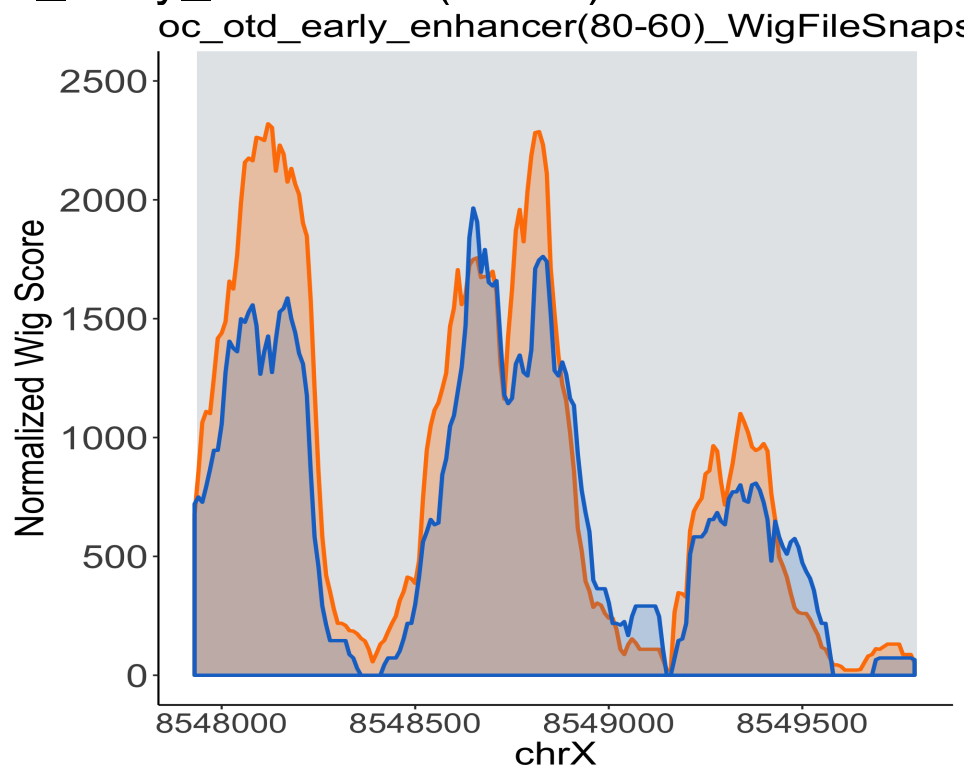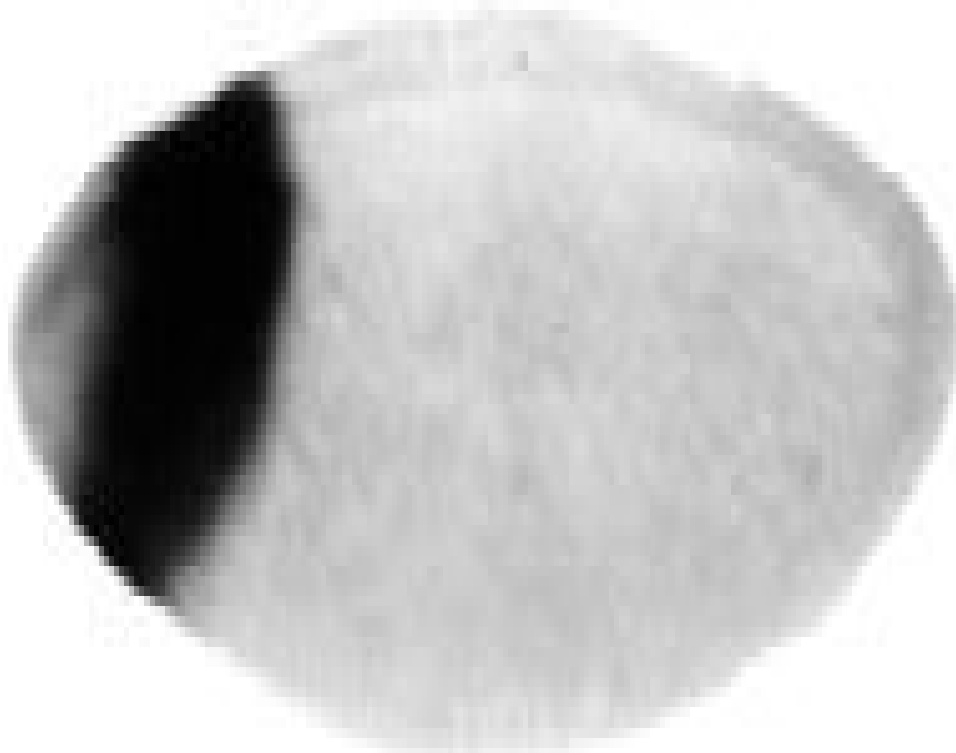

Location: Anterior Type: Enhancer ZScore: 0.466194576 PValue: 0.641076232

Supplement: S3 File — Reports consist of in situ hybridization images, ATAC-seq traces, and calculated p-value and Z Score for each region used in the final analysis. (ZIP) [file pgen.1007367.s015.zip › S3_File/oc_otd_early_enhancer(80-60)_Report.pdf]

oc\_otd\_EHE\_72

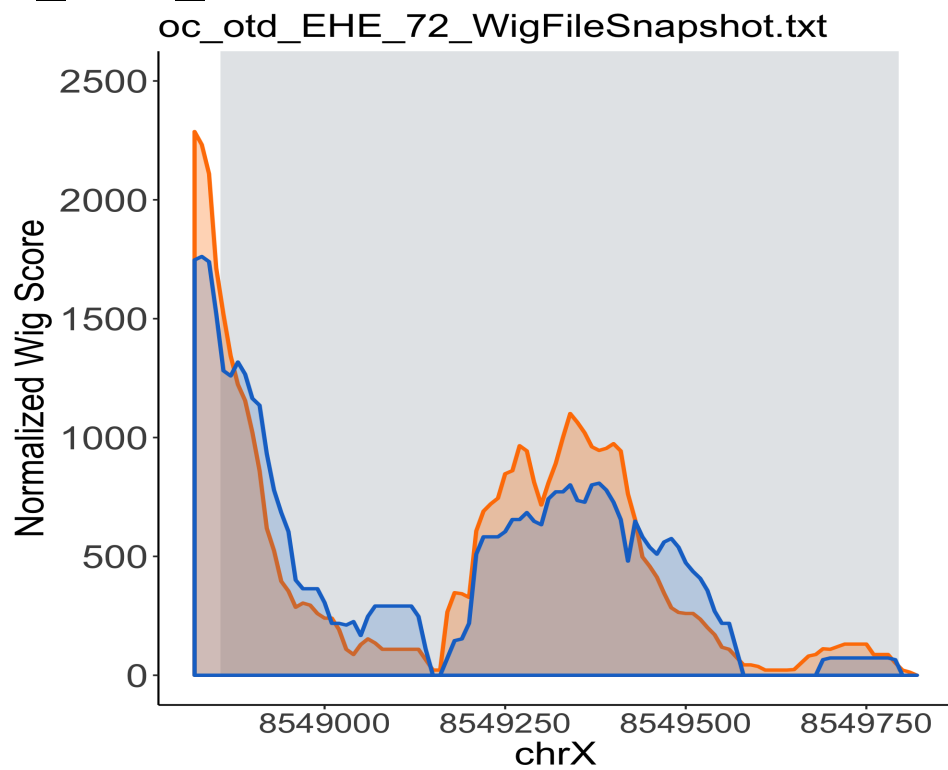

Location: Anterior Type: Enhancer ZScore: -0.008828854 PValue: 0.992955685

Supplement: S3 File — Reports consist of in situ hybridization images, ATAC-seq traces, and calculated p-value and Z Score for each region used in the final analysis. (ZIP) [file pgen.1007367.s015.zip › S3_File/oc_otd_EHE_72_Report.pdf]

OC

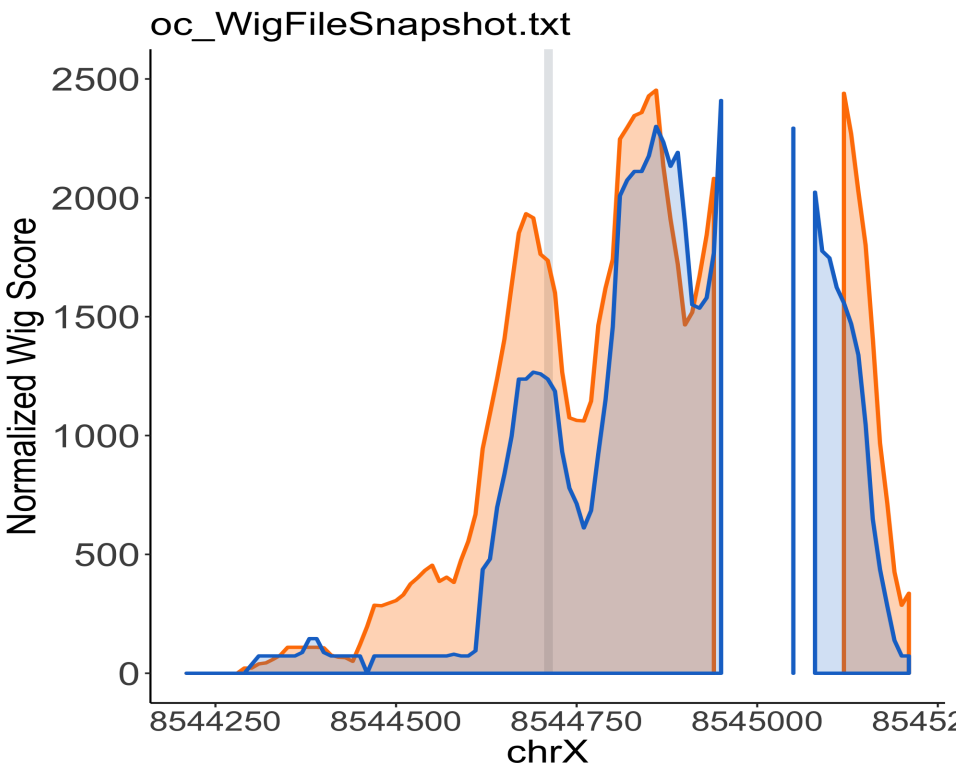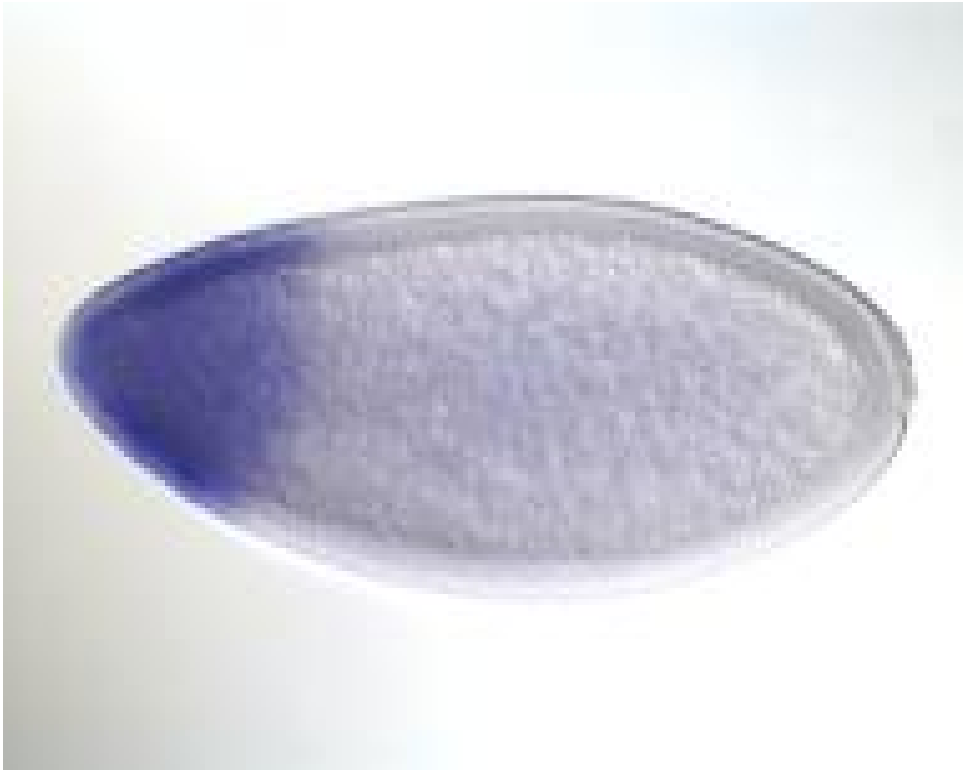

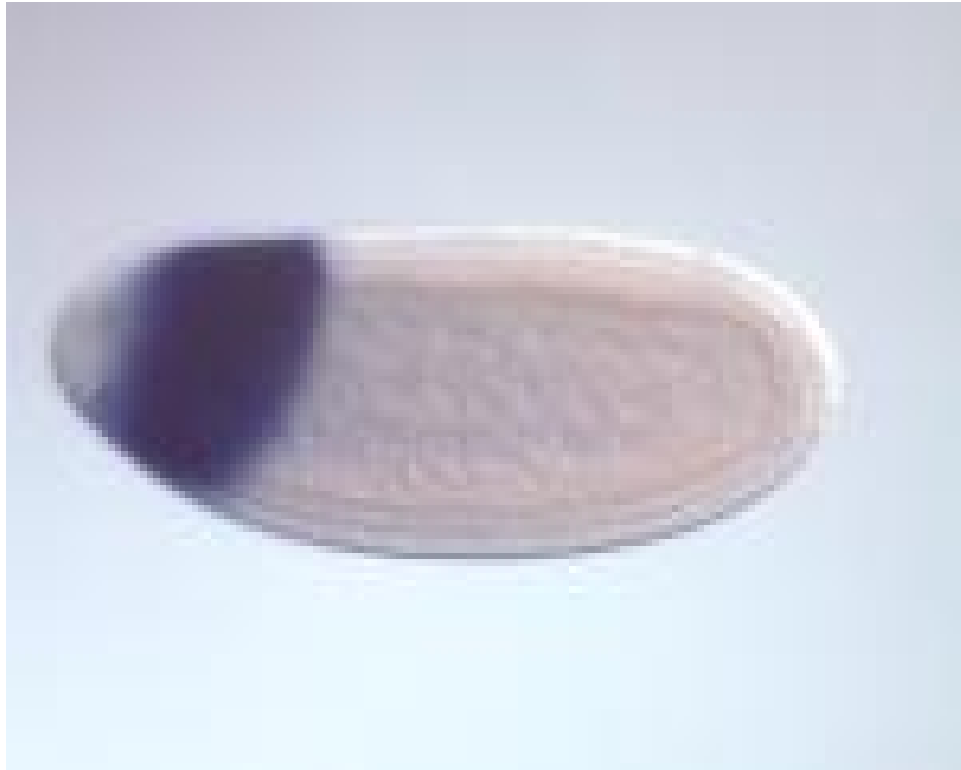

Location: Anterior Type: Promoter ZScore: 0.722521571 PValue: 0.469973868

Supplement: S3 File — Reports consist of in situ hybridization images, ATAC-seq traces, and calculated p-value and Z Score for each region used in the final analysis. (ZIP) [file pgen.1007367.s015.zip › S3_File/oc_Report.pdf]

## Ocho\_Ozdemir

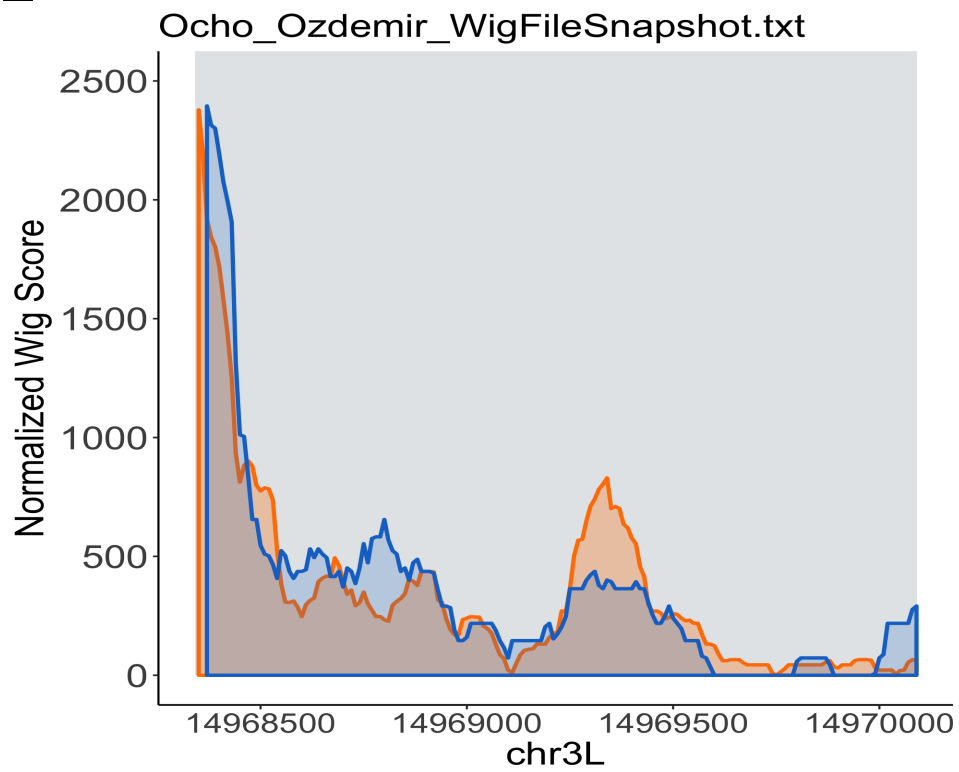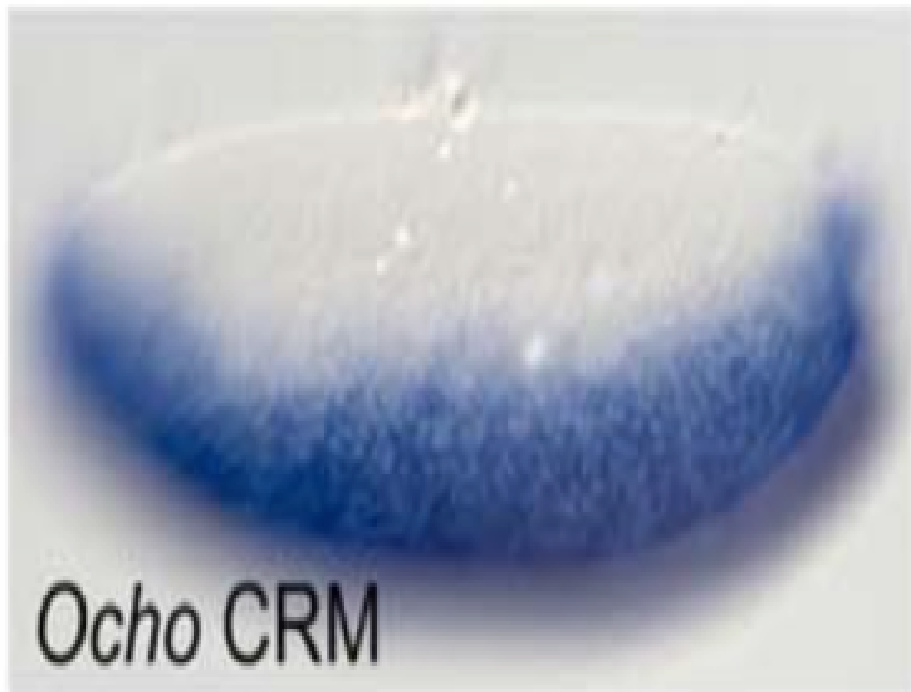

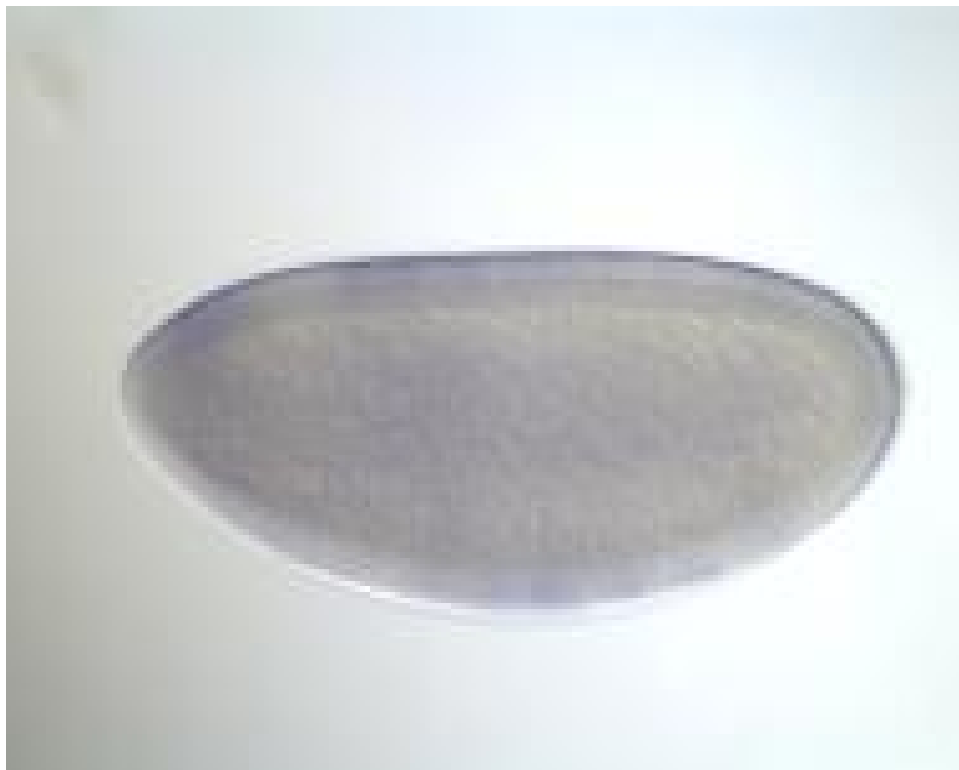

Location: Ventral Type: Enhancer ZScore: -0.208121003 PValue: 0.835134488

Supplement: S3 File — Reports consist of in situ hybridization images, ATAC-seq traces, and calculated p-value and Z Score for each region used in the final analysis. (ZIP) [file pgen.1007367.s015.zip › S3_File/Ocho_Ozdemir_Report.pdf]

OVO

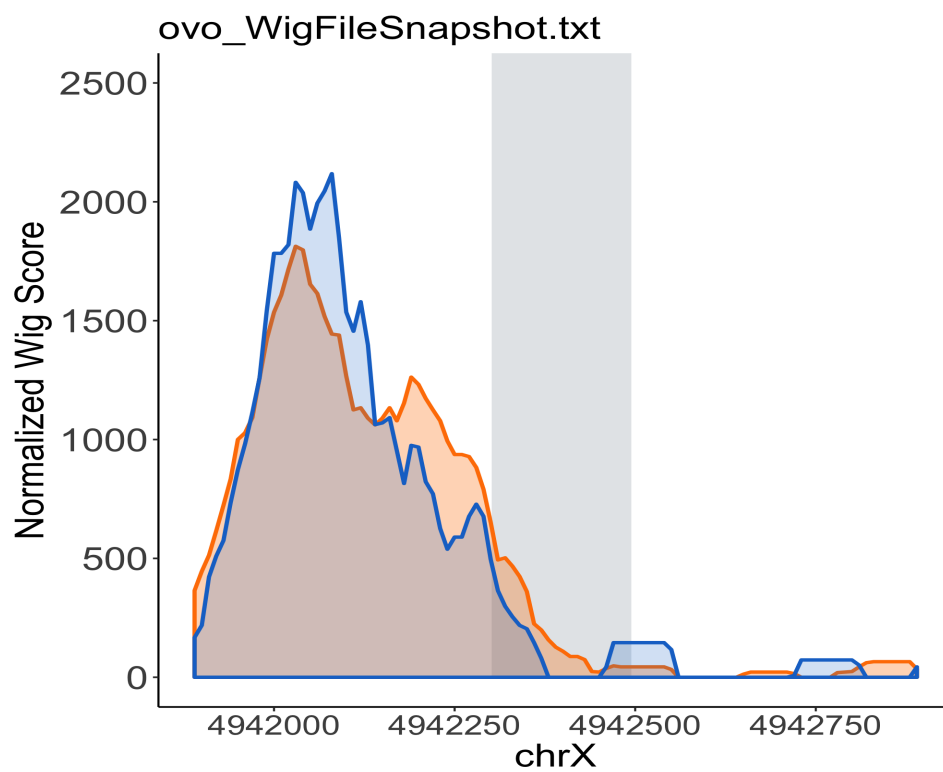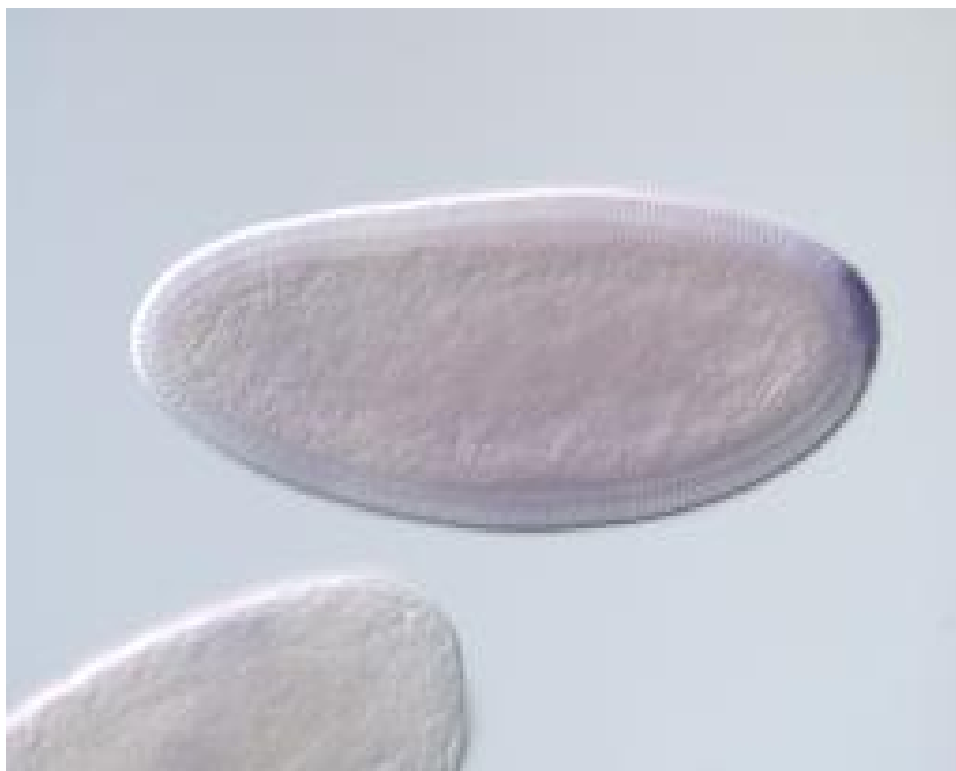

Location: Posterior Type: Promoter ZScore: -1.166802126 PValue: 0.243290288

Supplement: S3 File — Reports consist of in situ hybridization images, ATAC-seq traces, and calculated p-value and Z Score for each region used in the final analysis. (ZIP) [file pgen.1007367.s015.zip › S3_File/ovo_Report.pdf]

pdm2\_+1\_construct(50-30)

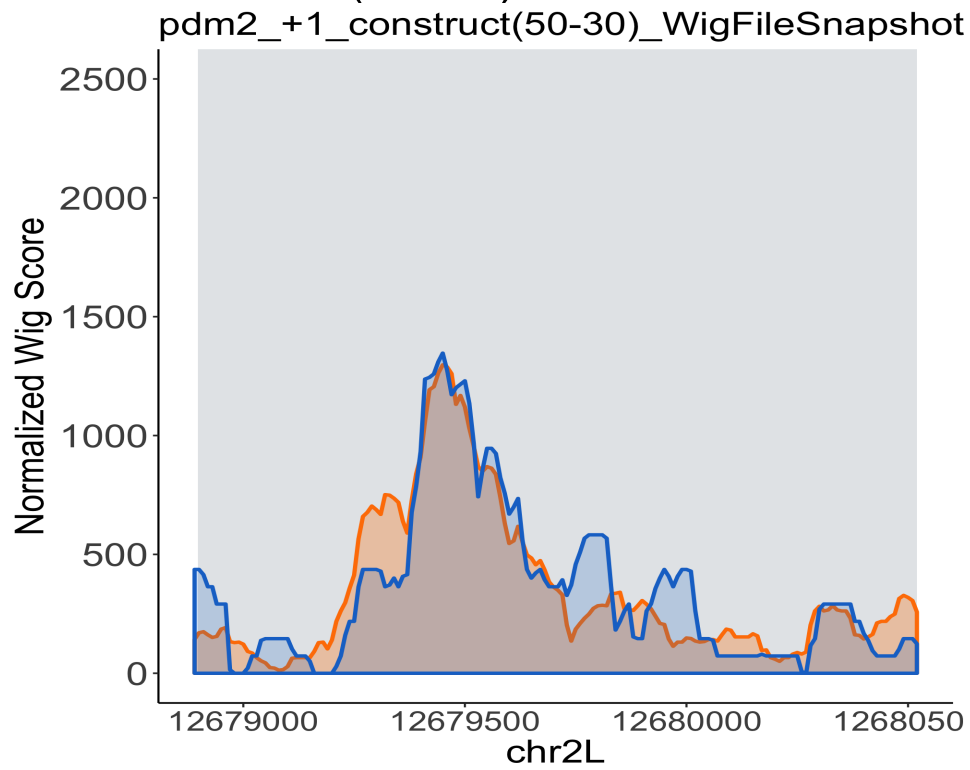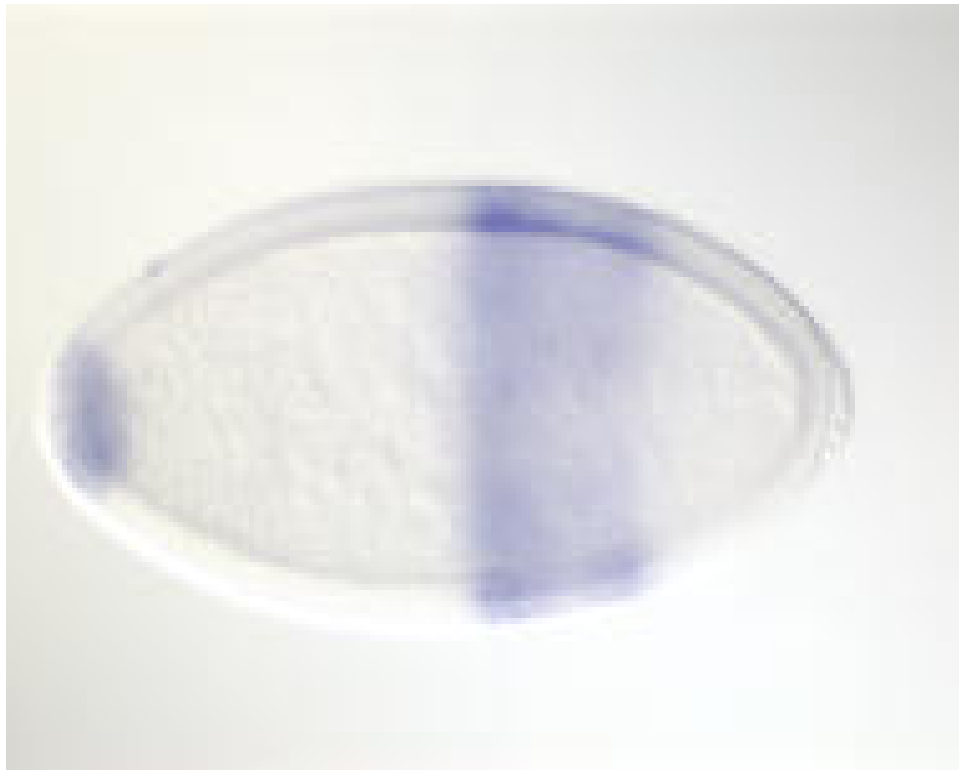

Location: Mostly Post Type: Enhancer ZScore: -0.043025859 PValue: 0.96568092

Supplement: S3 File — Reports consist of in situ hybridization images, ATAC-seq traces, and calculated p-value and Z Score for each region used in the final analysis. (ZIP) [file pgen.1007367.s015.zip › S3_File/pdm2_+1_construct(50-30)_Report.pdf]

# Pepck

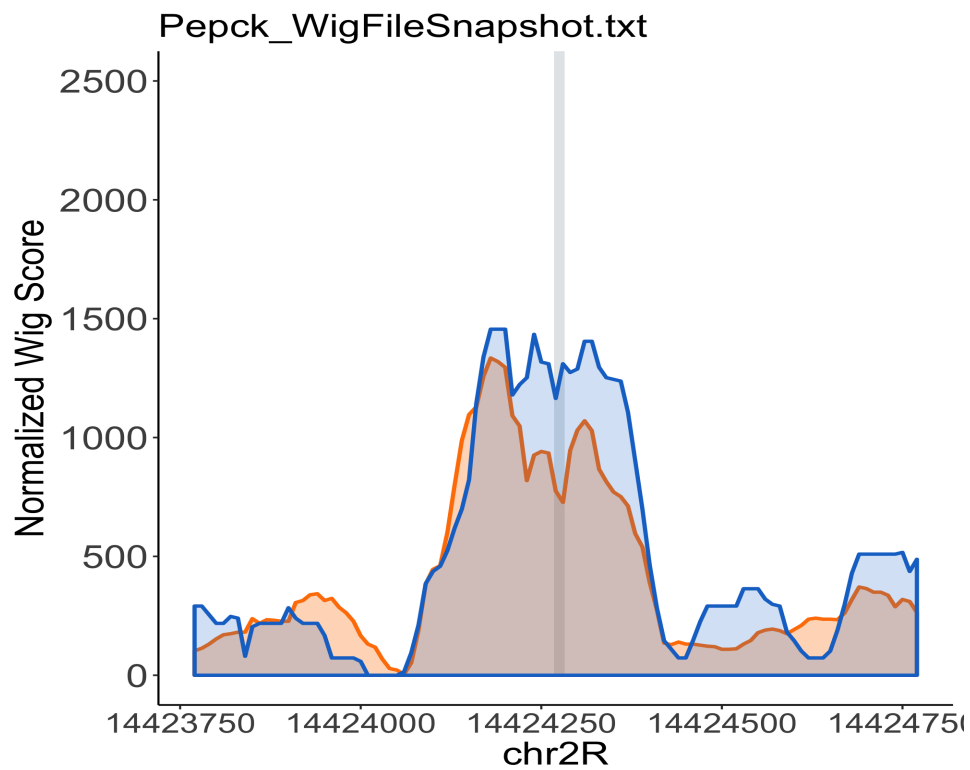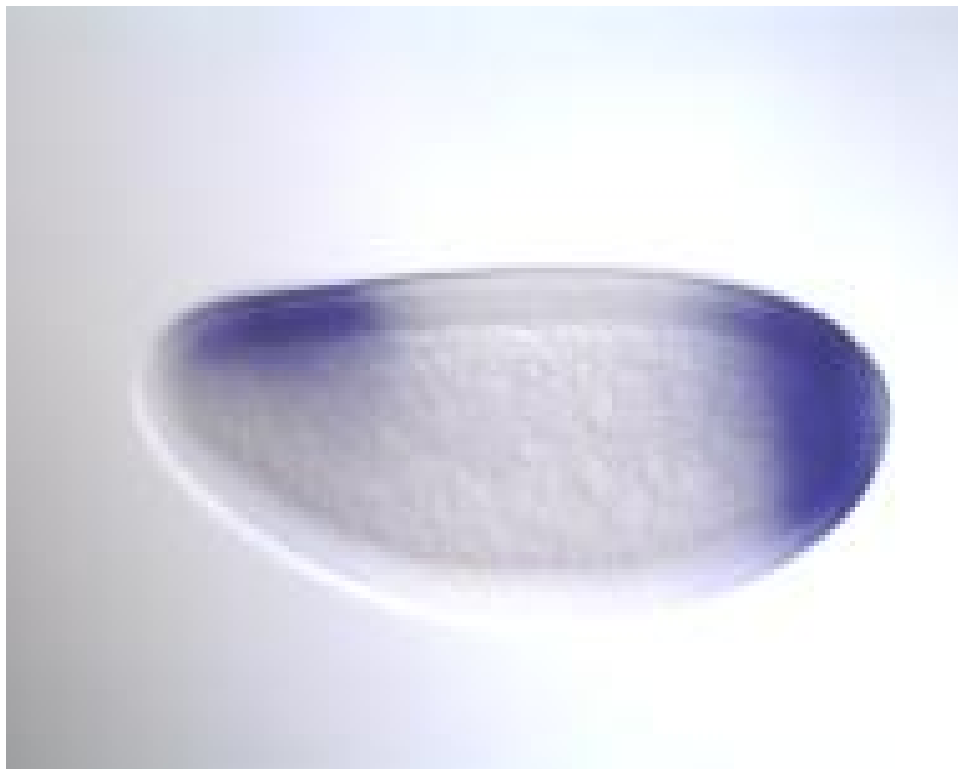

Location: Mostly Post Type: Promoter ZScore: 0.949377608 PValue: 0.342428595

Supplement: S3 File — Reports consist of in situ hybridization images, ATAC-seq traces, and calculated p-value and Z Score for each region used in the final analysis. (ZIP) [file pgen.1007367.s015.zip › S3_File/Pepck_Report.pdf]

# Phm\_Markstein

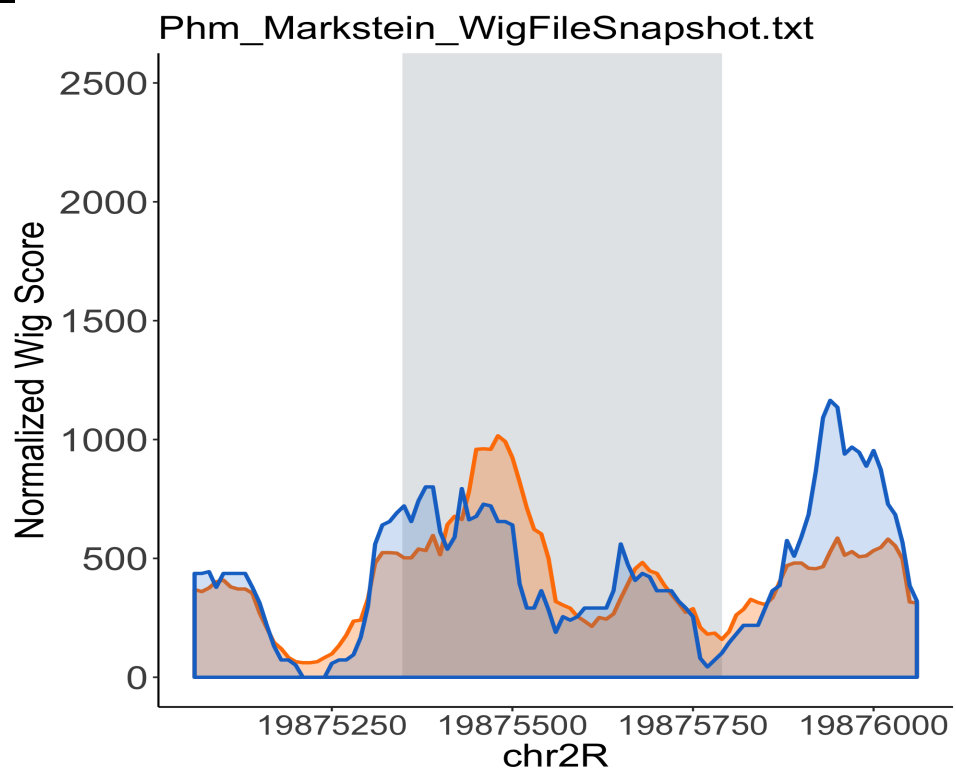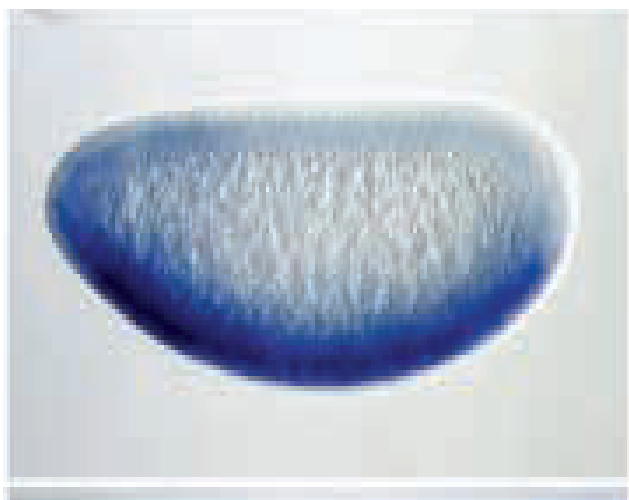

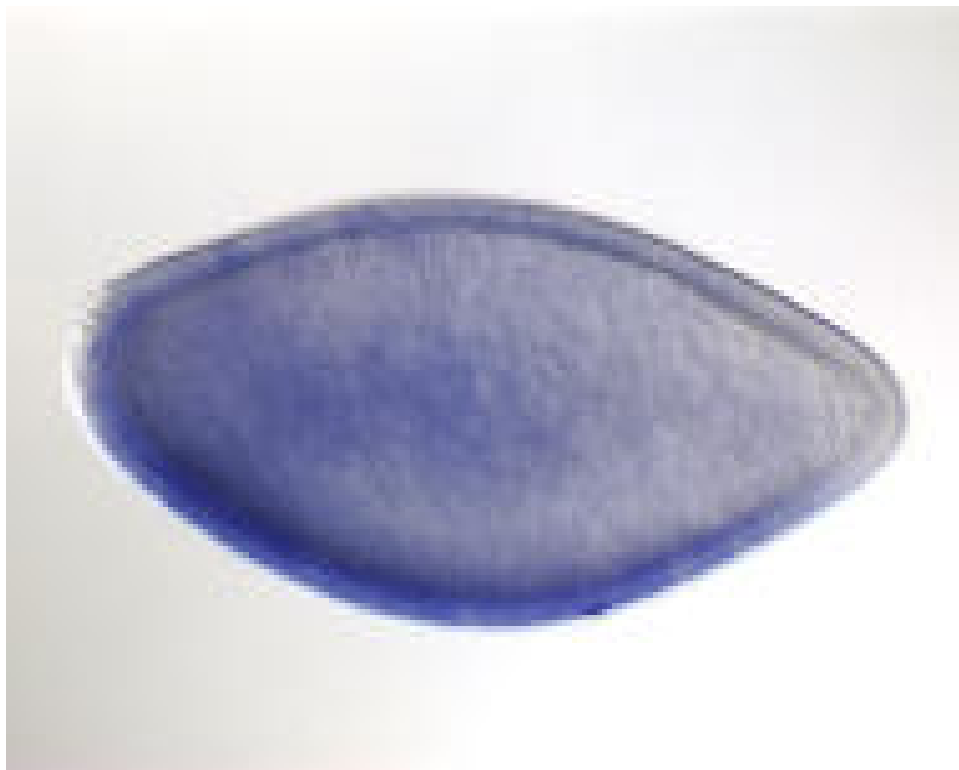

Location: Ventral Type: Enhancer ZScore: 0.220041267 PValue: 0.825839016

Supplement: S3 File — Reports consist of in situ hybridization images, ATAC-seq traces, and calculated p-value and Z Score for each region used in the final analysis. (ZIP) [file pgen.1007367.s015.zip › S3_File/Phm_Markstein_Report.pdf]

phm

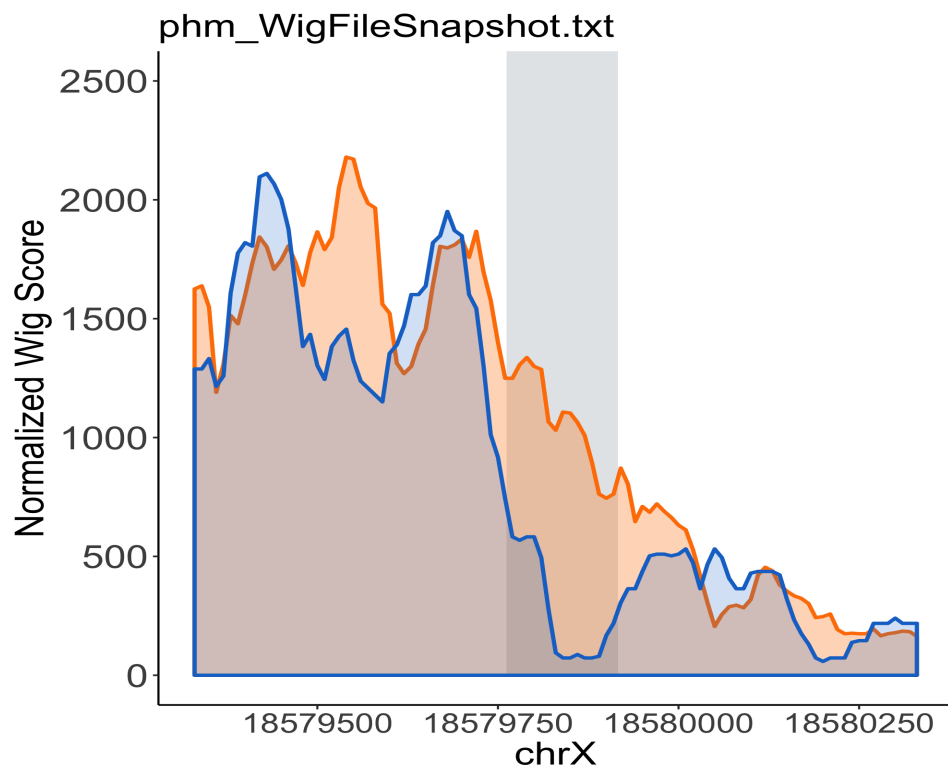

Location: Ventral Type: Promoter ZScore: 2.560383995 PValue: 0.010455656

Supplement: S3 File — Reports consist of in situ hybridization images, ATAC-seq traces, and calculated p-value and Z Score for each region used in the final analysis. (ZIP) [file pgen.1007367.s015.zip › S3_File/phm_Report.pdf]

# Pi3K21B

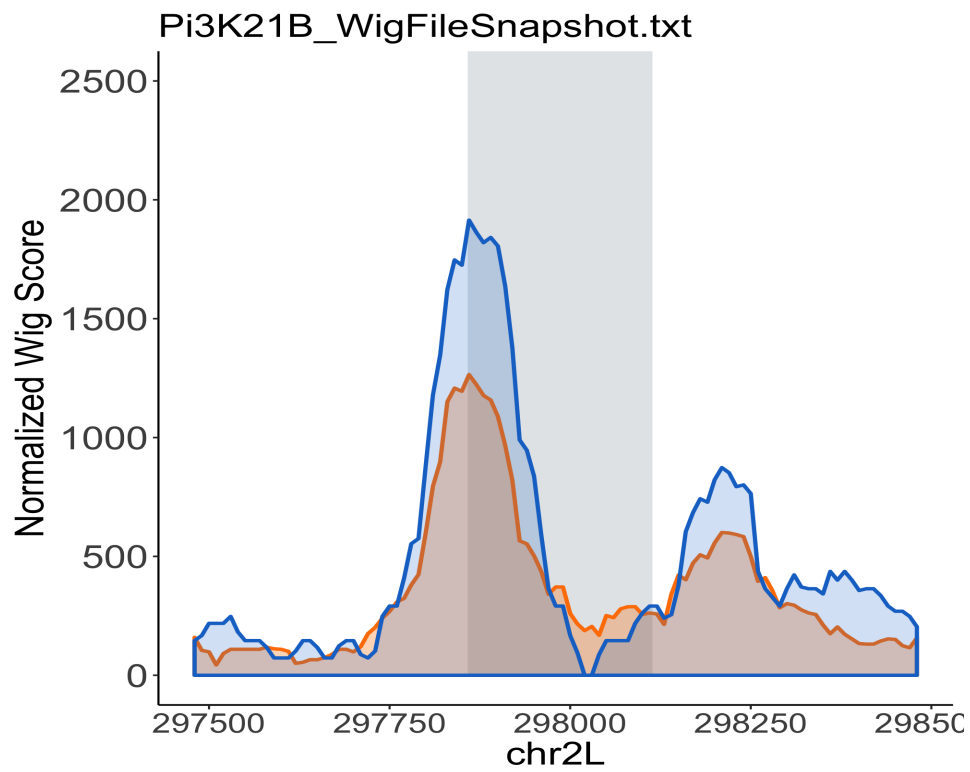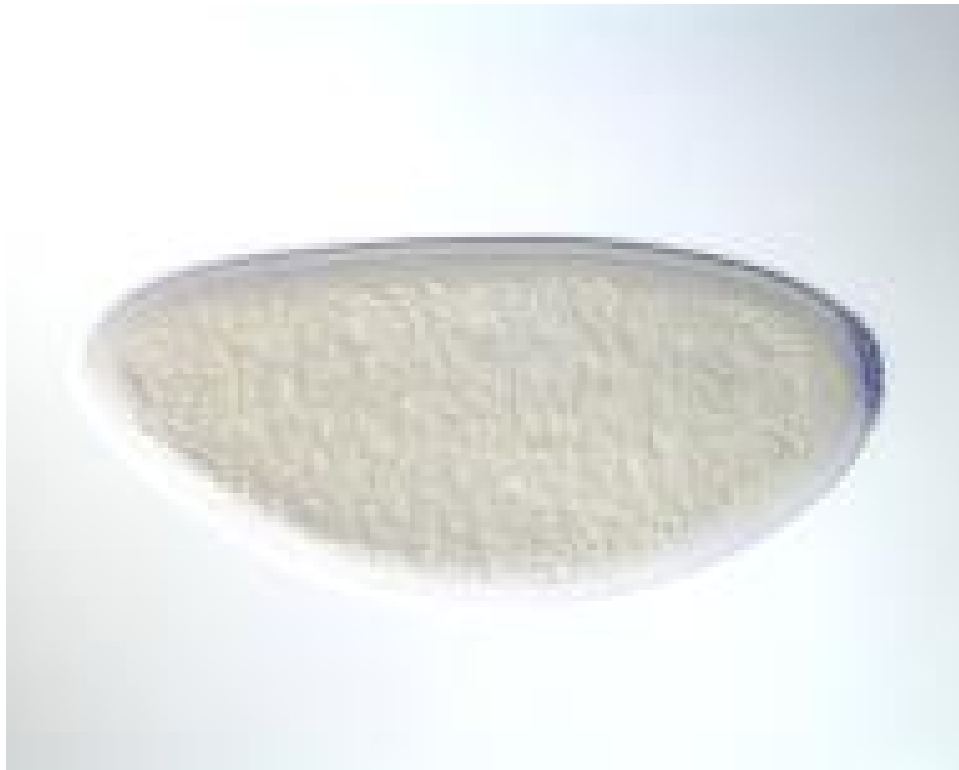

Location: Posterior Type: Promoter ZScore: 0.619816905 PValue: 0.535378338

Supplement: S3 File — Reports consist of in situ hybridization images, ATAC-seq traces, and calculated p-value and Z Score for each region used in the final analysis. (ZIP) [file pgen.1007367.s015.zip › S3_File/Pi3K21B_Report.pdf]

pnr

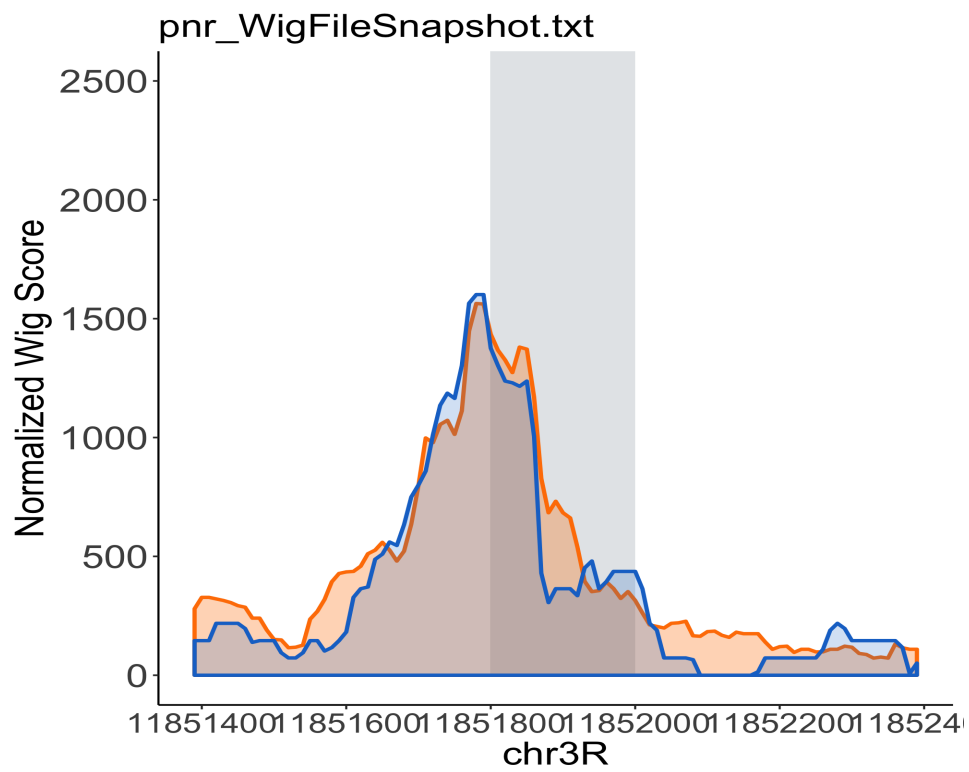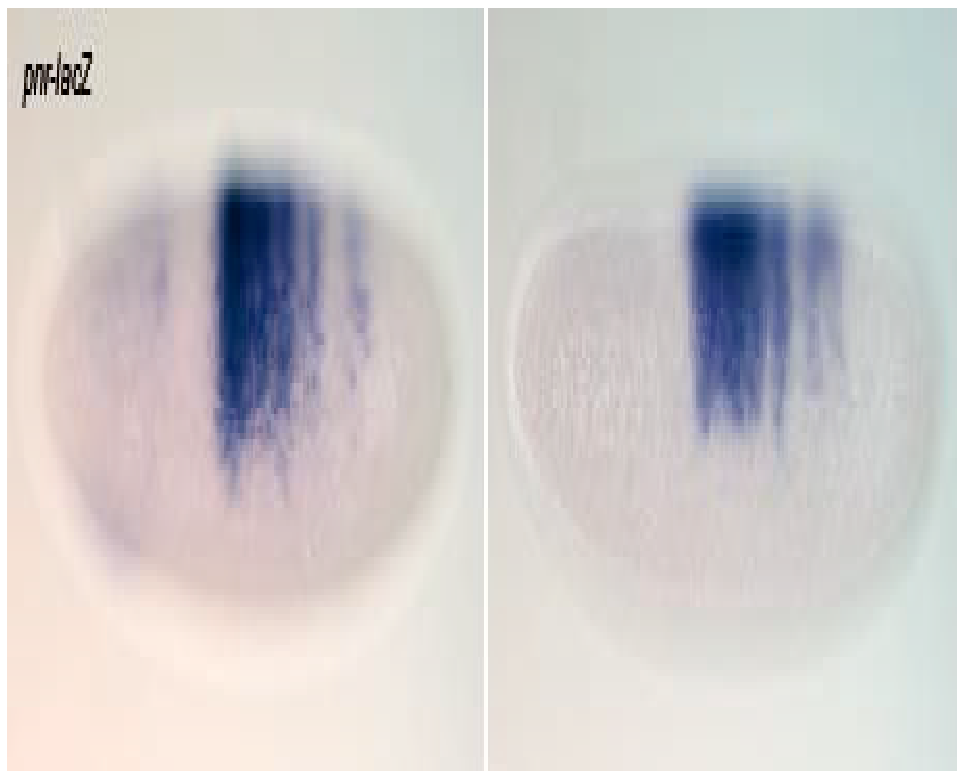

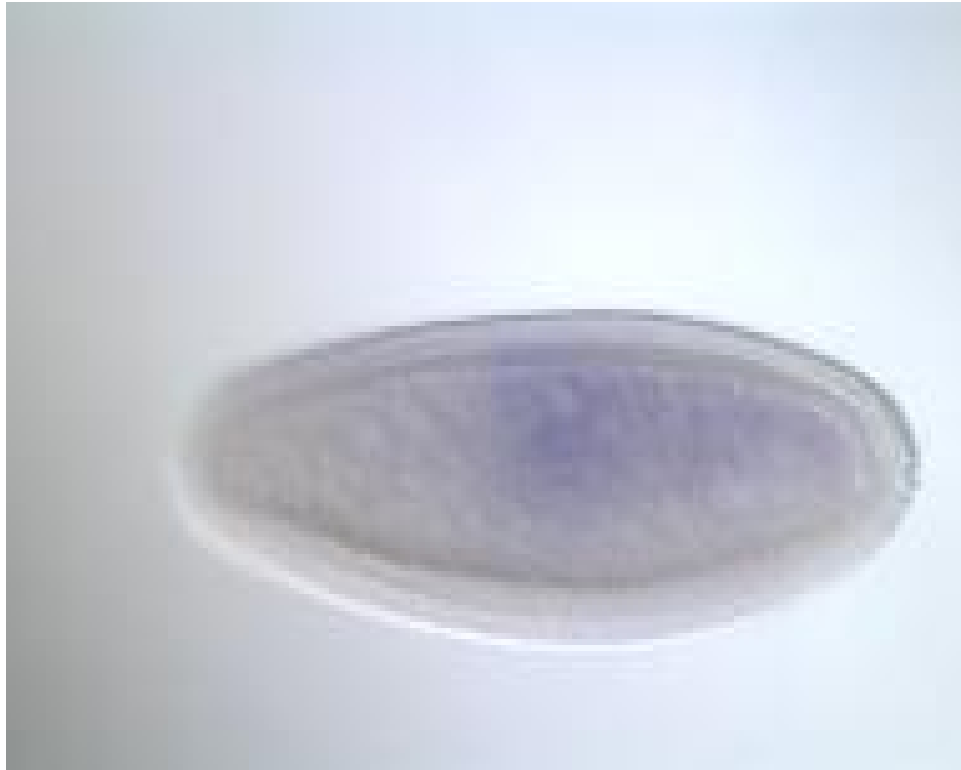

Location: Dorsal Type: Promoter ZScore: 0.236038213 PValue: 0.813403018

Supplement: S3 File — Reports consist of in situ hybridization images, ATAC-seq traces, and calculated p-value and Z Score for each region used in the final analysis. (ZIP) [file pgen.1007367.s015.zip › S3_File/pnr_Report.pdf]

# pnr\_Zeitlinger

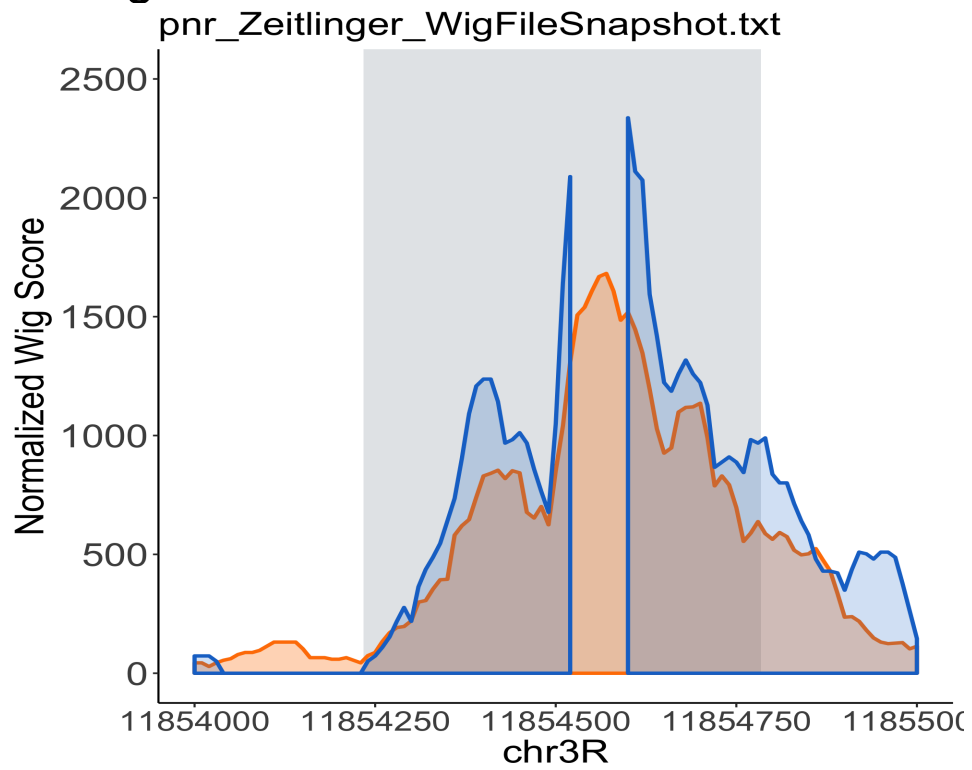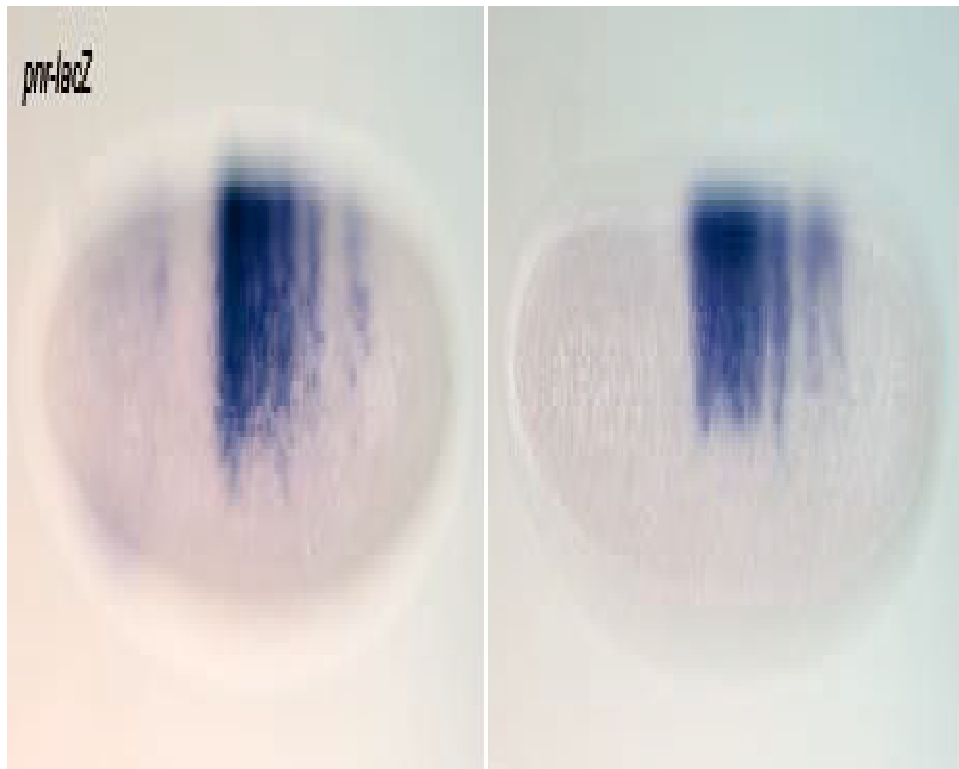

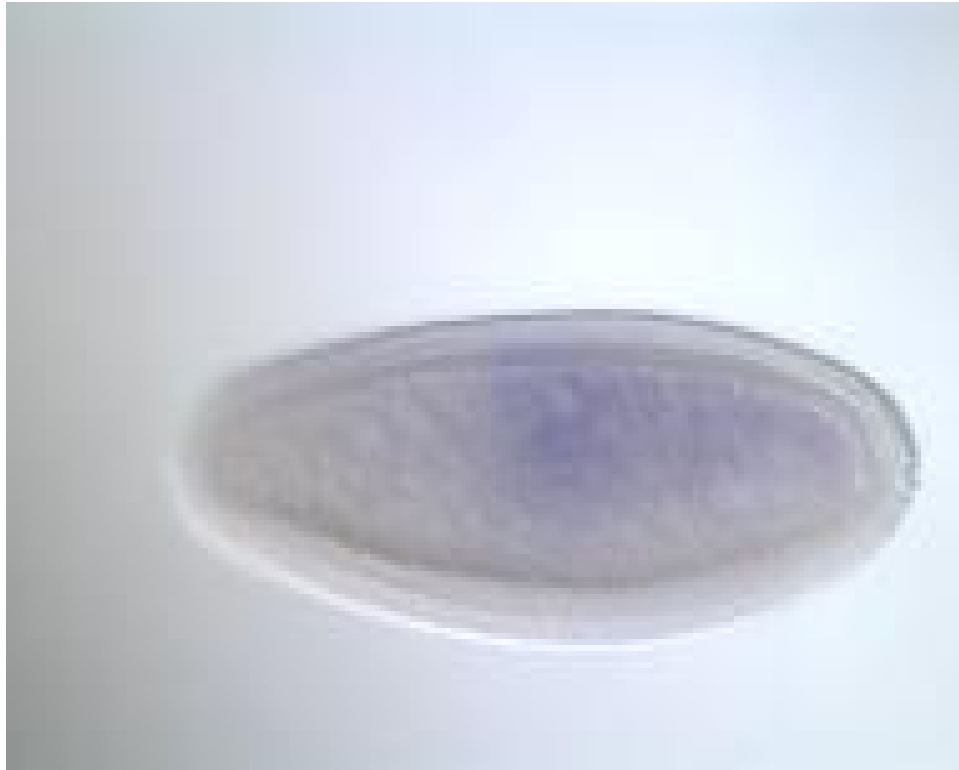

Location: Dorsal Type: Enhancer ZScore: -0.830910144 PValue: 0.406024392

Supplement: S3 File — Reports consist of in situ hybridization images, ATAC-seq traces, and calculated p-value and Z Score for each region used in the final analysis. (ZIP) [file pgen.1007367.s015.zip › S3_File/pnr_Zeitlinger_Report.pdf]

pnt\_Ozdemir

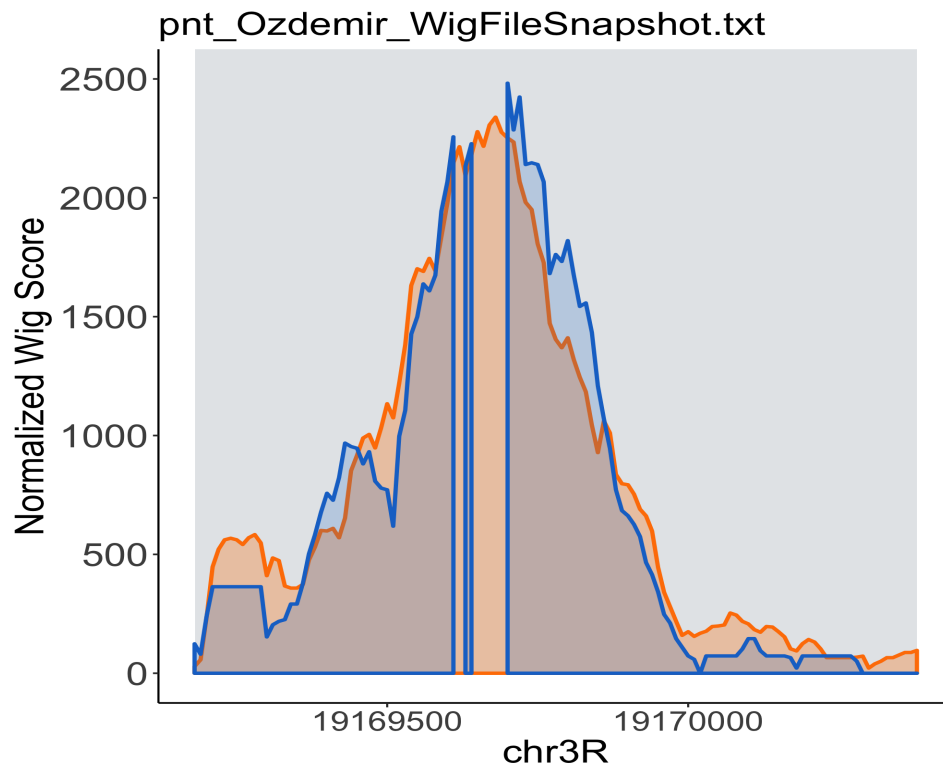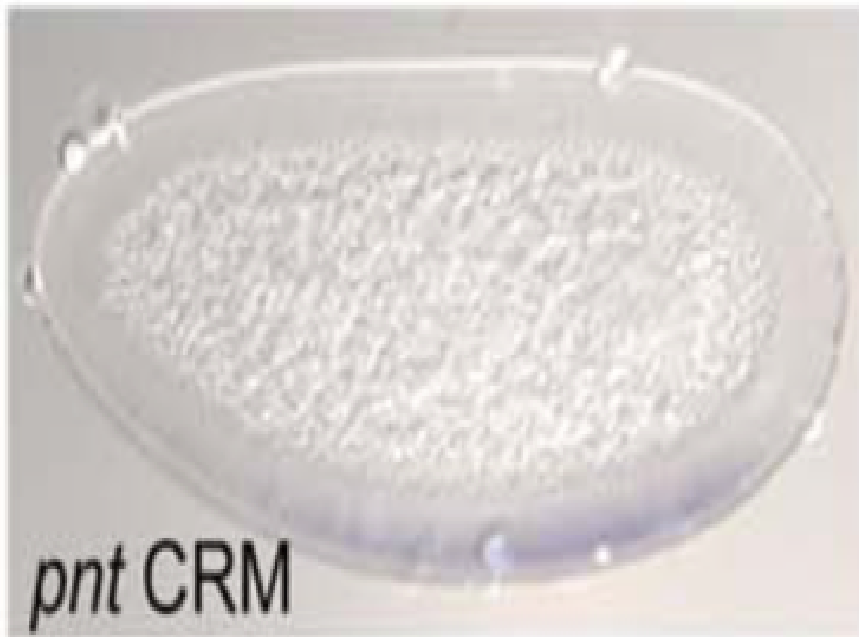

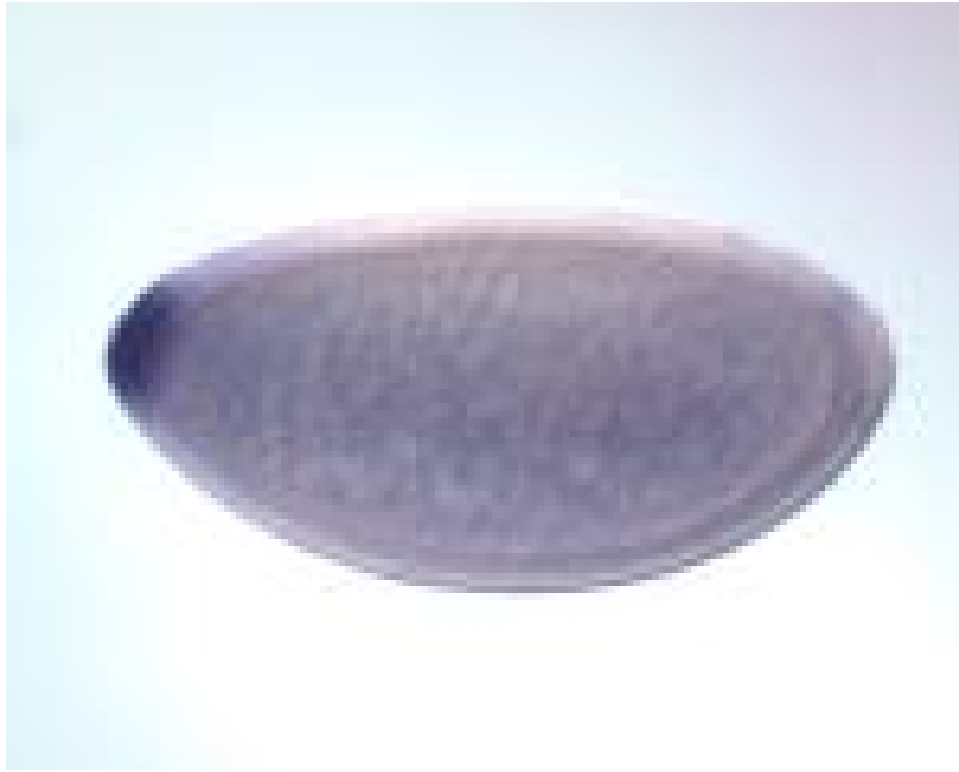

Location: Ventral Type: Enhancer ZScore: -0.023647202 PValue: 0.981134021

Supplement: S3 File — Reports consist of in situ hybridization images, ATAC-seq traces, and calculated p-value and Z Score for each region used in the final analysis. (ZIP) [file pgen.1007367.s015.zip › S3_File/pnt_Ozdemir_Report.pdf]

# Ptp4E

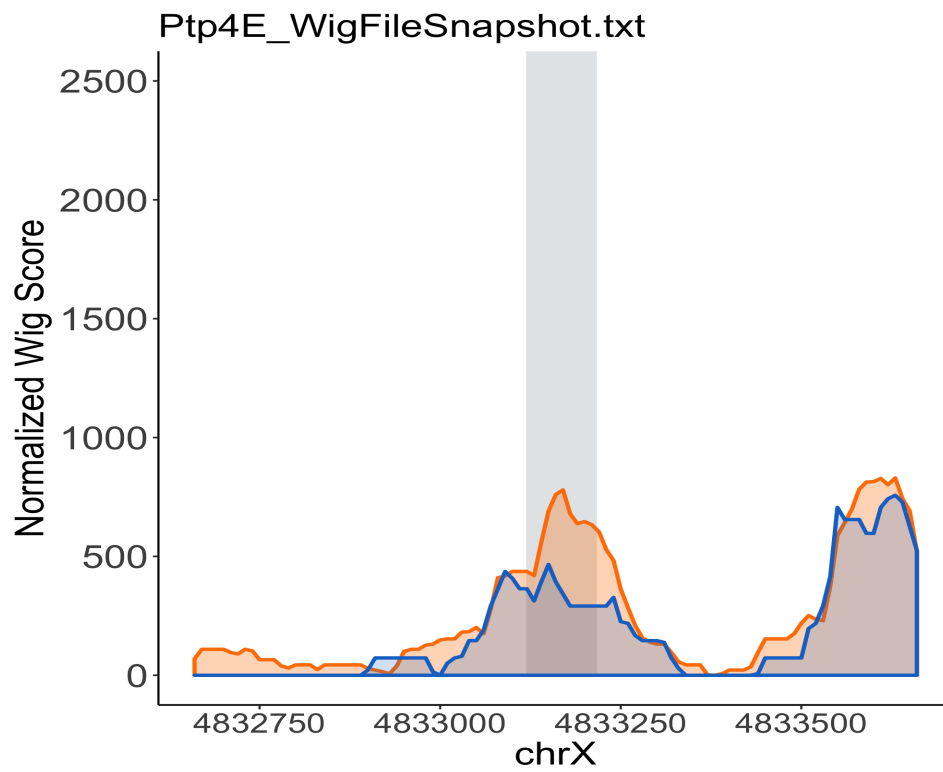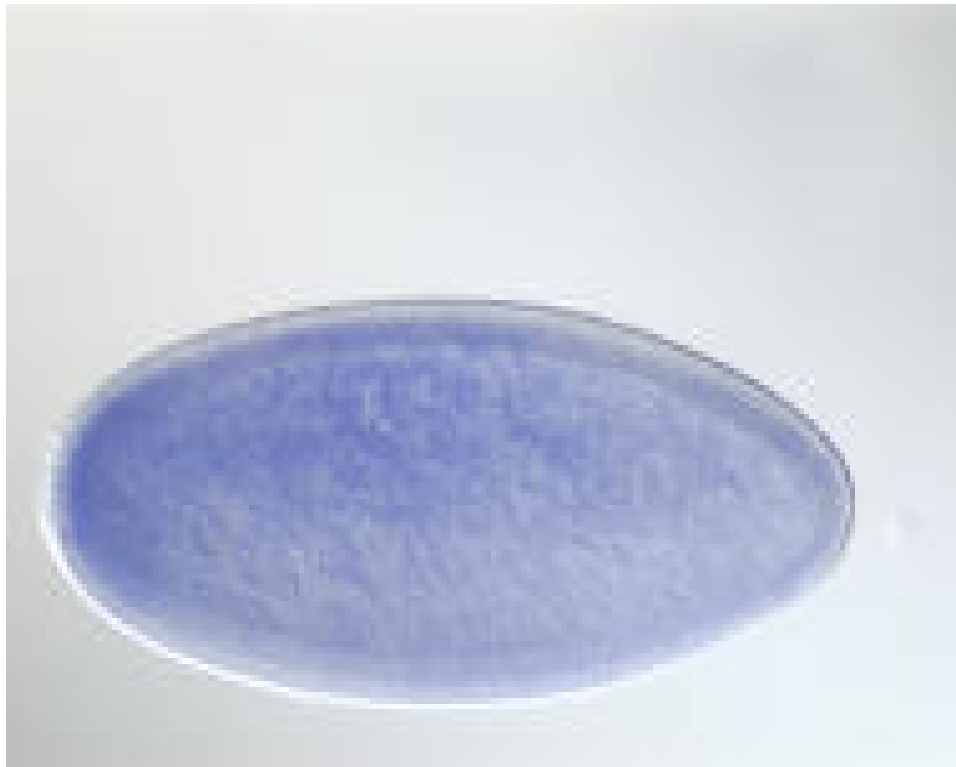

Location: Dorsal Type: Promoter ZScore: 1.216724328 PValue: 0.22370912

Supplement: S3 File — Reports consist of in situ hybridization images, ATAC-seq traces, and calculated p-value and Z Score for each region used in the final analysis. (ZIP) [file pgen.1007367.s015.zip › S3_File/Ptp4E_Report.pdf]

puc

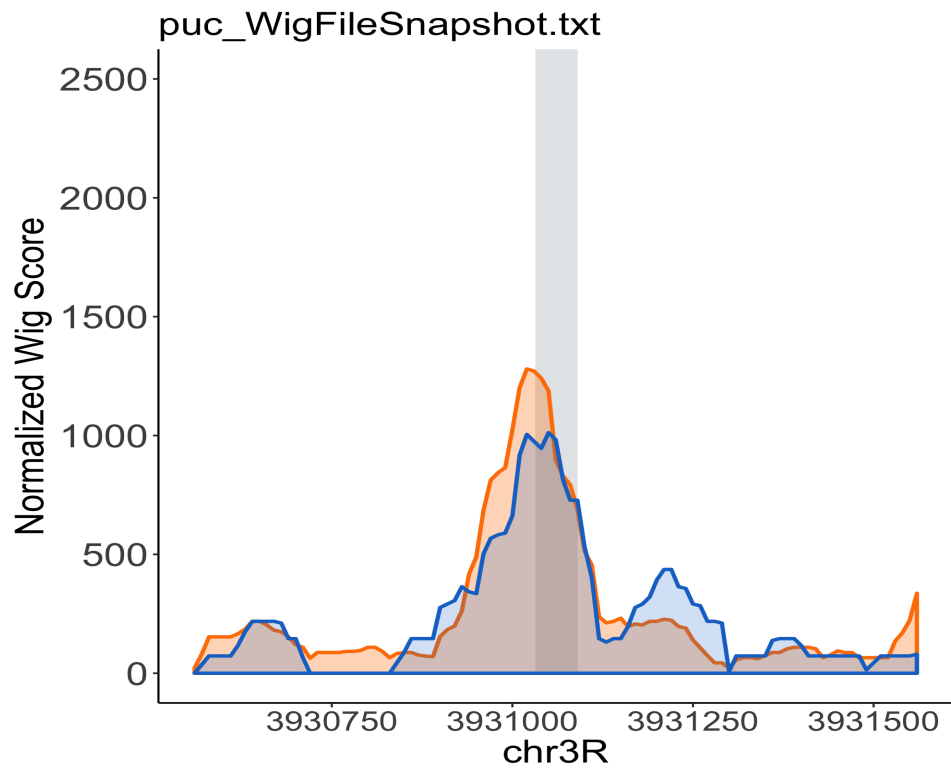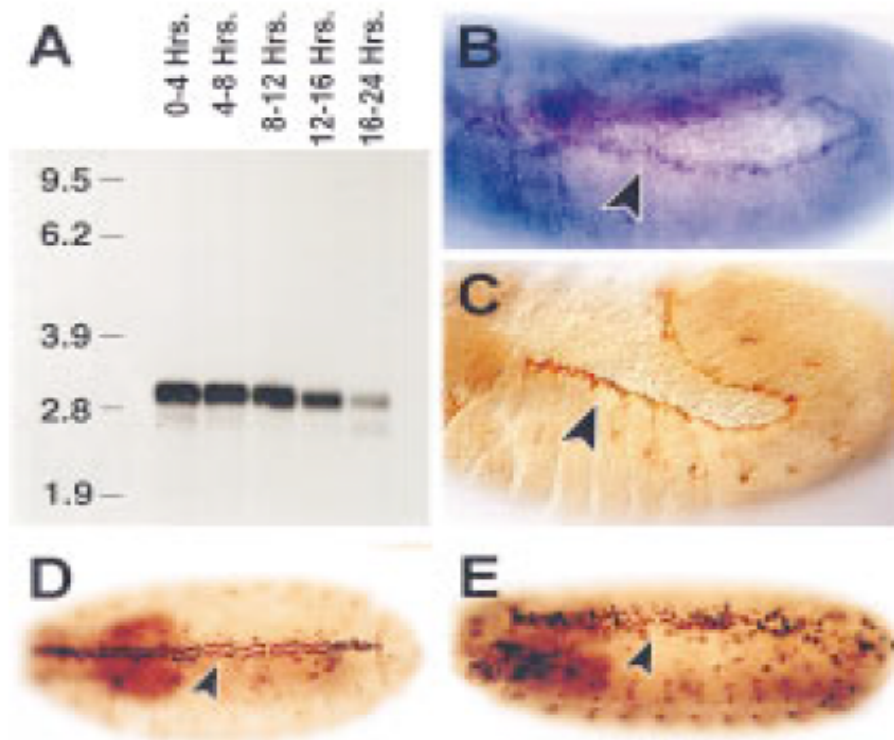

Location: Dorsal Type: Promoter ZScore: 0.208519977 PValue: 0.834822986

Supplement: S3 File — Reports consist of in situ hybridization images, ATAC-seq traces, and calculated p-value and Z Score for each region used in the final analysis. (ZIP) [file pgen.1007367.s015.zip › S3_File/puc_Report.pdf]

pyr

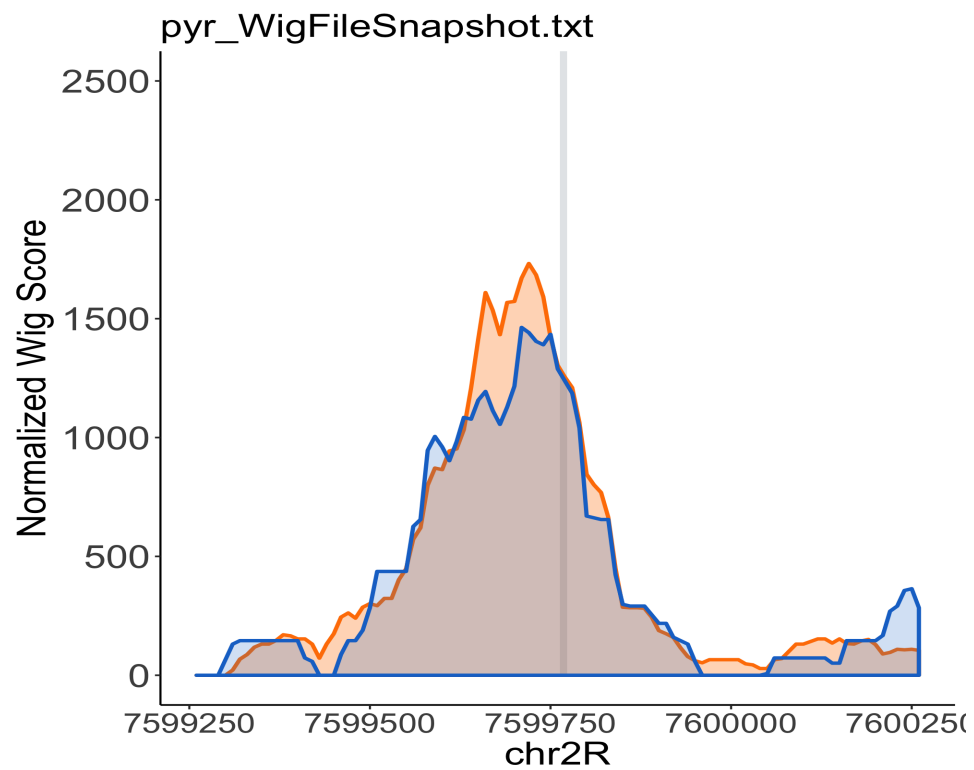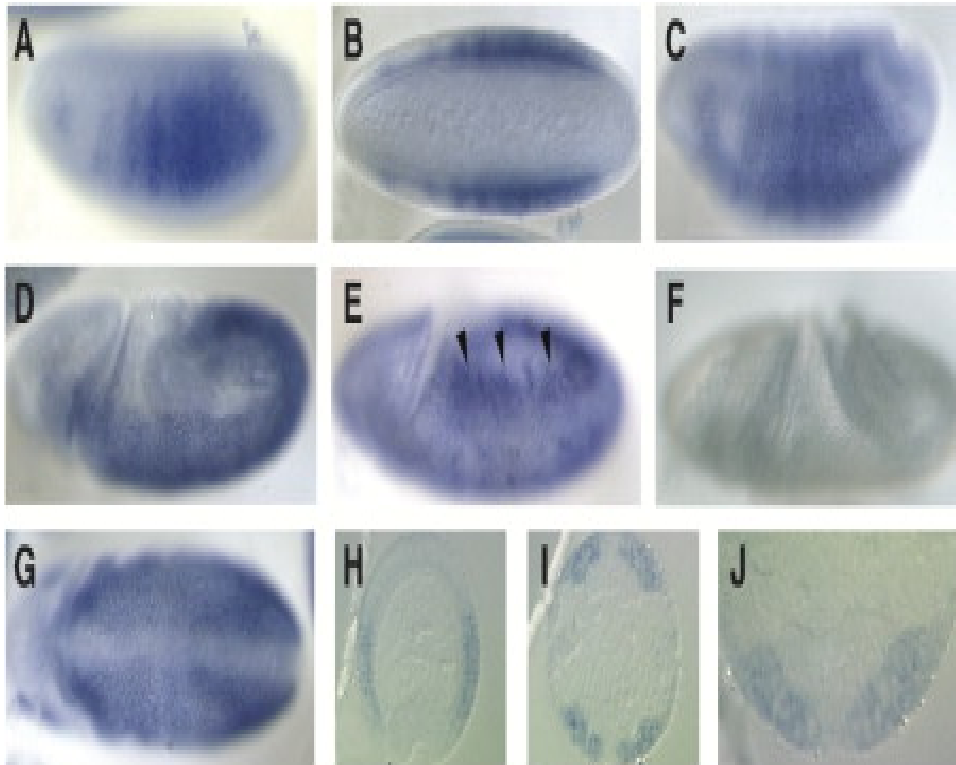

Location: Ventral Type: Promoter ZScore: -0.016386302 PValue: 0.986926207

Supplement: S3 File — Reports consist of in situ hybridization images, ATAC-seq traces, and calculated p-value and Z Score for each region used in the final analysis. (ZIP) [file pgen.1007367.s015.zip › S3_File/pyr_Report.pdf]

Rb97D

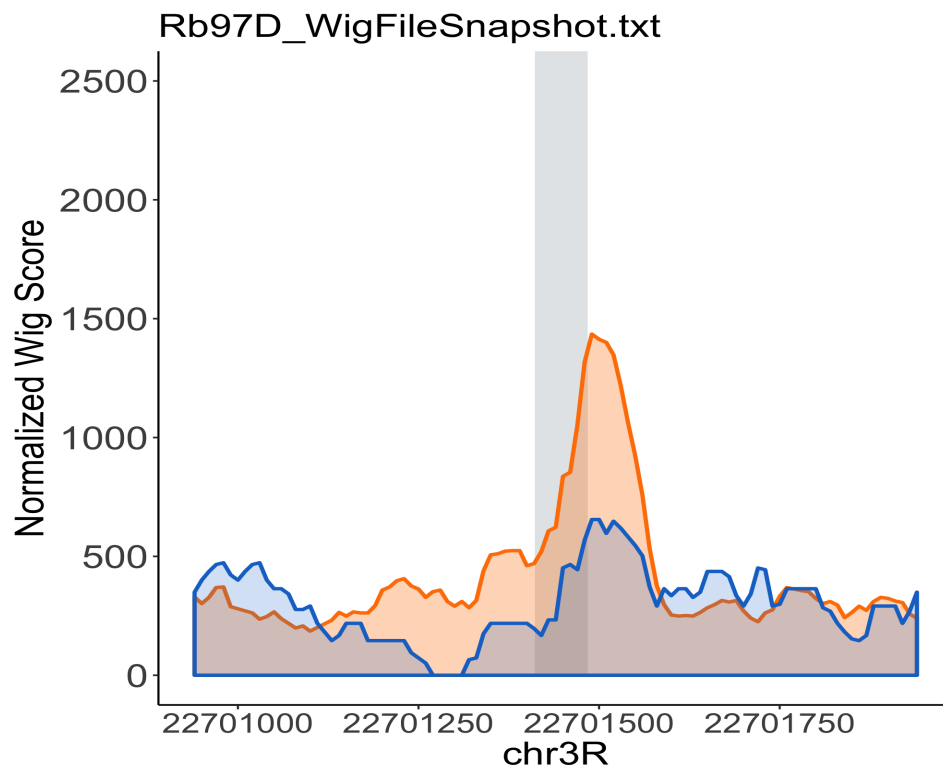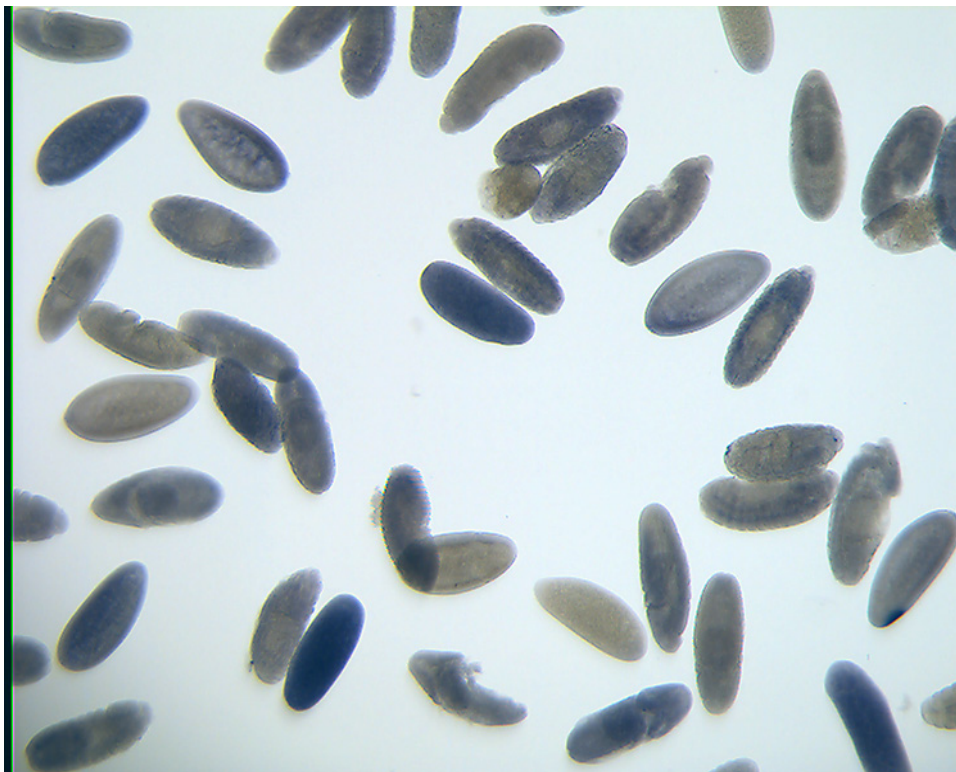

Location: Dorsal Type: Promoter ZScore: 1.74362832 PValue: 0.081223922

Supplement: S3 File — Reports consist of in situ hybridization images, ATAC-seq traces, and calculated p-value and Z Score for each region used in the final analysis. (ZIP) [file pgen.1007367.s015.zip › S3_File/Rb97D_Report.pdf]

rho

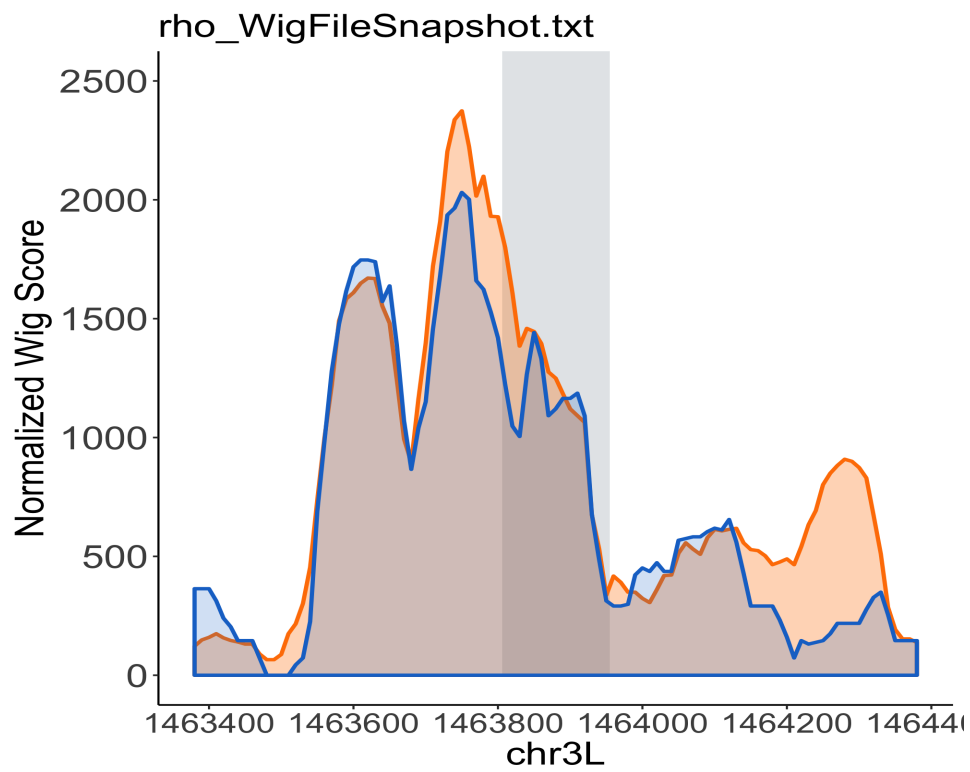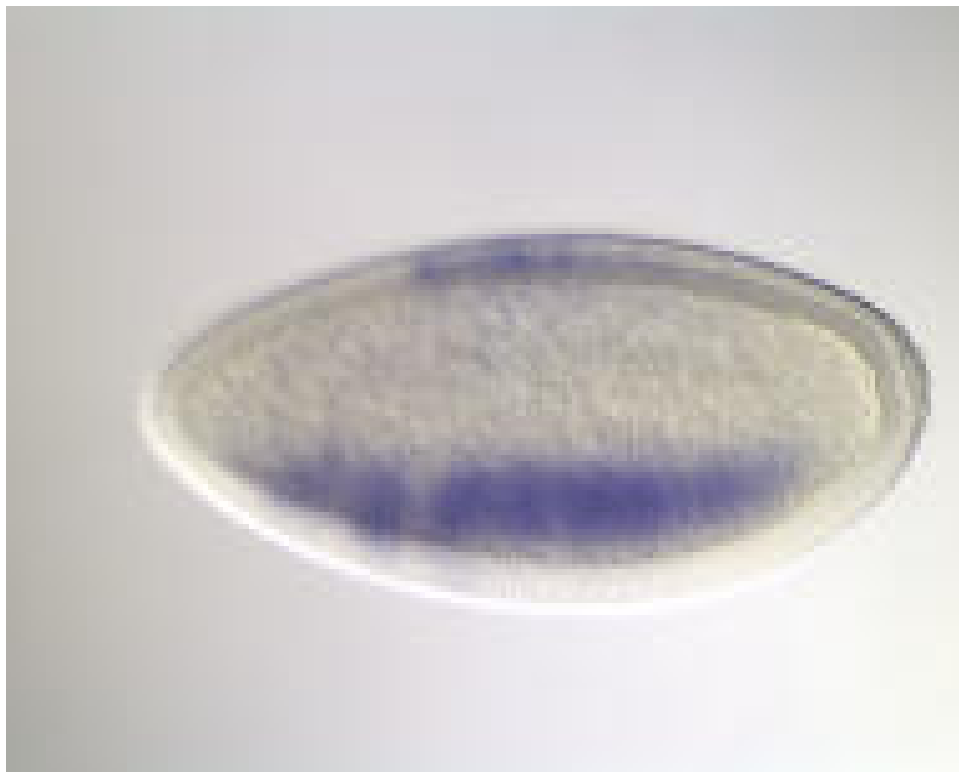

Location: Ventral Type: Promoter ZScore: 0.271119143 PValue: 0.786299398

Supplement: S3 File — Reports consist of in situ hybridization images, ATAC-seq traces, and calculated p-value and Z Score for each region used in the final analysis. (ZIP) [file pgen.1007367.s015.zip › S3_File/rho_Report.pdf]

rib

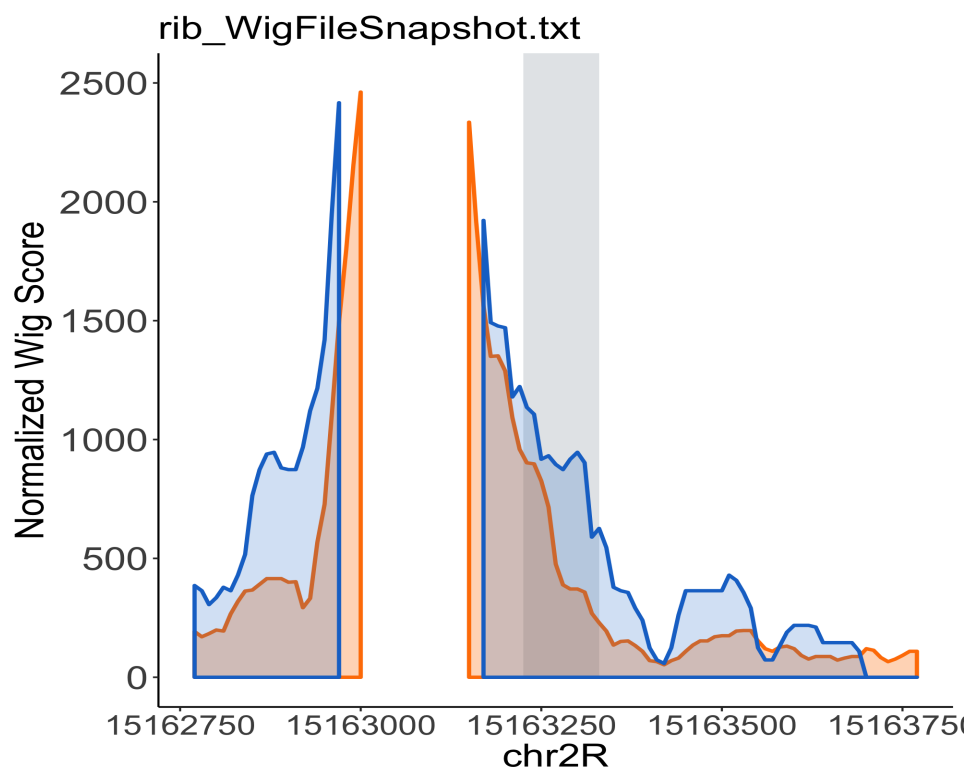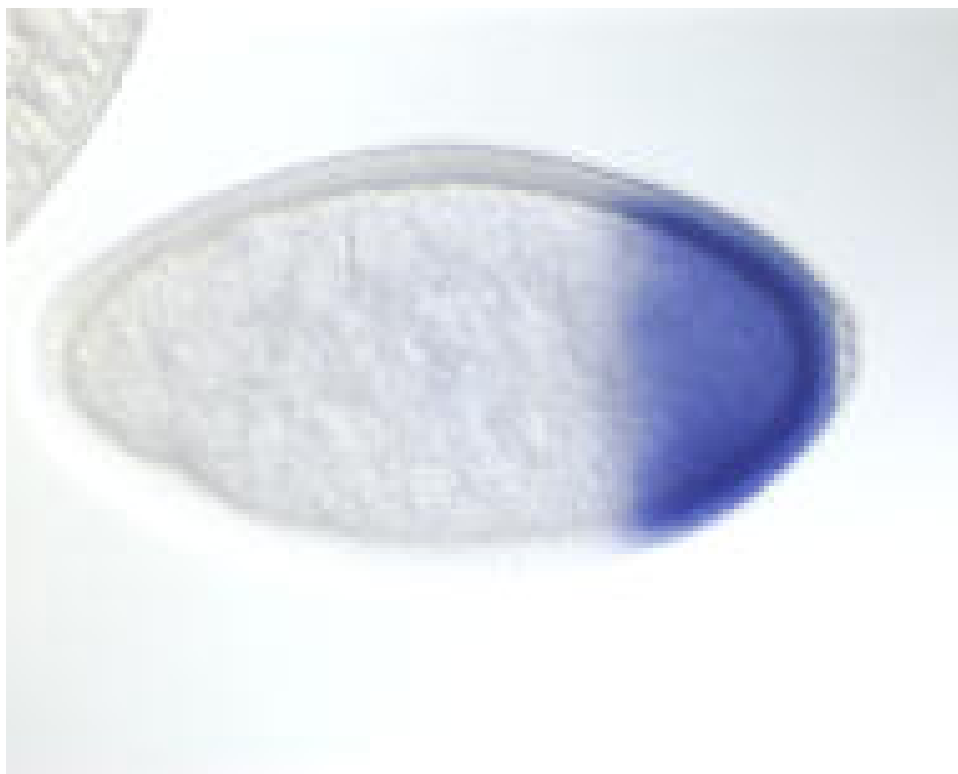

Location: Posterior Type: Promoter ZScore: 1.062408261 PValue: 0.288050385

Supplement: S3 File — Reports consist of in situ hybridization images, ATAC-seq traces, and calculated p-value and Z Score for each region used in the final analysis. (ZIP) [file pgen.1007367.s015.zip › S3_File/rib_Report.pdf]

RnrS

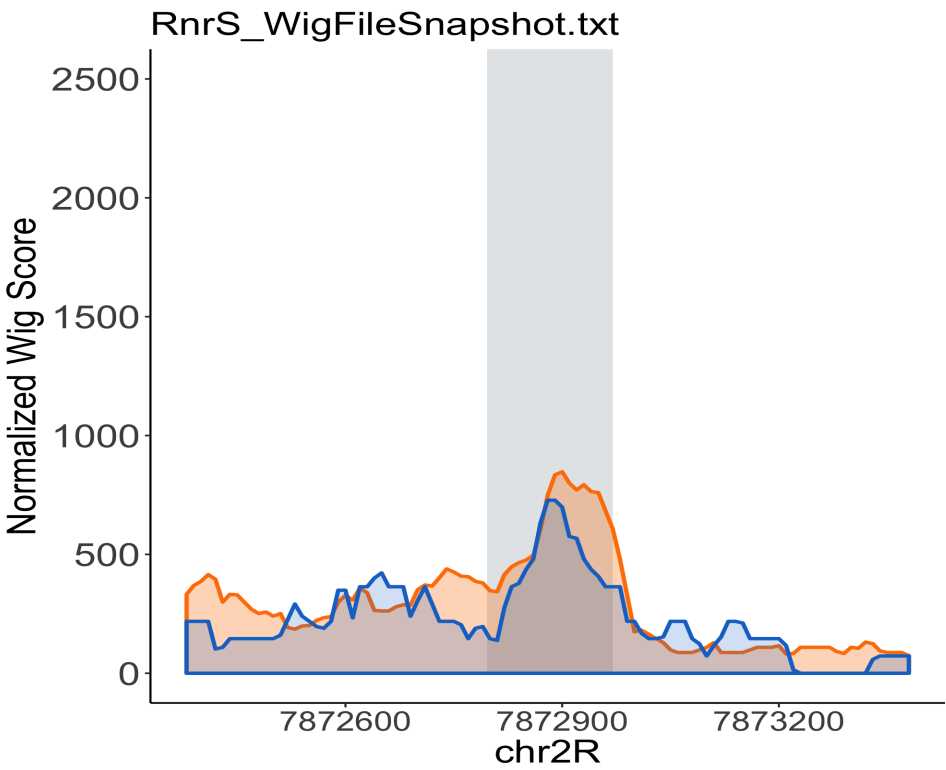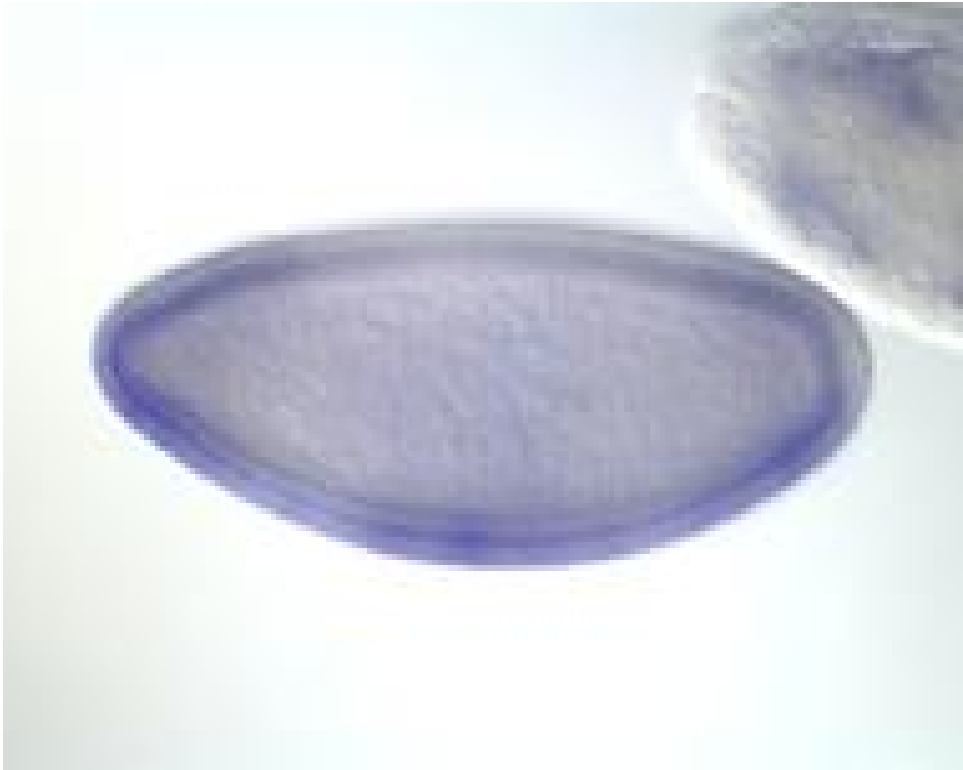

Location: Ventral Type: Promoter ZScore: 0.687073455 PValue: 0.492036441

Supplement: S3 File — Reports consist of in situ hybridization images, ATAC-seq traces, and calculated p-value and Z Score for each region used in the final analysis. (ZIP) [file pgen.1007367.s015.zip › S3_File/RnrS_Report.pdf]

run

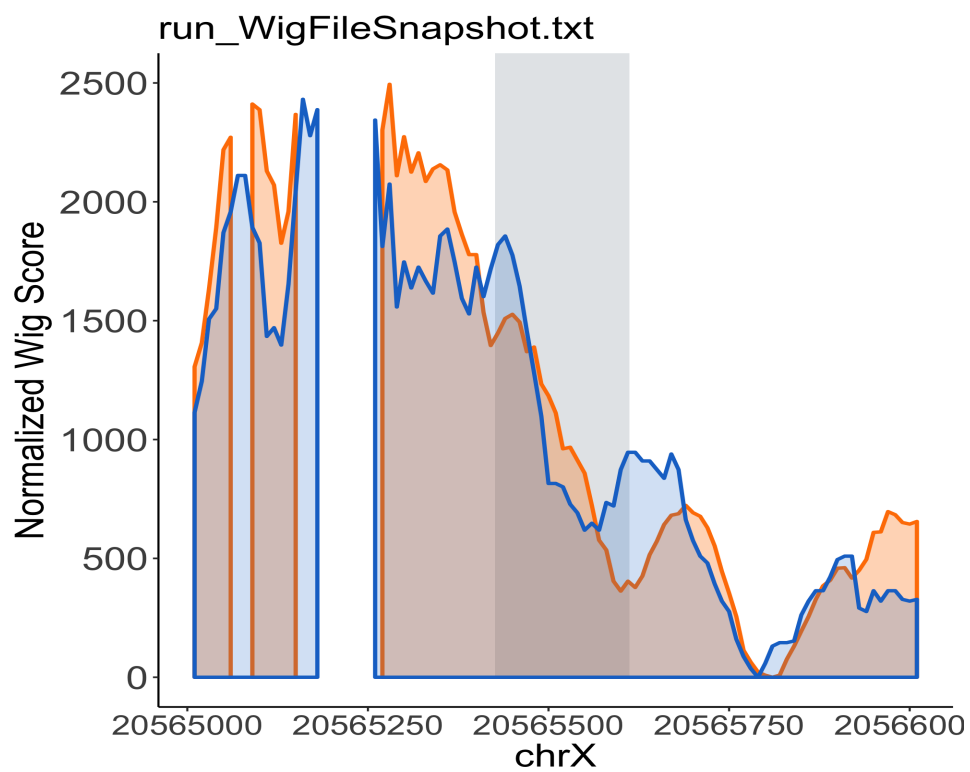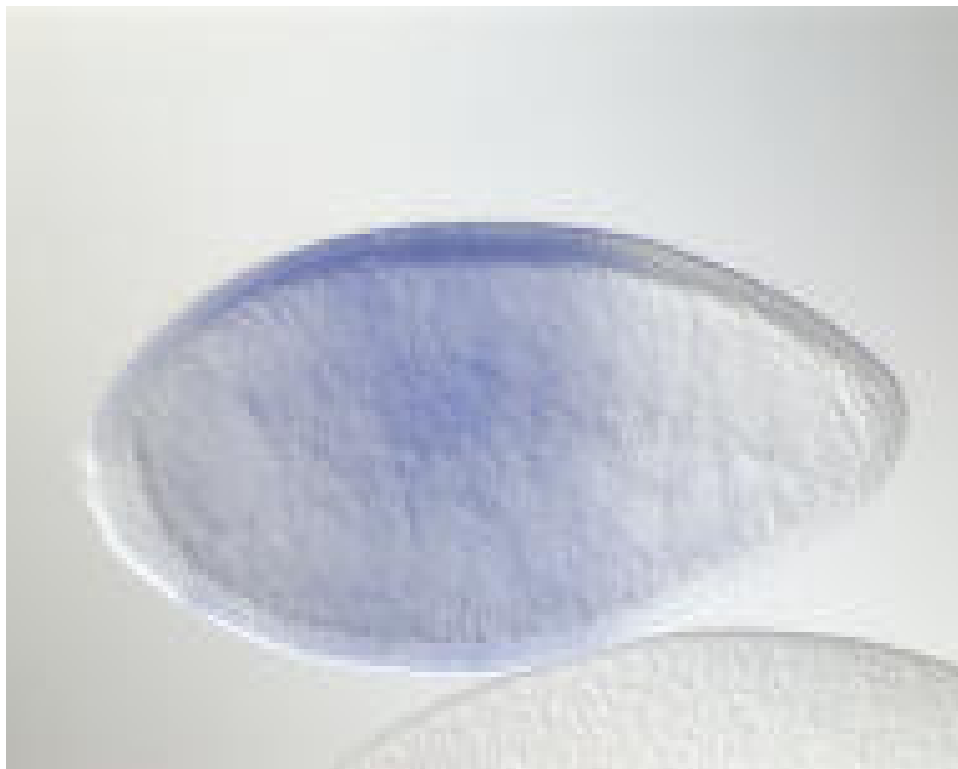

Location: Mostly Post Type: Promoter ZScore: 0.096425371 PValue: 0.923182743

Supplement: S3 File — Reports consist of in situ hybridization images, ATAC-seq traces, and calculated p-value and Z Score for each region used in the final analysis. (ZIP) [file pgen.1007367.s015.zip › S3_File/run_Report.pdf]

sad

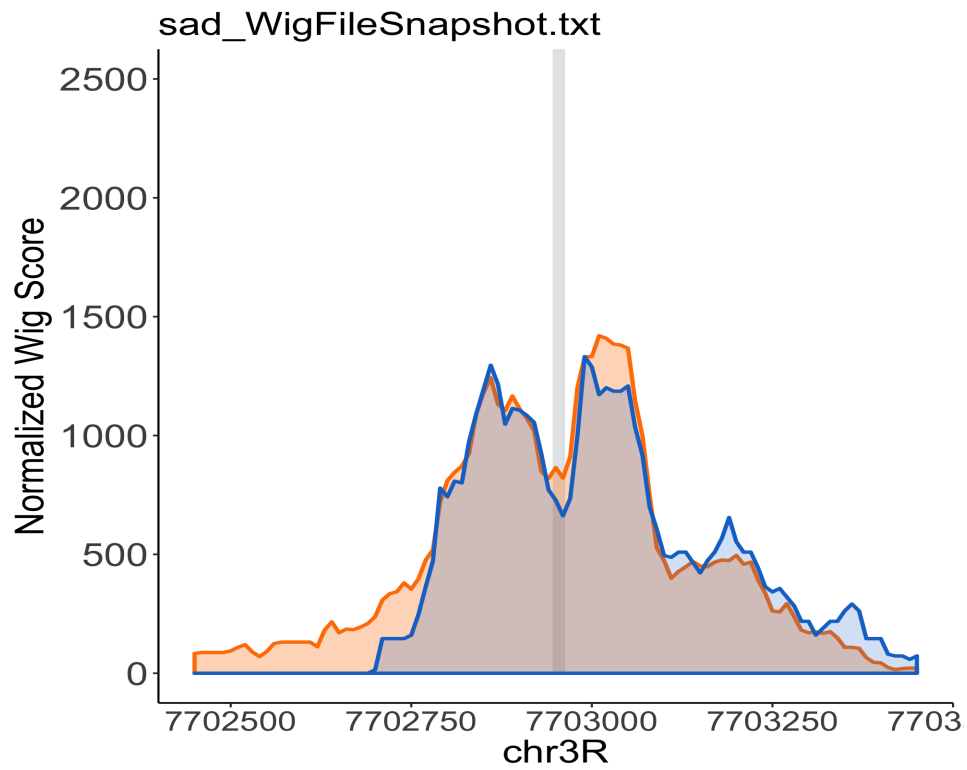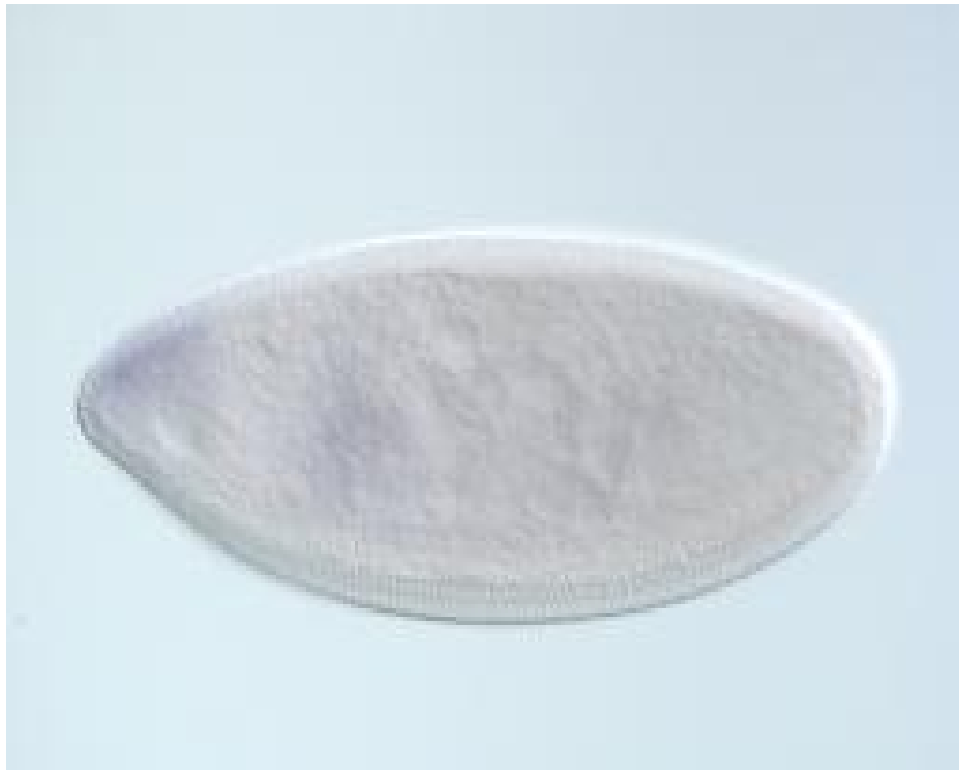

Location: Anterior Type: Promoter ZScore: 0.292699418 PValue: 0.769751915

Supplement: S3 File — Reports consist of in situ hybridization images, ATAC-seq traces, and calculated p-value and Z Score for each region used in the final analysis. (ZIP) [file pgen.1007367.s015.zip › S3_File/sad_Report.pdf]

sd

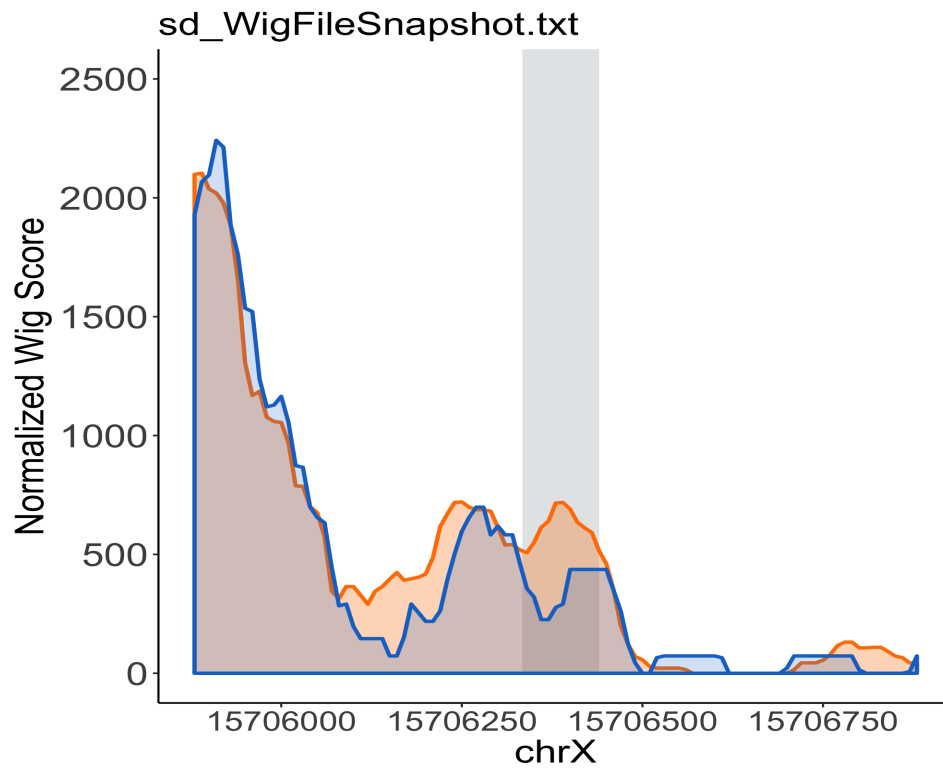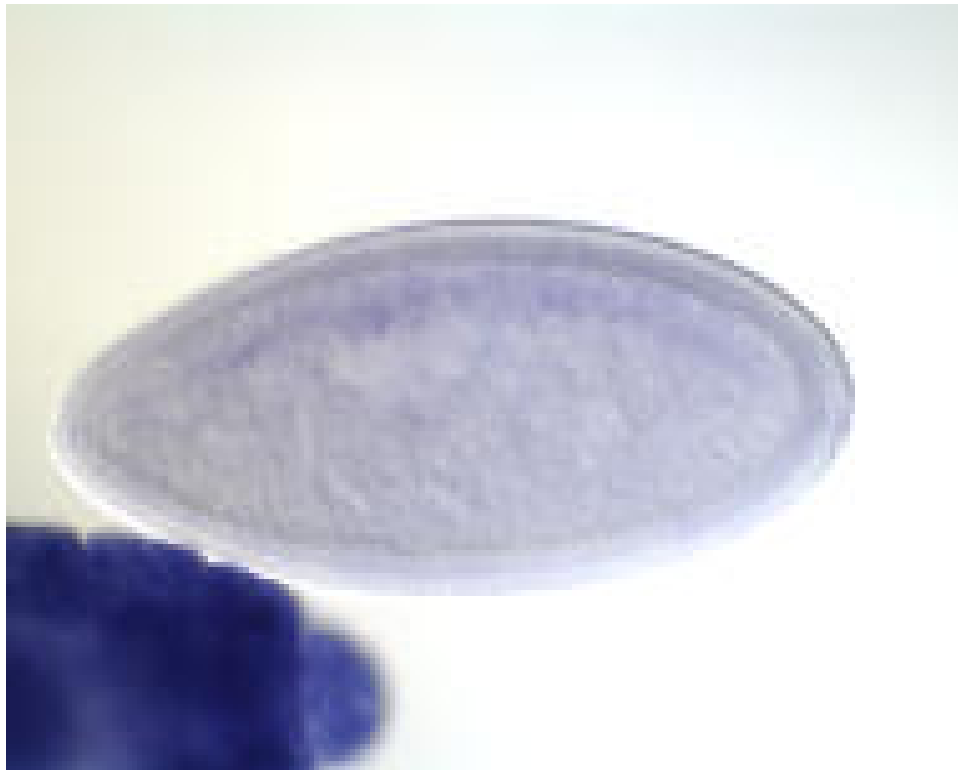

Location: Ventral Type: Promoter ZScore: 1.122890506 PValue: 0.261484

Supplement: S3 File — Reports consist of in situ hybridization images, ATAC-seq traces, and calculated p-value and Z Score for each region used in the final analysis. (ZIP) [file pgen.1007367.s015.zip › S3_File/sd_Report.pdf]

# Sec61beta

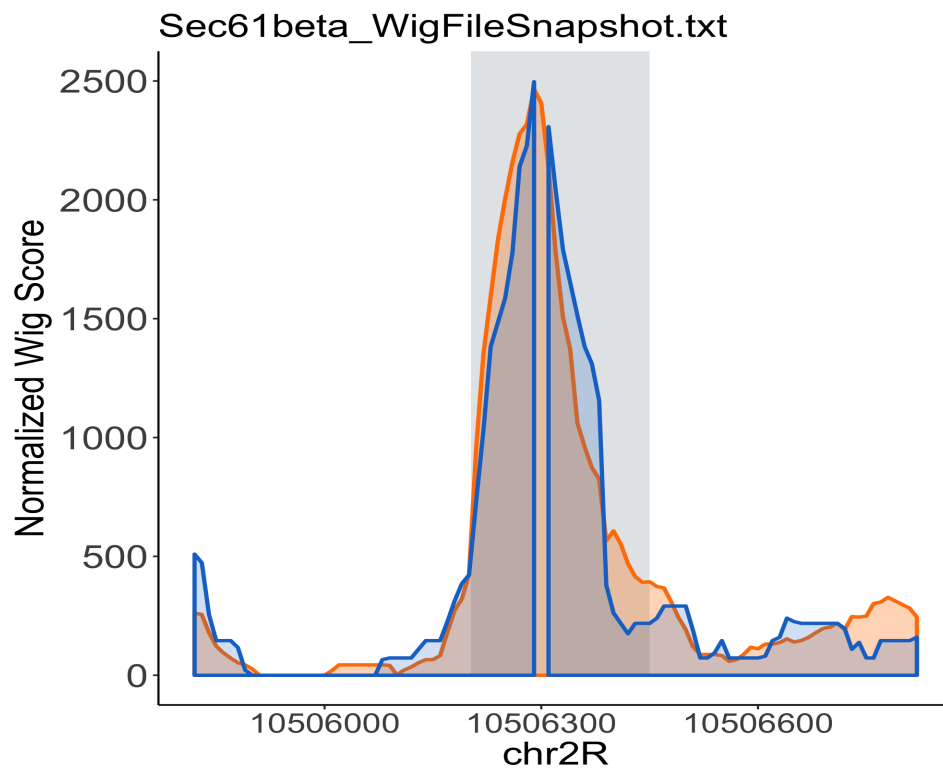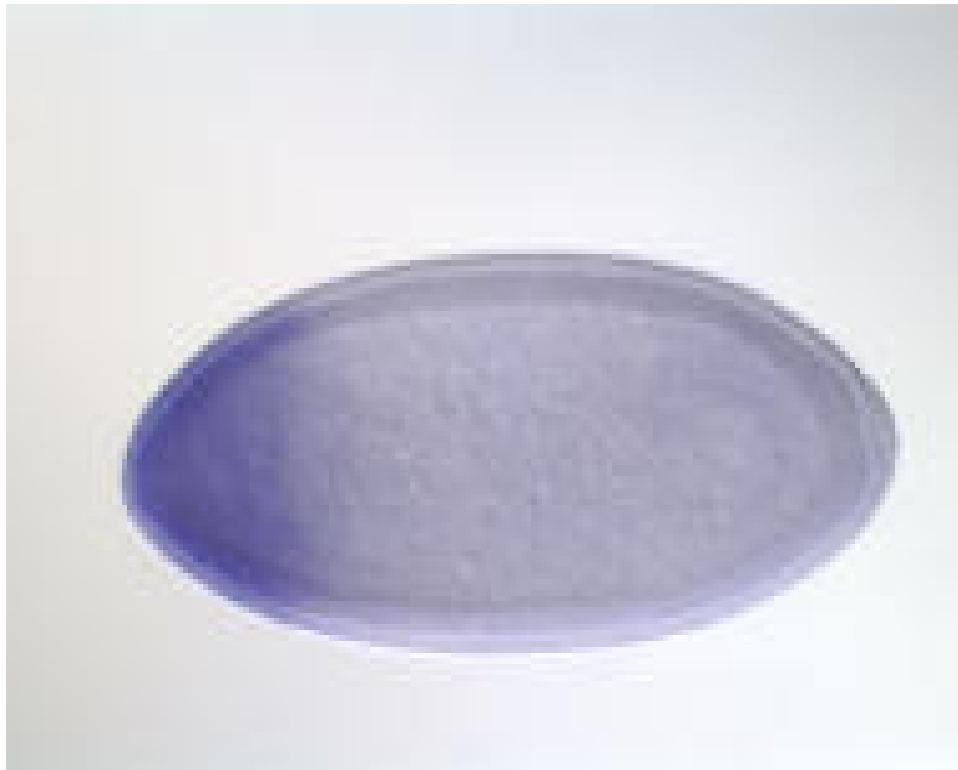

Location: Anterior Type: Promoter ZScore: 0.028699596 PValue: 0.977104179

Supplement: S3 File — Reports consist of in situ hybridization images, ATAC-seq traces, and calculated p-value and Z Score for each region used in the final analysis. (ZIP) [file pgen.1007367.s015.zip › S3_File/Sec61beta_Report.pdf]

sec63

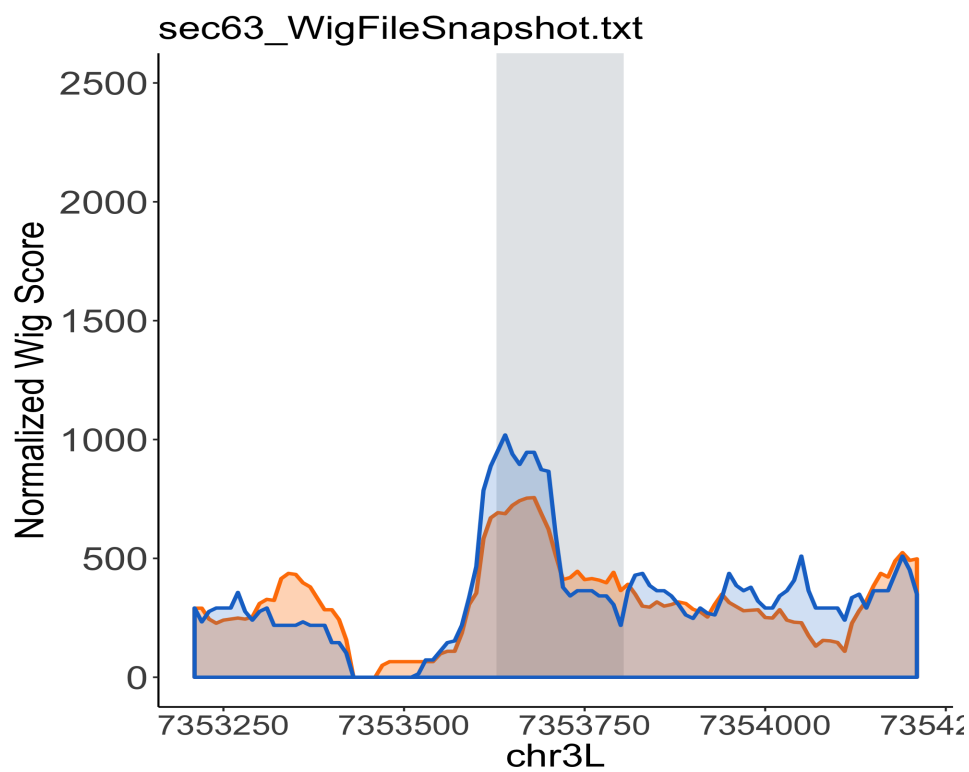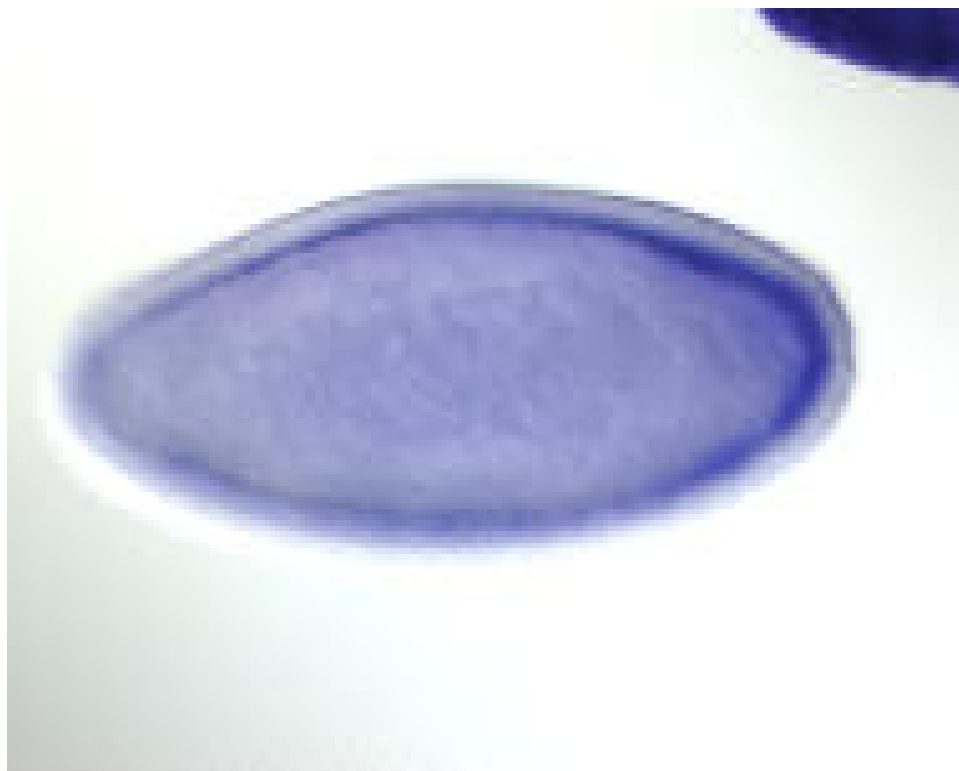

Location: Mostly Post Type: Promoter ZScore: 0.236944804 PValue: 0.812699611

Supplement: S3 File — Reports consist of in situ hybridization images, ATAC-seq traces, and calculated p-value and Z Score for each region used in the final analysis. (ZIP) [file pgen.1007367.s015.zip › S3_File/sec63_Report.pdf]

slp1

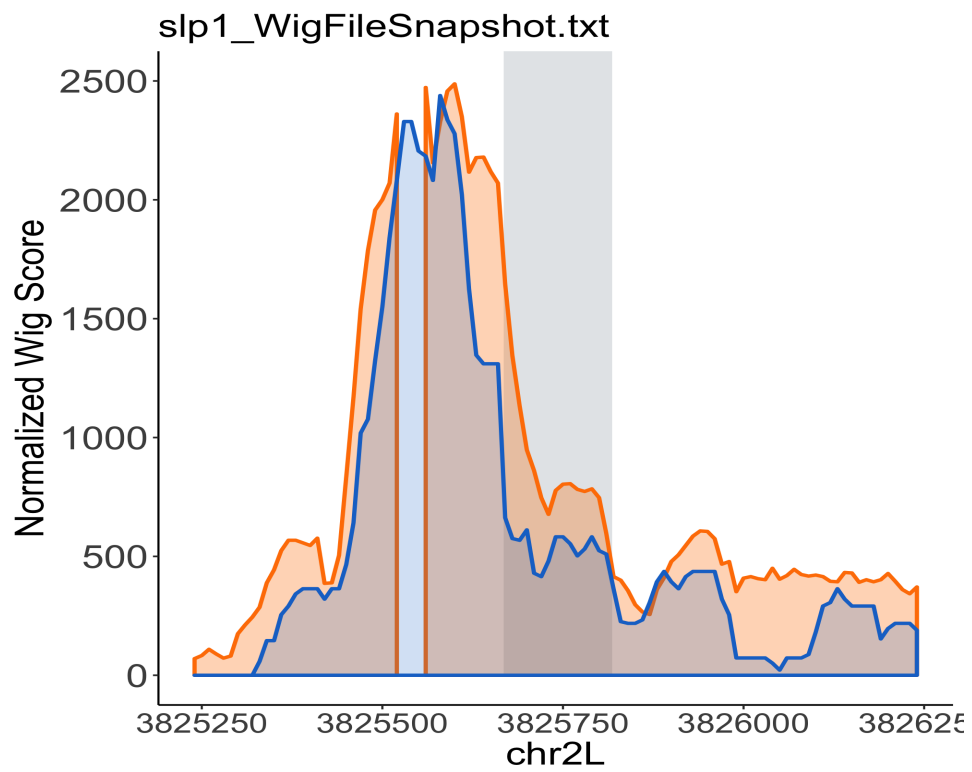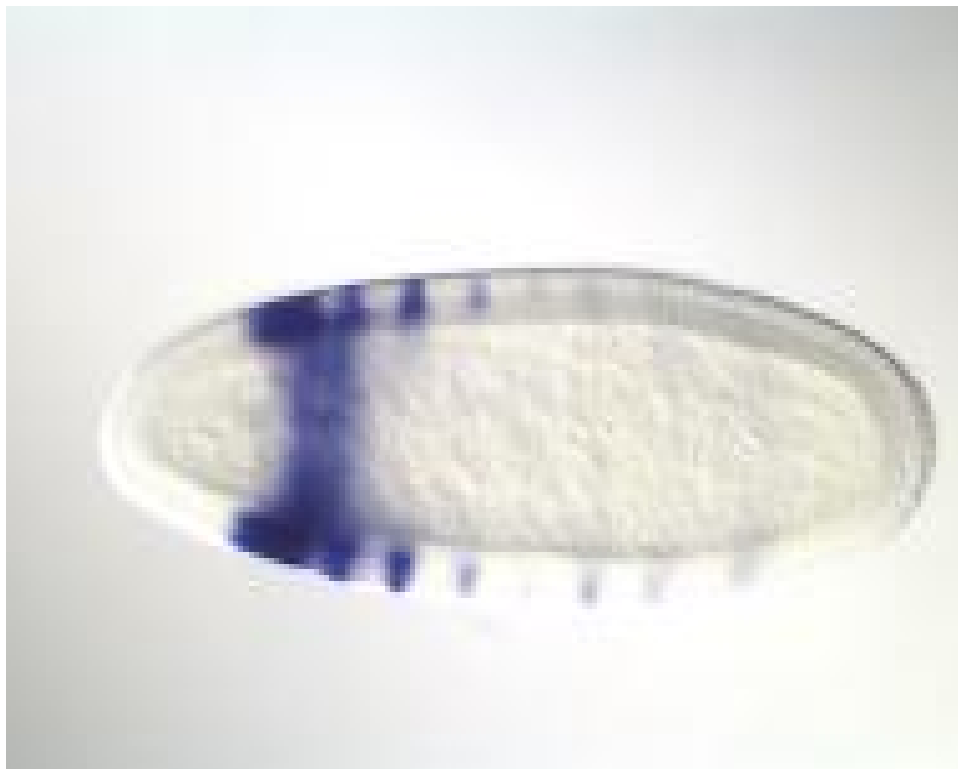

Location: Anterior Type: Promoter ZScore: 1.074670193 PValue: 0.282522413

Supplement: S3 File — Reports consist of in situ hybridization images, ATAC-seq traces, and calculated p-value and Z Score for each region used in the final analysis. (ZIP) [file pgen.1007367.s015.zip › S3_File/slp1_Report.pdf]

slp1\_slp\_B\_70

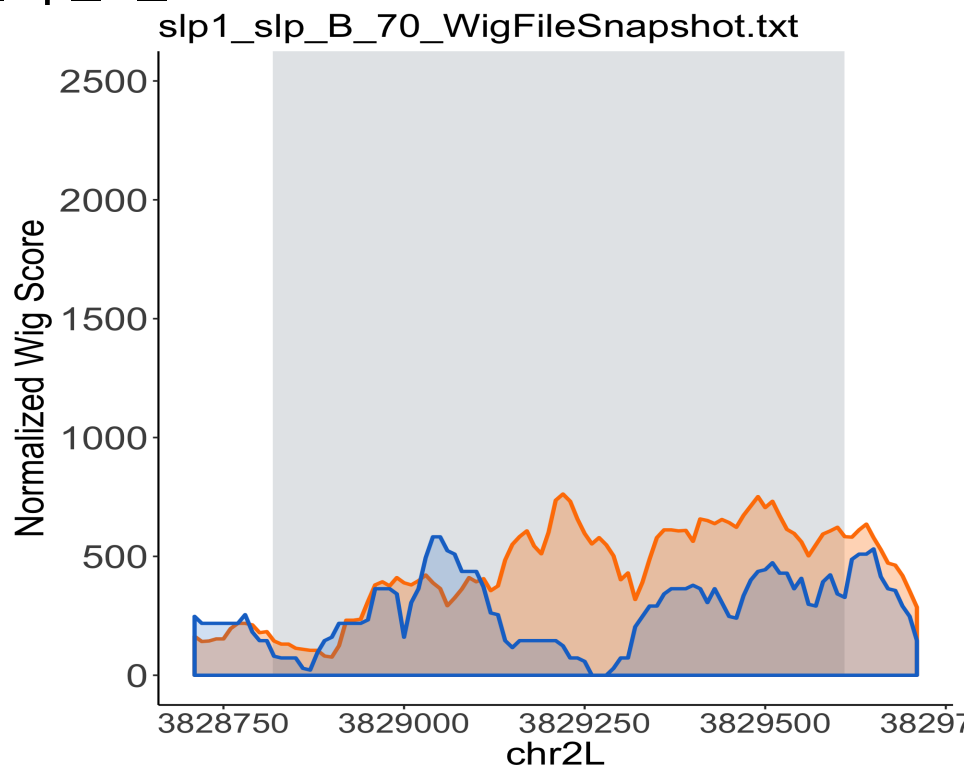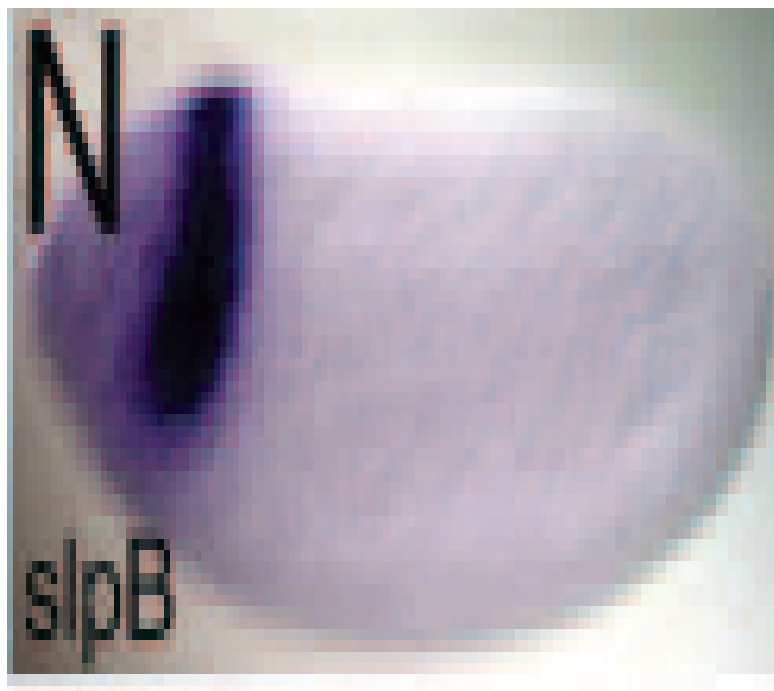

Location: Anterior Type: Enhancer ZScore: 1.231431386 PValue: 0.218161564

Supplement: S3 File — Reports consist of in situ hybridization images, ATAC-seq traces, and calculated p-value and Z Score for each region used in the final analysis. (ZIP) [file pgen.1007367.s015.zip › S3_File/slp1_slp_B_70_Report.pdf]

## slp2\_-3\_construct(70)

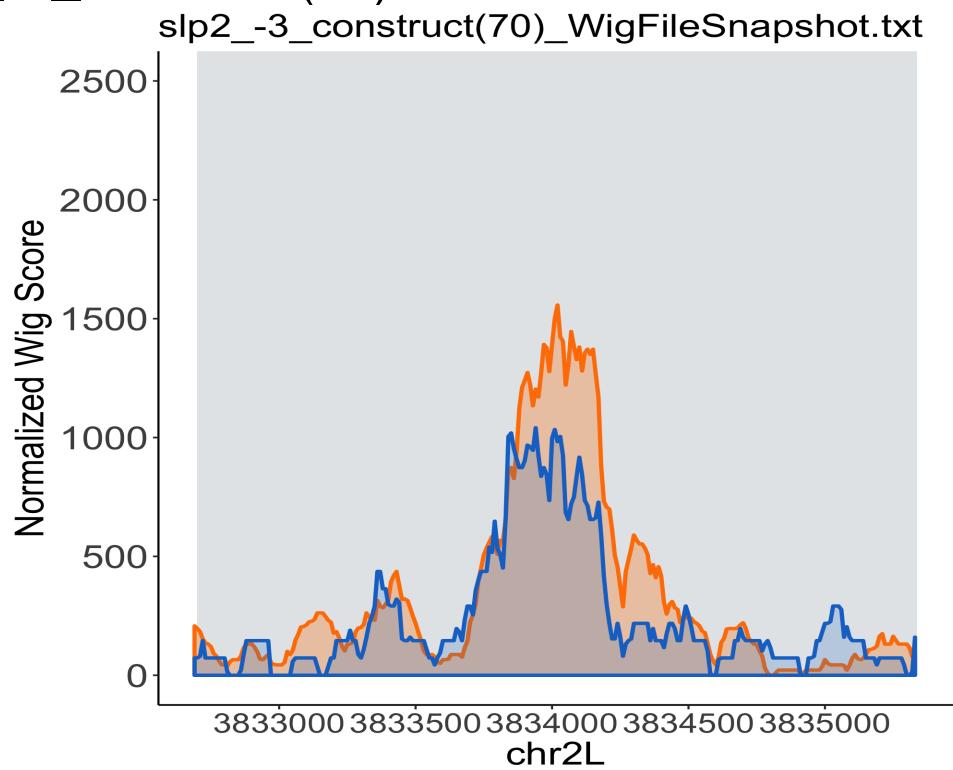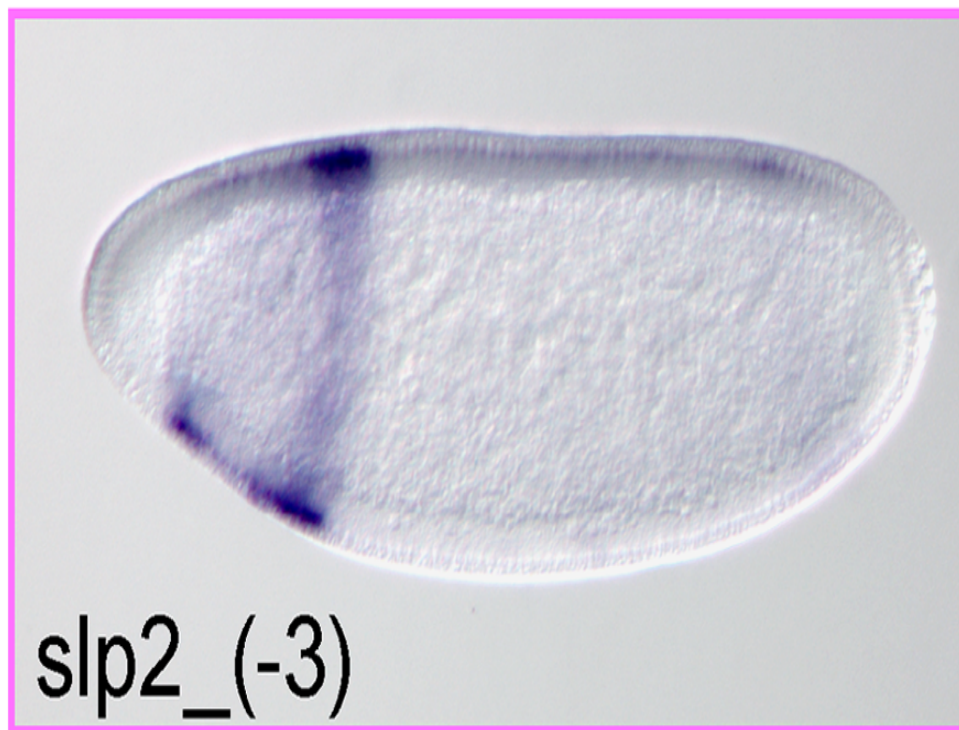

Location: Anterior Type: Enhancer ZScore: 0.6916821 PValue: 0.48913699

Supplement: S3 File — Reports consist of in situ hybridization images, ATAC-seq traces, and calculated p-value and Z Score for each region used in the final analysis. (ZIP) [file pgen.1007367.s015.zip › S3_File/slp2_-3_construct(70)_Report.pdf]

sna\_ip

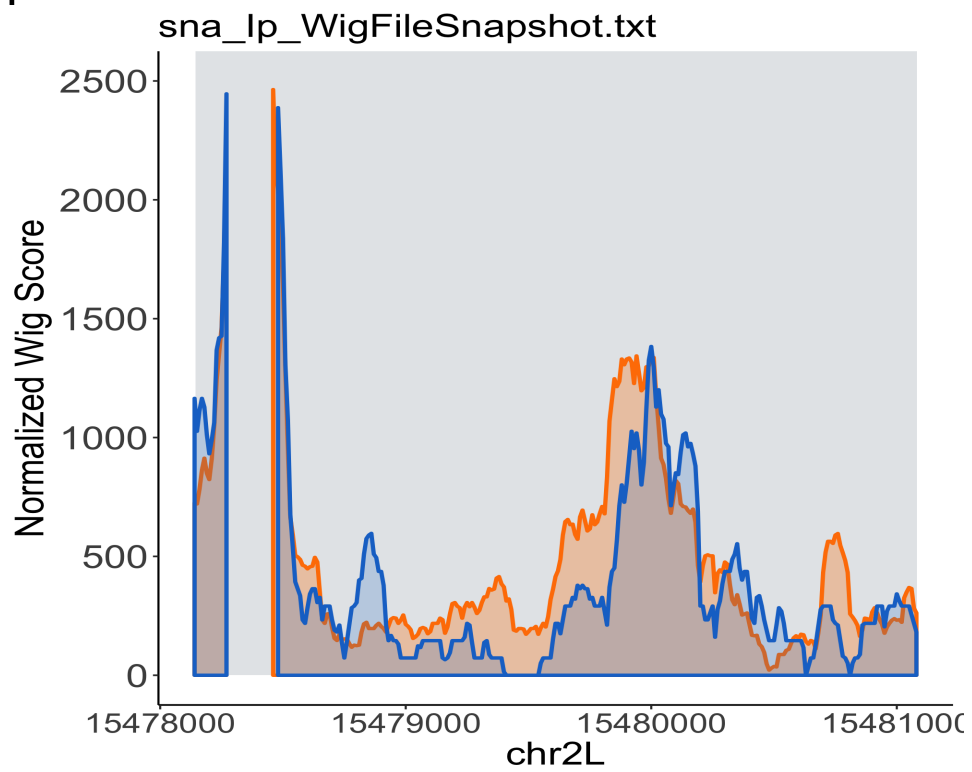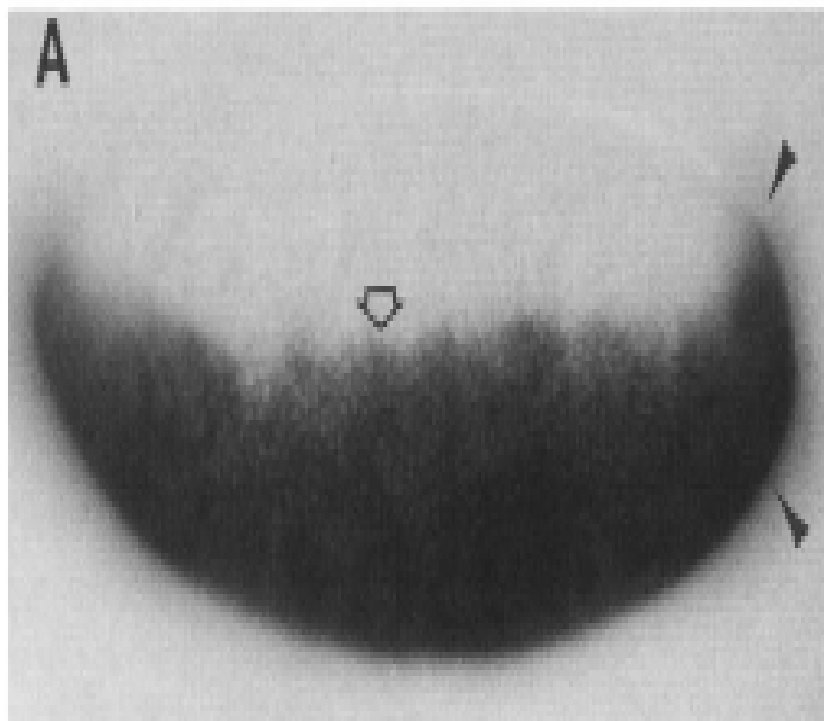

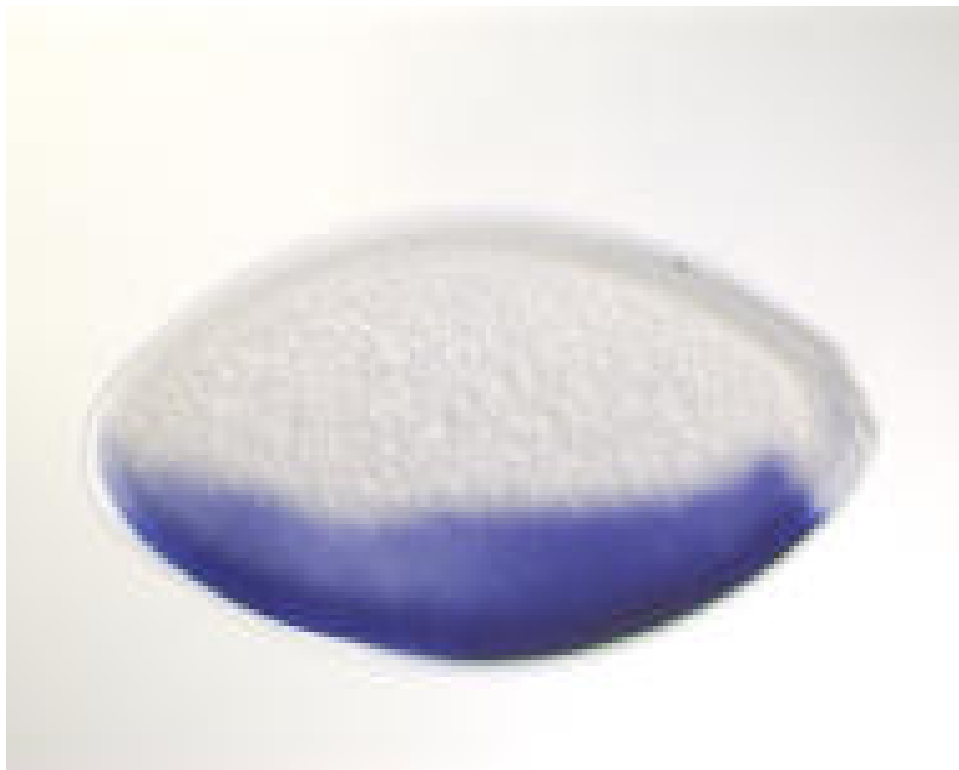

Location: Ventral Type: Enhancer ZScore: 0.150202567 PValue: 0.8806048

Supplement: S3 File — Reports consist of in situ hybridization images, ATAC-seq traces, and calculated p-value and Z Score for each region used in the final analysis. (ZIP) [file pgen.1007367.s015.zip › S3_File/sna_Ip_Report.pdf]

sna

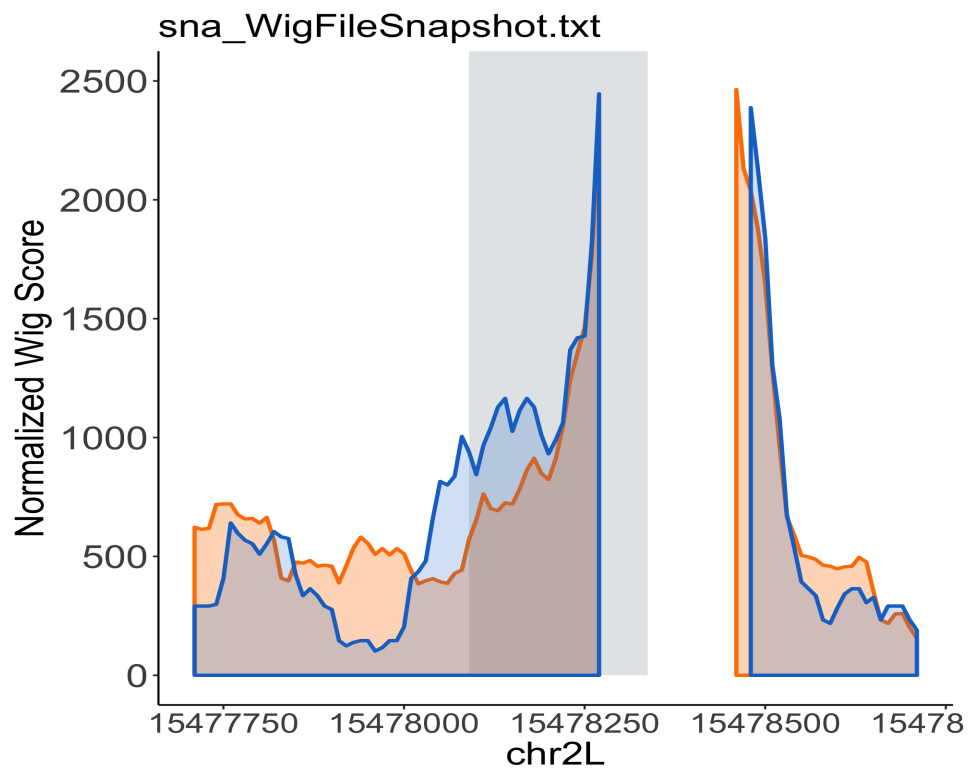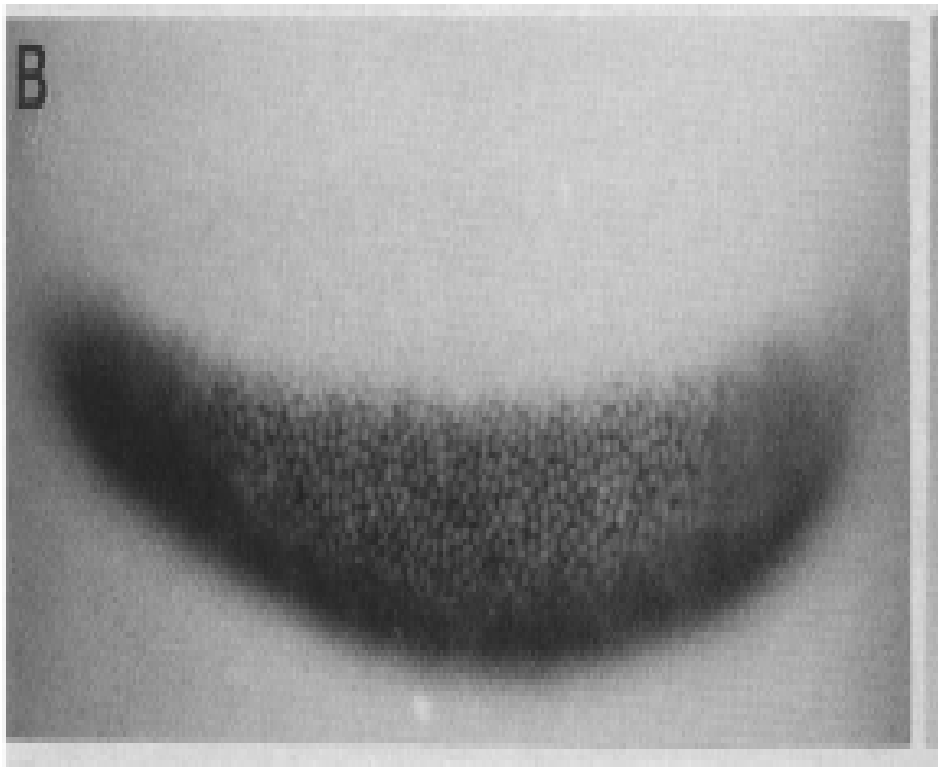

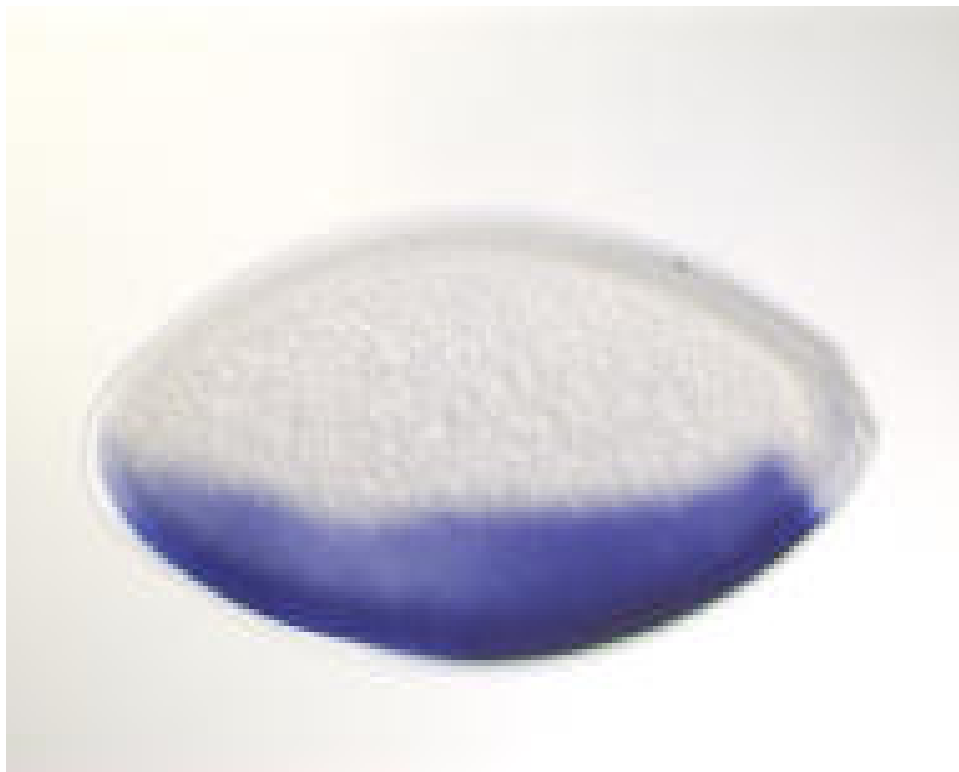

Location: Ventral Type: Promoter ZScore: -0.305394639 PValue: 0.760065604

Supplement: S3 File — Reports consist of in situ hybridization images, ATAC-seq traces, and calculated p-value and Z Score for each region used in the final analysis. (ZIP) [file pgen.1007367.s015.zip › S3_File/sna_Report.pdf]

sog

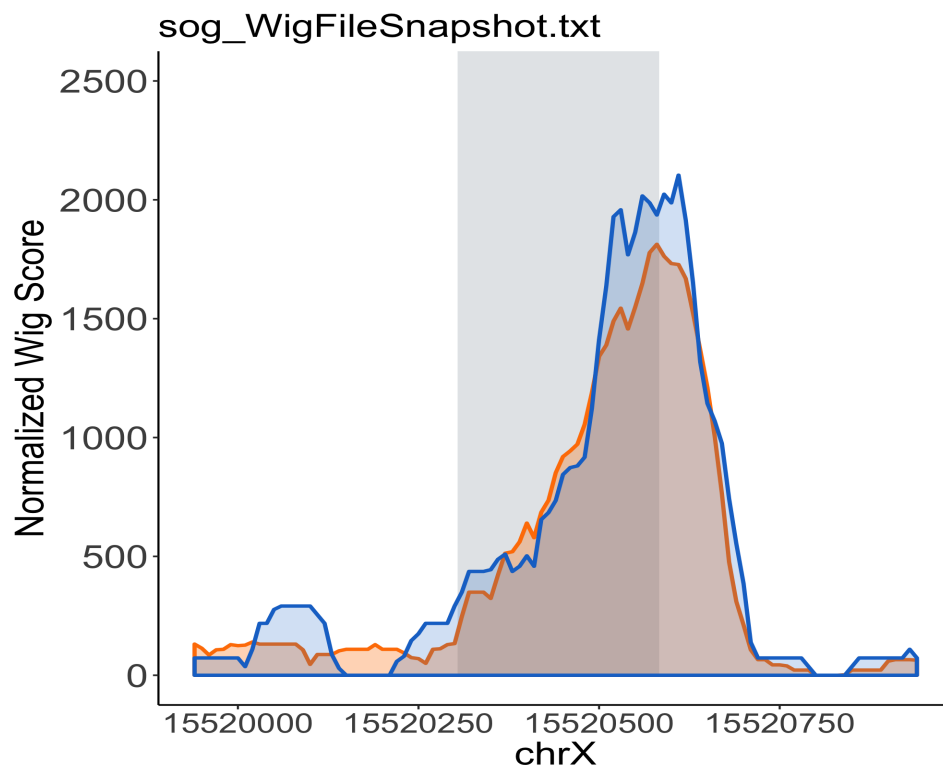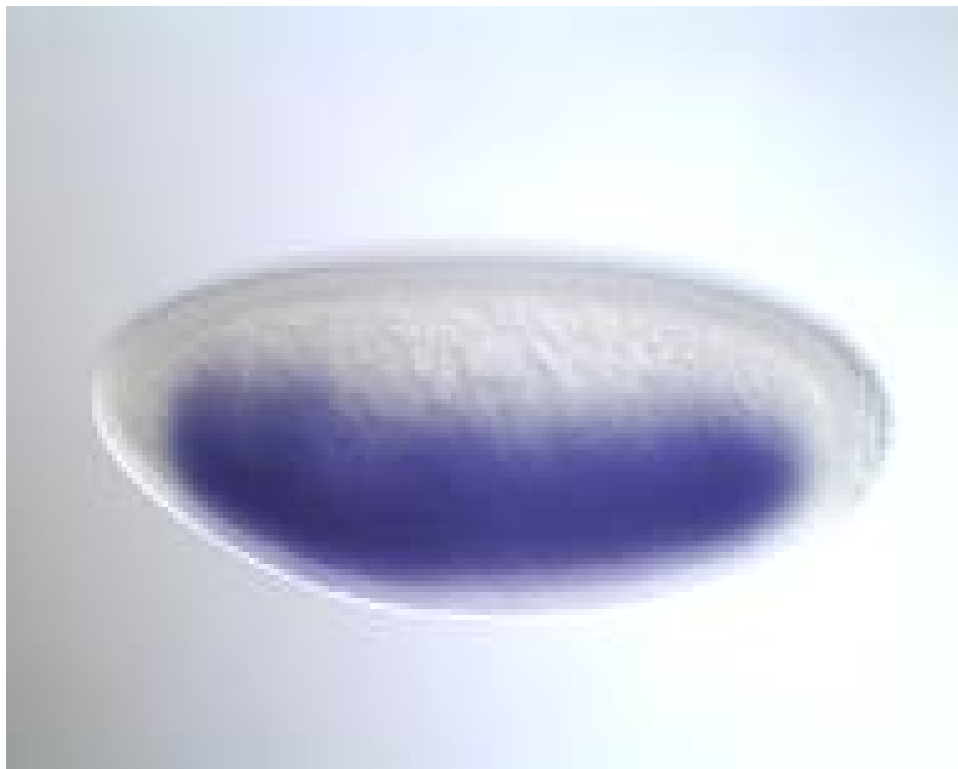

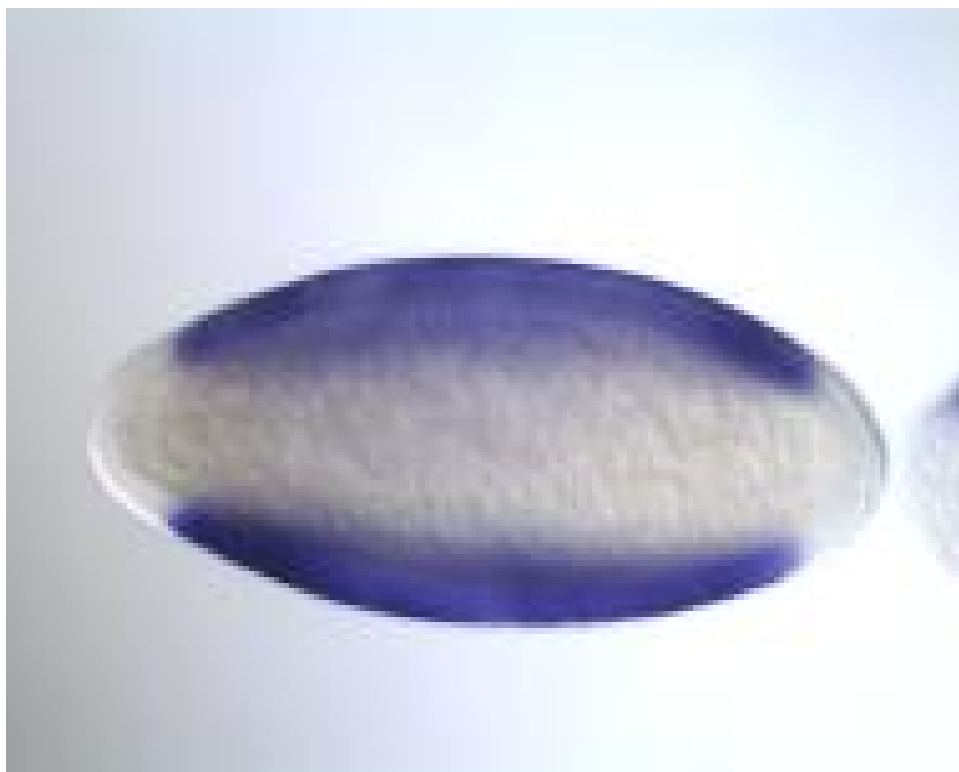

Location: Ventral Type: Promoter ZScore: -0.222797964 PValue: 0.823692752

Supplement: S3 File — Reports consist of in situ hybridization images, ATAC-seq traces, and calculated p-value and Z Score for each region used in the final analysis. (ZIP) [file pgen.1007367.s015.zip › S3_File/sog_Report.pdf]

SoxN

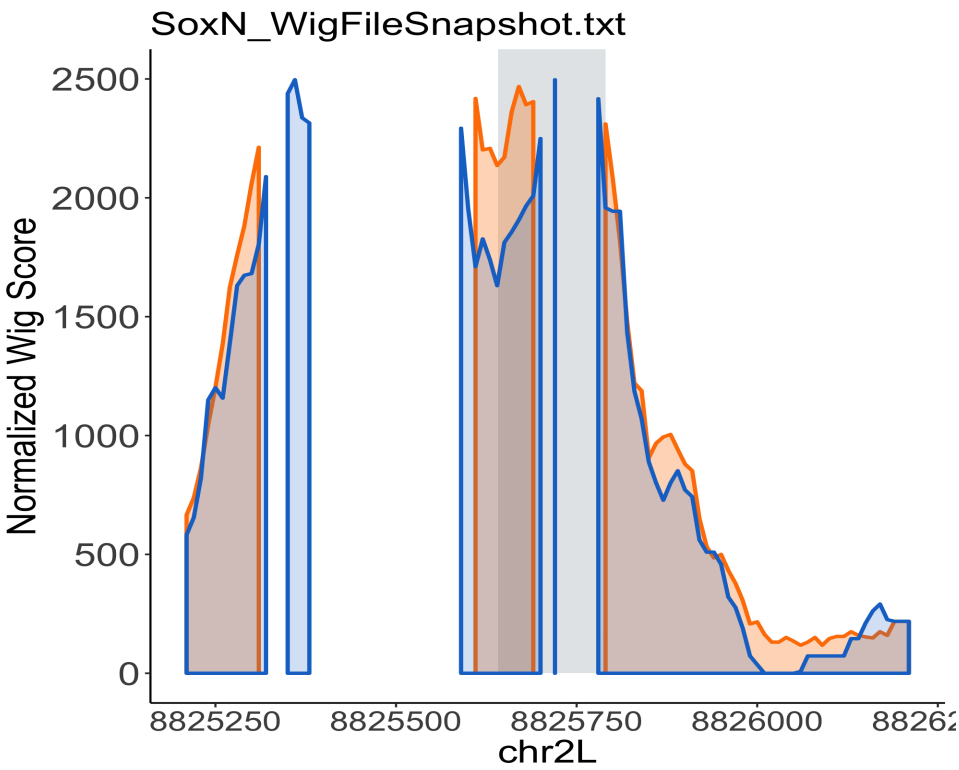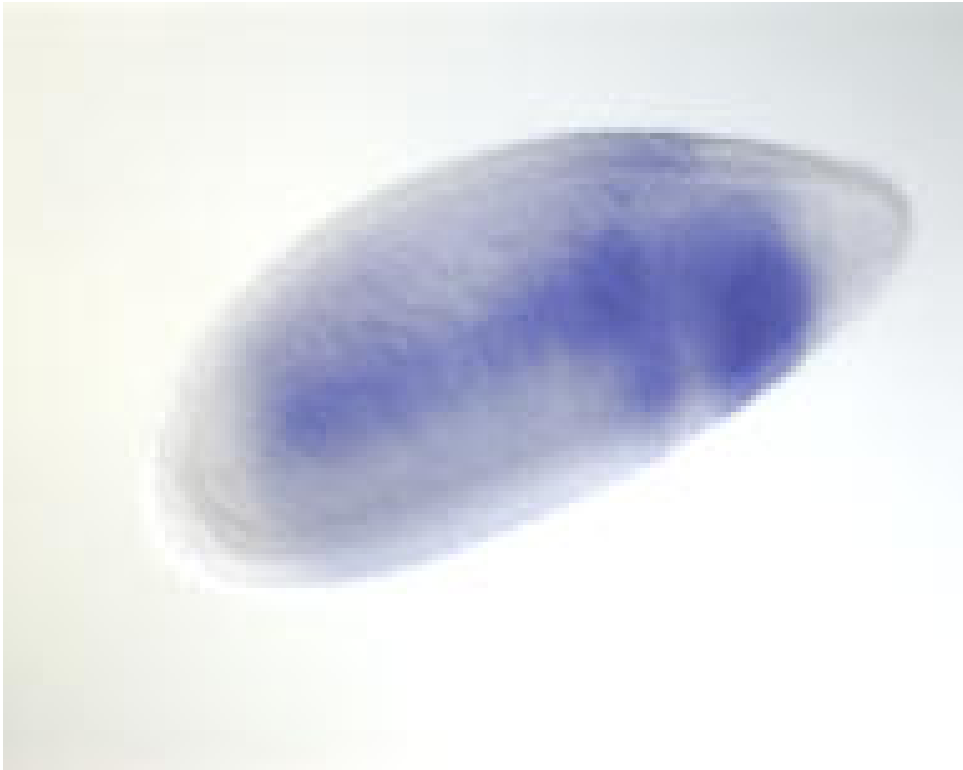

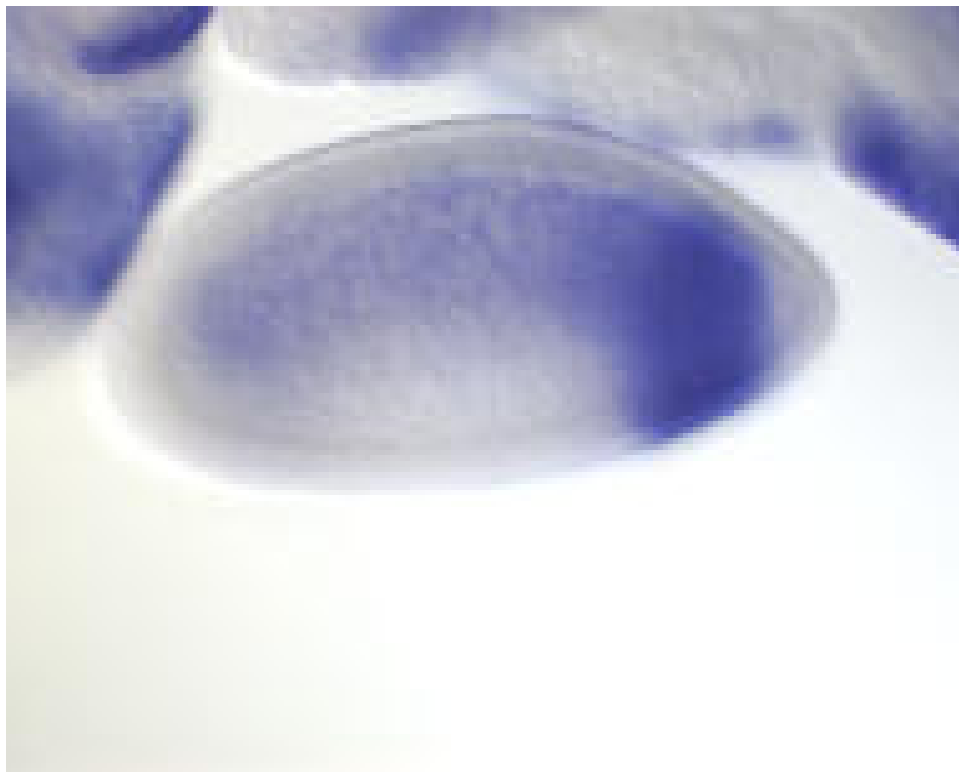

Location: Dorsal Type: Promoter ZScore: 0.274499075 PValue: 0.783701115

Supplement: S3 File — Reports consist of in situ hybridization images, ATAC-seq traces, and calculated p-value and Z Score for each region used in the final analysis. (ZIP) [file pgen.1007367.s015.zip › S3_File/SoxN_Report.pdf]

spri

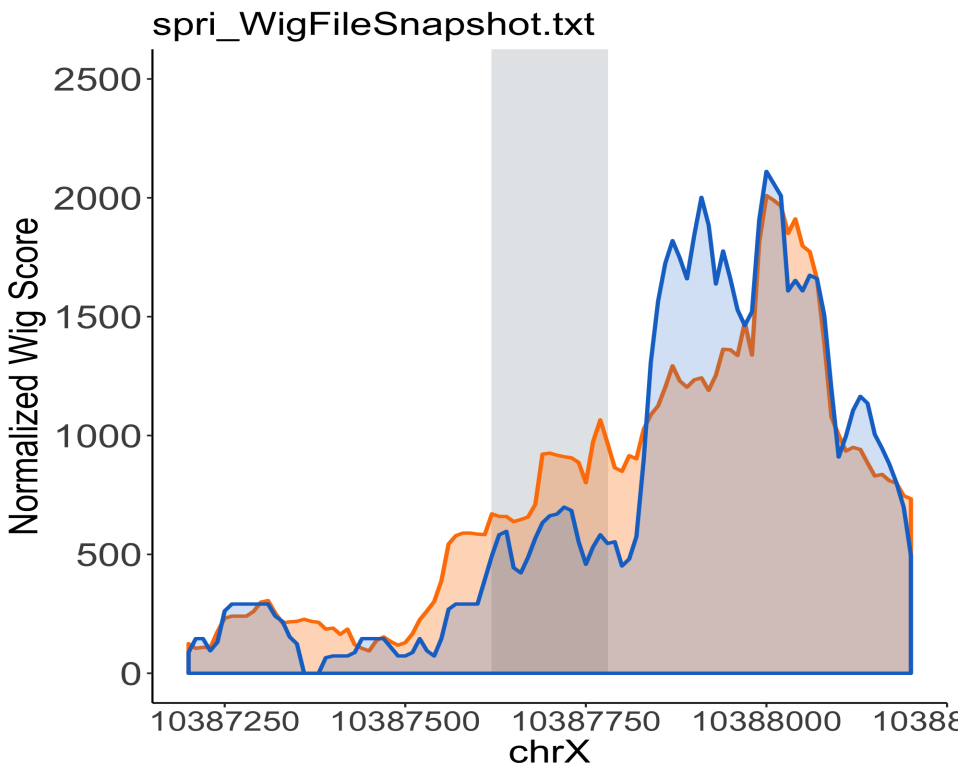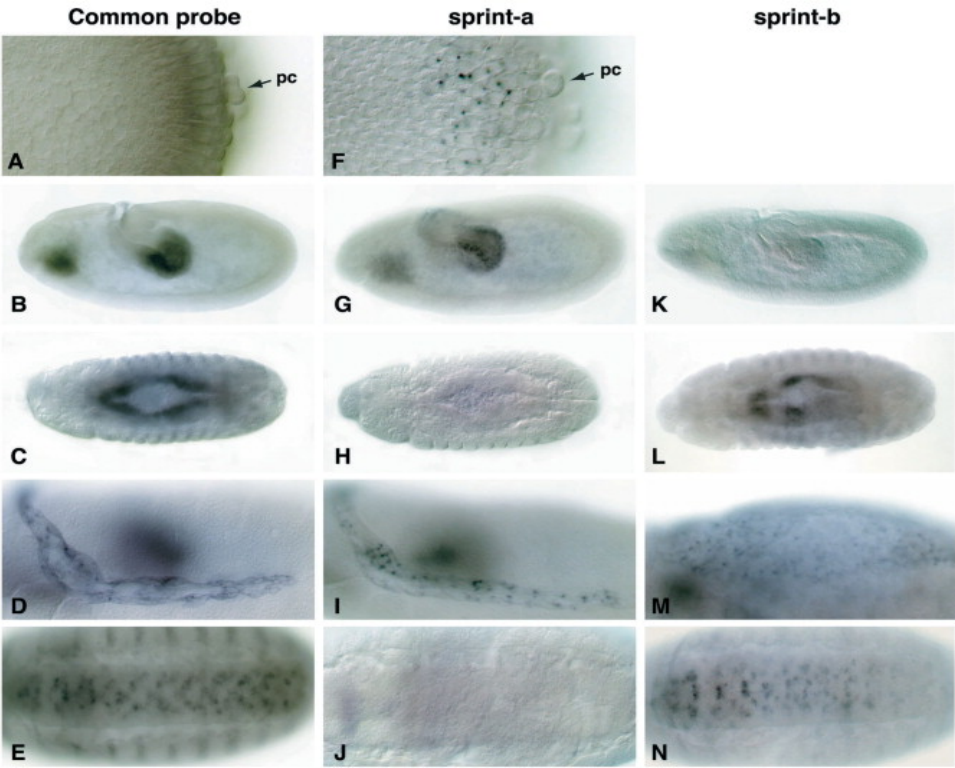

Location: Posterior Type: Promoter ZScore: -0.887018569 PValue: 0.375068903

Supplement: S3 File — Reports consist of in situ hybridization images, ATAC-seq traces, and calculated p-value and Z Score for each region used in the final analysis. (ZIP) [file pgen.1007367.s015.zip › S3_File/spri_Report.pdf]

## stumps\_Sandmann

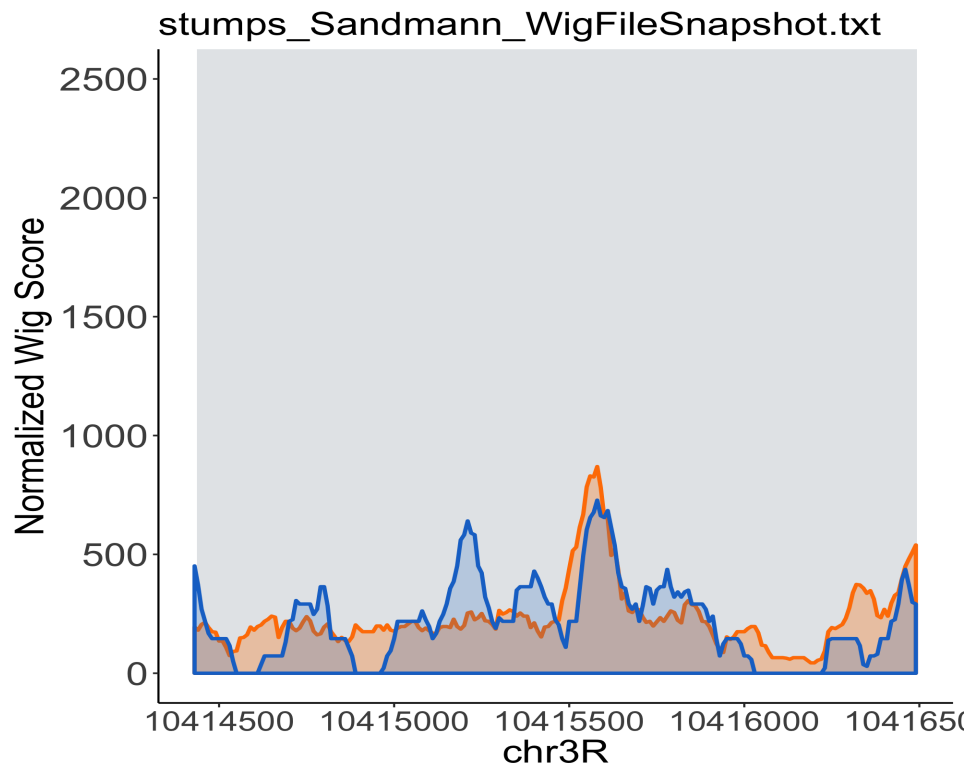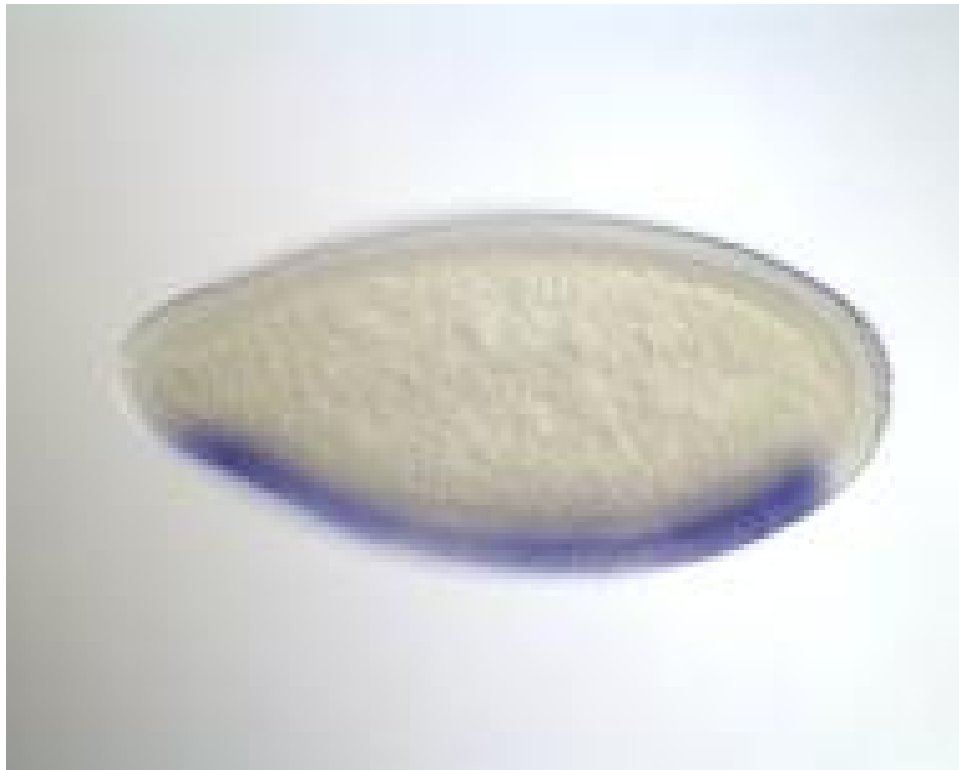

Location: Ventral Type: Enhancer ZScore: 0.147777978 PValue: 0.882517989

Supplement: S3 File — Reports consist of in situ hybridization images, ATAC-seq traces, and calculated p-value and Z Score for each region used in the final analysis. (ZIP) [file pgen.1007367.s015.zip › S3_File/stumps_Sandmann_Report.pdf]

## T48\_Sandmann

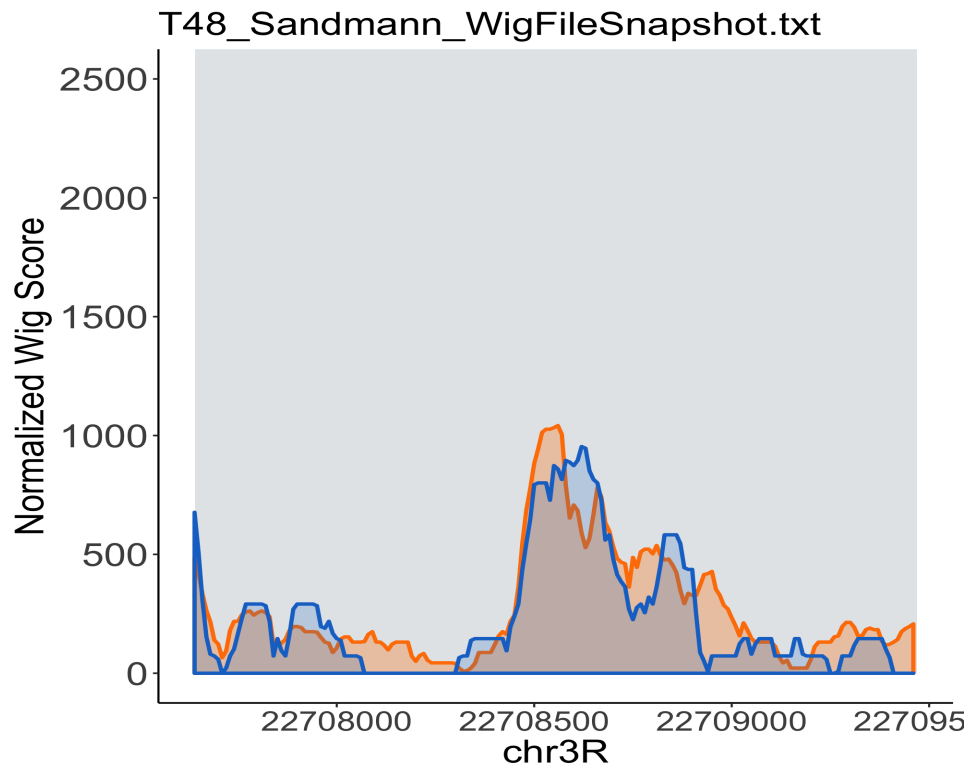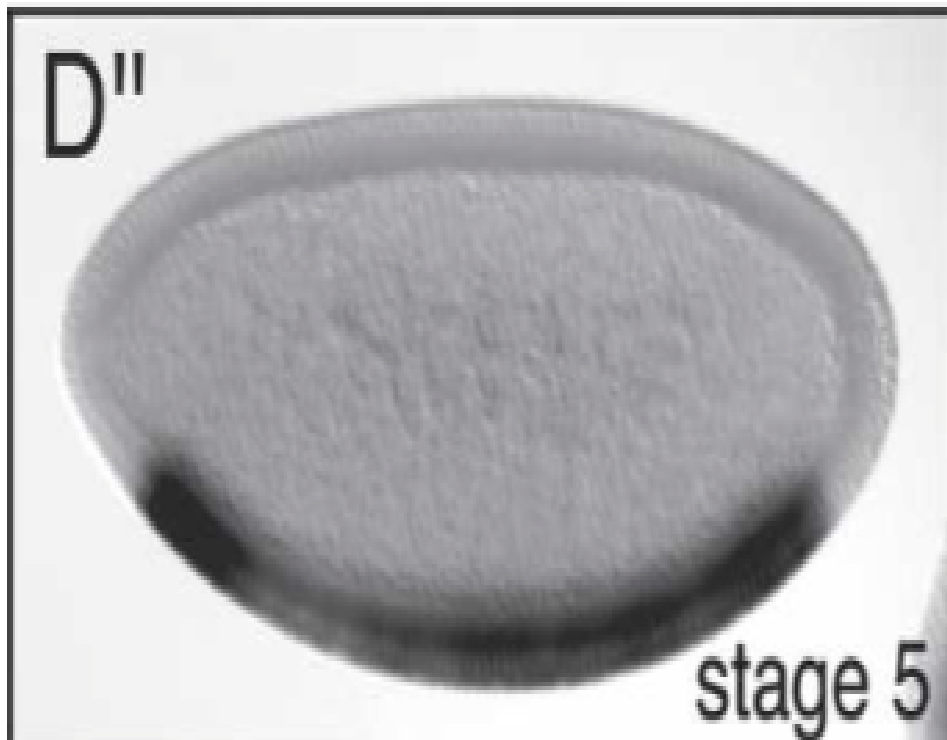

Location: Ventral Type: Enhancer ZScore: 0.327397819 PValue: 0.743367016

Supplement: S3 File — Reports consist of in situ hybridization images, ATAC-seq traces, and calculated p-value and Z Score for each region used in the final analysis. (ZIP) [file pgen.1007367.s015.zip › S3_File/T48_Sandmann_Report.pdf]

tin

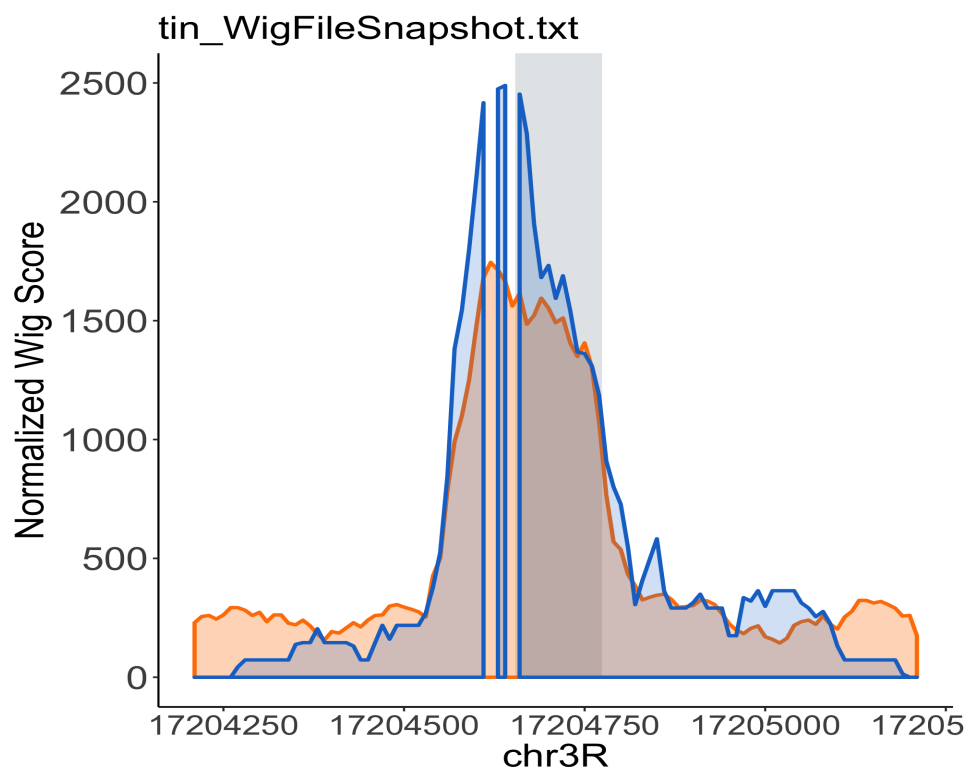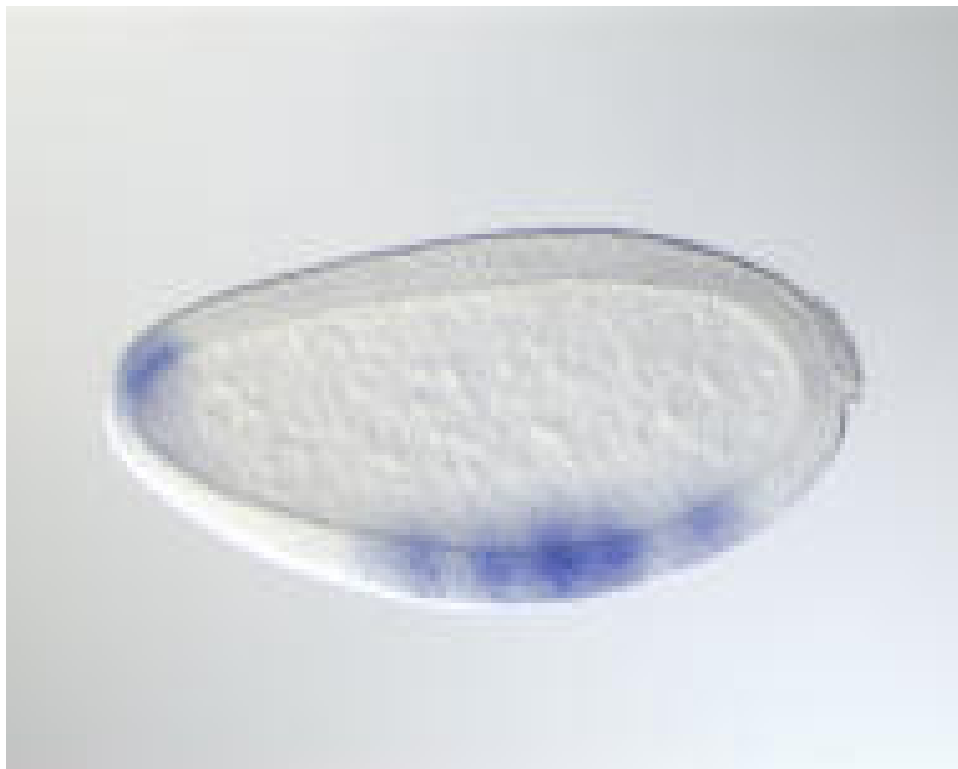

Location: Ventral Type: Promoter ZScore: -0.462036359 PValue: 0.644055247

Supplement: S3 File — Reports consist of in situ hybridization images, ATAC-seq traces, and calculated p-value and Z Score for each region used in the final analysis. (ZIP) [file pgen.1007367.s015.zip › S3_File/tin_Report.pdf]

tin\_Yin

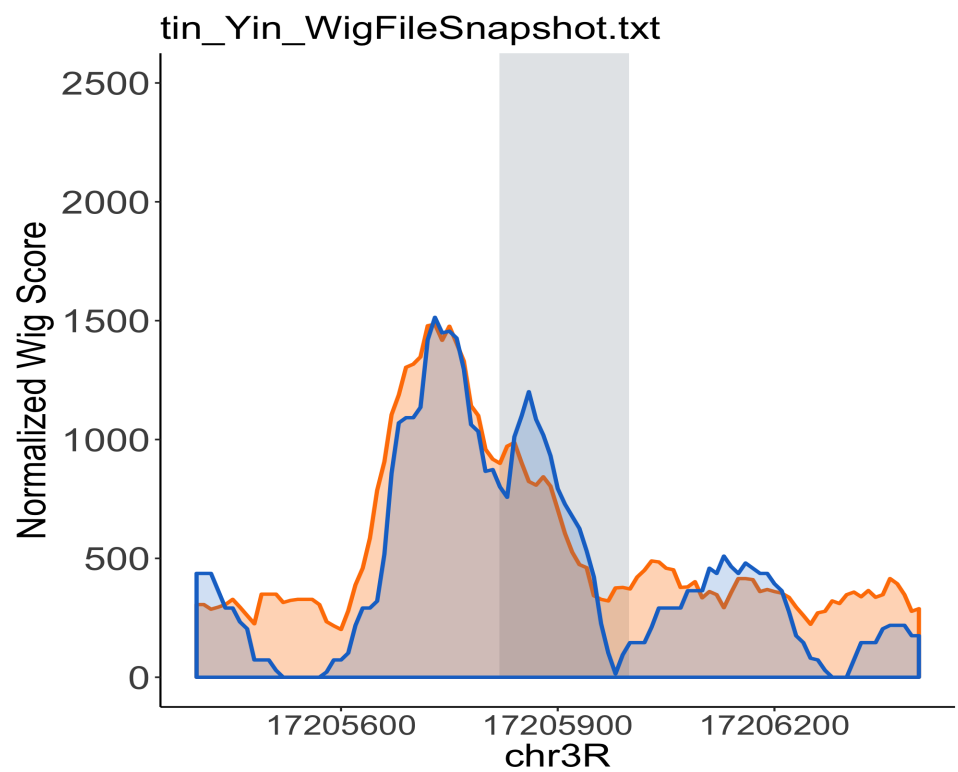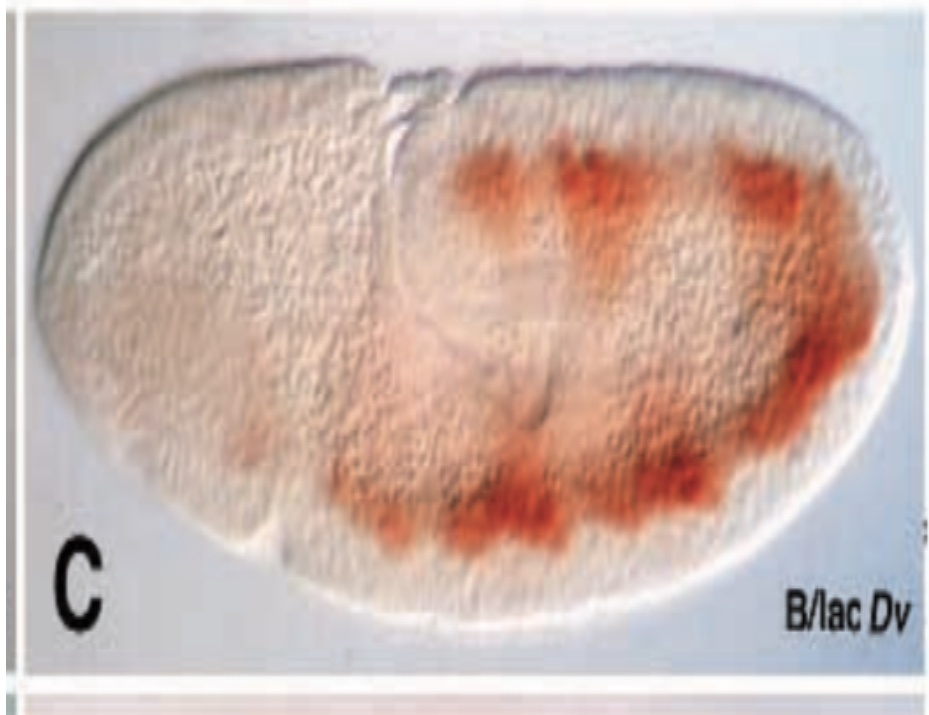

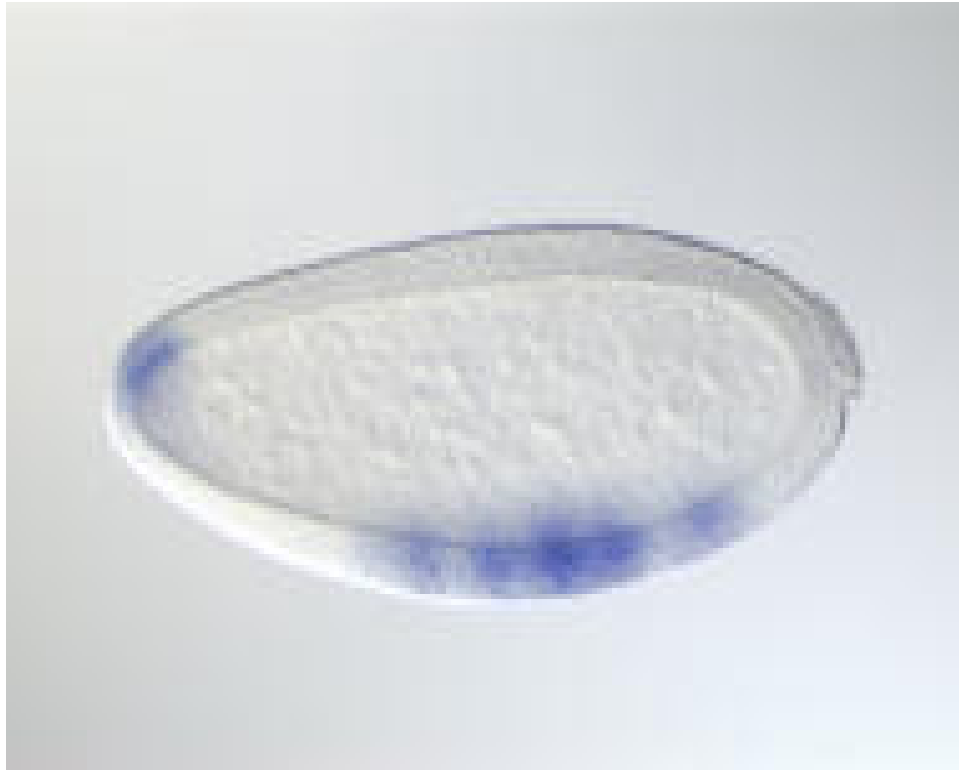

Location: Ventral Type: Enhancer ZScore: -0.13878009 PValue: 0.889623928

Supplement: S3 File — Reports consist of in situ hybridization images, ATAC-seq traces, and calculated p-value and Z Score for each region used in the final analysis. (ZIP) [file pgen.1007367.s015.zip › S3_File/tin_Yin_Report.pdf]

tld\_Kirov

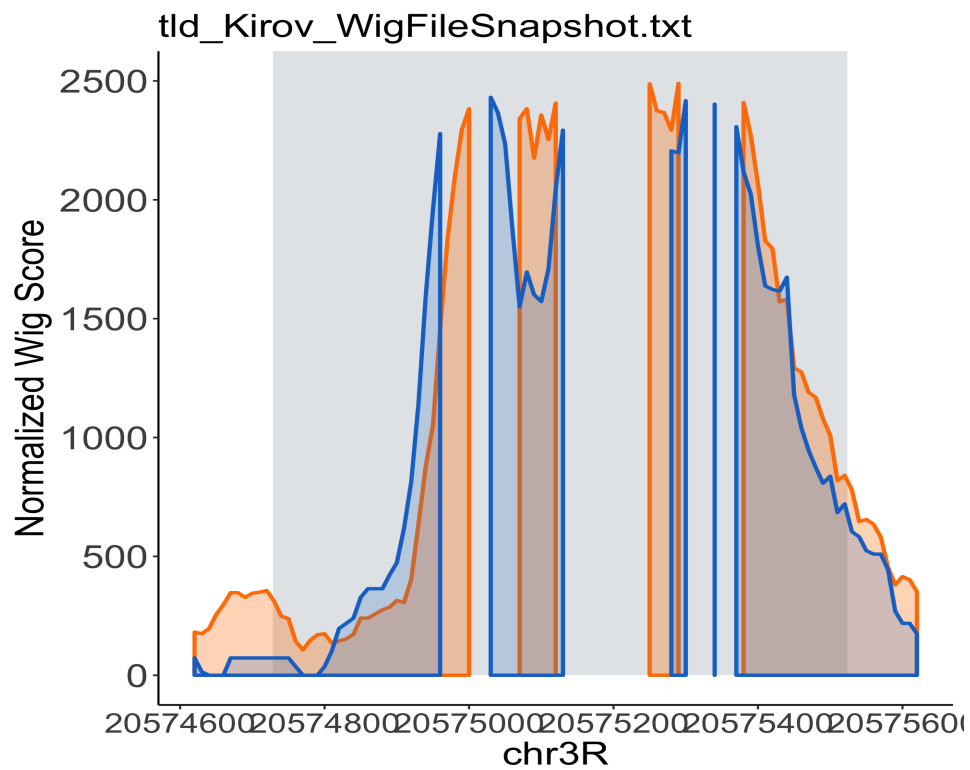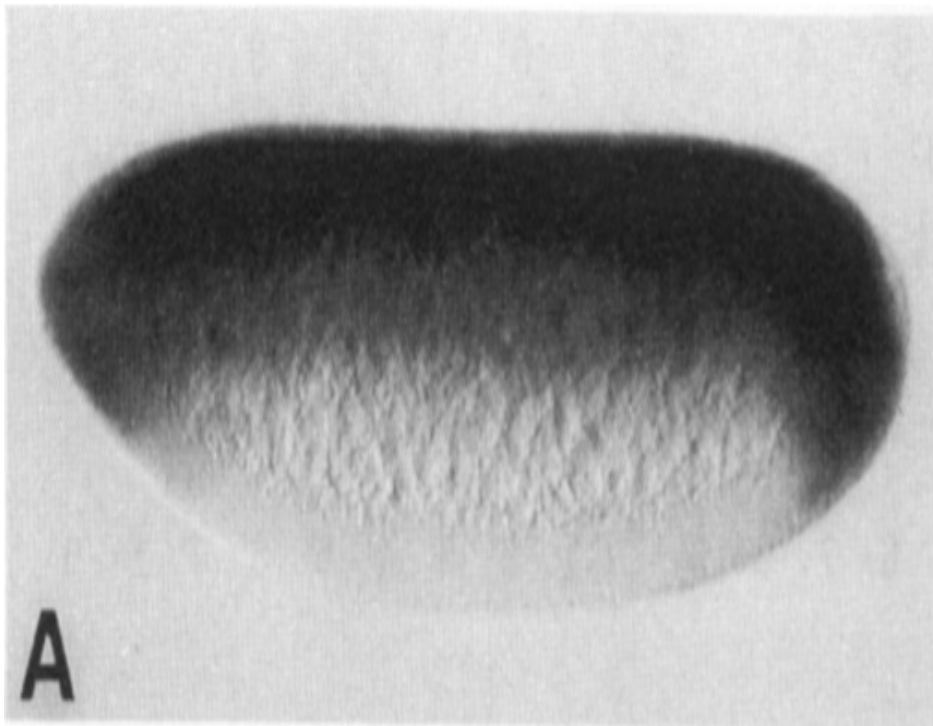

Location: Dorsal Type: Enhancer ZScore: -0.026214834 PValue: 0.979085984

Supplement: S3 File — Reports consist of in situ hybridization images, ATAC-seq traces, and calculated p-value and Z Score for each region used in the final analysis. (ZIP) [file pgen.1007367.s015.zip › S3_File/tld_Kirov_Report.pdf]

tll\_O-E(69)

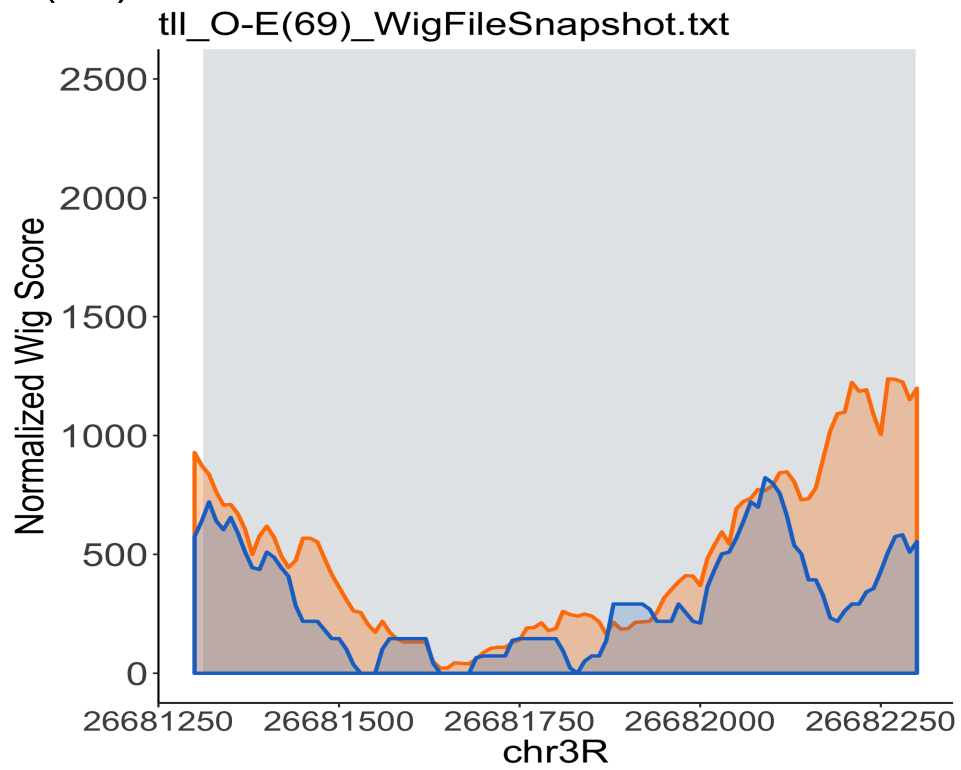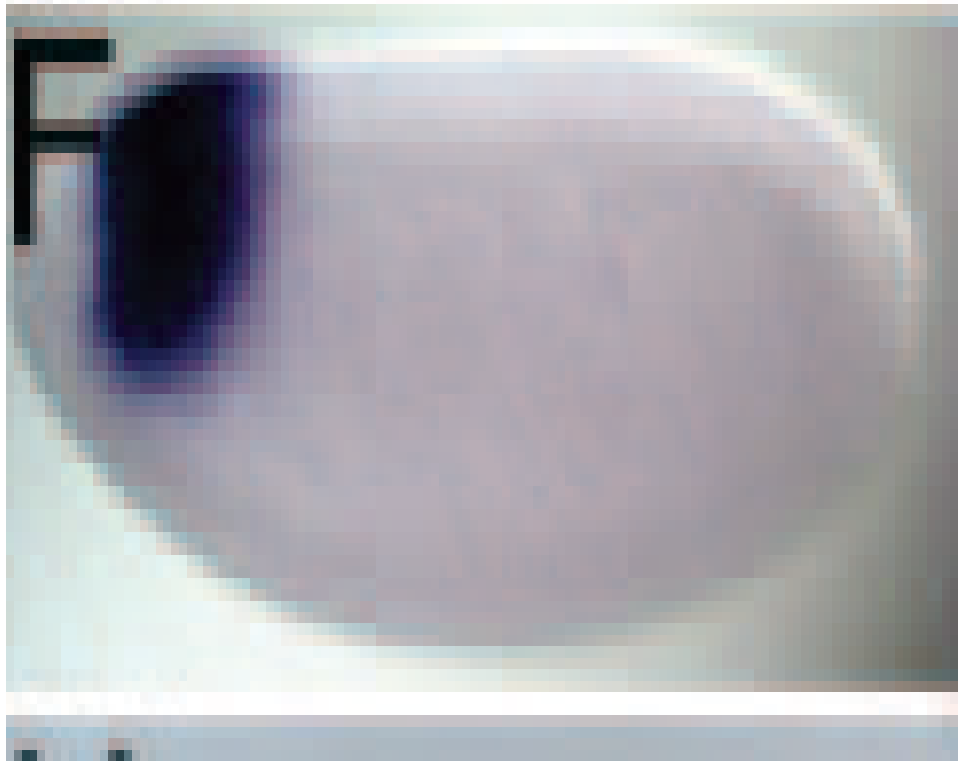

Location: Anterior Type: Enhancer ZScore: 1.054033428 PValue: 0.291867613

Supplement: S3 File — Reports consist of in situ hybridization images, ATAC-seq traces, and calculated p-value and Z Score for each region used in the final analysis. (ZIP) [file pgen.1007367.s015.zip › S3_File/tll_O-E(69)_Report.pdf]

toc

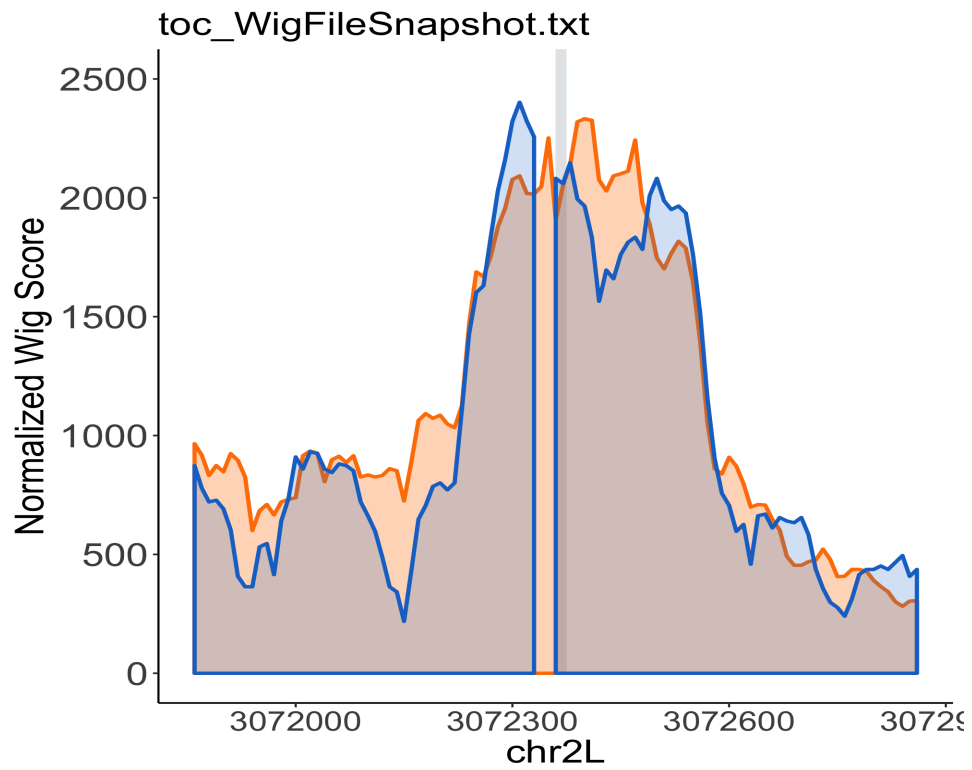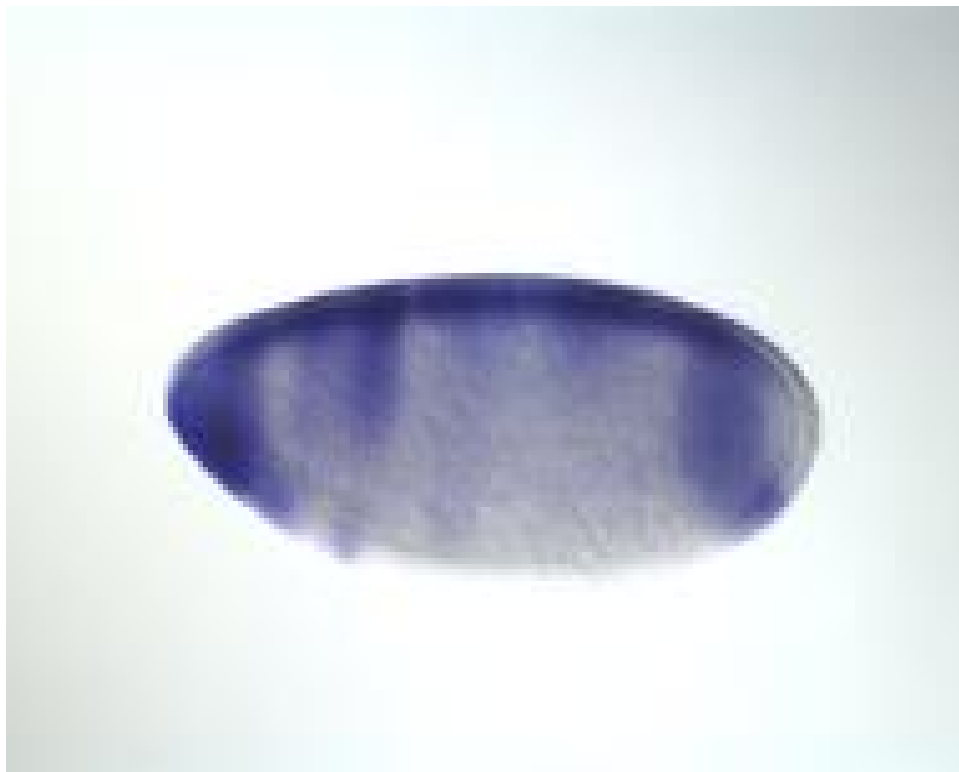

Location: Dorsal Type: Promoter ZScore: -0.149496738 PValue: 0.881161683

Supplement: S3 File — Reports consist of in situ hybridization images, ATAC-seq traces, and calculated p-value and Z Score for each region used in the final analysis. (ZIP) [file pgen.1007367.s015.zip › S3_File/toc_Report.pdf]

trbl\_Sandmann

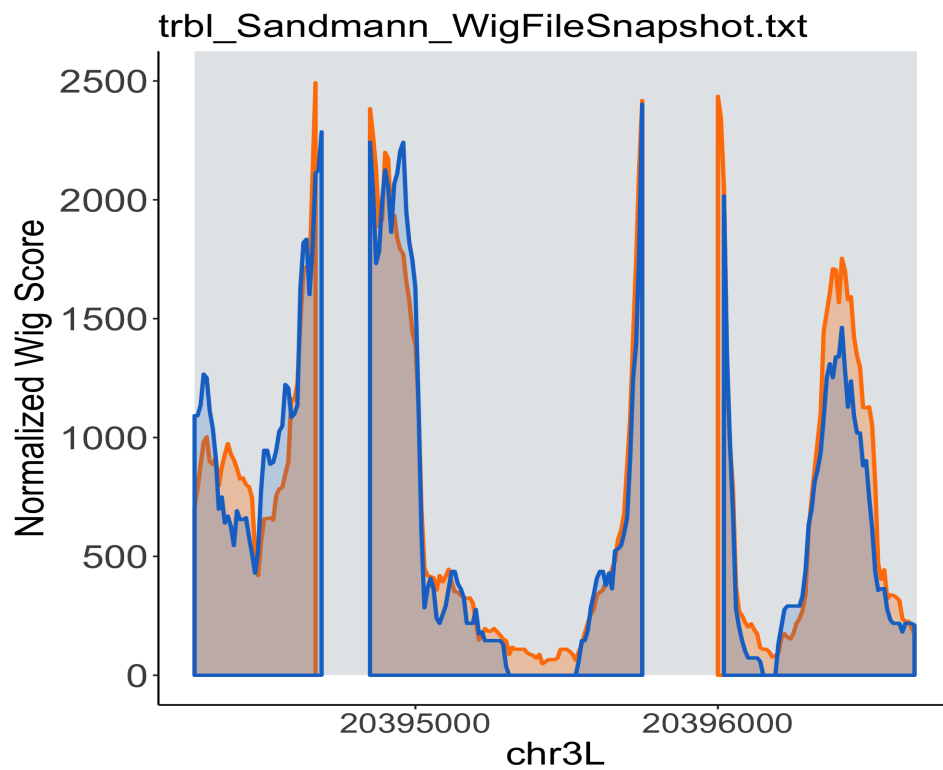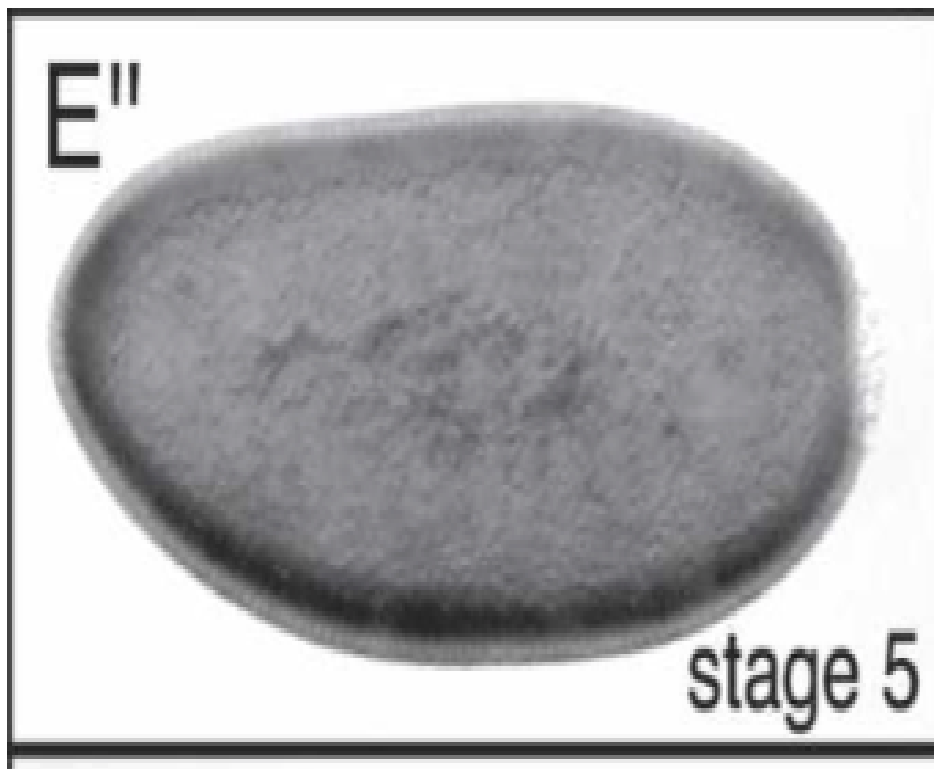

Location: Ventral Type: Enhancer ZScore: -0.083892416 PValue: 0.93314197

Supplement: S3 File — Reports consist of in situ hybridization images, ATAC-seq traces, and calculated p-value and Z Score for each region used in the final analysis. (ZIP) [file pgen.1007367.s015.zip › S3_File/trbl_Sandmann_Report.pdf]

tup

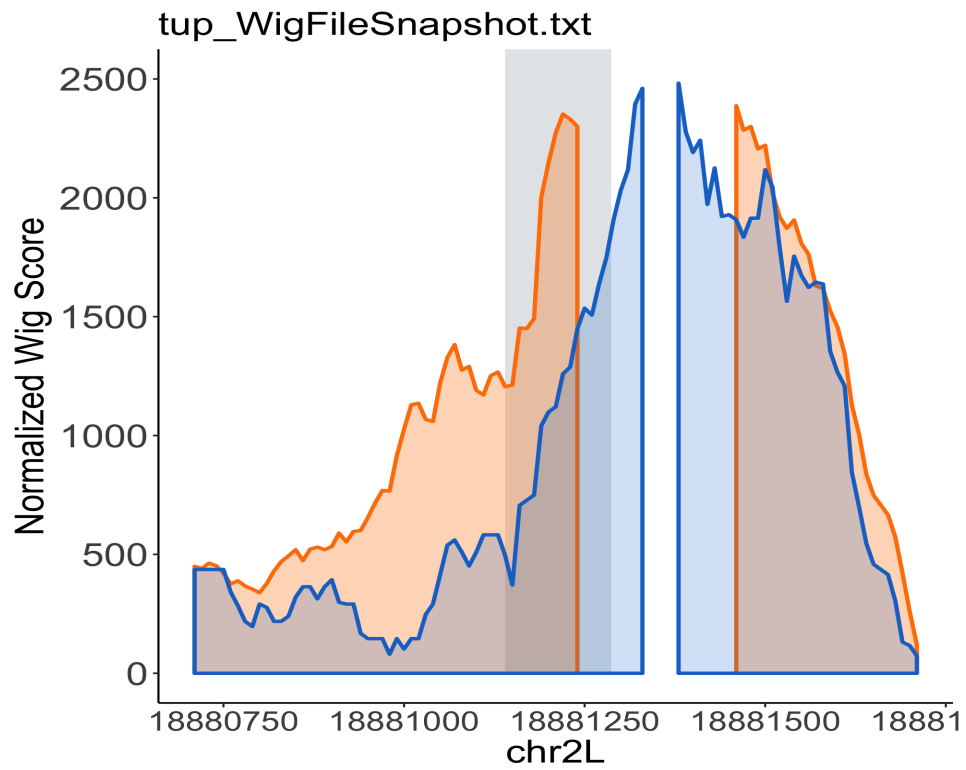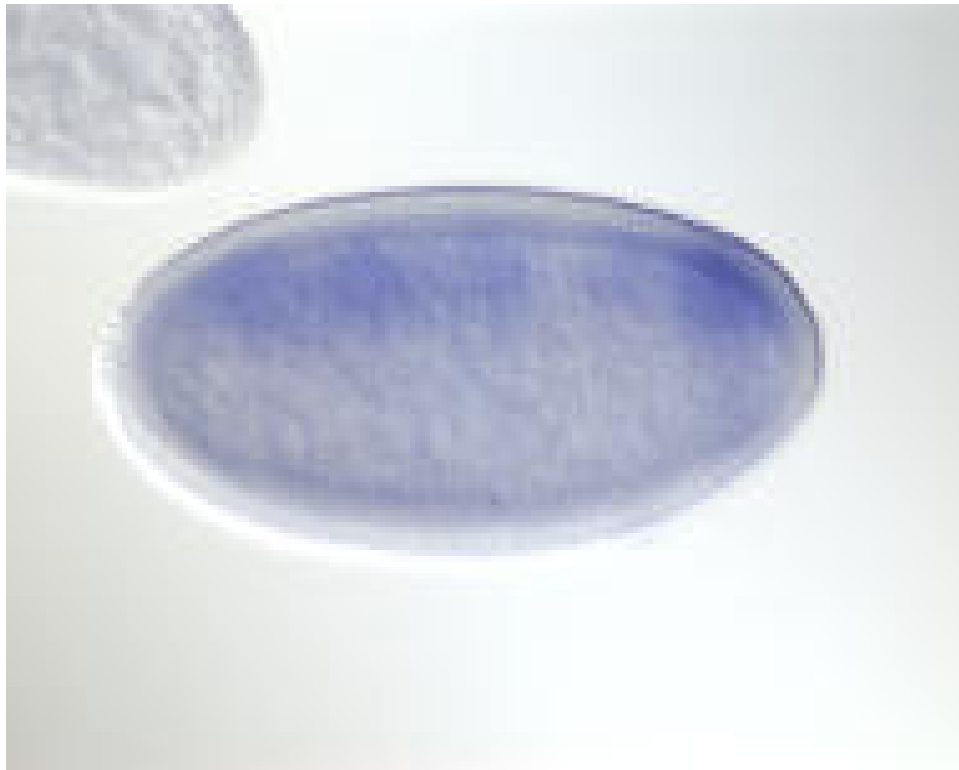

Location: Dorsal Type: Promoter ZScore: 1.360716613 PValue: 0.173603262

Supplement: S3 File — Reports consist of in situ hybridization images, ATAC-seq traces, and calculated p-value and Z Score for each region used in the final analysis. (ZIP) [file pgen.1007367.s015.zip › S3_File/tup_Report.pdf]

## tup\_Zeitlinger

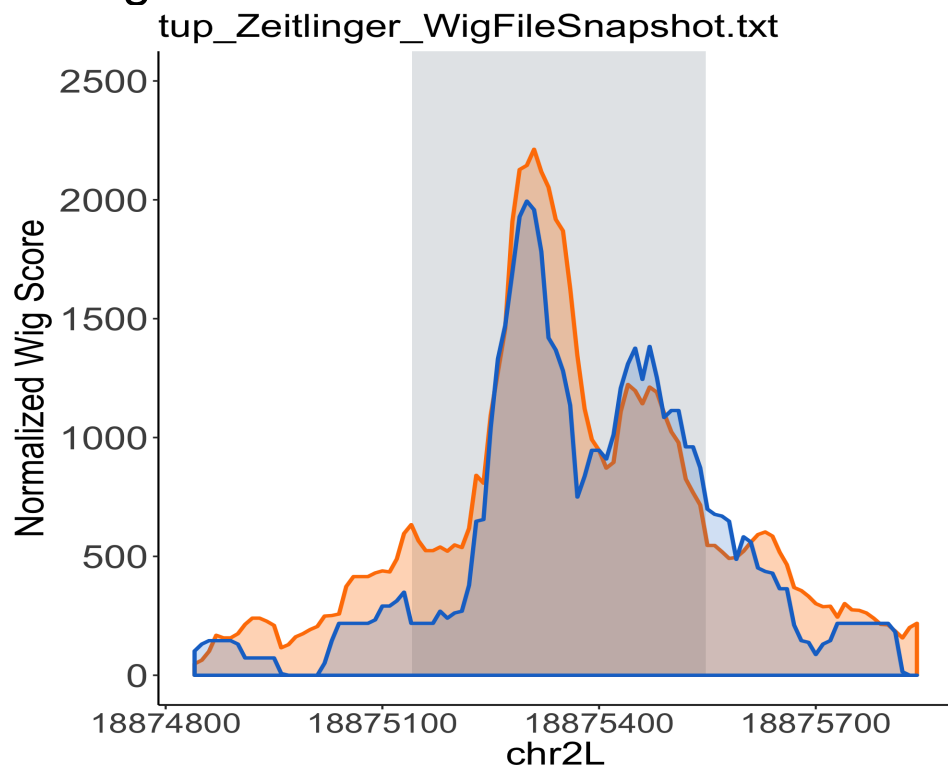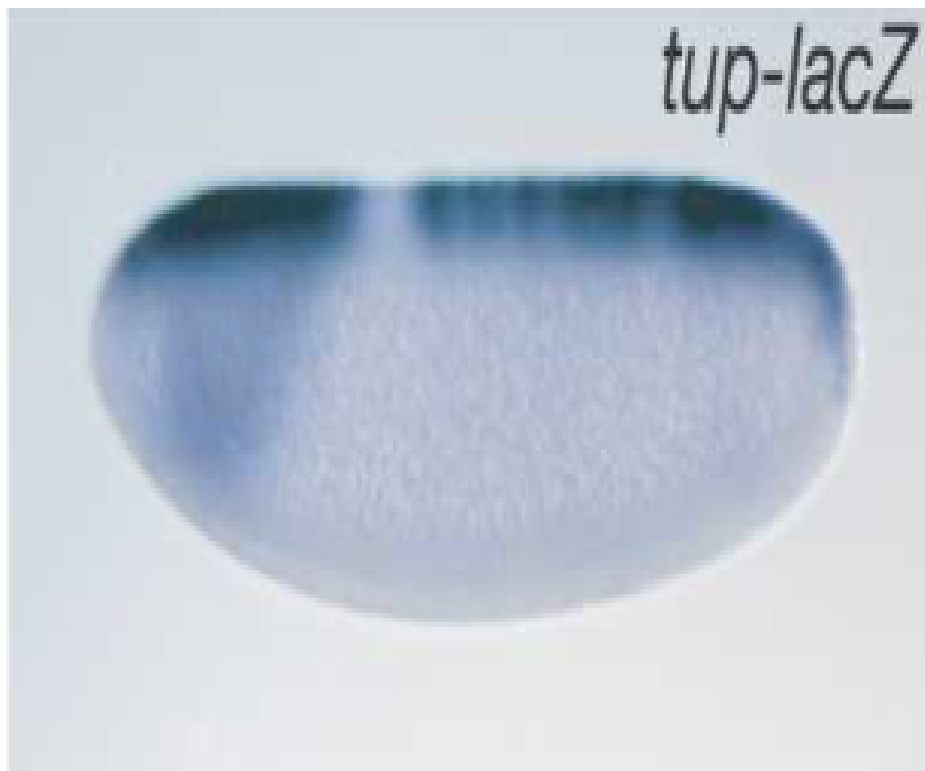

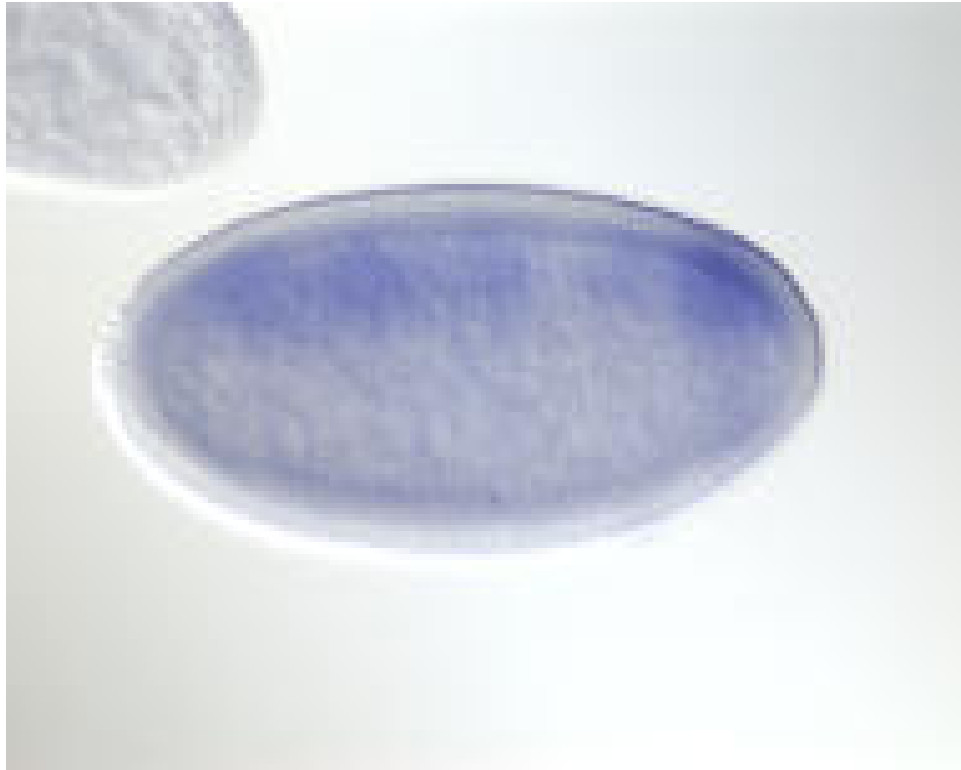

Location: Dorsal Type: Enhancer ZScore: 0.258334731 PValue: 0.796148584

Supplement: S3 File — Reports consist of in situ hybridization images, ATAC-seq traces, and calculated p-value and Z Score for each region used in the final analysis. (ZIP) [file pgen.1007367.s015.zip › S3_File/tup_Zeitlinger_Report.pdf]

twi\_Jiang

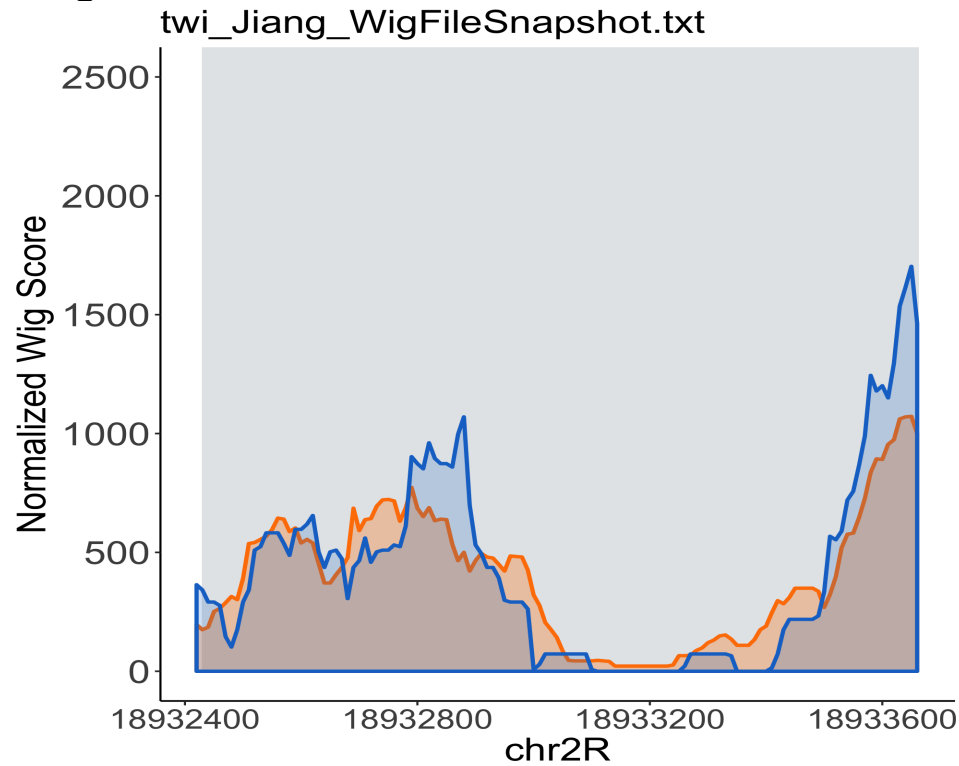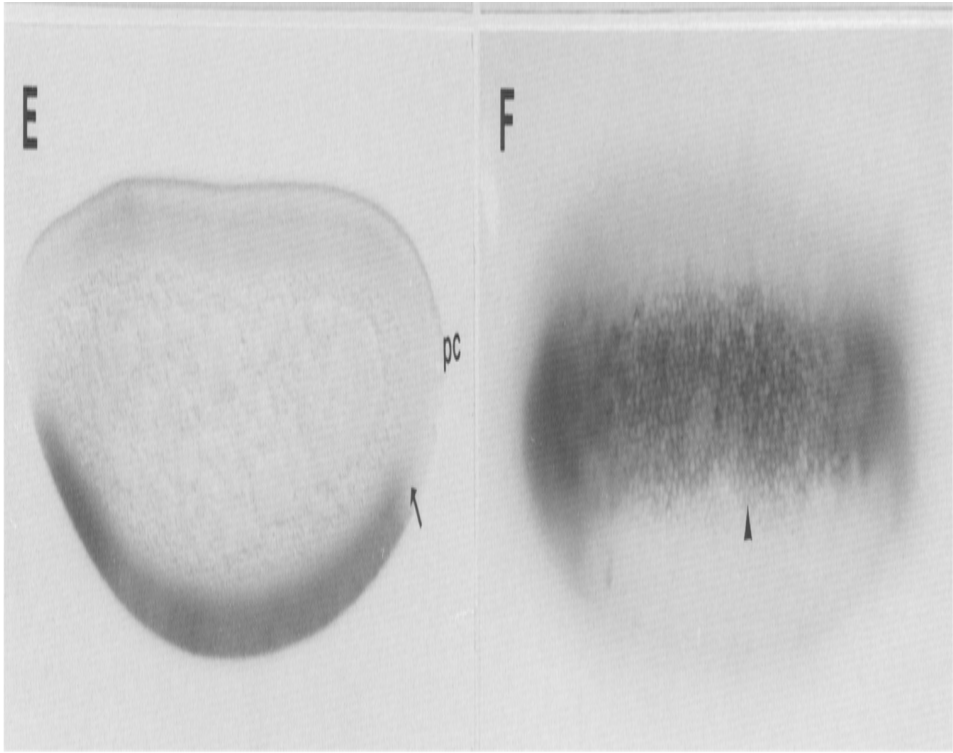

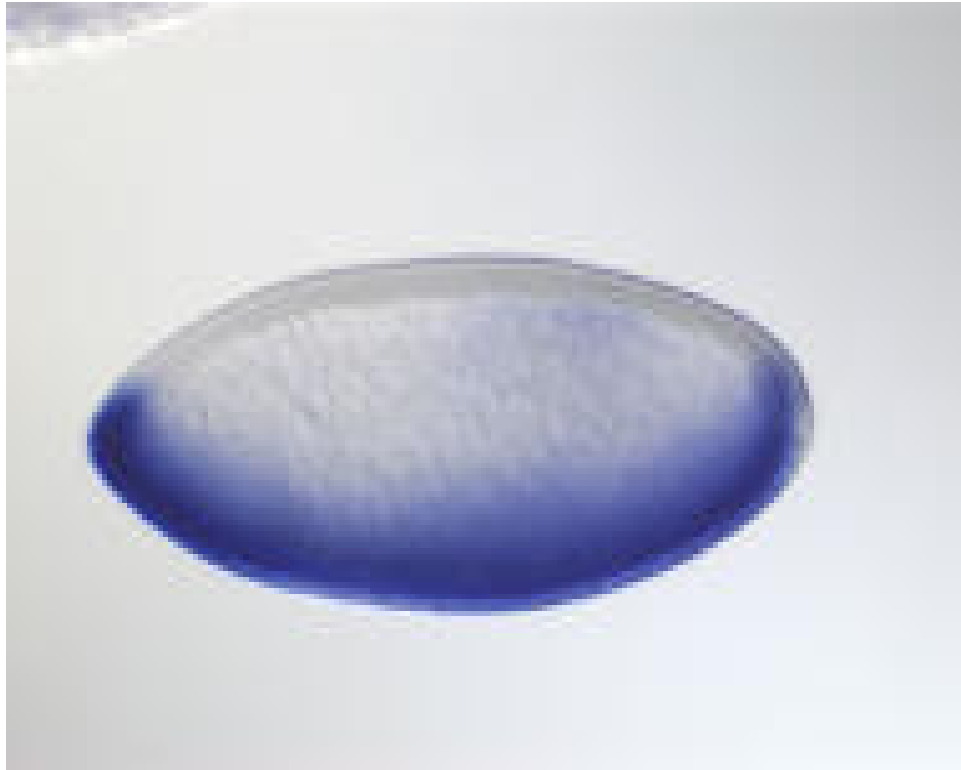

Location: Ventral Type: Enhancer ZScore: -0.123592628 PValue: 0.90163783

Supplement: S3 File — Reports consist of in situ hybridization images, ATAC-seq traces, and calculated p-value and Z Score for each region used in the final analysis. (ZIP) [file pgen.1007367.s015.zip › S3_File/twi_Jiang_Report.pdf]

twi\_Ozdemir

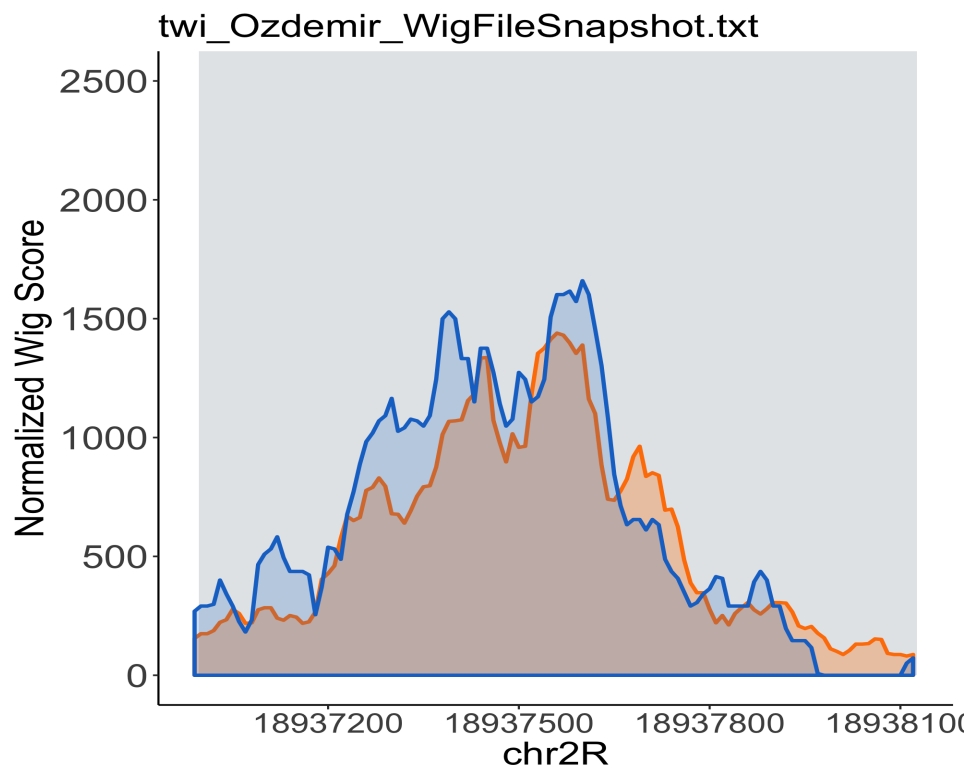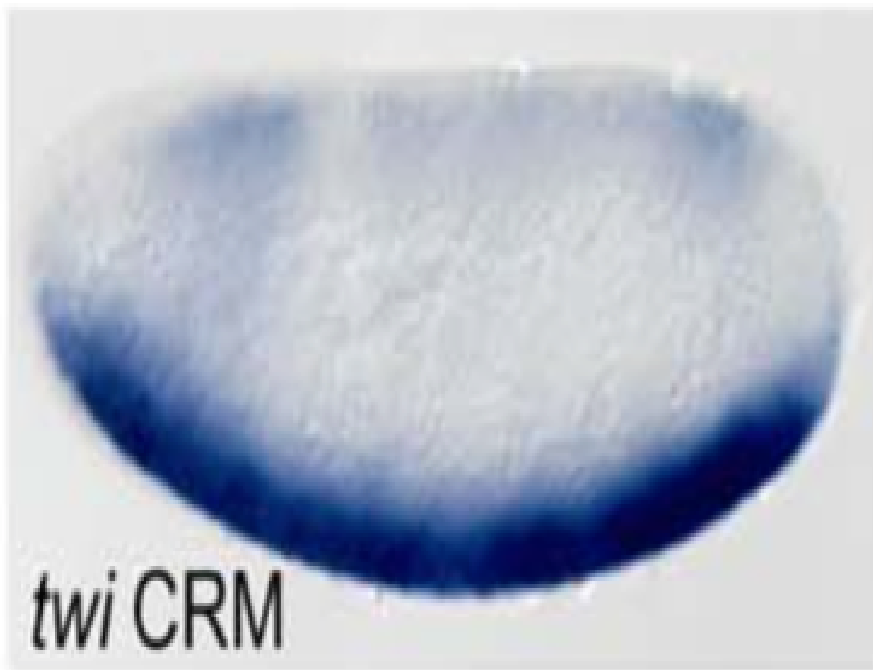

Location: Ventral Type: Enhancer ZScore: -0.334098814 PValue: 0.738304995

Supplement: S3 File — Reports consist of in situ hybridization images, ATAC-seq traces, and calculated p-value and Z Score for each region used in the final analysis. (ZIP) [file pgen.1007367.s015.zip › S3_File/twi_Ozdemir_Report.pdf]

vn

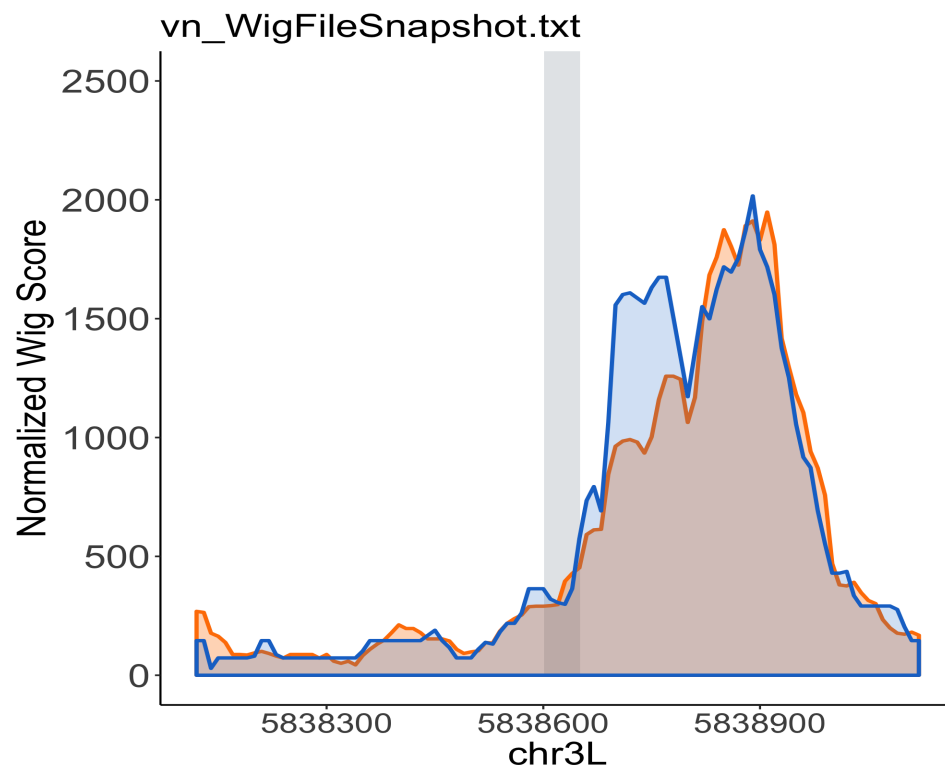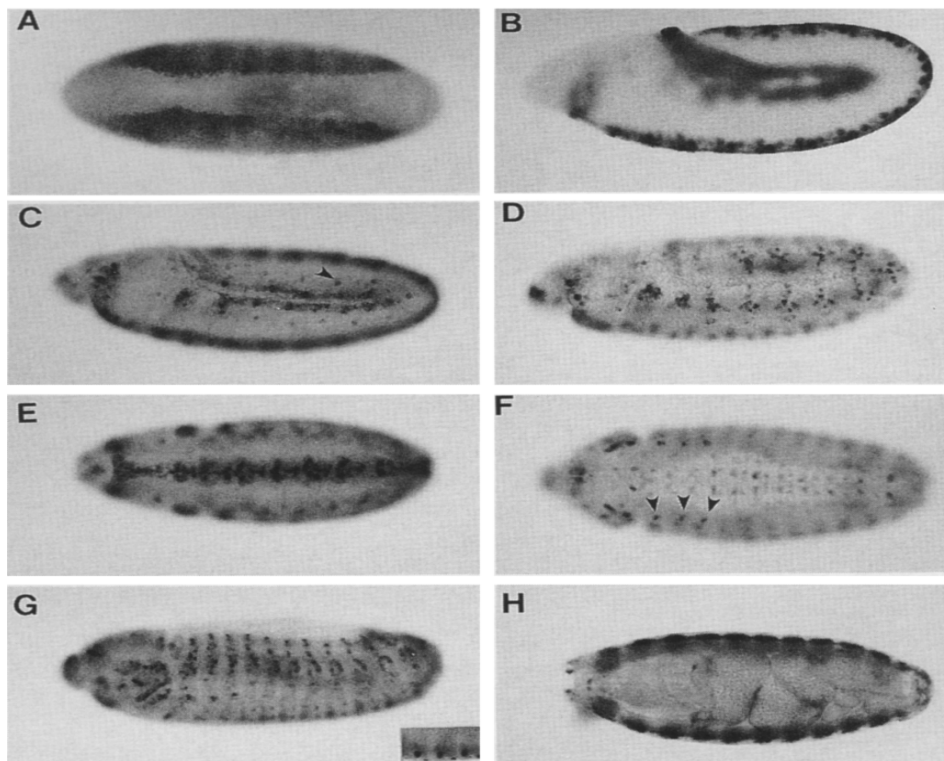

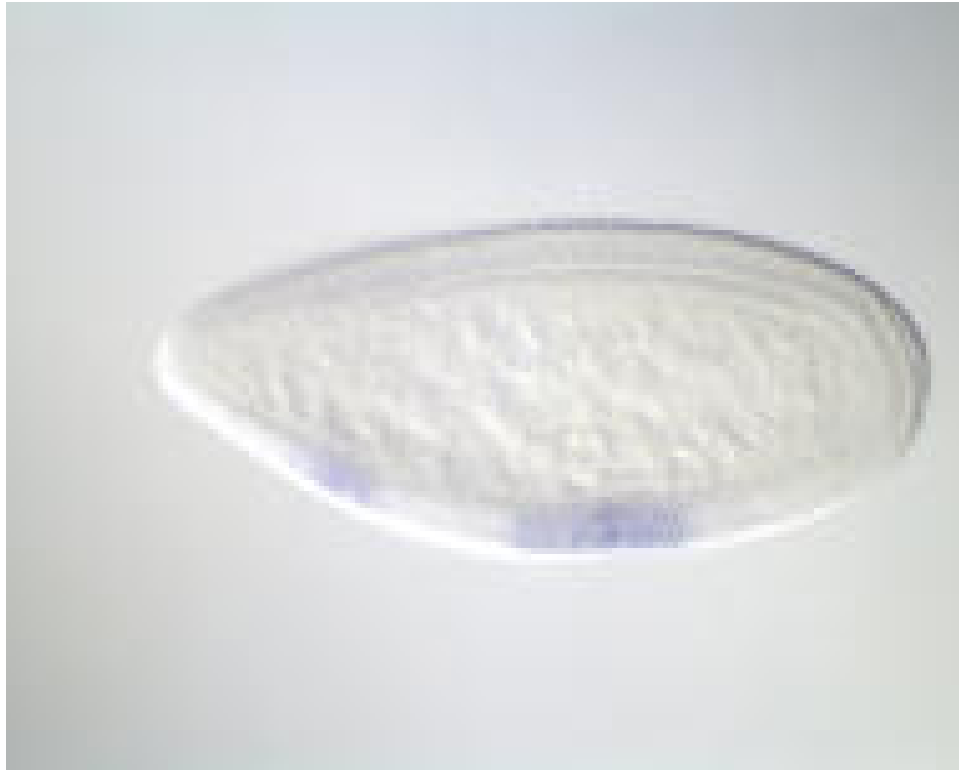

Location: Ventral Type: Promoter ZScore: -0.12016752 PValue: 0.904350446

Supplement: S3 File — Reports consist of in situ hybridization images, ATAC-seq traces, and calculated p-value and Z Score for each region used in the final analysis. (ZIP) [file pgen.1007367.s015.zip › S3_File/vn_Report.pdf]

# VT0984\_Kvon

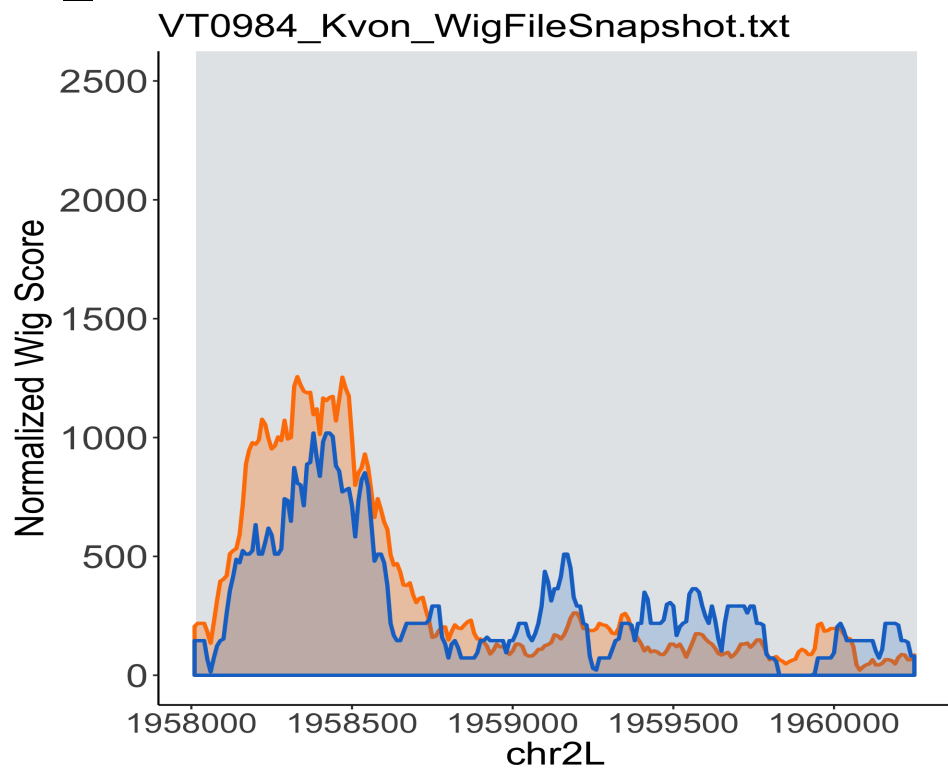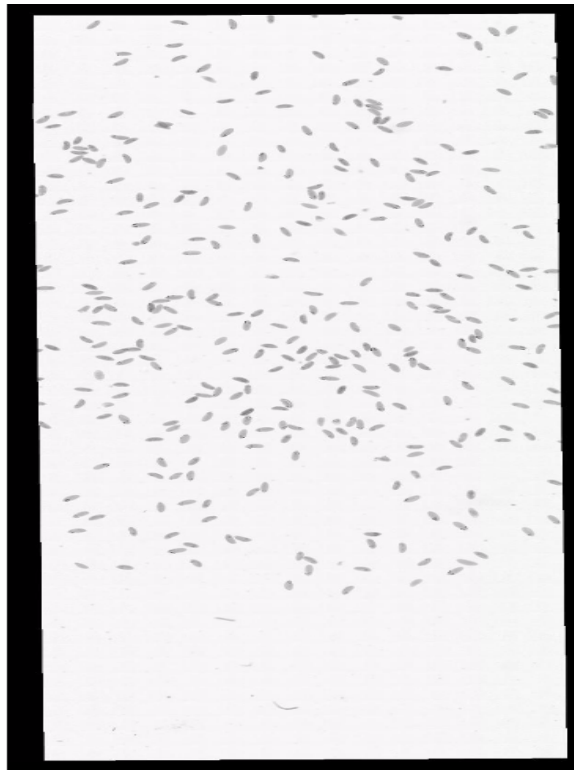

Location: Anterior Type: Enhancer ZScore: 0.293712513 PValue: 0.768977592

Supplement: S3 File — Reports consist of in situ hybridization images, ATAC-seq traces, and calculated p-value and Z Score for each region used in the final analysis. (ZIP) [file pgen.1007367.s015.zip › S3_File/VT0984_Kvon_Report.pdf]

VT13665

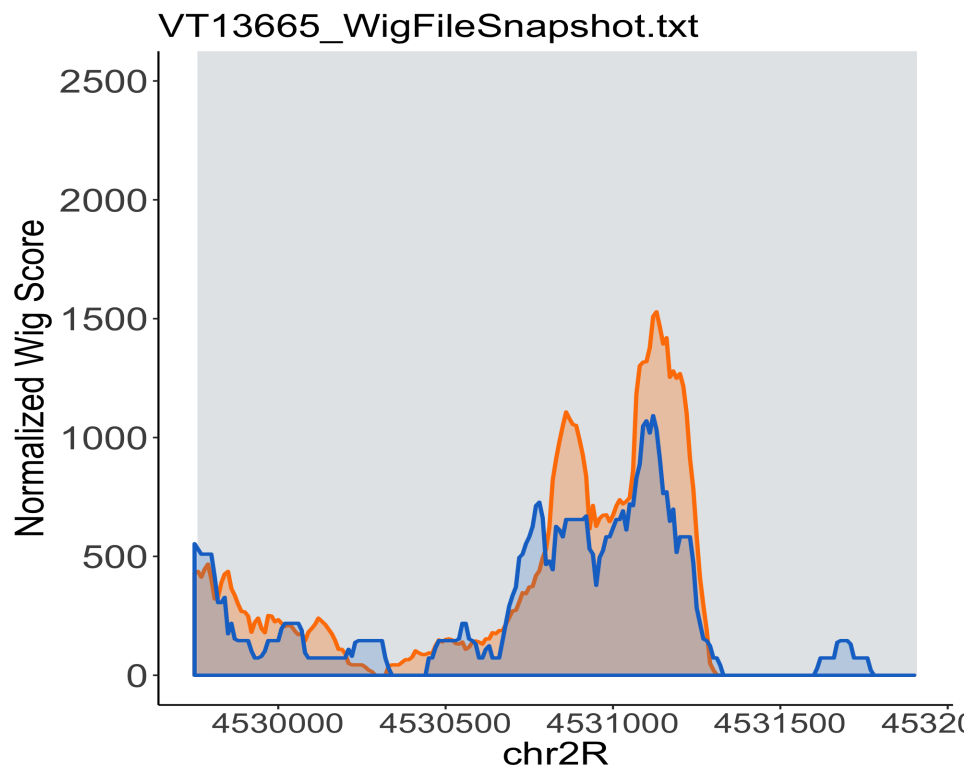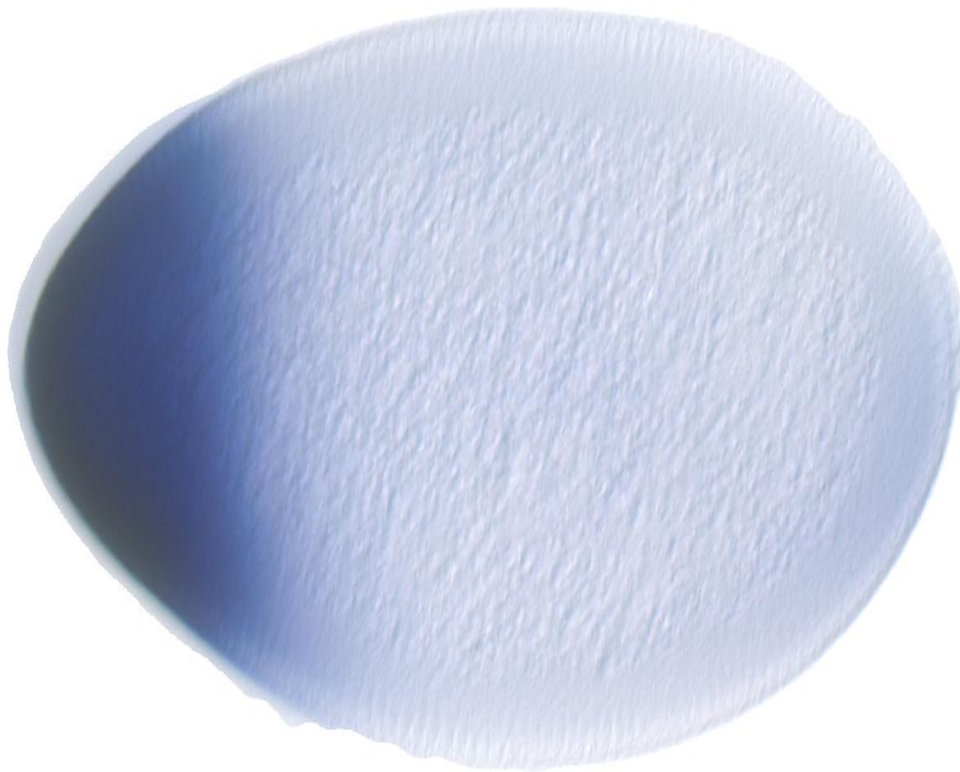

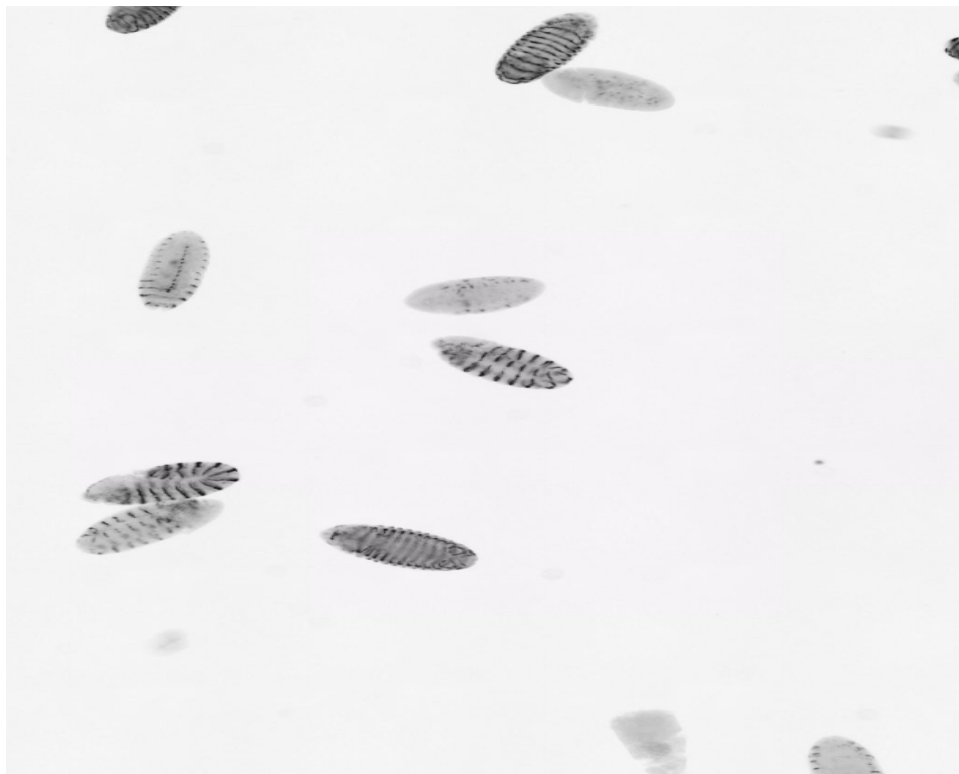

Location: Anterior Type: Enhancer ZScore: 0.50040511 PValue: 0.616789856

Supplement: S3 File — Reports consist of in situ hybridization images, ATAC-seq traces, and calculated p-value and Z Score for each region used in the final analysis. (ZIP) [file pgen.1007367.s015.zip › S3_File/VT13665_Report.pdf]

## VT1404\_Kvon

VT1404\_Kvon\_WigFileSnapshot.txt

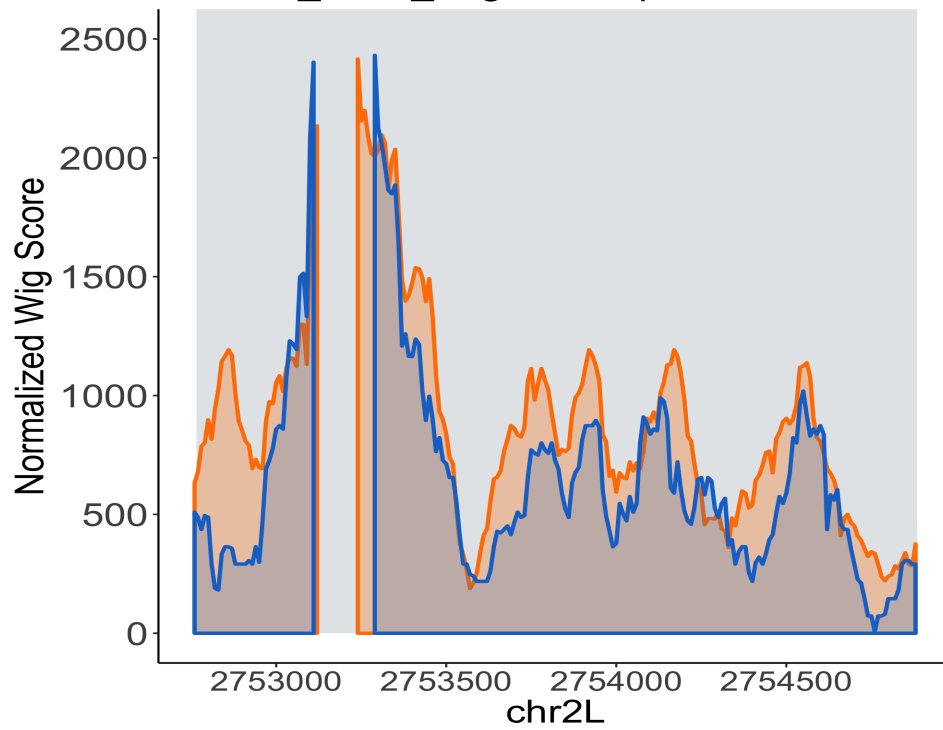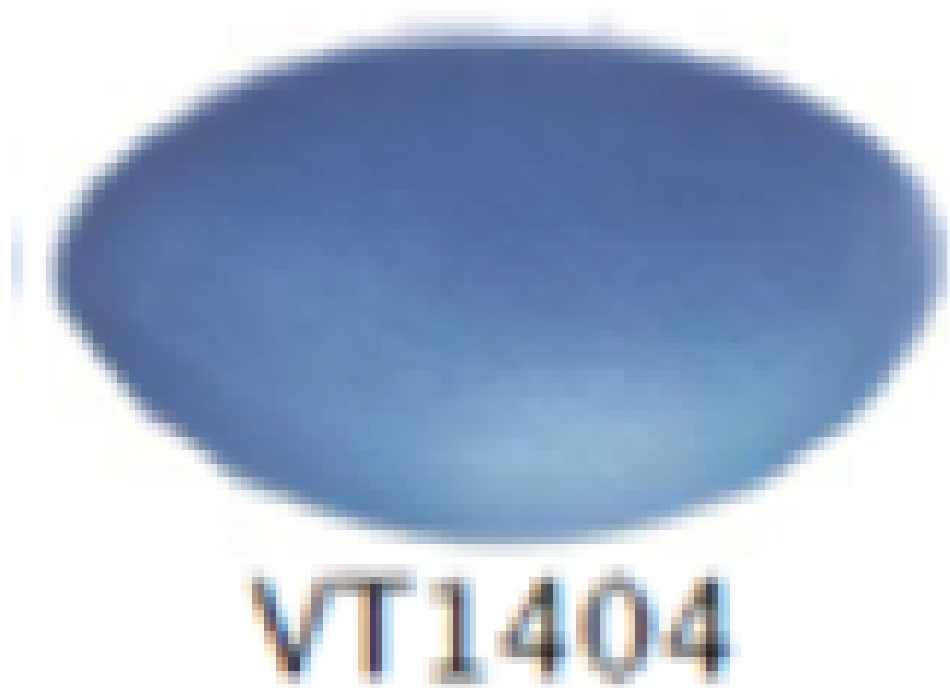

Location: Dorsal Type: Enhancer ZScore: 0.316905731 PValue: 0.751315133

Supplement: S3 File — Reports consist of in situ hybridization images, ATAC-seq traces, and calculated p-value and Z Score for each region used in the final analysis. (ZIP) [file pgen.1007367.s015.zip › S3_File/VT1404_Kvon_Report.pdf]

VT14979

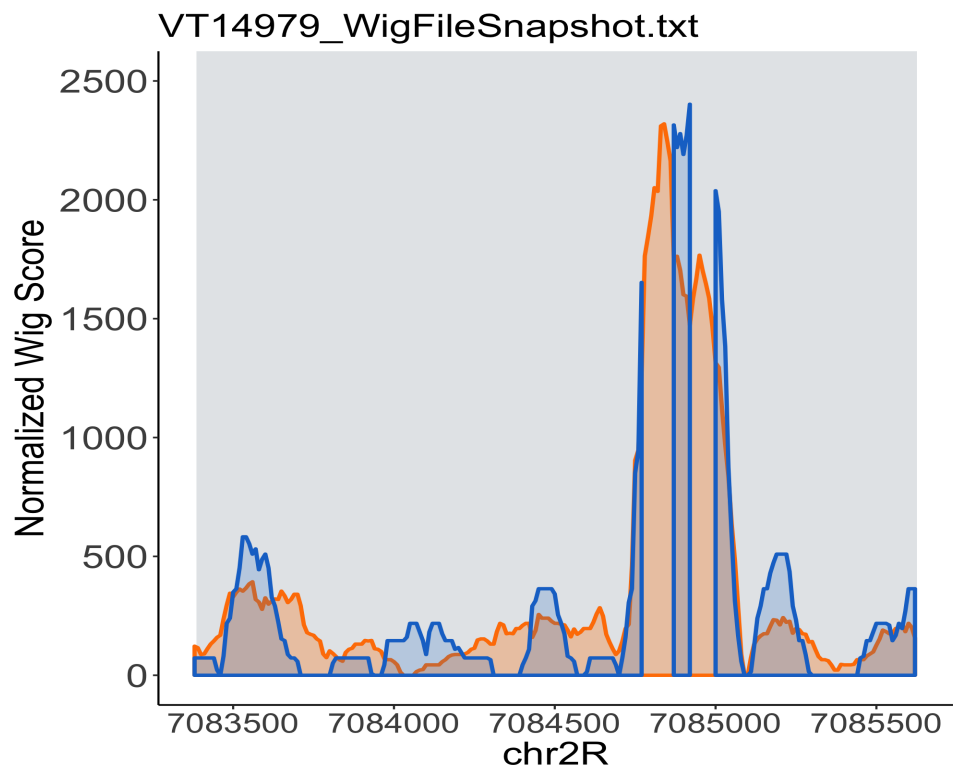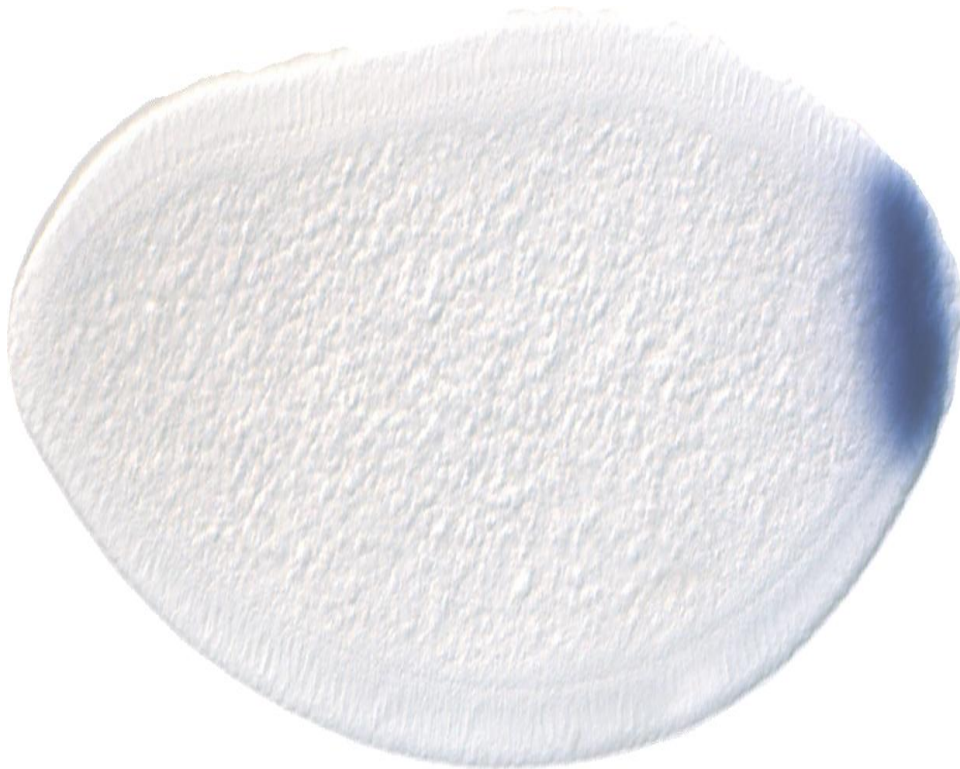

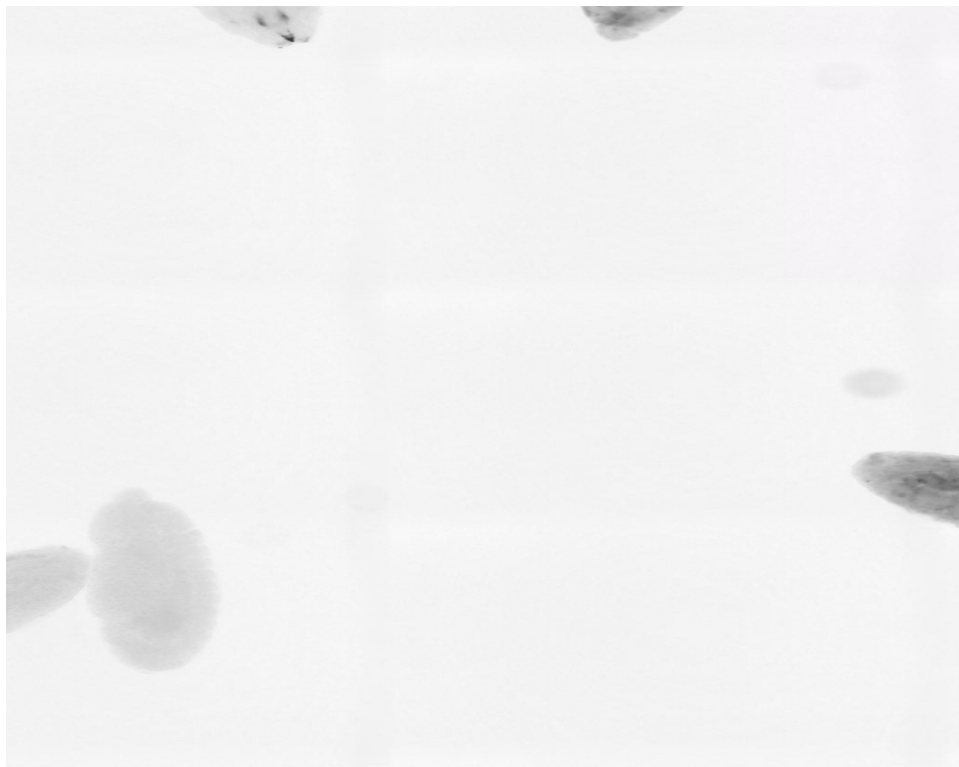

Location: Posterior Type: Enhancer ZScore: 0.462230654 PValue: 0.643915923

Supplement: S3 File — Reports consist of in situ hybridization images, ATAC-seq traces, and calculated p-value and Z Score for each region used in the final analysis. (ZIP) [file pgen.1007367.s015.zip › S3_File/VT14979_Report.pdf]

# VT15341\_Kvon

VT15341\_Kvon\_WigFileSnapshot.txt

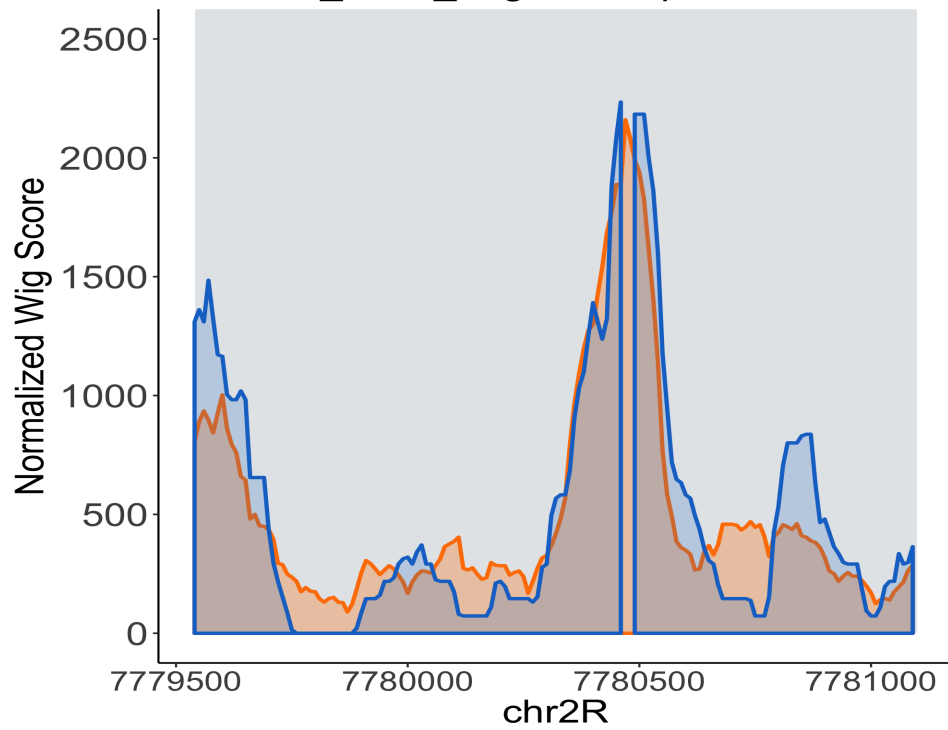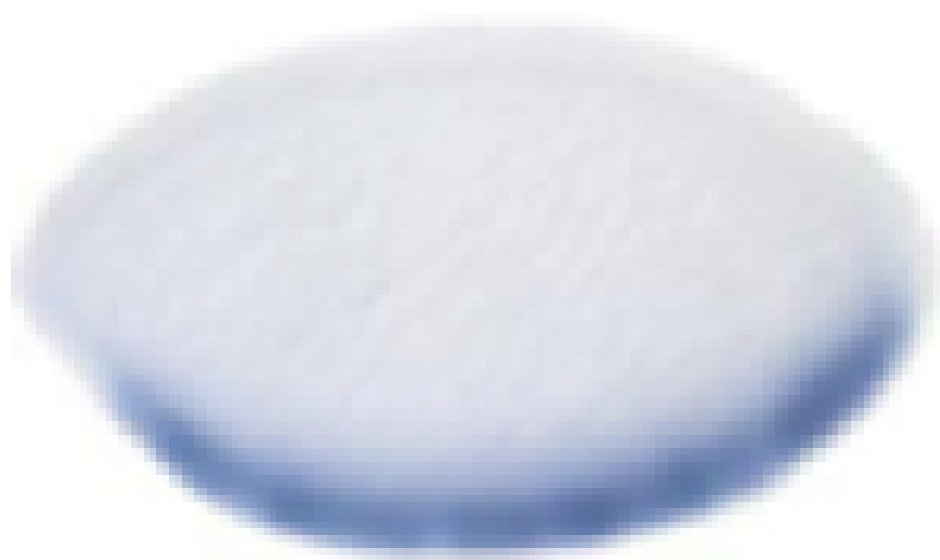

VT15341

Location: Ventral Type: Enhancer ZScore: -0.165570986 PValue: 0.868494582

Supplement: S3 File — Reports consist of in situ hybridization images, ATAC-seq traces, and calculated p-value and Z Score for each region used in the final analysis. (ZIP) [file pgen.1007367.s015.zip › S3_File/VT15341_Kvon_Report.pdf]

# VT20119\_Kvon

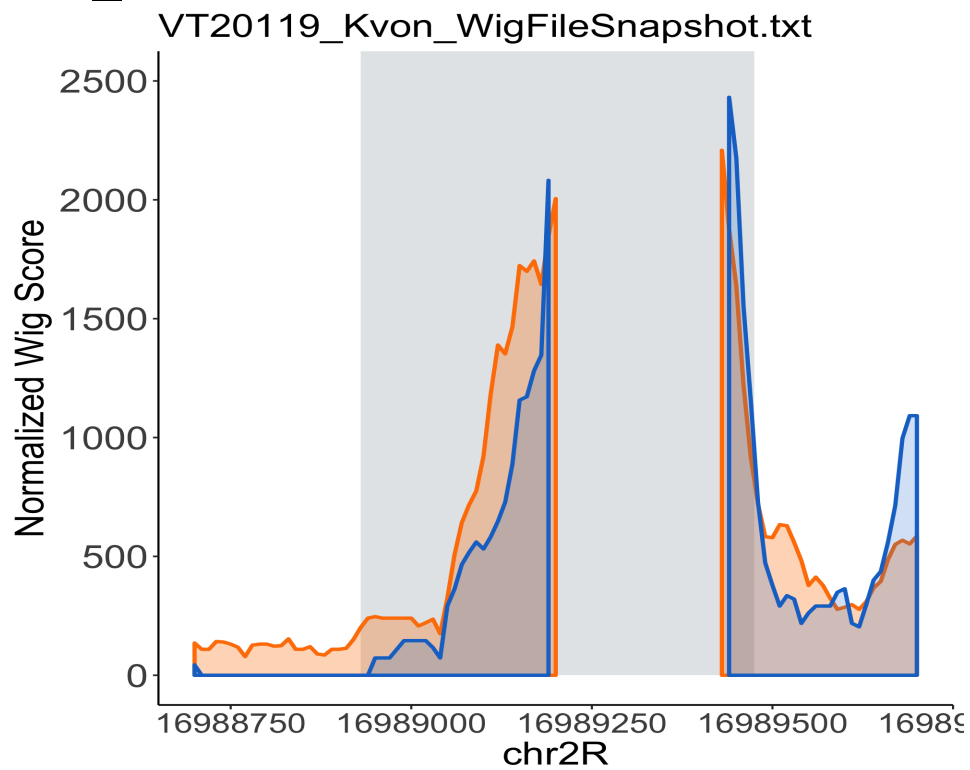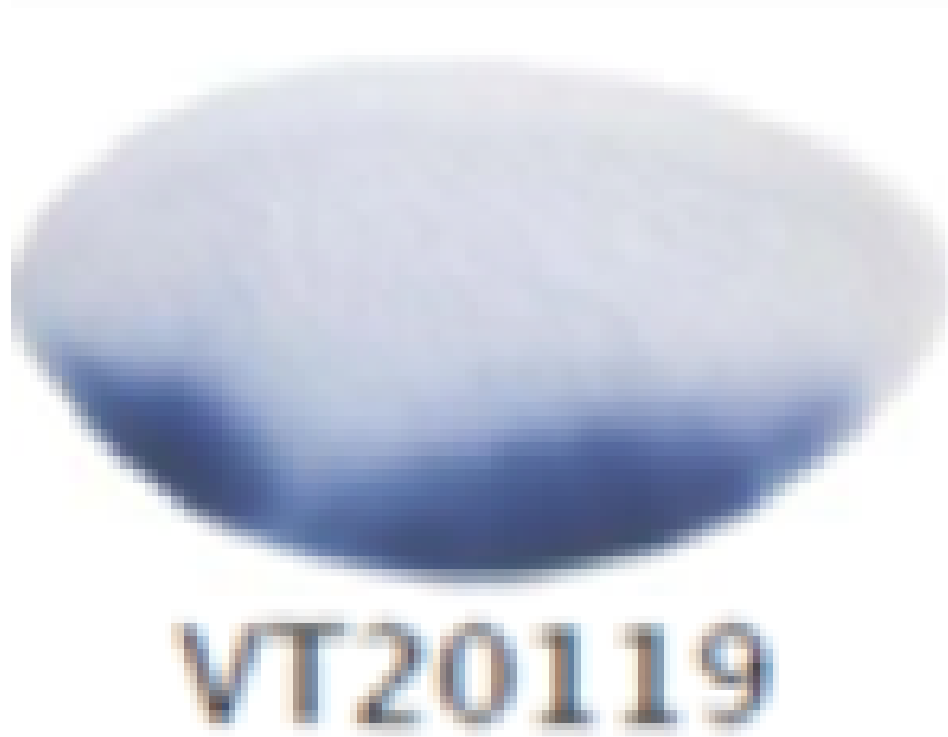

Location: Ventral Type: Enhancer ZScore: -0.450824085 PValue: 0.652116341

Supplement: S3 File — Reports consist of in situ hybridization images, ATAC-seq traces, and calculated p-value and Z Score for each region used in the final analysis. (ZIP) [file pgen.1007367.s015.zip › S3_File/VT20119_Kvon_Report.pdf]

# VT21015\_Kvon

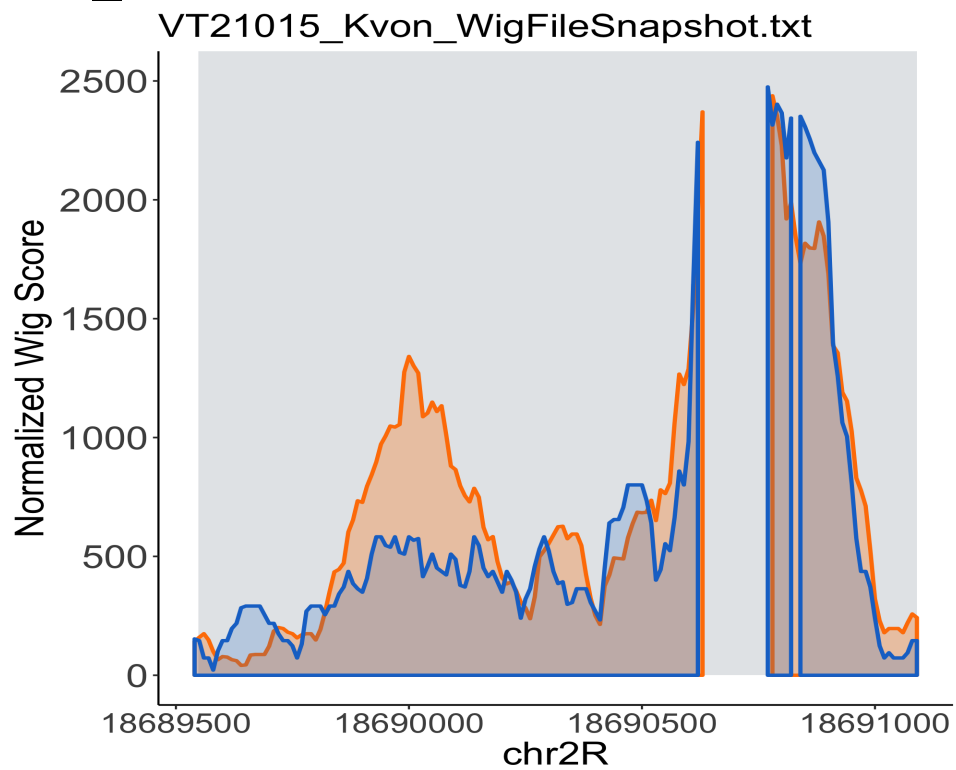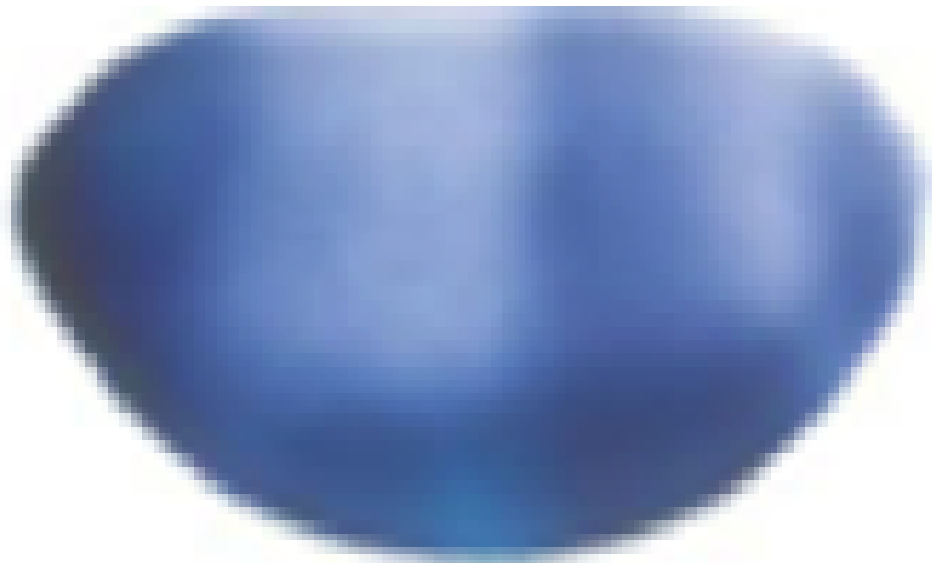

VT21015

Location: Ventral Type: Enhancer ZScore: 0.077375548 PValue: 0.938324792

Supplement: S3 File — Reports consist of in situ hybridization images, ATAC-seq traces, and calculated p-value and Z Score for each region used in the final analysis. (ZIP) [file pgen.1007367.s015.zip › S3_File/VT21015_Kvon_Report.pdf]

# VT26325\_Kvon

VT26325\_Kvon\_WigFileSnapshot.txt

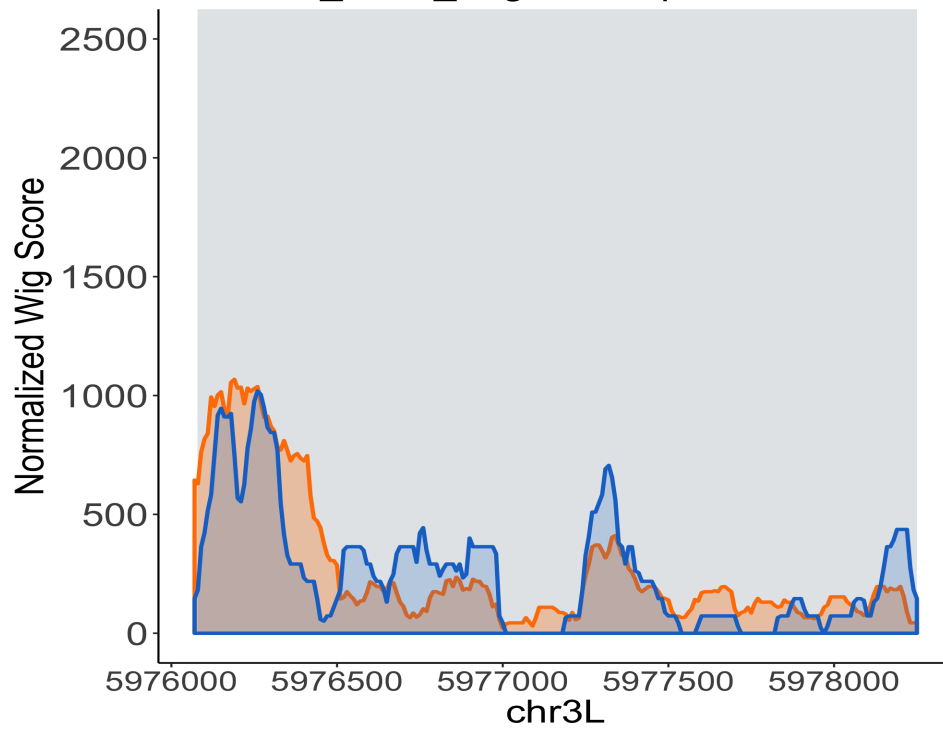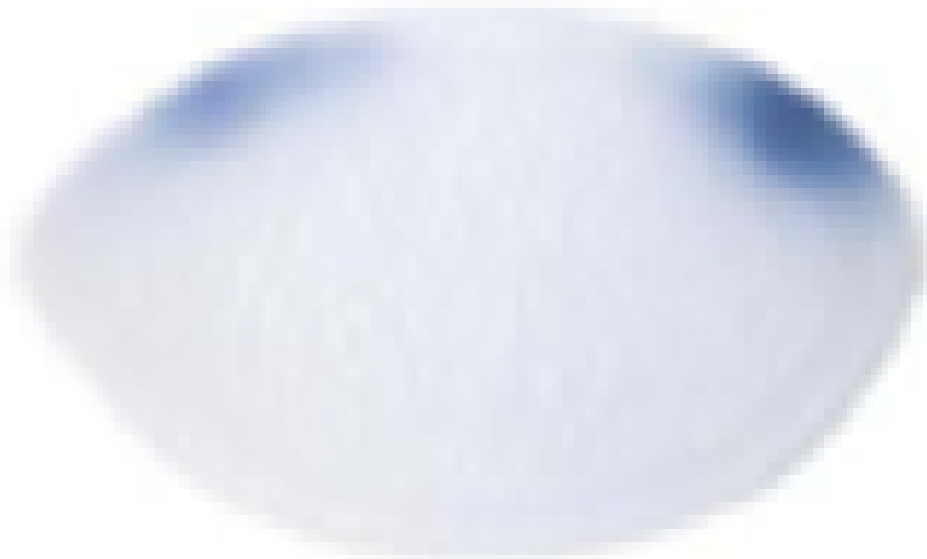

VT26325

Location: Dorsal Type: Enhancer ZScore: 0.144373013 PValue: 0.885205926

Supplement: S3 File — Reports consist of in situ hybridization images, ATAC-seq traces, and calculated p-value and Z Score for each region used in the final analysis. (ZIP) [file pgen.1007367.s015.zip › S3_File/VT26325_Kvon_Report.pdf]

VT33934

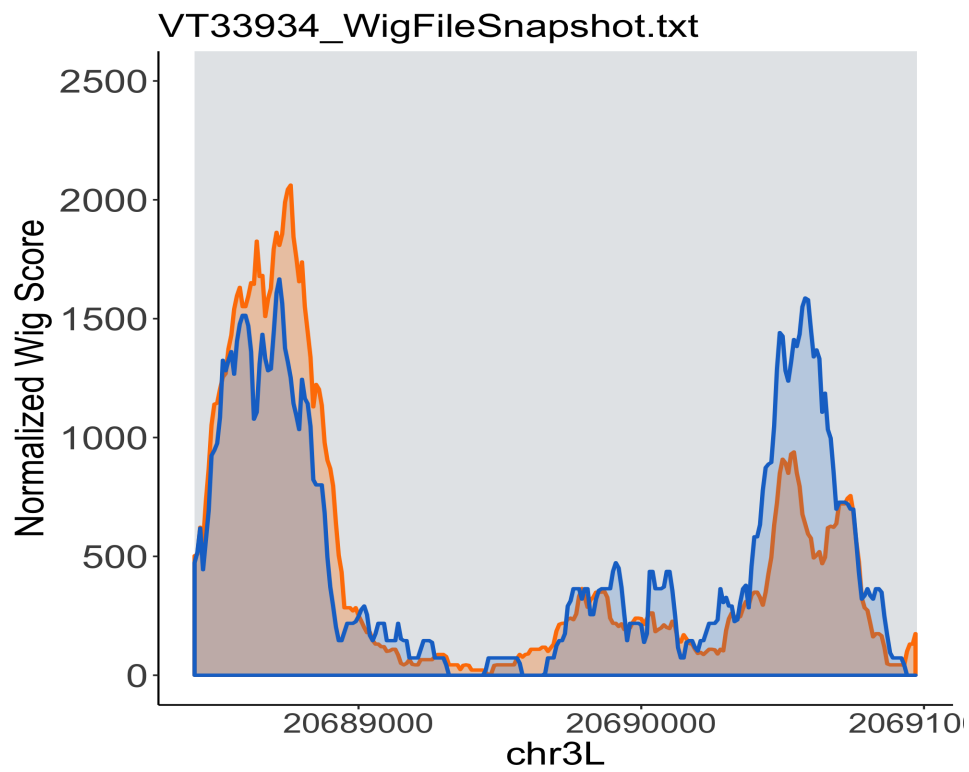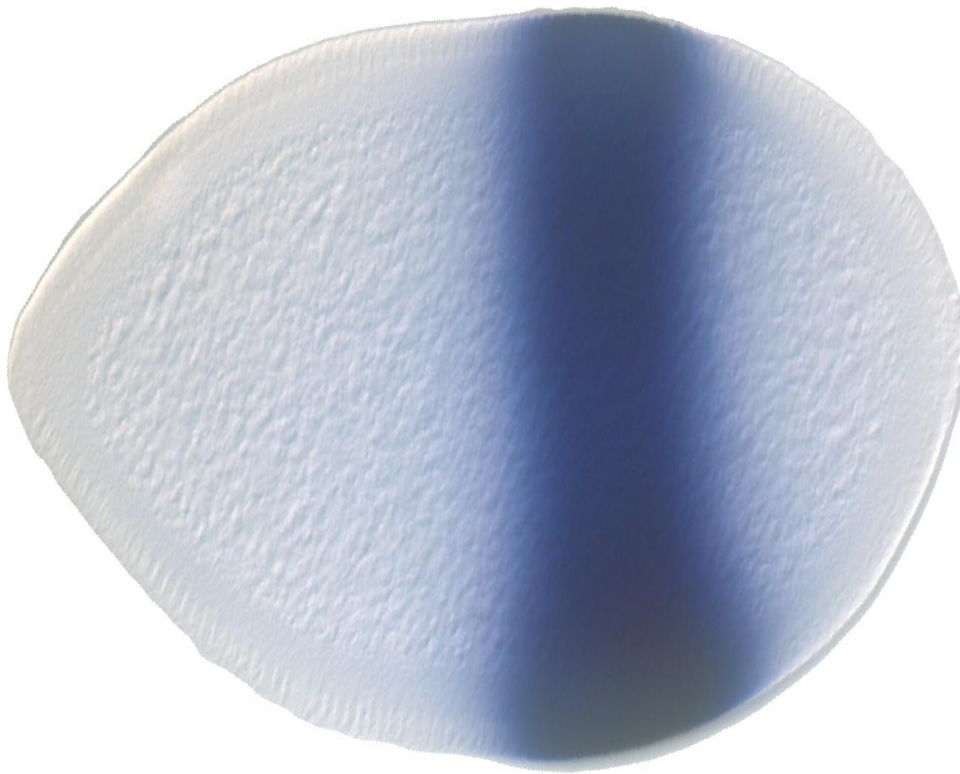

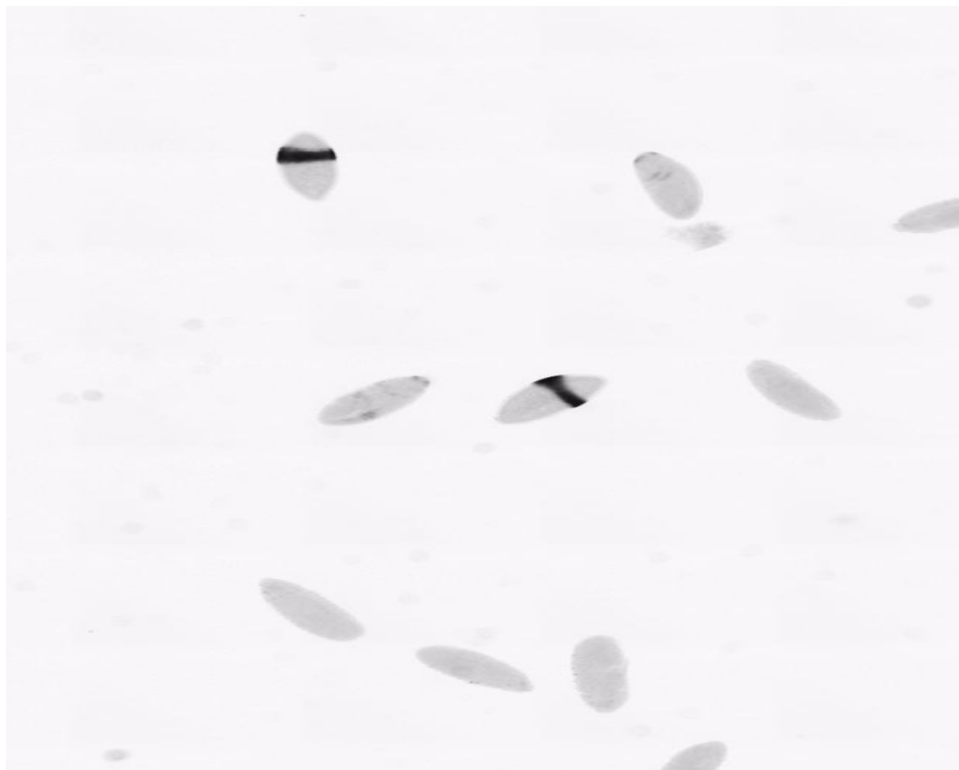

Location: Posterior Type: Enhancer ZScore: 0.051514663 PValue: 0.958915418

Supplement: S3 File — Reports consist of in situ hybridization images, ATAC-seq traces, and calculated p-value and Z Score for each region used in the final analysis. (ZIP) [file pgen.1007367.s015.zip › S3_File/VT33934_Report.pdf]

## VT34695\_Kvon

VT34695\_Kvon\_WigFileSnapshot.txt

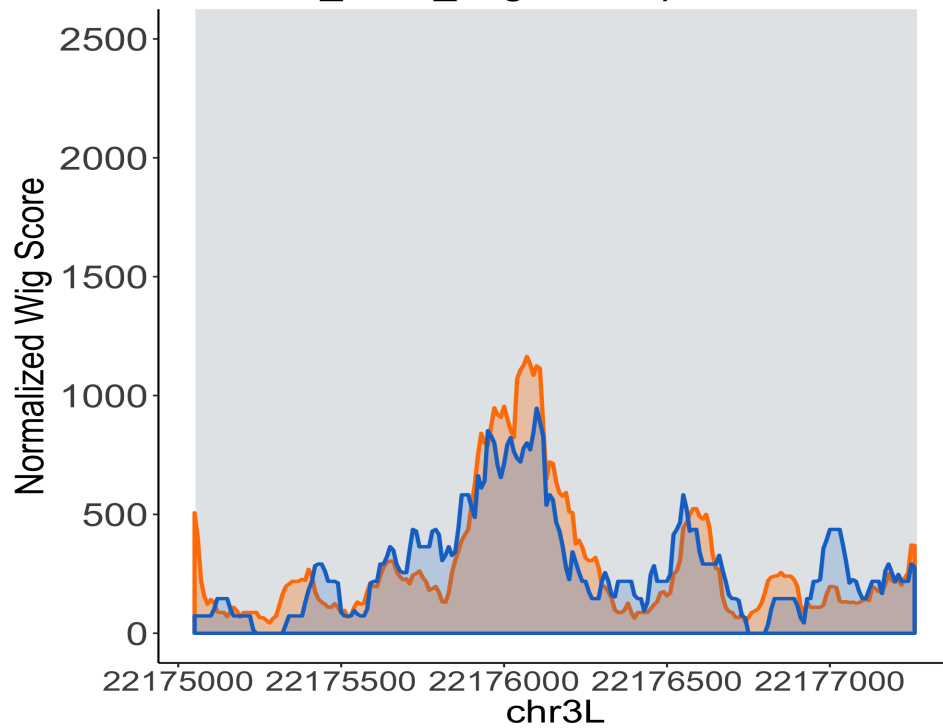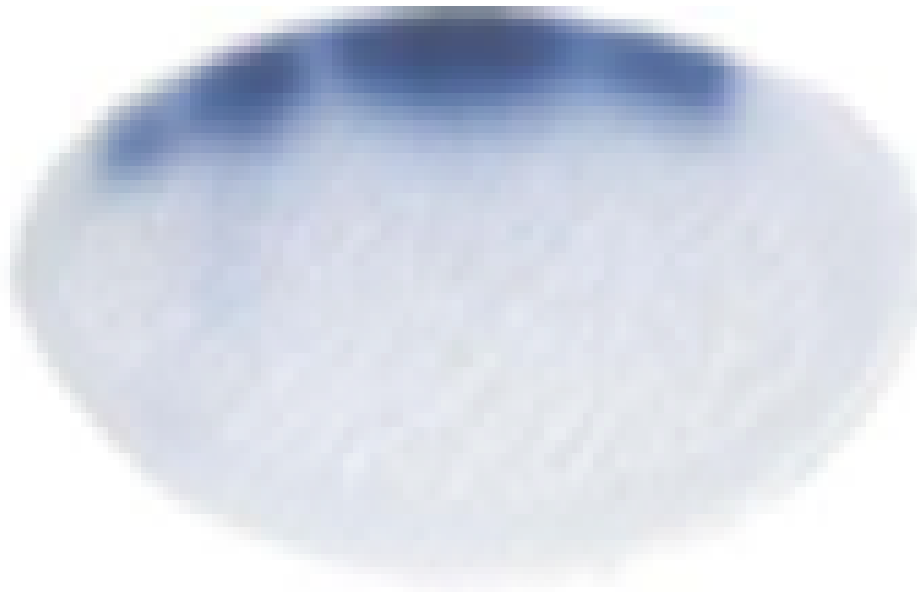

VT34695

Location: Dorsal Type: Enhancer ZScore: 0.057836726 PValue: 0.953878684

Supplement: S3 File — Reports consist of in situ hybridization images, ATAC-seq traces, and calculated p-value and Z Score for each region used in the final analysis. (ZIP) [file pgen.1007367.s015.zip › S3_File/VT34695_Kvon_Report.pdf]

## VT36350\_Kvon

VT36350\_Kvon\_WigFileSnapshot.txt

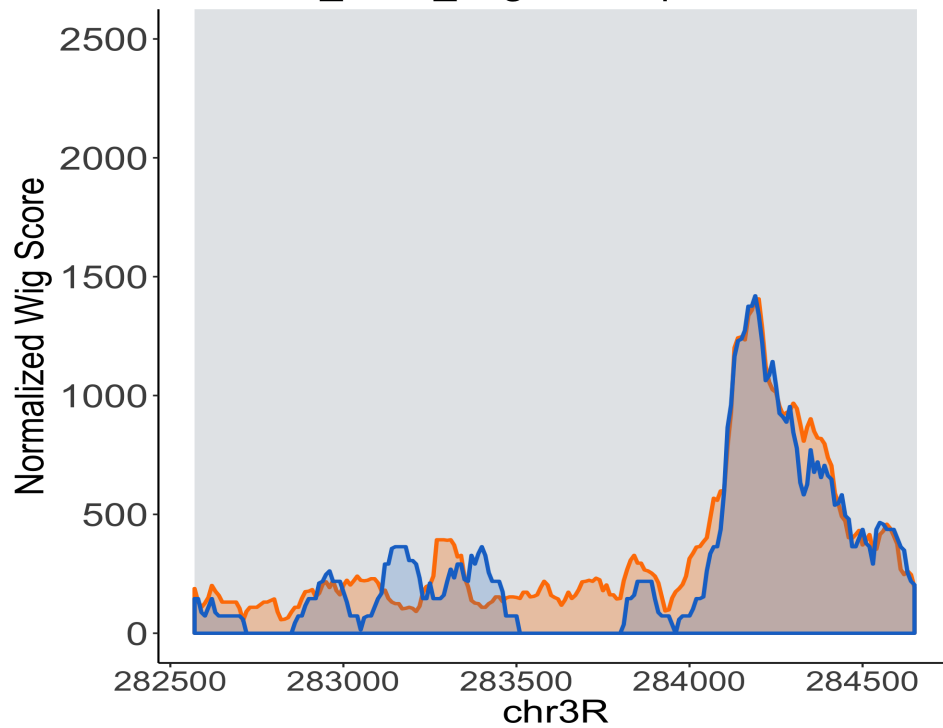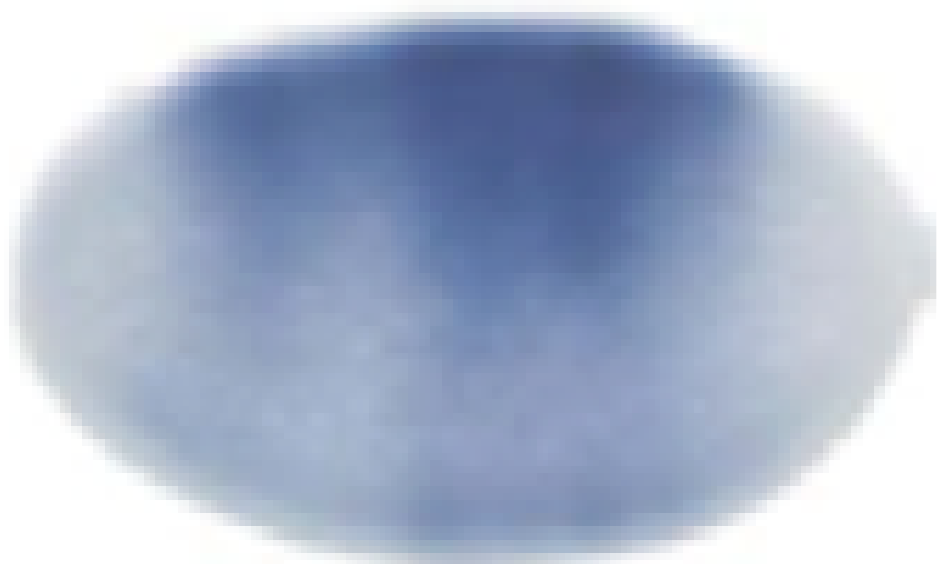

VT36350

Location: Dorsal Type: Enhancer ZScore: 0.389718388 PValue: 0.696744798

Supplement: S3 File — Reports consist of in situ hybridization images, ATAC-seq traces, and calculated p-value and Z Score for each region used in the final analysis. (ZIP) [file pgen.1007367.s015.zip › S3_File/VT36350_Kvon_Report.pdf]

VT37495\_Kvon

VT37495\_Kvon\_WigFileSnapshot.txt

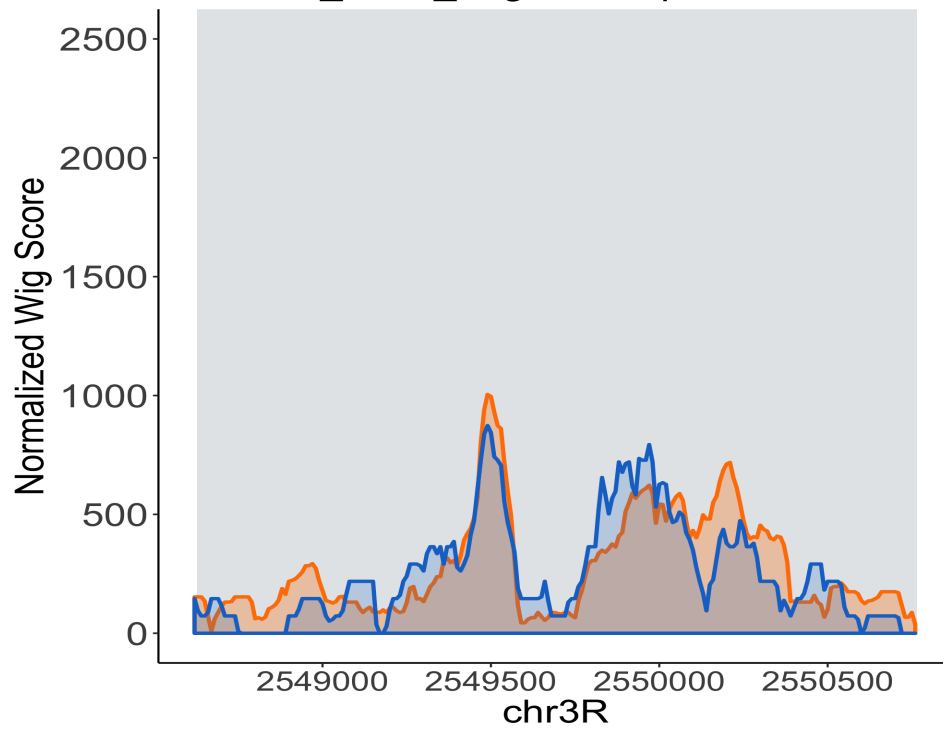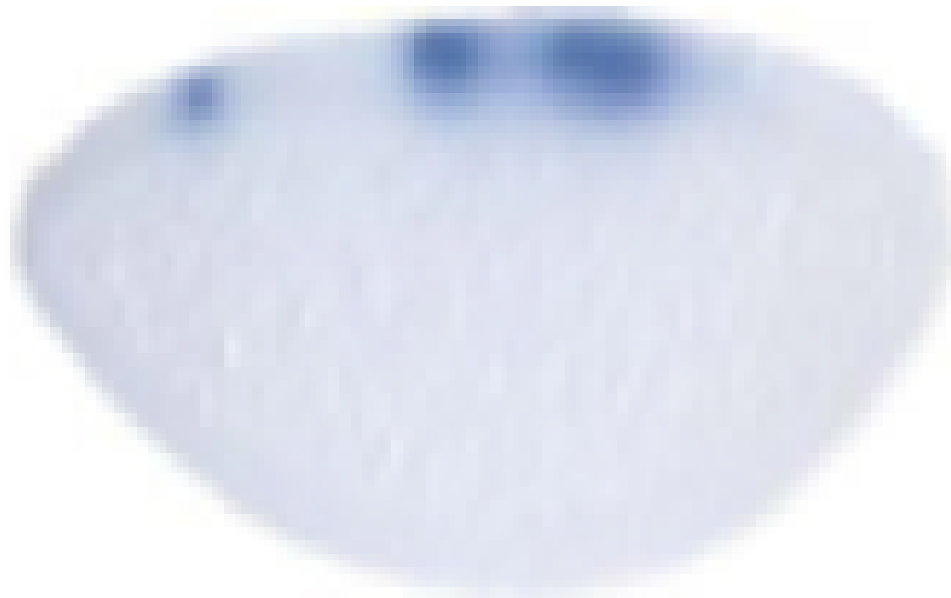

VT37495

Location: Dorsal Type: Enhancer ZScore: 0.178094021 PValue: 0.858649137

Supplement: S3 File — Reports consist of in situ hybridization images, ATAC-seq traces, and calculated p-value and Z Score for each region used in the final analysis. (ZIP) [file pgen.1007367.s015.zip › S3_File/VT37495_Kvon_Report.pdf]

VT42486

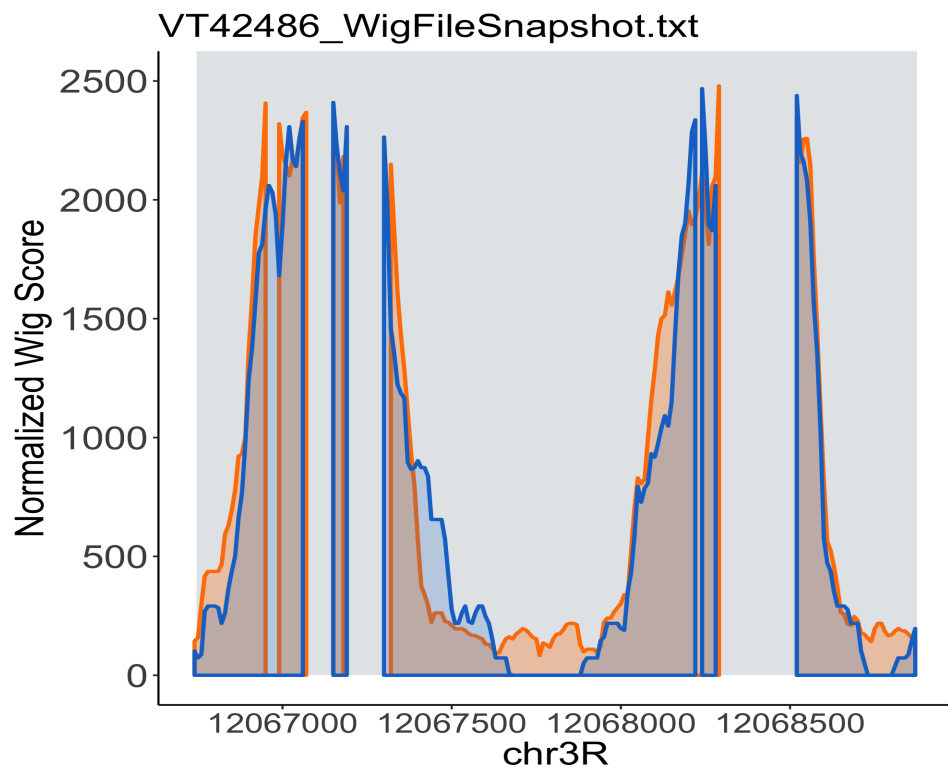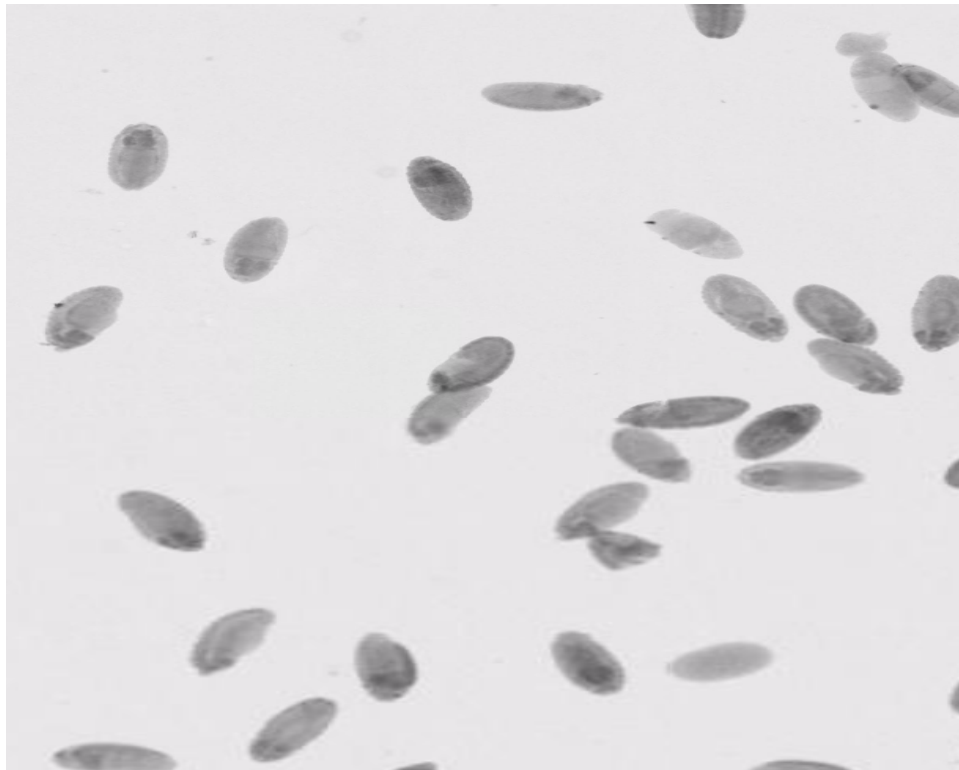

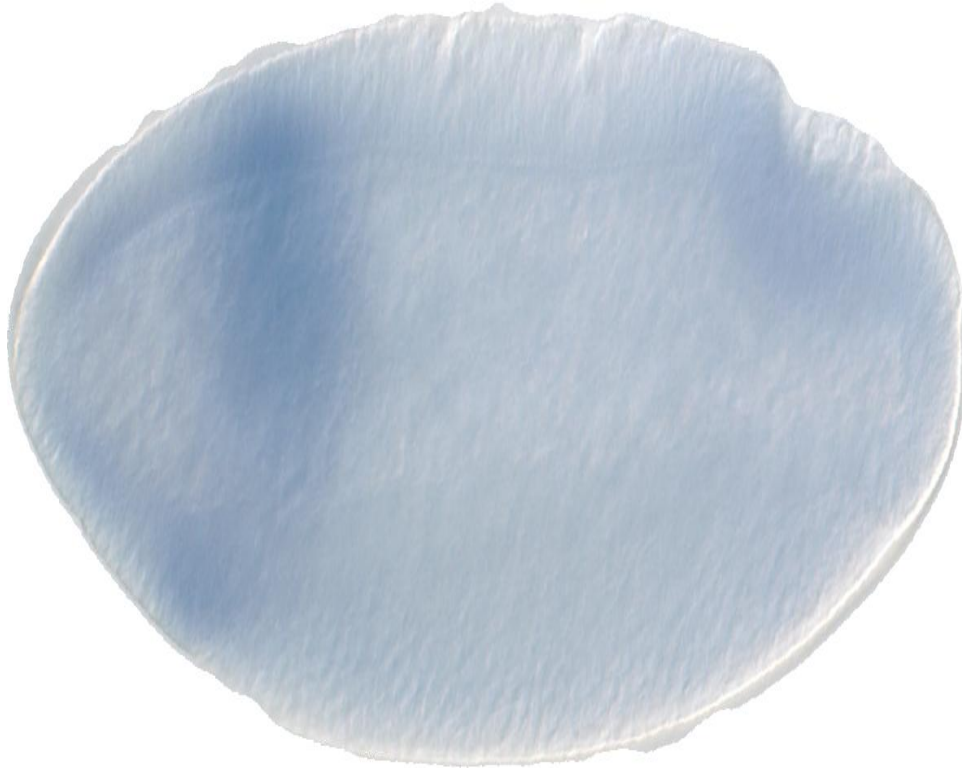

Location: Mostly Ant Type: Enhancer ZScore: 0.064754032 PValue: 0.948369842

Supplement: S3 File — Reports consist of in situ hybridization images, ATAC-seq traces, and calculated p-value and Z Score for each region used in the final analysis. (ZIP) [file pgen.1007367.s015.zip › S3_File/VT42486_Report.pdf]

## VT42492\_Kvon

VT42492\_Kvon\_WigFileSnapshot.txt

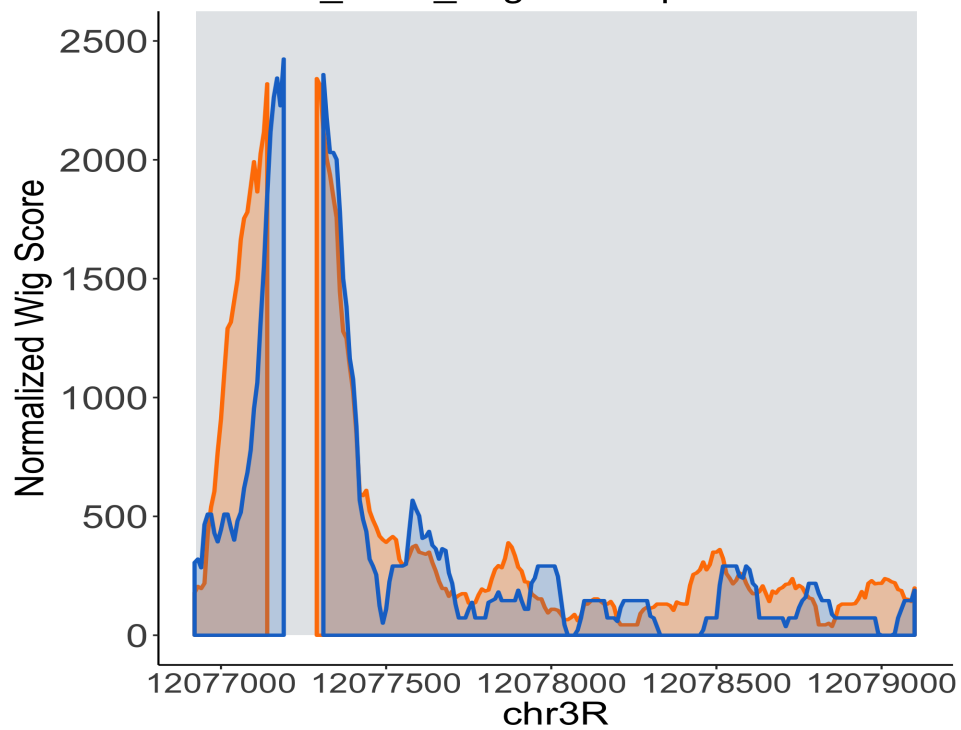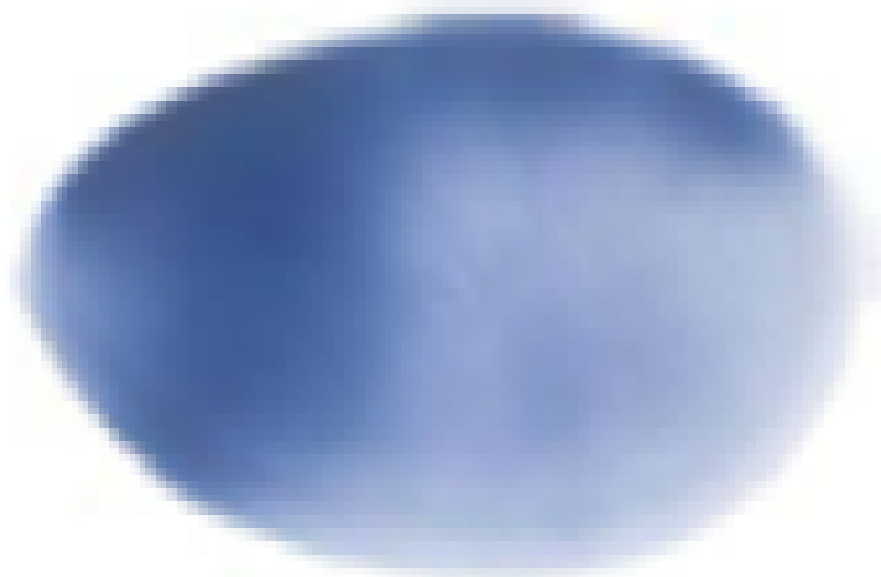

VT42492

Location: Mostly Ant Type: Enhancer ZScore: 0.456369084 PValue: 0.648124594

Supplement: S3 File — Reports consist of in situ hybridization images, ATAC-seq traces, and calculated p-value and Z Score for each region used in the final analysis. (ZIP) [file pgen.1007367.s015.zip › S3_File/VT42492_Kvon_Report.pdf]

VT42832

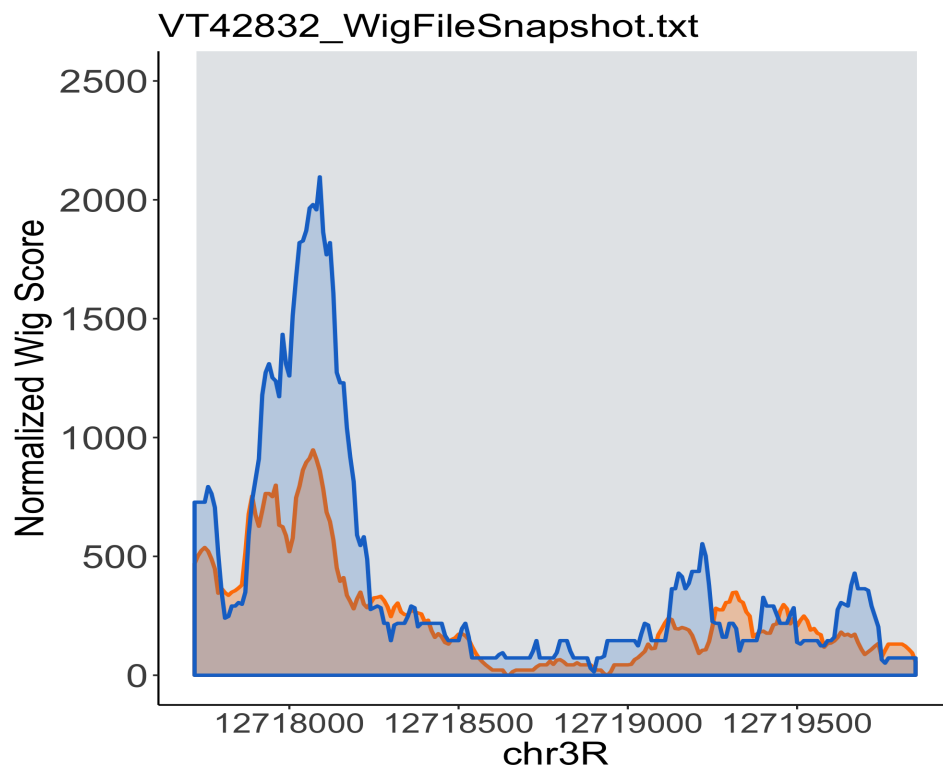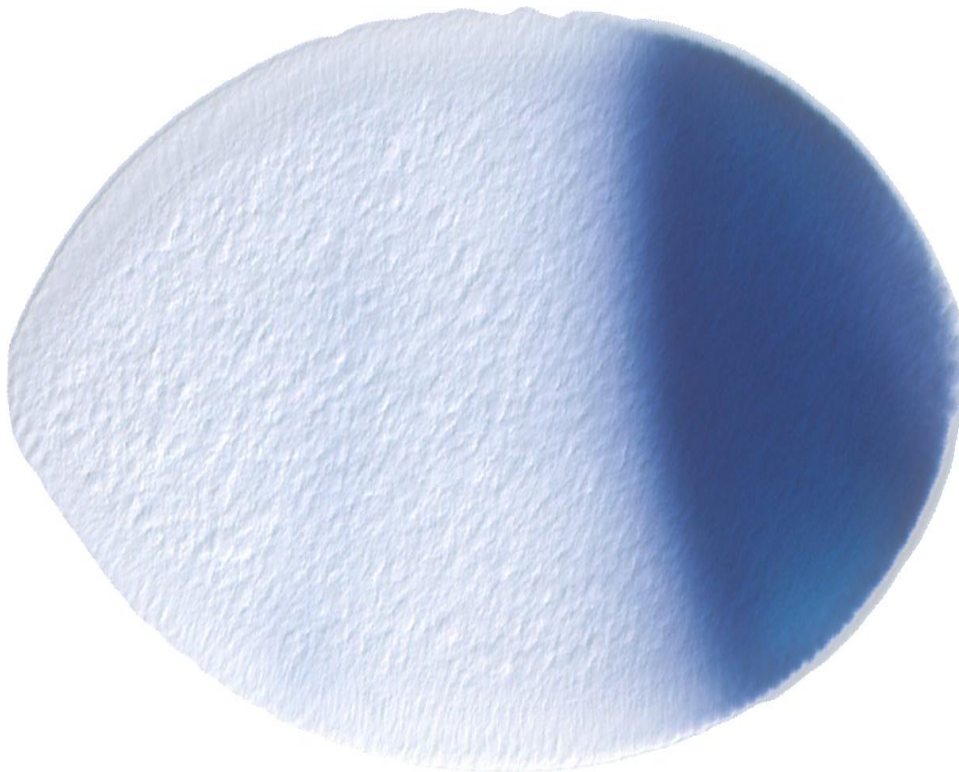

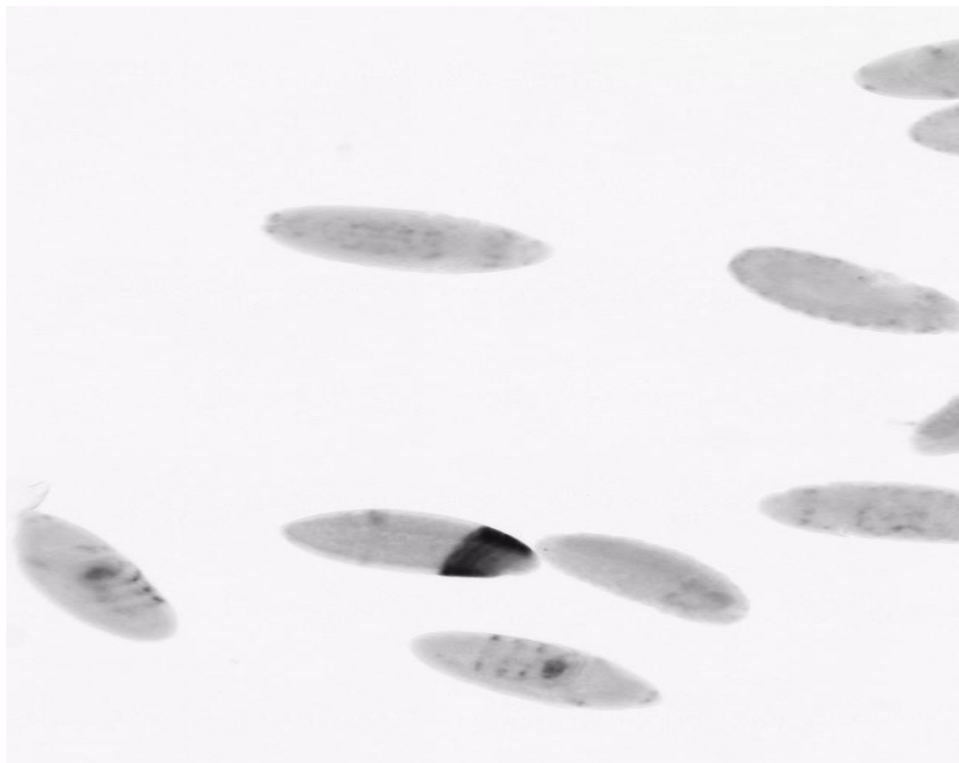

Location: Posterior Type: Enhancer ZScore: 1.00868099 PValue: 0.313127651

Supplement: S3 File — Reports consist of in situ hybridization images, ATAC-seq traces, and calculated p-value and Z Score for each region used in the final analysis. (ZIP) [file pgen.1007367.s015.zip › S3_File/VT42832_Report.pdf]

VT42837

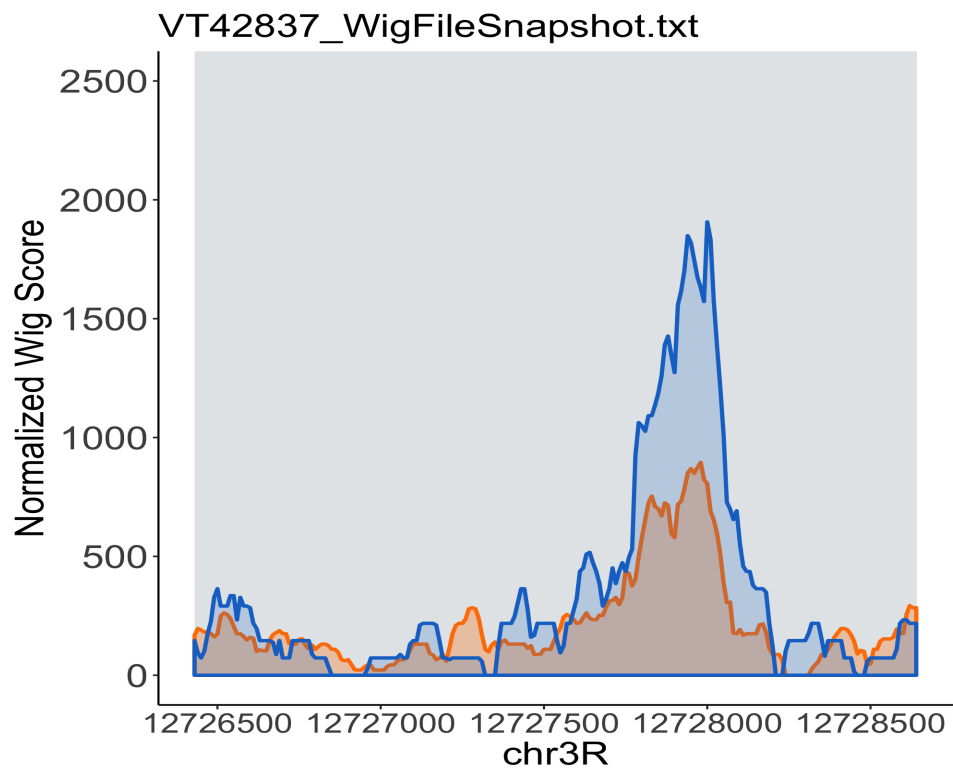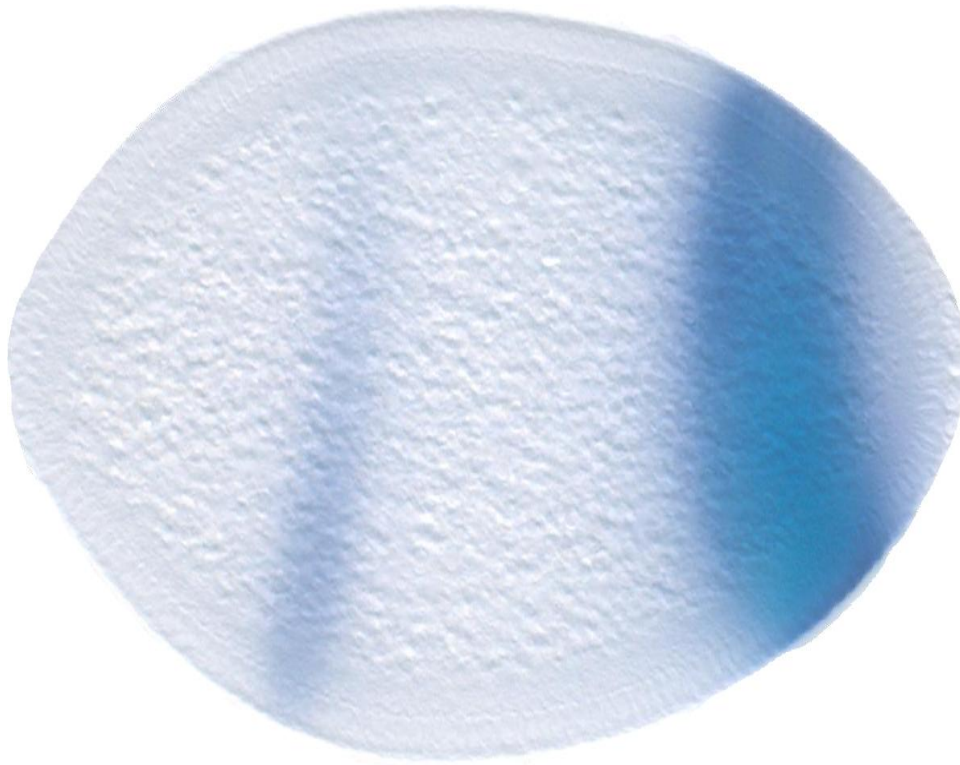

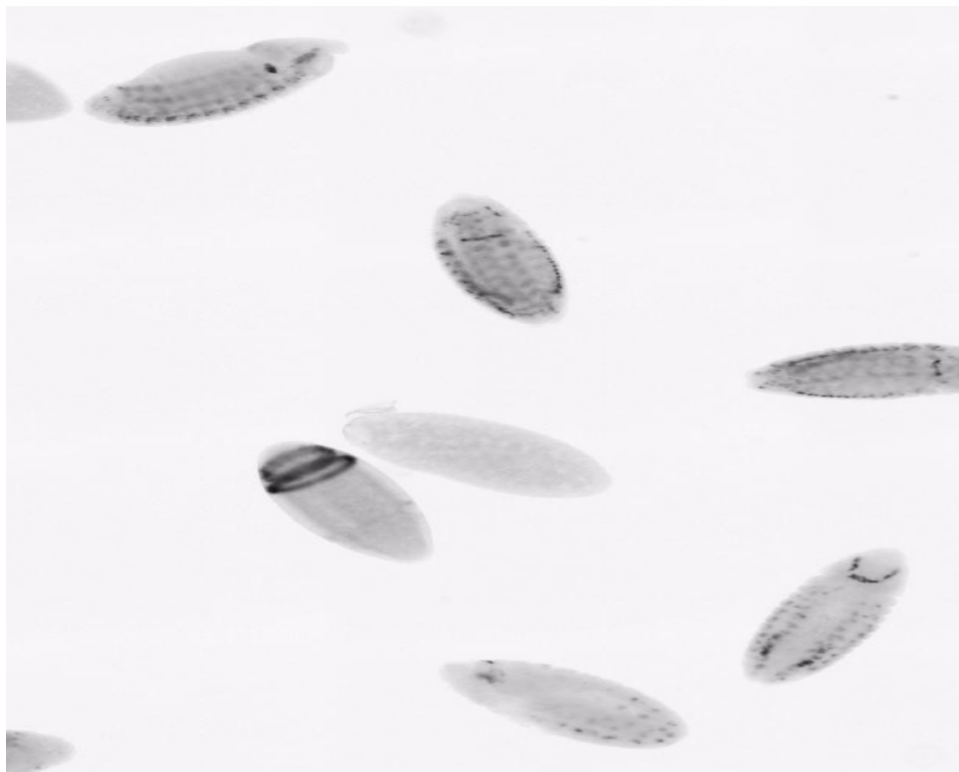

Location: Mostly Post Type: Enhancer ZScore: 0.967556026 PValue: 0.333266148

Supplement: S3 File — Reports consist of in situ hybridization images, ATAC-seq traces, and calculated p-value and Z Score for each region used in the final analysis. (ZIP) [file pgen.1007367.s015.zip › S3_File/VT42837_Report.pdf]

# VT44117\_Kvon

VT44117\_Kvon\_WigFileSnapshot.txt

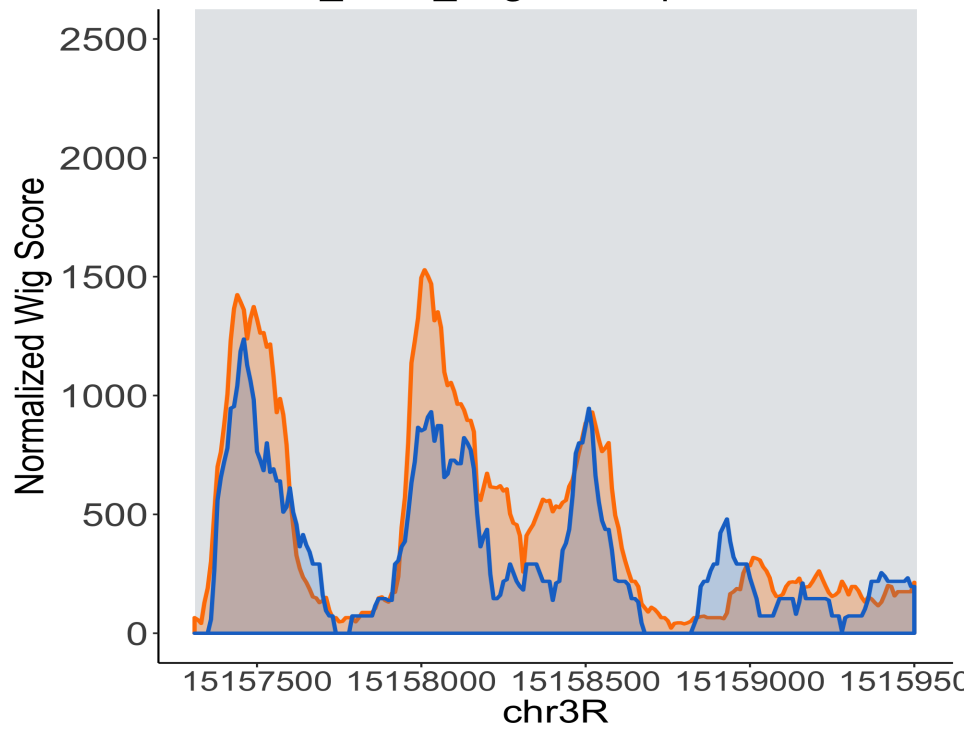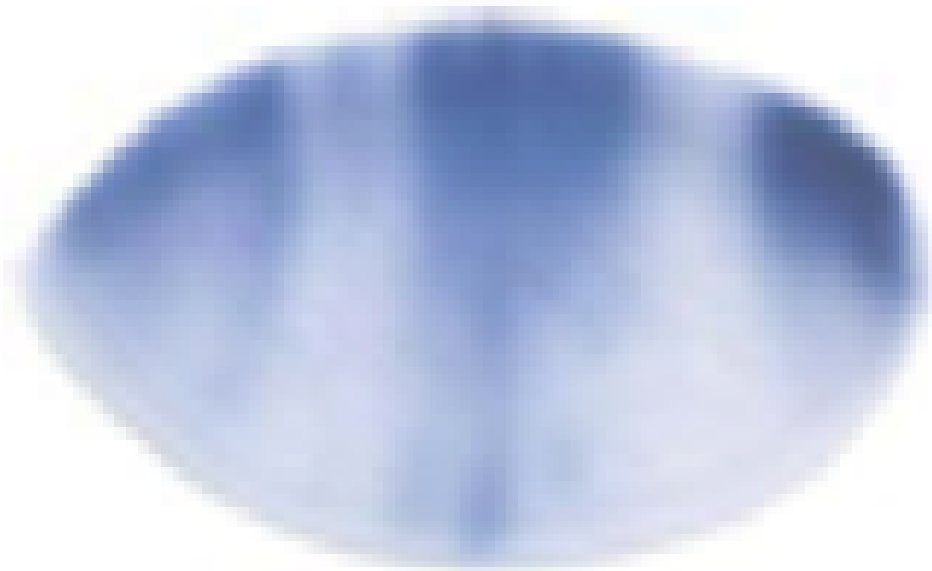

VT44117

Location: Dorsal Type: Enhancer ZScore: 0.678158591 PValue: 0.497671144

Supplement: S3 File — Reports consist of in situ hybridization images, ATAC-seq traces, and calculated p-value and Z Score for each region used in the final analysis. (ZIP) [file pgen.1007367.s015.zip › S3_File/VT44117_Kvon_Report.pdf]

VT4448

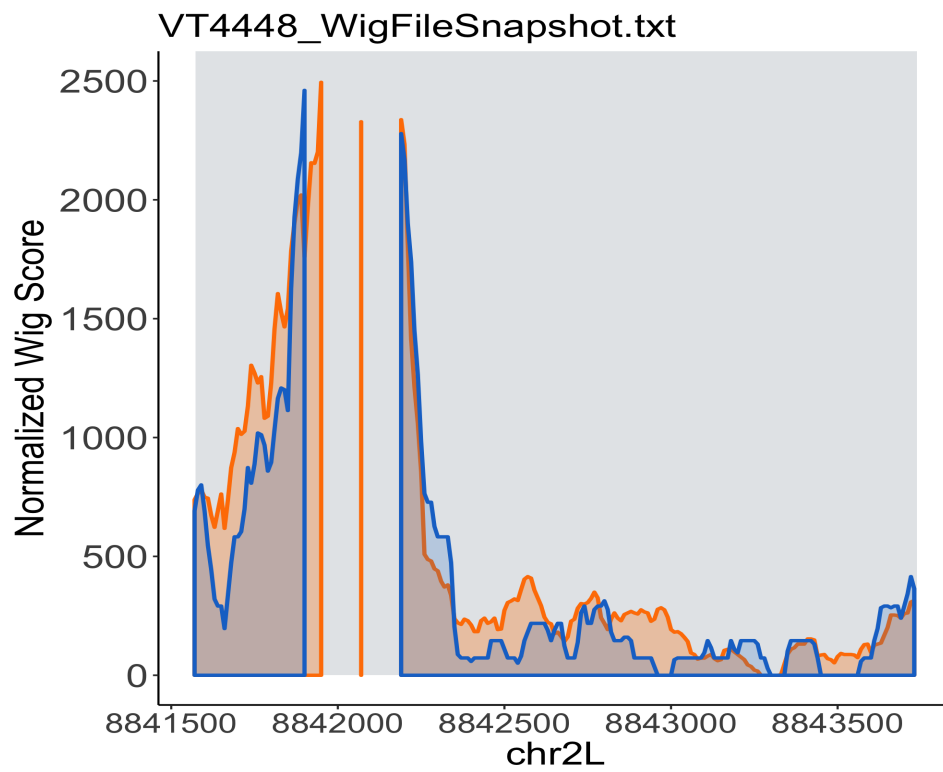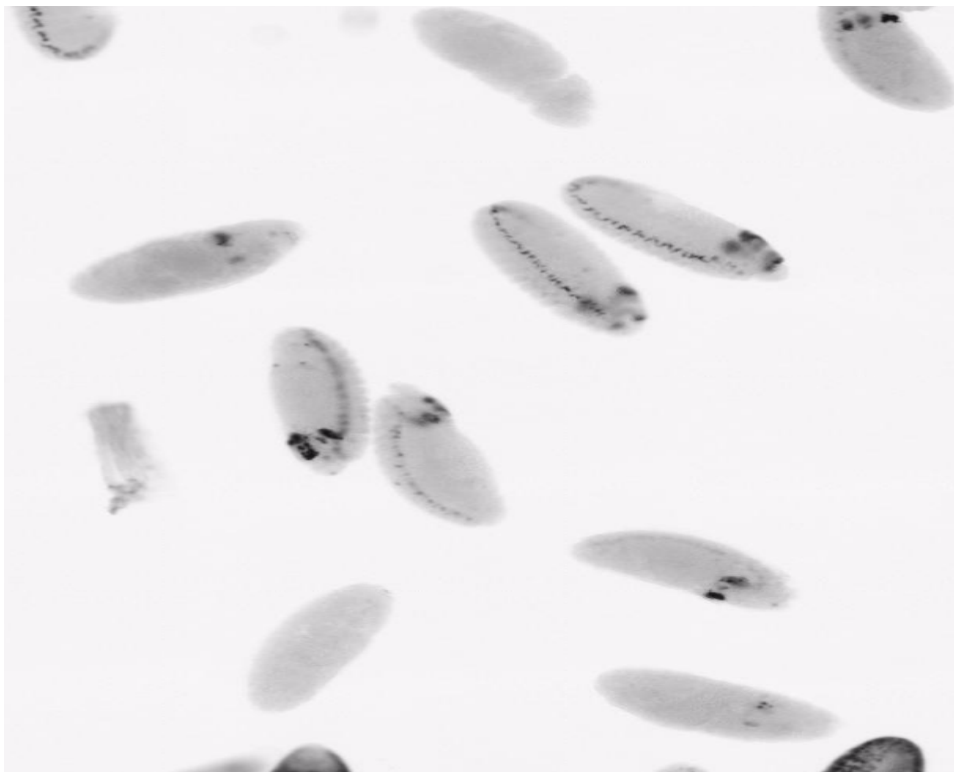

Location: Ventral Type: Enhancer ZScore: -0.062068858 PValue: 0.950507997

Supplement: S3 File — Reports consist of in situ hybridization images, ATAC-seq traces, and calculated p-value and Z Score for each region used in the final analysis. (ZIP) [file pgen.1007367.s015.zip › S3_File/VT4448_Report.pdf]

VT47407

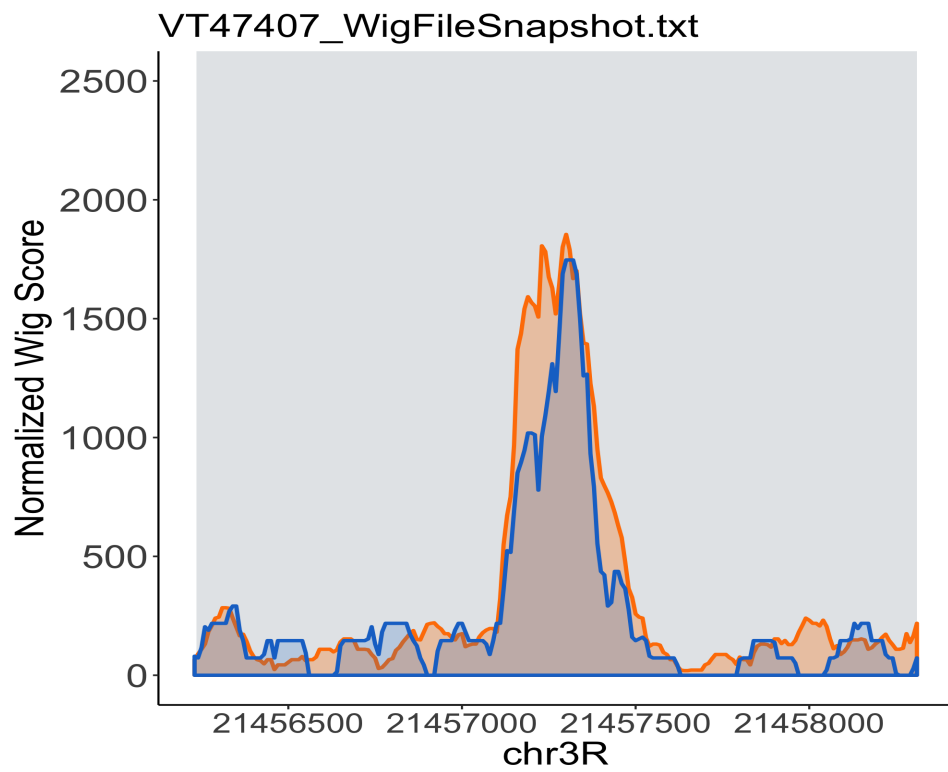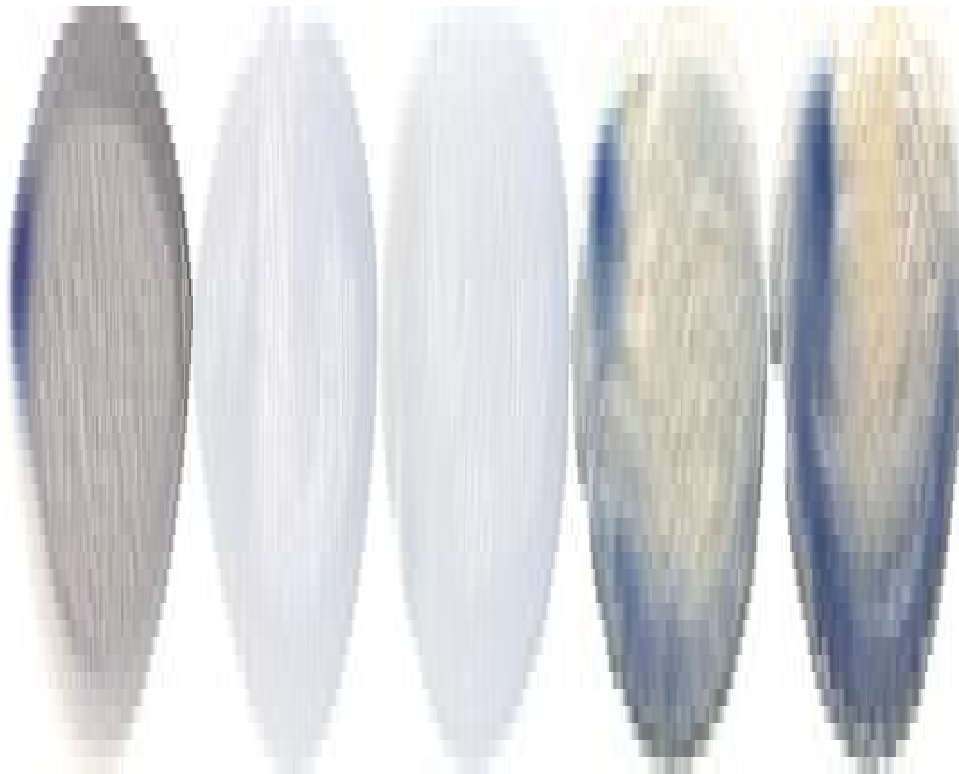

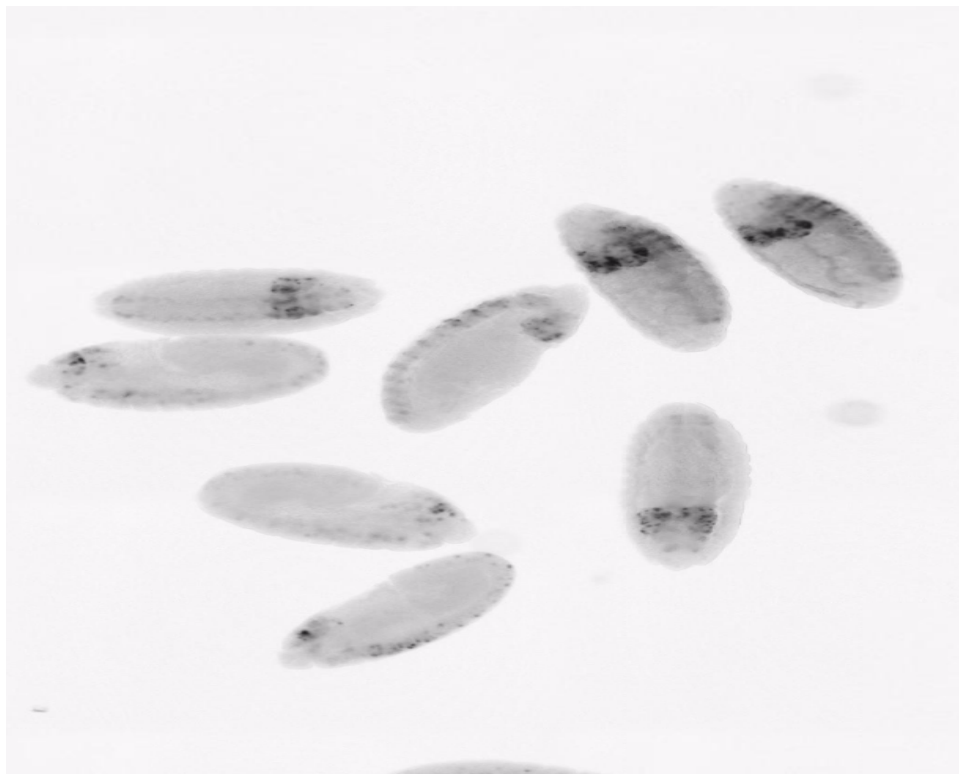

Location: Anterior Type: Enhancer ZScore: 0.567705456 PValue: 0.570234986

Supplement: S3 File — Reports consist of in situ hybridization images, ATAC-seq traces, and calculated p-value and Z Score for each region used in the final analysis. (ZIP) [file pgen.1007367.s015.zip › S3_File/VT47407_Report.pdf]

VT49279\_Kvon

VT49279\_Kvon\_WigFileSnapshot.txt

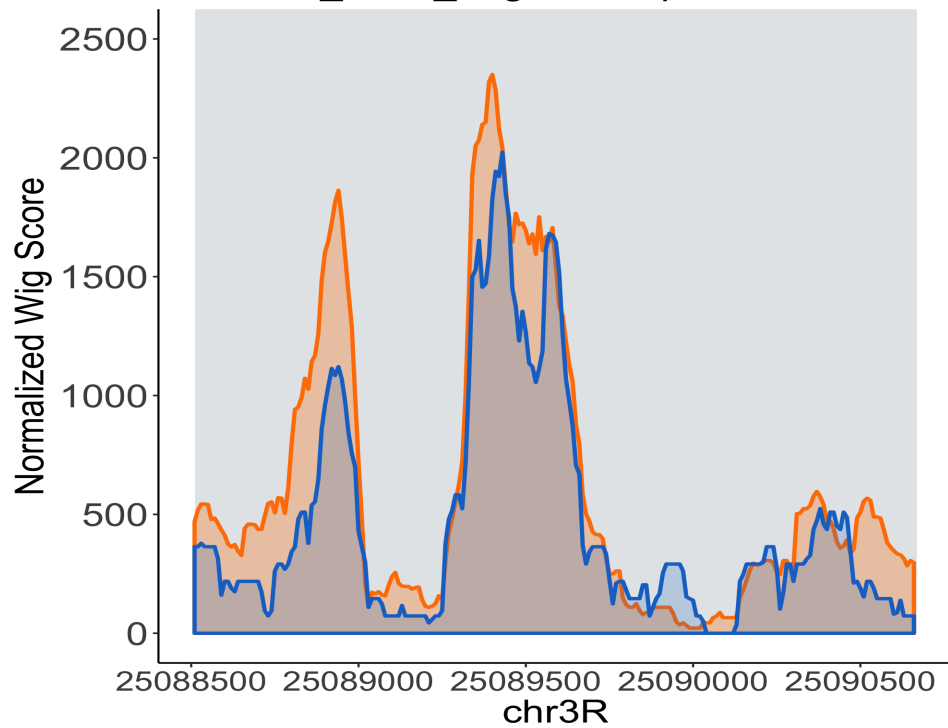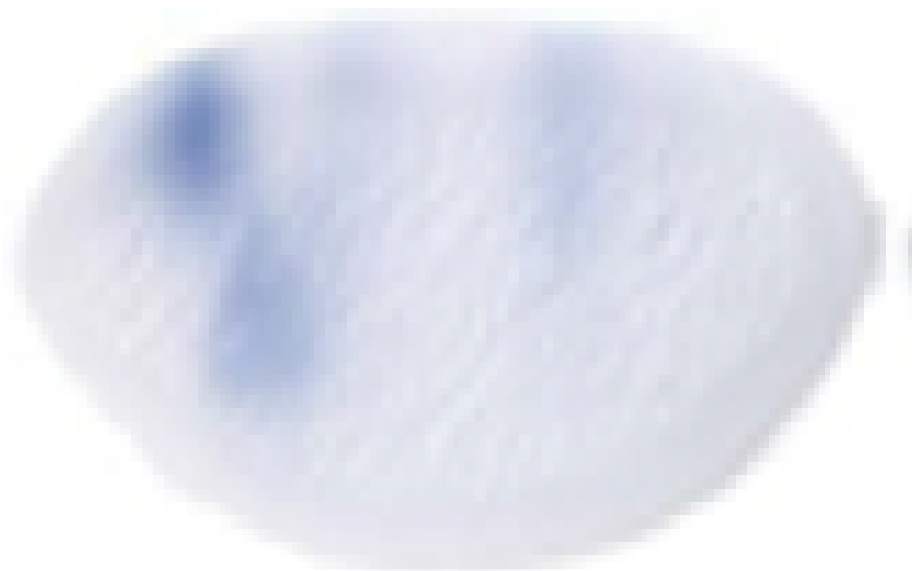

VT49279

Location: Anterior Type: Enhancer ZScore: 0.662879706 PValue: 0.5074076

Supplement: S3 File — Reports consist of in situ hybridization images, ATAC-seq traces, and calculated p-value and Z Score for each region used in the final analysis. (ZIP) [file pgen.1007367.s015.zip › S3_File/VT49279_Kvon_Report.pdf]

VT57022\_Kvon

VT57022\_Kvon\_WigFileSnapshot.txt

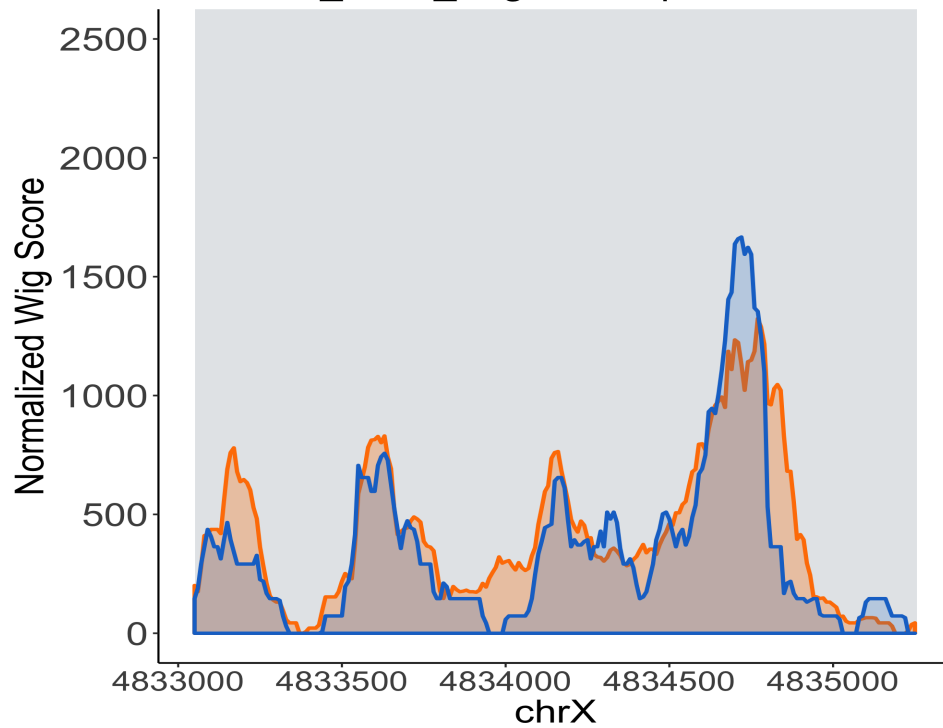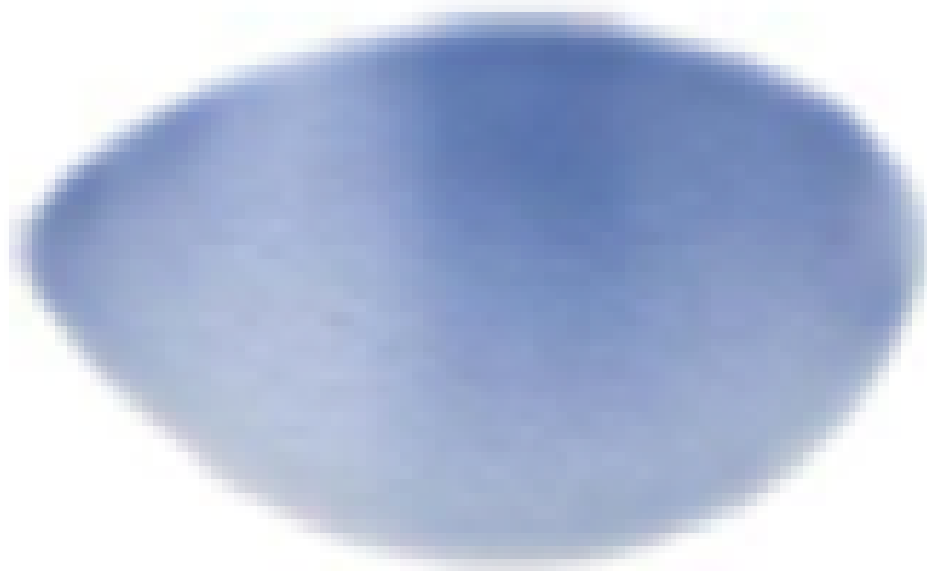

VT57022

Location: Dorsal Type: Enhancer ZScore: 0.365074983 PValue: 0.715055434

Supplement: S3 File — Reports consist of in situ hybridization images, ATAC-seq traces, and calculated p-value and Z Score for each region used in the final analysis. (ZIP) [file pgen.1007367.s015.zip › S3_File/VT57022_Kvon_Report.pdf]

VT6477\_Kvon

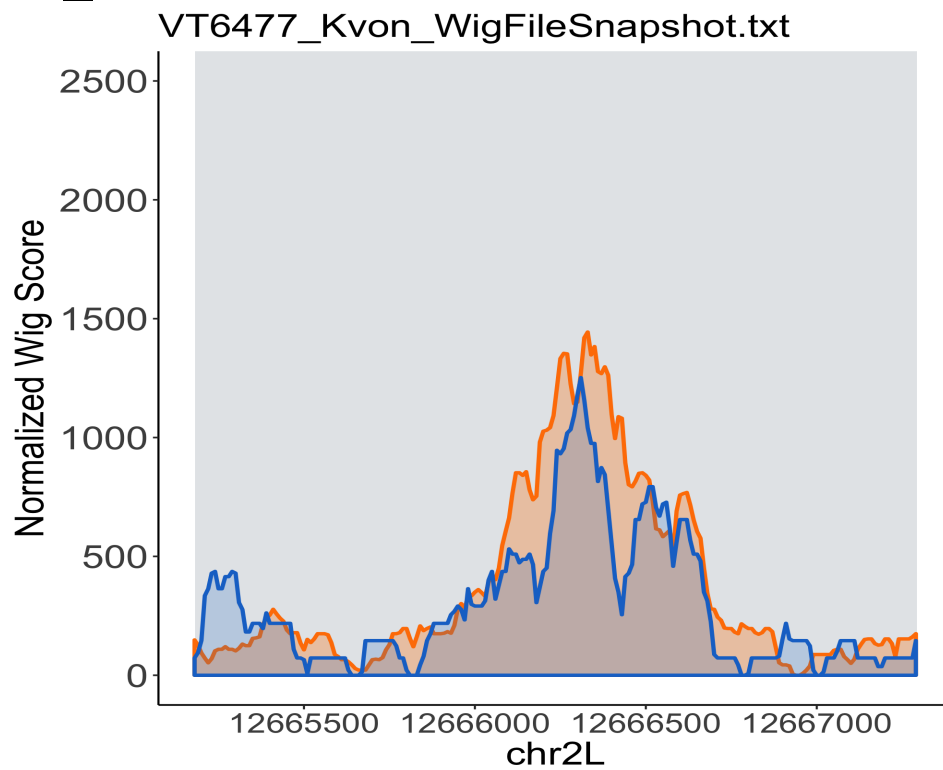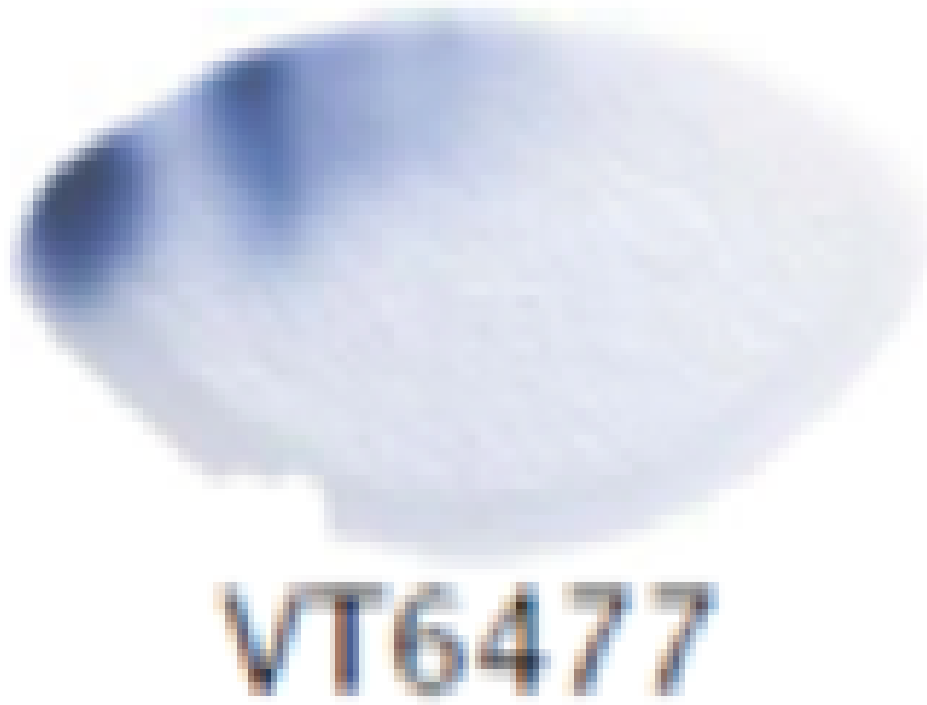

Location: Anterior Type: Enhancer ZScore: 0.505812472 PValue: 0.612988302

Supplement: S3 File — Reports consist of in situ hybridization images, ATAC-seq traces, and calculated p-value and Z Score for each region used in the final analysis. (ZIP) [file pgen.1007367.s015.zip › S3_File/VT6477_Kvon_Report.pdf]

VT7842

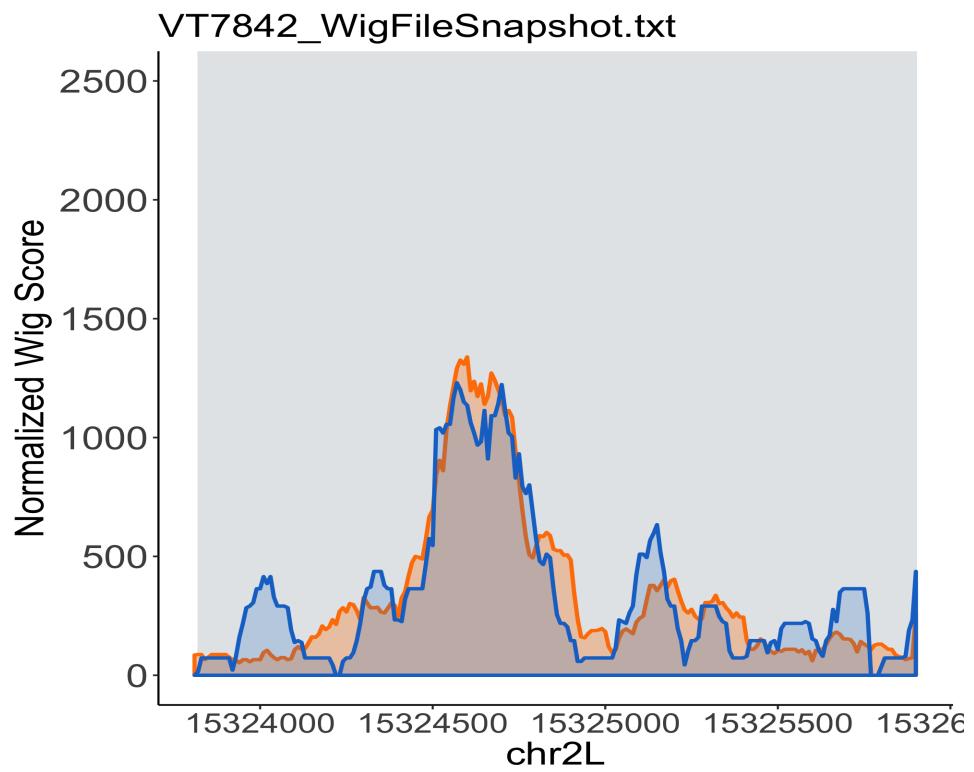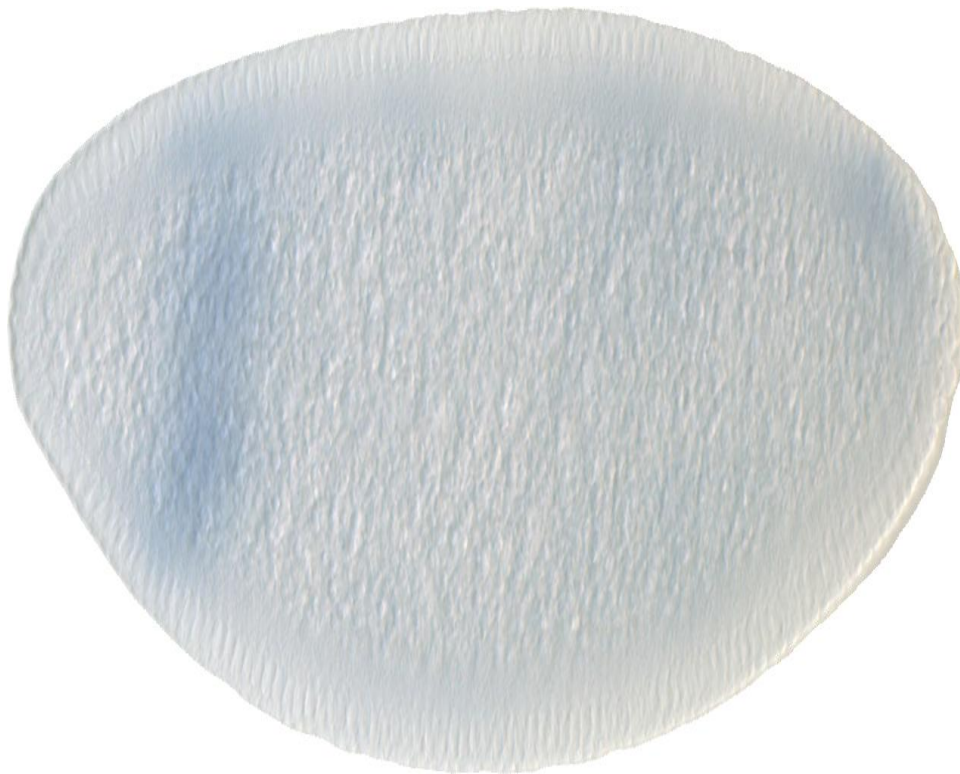

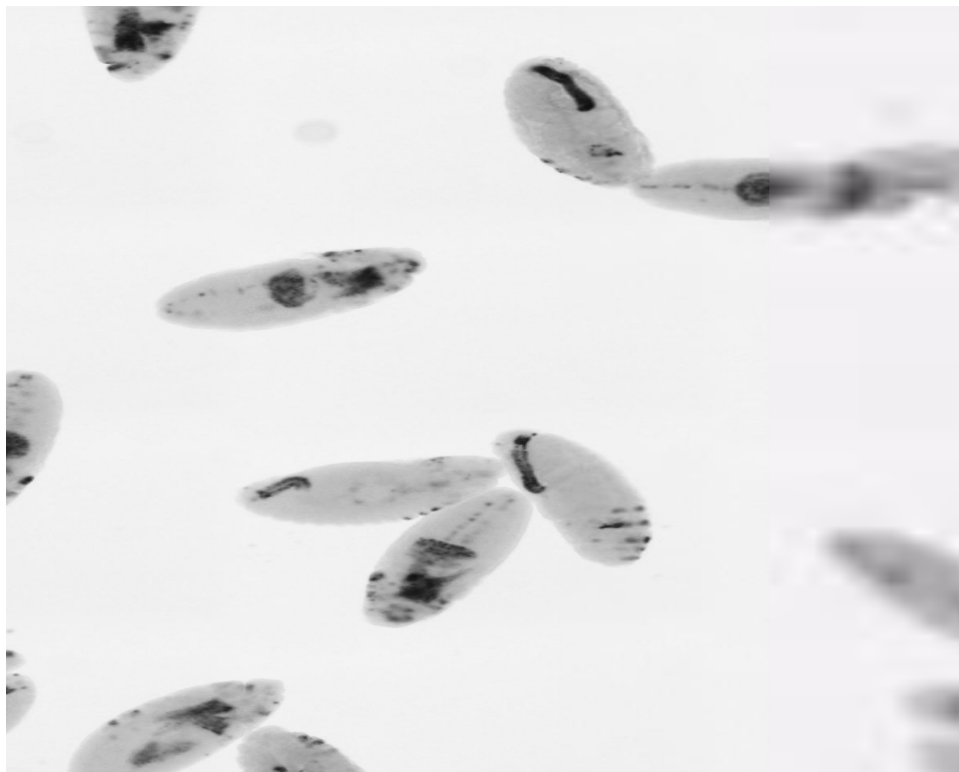

Location: Mostly Ant Type: Enhancer ZScore: -0.025196233 PValue: 0.979898442

Supplement: S3 File — Reports consist of in situ hybridization images, ATAC-seq traces, and calculated p-value and Z Score for each region used in the final analysis. (ZIP) [file pgen.1007367.s015.zip › S3_File/VT7842_Report.pdf]

## VT8889\_Kvon

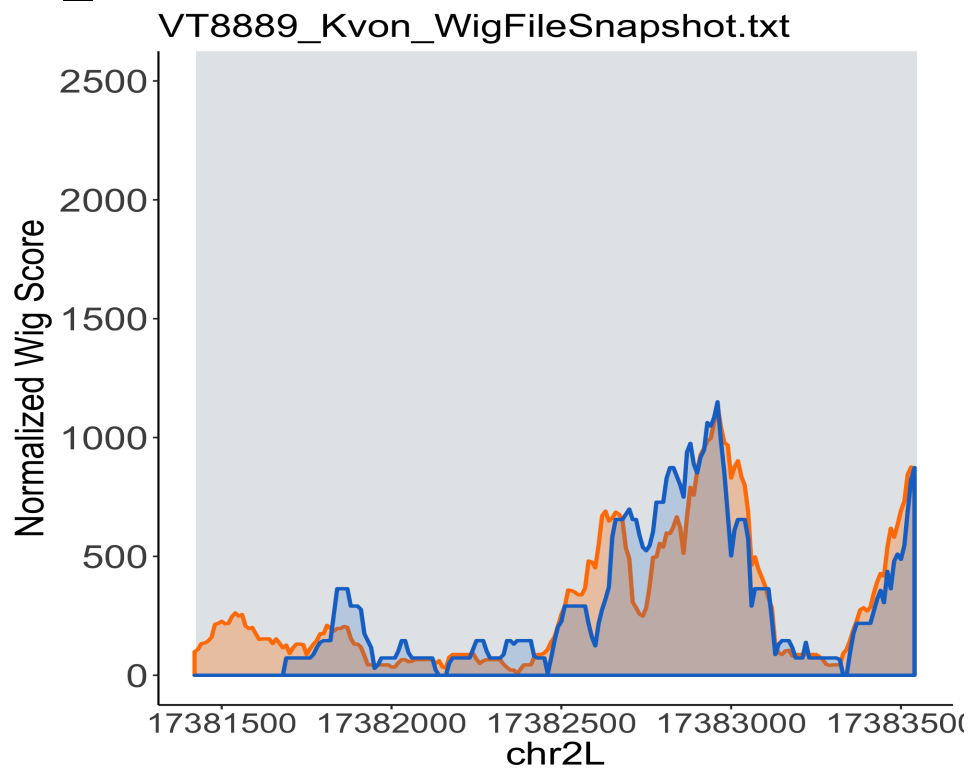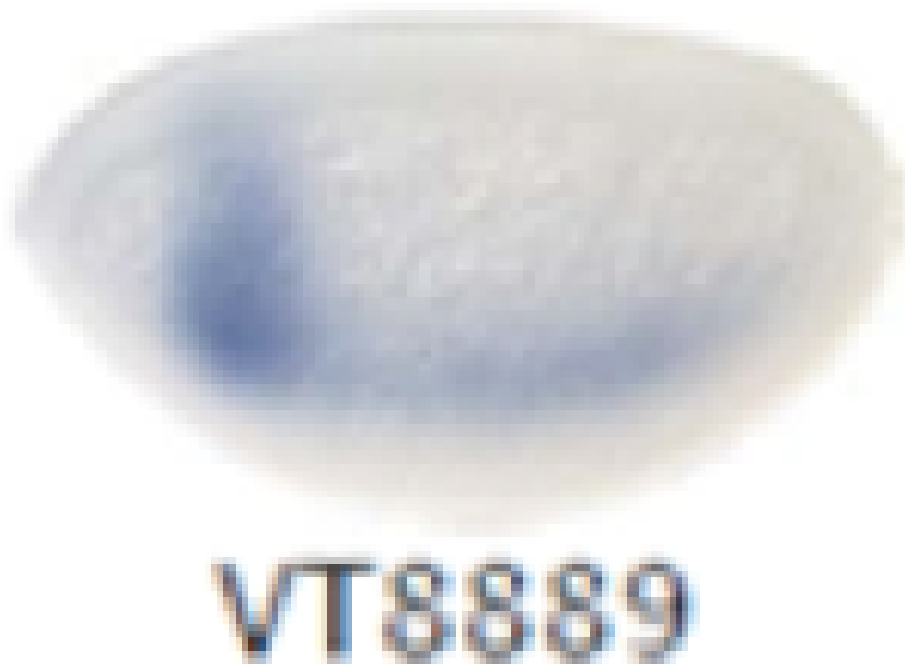

Location: Ventral Type: Enhancer ZScore: 0.127303288 PValue: 0.898700358

Supplement: S3 File — Reports consist of in situ hybridization images, ATAC-seq traces, and calculated p-value and Z Score for each region used in the final analysis. (ZIP) [file pgen.1007367.s015.zip › S3_File/VT8889_Kvon_Report.pdf]

VT9677\_Kvon

VT9677\_Kvon\_WigFileSnapshot.txt

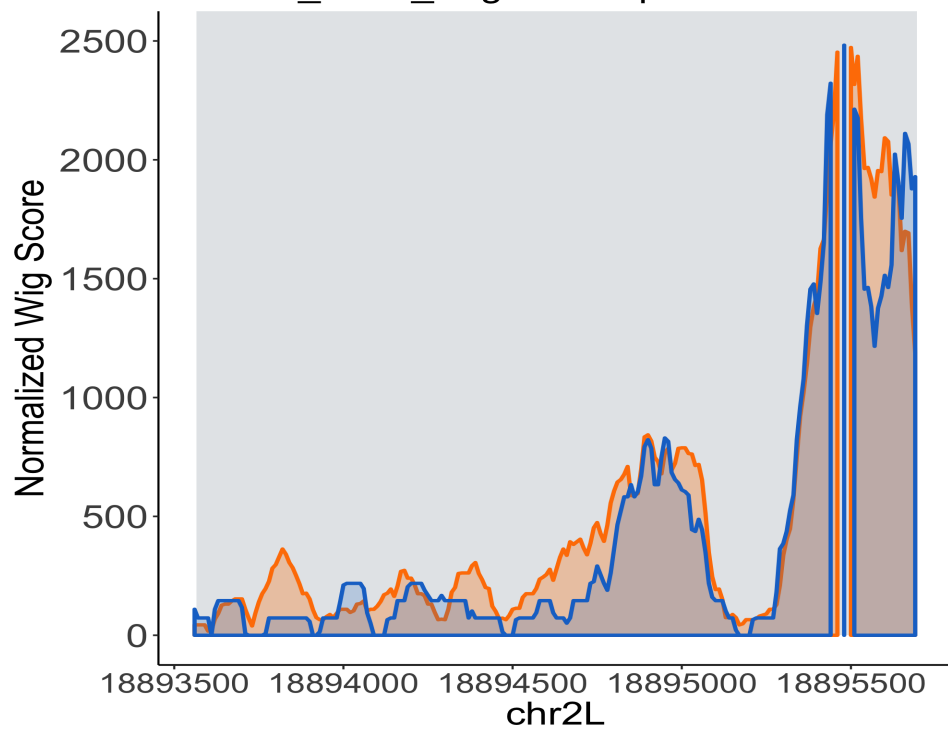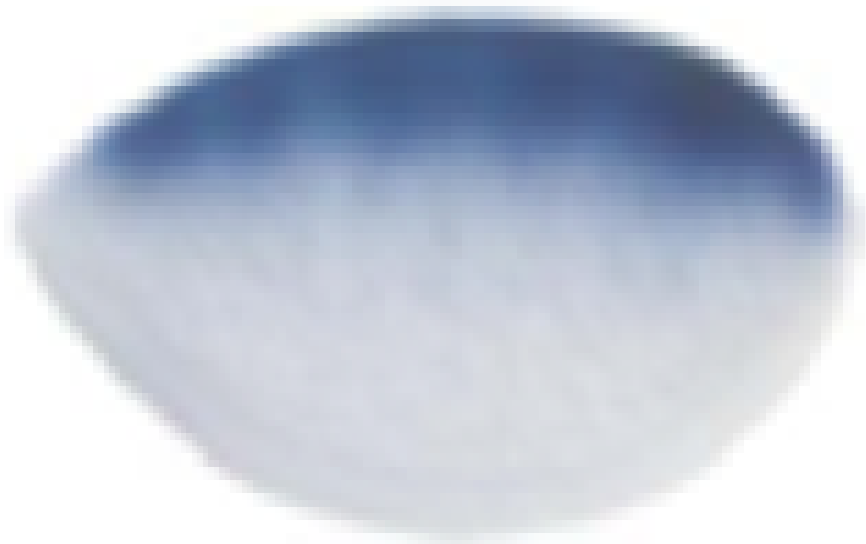

VT9677

Location: Dorsal Type: Enhancer ZScore: 0.2942456 PValue: 0.768570238

Supplement: S3 File — Reports consist of in situ hybridization images, ATAC-seq traces, and calculated p-value and Z Score for each region used in the final analysis. (ZIP) [file pgen.1007367.s015.zip › S3_File/VT9677_Kvon_Report.pdf]

wgn

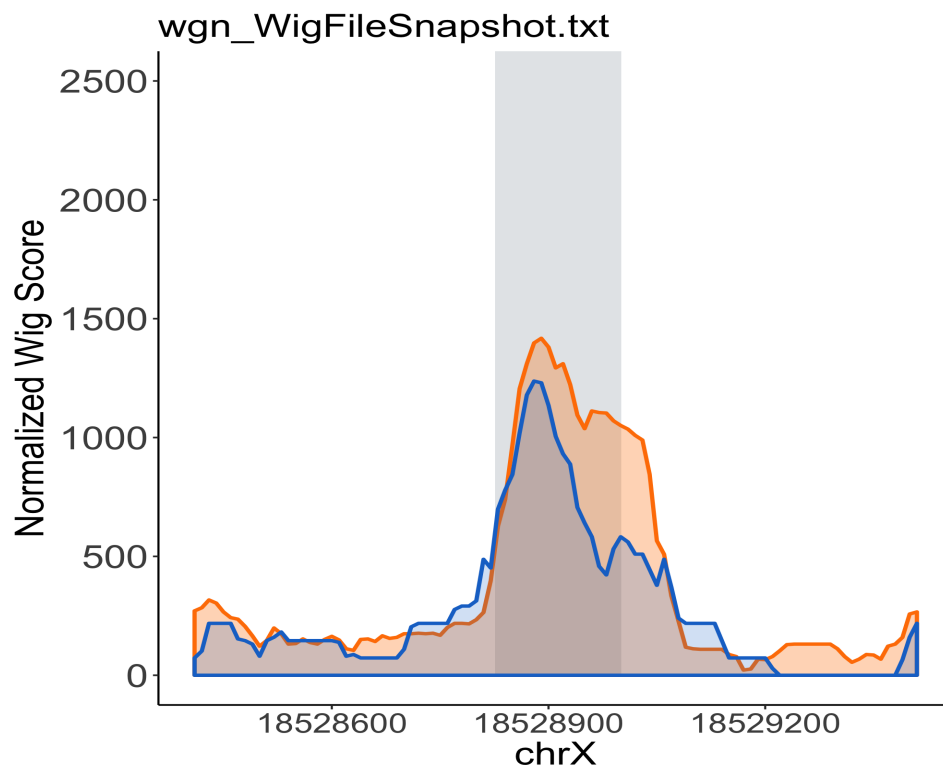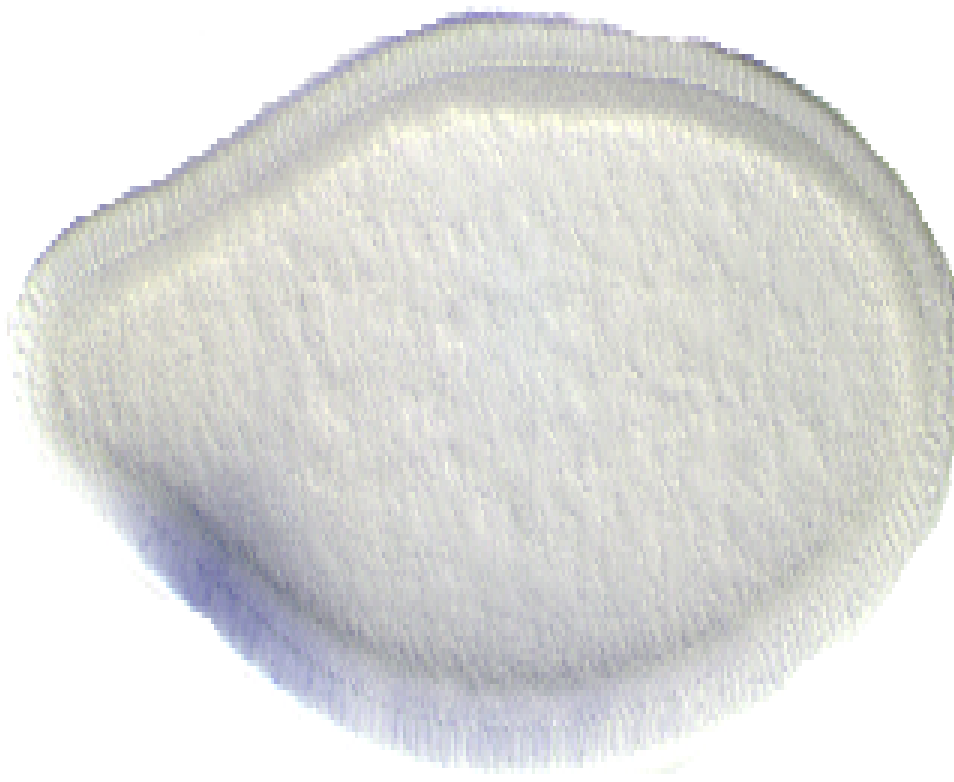

Location: Ventral Type: Promoter ZScore: 0.655884238 PValue: 0.511898611

Supplement: S3 File — Reports consist of in situ hybridization images, ATAC-seq traces, and calculated p-value and Z Score for each region used in the final analysis. (ZIP) [file pgen.1007367.s015.zip › S3_File/wgn_Report.pdf]

## wntD\_Zeitlinger

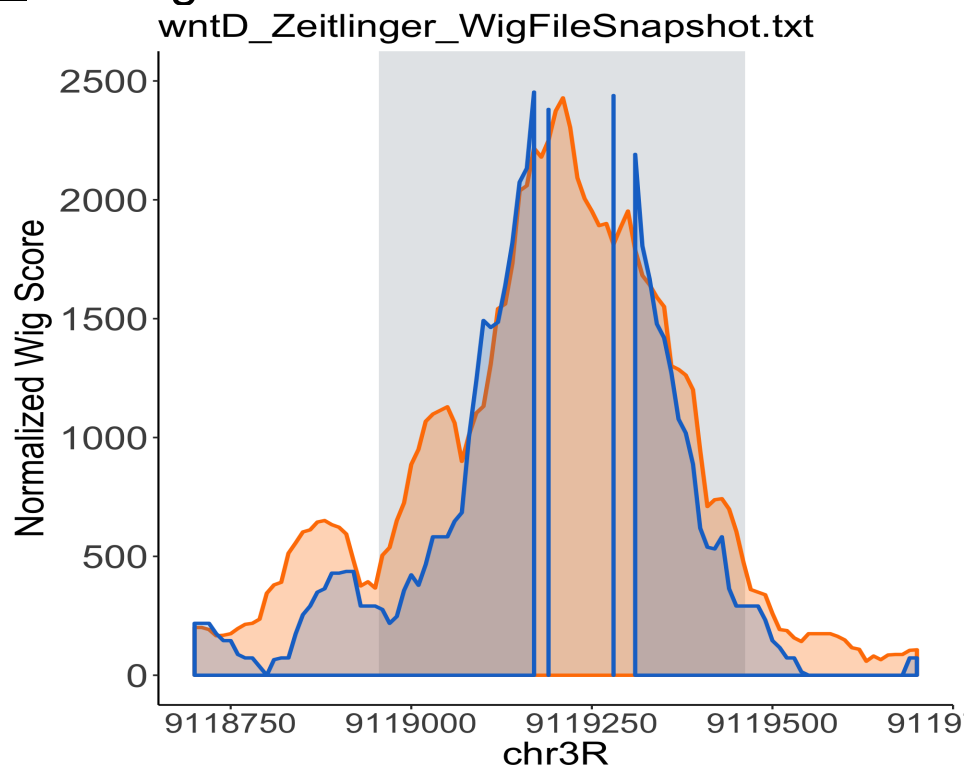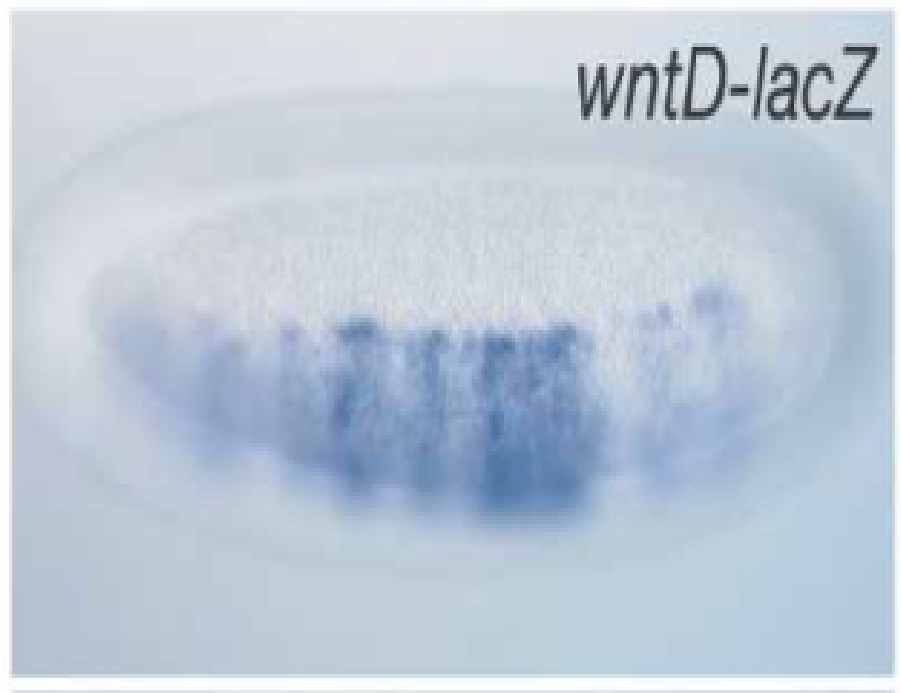

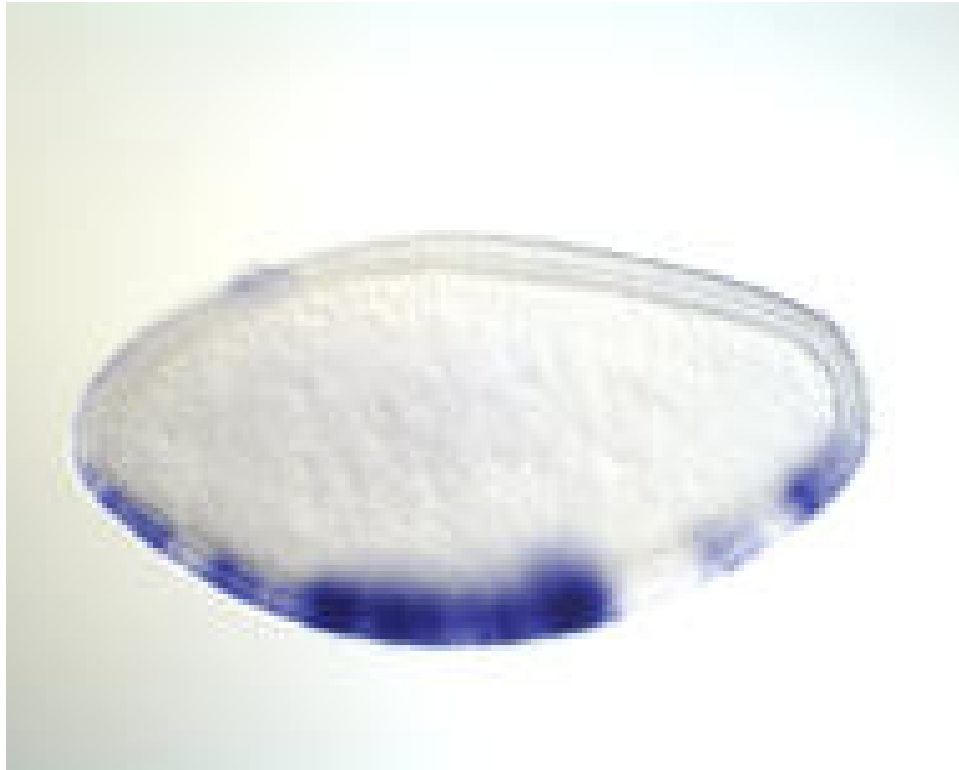

Location: Ventral Type: Enhancer ZScore: -0.092259736 PValue: 0.926491678

Supplement: S3 File — Reports consist of in situ hybridization images, ATAC-seq traces, and calculated p-value and Z Score for each region used in the final analysis. (ZIP) [file pgen.1007367.s015.zip › S3_File/wntD_Zeitlinger_Report.pdf]

zen2

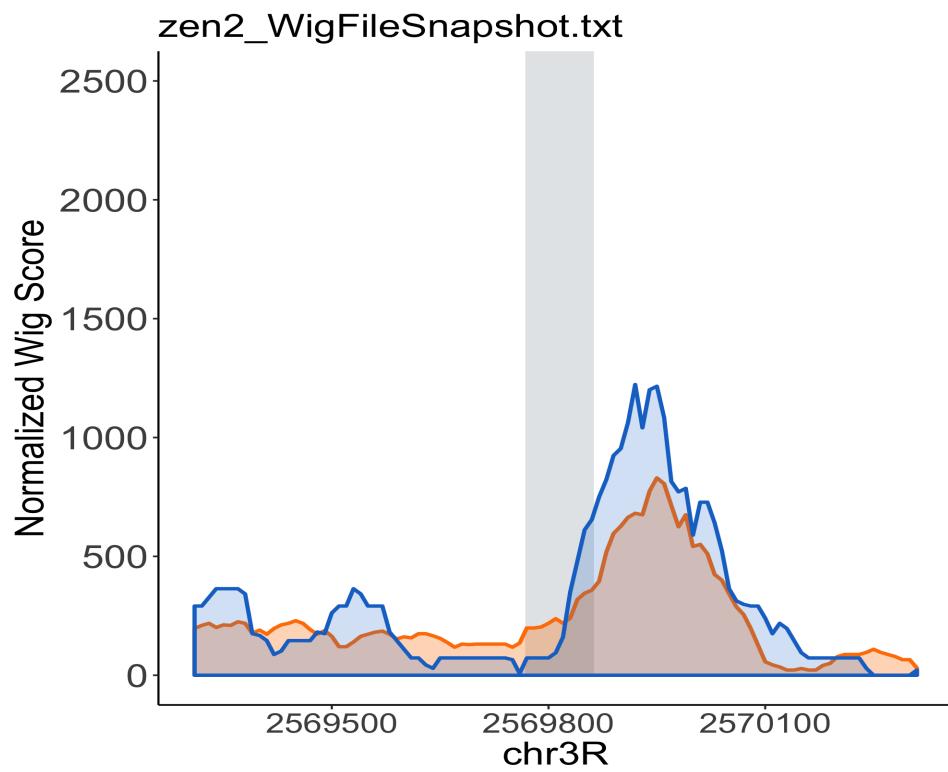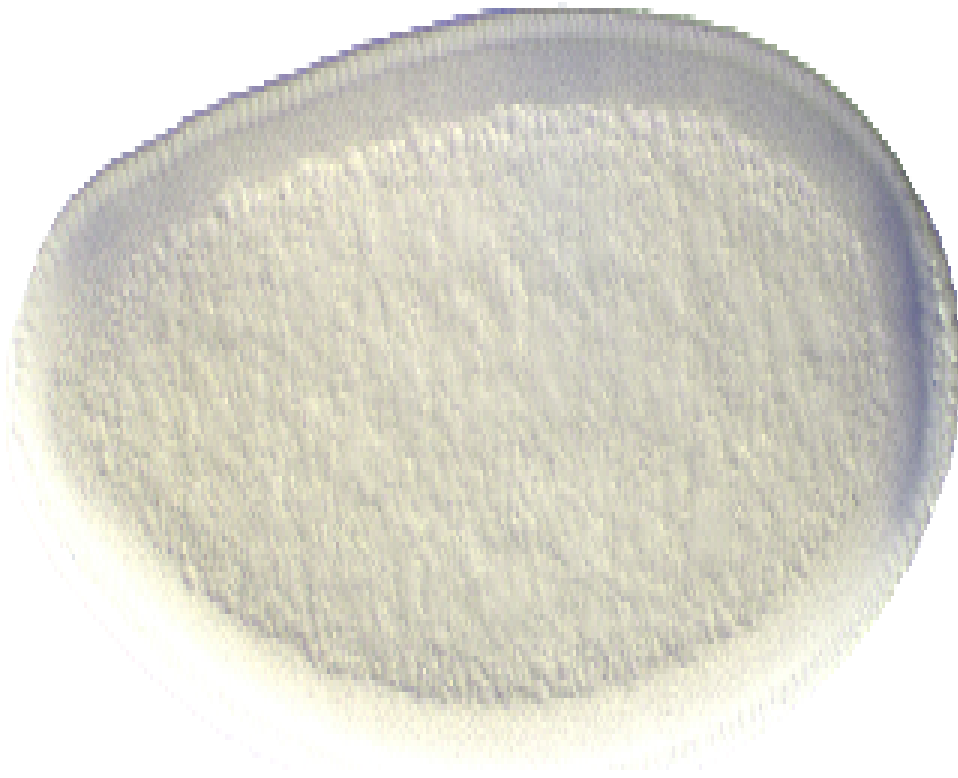

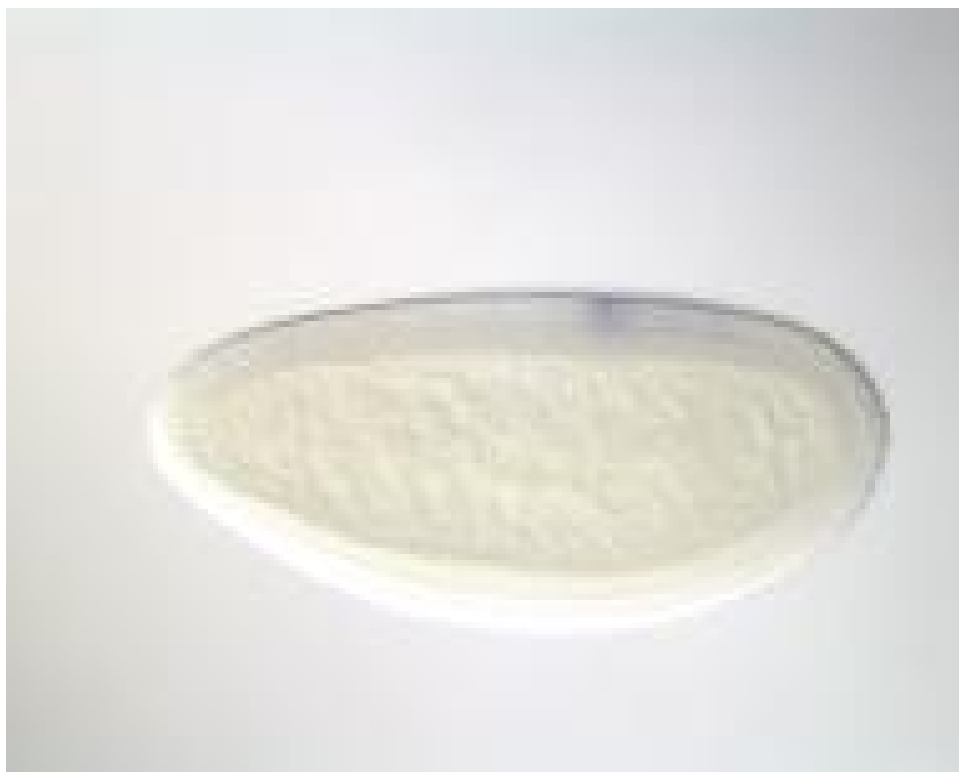

Location: Dorsal Type: Promoter ZScore: -0.024566742 PValue: 0.980400548

Supplement: S3 File — Reports consist of in situ hybridization images, ATAC-seq traces, and calculated p-value and Z Score for each region used in the final analysis. (ZIP) [file pgen.1007367.s015.zip › S3_File/zen2_Report.pdf]

## zen\_dist\_Doyle

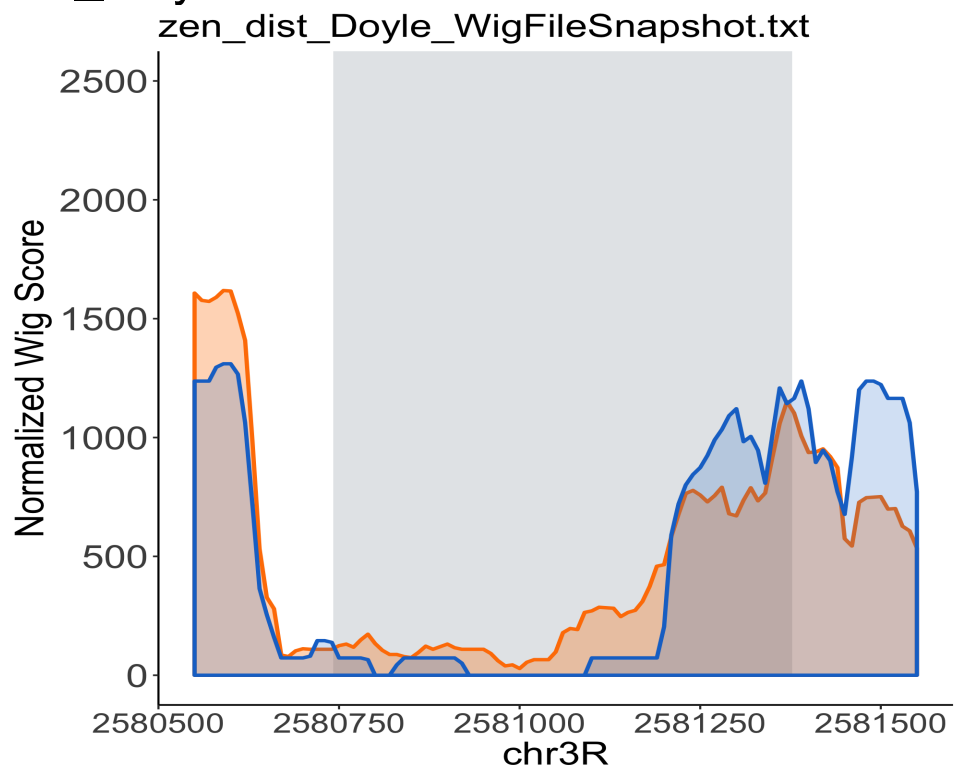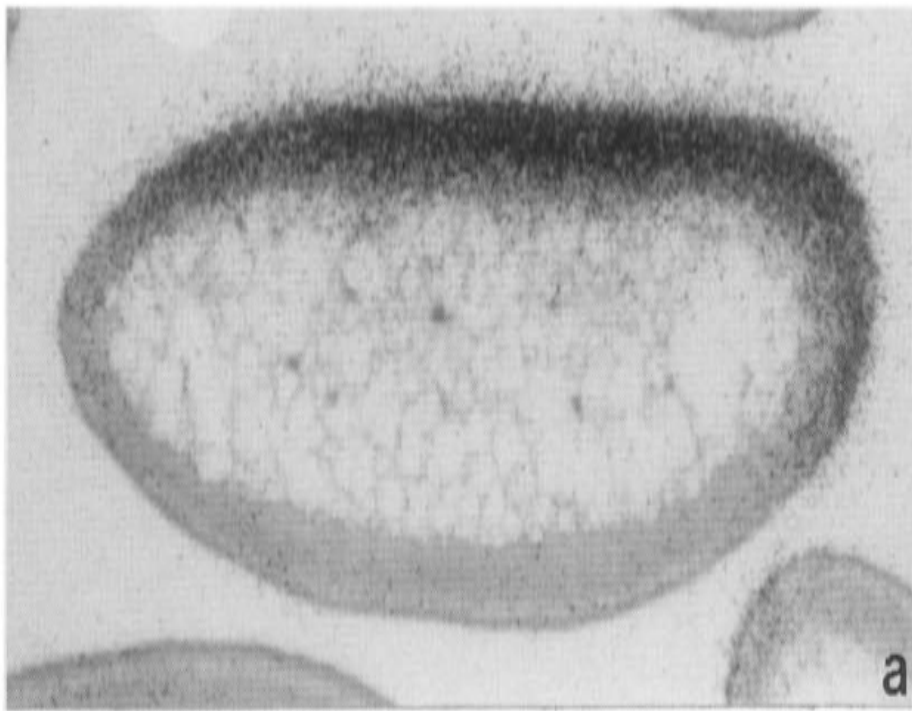

Location: Dorsal Type: Enhancer ZScore: 0.261276002 PValue: 0.793879672

Supplement: S3 File — Reports consist of in situ hybridization images, ATAC-seq traces, and calculated p-value and Z Score for each region used in the final analysis. (ZIP) [file pgen.1007367.s015.zip › S3_File/zen_dist_Doyle_Report.pdf]

## zen\_prox\_Doyle

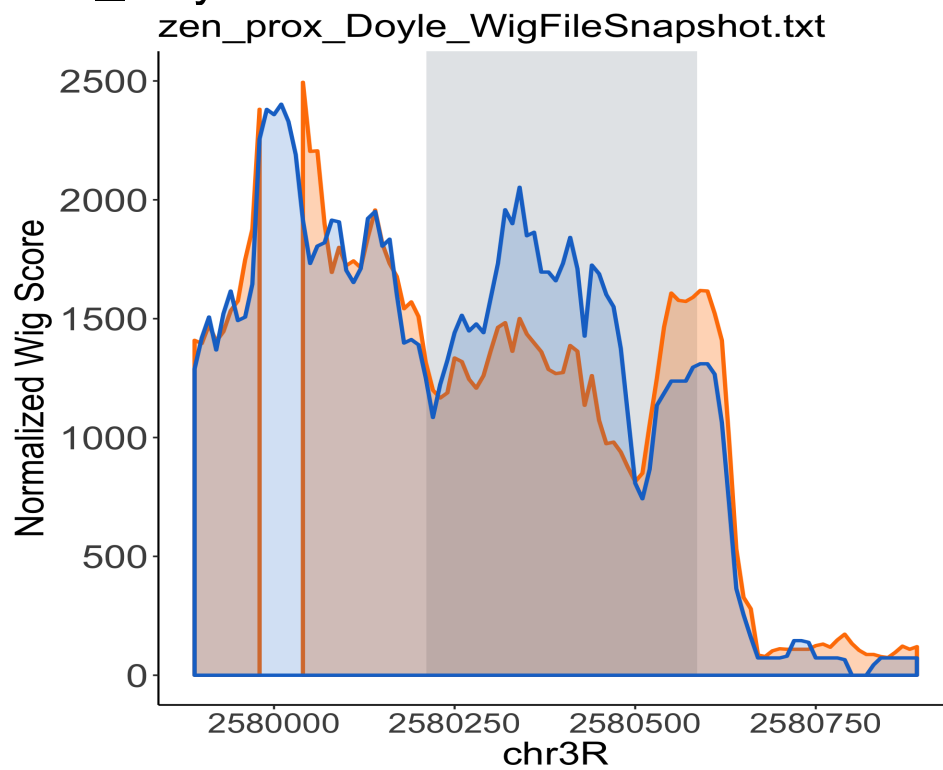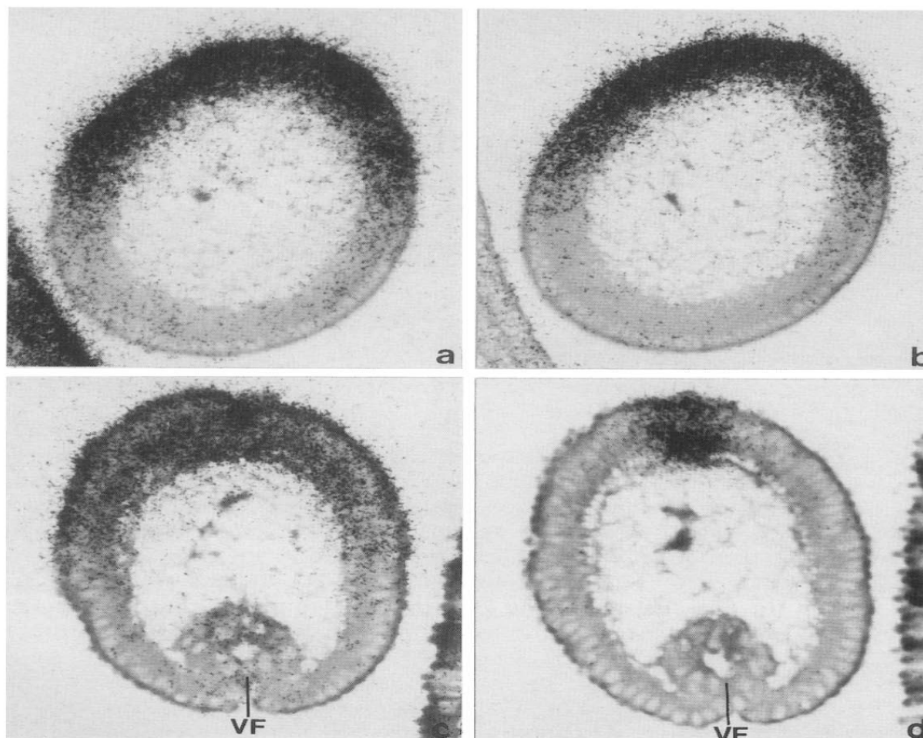

Location: Dorsal Type: Enhancer ZScore: -0.376172549 PValue: 0.706788622

Supplement: S3 File — Reports consist of in situ hybridization images, ATAC-seq traces, and calculated p-value and Z Score for each region used in the final analysis. (ZIP) [file pgen.1007367.s015.zip › S3_File/zen_prox_Doyle_Report.pdf]

zen

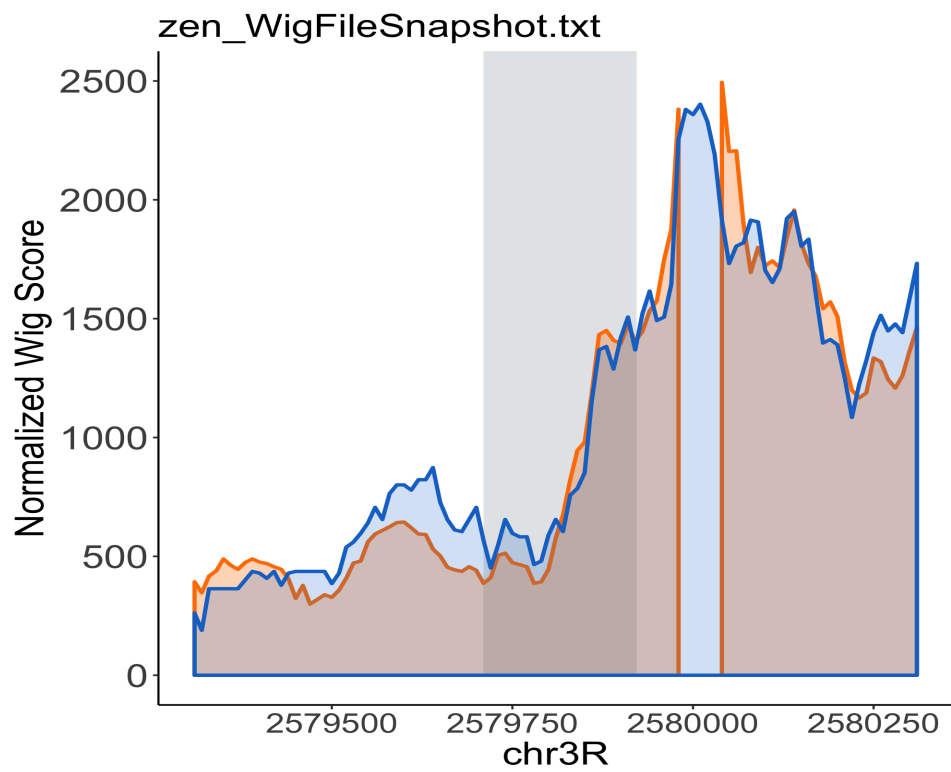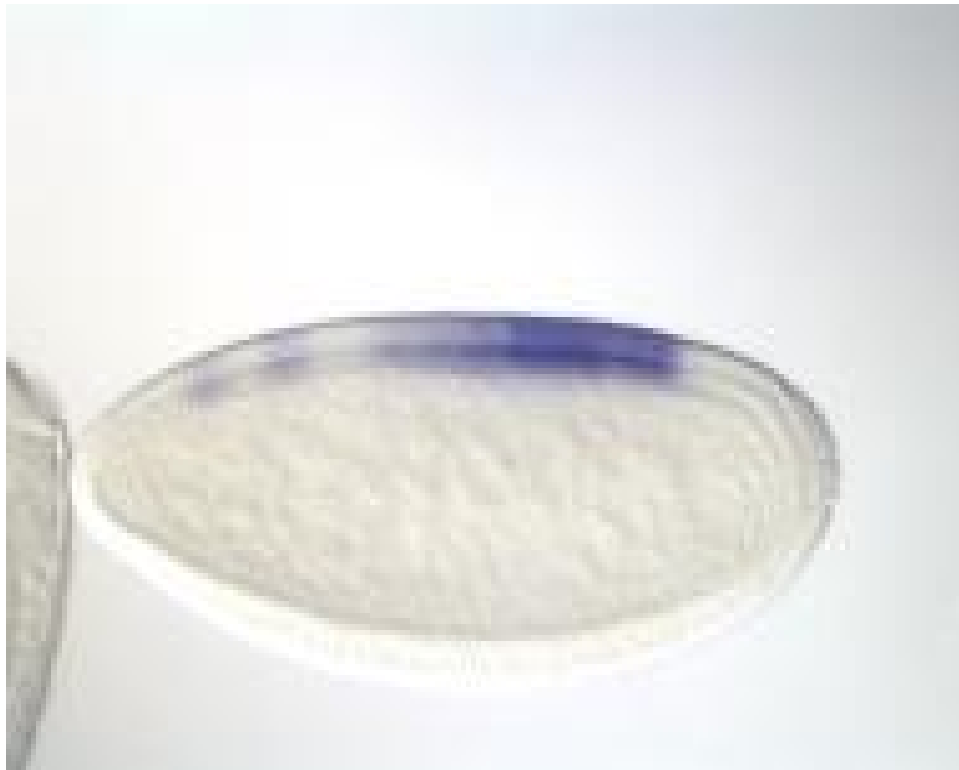

Location: Dorsal Type: Promoter ZScore: -0.103892418 PValue: 0.917254724

Supplement: S3 File — Reports consist of in situ hybridization images, ATAC-seq traces, and calculated p-value and Z Score for each region used in the final analysis. (ZIP) [file pgen.1007367.s015.zip › S3_File/zen_Report.pdf]

zfh1

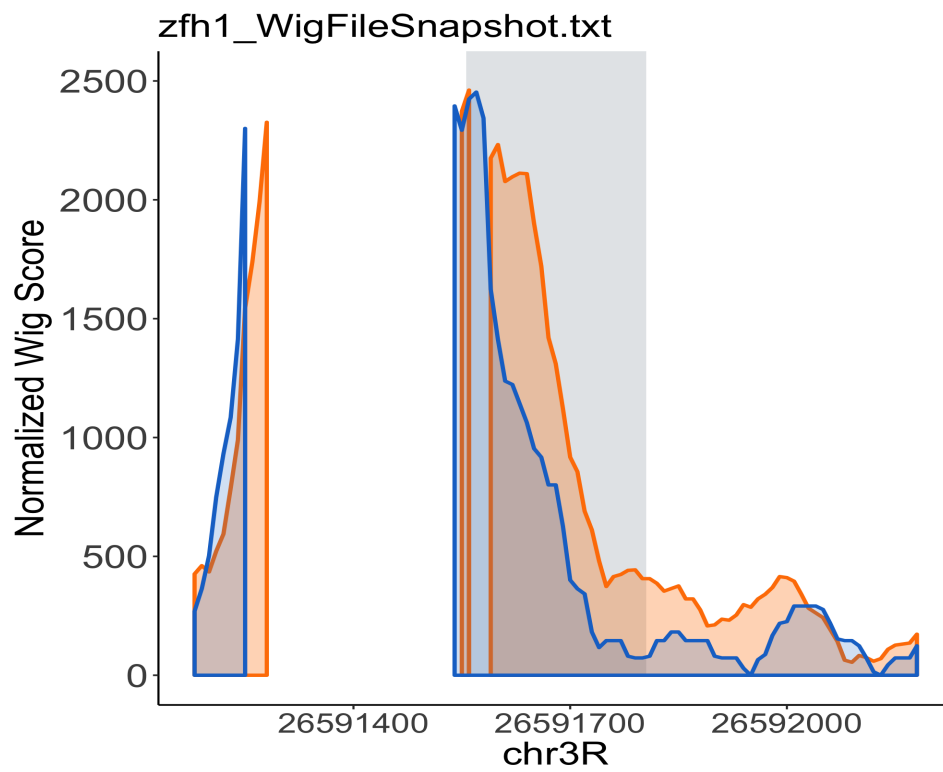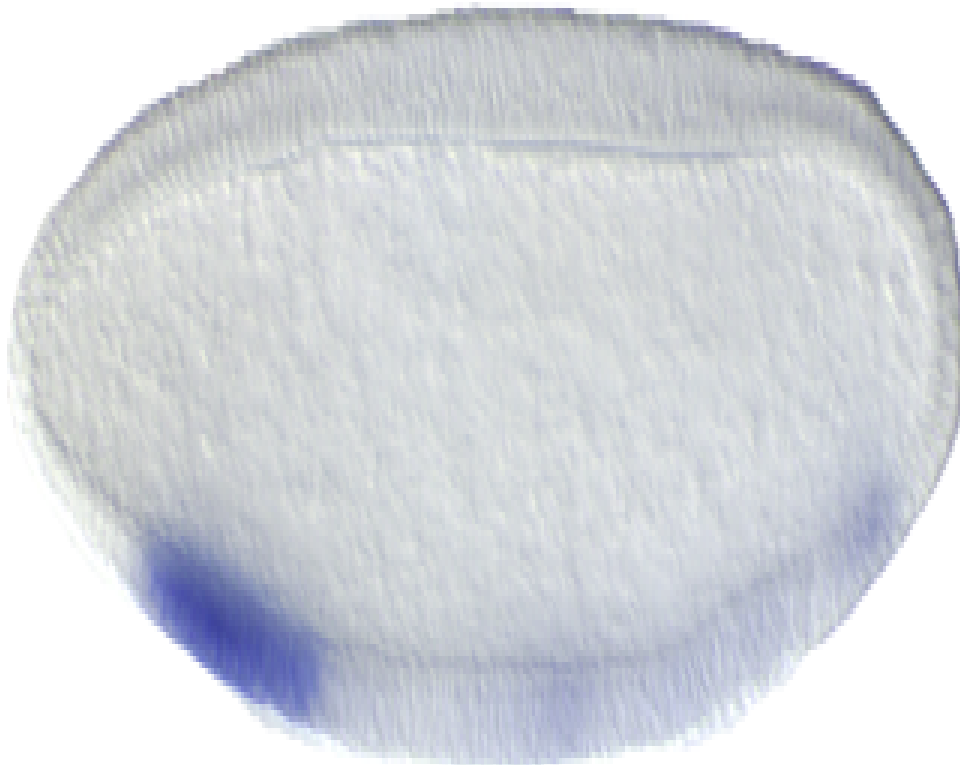

Location: Ventral Type: Promoter ZScore: 0.945050731 PValue: 0.344632977

Supplement: S3 File — Reports consist of in situ hybridization images, ATAC-seq traces, and calculated p-value and Z Score for each region used in the final analysis. (ZIP) [file pgen.1007367.s015.zip › S3_File/zfh1_Report.pdf]

zpg

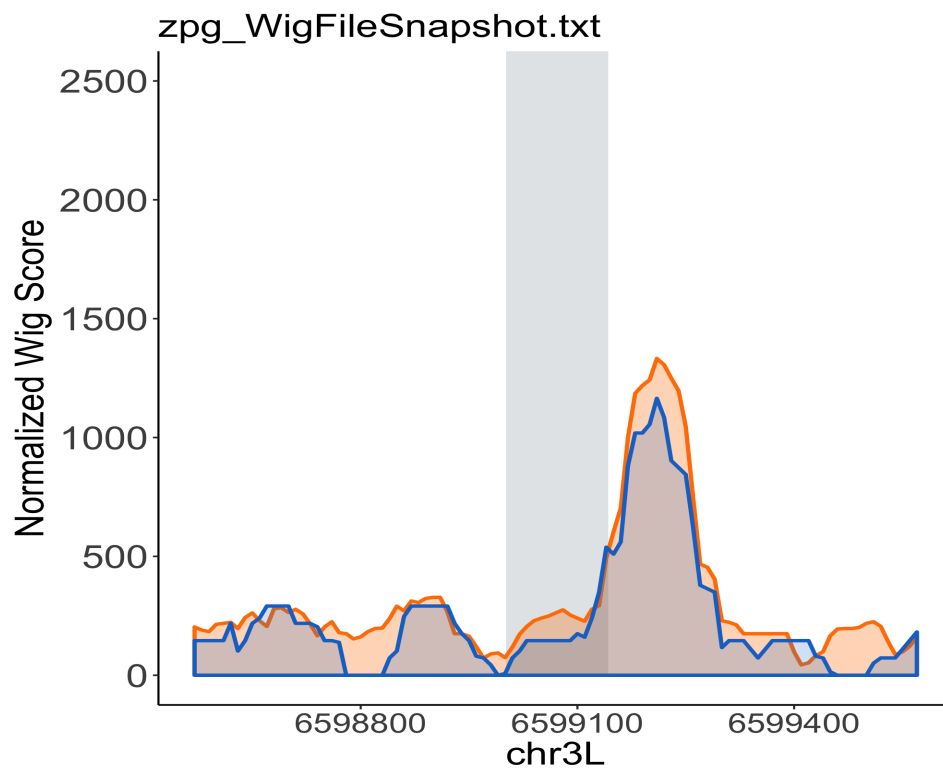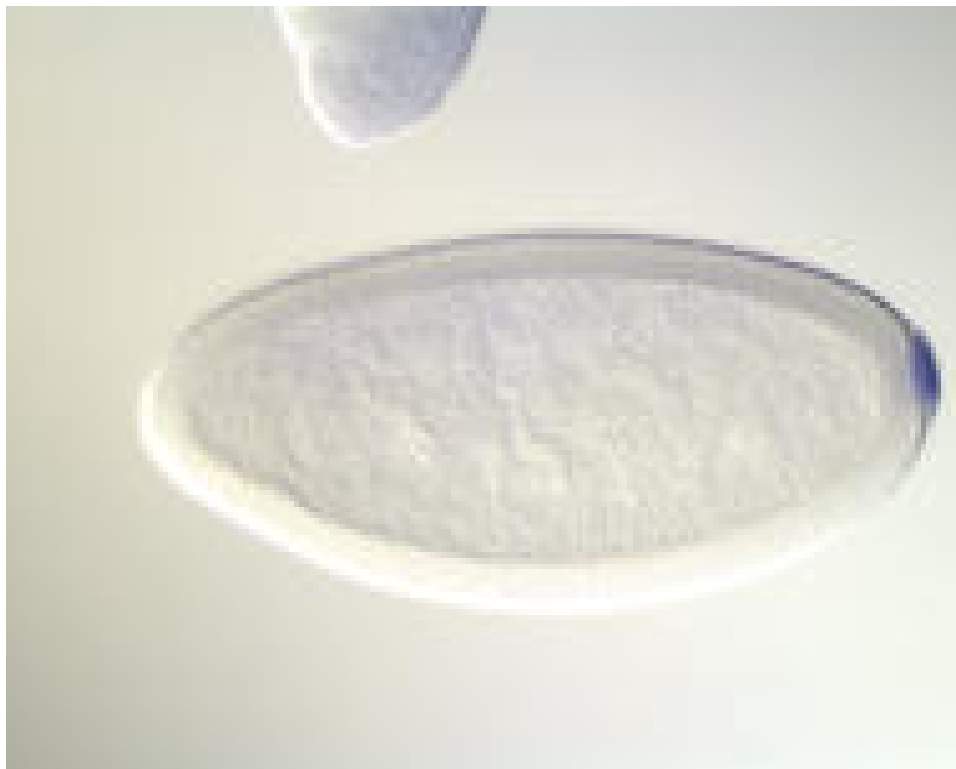

Location: Posterior Type: Promoter ZScore: -0.737618682 PValue: 0.4607462

Supplement: S3 File — Reports consist of in situ hybridization images, ATAC-seq traces, and calculated p-value and Z Score for each region used in the final analysis. (ZIP) [file pgen.1007367.s015.zip › S3_File/zpg_Report.pdf]
