# Supplementary material for: Patterns of chromatin accessibility along the anterior-posterior axis in the early Drosophila embryo
Source: PLoS Genet. 2018 May 4;14(5):e1007367. doi: 10.1371/journal.pgen.1007367 (PMC5955596; doi:10.1371/journal.pgen.1007367)
Supplement: S4 File — HTML file of all scripts used in the final analysis. (HTML) [file pgen.1007367.s016.html]

011718\_ATACHalves\_FinalCode\_Reviews


# Final Code/scripts used to Analyze data from ATAC Halves Paper¶

## Post revisions: 041118¶

### Main changes : Bamtobed used -bedpe parameter to keep reads in pairs, averaged wig signal over bed regions using custom script instead of deeptools for all figure generation, added global peak analysis for reviewers as well single halves analysis and replicate data.¶

### 04/18 - Added code used to analyze single cell ATAC-seq data from Cusanovich 2018.¶

https://www.nature.com/articles/nature25981

## Alignment¶

### 041217 Bowtie 2 with 1105-ATACSlice2 and 1109-ATACSlice3¶

In [ ]:

```
############################################################################

##########   041217_BowtieAlignment_MEJH112315_Bowtie2.sh                 ##########
##########   shell script to redo bowtie alignment - with Bowtie2         ##########

############################################################################
#! /bin/bash
bowtie2 -p 10 -5 5 -3 5 -N 1 -X 2000 --local --very-sensitive-local --un 041217Unmapped_ME_JH_112315_1105-ATACSlice2-01-20a_S10_L002_R1_001.fastq -x ~/Indexed_Genomes/dmel-r5.57-index -1 ME_JH_112315_1105-ATACSlice2-01-20a_S10_L002_R1_001.fastq -2 ME_JH_112315_1105-ATACSlice2-01-20a_S10_L002_R2_001.fastq -S 041217_Bowtie2_ME_JH_112315_1105-ATACSlice2-01-20a.sam
36984564 reads; of these:
  36984564 (100.00%) were paired; of these:
    11682987 (31.59%) aligned concordantly 0 times
    7150892 (19.33%) aligned concordantly exactly 1 time
    18150685 (49.08%) aligned concordantly >1 times
    ----
    11682987 pairs aligned concordantly 0 times; of these:
      3002509 (25.70%) aligned discordantly 1 time
    ----
    8680478 pairs aligned 0 times concordantly or discordantly; of these:
      17360956 mates make up the pairs; of these:
        5409439 (31.16%) aligned 0 times
        76837 (0.44%) aligned exactly 1 time
        11874680 (68.40%) aligned >1 times
92.69% overall alignment rate
bowtie2 -p 10 -5 5 -3 5 -N 1 -X 2000 --local --very-sensitive-local --un 041217Unmapped_ME_JH_112315_1105-ATACSlice2-02-20p_S11_L002_R1_001.fastq -x ~/Indexed_Genomes/dmel-r5.57-index -1 ME_JH_112315_1105-ATACSlice2-02-20p_S11_L002_R1_001.fastq -2 ME_JH_112315_1105-ATACSlice2-02-20p_S11_L002_R2_001.fastq -S 041217_Bowtie2_ME_JH_112315_1105-ATACSlice2-02-20p.sam
19383698 reads; of these:
  19383698 (100.00%) were paired; of these:
    6424010 (33.14%) aligned concordantly 0 times
    3825922 (19.74%) aligned concordantly exactly 1 time
    9133766 (47.12%) aligned concordantly >1 times
    ----
    6424010 pairs aligned concordantly 0 times; of these:
      1913417 (29.79%) aligned discordantly 1 time
    ----
    4510593 pairs aligned 0 times concordantly or discordantly; of these:
      9021186 mates make up the pairs; of these:
        1261391 (13.98%) aligned 0 times
        46631 (0.52%) aligned exactly 1 time
        7713164 (85.50%) aligned >1 times
96.75% overall alignment rate
bowtie2 -p 10 -5 5 -3 5 -N 1 -X 2000 --local --very-sensitive-local --un 041217Unmapped_ME_JH_112315_1105-ATACSlice2-5-1A_S12_L002_R1_001.fastq -x ~/Indexed_Genomes/dmel-r5.57-index -1 ME_JH_112315_1105-ATACSlice2-5-1A_S12_L002_R1_001.fastq -2 ME_JH_112315_1105-ATACSlice2-5-1A_S12_L002_R2_001.fastq -S 041217_Bowtie2_ME_JH_112315_1105-ATACSlice2-5-1A.sam
38641395 reads; of these:
  38641395 (100.00%) were paired; of these:
    8833286 (22.86%) aligned concordantly 0 times
    11791951 (30.52%) aligned concordantly exactly 1 time
    18016158 (46.62%) aligned concordantly >1 times
    ----
    8833286 pairs aligned concordantly 0 times; of these:
      2530398 (28.65%) aligned discordantly 1 time
    ----
    6302888 pairs aligned 0 times concordantly or discordantly; of these:
      12605776 mates make up the pairs; of these:
        6534429 (51.84%) aligned 0 times
        97541 (0.77%) aligned exactly 1 time
        5973806 (47.39%) aligned >1 times
91.54% overall alignment rate
bowtie2 -p 10 -5 5 -3 5 -N 1 -X 2000 --local --very-sensitive-local --un 041217Unmapped_ME_JH_112315_1105-ATACSlice2-6-1p_S13_L002_R1_001.fastq -x ~/Indexed_Genomes/dmel-r5.57-index -1 ME_JH_112315_1105-ATACSlice2-6-1p_S13_L002_R1_001.fastq -2 ME_JH_112315_1105-ATACSlice2-6-1p_S13_L002_R2_001.fastq -S 041217_Bowtie2_ME_JH_112315_1105-ATACSlice2-6-1p.sam
38796161 reads; of these:
  38796161 (100.00%) were paired; of these:
    15860018 (40.88%) aligned concordantly 0 times
    12329309 (31.78%) aligned concordantly exactly 1 time
    10606834 (27.34%) aligned concordantly >1 times
    ----
    15860018 pairs aligned concordantly 0 times; of these:
      3588306 (22.62%) aligned discordantly 1 time
    ----
    12271712 pairs aligned 0 times concordantly or discordantly; of these:
      24543424 mates make up the pairs; of these:
        21090425 (85.93%) aligned 0 times
        109906 (0.45%) aligned exactly 1 time
        3343093 (13.62%) aligned >1 times
72.82% overall alignment rate
bowtie2 -p 10 -5 5 -3 5 -N 1 -X 2000 --local --very-sensitive-local --un 041217Unmapped_ME_JH_112315_1105-ATACSlice2-7-10whole_S14_L002_R1_001.fastq -x ~/Indexed_Genomes/dmel-r5.57-index -1 ME_JH_112315_1105-ATACSlice2-7-10whole_S14_L002_R1_001.fastq -2 ME_JH_112315_1105-ATACSlice2-7-10whole_S14_L002_R2_001.fastq -S 041217_Bowtie2_ME_JH_112315_1105-ATACSlice2-7-10whole.sam
47244276 reads; of these:
  47244276 (100.00%) were paired; of these:
    11029803 (23.35%) aligned concordantly 0 times
    12744976 (26.98%) aligned concordantly exactly 1 time
    23469497 (49.68%) aligned concordantly >1 times
    ----
    11029803 pairs aligned concordantly 0 times; of these:
      3886775 (35.24%) aligned discordantly 1 time
    ----
    7143028 pairs aligned 0 times concordantly or discordantly; of these:
      14286056 mates make up the pairs; of these:
        2193818 (15.36%) aligned 0 times
        105007 (0.74%) aligned exactly 1 time
        11987231 (83.91%) aligned >1 times
97.68% overall alignment rate
bowtie2 -p 10 -5 5 -3 5 -N 1 -X 2000 --local --very-sensitive-local --un 041217Unmapped_ME_JH_112315_1109-ATACSlice03-01-20Ant_S6_L002_R1_001.fastq -x ~/Indexed_Genomes/dmel-r5.57-index -1 ME_JH_112315_1109-ATACSlice03-01-20Ant_S6_L002_R1_001.fastq -2 ME_JH_112315_1109-ATACSlice03-01-20Ant_S6_L002_R2_001.fastq -S 041217_Bowtie2_ME_JH_112315_1109-ATACSlice03-01-20Ant.sam
50087614 reads; of these:
  50087614 (100.00%) were paired; of these:
    16323533 (32.59%) aligned concordantly 0 times
    16922406 (33.79%) aligned concordantly exactly 1 time
    16841675 (33.62%) aligned concordantly >1 times
    ----
    16323533 pairs aligned concordantly 0 times; of these:
      8829610 (54.09%) aligned discordantly 1 time
    ----
    7493923 pairs aligned 0 times concordantly or discordantly; of these:
      14987846 mates make up the pairs; of these:
        5010642 (33.43%) aligned 0 times
        164006 (1.09%) aligned exactly 1 time
        9813198 (65.47%) aligned >1 times
95.00% overall alignment rate
bowtie2 -p 10 -5 5 -3 5 -N 1 -X 2000 --local --very-sensitive-local --un 041217Unmapped_ME_JH_112315_1109-ATACSlice03-02-20Post_S7_L002_R1_001.fastq -x ~/Indexed_Genomes/dmel-r5.57-index -1 ME_JH_112315_1109-ATACSlice03-02-20Post_S7_L002_R1_001.fastq -2 ME_JH_112315_1109-ATACSlice03-02-20Post_S7_L002_R2_001.fastq -S 041217_Bowtie2_ME_JH_112315_1109-ATACSlice03-02-20Post.sam
34457240 reads; of these:
  34457240 (100.00%) were paired; of these:
    25556167 (74.17%) aligned concordantly 0 times
    4056100 (11.77%) aligned concordantly exactly 1 time
    4844973 (14.06%) aligned concordantly >1 times
    ----
    25556167 pairs aligned concordantly 0 times; of these:
      2286019 (8.95%) aligned discordantly 1 time
    ----
    23270148 pairs aligned 0 times concordantly or discordantly; of these:
      46540296 mates make up the pairs; of these:
        42621281 (91.58%) aligned 0 times
        582488 (1.25%) aligned exactly 1 time
        3336527 (7.17%) aligned >1 times
38.15% overall alignment rate
bowtie2 -p 10 -5 5 -3 5 -N 1 -X 2000 --local --very-sensitive-local --un 041217Unmapped_ME_JH_112315_1109-ATACSlice03-03-1plus2_S8_L002_R1_001.fastq -x ~/Indexed_Genomes/dmel-r5.57-index -1 ME_JH_112315_1109-ATACSlice03-03-1plus2_S8_L002_R1_001.fastq -2 ME_JH_112315_1109-ATACSlice03-03-1plus2_S8_L002_R2_001.fastq -S 041217_Bowtie2_ME_JH_112315_1109-ATACSlice03-03-1plus2.sam
42215974 reads; of these:
  42215974 (100.00%) were paired; of these:
    25607270 (60.66%) aligned concordantly 0 times
    7688333 (18.21%) aligned concordantly exactly 1 time
    8920371 (21.13%) aligned concordantly >1 times
    ----
    25607270 pairs aligned concordantly 0 times; of these:
      3471143 (13.56%) aligned discordantly 1 time
    ----
    22136127 pairs aligned 0 times concordantly or discordantly; of these:
      44272254 mates make up the pairs; of these:
        38305732 (86.52%) aligned 0 times
        567172 (1.28%) aligned exactly 1 time
        5399350 (12.20%) aligned >1 times
54.63% overall alignment rate
bowtie2 -p 10 -5 5 -3 5 -N 1 -X 2000 --local --very-sensitive-local --un 041217Unmapped_ME_JH_112315_1109-ATACSlice03-04-10whole_S9_L002_R1_001.fastq -x ~/Indexed_Genomes/dmel-r5.57-index -1 ME_JH_112315_1109-ATACSlice03-04-10whole_S9_L002_R1_001.fastq -2 ME_JH_112315_1109-ATACSlice03-04-10whole_S9_L002_R2_001.fastq -S 041217_Bowtie2_ME_JH_112315_1109-ATACSlice03-04-10whole.sam
43706348 reads; of these:
  43706348 (100.00%) were paired; of these:
    11553413 (26.43%) aligned concordantly 0 times
    14554768 (33.30%) aligned concordantly exactly 1 time
    17598167 (40.26%) aligned concordantly >1 times
    ----
    11553413 pairs aligned concordantly 0 times; of these:
      5625119 (48.69%) aligned discordantly 1 time
    ----
    5928294 pairs aligned 0 times concordantly or discordantly; of these:
      11856588 mates make up the pairs; of these:
        2890921 (24.38%) aligned 0 times
        146097 (1.23%) aligned exactly 1 time
        8819570 (74.39%) aligned >1 times
96.69% overall alignment rate
```

## Filtering and conversion to Wig Files¶

### Then I converted sam files to paired end bed files (quality control above 30), shifted them, converted to wig files, then normalized each wig to 1 million reads.¶

1) Sam file generated from above alignment
2) Sam to Bam --> sam file filtered out

```
> -F 1804
    - unmapped
    - not primary alignment
    - reads fails platform/vendor quality checks
    - read is PCR or optical duplicate
```

> -f 2
>
> ```
>    - kept only reads mapped in proper pair
> ```
>
> - MAPQ score greater than 30

3) Picard remove duplicates : REMOVE\_DUPLICATES = TRUE
4) Bam to bed

```
- bedtools bamtobed with -BEDPE parameter that outputs both read pairs on a single line
```

5) Bed shift -> take only the start of read one and the end of read 2 and shift the start + 4 -1 for the count and then the end - 5 - 1 or -6 as in Buenrostro 2013 (https://www.nature.com/articles/nmeth.2688). Then filtered out everything less than or equal to 130bp. Keep the bed for merging and peak calling.
6) Bed to Wig with XY custom script (see below) xl-bed2wig-dirproc-HOA-2014-new-chrsz
7) Count number of lines to estimate reads in each file and normalize each file to 1 million reads (merged files below will be normalized roughtly to 10 million reads) by finding wig signal \* (1E6/read number)

In [ ]:

```
################################################################################################
##########   011618_postalign_RemoveDuplicates_SortedBam_PairedEnd_Sam_FilterFragments.sh ##########
##########   Input:  sam file                                                          ##########
#########    Output: DupRemovedBam file                                               ##########
#########    ATAC shifted, 130 and below bed file, wig file, 10Mnormalized wig file   ##########
#########                                                                             ##########
################################################################################################

#bash 011618_postalign_RemoveDuplicates_SortedBam_PairedEnd_Sam_FilterFragments.sh 2> 011618_postalign_RemoveDuplicates_SortedBam_PairedEnd_Sam_FilterFragments_STERR.txt | cat > 011618_postalign_RemoveDuplicates_SortedBam_PairedEnd_Sam_FilterFragments_STDOUT.txt &

#! /bin/bash


# original sam file
Bowtie2SamFile[1]=041217_Bowtie2_ME_JH_112315_1105-ATACSlice2-01-20a.sam
Bowtie2SamFile[2]=041217_Bowtie2_ME_JH_112315_1105-ATACSlice2-02-20p.sam
Bowtie2SamFile[3]=041217_Bowtie2_ME_JH_112315_1105-ATACSlice2-5-1A.sam
Bowtie2SamFile[4]=041217_Bowtie2_ME_JH_112315_1105-ATACSlice2-6-1p.sam
Bowtie2SamFile[5]=041217_Bowtie2_ME_JH_112315_1105-ATACSlice2-7-10whole.sam
Bowtie2SamFile[6]=041217_Bowtie2_ME_JH_112315_1109-ATACSlice03-01-20Ant.sam
Bowtie2SamFile[7]=041217_Bowtie2_ME_JH_112315_1109-ATACSlice03-02-20Post.sam
Bowtie2SamFile[8]=041217_Bowtie2_ME_JH_112315_1109-ATACSlice03-03-1plus2.sam
Bowtie2SamFile[9]=041217_Bowtie2_ME_JH_112315_1109-ATACSlice03-04-10whole.sam

#Core name that will be carried with the sample
filesetname[1]=101117_RemDUP_PE_041217_Bowtie2_ME_JH_112315_1105-ATACSlice2-01-20a 
filesetname[2]=081817_RemDUP_041217_Bowtie2_ME_JH_112315_1105-ATACSlice2-02-20p
filesetname[3]=081817_RemDUP_041217_Bowtie2_ME_JH_112315_1105-ATACSlice2-5-1A   
filesetname[4]=081817_RemDUP_041217_Bowtie2_ME_JH_112315_1105-ATACSlice2-6-1p
filesetname[5]=081817_RemDUP_041217_Bowtie2_ME_JH_112315_1105-ATACSlice2-7-10whole         
filesetname[6]=081817_RemDUP_041217_Bowtie2_ME_JH_112315_1109-ATACSlice03-01-20Ant
filesetname[7]=081817_RemDUP_041217_Bowtie2_ME_JH_112315_1109-ATACSlice03-02-20Post 
filesetname[8]=081817_RemDUP_041217_Bowtie2_ME_JH_112315_1109-ATACSlice03-03-1plus2
filesetname[9]=081817_RemDUP_041217_Bowtie2_ME_JH_112315_1109-ATACSlice03-04-10whole

for k in 1 2 3 4 5 6 7 8 9
do
    samfile=${Bowtie2SamFile[${k}]}
    echo 'sam file imported'
    filesetname=${filesetname[${k}]}
    samtools view -bS -F 1804 -f 2 -q 30 ${samfile} | samtools sort - ${filesetname}
    java -jar ~/scripts/picard.jar MarkDuplicates REMOVE_DUPLICATES=TRUE \
      I=${filesetname}.bam \
      O=${filesetname}_nodups.bam \
      M=${filesetname}_marked_dup_metrics.txt
    echo 'Duplicates removed'
    samtools sort -n ${filesetname}_nodups.bam ${filesetname}.srt.temp 
    bedtools bamtobed -bedpe -i ${filesetname}.srt.temp.bam | perl -n -e '@A = split (/\t/, $_); $start = $A[1] +4 ; $end = $A[5] - 6 ; print "$A[0]\t$start\t$end\t$A[6]\t$A[7]\t$A[8]\t$[9]\n"' > 011617_${filesetname}_shifted.bed
    echo 'shifted bed PE file made'
    perl -n -e '@A = split (/\t/, $_); $readsize = abs($A[2] - $A[1]); if ($readsize <= 130) {print $_}' 011617_${filesetname}_shifted.bed > 011617_${filesetname}_shifted_lessthan130.bed
    echo 'filtered based on 130 bp threshold'
   
done
echo 'making wig files'
mkdir Bedfiles_011617
mv *.bed Bedfiles_011617
perl ~/scripts/xl-bed2wig-dirproc-HOA-2014-new-chrsz Bedfiles_011617

#This script will take the filtered bed files.. count the number of reads and then use that to noramlize the wig files

for k in 1 2 3 4 5 6 7 8 9
do
  cd Bedfiles_011617
  filesetname=${filesetname[${k}]}
  NumberofReads=$(wc -l < "011617_${filesetname}_shifted_lessthan130.bed")
  echo "$NumberofReads"
  export NumberofReads
  echo "Normalizing to 1M reads"
  perl -n -e ' $Scale = 1000000/$ENV{NumberofReads} ; if (/^\d+/) {@A = split (/\s+/, $_); $norm = $A[1] * $Scale ; print "$A[0]\t $norm \n"} else { print "$_"}' 011617_${filesetname}_shifted_lessthan130.wig > 011617_${filesetname}_shifted_lessthan130_1MNorm.wig
  echo "${filesetname} Normalized wig File made"

done
```

### Merged Replicates¶

In [ ]:

```
cat 011617_081817_RemDUP_041217_Bowtie2_ME_JH_112315_1105-ATACSlice2-02-20p_shifted_lessthan130.bed 011617_081817_RemDUP_041217_Bowtie2_ME_JH_112315_1109-ATACSlice03-02-20Post_shifted_lessthan130.bed > 011718_MERGED_2reps_081817_RemDUP_041217_Bowtie2_ME_JH_112315_20Post_shifted_lessthan130.bed &
cat 011617_081817_RemDUP_041217_Bowtie2_ME_JH_112315_1109-ATACSlice03-01-20Ant_shifted_lessthan130.bed 011617_101117_RemDUP_PE_041217_Bowtie2_ME_JH_112315_1105-ATACSlice2-01-20a_shifted_lessthan130.bed > 011718_MERGED_2reps_081817_RemDUP_041217_Bowtie2_ME_JH_112315_20Ant_shifted_lessthan130.bed &
cat 011617_081817_RemDUP_041217_Bowtie2_ME_JH_112315_1105-ATACSlice2-7-10whole_shifted_lessthan130.bed 011617_081817_RemDUP_041217_Bowtie2_ME_JH_112315_1109-ATACSlice03-04-10whole_shifted_lessthan130.bed > 011718_MERGED_2reps_081817_RemDUP_041217_Bowtie2_ME_JH_112315_10whole_shifted_lessthan130.bed &

#Then made into wig files with script below
perl ~/scripts/xl-bed2wig-dirproc-HOA-2014-new-chrsz 011718_mergedfiles
```

In [ ]:

```
##########################################################################################
##########   ~/scripts/xl-bed2wig-dirproc-HOA-2014-new-chrsz                    ##########
##########   Input: Bed files                                                   ##########
#########    Output: Wig files                                                  ##########
##########################################################################################


#!/usr/bin/perl

use warnings;

my $dir = shift;

opendir DIR, $dir or die "can't open $dir: $!";
while ( my $file = readdir DIR ) {
  if ($file =~ /^(.+).(BED|bed|bedgraph)/) {
    $In_file = "$dir/$file";
    print "infile:$In_file\n";
    my $output ="$dir/$1.wig";
    print "output:$output\n";
    my $wigname = "$1";
    my %HOA = ();
    my %chr_sz=(
                "chr2L" => 23011344, #new size info, aug 2014
                "chr2R" => 21146608,
                "chr3L" => 24543457,
                "chr3R" => 27905053,
                "chr4" => 1351757,
                "chrX" => 22332727 );

    foreach $chr (keys %chr_sz){
      $n = int($chr_sz{$chr}/10);
      @a = (0)x$n;
      $HOA{$chr} = [@a];
    }

    my ($chr2, $stp, $endp);
    open (INPUT, '<', $In_file);
    while (<INPUT>) {
      next if (/(tr|U|Het|dmel)/);
      my @spl = split /\s+/, $_;
      # $chr2 = $spl[0];
      # $stp = $spl[1]; 
      # $endp = $spl[2];
      my @n;
      my $chp;
      $chr_n = $spl[0];
      $chr_n =~ tr/chr//d;
      $chr2 = "chr$chr_n";
        
      if ($spl[2] < $chr_sz{$chr2}) {
        foreach $n($spl[1] .. $spl[2]) {                      
          $chp = int($n/10);
          $HOA{$chr2}[$chp] +=1;
        }
      }
    }                           
                        
    close INPUT;

    open (OUTPUT, '>', $output);
    print OUTPUT "track type=wiggle_0 name=\"$wigname\" description=\"$wigname\"visibility=full autoScale=off maxHeightPixels=100:50:20\n";
    foreach $key (keys %HOA){
      print OUTPUT "variableStep chrom=$key span=10\n";
      foreach $i (0 .. $#{$HOA{$key}}) {
        my $m = ($i+1)*10;
        my $v = $HOA{$key}[$i];
        if ($v > 0){
          print OUTPUT "$m\t$v\n";
                }
      }
    }  
    close OUTPUT;
  }
}
closedir(DIR);
```

#### Normalized merged files to 10 million reads¶

In [ ]:

```
################################################################################################
##########   011718_10Mnormalization_mergedfiles.sh                                   ##########
##########   Input:  merged bed files  + merged wig files                             ##########
#########    Output: merged wig files normalized to 10M reads                         ##########
################################################################################################

#bash 011718_10Mnormalization_mergedfiles.sh 2> 011718_10Mnormalization_mergedfiles_STERR.txt | cat > 011718_10Mnormalization_mergedfiles_STDOUT.txt &

#! /bin/bash


filesetname[1]=011718_MERGED_2reps_081817_RemDUP_041217_Bowtie2_ME_JH_112315_10whole
filesetname[2]=011718_MERGED_2reps_081817_RemDUP_041217_Bowtie2_ME_JH_112315_20Ant
filesetname[3]=011718_MERGED_2reps_081817_RemDUP_041217_Bowtie2_ME_JH_112315_20Post

for k in 1 2 3 
do
  cd 011718_mergedfiles
  filesetname=${filesetname[${k}]}
  NumberofReads=$(wc -l < "${filesetname}_shifted_lessthan130.bed")
  echo "$NumberofReads"
  export NumberofReads
  echo "Normalizing to 10M reads"
  perl -n -e ' $Scale = 10000000/$ENV{NumberofReads} ; if (/^\d+/) {@A = split (/\s+/, $_); $norm = $A[1] * $Scale ; print "$A[0]\t $norm \n"} else { print "$_"}' ${filesetname}_shifted_lessthan130.wig > ${filesetname}_shifted_lessthan130_10MNorm.wig
  echo "${filesetname} Normalized wig File made"

done
```

### Output from above script (011718\_10Mnormalization\_mergedfiles.sh):¶

6331889 011718\_MERGED\_2reps\_081817\_RemDUP\_041217\_Bowtie2\_ME\_JH\_112315\_10whole Normalized wig File made
Normalizing to 10M reads

7911835 011718\_MERGED\_2reps\_081817\_RemDUP\_041217\_Bowtie2\_ME\_JH\_112315\_20Ant Normalized wig File made
Normalizing to 10M reads

2265938 011718\_MERGED\_2reps\_081817\_RemDUP\_041217\_Bowtie2\_ME\_JH\_112315\_20Post Normalized wig File made
Normalizing to 10M reads

### Converted wigs to bigwigs using wigToBigWig from UCSC¶

# Normalization¶

#### First Normalized to 10 million mapped Dmel Reads based on the total amount of lines in the combined bedfiles (above script)¶

#### Then normalized to Anterior, Posterior, and AplusP merged files to the linear regression model against the Whole sample ignoring the y intercept¶

- The below R script was used to generate the values used to normalize the wig files

In [ ]:

```
############### ################ ################ ################ ################
################ 011818_RefinedGeneList_Dataanalysis_linregv2_PEReviews.r     ################ 
################ make scatterplots with ATAC Score and position               ################ 
################calculated in 051117_randomdistribution_script                ################
################ ################ ################ ################ ################


library("plyr")
library("dplyr")
library("ggplot2")


############################ Linear Regression Normalization ############################ 

###### ### ###  Working directory
setwd("~/011618_PairedEndAnalysisforReviews/")

###### ### ###  Input files

# Opens the entire merged wig file made in bedtools that binned every 1Kb
# first input file is 011918_multiBigWigCompare_1KB_041417_Bowtie2_ATACPools_merged.txt
# multiBigwigSummary bins -bs 1000 -b \
# /Users/jennahaines/'Box Sync'/Eisen_Lab/Experiments/ATAC-seq/Halves/ATAC-seq_Pools/040617_Analysis/041717_NormalizedWigs/bigwig/011718_MERGED_2reps_081817_RemDUP_041217_Bowtie2_ME_JH_112315_10whole_shifted_lessthan130_10MNorm.bw \
# /Users/jennahaines/'Box Sync'/Eisen_Lab/Experiments/ATAC-seq/Halves/ATAC-seq_Pools/040617_Analysis/041717_NormalizedWigs/bigwig/011718_MERGED_2reps_081817_RemDUP_041217_Bowtie2_ME_JH_112315_20Ant_shifted_lessthan130_10MNorm.bw \
# /Users/jennahaines/'Box Sync'/Eisen_Lab/Experiments/ATAC-seq/Halves/ATAC-seq_Pools/040617_Analysis/041717_NormalizedWigs/bigwig/011718_MERGED_2reps_081817_RemDUP_041217_Bowtie2_ME_JH_112315_20Post_shifted_lessthan130_10MNorm.bw \
# /Users/jennahaines/'Box Sync'/Eisen_Lab/Experiments/ATAC-seq/Halves/ATAC-seq_Pools/040617_Analysis/041717_NormalizedWigs/bigwig/011818_100417_BDTNP_DNaseI_stage5_ALLREPSCOMBINED_shifted_10MNorm.bw \
# -out 011918_multiBigWigCompare_1KBbins_041417_Bowtie2_ATACPools_merged.npz --outRawCounts 011918_multiBigWigCompare_1KB_041417_Bowtie2_ATACPools_merged.txt

merged_norm_1kb = read.delim2("011918_multiBigWigCompare_1KB_041417_Bowtie2_ATACPools_merged.txt", sep = "\t", stringsAsFactors = FALSE) 

colnames(merged_norm_1kb)  = c("chr", "start", "end", "Whole", "Ant", "Post", "DnaseI")

allSamples_NoNAs = merged_norm_1kb %>%
  select(chr, start, end, Whole, Ant, Post, DnaseI) %>%
  filter(Whole != 'nan') %>%
  filter(Ant != 'nan') %>%
  filter(Post != 'nan') %>%
  filter(DnaseI != 'nan') %>%
  mutate(Type = "NA") %>%
  mutate(Location = "NA") %>%
  mutate(Name = "NA") %>%
  mutate(Dotsize = 1)

### calculate linear regression model
merged_norm_1kb_AntvsWhole.lm = lm(as.numeric(allSamples_NoNAs$Ant) ~ as.numeric(allSamples_NoNAs$Whole))
merged_norm_1kb_PostvsWhole.lm = lm(as.numeric(allSamples_NoNAs$Post) ~ as.numeric(allSamples_NoNAs$Whole))

###### ### ###  Ant vs Whole
# Call:
#   lm(formula = as.numeric(allSamples_NoNAs$Ant) ~ as.numeric(allSamples_NoNAs$Whole))
# 
# Coefficients:
#   (Intercept)  
# 36.0167  
# as.numeric(allSamples_NoNAs$Whole)  
# 0.5787

###### ### ###  Post vs Whole
# Call:
#   lm(formula = as.numeric(allSamples_NoNAs$Post) ~ as.numeric(allSamples_NoNAs$Whole))
# 
# Coefficients:
#   (Intercept)  
# 50.0868  
# as.numeric(allSamples_NoNAs$Whole)  
# 0.6063 

#### Correlations 
AP_NoNormCor = cor(as.numeric(allSamples_NoNAs$Ant),as.numeric(allSamples_NoNAs$Post), method = "spearman")
AP_NoNormCor_P = cor(as.numeric(allSamples_NoNAs$Ant),as.numeric(allSamples_NoNAs$Post), method = "pearson")
AP_NoNormCor_P_sq = (AP_NoNormCor_P)^2
AP_NoNormCor_sq = (AP_NoNormCor)^2

AW_NoNormCor = cor(as.numeric(allSamples_NoNAs$Ant),as.numeric(allSamples_NoNAs$Whole), method = "spearman")
AW_NoNormCor_P = cor(as.numeric(allSamples_NoNAs$Ant),as.numeric(allSamples_NoNAs$Whole), method = "pearson")
AW_NoNormCor_P_sq = (AW_NoNormCor_P)^2
AW_NoNormCor_sq = (AW_NoNormCor)^2

PW_NoNormCor = cor(as.numeric(allSamples_NoNAs$Post),as.numeric(allSamples_NoNAs$Whole), method = "spearman")
PW_NoNormCor_P = cor(as.numeric(allSamples_NoNAs$Post),as.numeric(allSamples_NoNAs$Whole), method = "pearson")
PW_NoNormCor_P_sq = (PW_NoNormCor_P)^2
PW_NoNormCor_sq = (PW_NoNormCor)^2

AW_NormCor = cor((as.numeric(allSamples_NoNAs$Ant)/ as.numeric(merged_norm_1kb_AntvsWhole.lm$coefficients[2])),as.numeric(allSamples_NoNAs$Whole), method = "spearman")
AW_NormCor_P = cor((as.numeric(allSamples_NoNAs$Ant)/ as.numeric(merged_norm_1kb_AntvsWhole.lm$coefficients[2])),as.numeric(allSamples_NoNAs$Whole), method = "pearson")
AW_NormCor_P_sq = (AW_NormCor_P)^2
AW_NormCor_sq = (AW_NormCor)^2

PW_NormCor = cor((as.numeric(allSamples_NoNAs$Post)/ as.numeric(merged_norm_1kb_PostvsWhole.lm$coefficients[2])),as.numeric(allSamples_NoNAs$Whole), method = "spearman")
PW_NormCor_P = cor((as.numeric(allSamples_NoNAs$Post)/ as.numeric(merged_norm_1kb_PostvsWhole.lm$coefficients[2])),as.numeric(allSamples_NoNAs$Whole), method = "pearson")
PW_NormCor_P_sq = (PW_NormCor_P)^2
PW_NormCor_sq = (PW_NormCor)^2

############################ S1 Figs. ############################

############## No Normalization ############## 

### Ant vs Post - No Lin reg normalization
png('011918_AllRegions1KB_Scatter_Merged_PE_10M_NoNormalization_AntVsPost.png', width = 2000, height = 2000, units = "px",  res=300) 
ggplot(allSamples_NoNAs,
       aes(x = as.numeric(Ant),y= as.numeric(Post))) +
  #scale_color_manual(name = 'Legand', values=c("darkorange1" , "dodgerblue3")) +
  geom_point(colour="grey35", alpha = 0.2) +
  geom_density2d(colour="lightblue1") + 
  ggtitle('011918_AllRegions1KB_Scatter_Merged_PE_10M_NoNormalization_AntVsPost') +
  #scale_x_log10() +
  #scale_y_log10() +
  xlab('Anterior Halves merged') +
  ylab('Posterior Halves merged') +
  geom_smooth(method='lm', formula = y~x) +
  coord_equal() +
  ylim(0,3000) + 
  xlim(0,3000) +
  geom_abline(slope = 1, intercept=0, linetype = 'dotted') +
  #annotate("text", x=100, y=500, label= "slope = 0.996 ", colour = 'darkorange1') +
  #annotate("text", x=500, y=1500, label= Post_Prom_Label, colour = 'dodgerblue3') +
  theme(panel.border = element_blank(),
        panel.background = element_rect(fill= NA),
        axis.line = element_line(colour = "black"),
        axis.title.x = element_text(vjust = 0, size = 20),
        axis.title.y = element_text(vjust = 1, size = 20),
        axis.text.x = element_text(size=20),
        axis.text.y  = element_text(size=20),
        plot.title = element_text(size = 12),
        legend.text = element_text(size = 20),
        legend.title = element_text(size = 20),
        strip.text.x = element_text(size = 12),
        strip.text.y = element_text(size = 12))
dev.off()

############ S1Fig-A Whole Vs Ant ############ 

png('011918_AllRegions1KB_Scatter_Merged_PE_10M_NoNormalization_AntVsWhole.png', width = 2000, height = 2000, units = "px",  res=300) 
ggplot(allSamples_NoNAs,
       aes(x = as.numeric(Whole),y= as.numeric(Ant))) +
  geom_point(colour="grey35", alpha = 0.2) +
  geom_density2d(colour="lightblue1") + 
  ggtitle('011918_AllRegions1KB_Scatter_Merged_PE_10M_NoNormalization_WholevsAnt') +
  xlab('Whole Halves merged') +
  ylab('Ant Halves merged') +
  geom_smooth(method='lm', formula = y~x) +
  coord_equal() +
  ylim(0,3000) + 
  xlim(0,3000) +
  geom_abline(slope = 1, intercept=0, linetype = 'dotted') +
  theme(panel.border = element_blank(),
        panel.background = element_rect(fill= NA),
        axis.line = element_line(colour = "black"),
        axis.title.x = element_text(vjust = 0, size = 20),
        axis.title.y = element_text(vjust = 1, size = 20),
        axis.text.x = element_text(size=20),
        axis.text.y  = element_text(size=20),
        plot.title = element_text(size = 12),
        legend.text = element_text(size = 20),
        legend.title = element_text(size = 20),
        strip.text.x = element_text(size = 12),
        strip.text.y = element_text(size = 12))
dev.off()

############ S1Fig-C Whole Vs Post ############ 

png('011918_AllRegions1KB_Scatter_Merged_PE_10M_NoNormalization_WholevsPost.png', width = 2000, height = 2000, units = "px",  res=300) 
ggplot(allSamples_NoNAs,
       aes(x = as.numeric(Whole),y= as.numeric(Post))) +
  geom_point(colour="grey35", alpha = 0.2) +
  geom_density2d(colour="lightblue1") + 
  ggtitle('011918_AllRegions1KB_Scatter_Merged_PE_10M_NoNormalization_WholevsPost') +
  xlab('Whole Halves merged') +
  ylab('Post Halves merged') +
  geom_smooth(method='lm', formula = y~x) +
  coord_equal() +
  ylim(0,3000) + 
  xlim(0,3000) +
  geom_abline(slope = 1, intercept=0, linetype = 'dotted') +
  theme(panel.border = element_blank(),
        panel.background = element_rect(fill= NA),
        axis.line = element_line(colour = "black"),
        axis.title.x = element_text(vjust = 0, size = 20),
        axis.title.y = element_text(vjust = 1, size = 20),
        axis.text.x = element_text(size=20),
        axis.text.y  = element_text(size=20),
        plot.title = element_text(size = 12),
        legend.text = element_text(size = 20),
        legend.title = element_text(size = 20),
        strip.text.x = element_text(size = 12),
        strip.text.y = element_text(size = 12))
dev.off()

############## Lin Reg Normalization ##############
############## S1Fig-B
png('011918_AllRegions1KB_Scatter_Merged_PE_10M_Linregwhole_WholevsAnt.png', width = 2000, height = 2000, units = "px",  res=300) 
ggplot(allSamples_NoNAs,
       aes(x = as.numeric(Whole),y= (as.numeric(Ant)/ as.numeric(merged_norm_1kb_AntvsWhole.lm$coefficients[2])))) +
  geom_point(colour="grey35", alpha = 0.2) +
  geom_density2d(colour="lightblue1") + 
  ggtitle('011918_AllRegions1KB_Scatter_Merged_PE_10M_Linregwhole_WholevsAnt') +
  # scale_x_log10() +
  # scale_y_log10() +
  coord_equal() +
  ylim(0,3000) + 
  xlim(0,3000) +
  xlab('Whole Halves merged') +
  ylab('Ant LinRegWhole merged') +
  geom_smooth(method='lm', formula = y~x) +
  geom_abline(slope = 1, intercept=0, linetype = 'dotted') +
  theme(panel.border = element_blank(),
        panel.background = element_rect(fill= NA),
        axis.line = element_line(colour = "black"),
        axis.title.x = element_text(vjust = 0, size = 20),
        axis.title.y = element_text(vjust = 1, size = 20),
        axis.text.x = element_text(size=20),
        axis.text.y  = element_text(size=20),
        plot.title = element_text(size = 12),
        legend.text = element_text(size = 20),
        legend.title = element_text(size = 20),
        strip.text.x = element_text(size = 12),
        strip.text.y = element_text(size = 12))
dev.off()

png('011918_AllRegions1KB_Scatter_Merged_PE_10M_Linregwhole_WholevsPost.png', width = 2000, height = 2000, units = "px",  res=300) 
ggplot(allSamples_NoNAs,
       aes(x = as.numeric(Whole),y= (as.numeric(Post)/ as.numeric(merged_norm_1kb_PostvsWhole.lm$coefficients[2])))) +
  geom_point(colour="grey35", alpha = 0.2) +
  geom_density2d(colour="lightblue1") + 
  ggtitle('011918_AllRegions1KB_Scatter_Merged_PE_10M_Linregwhole_WholevsPost') +
  xlab('Whole Halves merged') +
  ylab('Post linregWhole') +
  geom_smooth(method='lm', formula = y~x) +
  coord_equal() +
  ylim(0,3000) + 
  xlim(0,3000) +
  geom_abline(slope = 1, intercept=0, linetype = 'dotted') +
  theme(panel.border = element_blank(),
        panel.background = element_rect(fill= NA),
        axis.line = element_line(colour = "black"),
        axis.title.x = element_text(vjust = 0, size = 20),
        axis.title.y = element_text(vjust = 1, size = 20),
        axis.text.x = element_text(size=20),
        axis.text.y  = element_text(size=20),
        plot.title = element_text(size = 12),
        legend.text = element_text(size = 20),
        legend.title = element_text(size = 20),
        strip.text.x = element_text(size = 12),
        strip.text.y = element_text(size = 12))
dev.off()
```

### Linear Regression Normalization values from above R script were then used to normalize the merged anterior and posterior wig files¶

In [ ]:

```
perl -n -e ' $Scale = 0.5787 ; $Yint = 0 ; if (/^\d+/) {@A = split (/\s+/, $_); $norm = ($A[1] - $Yint) / $Scale ; print "$A[0]\t $norm \n"} else { print "$_"}' 011718_MERGED_2reps_081817_RemDUP_041217_Bowtie2_ME_JH_112315_20Ant_shifted_lessthan130_10MNorm.wig > 011718_MERGED_2reps_081817_RemDUP_041217_Bowtie2_ME_JH_112315_20Ant_shifted_lessthan130_10MNorm_linregwhole.wig &
perl -n -e ' $Scale = 0.6063 ; $Yint = 0 ; if (/^\d+/) {@A = split (/\s+/, $_); $norm = ($A[1] - $Yint) / $Scale ; print "$A[0]\t $norm \n"} else { print "$_"}' 011718_MERGED_2reps_081817_RemDUP_041217_Bowtie2_ME_JH_112315_20Post_shifted_lessthan130_10MNorm.wig > 011718_MERGED_2reps_081817_RemDUP_041217_Bowtie2_ME_JH_112315_20Post_shifted_lessthan130_10MNorm_linregwhole.wig &


'wigToBigWig (3)' 011718_MERGED_2reps_081817_RemDUP_041217_Bowtie2_ME_JH_112315_20Ant_shifted_lessthan130_10MNorm_linregwhole.wig /Users/jennahaines/'Box Sync'/Eisen_Lab/Experiments/ATAC-seq/Halves/ATAC-seq_Pools/040617_Analysis/041717_NormalizedWigs/dm3.chrom.sizes 011718_MERGED_2reps_081817_RemDUP_041217_Bowtie2_ME_JH_112315_20Ant_shifted_lessthan130_10MNorm_linregwhole.bw
'wigToBigWig (3)' 011718_MERGED_2reps_081817_RemDUP_041217_Bowtie2_ME_JH_112315_20Post_shifted_lessthan130_10MNorm_linregwhole.wig /Users/jennahaines/'Box Sync'/Eisen_Lab/Experiments/ATAC-seq/Halves/ATAC-seq_Pools/040617_Analysis/041717_NormalizedWigs/dm3.chrom.sizes 011718_MERGED_2reps_081817_RemDUP_041217_Bowtie2_ME_JH_112315_20Post_shifted_lessthan130_10MNorm_linregwhole.bw
```

## DNAseI Read Alignment for S3 Fig.¶

### Raw data accessed via SRA¶

In [ ]:

```
######################################################################
##########   100317_SRAdump.sh                              ##########
######################################################################


#! /bin/bash

# already did all of stage 5

# stage 9 Rep 1
inputname[1]=SRR060800

# stage 9 Rep 2
inputname[2]=SRR060801  
inputname[3]=SRR060802  
inputname[4]=SRR060803  
inputname[5]=SRR060804  
inputname[6]=SRR060805  

# stage 10 Rep 1
inputname[7]=SRR060769  
inputname[8]=SRR060770  
inputname[9]=SRR060771  

# stage 10 Rep 2
inputname[10]=SRR060772 
inputname[11]=SRR060773 
inputname[12]=SRR060774 

#Stage 11 rep1
inputname[13]=SRR060775 
inputname[14]=SRR060776 

#Stage 11 rep2
inputname[15]=SRR060778 
inputname[16]=SRR060779 

#stage 14 rep1
inputname[17]=SRR060780 
inputname[18]=SRR060781 

#stage 14 rep2
inputname[19]=SRR060782 
inputname[20]=SRR060783 

for k in 1 2 3 4 5 6 7 8 9 10 11 12 13 14 15 16 17 18 19 20
do
    input=${inputname[${k}]}
    fastq-dump ${input}
    echo "done"
done
```

#### Bowtie2 Alignment of all replicates¶

In [ ]:

```
#100417_BDTNP_DNaseI_stage5
bowtie2 -p 10 -5 5 -3 5 -N 1 -X 2000 --local --very-sensitive-local --un 100417_BDTNP_DNaseI_stage5_ALLREPSCOMBINED_UNMAPPED.fastq -x ~/Indexed_Genomes/dmel-r5.57-index -U SRR060796.fastq,SRR060797.fastq,SRR060798.fastq,SRR060799.fastq -S 100417_BDTNP_DNaseI_stage5_ALLREPSCOMBINED.sam &

62986535 (100.00%) were unpaired; of these:
    32100628 (50.96%) aligned 0 times
    24785016 (39.35%) aligned exactly 1 time
    6100891 (9.69%) aligned >1 times
49.04% overall alignment rate

#100417_BDTNP_DNaseI_stage9
bowtie2 -p 10 -5 5 -3 5 -N 1 -X 2000 --local --very-sensitive-local --un 100417_BDTNP_DNaseI_stage9_ALLREPSCOMBINED_UNMAPPED.fastq -x ~/Indexed_Genomes/dmel-r5.57-index \
-U SRR060800.fastq,SRR060801.fastq,SRR060802.fastq,SRR060803.fastq,SRR060804.fastq,SRR060805.fastq \
-S 100417_BDTNP_DNaseI_stage9_ALLREPSCOMBINED.sam &

82176094 (100.00%) were unpaired; of these:
    39657640 (48.26%) aligned 0 times
    25870933 (31.48%) aligned exactly 1 time
    16647521 (20.26%) aligned >1 times
51.74% overall alignment rate

#100417_BDTNP_DNaseI_stage11
bowtie2 -p 10 -5 5 -3 5 -N 1 -X 2000 --local --very-sensitive-local --un 100417_BDTNP_DNaseI_stage11_ALLREPSCOMBINED_UNMAPPED.fastq -x ~/Indexed_Genomes/dmel-r5.57-index \
-U SRR060775.fastq,SRR060776.fastq,SRR060778.fastq,SRR060779.fastq \
-S 100417_BDTNP_DNaseI_stage11_ALLREPSCOMBINED.sam &

53097830 (100.00%) were unpaired; of these:
    23768088 (44.76%) aligned 0 times
    22849933 (43.03%) aligned exactly 1 time
    6479809 (12.20%) aligned >1 times
55.24% overall alignment rate

#100417_BDTNP_DNaseI_stage14
bowtie2 -p 10 -5 5 -3 5 -N 1 -X 2000 --local --very-sensitive-local --un 100417_BDTNP_DNaseI_stage14_ALLREPSCOMBINED_UNMAPPED.fastq -x ~/Indexed_Genomes/dmel-r5.57-index \
-U SRR060780.fastq,SRR060781.fastq,SRR060782.fastq,SRR060783.fastq \
-S 100417_BDTNP_DNaseI_stage14_ALLREPSCOMBINED.sam &

64010099 (100.00%) were unpaired; of these:
    32795498 (51.23%) aligned 0 times
    22046586 (34.44%) aligned exactly 1 time
    9168015 (14.32%) aligned >1 times
48.77% overall alignment rate
```

#### Remove Duplicates, Filter for quality, Convert to Wig, Normalized to 10 million reads as above¶

In [ ]:

```
################################################################################################
##########   011818_postalign_RemoveDuplicates_SortedBam_PairedEnd_Sam_FilterFragments_DNASEI.sh ##########
##########   Input:  sam file                                                          ##########
#########    Output: DupRemovedBam file                                               ##########
#########    ATAC shifted, 130 and below bed file, wig file, 10Mnormalized wig file   ##########
#########                                                                             ##########
################################################################################################

#bash 011818_postalign_RemoveDuplicates_SortedBam_PairedEnd_Sam_FilterFragments_DNASEI.sh 2> 011818_postalign_RemoveDuplicates_SortedBam_PairedEnd_Sam_FilterFragments_DNASEI_STERR.txt | cat > 011818_postalign_RemoveDuplicates_SortedBam_PairedEnd_Sam_FilterFragments_DNASEI_STDOUT.txt &

#! /bin/bash

#######  made a new working directory where everything will be in
#Directory='/netdata/jhaines/BDTNP_s5_DNase-seq/100317-SRADownload/011818_AnalysisforReviews_PE'

# original sam file
Bowtie2SamFile[1]=100417_BDTNP_DNaseI_stage5_ALLREPSCOMBINED.sam
Bowtie2SamFile[2]=100417_BDTNP_DNaseI_stage9_ALLREPSCOMBINED.sam
Bowtie2SamFile[3]=100417_BDTNP_DNaseI_stage11_ALLREPSCOMBINED.sam
Bowtie2SamFile[4]=100417_BDTNP_DNaseI_stage14_ALLREPSCOMBINED.sam


#Core name that will be carried with the sample
filesetname[1]=011818_100417_BDTNP_DNaseI_stage5_ALLREPSCOMBINED
filesetname[2]=011818_100417_BDTNP_DNaseI_stage9_ALLREPSCOMBINED
filesetname[3]=011818_100417_BDTNP_DNaseI_stage11_ALLREPSCOMBINED
filesetname[4]=011818_100417_BDTNP_DNaseI_stage14_ALLREPSCOMBINED

for k in 1 2 3 4
do
    samfile=${Bowtie2SamFile[${k}]}
    echo 'sam file imported'
    filesetname=${filesetname[${k}]}
    samtools view -bS -F 1804 -q 30 ${samfile} | samtools sort - ${filesetname}
    java -jar ~/scripts/picard.jar MarkDuplicates REMOVE_DUPLICATES=TRUE \
      I=${filesetname}.bam \
      O=${filesetname}_nodups.bam \
      M=${filesetname}_marked_dup_metrics.txt
    echo 'remove duplicates from bam complete'
    bedtools bamtobed -i ${filesetname}_nodups.bam > ${filesetname}_shifted.bed
    echo 'bed file created'
done
echo 'all samples done'
mkdir 011818_Bedfiles
mv *.bed 011818_Bedfiles
perl ~/scripts/xl-bed2wig-dirproc-HOA-2014-new-chrsz 011818_Bedfiles

#This script will take the filtered bed files.. count the number of reads and then use that to noramlize the wig files

for k in 1 2 3 4
do
  cd 011818_Bedfiles
  filesetname=${filesetname[${k}]}
  NumberofReads=$(wc -l < "${filesetname}_shifted.bed")
  echo "$NumberofReads"
  export NumberofReads
  echo "Normalizing to 1M reads"
  perl -n -e ' $Scale = 10000000/$ENV{NumberofReads} ; if (/^\d+/) {@A = split (/\s+/, $_); $norm = $A[1] * $Scale ; print "$A[0]\t $norm \n"} else { print "$_"}' ${filesetname}_shifted.wig > ${filesetname}_shifted_10MNorm.wig
  echo "${filesetname} Normalized wig File made"

done
```

## PEAKS¶

1) Called peaks using MACS2 with --nomodel and a p value threshold of 0.001 to find highly significant peaks.

2) Intersected replicate peaks to find a list of high confidence peaks for each sample as per ENCODE ATAC-seq guidelines

In [ ]:

```
##########################################################################################
##########   011818_RecallPeaksIDR_PE.sh   ###########
#########################################################################################
#bash 011818_RecallPeaksIDR_PE.sh 2> 011818_RecallPeaksIDR_PE_stderr.txt | cat > 011818_RecallPeaksIDR_PE_stdout.txt &
#! /bin/bash

#Import files
bedfile[1]=011617_081817_RemDUP_041217_Bowtie2_ME_JH_112315_1105-ATACSlice2-02-20p_shifted
bedfile[2]=011617_081817_RemDUP_041217_Bowtie2_ME_JH_112315_1105-ATACSlice2-02-20p_shifted_lessthan130
bedfile[3]=011617_081817_RemDUP_041217_Bowtie2_ME_JH_112315_1105-ATACSlice2-5-1A_shifted_lessthan130
bedfile[4]=011617_081817_RemDUP_041217_Bowtie2_ME_JH_112315_1105-ATACSlice2-6-1p_shifted_lessthan130
bedfile[5]=011617_081817_RemDUP_041217_Bowtie2_ME_JH_112315_1105-ATACSlice2-7-10whole_shifted_lessthan130
bedfile[6]=011617_081817_RemDUP_041217_Bowtie2_ME_JH_112315_1109-ATACSlice03-01-20Ant_shifted_lessthan130
bedfile[7]=011617_081817_RemDUP_041217_Bowtie2_ME_JH_112315_1109-ATACSlice03-02-20Post_shifted_lessthan130
bedfile[8]=011617_081817_RemDUP_041217_Bowtie2_ME_JH_112315_1109-ATACSlice03-03-1plus2_shifted_lessthan130
bedfile[9]=011617_081817_RemDUP_041217_Bowtie2_ME_JH_112315_1109-ATACSlice03-04-10whole_shifted_lessthan130
bedfile[10]=011617_101117_RemDUP_PE_041217_Bowtie2_ME_JH_112315_1105-ATACSlice2-01-20a_shifted_lessthan130
bedfile[11]=011718_MERGED_2reps_081817_RemDUP_041217_Bowtie2_ME_JH_112315_10whole_shifted_lessthan130
bedfile[12]=011718_MERGED_2reps_081817_RemDUP_041217_Bowtie2_ME_JH_112315_20Ant_shifted_lessthan130
bedfile[13]=011718_MERGED_2reps_081817_RemDUP_041217_Bowtie2_ME_JH_112315_20Post_shifted_lessthan130

for k in {2..13}
do
    bedfile=${bedfile[${k}]}
    perl -pe '$_ =~ tr/chr//d' ${bedfile}.bed > ${bedfile}_nochr.bed
    echo "Took out chr"
    macs2 callpeak --nomodel -f BEDPE -g dm -p 1e-3 --call-summits --bdg -t ${bedfile}_nochr.bed -n ${bedfile}_nochr
    echo "Called peaks"
    Peakfile=${bedfile[${k}]}_nochr_peaks.narrowPeak
    sort -k 8gr,8gr $Peakfile | awk 'BEGIN{OFS="\t"}{$4="Peak_"NR ; print $0}' | gzip -c > Sorted_$Peakfile.gz
done

#Make a directory for all of the peak files
mkdir 011818_RecallPeaksIDR_PE_Dir_PEAKS
mv *_nochr* 011818_RecallPeaksIDR_PE_Dir_PEAKS
cd 011818_RecallPeaksIDR_PE_Dir_PEAKS

#Anterior High Confidence replicate Overlap peaks
Antrep1=Sorted_011617_081817_RemDUP_041217_Bowtie2_ME_JH_112315_1109-ATACSlice03-01-20Ant_shifted_lessthan130_nochr_peaks.narrowPeak.gz
Antrep2=Sorted_011617_101117_RemDUP_PE_041217_Bowtie2_ME_JH_112315_1105-ATACSlice2-01-20a_shifted_lessthan130_nochr_peaks.narrowPeak.gz
Antmerged=Sorted_011718_MERGED_2reps_081817_RemDUP_041217_Bowtie2_ME_JH_112315_20Ant_shifted_lessthan130_nochr_peaks.narrowPeak.gz

#Intersect merged with rep 1 then intersect that with rep2
intersectBed -wo -a ${Antmerged} \
-b ${Antrep1} \
| awk 'BEGIN{FS="\t";OFS="\t"}{s1=$3-$2; s2=$13-$12; if (($21/s1 >= 0.5) || ($21/s2 >= 0.5))
{print $0}}' | cut -f 1-10 | sort | uniq | \
intersectBed -wo -a stdin -b ${Antrep2} \
| awk 'BEGIN{FS="\t";OFS="\t"}{s1=$3-$2; s2=$13-$12; if (($21/s1 >= 0.5) || ($21/s2 >= 0.5))
{print $0}}' | cut -f 1-10 | sort | uniq > 011818_ANTERIORPooledInRep1AndRep2.narrowPeak.gz

echo "Anterior High confidence peak file made"

# Posterior High Confidence replicate Overlap peaks
Postrep1=Sorted_011617_081817_RemDUP_041217_Bowtie2_ME_JH_112315_1105-ATACSlice2-02-20p_shifted_lessthan130_nochr_peaks.narrowPeak.gz
Postrep2=Sorted_011617_081817_RemDUP_041217_Bowtie2_ME_JH_112315_1109-ATACSlice03-02-20Post_shifted_lessthan130_nochr_peaks.narrowPeak.gz
Postmerged=Sorted_011718_MERGED_2reps_081817_RemDUP_041217_Bowtie2_ME_JH_112315_20Post_shifted_lessthan130_nochr_peaks.narrowPeak.gz

#Intersect merged with rep 1 
intersectBed -wo -a ${Postmerged} \
-b ${Postrep1} \
| awk 'BEGIN{FS="\t";OFS="\t"}{s1=$3-$2; s2=$13-$12; if (($21/s1 >= 0.5) || ($21/s2 >= 0.5))
{print $0}}' | cut -f 1-10 | sort | uniq | \
intersectBed -wo -a stdin -b ${Postrep2} \
| awk 'BEGIN{FS="\t";OFS="\t"}{s1=$3-$2; s2=$13-$12; if (($21/s1 >= 0.5) || ($21/s2 >= 0.5))
{print $0}}' | cut -f 1-10 | sort | uniq > 011818_POSTERIORPooledInRep1AndRep2.narrowPeak.gz

echo "Posterior High confidence peak file made"

# Whole High Confidence replicate Overlap peaks
Wholerep1=Sorted_011617_081817_RemDUP_041217_Bowtie2_ME_JH_112315_1105-ATACSlice2-7-10whole_shifted_lessthan130_nochr_peaks.narrowPeak.gz
Wholerep2=Sorted_011617_081817_RemDUP_041217_Bowtie2_ME_JH_112315_1109-ATACSlice03-04-10whole_shifted_lessthan130_nochr_peaks.narrowPeak.gz
Wholemerged=Sorted_011718_MERGED_2reps_081817_RemDUP_041217_Bowtie2_ME_JH_112315_10whole_shifted_lessthan130_nochr_peaks.narrowPeak.gz

#Whole merged with rep 1 
intersectBed -wo -a ${Wholemerged} \
-b ${Wholerep1} \
| awk 'BEGIN{FS="\t";OFS="\t"}{s1=$3-$2; s2=$13-$12; if (($21/s1 >= 0.5) || ($21/s2 >= 0.5))
{print $0}}' | cut -f 1-10 | sort | uniq | \
intersectBed -wo -a stdin -b ${Wholerep2} \
| awk 'BEGIN{FS="\t";OFS="\t"}{s1=$3-$2; s2=$13-$12; if (($21/s1 >= 0.5) || ($21/s2 >= 0.5))
{print $0}}' | cut -f 1-10 | sort | uniq > 011818_WholePooledInRep1AndRep2.narrowPeak.gz

echo "Whole High confidence peak file made"

echo "All Done! :)"
```

# Summarize average wig signal¶

1) Used perl script called wig\_sig\_around\_bedfile\_013018.pl (below) to generate a txt file containing average wig signal at 1kb intervals, with a column per normalized wig file. This file is then used to create the gray genomic regions in the scatterplots in figures 1, 3, and 4.

In [ ]:

```
perl wig_sig_around_bedfile_013018.pl \
~/Dmel_1kb_windows.bed \
~/011618_WigDirectory \
013118_wig_sig_around_bedfile_1kbwindows.txt
```

2) Used perl script called wig\_sig\_around\_bedfile\_013018.pl (below) to generate a txt file that averages normalized wig signal at genomic regions specified by my region file containing coordinates for select A-P patterned enhancers and promoters. I used this file both for randomization script

In [ ]:

```
perl /Users/jennahaines/'Box Sync'/Eisen_Lab/Scripts/wig_sig_around_bedfile_013018.pl \
~/012918_Redid_011618_ReviewsRevised_RevisedAPDV_enhPromRegions.bed \
~/011618_WigDirectory \
013118_wig_sig_around_bedfile_012318GENELIST.txt
```

In [ ]:

```
#wig_sig_around_bedfile_013018.pl
#!/usr/bin/perl

use warnings;

#average signal over a bed region
my $peaks =shift; #bed file 
my $dir = shift;  #wig file dir
# my $NF = shift;  #normalization table
my $output = shift;
#my $win = shift;

# my %NF_t;

# open (NFT,'<', $NF);
# while (<NFT>){
#  next if(/#/);

#  my ($FS, $v) = split /\s+/,$_;
# $NF_t{$FS} = $v;

# }

my %HOAg=();

open (OUTPUT, '>', $output);
#print OUTPUT "#gen_ann,average signal within cluster region\n";
#print OUTPUT "#ID\tchr\tpeak_pos1\tpeak_pos2\tinterval_length\t";  #for union of peaks file
open (INPUTp, '<', $peaks);

my $N=1;
while (<INPUTp>){
   if (/^chr(\d|X)/) {
    @split = split (/\s+/, $_);

   # my $PID = $split[0];
    $HOAg{$N} = [@split];
    $N +=1;
  } else {
     @split = split (/\s+/, $_);
     $st = join ("\t",@split);
     print OUTPUT "$st";
    }
}


opendir DIR, $dir or die "can't open $dir: $!";

while ( my $file = readdir DIR ) {
  if ($file =~ /(.+).wig$/) {
    $HM = "$1";
    print OUTPUT "\t$HM";

    $In_file = "$dir/$file";
    print "infile:$file\n";

    my %HOA;
    my $chr2 = "NA";
    my %chr_sz=(
	   "chr2L" => 23011344, #new size info, aug 2014
	   "chr2R" => 21146608,
	   "chr3L" => 24543457,
	   "chr3R" => 27905053,
	   "chr4" => 1351757,
	   "chrX" => 22332727);

    foreach $chr (keys %chr_sz){
      $n = int($chr_sz{$chr}/10);
      @a = (0)x$n;
      $HOA{$chr} = [@a];
		}


    open (INPUT, '<', $In_file);
    while ( <INPUT> ) {
      if ($_ =~/^tr/){
	next}elsif ($_ =~ /chrom=(\w+)\s+/){
	  $chr2 = $1;
	}elsif ($_ =~/^\d+/){
	  @s=split (/\s+/, $_);
	  $p =int( $s[0]/10);		      		     
	  $HOA{$chr2}[$p] += $s[1];		     
	}
}
close INPUT;

    my $total=0;
    my $L;
    foreach $i (keys %HOAg){
      $chr3 = $HOAg{$i}[0];  #
  
   $st_p = int($HOAg{$i}[1]/10); # 5873439 becomes 587343
   $en_p = int($HOAg{$i}[2]/10);

    $L = ($en_p - $st_p);


      @ar= @{$HOA{$chr3}}[$st_p..$en_p];
      #print "@ar\n";

      $total += $_ for @ar;
  $val = $total/$L;
  #$vf= sprintf("%.2f", $val);
  push @{$HOAg{$i}}, $val;
   #push @{$HOAg{$i}}, $total;
	  
      $total=0
    }
  }
}

print OUTPUT "\n";

foreach $i (sort {$a <=> $b}(keys %HOAg)){
  $str = join ("\t", @{$HOAg{$i}});
print OUTPUT "$str\n"
}
close DIR;
close OUTPUT;
```

### Randomization Script¶

#### This script will take the random regions and the patterned regions , calculate the ATACSkew score (also known as the accessibility skew score) for both and match one random region up to each patterned region such that the overall total signal matches.¶

#### The matched set of random regions is then used to produce a normal curve - the standard deviation and average of which are then used to calculate the p value and Z score for each region.¶

In [ ]:

```
############################## Randomization script ####################
# Make a list of random regions excluding genes and patterning regions
bedtools intersect -v -a 081517_Dmel5_1E6_randomregions.bed -b 081517_newgenelist_stripped_plus_FlybaseGenes_coordinates.bed > 081517_RandomRegions_Excl_flybasegenes_Enhancers.bed

# Random region match up 

perl ~/Scripts/wig_sig_around_bedfile_013018.pl \
013118_081517_RandomRegions_Excl_flybasegenes_Enhancers.bed \
~/013118_RandomWigDirectory \
013118_wig_sig_around_bedfile_randomregions.txt


#063017_RandomDistributionScript_V5.py
python 012418_RandomDistributionScript_V5.py \
-W 013118_wig_sig_around_bedfile_randomregions.txt \
-R 013118_wig_sig_around_bedfile_012318GENELIST_matched.txt \
-o 013118_012318GENELIST_Randomregv2_output.txt

echo "randomization script complete"
```

In [ ]:

```
####################################################################
######### 012418_RandomDistributionScript_V5.py             #########
######### Take a bed file and find random regions           #########
######### that have same overall wig signal                 #########
######### ouput : Random Region overlap file, and histograms#########
#####################################################################

#!/usr/bin/env python


import os
from math import *
from optparse import OptionParser
import sys
import re
from pylab import figure, title, xlabel, ylabel, hist, axis, grid, savefig
import matplotlib.pyplot as plt
import numpy as np
from scipy.stats import norm
from random import shuffle

################# Argument Import : Uncomment for actual running script  #################

def parse_options():
	parser = OptionParser()
	parser.add_option("-W", "--wigfile", dest="wigfile",
					  help="Random region file - 4 columned TXT file (unix line endings) with columns : #'chr'	'start'	'end'	'041417_Bowtie2_ATACPools_merged_20Anterior_10MDmel.bw'	'062817_LinregNorm_041417_Bowtie2_ATACPools_merged_20Posterior_10MDmel.bw'")
	parser.add_option("-R", "--regfile", dest="regfile",
					  help="5 columned TXT file of wanted genomic regions columns: chr	start	end	Type	New Location Assignment	Name	Blind use?	Old Location Assignment	Insitu?	Blind Notes	Length	FlybaseID	Source	#'chr'	'start'	'end'	'041417_Bowtie2_ATACPools_merged_10Whole_10MDmel.bw'	'081217_LinregNormWhole_041417_Bowtie2_ATACPools_merged_20Anterior_10MDmel.bw'	'081217_LinregNormWhole_041417_Bowtie2_ATACPools_merged_20Posterior_10MDmel.bw'	'081217_LinregNormWhole_041417_Bowtie2_ATACPools_merged_AplusP_10MDmel.bw'	'101416_10MNorm_DNase-I_dmels5r1.bw'")
	parser.add_option("-o", "--output", dest="output",
					  help="name of output file")
	(options, args) = parser.parse_args()
	return options
            
options = parse_options()
parser = OptionParser()
if not options.wigfile:
    print("Wigfile option is missing\n")
    parser.print_help()
    exit(-1)
if not options.regfile:
    print("Region file option is missing\n")
    parser.print_help()
    exit(-1)
if not options.output:
    print("output file is missing\n")
    parser.print_help()
    exit(-1)
    
################# Data Import   #################
enhancerList_file = open(str(options.regfile), 'r')
enhancerList = enhancerList_file.readlines()
enhancerList_file.close()

#   B. Read in Random region ATAC file (zeros included)
#Header : #'chr'	'start'	'end'	'041417_Bowtie2_ATACPools_merged_20Anterior_10MDmel.bw'	'062817_LinregNorm_041417_Bowtie2_ATACPools_merged_20Posterior_10MDmel.bw'
randomList_file = open(str(options.wigfile), 'r')
randomList = randomList_file.readlines()
randomList_file.close()

#   D. Take out the header of both random and enhancer ATAC files. it is a list
enhFirstLine = enhancerList.pop(0)
enhFirstLine = enhFirstLine.strip("/n")
randFirstLine = randomList.pop(0)
randFirstLine = randFirstLine.strip("/n")

print("files imported")

################# Create Dictionaries for region and random files   #################
enhListElement = []
randomListElement = []
enhScoreDict = {}
enhInfoDict = {}
enhLocDict = {}
enhTypeDict = {}
enhAntDict = {}
enhPostRDict = {}
enhNameList = []
Insitu_use ={}


# For each enhancer, strip the line ending, and split up line by \t 
# make a dictionary for score and dictionary for the line by name

for line in enhancerList:
    line2 = line.strip('\n')
    enhListElement = line2.split('\t')
    enhNameList.append(enhListElement[5])
    EnhancerTotalScore = float(enhListElement[25]) + float(enhListElement[27])
    enhScoreDict[enhListElement[5]] = EnhancerTotalScore
    enhInfoDict[enhListElement[5]] = line2
    enhLocDict[enhListElement[5]] = enhListElement[4]
    enhTypeDict[enhListElement[5]] = enhListElement[3]
    enhAntDict[enhListElement[5]] = enhListElement[25]
    enhPostRDict[enhListElement[5]] = enhListElement[27]
    Insitu_use[enhListElement[5]] = enhListElement[6]


# Calculate the ATACskewScore and add it to the output file
EnhancerATACSkewScoreList =[]
AnteriorATACSkewScoreList = []
PosteriorATACSkewScoreList =[]
PosteriorPromoterATACSkewScoreList =[]
AnteriorPromoterATACSkewScoreList =[]
PromoterATACSkewScoreList =[]
Insitu_use_List =[]
ATACSkewScoreDict = {}


for enhancer in enhNameList:
    if Insitu_use[enhancer] == "yes": #if used in the final list
        if enhTypeDict[enhancer] == "Enhancer":
            if enhLocDict[enhancer] == "Anterior" or enhLocDict[enhancer] == "Mostly Ant" :
                ATACSkewScore = (float(enhAntDict[enhancer]) - float(enhPostRDict[enhancer])) / (float(enhAntDict[enhancer]) + float(enhPostRDict[enhancer]))
                print("Anterior: ", enhancer)
                EnhancerATACSkewScoreList.append(ATACSkewScore) #add it to the general enhancers list
                AnteriorATACSkewScoreList.append(ATACSkewScore) #add it to the anterior enhancers only list
            if enhLocDict[enhancer] == "Posterior" or enhLocDict[enhancer] == "Mostly Post" :
                ATACSkewScore = (float(enhPostRDict[enhancer]) - float(enhAntDict[enhancer])) / (float(enhAntDict[enhancer]) + float(enhPostRDict[enhancer]))
                print("Posterior: ", enhancer)
                EnhancerATACSkewScoreList.append(ATACSkewScore) #add it to the general enhancers list
                PosteriorATACSkewScoreList.append(ATACSkewScore) #add it to the posterior enhancers list
            else: #DV enhancers
                ATACSkewScore = (float(enhAntDict[enhancer]) - float(enhPostRDict[enhancer])) / (float(enhAntDict[enhancer]) + float(enhPostRDict[enhancer]))
                EnhancerATACSkewScoreList.append(ATACSkewScore)
        else: #promoter
            if enhLocDict[enhancer] == "Anterior" or enhLocDict[enhancer] == "Mostly Ant" :
                ATACSkewScore = (float(enhAntDict[enhancer]) - float(enhPostRDict[enhancer])) / (float(enhAntDict[enhancer]) + float(enhPostRDict[enhancer]))
                PromoterATACSkewScoreList.append(ATACSkewScore) #add it to the promoters list
                AnteriorPromoterATACSkewScoreList.append(ATACSkewScore) #add it to the anterior promoters list
            if enhLocDict[enhancer] == "Posterior" or enhLocDict[enhancer] == "Mostly Post" :
                ATACSkewScore = (float(enhPostRDict[enhancer]) - float(enhAntDict[enhancer])) / (float(enhAntDict[enhancer]) + float(enhPostRDict[enhancer]))
                PromoterATACSkewScoreList.append(ATACSkewScore) #add it to the promoters list
                PosteriorPromoterATACSkewScoreList.append(ATACSkewScore) #add it to the posterior promoters list
            else: #DV promoters
                ATACSkewScore = (float(enhAntDict[enhancer]) - float(enhPostRDict[enhancer])) / (float(enhAntDict[enhancer]) + float(enhPostRDict[enhancer]))
                PromoterATACSkewScoreList.append(ATACSkewScore)
        ATACSkewScoreDict[enhancer] = ATACSkewScore
print("ATACSkewScores Calculated!")


###### Need to shuffle the random region list and peform the following standard deviation calculation
#Import Random regions, calculate the Random skew score for each region
RandScoreDict = {}
RandInfoDict = {}
EnhrandATACSkewScoreList = []
RandomATACSkewScoreDict = {}
PromrandATACSkewScoreList =[]
randNamelist =[]
perm_counter = 0

##### Mu and Std Lists
mu_enh_list =[]
mu_prom_list = []
std_enh_list = []
std_prom_list = []

for line in randomList:
    line2 = line.strip('\n')
    randomListElement = line2.split('\t')
    RandomTotalScore = float(randomListElement[3]) + float(randomListElement[4])
    RandName = "-".join([randomListElement[0], randomListElement[1], randomListElement[2]])
    randNamelist.append(RandName)
    RandScoreDict[RandName] = RandomTotalScore
    RandInfoDict[RandName] = line2
    if RandomTotalScore != 0:
        randATACSkewScore = (float(randomListElement[3]) - float(randomListElement[4])) / RandomTotalScore
        RandomATACSkewScoreDict[RandName] = randATACSkewScore
            

RandUniqueDict = {}
enhRandregionMatchDict ={}
MatchedRandomATACSkewScoreDict ={}

shuffle(randNamelist)
for enhancer in enhNameList:
    if Insitu_use[enhancer] == "yes": #if used in the final list
        enhancerCounter = 0
        TotalScore = enhScoreDict[enhancer]
        for randomkey in randNamelist:
            if enhancerCounter < 1:
                if randomkey not in RandUniqueDict:
                    randValue = RandScoreDict[randomkey]
                    if TotalScore * 0.8 < randValue < TotalScore * 1.1:
                        enhancerCounter += 1
                        EnhrandATACSkewScoreList.append(RandomATACSkewScoreDict[randomkey])
                        RandUniqueDict[randomkey] = True
                        enhRandregionMatchDict[enhancer] = RandInfoDict[randomkey]
                        MatchedRandomATACSkewScoreDict[enhancer] = RandomATACSkewScoreDict[randomkey]
                        print("Match!")
                        break
# Calculate Mu and Std from RandATAC Score List
mu_enh, std_enh = norm.fit(EnhrandATACSkewScoreList)
print("mu_enh: ", mu_enh, "std_enh :",  std_enh)

# Making the output file
print("Making output file")
outputfileName = str(options.output)
outputFile = open(str(outputfileName), 'w')
outputheader = "\t".join([enhFirstLine.strip("\n"), 'ATACSkewScore', randFirstLine.strip("\n"), 'RandSkewScore' , 'ZScore', 'PValue', "\n"])
outputFile.write(outputheader)

for enhancer in enhNameList:
    if Insitu_use[enhancer] == "yes":
        outputFile.write(enhInfoDict[enhancer])
        outputFile.write("\t")
        outputFile.write(str(ATACSkewScoreDict[enhancer]))
        outputFile.write("\t")
        EnhancerZscore = (ATACSkewScoreDict[enhancer] - float(mu_enh))/float(std_enh)
        enhpValue = norm.sf(abs((EnhancerZscore)))*2
        outputFile.write(enhRandregionMatchDict[enhancer])
        outputFile.write("\t")
        outputFile.write(str(MatchedRandomATACSkewScoreDict[enhancer]))
        outputFile.write("\t")
        outputFile.write(str(EnhancerZscore))
        outputFile.write('\t')
        outputFile.write(str(enhpValue))
        outputFile.write('\n')
outputFile.close() 

#Making the graphs

#random region histogram
plt.hist(EnhrandATACSkewScoreList, bins=20, normed=True, alpha=0.6, color='r')
xmin, xmax = plt.xlim()
x = np.linspace(xmin, xmax, 100)
p = norm.pdf(x, mu_enh, std_enh)
plt.plot(x, p, 'k', linewidth=2)
title = "Fit results: mu = %.2f,  std = %.2f" % (mu_enh, std_enh)
plt.title(title)
savefig('RandomRegionHistogram.png')
plt.close()
print("Done calculating Enhancer_RandomRegion Curve")
```

# Figure Generation R script¶

##### Note : For comaprisons to DnaseI, we used the previously published Dnase-I stage 5 replicate 1 wig file from BDTNP.¶

In [ ]:

```
############### ################ ################ ################ ################
################ 011818_RefinedGeneList_Dataanalysis_linregv2_PEReviews.r     ################ 
################ make scatterplots with ATAC Score and position               ################ 
################calculated in 051117_randomdistribution_script                ################
################ ################ ################ ################ ################


library("plyr")
library("dplyr")
library("ggplot2")

######### ######### ######### Input ######### ######### ######### ######### ######### ######### 

allSamples <-read.delim2("021218_012318GENELIST_Randomregv2_output.txt", sep = "\t", stringsAsFactors = FALSE)
allSamples_1kb_bins <- read.delim2("021218_wig_sig_around_bedfile_1kbwindows_all.txt", sep = "\t", stringsAsFactors = FALSE)
allPeaksFile <- read.delim2("021518_2reps_REppeaks_overlap_REVISEDGENELIST.txt", sep ="\t", stringsAsFactors = FALSE, header = FALSE)

####### Peak matching --> Match the filtered peak file back to the sample list #######
# Parse all the peaks out by sample, then only keep the highest fold change per sample, then match it back to the original dataframe
allPeaks = allPeaksFile %>%
  select(V1, V2, V3, V6, V11, V12, V13, V17, V21)
colnames(allPeaks) = c("chr", "start", "end", "name", "chrPeak",	"startPeak",	"endPeak", "fold_enrichment", "Peakname")

allPeaksWhole <- allPeaks %>%
  dplyr::filter(grepl(pattern = "Whole", Peakname)) %>%
  group_by(name) %>%
  arrange(as.numeric(fold_enrichment)) %>%
  dplyr::top_n(1, as.numeric(fold_enrichment))
allPeaksAnt <- allPeaks %>%
  dplyr::filter(grepl(pattern = "Anterior", Peakname)) %>%
  group_by(name) %>%
  arrange(as.numeric(fold_enrichment)) %>%
  dplyr::top_n(1, as.numeric(fold_enrichment))
allPeaksPost <- allPeaks %>%
  dplyr::filter(grepl(pattern = "Posterior", Peakname)) %>%
  group_by(name) %>%
  arrange(as.numeric(fold_enrichment)) %>%
  dplyr::top_n(1, as.numeric(fold_enrichment))
allPeaksNone <- allPeaks %>%
  dplyr::filter(Peakname == ".")

#Add back to allSamples
allSamples$WholePeaks = as.numeric(allPeaksWhole$fold_enrichment)[match(allSamples$Name, allPeaksWhole$name)]
allSamples$AntPeaks = as.numeric(allPeaksAnt$fold_enrichment)[match(allSamples$Name, allPeaksAnt$name)]
allSamples$PostPeaks = as.numeric(allPeaksPost$fold_enrichment)[match(allSamples$Name, allPeaksPost$name)]
allSamples$NoPeak = as.character(allPeaksNone$fold_enrichment)[match(allSamples$Name, allPeaksNone$name)]

################# Filter out regions that were confirmed spatially resolved in situ expression and overlap peaks
allSamples_yes_peaks = allSamples %>%
  filter(X011617.use == "yes") %>%
  filter(is.na(NoPeak) == TRUE) %>%
  select(chr, start, end, X011718_MERGED_2reps_081817_RemDUP_041217_Bowtie2_ME_JH_112315_10whole_shifted_lessthan130_10MNorm, X011718_MERGED_2reps_081817_RemDUP_041217_Bowtie2_ME_JH_112315_20Ant_shifted_lessthan130_10MNorm_linregwhole, X011718_MERGED_2reps_081817_RemDUP_041217_Bowtie2_ME_JH_112315_20Post_shifted_lessthan130_10MNorm_linregwhole, X011617_081817_RemDUP_041217_Bowtie2_ME_JH_112315_1109.ATACSlice03.03.1plus2_shifted_lessthan130_1MNorm, X101416_10MNorm_DNase.I_dmels5r1, Name,New.Location.Assignment, Type) %>%
  mutate(Dotsize = 1.5)
colnames(allSamples_yes_peaks) = c("Chr", "Start", "End", "Whole", "Ant", "LinregPost", "AplusP", "Dnase1", "Name", "Location", "Type", "Dotsize")


AP_Enhancers = allSamples_yes_peaks %>%
  filter(Type == "Enhancer") %>%
  filter(Location %in% c('Anterior', "Posterior", "Mostly Ant", "Mostly Post"))

AP_Promoters = allSamples_yes_peaks %>%
  filter(Type == "Promoter") %>%
  filter(Location %in% c('Anterior', "Posterior", "Mostly Ant", "Mostly Post"))

DV_Enhancers = allSamples_yes_peaks %>%
  filter(Type == "Enhancer") %>%
  filter(Location %in% c('Dorsal', "Ventral"))

DV_Promoters = allSamples_yes_peaks %>%
  filter(Type == "Promoter") %>%
  filter(Location %in% c('Dorsal', "Ventral"))
```

#### Figure 1¶

In [ ]:

```
######### ######### ######### Figure 1 Scatterplots of 1kb regions

################# filter our NANs in the 1kb bin file and make standard column names
allSamples_NoNAs = allSamples_1kb_bins %>%
  select(X.chr., X.start., X.end., X011718_MERGED_2reps_081817_RemDUP_041217_Bowtie2_ME_JH_112315_10whole_shifted_lessthan130_10MNorm, X011718_MERGED_2reps_081817_RemDUP_041217_Bowtie2_ME_JH_112315_20Ant_shifted_lessthan130_10MNorm_linregwhole, X011718_MERGED_2reps_081817_RemDUP_041217_Bowtie2_ME_JH_112315_20Post_shifted_lessthan130_10MNorm_linregwhole, X011617_081817_RemDUP_041217_Bowtie2_ME_JH_112315_1109.ATACSlice03.03.1plus2_shifted_lessthan130_1MNorm, X101416_10MNorm_DNase.I_dmels5r1) %>%
  filter(X011718_MERGED_2reps_081817_RemDUP_041217_Bowtie2_ME_JH_112315_10whole_shifted_lessthan130_10MNorm != 'nan') %>%
  filter(X011718_MERGED_2reps_081817_RemDUP_041217_Bowtie2_ME_JH_112315_20Ant_shifted_lessthan130_10MNorm_linregwhole!= 'nan') %>%
  filter(X011617_081817_RemDUP_041217_Bowtie2_ME_JH_112315_1109.ATACSlice03.03.1plus2_shifted_lessthan130_1MNorm!= 'nan') %>%
  filter(X101416_10MNorm_DNase.I_dmels5r1!= 'nan') %>%
  filter(X011718_MERGED_2reps_081817_RemDUP_041217_Bowtie2_ME_JH_112315_20Post_shifted_lessthan130_10MNorm_linregwhole!= 'nan') %>%
  mutate(Name = "NA") %>%
  mutate(Location = "NA") %>%
  mutate(Type = "NA") %>%
  mutate(Dotsize = 1)
colnames(allSamples_NoNAs) = c("Chr", "Start", "End", "Whole", "Ant", "LinregPost", "AplusP", "Dnase1", "Name", "Location", "Type", "Dotsize")


######### ######### ######### Ant

### Correction new graph for figure 1 that is just AvsP
AvsP_cor = cor(as.numeric(allSamples_NoNAs$Ant),y= as.numeric(allSamples_NoNAs$LinregPost), method = "spearman")
AvsP_cor_Pearson = cor(as.numeric(allSamples_NoNAs$Ant),y= as.numeric(allSamples_NoNAs$LinregPost), method = "pearson")
AvsP_cor_rsq = (AvsP_cor)^2
AvsP_cor_Pearson_rsq = (AvsP_cor_Pearson)^2


png('020118_PE2reps_nodups_LinRegWhole_AllRegions1KB_AvsP_log.png', width = 4000, height = 2000, units = "px",  res=300) 
ggplot(allSamples_NoNAs,
       aes(x = as.numeric(LinregPost),y= as.numeric(Ant), colour = as.factor(Location))) +
  scale_color_manual(values=c("lightsteelblue4")) +
  geom_point(alpha = 0.05, size = 0.3, show.legend = FALSE) +
  geom_density2d(colour="lightskyblue2", alpha = 0.5, size = 0.4) + 
  ggtitle('020118_PE_2reps_nodups_LinRegWhole_AllRegions1KB_AvsP') +
  scale_x_log10(limits = c(10,1500)) +
  scale_y_log10(limits = c(10,1500)) +
  xlab('Reg Norm Whole Posterior') +
  ylab('Reg Norm Whole Anterior') +
  coord_equal() +
  geom_abline(slope = 1, intercept=0, linetype = 'dotted') +
  annotate("text", x=1000, y=20, label= AvsP_cor) +
  theme(panel.border = element_blank(),
        panel.background = element_rect(fill= NA),
        axis.line = element_line(colour = "black"),
        axis.title.x = element_text(vjust = 0, size = 20),
        axis.title.y = element_text(vjust = 1, size = 20),
        axis.text.x = element_text(size=20),
        axis.text.y  = element_text(size=20),
        plot.title = element_text(size = 12),
        legend.text = element_text(size = 20),
        legend.title = element_text(size = 20),
        strip.text.x = element_text(size = 12),
        strip.text.y = element_text(size = 12))
dev.off()


png('020118_PE2reps_nodups_LinRegWhole_AllRegions1KB_AvsP_nolog.png', width = 4000, height = 2000, units = "px",  res=300) 
ggplot(allSamples_NoNAs,
       aes(x = as.numeric(LinregPost),y= as.numeric(Ant), colour = as.factor(Location))) +
  scale_color_manual(values=c("lightsteelblue4")) +
  geom_point(alpha = 0.05, size = 0.3, show.legend = FALSE) +
  geom_density2d(colour="lightskyblue2", alpha = 0.5, size = 0.4) + 
  ggtitle('020118_PE_2reps_nodups_LinRegWhole_AllRegions1KB_AvsP') +
  xlab('Reg Norm Whole Posterior') +
  ylab('Reg Norm Whole Anterior') +
  ylim(0,1500) + 
  xlim(0,1500) +
  coord_equal() +
  geom_abline(slope = 1, intercept=0, linetype = 'dotted') +
  annotate("text", x=1000, y=20, label= AvsP_cor) +
  theme(panel.border = element_blank(),
        panel.background = element_rect(fill= NA),
        axis.line = element_line(colour = "black"),
        axis.title.x = element_text(vjust = 0, size = 20),
        axis.title.y = element_text(vjust = 1, size = 20),
        axis.text.x = element_text(size=20),
        axis.text.y  = element_text(size=20),
        plot.title = element_text(size = 12),
        legend.text = element_text(size = 20),
        legend.title = element_text(size = 20),
        strip.text.x = element_text(size = 12),
        strip.text.y = element_text(size = 12))
dev.off()
```

#### Figure 3A, 3B, 4A, 4B¶

In [ ]:

```
################# merge the regions with the 1kb windows
MergedALL = rbind(allSamples_NoNAs, allSamples_yes_peaks)


######### ######### ######### AP ENHANCER SCATTERPLOT
AP_Enhancers_windows = MergedALL %>%
  filter(Type %in% c("Enhancer", "NA")) %>%
  filter(Location %in% c("NA", 'Anterior', "Posterior", "Mostly Post"))
AP_Enhancers_windows$Location <- factor(AP_Enhancers_windows$Location,
                                        levels = c("NA", 'Anterior', "Mostly Post", 'Posterior') ,ordered = TRUE)

png('020118_PE_2reps_nodups_AllRegions1KB_RegNormWhole_APEnhancers_Scatter_0118Newgenelist.png', width = 4000, height = 2000, units = "px",  res=300) 
ggplot(AP_Enhancers_windows,
       aes(x = as.numeric(Ant),y= as.numeric(LinregPost), colour = as.factor(Location))) +
  scale_color_manual(name = 'Legand', values=c("grey70","darkorange1",  "dodgerblue3", "dodgerblue3")) +
  geom_point(alpha = 0.8, aes(size = Dotsize)) +
  scale_size_continuous(range = c(1, 2)) +
  ggtitle('020118_PE_2reps_nodups_AllRegions1KB_RegNormWhole_APEnhancers_Scatter_0118Newgenelist') +
  xlab('Reg Norm Whole Anterior') +
  ylab('Reg Norm Whole Posterior') +
  ylim(0,1500) + 
  xlim(0,1500) +
  coord_equal() +
  geom_abline(slope = 1, intercept=0, linetype = 'dotted') +
  theme(panel.border = element_blank(),
        panel.background = element_rect(fill= NA),
        axis.line = element_line(colour = "black"),
        axis.title.x = element_text(vjust = 0, size = 20),
        axis.title.y = element_text(vjust = 1, size = 20),
        axis.text.x = element_text(size=20),
        axis.text.y  = element_text(size=20),
        plot.title = element_text(size = 12),
        legend.text = element_text(size = 20),
        legend.title = element_text(size = 20),
        strip.text.x = element_text(size = 12),
        strip.text.y = element_text(size = 12))
dev.off()

######### ######### ######### DV ENHANCER SCATTERPLOT
DV_Enhancers_windows = MergedALL %>%
  filter(Type %in% c("Enhancer", "NA")) %>%
  filter(Location %in% c("NA", 'Dorsal', "Ventral"))
DV_Enhancers_windows$Location <- factor(DV_Enhancers_windows$Location,
                                        levels = c("NA", 'Dorsal', 'Ventral') ,ordered = TRUE)

png('020118_PE_2reps_nodups_AllRegions1KB_DVEnhancers_LinRegWhole_Scatter_0118newgeneList.png', width = 4000, height = 2000, units = "px",  res=300) 
ggplot(DV_Enhancers_windows,
       aes(x = as.numeric(Ant),y= as.numeric(LinregPost), colour = as.factor(Location))) +
  scale_color_manual(name = 'Legend', values=c( "grey70", "orchid4", "seagreen4")) +
  geom_point(alpha = 0.9, aes(size = Dotsize)) +
  scale_size_continuous(range = c(1, 2)) +
  ggtitle('020118_PE_2reps_nodups_AllRegions1KB_DVEnhancers_LinRegWhole_Scatter_0118newgeneList') +
  xlab('LinregWhole Anterior') +
  ylab('LinregWhole  Posterior') +
  ylim(0,1500) + 
  xlim(0,1500) +
  coord_equal() +
  geom_abline(slope = 1, intercept=0, linetype = 'dotted') +
  theme(panel.border = element_blank(),
        panel.background = element_rect(fill= NA),
        axis.line = element_line(colour = "black"),
        axis.title.x = element_text(vjust = 0, size = 20),
        axis.title.y = element_text(vjust = 1, size = 20),
        axis.text.x = element_text(size=20),
        axis.text.y  = element_text(size=20),
        plot.title = element_text(size = 20),
        legend.text = element_text(size = 20),
        legend.title = element_text(size = 20),
        strip.text.x = element_text(size = 20),
        strip.text.y = element_text(size = 20))
dev.off()


######### ######### ######### AP Promoters SCATTERPLOT

AP_Promoters_windows = MergedALL %>%
  filter(Type %in% c("Promoter", "NA")) %>%
  filter(Location %in% c("NA", 'Anterior', "Posterior", "Mostly Ant", "Mostly Post"))
AP_Promoters_windows$Location <- factor(AP_Promoters_windows$Location,
                                        levels = c("NA", 'Anterior', "Mostly Ant", "Mostly Post", 'Posterior') ,ordered = TRUE)


png('020118_PE_2reps_nodups_AllRegions1KB_APPromoters_LinRegWhole_Scatter_0118newgeneList.png', width = 4000, height = 2000, units = "px",  res=300) 
ggplot(AP_Promoters_windows,
       aes(x = as.numeric(Ant),y= as.numeric(LinregPost), colour = as.factor(Location))) +
  scale_color_manual(name = 'Legand', values=c("grey70","darkorange1" , "darkorange1",  "dodgerblue3", "dodgerblue3")) +
  geom_point(alpha = 1, aes(size = Dotsize)) +
  scale_size_continuous(range = c(1, 2)) +
  ggtitle('020118_PE_2reps_nodups_AllRegions1KB_APPromoters_LinRegWhole_Scatter_0118newgeneList') +
  xlab('LinRegWhole Anterior') +
  ylab('LinRegWhole Posterior') +
  ylim(0,1500) + 
  xlim(0,1500) +
  coord_equal() +
  geom_abline(slope = 1, intercept=0, linetype = 'dotted') +
  theme(panel.border = element_blank(),
        panel.background = element_rect(fill= NA),
        axis.line = element_line(colour = "black"),
        axis.title.x = element_text(vjust = 0, size = 20),
        axis.title.y = element_text(vjust = 1, size = 20),
        axis.text.x = element_text(size=20),
        axis.text.y  = element_text(size=20),
        plot.title = element_text(size = 12),
        legend.text = element_text(size = 20),
        legend.title = element_text(size = 20),
        strip.text.x = element_text(size = 12),
        strip.text.y = element_text(size = 12))
dev.off()

######### ######### ######### DV Promoters SCATTERPLOT
DV_Promoter_windows = MergedALL %>%
  filter(Type %in% c("Promoter", "NA")) %>%
  filter(Location %in% c("NA", 'Dorsal', "Ventral"))
DV_Promoter_windows$Location <- factor(DV_Promoter_windows$Location,
                                       levels = c("NA", 'Dorsal', 'Ventral') ,ordered = TRUE)

png('020118_PE_2reps_nodups_AllRegions1KB_DVPromoters_linRegWhole_Scatter_0118newGeneList.png', width = 4000, height = 2000, units = "px",  res=300) 
ggplot(DV_Promoter_windows,
       aes(x = as.numeric(Ant),y= as.numeric(LinregPost), colour = as.factor(Location))) +
  scale_color_manual(name = 'Legand', values=c( "grey70", "orchid4", "seagreen4")) +
  geom_point(alpha = 0.9, aes(size = Dotsize)) +
  scale_size_continuous(range = c(1, 2)) +
  ggtitle('020118_PE_2reps_nodups_AllRegions1KB_DVPromoters_linRegWhole_Scatter_0118newGeneList') +
  xlab('LinregWhole Anterior') +
  ylab('LinRegWhole Posterior') +
  ylim(0,1500) + 
  xlim(0,1500) +
  coord_equal() +
  geom_abline(slope = 1, intercept=0, linetype = 'dotted') +
  theme(panel.border = element_blank(),
        panel.background = element_rect(fill= NA),
        axis.line = element_line(colour = "black"),
        axis.title.x = element_text(vjust = 0, size = 20),
        axis.title.y = element_text(vjust = 1, size = 20),
        axis.text.x = element_text(size=20),
        axis.text.y  = element_text(size=20),
        plot.title = element_text(size = 20),
        legend.text = element_text(size = 20),
        legend.title = element_text(size = 20),
        strip.text.x = element_text(size = 20),
        strip.text.y = element_text(size = 20))
dev.off()
```

#### Figure 3D, 4D, Supp Fig 4¶

In [ ]:

```
######### ######### ######### AP Enhancer Bargraphs 
#########  Filter out A vs P enhancers to calculate the positional score

allSamples_yes_peaks_ATACSkew = allSamples %>%
  filter(X011617.use == "yes") %>%
  filter(is.na(NoPeak) == TRUE) %>%
  select(chr, start, end, X011718_MERGED_2reps_081817_RemDUP_041217_Bowtie2_ME_JH_112315_10whole_shifted_lessthan130_10MNorm, X011718_MERGED_2reps_081817_RemDUP_041217_Bowtie2_ME_JH_112315_20Ant_shifted_lessthan130_10MNorm_linregwhole, X011718_MERGED_2reps_081817_RemDUP_041217_Bowtie2_ME_JH_112315_20Post_shifted_lessthan130_10MNorm_linregwhole, X011617_081817_RemDUP_041217_Bowtie2_ME_JH_112315_1109.ATACSlice03.03.1plus2_shifted_lessthan130_1MNorm, X011818_100417_BDTNP_DNaseI_stage5_ALLREPSCOMBINED_shifted_10MNorm, Name,New.Location.Assignment, Type, ATACSkewScore) %>%
  mutate(Dotsize = 1.5)
colnames(allSamples_yes_peaks_ATACSkew) = c("Chr", "Start", "End", "Whole", "Ant", "LinregPost", "AplusP", "Dnase1", "Name", "Location", "Type", "ATACSkewScore", "Dotsize")


JustAEnhancers = allSamples_yes_peaks_ATACSkew %>%
  select(Chr, Start, End, Location, Type, Name, Ant, LinregPost, ATACSkewScore) %>%
  filter(Location %in%  c('Anterior')) %>%
  filter(Type == 'Enhancer') %>%
  mutate(PositionalScore = (as.numeric(Ant) - as.numeric(LinregPost)) / (as.numeric(Ant) + as.numeric(LinregPost))) %>%
  distinct()

JustPEnhancers = allSamples_yes_peaks_ATACSkew %>%
  select(Chr, Start, End, Location, Type, Name, Ant, LinregPost, ATACSkewScore) %>%
  filter(Location %in%  c('Posterior', 'Mostly Post')) %>%
  filter(Type == 'Enhancer') %>%
  mutate(PositionalScore = (as.numeric(Ant) - as.numeric(LinregPost)) / (as.numeric(Ant) + as.numeric(LinregPost))) %>%
  distinct()

JustAPEnhancers = bind_rows(JustAEnhancers, JustPEnhancers)

write.csv(JustAPEnhancers, "020318_2Reps_NewGenelist_PE_Analysis_APEnhancers_PositionalScore.csv")

JustAPEnhancers$Location <- factor(JustAPEnhancers$Location,
                                 levels = c('Anterior', 'Posterior', 'Mostly Post'),ordered = TRUE)
ggplot(JustAPEnhancers,
       aes(x = reorder(as.factor(Name),- as.numeric(PositionalScore)),
           y = as.numeric(PositionalScore),
           fill = Location)) +
  geom_col(position = "stack", colour = "black") +
  ylim(-0.6, 0.6) +
  xlab("A-P Enhancers") +
  ylab("PositionalScore") +
  ggtitle("020118_PE_2reps_nodups_LinRegWhole_AP_Enhancers_PositionalScore_Bargraph_Position_0118newgeneList") +
  scale_fill_manual(name = "Legand", values=c("darkorange1", "dodgerblue3", "dodgerblue3")) +
  theme(panel.border = element_blank(),
        panel.background = element_rect(fill= NA),
        axis.line = element_line(colour = "black"),
        axis.title.x = element_text(vjust = 0, size = 15),
        axis.title.y = element_text(vjust = 1, size = 15),
        axis.text.x = element_text(size = 7, angle = -90),
        axis.text.y  = element_text(size= 15),
        plot.title = element_text(size = 15),
        legend.text = element_text(size = 15),
        legend.title = element_text(size = 15),
        strip.text.x = element_text(size = 12),
        strip.text.y = element_text(size = 12)) +
  png('020118_PE_2reps_nodups_LinRegWhole_AP_Enhancers_PositionalScore_Bargraph_Position_0118newgeneList.png', width = 5000, height = 2000, units = "px",  res=300)
dev.off()


######### ######### ######### AP Promoters Bargraphs 
#########  Filter out A vs P enhancers to calculate the positional score

JustAPromoters = allSamples_yes_peaks_ATACSkew %>%
  select(Location, Type, Name, Ant, LinregPost, ATACSkewScore) %>%
  filter(Location %in%  c('Anterior', 'Mostly Ant')) %>%
  filter(Type == 'Promoter') %>%
  mutate(PositionalScore = (as.numeric(Ant) - as.numeric(LinregPost)) / (as.numeric(Ant) + as.numeric(LinregPost))) %>%
  distinct()

JustPPromoters = allSamples_yes_peaks_ATACSkew %>%
  select(Location, Type, Name, Ant, LinregPost, ATACSkewScore) %>%
  filter(Location %in%  c('Posterior', 'Mostly Post')) %>%
  filter(Type == 'Promoter') %>%
  mutate(PositionalScore = (as.numeric(Ant) - as.numeric(LinregPost)) / (as.numeric(Ant) + as.numeric(LinregPost))) %>%
  distinct()

JustAPPromoters = bind_rows(JustAPromoters, JustPPromoters)

JustAPPromoters$Location <- factor(JustAPPromoters$Location,
                                   levels = c('Anterior', 'Mostly Ant', 'Posterior', 'Mostly Post'),ordered = TRUE)

png('020118_2reps_nodups_AP_Promoters_PositionalScore_Bargraph_Position_0118newgeneList.png', width = 5000, height = 2000, units = "px",  res=300)
ggplot(JustAPPromoters,
       aes(x = reorder(as.factor(Name),- as.numeric(PositionalScore)),
           y = as.numeric(PositionalScore),
           fill = Location)) +
  geom_col(position = "stack", colour = "black") +
  ylim(-0.6, 0.6) +
  xlab("A-P Promoters") +
  ylab("PositionalScore") +
  ggtitle("020118_2reps_nodups_AP_Promoters_PositionalScore_Bargraph_Position_0118newgeneList") +
  scale_fill_manual(name = "Legand", values=c("darkorange1", "darkorange1", "dodgerblue3", "dodgerblue3")) +
  theme(panel.border = element_blank(),
        panel.background = element_rect(fill= NA),
        axis.line = element_line(colour = "black"),
        axis.title.x = element_text(vjust = 0, size = 15),
        axis.title.y = element_text(vjust = 1, size = 15),
        axis.text.x = element_text(size = 7, angle = -90),
        axis.text.y  = element_text(size= 15),
        plot.title = element_text(size = 15),
        legend.text = element_text(size = 15),
        legend.title = element_text(size = 15),
        strip.text.x = element_text(size = 12),
        strip.text.y = element_text(size = 12))

dev.off()

######### ######### ######### DV Enhancer Bargraphs 
JustDorsalEnhancers = allSamples_yes_peaks_ATACSkew %>%
  select(Location, Type, Name, Ant, LinregPost, ATACSkewScore) %>%
  filter(Location == "Dorsal") %>%
  filter(Type == 'Enhancer') %>%
  mutate(PositionalScore = (as.numeric(Ant) - as.numeric(LinregPost)) / (as.numeric(Ant) + as.numeric(LinregPost))) %>%
  distinct()

JustVentralEnhancers = allSamples_yes_peaks_ATACSkew %>%
  select(Location, Type, Name, Ant, LinregPost, ATACSkewScore) %>%
  filter(Location == "Ventral") %>%
  filter(Type == 'Enhancer') %>%
  mutate(PositionalScore = (as.numeric(Ant) - as.numeric(LinregPost)) / (as.numeric(Ant) + as.numeric(LinregPost))) %>%
  distinct()

JustDVEnhancers = bind_rows(JustDorsalEnhancers, JustVentralEnhancers)

JustDVEnhancers$Location <- factor(JustDVEnhancers$Location,
                                   levels = c("Dorsal", "Ventral"),ordered = TRUE)

ggplot(JustDVEnhancers,
       aes(x = reorder(as.factor(Name),- as.numeric(PositionalScore)),
           y = as.numeric(PositionalScore),
           fill = Location)) +
  geom_col(position = "stack", colour = "black") +
  ylim(-0.6, 0.6) +
  xlab("D-V Enhancers") +
  ylab("PositionalScore") +
  ggtitle("020118_PE_2reps_nodups_LinRegWhole_DV_Enhancers_PositionalScore_Bargraph_Position_0118newgeneList") +
  scale_fill_manual(name = "Legand", values=c("orchid4", "seagreen4")) +
  theme(panel.border = element_blank(),
        panel.background = element_rect(fill= NA),
        axis.line = element_line(colour = "black"),
        axis.title.x = element_text(vjust = 0, size = 15),
        axis.title.y = element_text(vjust = 1, size = 15),
        axis.text.x = element_text(size = 7, angle = -90),
        axis.text.y  = element_text(size= 15),
        plot.title = element_text(size = 15),
        legend.text = element_text(size = 15),
        legend.title = element_text(size = 15),
        strip.text.x = element_text(size = 12),
        strip.text.y = element_text(size = 12)) +
  png('020118_PE_2reps_nodups_LinRegWhole_DV_Enhancers_PositionalScore_Bargraph_Position_0118newgeneList.png', width = 5000, height = 2000, units = "px",  res=300)
dev.off()

######### ######### ######### DV Promoter Bargraphs 

JustDorsalPromoter = allSamples_yes_peaks_ATACSkew %>%
  select(Location, Type, Name, Ant, LinregPost, ATACSkewScore) %>%
  filter(Location == "Dorsal") %>%
  filter(Type == 'Promoter') %>%
  mutate(PositionalScore = (as.numeric(Ant) - as.numeric(LinregPost)) / (as.numeric(Ant) + as.numeric(LinregPost))) %>%
  distinct()

JustVentralPromoter = allSamples_yes_peaks_ATACSkew %>%
  select(Location, Type, Name, Ant, LinregPost, ATACSkewScore) %>%
  filter(Location == "Ventral") %>%
  filter(Type == 'Promoter') %>%
  mutate(PositionalScore = (as.numeric(Ant) - as.numeric(LinregPost)) / (as.numeric(Ant) + as.numeric(LinregPost))) %>%
  distinct()

JustDVPromoter = bind_rows(JustDorsalPromoter, JustVentralPromoter)

JustDVPromoter$Location <- factor(JustDVPromoter$Location,
                                  levels = c("Dorsal", "Ventral"),ordered = TRUE)

ggplot(JustDVPromoter,
       aes(x = reorder(as.factor(Name),- as.numeric(PositionalScore)),
           y = as.numeric(PositionalScore),
           fill = Location)) +
  #guides(fill= FALSE) +
  geom_col(position = "stack", colour = "black") +
  ylim(-0.6, 0.6) +
  xlab("D-V Promoters") +
  ylab("PositionalScore") +
  ggtitle("020118_2reps_nodups_LinRegWhole_DV_Promoters_PositionalScore_Bargraph_Position_0118newgeneList") +
  scale_fill_manual(name = "Legand", values=c("orchid4", "seagreen4")) +
  theme(panel.border = element_blank(),
        panel.background = element_rect(fill= NA),
        axis.line = element_line(colour = "black"),
        axis.title.x = element_text(vjust = 0, size = 15),
        axis.title.y = element_text(vjust = 1, size = 15),
        axis.text.x = element_text(size = 7, angle = -90),
        axis.text.y  = element_text(size= 15),
        plot.title = element_text(size = 15),
        legend.text = element_text(size = 15),
        legend.title = element_text(size = 15),
        strip.text.x = element_text(size = 12),
        strip.text.y = element_text(size = 12)) +
  png('020118_2reps_nodups_LinRegWhole_DV_Promoters_PositionalScore_Bargraph_Position_0118newgeneList.png', width = 5000, height = 2000, units = "px",  res=300)
dev.off()
```

#### Figure 3C¶

In [ ]:

```
allSamples$Sample <- seq.int(nrow(allSamples)) # Add an sampleID row at the end

RandomRegions = allSamples %>%
  select(Sample, X011718_MERGED_2reps_081817_RemDUP_041217_Bowtie2_ME_JH_112315_20Ant_shifted_lessthan130_10MNorm_linregwhole.1, X011718_MERGED_2reps_081817_RemDUP_041217_Bowtie2_ME_JH_112315_20Post_shifted_lessthan130_10MNorm_linregwhole.1, New.Location.Assignment, Type, RandSkewScore) %>%
  filter(Type =="Enhancer") %>%
  mutate(Location.real = "Random") %>%
  mutate(Type.real = "Random") %>%
  mutate(TotalScore = as.numeric(X011718_MERGED_2reps_081817_RemDUP_041217_Bowtie2_ME_JH_112315_20Ant_shifted_lessthan130_10MNorm_linregwhole.1) + as.numeric(X011718_MERGED_2reps_081817_RemDUP_041217_Bowtie2_ME_JH_112315_20Post_shifted_lessthan130_10MNorm_linregwhole.1)) %>%
  select(Sample, Location.real, Type.real, RandSkewScore, TotalScore)
colnames(RandomRegions) = c("Name", "Location", "Type", "ATACSkewScore", "TotalScore")
RandomRegions$Name = as.character(RandomRegions$Name)

RandomRegions$ATACSkewScore = as.numeric(RandomRegions$ATACSkewScore)

JustAEnhancers_merged = JustAEnhancers %>%
  filter(Location %in%  c('Anterior')) %>%
  filter(Type == 'Enhancer') %>%
  mutate(TotalScore = as.numeric(Ant) + as.numeric(LinregPost)) %>%
  mutate(merged_location = "Anterior") %>%
  select(Name, merged_location, Type, PositionalScore, TotalScore) %>%
  distinct()


JustPEnhancers_merged = JustPEnhancers %>%
  filter(Location %in%  c('Posterior', 'Mostly Post')) %>%
  filter(Type == 'Enhancer') %>%
  mutate(TotalScore = as.numeric(Ant) + as.numeric(LinregPost)) %>%
  mutate(merged_location = "Posterior") %>%
  select(Name, merged_location, Type, PositionalScore, TotalScore) %>%
  distinct()

JustDorsalEnhancers_merged = allSamples_yes_peaks_ATACSkew %>%
  filter(Location == "Dorsal") %>%
  filter(Type == 'Enhancer') %>%
  mutate(TotalScore = as.numeric(Ant) + as.numeric(LinregPost)) %>%
  mutate(merged_location = "Dorsal") %>%
  select(Name, merged_location, Type, ATACSkewScore, TotalScore) %>%
  distinct()
JustDorsalEnhancers_merged$ATACSkewScore = as.numeric(JustDorsalEnhancers_merged$ATACSkewScore)

JustVentralEnhancers_merged = allSamples_yes_peaks_ATACSkew %>%
  filter(Location == "Ventral") %>%
  filter(Type == 'Enhancer') %>%
  mutate(TotalScore = as.numeric(Ant) + as.numeric(LinregPost)) %>%
  mutate(merged_location = "Ventral") %>%
  select(Name, merged_location, Type, ATACSkewScore, TotalScore) %>%
  distinct()
JustVentralEnhancers_merged$ATACSkewScore = as.numeric(JustVentralEnhancers_merged$ATACSkewScore)

colnames(JustAEnhancers_merged) = c("Name", "Location", "Type", "ATACSkewScore", "TotalScore")
colnames(JustPEnhancers_merged  ) = c("Name", "Location", "Type", "ATACSkewScore", "TotalScore")
colnames(JustDorsalEnhancers_merged) = c("Name", "Location", "Type", "ATACSkewScore", "TotalScore")
colnames(JustVentralEnhancers_merged) = c("Name", "Location", "Type", "ATACSkewScore", "TotalScore")

rand_and_Enhancers = bind_rows(RandomRegions, JustAEnhancers_merged, JustPEnhancers_merged, JustDorsalEnhancers_merged, JustVentralEnhancers_merged)
rand_and_Enhancers$Location <- factor(rand_and_Enhancers$Location,
                                      levels = c('Anterior',  'Posterior', 'Dorsal', 'Ventral', 'Random') ,ordered = TRUE)


ggplot(rand_and_Enhancers,
       aes(x = as.factor(Location),
           y = as.numeric(ATACSkewScore),
           fill = Location)) +
  #guides(fill= FALSE) +
  geom_boxplot(outlier.size = 0) +
  geom_dotplot(binaxis='y', stackdir='center', dotsize = 0.2 , alpha = 0.3, binpositions="all") +
  xlab("Location") +
  ylab("Positional Skew Score") +
  coord_cartesian(ylim = c(-0.5, 0.7)) +
  geom_abline(slope = 0, intercept=0, linetype = 'dotted') +
  ggtitle("020118_PE_2reps_nodups_LinRegWhole_AllLocations_Enhancers_ATACSkewScore_WithRandom_0118newgeneList") +
  scale_fill_manual(values=c("darkorange1", "dodgerblue3", "orchid4", "seagreen4", "grey50")) +
  theme(panel.border = element_blank(),
        panel.background = element_rect(fill= NA),
        axis.line = element_line(colour = "black"),
        axis.title.x = element_text(vjust = 0, size = 20),
        axis.title.y = element_text(vjust = 1, size = 20),
        axis.text.x = element_text(size=20),
        axis.text.y  = element_text(size=20),
        plot.title = element_text(size = 12),
        legend.text = element_text(size = 20),
        legend.title = element_text(size = 20),
        strip.text.x = element_text(size = 12),
        strip.text.y = element_text(size = 12),
        legend.position="none") +
  png('020118_PE_2reps_nodups_LinRegWhole_AllLocations_Enhancers_ATACSkewScore_WithRandom_0118newgeneList.png', width = 2000, height = 2000, units = "px",  res=300)
dev.off()

################# ANOVAS for Enhancers  ################# 
Anova_Enhancers_dataframe = rand_and_Enhancers %>%
  select(Location, ATACSkewScore)

Anova_Enhancers_lm = lm(as.numeric(Anova_Enhancers_dataframe$ATACSkewScore) ~ Anova_Enhancers_dataframe$Location)
Anova_Enhancer = aov(formula = as.numeric(Anova_Enhancers_dataframe$ATACSkewScore) ~ Anova_Enhancers_dataframe$Location)
Tukey_Enhancers = TukeyHSD(Anova_Enhancer)

write.table(Tukey_Enhancers$`Anova_Enhancers_dataframe$Location`, "020118_Anova_ALLLocations_Enhancers.txt")
```

#### Figure 4C¶

In [ ]:

```
Susan_RNAseq_data <- read.delim2("~/Box Sync/Eisen_Lab/Experiments/ATAC-seq/Halves/ATAC-seq_Pools/040617_Analysis/061417_BrowserTraces/062317_RegressionNormalization/081617_2reps/Lott2011_RNA-seq_GeneExpression.txt", sep = "\t", stringsAsFactors = FALSE)
colnames(Susan_RNAseq_data)[1] = "Name"
Joined_ATACPeaks_plus_RNAseq = full_join(allSamples, Susan_RNAseq_data, by = "Name")

JustPromoters = Joined_ATACPeaks_plus_RNAseq %>%
  filter(Type == "Promoter") %>%
  select(Name, Type, New.Location.Assignment,X011718_MERGED_2reps_081817_RemDUP_041217_Bowtie2_ME_JH_112315_20Ant_shifted_lessthan130_10MNorm_linregwhole ,X011718_MERGED_2reps_081817_RemDUP_041217_Bowtie2_ME_JH_112315_20Post_shifted_lessthan130_10MNorm_linregwhole , ATACSkewScore, PValue, WholePeaks, AntPeaks, PostPeaks, NoPeak, CLASS, F10, F11, F12, U13, F14A, U14A, F14B, F14B_r2, F14C, F14C_r2, F14D, U14D)

write.csv(JustPromoters, file = "020118_Promoters_peaks_RNAseq.csv")

JustAProm_merged_ZYG = JustAProm_merged %>%
  filter(CLASS == "zyg")

JustP_prom_merged_ZYG = JustPProm_merged %>%
  filter(CLASS == "zyg")

JustDorsal_Prom_merged_ZYG = JustDorsal_Prom_merged %>%
  filter(CLASS == "zyg")

JustVentral_Prom_merged_ZYG = JustVentral_Prom_merged %>%
  filter(CLASS == "zyg")


rand_and_PromotersZYG = bind_rows(RandomRegions, JustAProm_merged_ZYG, JustP_prom_merged_ZYG, JustDorsal_Prom_merged_ZYG, JustVentral_Prom_merged_ZYG)
rand_and_PromotersZYG$Location <- factor(rand_and_PromotersZYG$Location,
                                         levels = c('Anterior', 'Posterior', 'Dorsal', 'Ventral', 'Random') ,ordered = TRUE)

ggplot(rand_and_PromotersZYG,
       aes(x = as.factor(Location),
           y = as.numeric(ATACSkewScore),
           fill = Location)) +
  #guides(fill= FALSE) +
  geom_boxplot(outlier.size = 0) +
  geom_dotplot(binaxis='y', stackdir='center', dotsize = 0.2 , alpha = 0.3, binpositions="all") +
  xlab("Location") +
  ylab("Positional Skew Score") +
  coord_cartesian(ylim = c(-0.5, 0.7)) +
  geom_abline(slope = 0, intercept=0, linetype = 'dotted') +
  ggtitle("020118_PE_2reps_nodups_LinRegWhole_ALLLocations_Promoters_ATACSkewScore_WithRandom_0118newgeneList_Zyggenesonly") +
  scale_fill_manual(values=c("darkorange1", "dodgerblue3", "orchid4", "seagreen4", "grey50")) +
  theme(panel.border = element_blank(),
        panel.background = element_rect(fill= NA),
        axis.line = element_line(colour = "black"),
        axis.title.x = element_text(vjust = 0, size = 20),
        axis.title.y = element_text(vjust = 1, size = 20),
        axis.text.x = element_text(size=20),
        axis.text.y  = element_text(size=20),
        plot.title = element_text(size = 12),
        legend.text = element_text(size = 20),
        legend.title = element_text(size = 20),
        strip.text.x = element_text(size = 12),
        strip.text.y = element_text(size = 12),
        legend.position="none") +
  png('020118_PE_2reps_nodups_LinRegWhole_ALLLocations_Promoters_ATACSkewScore_WithRandom_0118newgeneList_Zyggenesonly.png', width = 2000, height = 2000, units = "px",  res=300)
dev.off()

################# ANOVAS for BoxPlot Zyg only promoters  ################# 
Anova_Promoters_dataframe = rand_and_PromotersZYG %>%
  select(Location, ATACSkewScore)

Anova_Promoters_lm = lm(as.numeric(Anova_Promoters_dataframe$ATACSkewScore) ~ Anova_Promoters_dataframe$Location)
Anova_Promoter = aov(formula = as.numeric(Anova_Promoters_dataframe$ATACSkewScore) ~ Anova_Promoters_dataframe$Location)
Tukey_Promoters = TukeyHSD(Anova_Promoter)
write.table(Tukey_Promoters$`Anova_Promoters_dataframe$Location`, "020118_Anova_ALLLocations_Promoters_Zygonly.txt")
```

#### Supplemental Figure¶

In [ ]:

```
################################################################################################
################################################################################################
###### Positional score replicates                                                        ###### 
################################################################################################

#first I need to calculate Positional score for all the replicates

RepSamples_NoNAs = allSamples %>%
  select(chr, start, end, Type, New.Location.Assignment, Name, X011617.use, ATACSkewScore, NoPeak, X011617_101117_RemDUP_PE_041217_Bowtie2_ME_JH_112315_1105.ATACSlice2.01.20a_shifted_lessthan130_1MNorm, X011617_081817_RemDUP_041217_Bowtie2_ME_JH_112315_1109.ATACSlice03.01.20Ant_shifted_lessthan130_1MNorm, X011617_081817_RemDUP_041217_Bowtie2_ME_JH_112315_1105.ATACSlice2.02.20p_shifted_lessthan130_1MNorm,  X011617_081817_RemDUP_041217_Bowtie2_ME_JH_112315_1109.ATACSlice03.02.20Post_shifted_lessthan130_1MNorm, X011617_081817_RemDUP_041217_Bowtie2_ME_JH_112315_1105.ATACSlice2.7.10whole_shifted_lessthan130_1MNorm, X011617_081817_RemDUP_041217_Bowtie2_ME_JH_112315_1109.ATACSlice03.04.10whole_shifted_lessthan130_1MNorm) %>%
  filter(X011617.use == "yes") %>%
  filter(is.na(NoPeak) == TRUE) %>%
  mutate(PositionalScore_1105 = (as.numeric(X011617_101117_RemDUP_PE_041217_Bowtie2_ME_JH_112315_1105.ATACSlice2.01.20a_shifted_lessthan130_1MNorm) - as.numeric(X011617_081817_RemDUP_041217_Bowtie2_ME_JH_112315_1105.ATACSlice2.02.20p_shifted_lessthan130_1MNorm)) / (as.numeric(X011617_101117_RemDUP_PE_041217_Bowtie2_ME_JH_112315_1105.ATACSlice2.01.20a_shifted_lessthan130_1MNorm) + as.numeric(X011617_081817_RemDUP_041217_Bowtie2_ME_JH_112315_1105.ATACSlice2.02.20p_shifted_lessthan130_1MNorm))) %>%
  mutate(PositionalScore_1109 = (as.numeric(X011617_081817_RemDUP_041217_Bowtie2_ME_JH_112315_1109.ATACSlice03.01.20Ant_shifted_lessthan130_1MNorm) - as.numeric(X011617_081817_RemDUP_041217_Bowtie2_ME_JH_112315_1109.ATACSlice03.02.20Post_shifted_lessthan130_1MNorm)) / (as.numeric(X011617_081817_RemDUP_041217_Bowtie2_ME_JH_112315_1109.ATACSlice03.01.20Ant_shifted_lessthan130_1MNorm) + as.numeric(X011617_081817_RemDUP_041217_Bowtie2_ME_JH_112315_1109.ATACSlice03.02.20Post_shifted_lessthan130_1MNorm))) %>%
  select(Name, PositionalScore_1105, PositionalScore_1109)

# Then add it back to the positional score data frame used in the bargraph
JustAEnhancers_Reps = inner_join(JustAEnhancers, RepSamples_NoNAs, by = "Name")
JustPEnhancers_Reps = inner_join(JustPEnhancers, RepSamples_NoNAs, by = "Name")

JustAPEnhancers_Reps = bind_rows(JustAEnhancers_Reps, JustPEnhancers_Reps)

JustAPEnhancers_Reps$Location <- factor(JustAPEnhancers_Reps$Location,
                                   levels = c('Anterior', 'Posterior', 'Mostly Post'),ordered = TRUE)

ggplot(JustAPEnhancers_Reps,
       aes(x = reorder(as.factor(Name),- as.numeric(PositionalScore)),
           y = as.numeric(PositionalScore),
           fill = Location)) +
  geom_col(position = "stack", alpha = 0.2) +
  geom_boxplot(outlier.size = 0) +
  ylim(-0.8, 0.8) +
  xlab("A-P Enhancers") +
  ylab("PositionalScore") +
  geom_dotplot(aes(x = reorder(as.factor(Name),- as.numeric(PositionalScore)), y = as.numeric(PositionalScore_1105)) ,binaxis='y', stackdir='center', dotsize = 0.7 , alpha = 1, binpositions="all") +
  geom_dotplot(aes(x = reorder(as.factor(Name),- as.numeric(PositionalScore)), y = as.numeric(PositionalScore_1109)) ,binaxis='y', stackdir='center', dotsize = 0.7 , alpha = 1, binpositions="all") +
  geom_hline(yintercept = 0) +
  ggtitle("020118_PE_2reps_nodups_LinRegWhole_AP_Enhancers_PositionalScore_Bargraph_Position_0118newgeneList_REPLICATES") +
  scale_fill_manual(name = "Legand", values=c("darkorange1", "dodgerblue3", "dodgerblue3")) +
  theme(panel.border = element_blank(),
        panel.background = element_rect(fill= NA),
        axis.line = element_line(colour = "black"),
        axis.title.x = element_text(vjust = 0, size = 15),
        axis.title.y = element_text(vjust = 1, size = 15),
        axis.text.x = element_text(size = 7, angle = -90),
        axis.text.y  = element_text(size= 15),
        plot.title = element_text(size = 15),
        legend.text = element_text(size = 15),
        legend.title = element_text(size = 15),
        strip.text.x = element_text(size = 12),
        strip.text.y = element_text(size = 12)) +
  png('020118_PE_2reps_nodups_LinRegWhole_AP_Enhancers_PositionalScore_Bargraph_Position_0118newgeneListREPLICATES.png', width = 5000, height = 2000, units = "px",  res=300)
dev.off()
```

#### S2 Figure¶

In [ ]:

```
######### ######### ######### Ant

### Correction new graph for figure 1 that is just AvsP
AvsP_cor = cor(as.numeric(allSamples_NoNAs$Ant),y= as.numeric(allSamples_NoNAs$LinregPost), method = "spearman")
AvsP_cor_Pearson = cor(as.numeric(allSamples_NoNAs$Ant),y= as.numeric(allSamples_NoNAs$LinregPost), method = "pearson")
AvsP_cor_rsq = (AvsP_cor)^2
AvsP_cor_Pearson_rsq = (AvsP_cor_Pearson)^2


png('020118_PE2reps_nodups_LinRegWhole_AllRegions1KB_AvsP_log.png', width = 4000, height = 2000, units = "px",  res=300) 
ggplot(allSamples_NoNAs,
       aes(x = as.numeric(LinregPost),y= as.numeric(Ant), colour = as.factor(Location))) +
  scale_color_manual(values=c("lightsteelblue4")) +
  #scale_size_continuous(range = c(1, 2)) +
  geom_point(alpha = 0.05, size = 0.3, show.legend = FALSE) +
  geom_density2d(colour="lightskyblue2", alpha = 0.5, size = 0.4) + 
  ggtitle('020118_PE_2reps_nodups_LinRegWhole_AllRegions1KB_AvsP') +
  scale_x_log10(limits = c(10,1500)) +
  scale_y_log10(limits = c(10,1500)) +
  xlab('Reg Norm Whole Posterior') +
  ylab('Reg Norm Whole Anterior') +
  #geom_smooth(method='lm', formula = y~x) +
  #ylim(0,1500) + 
  #xlim(0,1500) +
  coord_equal() +
  geom_abline(slope = 1, intercept=0, linetype = 'dotted') +
  annotate("text", x=1000, y=20, label= AvsP_cor) +
  #annotate("text", x=500, y=1500, label= Post_Prom_Label, colour = 'dodgerblue3') +
  theme(panel.border = element_blank(),
        panel.background = element_rect(fill= NA),
        axis.line = element_line(colour = "black"),
        axis.title.x = element_text(vjust = 0, size = 20),
        axis.title.y = element_text(vjust = 1, size = 20),
        axis.text.x = element_text(size=20),
        axis.text.y  = element_text(size=20),
        plot.title = element_text(size = 12),
        legend.text = element_text(size = 20),
        legend.title = element_text(size = 20),
        strip.text.x = element_text(size = 12),
        strip.text.y = element_text(size = 12))
dev.off()


png('020118_PE2reps_nodups_LinRegWhole_AllRegions1KB_AvsP_nolog.png', width = 4000, height = 2000, units = "px",  res=300) 
ggplot(allSamples_NoNAs,
       aes(x = as.numeric(LinregPost),y= as.numeric(Ant), colour = as.factor(Location))) +
  scale_color_manual(values=c("lightsteelblue4")) +
  #scale_size_continuous(range = c(1, 2)) +
  geom_point(alpha = 0.05, size = 0.3, show.legend = FALSE) +
  geom_density2d(colour="lightskyblue2", alpha = 0.5, size = 0.4) + 
  ggtitle('020118_PE_2reps_nodups_LinRegWhole_AllRegions1KB_AvsP') +
  #scale_x_log10(limits = c(10,1500)) +
  #scale_y_log10(limits = c(10,1500)) +
  xlab('Reg Norm Whole Posterior') +
  ylab('Reg Norm Whole Anterior') +
  #geom_smooth(method='lm', formula = y~x) +
  ylim(0,1500) + 
  xlim(0,1500) +
  coord_equal() +
  geom_abline(slope = 1, intercept=0, linetype = 'dotted') +
  annotate("text", x=1000, y=20, label= AvsP_cor) +
  #annotate("text", x=500, y=1500, label= Post_Prom_Label, colour = 'dodgerblue3') +
  theme(panel.border = element_blank(),
        panel.background = element_rect(fill= NA),
        axis.line = element_line(colour = "black"),
        axis.title.x = element_text(vjust = 0, size = 20),
        axis.title.y = element_text(vjust = 1, size = 20),
        axis.text.x = element_text(size=20),
        axis.text.y  = element_text(size=20),
        plot.title = element_text(size = 12),
        legend.text = element_text(size = 20),
        legend.title = element_text(size = 20),
        strip.text.x = element_text(size = 12),
        strip.text.y = element_text(size = 12))
dev.off()


AvsWhole_cor = cor(as.numeric(allSamples_NoNAs$Ant),y= as.numeric(allSamples_NoNAs$Whole), method = "spearman")
AvsWhole_cor_Pearson = cor(as.numeric(allSamples_NoNAs$Ant),y= as.numeric(allSamples_NoNAs$Whole), method = "pearson")
AvsWhole_cor_RSQ = (AvsWhole_cor)^2
AvsWhole_cor_Pearson_RSQ = (AvsWhole_cor_Pearson)^2


png('020118_PE_2reps_nodups_LinRegWhole_AvsWhole_nolog.png', width = 4000, height = 2000, units = "px",  res=300) 
ggplot(allSamples_NoNAs,
       aes(x = as.numeric(Ant),y= as.numeric(Whole), colour = as.factor(Location))) +
  scale_color_manual(values=c("lightsteelblue4")) +
  geom_point(alpha = 0.05, size = 0.3, show.legend = FALSE) +
  #scale_size_continuous(range = c(1, 2)) +
  geom_density2d(colour="lightblue1", alpha = 0.5, size = 0.4) + 
  ggtitle('020118_PE_2reps_nodups_LinRegWhole_AvsWhole') +
  #scale_x_log10(limit = c(10,1500)) +
  #scale_y_log10(limit = c(10,1500)) +
  xlab('LinRegWhole Anterior') +
  ylab('LinRegWhole Whole') +
  #geom_smooth(method='lm', formula = y~x) +
  ylim(0,1500) + 
  xlim(0,1500) +
  coord_equal() +
  geom_abline(slope = 1, intercept=0, linetype = 'dotted') +
  #annotate("text", x=20, y=1000, label= AvsWhole_cor) +
  #annotate("text", x=500, y=1500, label= Post_Prom_Label, colour = 'dodgerblue3') +
  theme(panel.border = element_blank(),
        panel.background = element_rect(fill= NA),
        axis.line = element_line(colour = "black"),
        axis.title.x = element_text(vjust = 0, size = 20),
        axis.title.y = element_text(vjust = 1, size = 20),
        axis.text.x = element_text(size=20),
        axis.text.y  = element_text(size=20),
        plot.title = element_text(size = 12),
        legend.text = element_text(size = 20),
        legend.title = element_text(size = 20),
        strip.text.x = element_text(size = 12),
        strip.text.y = element_text(size = 12))
dev.off()


AvsAP_cor = cor(as.numeric(allSamples_NoNAs$Ant),y= as.numeric(allSamples_NoNAs$AplusP), method = "spearman")
AvsAP_cor_Pearson = cor(as.numeric(allSamples_NoNAs$Ant),y= as.numeric(allSamples_NoNAs$AplusP), method = "pearson")
AvsAP_cor_rq = (AvsAP_cor)^2
AvsAP_cor_Pearson_rq = (AvsAP_cor_Pearson)^2

png('020118_PE_2reps_nodups_LinRegWhole_AvsAP.png', width = 4000, height = 2000, units = "px",  res=300) 
ggplot(allSamples_NoNAs,
       aes(x = as.numeric(Ant),y= as.numeric(AplusP), colour = as.factor(Location))) +
  scale_color_manual(values=c("lightsteelblue4")) +
  geom_point(alpha = 0.4, size = 0.3, show.legend = FALSE) +
  geom_density2d(colour="lightblue1") + 
  ggtitle('020118_PE_2reps_nodups_LinRegWhole_AvsAP') +
  xlab('LinregWhole Anterior') +
  ylab('LinregWhole AplusP') +
  ylim(0,1500) + 
  xlim(0,1500) +
  coord_equal() +
  geom_abline(slope = 1, intercept=0, linetype = 'dotted') +
  annotate("text", x=1500, y=500, label= AvsAP_cor) +
  theme(panel.border = element_blank(),
        panel.background = element_rect(fill= NA),
        axis.line = element_line(colour = "black"),
        axis.title.x = element_text(vjust = 0, size = 20),
        axis.title.y = element_text(vjust = 1, size = 20),
        axis.text.x = element_text(size=20),
        axis.text.y  = element_text(size=20),
        plot.title = element_text(size = 12),
        legend.text = element_text(size = 20),
        legend.title = element_text(size = 20),
        strip.text.x = element_text(size = 12),
        strip.text.y = element_text(size = 12))
dev.off()

AvsDnaseI_cor = cor(as.numeric(allSamples_NoNAs$Ant),y= as.numeric(allSamples_NoNAs$Dnase1), method = "spearman")
AvsDnaseI_cor_Pearson = cor(as.numeric(allSamples_NoNAs$Ant),y= as.numeric(allSamples_NoNAs$Dnase1), method = "pearson")
AvsDnaseI_cor_rsq = (AvsDnaseI_cor)^2
AvsDnaseI_cor_Pearson_rq = (AvsDnaseI_cor_Pearson)^2
png('021218_PE_2reps_nodups_LinRegWhole_AvsDnaseI.png', width = 4000, height = 2000, units = "px",  res=300) 
ggplot(allSamples_NoNAs,
       aes(x = as.numeric(Dnase1),y= as.numeric(Ant), colour = as.factor(Location))) +
  scale_color_manual(values=c("lightsteelblue4")) +
  geom_point(alpha = 0.4, size = 0.3, show.legend = FALSE) +
  geom_density2d(colour="lightblue1") + 
  ggtitle('020118_PE_2reps_nodups_LinRegWhole_AvsDnaseI') +
  xlab('RegNormWhole DnaseI') +
  ylab('RegNormWhole Anterior') +
  ylim(0,1500) + 
  xlim(0,1500) +
  coord_equal() +
  geom_abline(slope = 1, intercept=0, linetype = 'dotted') +
  #annotate("text", x=1500, y=500, label= AvsDnaseI_cor) +
  theme(panel.border = element_blank(),
        panel.background = element_rect(fill= NA),
        axis.line = element_line(colour = "black"),
        axis.title.x = element_text(vjust = 0, size = 20),
        axis.title.y = element_text(vjust = 1, size = 20),
        axis.text.x = element_text(size=20),
        axis.text.y  = element_text(size=20),
        plot.title = element_text(size = 12),
        legend.text = element_text(size = 20),
        legend.title = element_text(size = 20),
        strip.text.x = element_text(size = 12),
        strip.text.y = element_text(size = 12))
dev.off()


######### ######### ######### Posterior

PvsWhole_cor = cor(as.numeric(allSamples_NoNAs$LinregPost),y= as.numeric(allSamples_NoNAs$Whole), method = "spearman")
PvsWhole_cor_Pearson = cor(as.numeric(allSamples_NoNAs$LinregPost),y= as.numeric(allSamples_NoNAs$Whole), method = "pearson")
PvsWhole_cor_rsq = (PvsWhole_cor)^2
PvsWhole_cor_Pearson_rsq = (PvsWhole_cor_Pearson)^2
png('020118_PE_2reps_nodups_LinRegWhole_PostvsWhole.png', width = 4000, height = 2000, units = "px",  res=300) 
ggplot(allSamples_NoNAs,
       aes(x = as.numeric(LinregPost),y= as.numeric(Whole), colour = as.factor(Location))) +
  scale_color_manual(values=c("lightsteelblue4")) +
  geom_point(alpha = 0.4, size = 0.3, show.legend = FALSE) +
  geom_density2d(colour="lightblue1") + 
  ggtitle('020118_PE_2reps_nodups_LinRegWhole_PostvsWhole') +
  xlab('LinregWhole Post') +
  ylab('Whole') +
  ylim(0,1500) + 
  xlim(0,1500) +
  coord_equal() +
  geom_abline(slope = 1, intercept=0, linetype = 'dotted') +
  annotate("text", x=1500, y=500, label= PvsWhole_cor) +
  theme(panel.border = element_blank(),
        panel.background = element_rect(fill= NA),
        axis.line = element_line(colour = "black"),
        axis.title.x = element_text(vjust = 0, size = 20),
        axis.title.y = element_text(vjust = 1, size = 20),
        axis.text.x = element_text(size=20),
        axis.text.y  = element_text(size=20),
        plot.title = element_text(size = 12),
        legend.text = element_text(size = 20),
        legend.title = element_text(size = 20),
        strip.text.x = element_text(size = 12),
        strip.text.y = element_text(size = 12))
dev.off()

PvsAP_cor = cor(as.numeric(allSamples_NoNAs$LinregPost),y= as.numeric(allSamples_NoNAs$AplusP), method = "spearman")
PvsAP_cor_Pearson = cor(as.numeric(allSamples_NoNAs$LinregPost),y= as.numeric(allSamples_NoNAs$AplusP), method = "pearson")
png('020118_PE_2reps_nodups_LinRegWhole_PostvsAplusP.png', width = 4000, height = 2000, units = "px",  res=300) 
ggplot(allSamples_NoNAs,
       aes(x = as.numeric(LinregPost),y= as.numeric(AplusP), colour = as.factor(Location))) +
  scale_color_manual(values=c("lightsteelblue4")) +
  geom_point(alpha = 0.4, size = 0.3, show.legend = FALSE) +
  geom_density2d(colour="lightblue1") + 
  ggtitle('020118_PE_2reps_nodups_LinRegWhole_PostvsAplusP') +
  xlab('LinregPost') +
  ylab('LinregWhole AplusP') +
  ylim(0,1500) + 
  xlim(0,1500) +
  coord_equal() +
  geom_abline(slope = 1, intercept=0, linetype = 'dotted') +
  annotate("text", x=1500, y=500, label= PvsAP_cor) +
  theme(panel.border = element_blank(),
        panel.background = element_rect(fill= NA),
        axis.line = element_line(colour = "black"),
        axis.title.x = element_text(vjust = 0, size = 20),
        axis.title.y = element_text(vjust = 1, size = 20),
        axis.text.x = element_text(size=20),
        axis.text.y  = element_text(size=20),
        plot.title = element_text(size = 12),
        legend.text = element_text(size = 20),
        legend.title = element_text(size = 20),
        strip.text.x = element_text(size = 12),
        strip.text.y = element_text(size = 12))
dev.off()

PvsDnaseI_cor = cor(as.numeric(allSamples_NoNAs$LinregPost),y= as.numeric(allSamples_NoNAs$Dnase1), method = "spearman")
PvsDnaseI_cor_Pearson = cor(as.numeric(allSamples_NoNAs$LinregPost),y= as.numeric(allSamples_NoNAs$Dnase1), method = "pearson")
PvsDnaseI_cor_rsq = (PvsDnaseI_cor)^2
PvsDnaseI_cor_Pearson_rsq = (PvsDnaseI_cor_Pearson)^2

png('021218_PE_2reps_nodups_LinRegWhole_PostvsDnaseI.png', width = 4000, height = 2000, units = "px",  res=300) 
ggplot(allSamples_NoNAs,
       aes(x = as.numeric(Dnase1),y= as.numeric(LinregPost), colour = as.factor(Location))) +
  scale_color_manual(values=c("lightsteelblue4")) +
  geom_point(alpha = 0.4, size = 0.3, show.legend = FALSE) +
  geom_density2d(colour="lightblue1") + 
  ggtitle('020118_PE_2reps_nodups_LinRegWhole_PostvsDnaseI') +
  xlab('DnaseI') +
  ylab('LinregWhole LinregPost') +
  ylim(0,1500) + 
  xlim(0,1500) +
  coord_equal() +
  geom_abline(slope = 1, intercept=0, linetype = 'dotted') +
  #annotate("text", x=1500, y=500, label= PvsDnaseI_cor) +
  theme(panel.border = element_blank(),
        panel.background = element_rect(fill= NA),
        axis.line = element_line(colour = "black"),
        axis.title.x = element_text(vjust = 0, size = 20),
        axis.title.y = element_text(vjust = 1, size = 20),
        axis.text.x = element_text(size=20),
        axis.text.y  = element_text(size=20),
        plot.title = element_text(size = 12),
        legend.text = element_text(size = 20),
        legend.title = element_text(size = 20),
        strip.text.x = element_text(size = 12),
        strip.text.y = element_text(size = 12))
dev.off()

######### ######### ######### Whole


WholevsDnaseI_cor = cor(as.numeric(allSamples_NoNAs$Whole),y= as.numeric(allSamples_NoNAs$Dnase1), method = "spearman")
WholevsDnaseI_cor_Pearson = cor(as.numeric(allSamples_NoNAs$Whole),y= as.numeric(allSamples_NoNAs$Dnase1), method = "pearson")
WholevsDnaseI_cor_rsq = (WholevsDnaseI_cor)^2
WholevsDnaseI_cor_Pearson_rsq = (WholevsDnaseI_cor_Pearson)^2

png('021218_PE_2reps_nodups_LinRegWhole_WholevsDnaseI.png', width = 4000, height = 2000, units = "px",  res=300) 
ggplot(allSamples_NoNAs,
       aes(x = as.numeric(Dnase1),y= as.numeric(Whole), colour = as.factor(Location))) +
  scale_color_manual(values=c("lightsteelblue4")) +
  geom_point(alpha = 0.4, size = 0.3, show.legend = FALSE) +
  geom_density2d(colour="lightblue1") + 
  ggtitle('020118_PE_2reps_nodups_LinRegWhole_WholevsDnaseI') +
  xlab('Dnase1') +
  ylab('Whole') +
  ylim(0,1500) + 
  xlim(0,1500) +
  coord_equal() +
  geom_abline(slope = 1, intercept=0, linetype = 'dotted') +
  #annotate("text", x=1500, y=500, label= WholevsDnaseI_cor) +
  theme(panel.border = element_blank(),
        panel.background = element_rect(fill= NA),
        axis.line = element_line(colour = "black"),
        axis.title.x = element_text(vjust = 0, size = 20),
        axis.title.y = element_text(vjust = 1, size = 20),
        axis.text.x = element_text(size=20),
        axis.text.y  = element_text(size=20),
        plot.title = element_text(size = 12),
        legend.text = element_text(size = 20),
        legend.title = element_text(size = 20),
        strip.text.x = element_text(size = 12),
        strip.text.y = element_text(size = 12))
dev.off()

WholevsAplusP_cor = cor(as.numeric(allSamples_NoNAs$Whole),y= as.numeric(allSamples_NoNAs$AplusP), method = "spearman")
WholevsAplusP_cor_Pearson = cor(as.numeric(allSamples_NoNAs$Whole),y= as.numeric(allSamples_NoNAs$AplusP), method = "pearson")
WholevsAplusP_cor_Pearson_rsq = (WholevsAplusP_cor_Pearson)^2
WholevsAplusP_cor_rsq = (WholevsAplusP_cor)^2
png('020118_PE_2reps_nodups_LinRegWhole_WholevsAplusP.png', width = 4000, height = 2000, units = "px",  res=300) 
ggplot(allSamples_NoNAs,
       aes(x = as.numeric(Whole),y= as.numeric(AplusP), colour = as.factor(Location))) +
  scale_color_manual(values=c("lightsteelblue4")) +
  geom_point(alpha = 0.4, size = 0.3, show.legend = FALSE) +
  geom_density2d(colour="lightblue1") + 
  ggtitle('020118_PE_2reps_nodups_LinRegWhole_WholevsAplusP') +
  xlab('Whole') +
  ylab('RegNormWhole AplusP') +
  ylim(0,1500) + 
  xlim(0,1500) +
  coord_equal() +
  geom_abline(slope = 1, intercept=0, linetype = 'dotted') +
  annotate("text", x=1500, y=500, label= WholevsAplusP_cor) +
  theme(panel.border = element_blank(),
        panel.background = element_rect(fill= NA),
        axis.line = element_line(colour = "black"),
        axis.title.x = element_text(vjust = 0, size = 20),
        axis.title.y = element_text(vjust = 1, size = 20),
        axis.text.x = element_text(size=20),
        axis.text.y  = element_text(size=20),
        plot.title = element_text(size = 12),
        legend.text = element_text(size = 20),
        legend.title = element_text(size = 20),
        strip.text.x = element_text(size = 12),
        strip.text.y = element_text(size = 12))
dev.off()

######### ######### ######### ######### ######### ######### ######### ######### ######### ######### ######### ######### ######### ######### #########
## Supplementary Figure 1 --> Replicate Correlation Graphs


RepSamples_NoNAs = allSamples_1kb_bins %>%
  select(X.chr., X.start., X.end., X011617_101117_RemDUP_PE_041217_Bowtie2_ME_JH_112315_1105.ATACSlice2.01.20a_shifted_lessthan130_1MNorm, X011617_081817_RemDUP_041217_Bowtie2_ME_JH_112315_1109.ATACSlice03.01.20Ant_shifted_lessthan130_1MNorm, X011617_081817_RemDUP_041217_Bowtie2_ME_JH_112315_1105.ATACSlice2.02.20p_shifted_lessthan130_1MNorm,  X011617_081817_RemDUP_041217_Bowtie2_ME_JH_112315_1109.ATACSlice03.02.20Post_shifted_lessthan130_1MNorm, X011617_081817_RemDUP_041217_Bowtie2_ME_JH_112315_1105.ATACSlice2.7.10whole_shifted_lessthan130_1MNorm, X011617_081817_RemDUP_041217_Bowtie2_ME_JH_112315_1109.ATACSlice03.04.10whole_shifted_lessthan130_1MNorm, X011818_100417_BDTNP_DNaseI_stage5_ALLREPSCOMBINED_shifted_10MNorm) %>%
  filter(X011617_101117_RemDUP_PE_041217_Bowtie2_ME_JH_112315_1105.ATACSlice2.01.20a_shifted_lessthan130_1MNorm != 'nan') %>%
  filter(X011617_081817_RemDUP_041217_Bowtie2_ME_JH_112315_1109.ATACSlice03.01.20Ant_shifted_lessthan130_1MNorm != 'nan') %>%
  filter(X011617_081817_RemDUP_041217_Bowtie2_ME_JH_112315_1105.ATACSlice2.02.20p_shifted_lessthan130_1MNorm != 'nan') %>%
  filter(X011617_081817_RemDUP_041217_Bowtie2_ME_JH_112315_1109.ATACSlice03.02.20Post_shifted_lessthan130_1MNorm != 'nan') %>%
  filter(X011617_081817_RemDUP_041217_Bowtie2_ME_JH_112315_1105.ATACSlice2.7.10whole_shifted_lessthan130_1MNorm != 'nan') %>%
  filter(X011617_081817_RemDUP_041217_Bowtie2_ME_JH_112315_1109.ATACSlice03.04.10whole_shifted_lessthan130_1MNorm != 'nan') %>%
  filter(X011818_100417_BDTNP_DNaseI_stage5_ALLREPSCOMBINED_shifted_10MNorm != 'nan')
colnames(RepSamples_NoNAs) = c("Chr", "Start", "End", "1105-20a", "1109-20a", "1105-20p", "1109-20p", "1105-10w", "1109-10w", "DnaseI")


#### Anterior replicate graph
AntReps_cor = cor(as.numeric(RepSamples_NoNAs$`1105-20a`),y= as.numeric(RepSamples_NoNAs$`1109-20a`), method = "spearman")
AntReps_cor_pearson = cor(as.numeric(RepSamples_NoNAs$`1105-20a`),y= as.numeric(RepSamples_NoNAs$`1109-20a`), method = "pearson")
AntReps_cor_r_squared = (AntReps_cor)^2
AntReps_cor_pearson_Squared = (AntReps_cor_pearson)^2
png('020118_2reps_nodups_Antreps_nolog.png', width = 4000, height = 2000, units = "px",  res=300) 
ggplot(RepSamples_NoNAs,
       aes(x = as.numeric(`1105-20a`),y= as.numeric(`1109-20a`))) +
  geom_point(alpha = 0.05, size = 0.3, show.legend = FALSE, colour = "lightsteelblue4") +
  #scale_size_continuous(range = c(1, 2)) +
  geom_density2d(colour="lightblue1", alpha = 0.5, size = 0.4) + 
  ggtitle('020118_2reps_nodups_Antreps_nolog') +
  #scale_x_log10(limit = c(10,1500)) +
  #scale_y_log10(limit = c(10,1500)) +
  scale_x_log10() +
  scale_y_log10() +
  xlab('nodups 1105-20ant') +
  ylab('nodups 1109-20ant') +
  #geom_smooth(method='lm', formula = y~x) +
  #ylim(0, 300) + 
  #xlim(0, 300) +
  coord_equal() +
  geom_abline(slope = 1, intercept=0, linetype = 'dotted') +
  annotate("text", x=500, y=500, label= AntReps_cor) +
  #annotate("text", x=500, y=1500, label= Post_Prom_Label, colour = 'dodgerblue3') +
  theme(panel.border = element_blank(),
        panel.background = element_rect(fill= NA),
        axis.line = element_line(colour = "black"),
        axis.title.x = element_text(vjust = 0, size = 20),
        axis.title.y = element_text(vjust = 1, size = 20),
        axis.text.x = element_text(size=20),
        axis.text.y  = element_text(size=20),
        plot.title = element_text(size = 12),
        legend.text = element_text(size = 20),
        legend.title = element_text(size = 20),
        strip.text.x = element_text(size = 12),
        strip.text.y = element_text(size = 12))
dev.off()

#### Posterior replicate graph
PostReps_cor = cor(as.numeric(RepSamples_NoNAs$`1105-20p`),y= as.numeric(RepSamples_NoNAs$`1109-20p`), method = "spearman")
PostReps_cor_pearson = cor(as.numeric(RepSamples_NoNAs$`1105-20p`),y= as.numeric(RepSamples_NoNAs$`1109-20p`), method = "pearson")
PostReps_cor_rsq = (PostReps_cor)^2
PostReps_cor_pearson__rsq = (PostReps_cor_pearson)^2
png('020118_2reps_nodups_Postreps_nolog.png', width = 4000, height = 2000, units = "px",  res=300) 
ggplot(RepSamples_NoNAs,
       aes(x = as.numeric(`1105-20p`),y= as.numeric(`1109-20p`))) +
  geom_point(alpha = 0.05, size = 0.3, show.legend = FALSE, colour = "lightsteelblue4") +
  #scale_size_continuous(range = c(1, 2)) +
  geom_density2d(colour="lightblue1", alpha = 0.5, size = 0.4) + 
  ggtitle('020118_2reps_nodups_Postreps_nolog') +
  #scale_x_log10(limit = c(10,1500)) +
  #scale_y_log10(limit = c(10,1500)) +
  xlab('nodups 1105-20post') +
  ylab('nodups 1109-20post') +
  #geom_smooth(method='lm', formula = y~x) +
  scale_x_log10() +
  scale_y_log10() +
  coord_equal() +
  geom_abline(slope = 1, intercept=0, linetype = 'dotted') +
  annotate("text", x=500, y=500, label= PostReps_cor) +
  #annotate("text", x=500, y=1500, label= Post_Prom_Label, colour = 'dodgerblue3') +
  theme(panel.border = element_blank(),
        panel.background = element_rect(fill= NA),
        axis.line = element_line(colour = "black"),
        axis.title.x = element_text(vjust = 0, size = 20),
        axis.title.y = element_text(vjust = 1, size = 20),
        axis.text.x = element_text(size=20),
        axis.text.y  = element_text(size=20),
        plot.title = element_text(size = 12),
        legend.text = element_text(size = 20),
        legend.title = element_text(size = 20),
        strip.text.x = element_text(size = 12),
        strip.text.y = element_text(size = 12))
dev.off()

#### Whole replicate graph
WholeReps_cor = cor(as.numeric(RepSamples_NoNAs$`1105-10w`),y= as.numeric(RepSamples_NoNAs$`1109-10w`), method = "spearman")
WholeReps_cor_pearson = cor(as.numeric(RepSamples_NoNAs$`1105-10w`),y= as.numeric(RepSamples_NoNAs$`1109-10w`), method = "pearson")
WholeReps_cor_rsq = (WholeReps_cor)^2
WholeReps_cor_pearson_rsq = (WholeReps_cor_pearson)^2
png('020118_2reps_nodups_Wholereps_nolog.png', width = 4000, height = 2000, units = "px",  res=300) 
ggplot(RepSamples_NoNAs,
       aes(x = as.numeric(`1105-10w`),y= as.numeric(`1109-10w`))) +
  geom_point(alpha = 0.05, size = 0.3, show.legend = FALSE, colour = "lightsteelblue4") +
  #scale_size_continuous(range = c(1, 2)) +
  geom_density2d(colour="lightblue1", alpha = 0.5, size = 0.4) + 
  ggtitle('020118_2reps_nodups_wholereps_nolog') +
  #scale_x_log10(limit = c(10,1500)) +
  #scale_y_log10(limit = c(10,1500)) +
  xlab('nodups 1105-10whole') +
  ylab('nodups 1109-10whole') +
  #geom_smooth(method='lm', formula = y~x) +
  scale_x_log10() +
  scale_y_log10() +
  coord_equal() +
  geom_abline(slope = 1, intercept=0, linetype = 'dotted') +
  annotate("text", x=500, y=500, label= WholeReps_cor) +
  #annotate("text", x=500, y=1500, label= Post_Prom_Label, colour = 'dodgerblue3') +
  theme(panel.border = element_blank(),
        panel.background = element_rect(fill= NA),
        axis.line = element_line(colour = "black"),
        axis.title.x = element_text(vjust = 0, size = 20),
        axis.title.y = element_text(vjust = 1, size = 20),
        axis.text.x = element_text(size=20),
        axis.text.y  = element_text(size=20),
        plot.title = element_text(size = 12),
        legend.text = element_text(size = 20),
        legend.title = element_text(size = 20),
        strip.text.x = element_text(size = 12),
        strip.text.y = element_text(size = 12))
dev.off()
```

#### Supp Figure 3¶

In [ ]:

```
#### Import New DnaseI 1kb region graph 
allSamples_DNASEI_NoNAs = allSamples_1kb_bins %>%
  select(X.chr., X.start., X.end., X011818_100417_BDTNP_DNaseI_stage5_ALLREPSCOMBINED_shifted_10MNorm, X011818_100417_BDTNP_DNaseI_stage9_ALLREPSCOMBINED_shifted_10MNorm, X011818_100417_BDTNP_DNaseI_stage11_ALLREPSCOMBINED_shifted_10MNorm,  X011818_100417_BDTNP_DNaseI_stage14_ALLREPSCOMBINED_shifted_10MNorm) %>%
  filter(X011818_100417_BDTNP_DNaseI_stage5_ALLREPSCOMBINED_shifted_10MNorm != 'nan') %>%
  filter(X011818_100417_BDTNP_DNaseI_stage9_ALLREPSCOMBINED_shifted_10MNorm != 'nan') %>%
  filter(X011818_100417_BDTNP_DNaseI_stage11_ALLREPSCOMBINED_shifted_10MNorm != 'nan') %>%
  filter(X011818_100417_BDTNP_DNaseI_stage14_ALLREPSCOMBINED_shifted_10MNorm != 'nan') %>%
  mutate(Name = "NA") %>%
  mutate(Location = "NA") %>%
  mutate(Type = "NA") %>%
  mutate(Dotsize = 1) %>%
  select(Name, Location, Type, X011818_100417_BDTNP_DNaseI_stage5_ALLREPSCOMBINED_shifted_10MNorm, X011818_100417_BDTNP_DNaseI_stage9_ALLREPSCOMBINED_shifted_10MNorm, X011818_100417_BDTNP_DNaseI_stage11_ALLREPSCOMBINED_shifted_10MNorm, X011818_100417_BDTNP_DNaseI_stage14_ALLREPSCOMBINED_shifted_10MNorm, Dotsize)
#c


st5_st14_Cor_pearson = round(cor(as.numeric(allSamples_DNASEI_NoNAs$X011818_100417_BDTNP_DNaseI_stage5_ALLREPSCOMBINED_shifted_10MNorm), as.numeric(allSamples_DNASEI_NoNAs$X011818_100417_BDTNP_DNaseI_stage14_ALLREPSCOMBINED_shifted_10MNorm), method = "pearson"),2)
st5_st14_Cor = round(cor(as.numeric(allSamples_DNASEI_NoNAs$X011818_100417_BDTNP_DNaseI_stage5_ALLREPSCOMBINED_shifted_10MNorm), as.numeric(allSamples_DNASEI_NoNAs$X011818_100417_BDTNP_DNaseI_stage14_ALLREPSCOMBINED_shifted_10MNorm), method = "spearman"),2)
st5_st14_Cor_pearson_sq = (st5_st14_Cor_pearson)^2
st5_st14_Cor_sq = (st5_st14_Cor)^2

png('020318_DnaseI_Stg5-Stg14_AllRegions1KB_RegNormWhole_Scatter_Newgenelist_LOG.png', width = 4000, height = 2000, units = "px",  res=300) 
ggplot(allSamples_DNASEI_NoNAs,
       aes(x = as.numeric(BDTNP_DnaseAcce_S5r1),y= as.numeric(BDTNP_DnaseAcce_S14r1))) +
  scale_color_manual(name = 'Legand', values=c("grey70")) +
  geom_point(alpha = 0.3, colour = "grey44") +
  scale_size_continuous(range = c(1, 2)) +
  geom_density2d(colour="lightblue1") + 
  ggtitle('020318_DnaseI_Stg5-Stg14_AllRegions1KB_RegNormWhole_Scatter_Newgenelist_LOG') +
  scale_x_log10() +
  scale_y_log10() +
  xlab('BDTNP_DNaseI_stage5') +
  ylab('BDTNP_DNaseI_stage14') +
  #geom_smooth(method='lm', formula = y~x) +
  #ylim(0,300) + 
  #xlim(0,300) +
  #coord_equal() +
  geom_abline(slope = 1, intercept=0, linetype = 'dotted') +
  annotate("text", x=100, y=300, label= st5_st14_Cor, colour = 'black') +
  #annotate("text", x=500, y=1500, label= Post_Prom_Label, colour = 'dodgerblue3') +
  theme(panel.border = element_blank(),
        panel.background = element_rect(fill= NA),
        axis.line = element_line(colour = "black"),
        axis.title.x = element_text(vjust = 0, size = 20),
        axis.title.y = element_text(vjust = 1, size = 20),
        axis.text.x = element_text(size=20),
        axis.text.y  = element_text(size=20),
        plot.title = element_text(size = 12),
        legend.text = element_text(size = 20),
        legend.title = element_text(size = 20),
        strip.text.x = element_text(size = 12),
        strip.text.y = element_text(size = 12))
dev.off()


st5_st9_Cor = round(cor(as.numeric(allSamples_DNASEI_NoNAs$X011818_100417_BDTNP_DNaseI_stage5_ALLREPSCOMBINED_shifted_10MNorm), as.numeric(allSamples_DNASEI_NoNAs$X011818_100417_BDTNP_DNaseI_stage9_ALLREPSCOMBINED_shifted_10MNorm), method = "spearman"),2)
st5_st9_Cor_pearson = round(cor(as.numeric(allSamples_DNASEI_NoNAs$X011818_100417_BDTNP_DNaseI_stage5_ALLREPSCOMBINED_shifted_10MNorm), as.numeric(allSamples_DNASEI_NoNAs$X011818_100417_BDTNP_DNaseI_stage9_ALLREPSCOMBINED_shifted_10MNorm), method = "pearson"),2)

st5_st9_Cor_pearson_sq = (st5_st9_Cor_pearson)^2
st5_st9_Cor_sq = (st5_st9_Cor)^2


png('020318_DnaseI_Stg5-Stg9_AllRegions1KB_RegNormWhole_Scatter_Newgenelist_LOG.png', width = 4000, height = 2000, units = "px",  res=300) 
ggplot(allSamples_DNASEI_NoNAs,
       aes(x = as.numeric(X011818_100417_BDTNP_DNaseI_stage5_ALLREPSCOMBINED_shifted_10MNorm),y= as.numeric(X011818_100417_BDTNP_DNaseI_stage9_ALLREPSCOMBINED_shifted_10MNorm))) +
  scale_color_manual(name = 'Legand', values=c("grey70")) +
  geom_point(alpha = 0.3, colour = "grey44") +
  scale_size_continuous(range = c(1, 2)) +
  geom_density2d(colour="lightblue1") + 
  ggtitle('020318_DnaseI_Stg5-Stg9_AllRegions1KB_RegNormWhole_Scatter_Newgenelist_LOG') +
  scale_x_log10() +
  scale_y_log10() +
  xlab('BDTNP_DNaseI_stage5') +
  ylab('BDTNP_DNaseI_stage9') +
  #geom_smooth(method='lm', formula = y~x) +
  #ylim(0,300) + 
  #xlim(0,300) +
  coord_equal() +
  geom_abline(slope = 1, intercept=0, linetype = 'dotted') +
  annotate("text", x=100, y=300, label= st5_st9_Cor, colour = 'black') +
  #annotate("text", x=500, y=1500, label= Post_Prom_Label, colour = 'dodgerblue3') +
  theme(panel.border = element_blank(),
        panel.background = element_rect(fill= NA),
        axis.line = element_line(colour = "black"),
        axis.title.x = element_text(vjust = 0, size = 20),
        axis.title.y = element_text(vjust = 1, size = 20),
        axis.text.x = element_text(size=20),
        axis.text.y  = element_text(size=20),
        plot.title = element_text(size = 12),
        legend.text = element_text(size = 20),
        legend.title = element_text(size = 20),
        strip.text.x = element_text(size = 12),
        strip.text.y = element_text(size = 12))
dev.off()


st5_st11_Cor = round(cor(as.numeric(allSamples_DNASEI_NoNAs$X011818_100417_BDTNP_DNaseI_stage5_ALLREPSCOMBINED_shifted_10MNorm), as.numeric(allSamples_DNASEI_NoNAs$X011818_100417_BDTNP_DNaseI_stage11_ALLREPSCOMBINED_shifted_10MNorm), method = "spearman"),2)
st5_st11_Cor_pearson = round(cor(as.numeric(allSamples_DNASEI_NoNAs$X011818_100417_BDTNP_DNaseI_stage5_ALLREPSCOMBINED_shifted_10MNorm), as.numeric(allSamples_DNASEI_NoNAs$X011818_100417_BDTNP_DNaseI_stage11_ALLREPSCOMBINED_shifted_10MNorm), method = "pearson"),2)

st5_st11_Cor_pearson_sq = (st5_st11_Cor_pearson)^2
st5_st11_Cor_sq = (st5_st11_Cor)^2

png('020318_DnaseI_Stg5-Stg11_AllRegions1KB_RegNormWhole_Scatter_Newgenelist_LOG.png', width = 4000, height = 2000, units = "px",  res=300) 
ggplot(allSamples_DNASEI_NoNAs,
       aes(x = as.numeric(X011818_100417_BDTNP_DNaseI_stage5_ALLREPSCOMBINED_shifted_10MNorm),y= as.numeric(X011818_100417_BDTNP_DNaseI_stage11_ALLREPSCOMBINED_shifted_10MNorm))) +
  scale_color_manual(name = 'Legand', values=c("grey70")) +
  geom_point(alpha = 0.3, colour = "grey44") +
  scale_size_continuous(range = c(1, 2)) +
  geom_density2d(colour="lightblue1") + 
  ggtitle('020318_DnaseI_Stg5-Stg11_AllRegions1KB_RegNormWhole_Scatter_Newgenelist_LOG') +
  scale_x_log10() +
  scale_y_log10() +
  xlab('BDTNP_DNaseI_stage5') +
  ylab('BDTNP_DNaseI_stage11') +
  #geom_smooth(method='lm', formula = y~x) +
  #ylim(0,300) + 
  #xlim(0,300) +
  coord_equal() +
  geom_abline(slope = 1, intercept=0, linetype = 'dotted') +
  annotate("text", x=100, y=300, label= st5_st11_Cor, colour = 'black') +
  #annotate("text", x=500, y=1500, label= Post_Prom_Label, colour = 'dodgerblue3') +
  theme(panel.border = element_blank(),
        panel.background = element_rect(fill= NA),
        axis.line = element_line(colour = "black"),
        axis.title.x = element_text(vjust = 0, size = 20),
        axis.title.y = element_text(vjust = 1, size = 20),
        axis.text.x = element_text(size=20),
        axis.text.y  = element_text(size=20),
        plot.title = element_text(size = 12),
        legend.text = element_text(size = 20),
        legend.title = element_text(size = 20),
        strip.text.x = element_text(size = 12),
        strip.text.y = element_text(size = 12))
dev.off()


st9_st11_Cor = round(cor(as.numeric(allSamples_DNASEI_NoNAs$X011818_100417_BDTNP_DNaseI_stage9_ALLREPSCOMBINED_shifted_10MNorm), as.numeric(allSamples_DNASEI_NoNAs$X011818_100417_BDTNP_DNaseI_stage11_ALLREPSCOMBINED_shifted_10MNorm), method = "spearman"),2)
st9_st11_Cor_pearson = round(cor(as.numeric(allSamples_DNASEI_NoNAs$X011818_100417_BDTNP_DNaseI_stage9_ALLREPSCOMBINED_shifted_10MNorm), as.numeric(allSamples_DNASEI_NoNAs$X011818_100417_BDTNP_DNaseI_stage11_ALLREPSCOMBINED_shifted_10MNorm), method = "pearson"),2)

st9_st11_Cor_pearson_sq = (st9_st11_Cor_pearson)^2
st9_st11_Cor_sq = (st9_st11_Cor)^2

png('020318_DnaseI_Stg9-Stg11_AllRegions1KB_RegNormWhole_Scatter_Newgenelist_LOG.png', width = 4000, height = 2000, units = "px",  res=300) 
ggplot(allSamples_DNASEI_NoNAs,
       aes(x = as.numeric(X011818_100417_BDTNP_DNaseI_stage9_ALLREPSCOMBINED_shifted_10MNorm),y= as.numeric(X011818_100417_BDTNP_DNaseI_stage11_ALLREPSCOMBINED_shifted_10MNorm))) +
  scale_color_manual(name = 'Legand', values=c("grey70")) +
  geom_point(alpha = 0.3, colour = "grey44") +
  scale_size_continuous(range = c(1, 2)) +
  geom_density2d(colour="lightblue1") + 
  ggtitle('020318_DnaseI_Stg9-Stg11_AllRegions1KB_RegNormWhole_Scatter_Newgenelist_LOG') +
  scale_x_log10() +
  scale_y_log10() +
  xlab('BDTNP_DNaseI_stage9') +
  ylab('BDTNP_DNaseI_stage11') +
  #geom_smooth(method='lm', formula = y~x) +
  #ylim(0,300) + 
  #xlim(0,300) +
  coord_equal() +
  geom_abline(slope = 1, intercept=0, linetype = 'dotted') +
  annotate("text", x=100, y=300, label= st9_st11_Cor, colour = 'black') +
  #annotate("text", x=500, y=1500, label= Post_Prom_Label, colour = 'dodgerblue3') +
  theme(panel.border = element_blank(),
        panel.background = element_rect(fill= NA),
        axis.line = element_line(colour = "black"),
        axis.title.x = element_text(vjust = 0, size = 20),
        axis.title.y = element_text(vjust = 1, size = 20),
        axis.text.x = element_text(size=20),
        axis.text.y  = element_text(size=20),
        plot.title = element_text(size = 12),
        legend.text = element_text(size = 20),
        legend.title = element_text(size = 20),
        strip.text.x = element_text(size = 12),
        strip.text.y = element_text(size = 12))
dev.off()

st9_st14_Cor = round(cor(as.numeric(allSamples_DNASEI_NoNAs$X011818_100417_BDTNP_DNaseI_stage9_ALLREPSCOMBINED_shifted_10MNorm), as.numeric(allSamples_DNASEI_NoNAs$X011818_100417_BDTNP_DNaseI_stage14_ALLREPSCOMBINED_shifted_10MNorm), method = "spearman"),2)
st9_st14_Cor_pearson = round(cor(as.numeric(allSamples_DNASEI_NoNAs$X011818_100417_BDTNP_DNaseI_stage9_ALLREPSCOMBINED_shifted_10MNorm), as.numeric(allSamples_DNASEI_NoNAs$X011818_100417_BDTNP_DNaseI_stage14_ALLREPSCOMBINED_shifted_10MNorm), method = "pearson"),2)

st9_st14_Cor_pearson_sq = (st9_st14_Cor_pearson)^2
st9_st14_Cor_sq = (st9_st14_Cor)^2
png('020318_DnaseI_Stg9-Stg14_AllRegions1KB_RegNormWhole_Scatter_Newgenelist_LOG.png', width = 4000, height = 2000, units = "px",  res=300) 
ggplot(allSamples_DNASEI_NoNAs,
       aes(x = as.numeric(X011818_100417_BDTNP_DNaseI_stage9_ALLREPSCOMBINED_shifted_10MNorm),y= as.numeric(X011818_100417_BDTNP_DNaseI_stage14_ALLREPSCOMBINED_shifted_10MNorm))) +
  scale_color_manual(name = 'Legand', values=c("grey70")) +
  geom_point(alpha = 0.3, colour = "grey44") +
  scale_size_continuous(range = c(1, 2)) +
  geom_density2d(colour="lightblue1") + 
  ggtitle('020318_DnaseI_Stg9-Stg14_AllRegions1KB_RegNormWhole_Scatter_Newgenelist_LOG') +
  scale_x_log10() +
  scale_y_log10() +
  xlab('BDTNP_DNaseI_stage9') +
  ylab('BDTNP_DNaseI_stage14') +
  #geom_smooth(method='lm', formula = y~x) +
  #ylim(0,300) + 
  #xlim(0,300) +
  coord_equal() +
  geom_abline(slope = 1, intercept=0, linetype = 'dotted') +
  annotate("text", x=100, y=300, label= st9_st14_Cor, colour = 'black') +
  #annotate("text", x=500, y=1500, label= Post_Prom_Label, colour = 'dodgerblue3') +
  theme(panel.border = element_blank(),
        panel.background = element_rect(fill= NA),
        axis.line = element_line(colour = "black"),
        axis.title.x = element_text(vjust = 0, size = 20),
        axis.title.y = element_text(vjust = 1, size = 20),
        axis.text.x = element_text(size=20),
        axis.text.y  = element_text(size=20),
        plot.title = element_text(size = 12),
        legend.text = element_text(size = 20),
        legend.title = element_text(size = 20),
        strip.text.x = element_text(size = 12),
        strip.text.y = element_text(size = 12))
dev.off()

st11_st14_Cor = round(cor(as.numeric(allSamples_DNASEI_NoNAs$X011818_100417_BDTNP_DNaseI_stage11_ALLREPSCOMBINED_shifted_10MNorm), as.numeric(allSamples_DNASEI_NoNAs$X011818_100417_BDTNP_DNaseI_stage14_ALLREPSCOMBINED_shifted_10MNorm), method = "spearman"),2)
st11_st14_Cor_pearson = round(cor(as.numeric(allSamples_DNASEI_NoNAs$X011818_100417_BDTNP_DNaseI_stage11_ALLREPSCOMBINED_shifted_10MNorm), as.numeric(allSamples_DNASEI_NoNAs$X011818_100417_BDTNP_DNaseI_stage14_ALLREPSCOMBINED_shifted_10MNorm), method = "pearson"),2)
st11_st14_Cor_pearson_sq = (st11_st14_Cor_pearson)^2
st11_st14_Cor_sq = (st11_st14_Cor)^2


png('020318_DnaseI_Stg11-Stg14_AllRegions1KB_RegNormWhole_Scatter_Newgenelist_LOG.png', width = 4000, height = 2000, units = "px",  res=300) 
ggplot(allSamples_DNASEI_NoNAs,
       aes(x = as.numeric(X011818_100417_BDTNP_DNaseI_stage11_ALLREPSCOMBINED_shifted_10MNorm),y= as.numeric(X011818_100417_BDTNP_DNaseI_stage14_ALLREPSCOMBINED_shifted_10MNorm))) +
  scale_color_manual(name = 'Legand', values=c("grey70")) +
  geom_point(alpha = 0.3, colour = "grey44") +
  scale_size_continuous(range = c(1, 2)) +
  geom_density2d(colour="lightblue1") + 
  ggtitle('020318_DnaseI_Stg11-Stg14_AllRegions1KB_RegNormWhole_Scatter_Newgenelist_LOG') +
  scale_x_log10() +
  scale_y_log10() +
  xlab('BDTNP_DNaseI_stage11') +
  ylab('BDTNP_DNaseI_stage14') +
  #geom_smooth(method='lm', formula = y~x) +
  #ylim(0,300) + 
  #xlim(0,300) +
  coord_equal() +
  geom_abline(slope = 1, intercept=0, linetype = 'dotted') +
  annotate("text", x=100, y=300, label= st11_st14_Cor, colour = 'black') +
  #annotate("text", x=500, y=1500, label= Post_Prom_Label, colour = 'dodgerblue3') +
  theme(panel.border = element_blank(),
        panel.background = element_rect(fill= NA),
        axis.line = element_line(colour = "black"),
        axis.title.x = element_text(vjust = 0, size = 20),
        axis.title.y = element_text(vjust = 1, size = 20),
        axis.text.x = element_text(size=20),
        axis.text.y  = element_text(size=20),
        plot.title = element_text(size = 12),
        legend.text = element_text(size = 20),
        legend.title = element_text(size = 20),
        strip.text.x = element_text(size = 12),
        strip.text
```

### Single Halves Analysis¶

In [ ]:

```
allSamples_yes_peaks_ATACSkew_1Avs1P = allSamples %>%
  filter(X011617.use == "yes") %>%
  filter(is.na(NoPeak) == TRUE) %>%
  select(chr, start, end, X011617_081817_RemDUP_041217_Bowtie2_ME_JH_112315_1105.ATACSlice2.5.1A_shifted_lessthan130_1MNorm, X011617_081817_RemDUP_041217_Bowtie2_ME_JH_112315_1105.ATACSlice2.6.1p_shifted_lessthan130_1MNorm, Name,New.Location.Assignment, Type, ATACSkewScore) %>%
  mutate(Dotsize = 1.5)
colnames(allSamples_yes_peaks_ATACSkew_1Avs1P) = c("Chr", "Start", "End", "OneA", "OneP", "Name", "Location", "Type", "ATACSkewScore", "Dotsize")


JustAEnhancers_one = allSamples_yes_peaks_ATACSkew_1Avs1P %>%
  select(Chr, Start, End, Location, Type, Name, OneA, OneP, ATACSkewScore) %>%
  filter(Location %in%  c('Anterior')) %>%
  filter(Type == 'Enhancer') %>%
  mutate(PositionalScore = (as.numeric(OneA) - as.numeric(OneP)) / (as.numeric(OneA) + as.numeric(OneP))) %>%
  distinct()

JustPEnhancers_one = allSamples_yes_peaks_ATACSkew_1Avs1P %>%
  select(Chr, Start, End, Location, Type, Name, OneA, OneP, ATACSkewScore) %>%
  filter(Location %in%  c('Posterior', 'Mostly Post')) %>%
  filter(Type == 'Enhancer') %>%
  mutate(PositionalScore = (as.numeric(OneA) - as.numeric(OneP)) / (as.numeric(OneA) + as.numeric(OneP))) %>%
  distinct()

JustAPEnhancers_one = bind_rows(JustAEnhancers_one, JustPEnhancers_one)


JustAPEnhancers_one$Location <- factor(JustAPEnhancers_one$Location,
                                       levels = c('Anterior', 'Posterior', 'Mostly Post'),ordered = TRUE)
ggplot(JustAPEnhancers_one,
       aes(x = reorder(as.factor(Name),- as.numeric(PositionalScore)),
           y = as.numeric(PositionalScore),
           fill = Location)) +
  geom_col(position = "stack", colour = "black") +
  xlab("A-P Enhancers") +
  ylab("PositionalScore") +
  ggtitle("020918_PE_2reps_nodups_SingleHalves_AP_Enhancers_PositionalScore_Bargraph_Position_0118newgeneList") +
  scale_fill_manual(name = "Legand", values=c("darkorange1", "dodgerblue3", "dodgerblue3")) +
  theme(panel.border = element_blank(),
        panel.background = element_rect(fill= NA),
        axis.line = element_line(colour = "black"),
        axis.title.x = element_text(vjust = 0, size = 15),
        axis.title.y = element_text(vjust = 1, size = 15),
        axis.text.x = element_text(size = 7, angle = -90),
        axis.text.y  = element_text(size= 15),
        plot.title = element_text(size = 15),
        legend.text = element_text(size = 15),
        legend.title = element_text(size = 15),
        strip.text.x = element_text(size = 12),
        strip.text.y = element_text(size = 12)) +
  png('020918_PE_2reps_nodups_SingleHalves_AP_Enhancers_PositionalScore_Bargraph_Position_0118newgeneList.png', width = 5000, height = 2000, units = "px",  res=300)
dev.off()

oneAvsoneP_Cor_pearson = round(cor(as.numeric(allSamples_1kb_bins$X011617_081817_RemDUP_041217_Bowtie2_ME_JH_112315_1105.ATACSlice2.5.1A_shifted_lessthan130_1MNorm), as.numeric(allSamples_1kb_bins$X011617_081817_RemDUP_041217_Bowtie2_ME_JH_112315_1105.ATACSlice2.6.1p_shifted_lessthan130_1MNorm), method = "pearson"),2)
oneAvsoneP_Cor = round(cor(as.numeric(allSamples_1kb_bins$X011617_081817_RemDUP_041217_Bowtie2_ME_JH_112315_1105.ATACSlice2.5.1A_shifted_lessthan130_1MNorm), as.numeric(allSamples_1kb_bins$X011617_081817_RemDUP_041217_Bowtie2_ME_JH_112315_1105.ATACSlice2.6.1p_shifted_lessthan130_1MNorm), method = "spearman"),2)
oneAvsoneP_Cor_rsq = (oneAvsoneP_Cor)^2
oneAvsoneP_Cor_pearson_rsq = (oneAvsoneP_Cor_pearson)^2
oneAvsoneP_lm = lm(as.numeric(allSamples_1kb_bins$X011617_081817_RemDUP_041217_Bowtie2_ME_JH_112315_1105.ATACSlice2.5.1A_shifted_lessthan130_1MNorm) ~ as.numeric(allSamples_1kb_bins$X011617_081817_RemDUP_041217_Bowtie2_ME_JH_112315_1105.ATACSlice2.6.1p_shifted_lessthan130_1MNorm))


png('020918_1avs1p_Scatterplot_Norm.png', width = 4000, height = 2000, units = "px",  res=300) 
ggplot(allSamples_1kb_bins,
       aes(x = as.numeric(X011617_081817_RemDUP_041217_Bowtie2_ME_JH_112315_1105.ATACSlice2.6.1p_shifted_lessthan130_1MNorm),y= (as.numeric(X011617_081817_RemDUP_041217_Bowtie2_ME_JH_112315_1105.ATACSlice2.5.1A_shifted_lessthan130_1MNorm)/ as.numeric(oneAvsoneP_lm$coefficients[2])))) +
  scale_color_manual(name = 'Legand', values=c("grey70")) +
  geom_point(alpha = 0.3, colour = "grey44") +
  scale_size_continuous(range = c(1, 2)) +
  geom_density2d(colour="lightblue1") + 
  ggtitle('020918_1avs1p_Scatterplot_Norm') +
  #scale_x_log10() +
  #scale_y_log10() +
  xlab('1A') +
  ylab('1P') +
  #geom_smooth(method='lm', formula = y~x) +
  #ylim(0,300) + 
  #xlim(0,300) +
  coord_equal() +
  geom_abline(slope = 1, intercept=0, linetype = 'dotted') +
  #annotate("text", x=100, y=300, label= st5_st14_Cor, colour = 'black') +
  #annotate("text", x=500, y=1500, label= Post_Prom_Label, colour = 'dodgerblue3') +
  theme(panel.border = element_blank(),
        panel.background = element_rect(fill= NA),
        axis.line = element_line(colour = "black"),
        axis.title.x = element_text(vjust = 0, size = 20),
        axis.title.y = element_text(vjust = 1, size = 20),
        axis.text.x = element_text(size=20),
        axis.text.y  = element_text(size=20),
        plot.title = element_text(size = 12),
        legend.text = element_text(size = 20),
        legend.title = element_text(size = 20),
        strip.text.x = element_text(size = 12),
        strip.text.y = element_text(size = 12))
dev.off()

png('020918_1avs1p_Scatterplot.png', width = 4000, height = 2000, units = "px",  res=300) 
ggplot(allSamples_1kb_bins,
       aes(x = as.numeric(X011617_081817_RemDUP_041217_Bowtie2_ME_JH_112315_1105.ATACSlice2.5.1A_shifted_lessthan130_1MNorm),y= as.numeric(X011617_081817_RemDUP_041217_Bowtie2_ME_JH_112315_1105.ATACSlice2.6.1p_shifted_lessthan130_1MNorm))) +
  scale_color_manual(name = 'Legand', values=c("grey70")) +
  geom_point(alpha = 0.3, colour = "grey44") +
  scale_size_continuous(range = c(1, 2)) +
  geom_density2d(colour="lightblue1") + 
  ggtitle('020918_1avs1p_Scatterplot') +
  #scale_x_log10() +
  #scale_y_log10() +
  xlab('1A') +
  ylab('1P') +
  #geom_smooth(method='lm', formula = y~x) +
  #ylim(0,300) + 
  #xlim(0,300) +
  coord_equal() +
  geom_abline(slope = 1, intercept=0, linetype = 'dotted') +
  #annotate("text", x=100, y=300, label= st5_st14_Cor, colour = 'black') +
  #annotate("text", x=500, y=1500, label= Post_Prom_Label, colour = 'dodgerblue3') +
  theme(panel.border = element_blank(),
        panel.background = element_rect(fill= NA),
        axis.line = element_line(colour = "black"),
        axis.title.x = element_text(vjust = 0, size = 20),
        axis.title.y = element_text(vjust = 1, size = 20),
        axis.text.x = element_text(size=20),
        axis.text.y  = element_text(size=20),
        plot.title = element_text(size = 12),
        legend.text = element_text(size = 20),
        legend.title = element_text(size = 20),
        strip.text.x = element_text(size = 12),
        strip.text.y = element_text(size = 12))
dev.off()


JustAPromoters_one = allSamples_yes_peaks_ATACSkew_1Avs1P %>%
  select(Chr, Start, End, Location, Type, Name, OneA, OneP, ATACSkewScore) %>%
  filter(Location %in%  c('Anterior', 'Mostly Ant')) %>%
  filter(Type == 'Promoter') %>%
  mutate(PositionalScore = (as.numeric(OneA) - as.numeric(OneP)) / (as.numeric(OneA) + as.numeric(OneP))) %>%
  distinct()

JustPPromoters_one = allSamples_yes_peaks_ATACSkew_1Avs1P %>%
  select(Chr, Start, End, Location, Type, Name, OneA, OneP, ATACSkewScore) %>%
  filter(Location %in%  c('Posterior', 'Mostly Post')) %>%
  filter(Type == 'Promoter') %>%
  mutate(PositionalScore = (as.numeric(OneA) - as.numeric(OneP)) / (as.numeric(OneA) + as.numeric(OneP))) %>%
  distinct()

JustAPPromoters_one = bind_rows(JustAPromoters_one, JustPPromoters_one)

#write.csv(JustAPEnhancers_one, "020318_2Reps_NewGenelist_PE_Analysis_APEnhancers_PositionalScore.csv")


JustAPPromoters_one$Location <- factor(JustAPPromoters_one$Location,
                                       levels = c('Anterior', 'Mostly Ant', 'Posterior', 'Mostly Post'),ordered = TRUE)

png('020918_PE_2reps_nodups_SingleHalves_AP_Promoter_PositionalScore_Bargraph_Position_0118newgeneList.png', width = 5000, height = 2000, units = "px",  res=300)
ggplot(JustAPPromoters_one,
       aes(x = reorder(as.factor(Name),- as.numeric(PositionalScore)),
           y = as.numeric(PositionalScore),
           fill = Location)) +
  #guides(fill= FALSE) +
  geom_col(position = "stack", colour = "black") +
  #ylim(-0.6, 0.6) +
  xlab("A-P Promoters") +
  ylab("PositionalScore") +
  ggtitle("020918_PE_2reps_nodups_SingleHalves_AP_Promoter_PositionalScore_Bargraph_Position_0118newgeneList") +
  scale_fill_manual(name = "Legand", values=c("darkorange1", "darkorange1", "dodgerblue3", "dodgerblue3")) +
  #geom_abline(slope = 0, intercept=0.2, linetype = 'dashed', size = 1) +
  #geom_abline(slope = 0, intercept=-0.2, linetype = 'dashed', size = 1) +
  #annotate("rect", xmin = 0 , xmax = Inf,   ymin = -0.2, ymax = 0.2,   alpha = 0.2, fill = "slategray4") +
  #scale_fill_gradient(high = "darkorange1", low = "dodgerblue3") +
  #annotate("text", x=500, y=0, label= Ant_Label, colour = 'darkorange1') +
  theme(panel.border = element_blank(),
        panel.background = element_rect(fill= NA),
        axis.line = element_line(colour = "black"),
        axis.title.x = element_text(vjust = 0, size = 15),
        axis.title.y = element_text(vjust = 1, size = 15),
        axis.text.x = element_text(size = 7, angle = -90),
        axis.text.y  = element_text(size= 15),
        plot.title = element_text(size = 15),
        legend.text = element_text(size = 15),
        legend.title = element_text(size = 15),
        strip.text.x = element_text(size = 12),
        strip.text.y = element_text(size = 12))

dev.off()

######### ######### ######### DV Enhancer Bargraphs 
JustDorsalEnhancers_One = allSamples_yes_peaks_ATACSkew_1Avs1P %>%
  select(Location, Type, Name, OneA, OneP, ATACSkewScore) %>%
  filter(Location == "Dorsal") %>%
  filter(Type == 'Enhancer') %>%
  mutate(PositionalScore = (as.numeric(OneA) - as.numeric(OneP)) / (as.numeric(OneA) + as.numeric(OneP))) %>%
  distinct()

JustVentralEnhancers_One = allSamples_yes_peaks_ATACSkew_1Avs1P %>%
  select(Location, Type, Name, OneA, OneP, ATACSkewScore) %>%
  filter(Location == "Ventral") %>%
  filter(Type == 'Enhancer') %>%
  mutate(PositionalScore = (as.numeric(OneA) - as.numeric(OneP)) / (as.numeric(OneA) + as.numeric(OneP))) %>%
  distinct()


JustDVEnhancers_One = bind_rows(JustDorsalEnhancers_One, JustVentralEnhancers_One)

JustDVEnhancers_One$Location <- factor(JustDVEnhancers_One$Location,
                                   levels = c("Dorsal", "Ventral"),ordered = TRUE)

ggplot(JustDVEnhancers_One,
       aes(x = reorder(as.factor(Name),- as.numeric(PositionalScore)),
           y = as.numeric(PositionalScore),
           fill = Location)) +
  #guides(fill= FALSE) +
  geom_col(position = "stack", colour = "black") +
  #ylim(-0.6, 0.6) +
  xlab("D-V Enhancers") +
  ylab("PositionalScore") +
  ggtitle("020918_PE_2reps_nodups_SingleHalves_DV_Enhancers_PositionalScore_Bargraph_Position_0118newgeneList") +
  scale_fill_manual(name = "Legand", values=c("orchid4", "seagreen4")) +
  #geom_abline(slope = 0, intercept=0.2, linetype = 'dashed', size = 1) +
  #geom_abline(slope = 0, intercept=-0.2, linetype = 'dashed', size = 1) +
  #annotate("rect", xmin = 0 , xmax = Inf,   ymin = -0.2, ymax = 0.2,   alpha = 0.2, fill = "slategray4") +
  #scale_fill_gradient(high = "darkorange1", low = "dodgerblue3") +
  #annotate("text", x=500, y=0, label= Ant_Label, colour = 'darkorange1') +
  theme(panel.border = element_blank(),
        panel.background = element_rect(fill= NA),
        axis.line = element_line(colour = "black"),
        axis.title.x = element_text(vjust = 0, size = 15),
        axis.title.y = element_text(vjust = 1, size = 15),
        axis.text.x = element_text(size = 7, angle = -90),
        axis.text.y  = element_text(size= 15),
        plot.title = element_text(size = 15),
        legend.text = element_text(size = 15),
        legend.title = element_text(size = 15),
        strip.text.x = element_text(size = 12),
        strip.text.y = element_text(size = 12)) +
  png('020918_PE_2reps_nodups_SingleHalves_DV_Enhancers_PositionalScore_Bargraph_Position_0118newgeneList.png', width = 5000, height = 2000, units = "px",  res=300)
dev.off()
######### ######### ######### DV Promoter Bargraphs 
JustDorsalPromoter_One = allSamples_yes_peaks_ATACSkew_1Avs1P %>%
  select(Location, Type, Name, OneA, OneP, ATACSkewScore) %>%
  filter(Location == "Dorsal") %>%
  filter(Type == 'Promoter') %>%
  mutate(PositionalScore = (as.numeric(OneA) - as.numeric(OneP)) / (as.numeric(OneA) + as.numeric(OneP))) %>%
  distinct()

JustVentralPromoter_One = allSamples_yes_peaks_ATACSkew_1Avs1P %>%
  select(Location, Type, Name, OneA, OneP, ATACSkewScore) %>%
  filter(Location == "Ventral") %>%
  filter(Type == 'Promoter') %>%
  mutate(PositionalScore = (as.numeric(OneA) - as.numeric(OneP)) / (as.numeric(OneA) + as.numeric(OneP))) %>%
  distinct()

JustDVPromoterOne = bind_rows(JustDorsalPromoter_One, JustVentralPromoter_One)

JustDVPromoterOne$Location <- factor(JustDVPromoterOne$Location,
                                  levels = c("Dorsal", "Ventral"),ordered = TRUE)

ggplot(JustDVPromoterOne,
       aes(x = reorder(as.factor(Name),- as.numeric(PositionalScore)),
           y = as.numeric(PositionalScore),
           fill = Location)) +
  #guides(fill= FALSE) +
  geom_col(position = "stack", colour = "black") +
  ylim(-1, 1) +
  xlab("D-V Promoters") +
  ylab("PositionalScore") +
  ggtitle("020918_2reps_nodups_SingleHalves_DV_Promoters_PositionalScore_Bargraph_Position_0118newgeneList") +
  scale_fill_manual(name = "Legand", values=c("orchid4", "seagreen4")) +
  #geom_abline(slope = 0, intercept=0.2, linetype = 'dashed', size = 1) +
  #geom_abline(slope = 0, intercept=-0.2, linetype = 'dashed', size = 1) +
  #annotate("rect", xmin = 0 , xmax = Inf,   ymin = -0.2, ymax = 0.2,   alpha = 0.2, fill = "slategray4") +
  #scale_fill_gradient(high = "darkorange1", low = "dodgerblue3") +
  #annotate("text", x=500, y=0, label= Ant_Label, colour = 'darkorange1') +
  theme(panel.border = element_blank(),
        panel.background = element_rect(fill= NA),
        axis.line = element_line(colour = "black"),
        axis.title.x = element_text(vjust = 0, size = 15),
        axis.title.y = element_text(vjust = 1, size = 15),
        axis.text.x = element_text(size = 7, angle = -90),
        axis.text.y  = element_text(size= 15),
        plot.title = element_text(size = 15),
        legend.text = element_text(size = 15),
        legend.title = element_text(size = 15),
        strip.text.x = element_text(size = 12),
        strip.text.y = element_text(size = 12)) +
  png('020918_2reps_nodups_SingleHalves_DV_Promoters_PositionalScore_Bargraph_Position_0118newgeneList.png', width = 5000, height = 2000, units = "px",  res=300)
dev.off()
```

### Figure 5 and Supplementary Figure 5¶

In [ ]:

```
# ############################# Tf binding figure #############################

# Generate text file of average wig signal around 10 bp regions
perl wig_sig_around_bedfile_013018_for10bpwindows.pl \
/Volumes/'JHHD 1'/020118_dm3_10bpwindows.bed \
~/020218_TFwigfiles \
020218_TF_wig_sig_10bp_windows_XY.txt

## Make a text file containing only wig signal for supplied bed intervals
mkdir 020218_TF_Wigfilesplitter
cd 020218_TF_Wigfilesplitter

python /Users/jennahaines/'Box Sync'/Eisen_Lab/Scripts/061617_WigFileSnapshots_V2.py \
-W ~/020218_TF_wig_sig_10bp_windows_XY.txt \
-R ~/012318_ReviewsRevised_RevisedAPDV_enhPromRegions_FINAL_YESONLY.txt \
-l 3000
```

In [ ]:

```
#wig_sig_around_bedfile_013018_for10bpwindows.pl
#!/usr/bin/perl

use warnings;

#average signal over a bed region
my $peaks =shift; #bed file 
my $dir = shift;  #wig file dir
# my $NF = shift;  #normalization table
my $output = shift;
#my $win = shift;

# my %NF_t;

# open (NFT,'<', $NF);
# while (<NFT>){
#  next if(/#/);

#  my ($FS, $v) = split /\s+/,$_;
# $NF_t{$FS} = $v;

# }

my %HOAg=();

open (OUTPUT, '>', $output);
#print OUTPUT "#gen_ann,average signal within cluster region\n";
#print OUTPUT "#ID\tchr\tpeak_pos1\tpeak_pos2\tinterval_length\t";  #for union of peaks file
open (INPUTp, '<', $peaks);

my $N=1;
while (<INPUTp>){
   if (/^chr(\d|X)/) {
    @split = split (/\s+/, $_);

   # my $PID = $split[0];
    $HOAg{$N} = [@split];
    $N +=1;
  } else {
     @split = split (/\s+/, $_);
     $st = join ("\t",@split);
     print OUTPUT "$st";
    }
}


opendir DIR, $dir or die "can't open $dir: $!";

while ( my $file = readdir DIR ) {
  if ($file =~ /(.+).wig$/) {
    $HM = "$1";
    print OUTPUT "\t$HM";

    $In_file = "$dir/$file";
    print "infile:$file\n";

    my %HOA;
    my $chr2 = "NA";
    my %chr_sz=(
	   "chr2L" => 23011344, #new size info, aug 2014
	   "chr2R" => 21146608,
	   "chr3L" => 24543457,
	   "chr3R" => 27905053,
	   "chr4" => 1351757,
	   "chrX" => 22332727);

    foreach $chr (keys %chr_sz){
      $n = int($chr_sz{$chr}/10);
      @a = (0)x$n;
      $HOA{$chr} = [@a];
		}


    open (INPUT, '<', $In_file);
    while ( <INPUT> ) {
      if ($_ =~/^tr/){
	next}elsif ($_ =~ /chrom=(\w+)\s+/){
	  $chr2 = $1;
	}elsif ($_ =~/^\d+/){
	  @s=split (/\s+/, $_);
	  $p =int( $s[0]/10);		      		     
	  $HOA{$chr2}[$p] += $s[1];		     
	}
}
close INPUT;


    foreach $i (keys %HOAg){
      $chr3 = $HOAg{$i}[0];  #
     # $L = int(($HOAg{$i}[4])/10)-20;
     # $st_p = int($HOAg{$i}[2]/10)-($L/2);
     # $en_p = int($HOAg{$i}[2]/10)+($L/2);

   #$st_p = int($HOAg{$i}[2]/10)-($win/10/2);
   #$en_p = int($HOAg{$i}[2]/10)+($win/10/2); 
   $st_p = int($HOAg{$i}[1]/10); # 5873439 becomes 587343
   #$en_p = int($HOAg{$i}[2]/10);

    #$L = ($en_p - $st_p);


      $val= @{$HOA{$chr3}}[$st_p];
      #print "$val\n";
  #$vf= sprintf("%.2f", $val);
  push @{$HOAg{$i}}, $val;
   #push @{$HOAg{$i}}, $total;
	
    }
  }
}

print OUTPUT "\n";

foreach $i (sort {$a <=> $b}(keys %HOAg)){
  $str = join ("\t", @{$HOAg{$i}});
print OUTPUT "$str\n"
}
close DIR;
close OUTPUT;
```

In [ ]:

```
#############################################################
######### 061617_WigFileSnapshots_V2.py                #########
######### Have a wig file in four columns and       #########
######### then pull out wig signal for a given range#########
#############################################################

#!/usr/bin/env python


import os
from math import *
from optparse import OptionParser
import sys
import re

################# Argument Import : Uncomment for actual running script  #################

def parse_options():
	parser = OptionParser()
	parser.add_option("-W", "--wigfile", dest="wigfile",
					  help="4 columned TXT file of normalized wig signal: chr, start, end, wigvalue")
	parser.add_option("-R", "--regfile", dest="regfile",
					  help="5 columned TXT file of wanted genomic regions: Number, chr, start, end, Value")
	parser.add_option("-l", "--length", dest="length",
					  help="total genomic length returned")
	(options, args) = parser.parse_args()
	return options
            
options = parse_options()
parser = OptionParser()
if not options.wigfile:
    print("Wigfile option is missing\n")
    parser.print_help()
    exit(-1)
if not options.regfile:
    print("Region file option is missing\n")
    parser.print_help()
    exit(-1)
if not options.length:
    print("Length option is missing\n")
    parser.print_help()
    exit(-1)

################# Data Import   #################
# for jupyter Notebook pass these arguments
# wigfile = '061417_multiBigWigCompare_bins_041417_Bowtie2_ATACPools_merged.txt'
# regfile = 'MergedALL2.txt'
# length = 2000

####### Read in 4 columned Wig file
#'chr'	'start'	'end'	'041417_Bowtie2_ATACPools_merged_10Whole_10MDmel.bw'	'041417_Bowtie2_ATACPools_merged_20Anterior_10MDmel.bw'	'041417_Bowtie2_ATACPools_merged_20Posterior_10MDmel.bw'	'041417_Bowtie2_ATACPools_merged_AplusP_10MDmel.bw'	'101416_10MNorm_DNase-I_dmels5r1.bw'
#chr2R	0	10	nan	nan	nan	nan	nan
#chr2R	10	20	nan	nan	nan	nan	nan

WigFile_file = open(str(options.wigfile), 'r')
WigList = WigFile_file.readlines()
WigFile_file.close()
print('Wig File Imported')
                    
####### Read in File with regions I want to get
                    
#"Sample"	"chr"	"start"	"end"	"Merged_Whole"	"Merged_20Ant"	"Merged_20Post"	"Merged_AplusP"	"DnaseI"	"Type"	"Location"	"Name"	"Dotsize"
#"1"	"chr2R"	17598962	17599122	"118.7752312"	"135.1978857"	"71.82917128"	"164.9578282"	"67.41856544"	"Promoter"	"Posteriorly Primarily"	"CG30403"	1.5
#"2"	"chr3R"	7702946	7702963	"718.3436746"	"436.3480655"	"775.9198213"	"575.2080976"	"696.9214442"	"Promoter"	"OnlyA"	"sad"	1.5
#"3"	"chr2R"	8146899	8146969	"116.2616414"	"98.17407118"	"160.4658203"	"104.0412238"	"130.1133728"	"Promoter"	"Mostly A"	"Cam"	1.5

RegionsFile_file = open(str(options.regfile), 'r')
RegionsList = RegionsFile_file.readlines()
RegionsFile_file.close()
print('Regions File Imported')

WigFirstLine = WigList.pop(0)
RegionFirstLine = RegionsList.pop(0)

####### Create a list data frame where each list is a list containing information for one enhancer location  #################
RegionsListElement = []

# For each enhancer, strip the line ending, and split up line by \t and add that new list to a list of lists
WigDict = {}
for line in WigList:
    line = line.strip('\n')
    #WigListElement.append(line.split('\t'))
    wigregion = line.split('\t')
    #print(wigregion)
    wigChrom = wigregion[0]
    wigStart = int(wigregion[1])
    wigEnd = int(wigregion[2])
    WigKey = '_'.join([str(wigChrom),str(wigStart),str(wigEnd)])
    WigDict[WigKey] = line
#print(WigDict)
print("wig dictionary made")
print(WigDict["chrX_9583490_9583500"])

for line in RegionsList:
    line = line.strip('\n')
    RegionsListElement.append(line.split('\t'))
print(RegionsListElement[0:10])
print("Files made into lists")

################# Make a dictionary of all the keys between each region 

# Then for each region make a key and then compare it to the wigDict. If it's there, print the key value to a file. 
for region in RegionsListElement:
    #make variables 
    chrom = region[0]
    Start = int(region[1])
    End = int(region[2])
    TotalLength = End - Start
    #adjust the start and end to reflect the user input length
    if TotalLength < float(options.length):
        AddedLength = (float(options.length) - TotalLength)/2
        ExtendedStart = Start - AddedLength
        ExtendedEnd = End + AddedLength
    else:
        ExtendedStart = Start
        ExtendedEnd = End
    #round start and end so they match wig
    RoundedStart = int(int(ExtendedStart/10) * 10)
    #print(Start)
    #print(RoundedStart)
    RoundedEnd = int(int(ExtendedEnd/10) * 10) + 10
#     print(RoundedEnd)
#     print(chrom)
#     print(RoundedStart)
#     print(RoundedEnd)
    #make output files
    OutputFileNameString = str(region[5]) + "_WigFileSnapshot.txt"
    print(OutputFileNameString)
    outputFile = open(OutputFileNameString, 'w')
    
    #make a key for each 10bp wig interval in the extended region and compare that to wig dict
    while RoundedStart < RoundedEnd: # if start plus 10 is less than the end,
        RoundedStart += 10
        key = '_'.join([str(chrom),str(RoundedStart - 10),str(RoundedStart)]) #add this key
        outputFile.write(str(WigDict[key]))
        outputFile.write('\n')
    outputFile.close()
print("All done! :)")
```

In [ ]:

```
####################################################################
######### 020318 TF Browser Traces                      ############ 
####################################################################

library("plyr")
library("dplyr")
library("ggplot2")
library("grid")

#Set working directory
setwd("/021318_TF_Wigfilesplitter/")
RegionFile = read.delim2("011618_ReviewsRevised_RevisedAPDV_enhPromRegions.txt", sep = "\t", stringsAsFactors = FALSE, header = TRUE)
colnames(RegionFile) = c("chrom", "start", "end", "Type", "Location", "Name", "use", "length", "fbID", "source")
RegionFile = RegionFile %>% 
  filter(use == "yes")
  

#Make a list of the files
FileList = list.files("021318_TF_Wigfilesplitter/")

TestList = FileList[0:4]
LengthofList = as.numeric(n_distinct(FileList))
counter = 1

BicoidList = list()
HbList = list()
KRList = list()
GTList = list()
ZLD_st3List = list()
ZLD_st4List = list()
ZLD_st5List = list()
CADrList = list()
KNIList = list()
AntList = list()
PostList = list()
DnaseIList = list()
EnhancerNameList = list()

while(counter <= LengthofList){

  ExtendedRegionFile = read.delim2(paste(as.character(FileList[counter])), sep = "\t", stringsAsFactors = FALSE, header = FALSE)
  #Rename the columns
  colnames(ExtendedRegionFile) = c("chrom", "start", "end", 'whole', 'ant', 'post', 'dnaseI', 'Bcd',	'Hb',	'KR',	'GT',	'ZLD_st3', 'ZLD_st4', 'ZLD_st5', 'CADr', 'KNI', "chr1", "start1", "end1",'DnaseI2')

  ExtendedRegionFile[ExtendedRegionFile == 'nan'] <- 0 #remove the nan
  ExtendedRegionFile[ExtendedRegionFile == 'NaN'] <- 0 #remove the nan

  #Make the title the name of the file
  titleLabel = paste(as.character(FileList[counter]))

  #Extract out the region name from the file name
  NameofRegion = strsplit(titleLabel, '_WigFileSnapshot.txt')

  #Look up the name in the RegionFile and obtain the chr, start, and end coordinates
  RegionCoord = RegionFile %>%
    filter(Name == paste(as.character(NameofRegion[1])))

  RegionChr = as.character(RegionCoord$chrom)
  RegionStart = as.numeric(RegionCoord$start)
  RegionEnd = as.numeric(RegionCoord$end)

  #make the output file name
  fileLabel = paste(NameofRegion,"WigFileSnapshot.png", sep = "_")
  filelabelpath = paste("~/042418_TFWig/", fileLabel, sep = "")
  print("fileLabel")

  
   #strategy: I am going make a new graph for each column and then arange them in a 1x9 strip using grid
  #         Then I will merge all the grids into one panel for each region

  png(filelabelpath, width = 1500, height = 5000, units = "px",  res=300)
  grid.newpage()
  AntpostATAC = ggplot(ExtendedRegionFile,
                       aes(x = as.numeric(ExtendedRegionFile$start))) +
    annotate("rect", xmin = RegionStart[1], xmax = RegionEnd[1],   ymin = 0, ymax = Inf,   alpha = 0.2, fill = "slategray4") +
    geom_ribbon(aes(ymin = 0, ymax = as.numeric(ExtendedRegionFile$ant)), colour = "darkorange1", lwd = 1, fill = "darkorange1", alpha = 0.3)  +
    geom_ribbon(aes(ymin = 0, ymax = as.numeric(ExtendedRegionFile$post)), colour = "dodgerblue3", lwd = 1, fill = "dodgerblue3", alpha = 0.2)  +
    xlab(paste(RegionChr[1])) +
    #ylim(0,3000) +
    scale_y_continuous(limits = c(0,5000), breaks=c(0,5000)) +
    ggtitle(as.character(NameofRegion)) +
    #ylab("Normalized Wig Score") +
    theme(panel.border = element_blank(),
          panel.background = element_rect(fill= NA),
          axis.line = element_line(colour = "black"),
          axis.title.x = element_blank(),
          axis.title.y = element_text(vjust = 1, size = 20),
          axis.text.x = element_blank(),
          axis.text.y  = element_text(size=20),
          plot.title = element_text(size = 20),
          legend.text = element_text(size = 20),
          legend.title = element_text(size = 20),
          strip.text.x = element_text(size = 20),
          strip.text.y = element_text(size = 20),
          plot.margin = grid::unit(c(0.5, 0.5,
                                     0.5, 0.5), "cm"))
  DnaseI_plot = ggplot(ExtendedRegionFile,
                       aes(x = as.numeric(ExtendedRegionFile$start)))+
    annotate("rect", xmin = RegionStart[1], xmax = RegionEnd[1],   ymin = 0, ymax = Inf,   alpha = 0.2, fill = "slategray4") +
    geom_ribbon(aes(ymin = 0, ymax = as.numeric(ExtendedRegionFile$DnaseI2)), colour = "green", lwd = 1, fill = "green", alpha = 0.3, show.legend = TRUE)  +
    #xlab(paste(RegionChr[1])) +
    #ylim(0,3000) +
    scale_y_continuous(limits = c(0,3000), breaks=c(3000)) +
    #ylab("Normalized Wig Score") +
    theme(panel.border = element_blank(),
          panel.background = element_rect(fill= NA),
          axis.line = element_line(colour = "black"),
          axis.title.x = element_blank(),
          axis.title.y = element_text(vjust = 1, size = 20),
          axis.text.x = element_blank(),
          axis.text.y  = element_text(size=20),
          plot.title = element_text(size = 20),
          legend.text = element_text(size = 20),
          legend.title = element_text(size = 20),
          strip.text.x = element_text(size = 20),
          strip.text.y = element_text(size = 20))

  BCD_plot = ggplot(ExtendedRegionFile,
                    aes(x = as.numeric(ExtendedRegionFile$start)))+
    annotate("rect", xmin = RegionStart[1], xmax = RegionEnd[1],   ymin = 0, ymax = Inf,   alpha = 0.2, fill = "slategray4") +
    geom_ribbon(aes(ymin = 0, ymax = as.numeric(ExtendedRegionFile$Bcd)), colour = "indianred4", lwd = 1, fill = "indianred4", alpha = 0.3, show.legend = TRUE)  +
    #xlab(paste(RegionChr[1])) +
    #ylim(0,50) +
    scale_y_continuous(limits = c(0,50), breaks=c(0,50)) +
    #ylab("Normalized Wig Score") +
    theme(panel.border = element_blank(),
          panel.background = element_rect(fill= NA),
          axis.line = element_line(colour = "black"),
          axis.title.x = element_blank(),
          axis.title.y = element_text(vjust = 1, size = 20),
          axis.text.x = element_blank(),
          axis.text.y  = element_text(size=20),
          plot.title = element_text(size = 20),
          legend.text = element_text(size = 20),
          legend.title = element_text(size = 20),
          strip.text.x = element_text(size = 20),
          strip.text.y = element_text(size = 20))

  Hb_plot = ggplot(ExtendedRegionFile,
                   aes(x = as.numeric(ExtendedRegionFile$start)))+
    annotate("rect", xmin = RegionStart[1], xmax = RegionEnd[1],   ymin = 0, ymax = Inf,   alpha = 0.2, fill = "slategray4") +
    geom_ribbon(aes(ymin = 0, ymax = as.numeric(ExtendedRegionFile$Hb)), colour = "sienna2", lwd = 1, fill = "sienna2", alpha = 0.3, show.legend = TRUE)  +
    #ylab("Normalized Wig Score") +
    #ylim(0,150) +
    scale_y_continuous(limits = c(0,180), breaks=c(0,180)) +
    theme(panel.border = element_blank(),
          panel.background = element_rect(fill= NA),
          axis.line = element_line(colour = "black"),
          axis.title.x = element_blank(),
          axis.title.y = element_text(vjust = 1, size = 20),
          axis.text.x = element_blank(),
          axis.text.y  = element_text(size=20),
          plot.title = element_text(size = 20),
          legend.text = element_text(size = 20),
          legend.title = element_text(size = 20),
          strip.text.x = element_text(size = 20),
          axis.ticks.x=element_blank(),
          strip.text.y = element_text(size = 20))

  KR_plot = ggplot(ExtendedRegionFile,
                   aes(x = as.numeric(ExtendedRegionFile$start)))+
    annotate("rect", xmin = RegionStart[1], xmax = RegionEnd[1],   ymin = 0, ymax = Inf,   alpha = 0.2, fill = "slategray4") +
    geom_ribbon(aes(ymin = 0, ymax = as.numeric(ExtendedRegionFile$KR)), colour = "goldenrod2", lwd = 1, fill = "goldenrod2", alpha = 0.3, show.legend = TRUE)  +
    #ylab("Normalized Wig Score") +
    #ylim(0,150) +
    scale_y_continuous(limits = c(0,150), breaks=c(0,150)) +
    theme(panel.border = element_blank(),
          panel.background = element_rect(fill= NA),
          axis.line = element_line(colour = "black"),
          axis.title.x = element_blank(),
          axis.title.y = element_text(vjust = 1, size = 20),
          axis.text.x = element_blank(),
          axis.text.y  = element_text(size=20),
          legend.text = element_text(size = 20),
          legend.title = element_text(size = 20),
          strip.text.x = element_text(size = 20),
          axis.ticks.x=element_blank(),
          strip.text.y = element_text(size = 20))

  GT_plot = ggplot(ExtendedRegionFile,
                   aes(x = as.numeric(ExtendedRegionFile$start)))+
    annotate("rect", xmin = RegionStart[1], xmax = RegionEnd[1],   ymin = 0, ymax = Inf,   alpha = 0.2, fill = "slategray4") +
    geom_ribbon(aes(ymin = 0, ymax = as.numeric(ExtendedRegionFile$GT)), colour = "darkseagreen4", lwd = 1, fill = "darkseagreen4", alpha = 0.3, show.legend = TRUE)  +
    #ylab("Normalized Wig Score") +
    #ylim(0,150) +
    scale_y_continuous(limits = c(0,150), breaks=c(0,150)) +
    theme(panel.border = element_blank(),
          panel.background = element_rect(fill= NA),
          axis.line = element_line(colour = "black"),
          axis.title.x = element_blank(),
          axis.title.y = element_text(vjust = 1, size = 20),
          axis.text.x = element_blank(),
          axis.text.y  = element_text(size=20),
          plot.title = element_text(size = 20),
          legend.text = element_text(size = 20),
          legend.title = element_text(size = 20),
          strip.text.x = element_text(size = 20),
          axis.ticks.x=element_blank(),
          strip.text.y = element_text(size = 20))

  ZLD3_plot = ggplot(ExtendedRegionFile,
                     aes(x = as.numeric(ExtendedRegionFile$start)))+
    annotate("rect", xmin = RegionStart[1], xmax = RegionEnd[1],   ymin = 0, ymax = Inf,   alpha = 0.2, fill = "slategray4") +
    geom_ribbon(aes(ymin = 0, ymax = as.numeric(ExtendedRegionFile$ZLD_st3)), colour = "steelblue", lwd = 1, fill = "steelblue", alpha = 0.3, show.legend = TRUE)  +
    #geom_ribbon(aes(ymin = 0, ymax = as.numeric(ExtendedRegionFile$ZLD_st4)), colour = "steelblue3", lwd = 1, fill = NA, alpha = 0.3, show.legend = TRUE)  +
    #geom_ribbon(aes(ymin = 0, ymax = as.numeric(ExtendedRegionFile$ZLD_st5)), colour = "steelblue1", lwd = 1, fill = NA, alpha = 0.3, show.legend = TRUE)  +
    #ylab("Normalized Wig Score") +
    #ylim(0,500) +
    scale_y_continuous(limits = c(0,500), breaks=c(0,500)) +
    theme(panel.border = element_blank(),
          panel.background = element_rect(fill= NA),
          axis.line = element_line(colour = "black"),
          axis.title.x = element_blank(),
          axis.title.y = element_text(vjust = 1, size = 20),
          axis.text.x = element_blank(),
          axis.text.y  = element_text(size=20),
          plot.title = element_text(size = 20),
          legend.text = element_text(size = 20),
          legend.title = element_text(size = 20),
          strip.text.x = element_text(size = 20),
          axis.ticks.x=element_blank(),
          strip.text.y = element_text(size = 20))
  ZLD4_plot = ggplot(ExtendedRegionFile,
                     aes(x = as.numeric(ExtendedRegionFile$start)))+
    annotate("rect", xmin = RegionStart[1], xmax = RegionEnd[1],   ymin = 0, ymax = Inf,   alpha = 0.2, fill = "slategray4") +
    #geom_ribbon(aes(ymin = 0, ymax = as.numeric(ExtendedRegionFile$ZLD_st3)), colour = "steelblue", lwd = 1, fill = NA, alpha = 0.3, show.legend = TRUE)  +
    geom_ribbon(aes(ymin = 0, ymax = as.numeric(ExtendedRegionFile$ZLD_st4)), colour = "steelblue3", lwd = 1, fill = "steelblue3", alpha = 0.3, show.legend = TRUE)  +
    #geom_ribbon(aes(ymin = 0, ymax = as.numeric(ExtendedRegionFile$ZLD_st5)), colour = "steelblue1", lwd = 1, fill = NA, alpha = 0.3, show.legend = TRUE)  +
    #ylab("Normalized Wig Score") +
    #ylim(0,500) +
    scale_y_continuous(limits = c(0,500), breaks=c(0,500)) +
    theme(panel.border = element_blank(),
          panel.background = element_rect(fill= NA),
          axis.line = element_line(colour = "black"),
          axis.title.x = element_blank(),
          axis.title.y = element_text(vjust = 1, size = 20),
          axis.text.x = element_blank(),
          axis.text.y  = element_text(size=20),
          plot.title = element_text(size = 20),
          legend.text = element_text(size = 20),
          legend.title = element_text(size = 20),
          strip.text.x = element_text(size = 20),
          axis.ticks.x=element_blank(),
          strip.text.y = element_text(size = 20))

  ZLD5_plot = ggplot(ExtendedRegionFile,
                     aes(x = as.numeric(ExtendedRegionFile$start)))+
    annotate("rect", xmin = RegionStart[1], xmax = RegionEnd[1],   ymin = 0, ymax = Inf,   alpha = 0.2, fill = "slategray4") +
    #geom_ribbon(aes(ymin = 0, ymax = as.numeric(ExtendedRegionFile$ZLD_st3)), colour = "steelblue", lwd = 1, fill = NA, alpha = 0.3, show.legend = TRUE)  +
    #geom_ribbon(aes(ymin = 0, ymax = as.numeric(ExtendedRegionFile$ZLD_st4)), colour = "steelblue3", lwd = 1, fill = NA, alpha = 0.3, show.legend = TRUE)  +
    geom_ribbon(aes(ymin = 0, ymax = as.numeric(ExtendedRegionFile$ZLD_st5)), colour = "steelblue1", lwd = 1, fill = "steelblue1", alpha = 0.3, show.legend = TRUE)  +
    #ylab("Normalized Wig Score") +
    #ylim(0,500) +
    scale_y_continuous(limits = c(0,500), breaks=c(0,500)) +
    theme(panel.border = element_blank(),
          panel.background = element_rect(fill= NA),
          axis.line = element_line(colour = "black"),
          axis.title.x = element_blank(),
          axis.title.y = element_text(vjust = 1, size = 20),
          axis.text.x = element_blank(),
          axis.text.y  = element_text(size=20),
          plot.title = element_text(size = 20),
          legend.text = element_text(size = 20),
          legend.title = element_text(size = 20),
          strip.text.x = element_text(size = 20),
          axis.ticks.x=element_blank(),
          strip.text.y = element_text(size = 20))

  Cad_plot = ggplot(ExtendedRegionFile,
                    aes(x = as.numeric(ExtendedRegionFile$start)))+
    annotate("rect", xmin = RegionStart[1], xmax = RegionEnd[1],   ymin = 0, ymax = Inf,   alpha = 0.2, fill = "slategray4") +
    geom_ribbon(aes(ymin = 0, ymax = as.numeric(ExtendedRegionFile$CADr)), colour = "mediumpurple4", lwd = 1, fill = "mediumpurple3", alpha = 0.3, show.legend = TRUE)  +
    #ylab("Normalized Wig Score") +
    #ylim(0,50) +
    scale_y_continuous(limits = c(0,50), breaks=c(0,50)) +
    theme(panel.border = element_blank(),
          panel.background = element_rect(fill= NA),
          axis.line = element_line(colour = "black"),
          axis.title.x = element_blank(),
          axis.title.y = element_text(vjust = 1, size = 20),
          axis.text.x = element_blank(),
          axis.text.y  = element_text(size=20),
          plot.title = element_text(size = 20),
          legend.text = element_text(size = 20),
          legend.title = element_text(size = 20),
          strip.text.x = element_text(size = 20),
          axis.ticks.x=element_blank(),
          strip.text.y = element_text(size = 20))

  Kni_plot = ggplot(ExtendedRegionFile,
                    aes(x = as.numeric(ExtendedRegionFile$start)))+
    annotate("rect", xmin = RegionStart[1], xmax = RegionEnd[1],   ymin = 0, ymax = Inf,   alpha = 0.2, fill = "slategray4") +
    geom_ribbon(aes(ymin = 0, ymax = as.numeric(ExtendedRegionFile$KNI)), colour = "navajowhite4", lwd = 1, fill = "navajowhite4", alpha = 0.3, show.legend = TRUE)  +
    #ylab("Normalized Wig Score") +
    #ylim(0,50) +
    scale_y_continuous(limits = c(0,50), breaks=c(0,50)) +
    xlab(paste(RegionChr[1])) +
    theme(panel.border = element_blank(),
          panel.background = element_rect(fill= NA),
          axis.line = element_line(colour = "black"),
          axis.title.x = element_text(size = 20),
          axis.title.y = element_text(vjust = 1, size = 20),
          axis.text.x = element_text(size=16),
          axis.text.y  = element_text(size=20),
          plot.title = element_text(size = 20),
          legend.text = element_text(size = 20),
          legend.title = element_text(size = 20),
          strip.text.x = element_text(size = 20),
          strip.text.y = element_text(size = 20),
          plot.margin = grid::unit(c(1, 0.5,
                                     0.5, 0.5), "cm"))


  #grid.draw(rbind(ggplotGrob(AntpostATAC), ggplotGrob(DnaseI_plot), ggplotGrob(BCD_plot), ggplotGrob(Hb_plot), ggplotGrob(KR_plot), ggplotGrob(GT_plot), ggplotGrob(ZLD3_plot), ggplotGrob(ZLD4_plot), ggplotGrob(ZLD5_plot), ggplotGrob(Cad_plot), ggplotGrob(Kni_plot), size = "last"))
  g1 = ggplotGrob(AntpostATAC)
  g2 = ggplotGrob(DnaseI_plot)
  g3 = ggplotGrob(BCD_plot)
  g4 = ggplotGrob(Hb_plot)
  g5 = ggplotGrob(KR_plot)
  g6 = ggplotGrob(GT_plot)
  g7 = ggplotGrob(ZLD3_plot)
  g8 = ggplotGrob(ZLD4_plot)
  g9 = ggplotGrob(ZLD5_plot)
  g10 = ggplotGrob(Cad_plot)
  g11 = ggplotGrob(Kni_plot)
  g = rbind(g1, g2, g3, g4, g5, g6, g7, g8, g9, g10, g11, size = "last")
  #g = rbind(ggplotGrob(AntpostATAC), ggplotGrob(DnaseI_plot), ggplotGrob(BCD_plot), ggplotGrob(Hb_plot), ggplotGrob(KR_plot), ggplotGrob(GT_plot), ggplotGrob(ZLD3_plot), ggplotGrob(ZLD4_plot), ggplotGrob(ZLD5_plot), ggplotGrob(Cad_plot), ggplotGrob(Kni_plot), size = "last")
  g$widths = unit.pmax(g1$widths, g2$widths, g3$widths, g4$widths, g5$widths, g6$widths, g7$widths, g8$widths, g9$widths, g10$widths, g11$widths)
  grid.draw(g)
  #print(p)
  dev.off()
  counter = counter + 1
  }

####################################################################
######### 020318 TF Heatmap                 ############ 
####################################################################

library("plyr")
library("dplyr")
library("ggplot2")
library("grid")
library("ComplexHeatmap")
library("circlize")
#read in file
RegionFile = read.csv2("020318_2Reps_NewGenelist_PE_Analysis_APEnhancers_PositionalScore.csv", sep = ",", stringsAsFactors = FALSE, header = TRUE)

#open region file and order by positional score and only do enhancers
RegionFileEnhOnly = RegionFile %>%
  select(Chr, Start, End, Name, Type, Location, PositionalScore) %>%
  filter(Type == "Enhancer") %>%
  filter(Location %in% c('Anterior', 'Posterior', 'Mostly Post')) %>%
  arrange(desc(as.numeric(PositionalScore)))
colnames(RegionFileEnhOnly) = c("chrom", "start", "end", "Name", "Type", "Location","Positional_score")

#find the length of the list
Length = as.numeric(length(RegionFileEnhOnly$chrom))

#make the names a list
Namelist = as.list(as.character(RegionFileEnhOnly$Name))


# Loop that sums the values in the window
counter = 1

#make a bunch of empty lists -- one for each row of the heatmap
BicoidList_win = list()
HbList_win = list()
KRList_win = list()
GTList_win = list()
ZLD_st3List_win = list()
ZLD_st4List_win = list()
ZLD_st5List_win = list()
CADrList_win = list()
KNIList_win = list()
# AntList_win = list()
# PostList_win = list()
# DnaseIList_win = list()
EnhancerNameList = list()
#WholeList_win = list()

while(counter <= Length){
  
  # Make the extended region file name from the name list
  NameofRegion = paste((Namelist[counter]) , 'WigFileSnapshot.txt', sep = "_")
  
  #load the extended region file
  ExtendedRegionFile = read.delim2(paste(NameofRegion), sep = "\t", stringsAsFactors = FALSE, header = FALSE)
  #Rename the columns
  colnames(ExtendedRegionFile) = c("chrom", "start", "end", 'whole', 'ant', 'post', 'dnaseI', 'Bcd',	'Hb',	'KR',	'GT',	'ZLD_st3', 'ZLD_st4', 'ZLD_st5', 'CADr', 'KNI')
  
  #remove the nan's 
  ExtendedRegionFile[ExtendedRegionFile == 'nan'] <- 0 #remove the nan
  ExtendedRegionFile[ExtendedRegionFile == 'NaN'] <- 0 #remove the nan
  
  #  # Need to find the max peak height for each TF for that region
  # # 'Bcd',	'Hb',	'KR',	'GT',	'ZLD_st3', 'ZLD_st4', 'ZLD_st5', 'CADr', 'KNI'
  EnhancerNameList = append(EnhancerNameList, as.character((Namelist[counter])))
  
  bcdwin = sum(as.numeric(ExtendedRegionFile$Bcd))
  BicoidList_win = append(BicoidList_win, bcdwin)
  
  Hbwin = sum(as.numeric(ExtendedRegionFile$Hb))
  HbList_win = c(HbList_win, Hbwin )
  
  KRwin = sum(as.numeric(ExtendedRegionFile$KR))
  KRList_win = c(KRList_win, KRwin )
  
  GTwin = sum(as.numeric(ExtendedRegionFile$GT))
  GTList_win = c(GTList_win, GTwin)
  
  ZLD_st3win = sum(as.numeric(ExtendedRegionFile$ZLD_st3))
  ZLD_st3List_win = c(ZLD_st3List_win, ZLD_st3win )
  
  ZLD_st4win = sum(as.numeric(ExtendedRegionFile$ZLD_st4))
  ZLD_st4List_win = c(ZLD_st4List_win, ZLD_st4win )
  
  ZLD_st5win = sum(as.numeric(ExtendedRegionFile$ZLD_st5))
  ZLD_st5List_win = c(ZLD_st5List_win, ZLD_st5win )
  
  CADrwin = sum(as.numeric(ExtendedRegionFile$CADr))
  CADrList_win = c(CADrList_win, CADrwin )
  
  KNIwin = sum(as.numeric(ExtendedRegionFile$KNI))
  KNIList_win = c(KNIList_win, KNIwin)
  
  # antwin = sum(as.numeric(ExtendedRegionFile$ant))
  # AntList_win = c(AntList_win, antwin)
  # 
  # postmax = max(as.numeric(ExtendedRegionFile$post))
  # PostList = c(PostList, postmax)
  # 
  # DnaseImax = max(as.numeric(ExtendedRegionFile$dnaseI))
  # DnaseIList = c(DnaseIList, DnaseImax)
  # 
  # WholeMax = max(as.numeric(ExtendedRegionFile$whole))
  # WholeList = c(WholeList, WholeMax)
  
  #Extend the counter
  counter = counter + 1
}

# Then I need to make a matrix with regions as the rows and the columns are TFs 
maxMatrix = matrix(c(EnhancerNameList, BicoidList_win, CADrList_win, KNIList_win, GTList_win, HbList_win, KRList_win, ZLD_st3List_win,  ZLD_st4List_win, ZLD_st5List_win), nrow=length(EnhancerNameList))
colnames(maxMatrix) = c("EnhancerNameList", "bicoid", "caudal", "knirps", "giant", "hunchback", "kruppel", "zelda stage 3", "zelda stage 4", "zelda stage 5")
maxMatrix = as.data.frame(maxMatrix)

#Then I need to transpose the matrix so that the rows are TFs and the columns are regions but keeping the first row as the headers
transposed_maxMatrix = setNames(data.frame(t(maxMatrix[,-1])), maxMatrix[,1])
Max_datamatrix = data.matrix(transposed_maxMatrix)

#Scale by TF 
maxs = apply(Max_datamatrix,1,max)
mins = apply(Max_datamatrix,1,min)
Max_datamatrixScaled = scale(t(Max_datamatrix), center = mins, scale = maxs - mins)


png('020318_XYscript_NewList_TFbinding_heatmap_1kbwin_Colclust.png', width = 4000, height = 2000, units = "px",  res=300) 
ha = HeatmapAnnotation(df = data.frame(PositionalScore = as.numeric(RegionFileEnhOnly$Positional_score)), col = list(PositionalScore = colorRamp2(c(-0.3,0, 0.46),c("dodgerblue2", "white", "darkorange"))))
Heatmap(t(Max_datamatrixScaled), top_annotation = ha, cluster_columns = TRUE, cluster_rows = FALSE, col = colorRampPalette(c("white", "black"))(500), column_title = "1kb windows") 
dev.off()
```

#### Peak Analysis --> global analysis of accessibility around peaks¶

In [ ]:

```
 ############################# Peak Random Regions #############################


#Wig Signal from all TF and ATAC data around these whole peaks
perl /Users/jennahaines/'Box Sync'/Eisen_Lab/Scripts/wig_sig_around_bedfile_013018.pl \
020818_WholePooledInRep1AndRep2.narrowPeak_JustRegions.bed \
~/020218_TFwigfiles \
021618_wig_sig_around_Wholepeaks_TFbindingDirectory.txt

#random regions to get ATAC skew/ Positional scores and significance
python 020618_RandomRegions_AroundPeaks.py \
-W 013118_wig_sig_around_bedfile_randomregions.txt \
-R 021618_wig_sig_around_Wholepeaks_TFbindingDirectory.txt \
-o 020618_030628WholePeaks_Randomregv2_output.txt


# ############################# Peak overlap #############################
perl -pe '$_ =~ tr/chr//d' 020818_WholePooledInRep1AndRep2.narrowPeak_JustRegions.bed > 020818_WholePooledInRep1AndRep2.narrowPeak_JustRegions_nochr.bed
#took out Uextra, and Many het chromosomes to do this analysis

bedtools intersect -wao -names Zelda1hr Zelda2hr Zelda3hr Hb Kr Gt Bcd Cad Kni Hoskins Kvon Prom H3K27acc14a H3K27acc14c H3K4me1c14a H3K4me1c14c \
-a 020718_WholePeaks_plusATACScore_PValue_nochr.bed \
-b \
100817s6_1hr_ZLD-Inpc_peaks_nochr.bed \
100817s7_2hr_ZLD-Inpc_peaks_nochr.bed \
100817s8_3hr_ZLD-Inpc-chr_peaks_nochr.bed \
DvS2TG_090826s6_HB_peaks_nochr.bed \
DvS2TG_090826s7_KR_peaks_nochr.bed \
DvS2TG_090826s8_GT_peaks_nochr.bed \
GSM511083_Dmel-BCD_peaks_nochr.bed \
GSM511087_Dmel-CADpeaks_nochr.bed \
GSM511088_Dmel-KNIpeaks_nochr.bed \
011618_ReviewsRevised_RevisedAPDV_enhPromRegions_nochr.bed \
Kvon_AllCRMS_nochr.bed \
Supplementary_data_file3_IntegratedPromoters_nochr_shortened.bed \
GSM1424902_Dmel-H3K27ac-c14a-peaks_nochr.bed \
GSM1424903_Dmel-H3K27ac-c14c-peaks_nochr.bed \
GSM1424906_Dmel-H3K4me1-c14a-peaks_nochr.bed \
GSM1424907_Dmel-H3K4me1-c14c-peaks_nochr.bed > 020718_ReplicatedwholePeaks_BedtoolsOverlap_Kitchensink.txt


# First I want to sort them to make everything go faster
sort -k1,1 -k2,2n 


python 020718_Pivotlistconvert.py -W 020718_ReplicatedwholePeaks_BedtoolsOverlap_Kitchensink.txt -o 020718_WholePeaks_Pivotlistconvert_output.txt -n Names.txt
```

In [ ]:

```
#####################################################################################################################
##########   122617_Pivotlistconvert.py                           ##########
##########   Input is bedtools intersect for annotation           ##########
#########    output is a text file with each peak and a column with true or false for each peak file intersected                                                   ##########
#####################################################################################################################
#!/usr/bin/env python

################# Argument Import : Uncomment for actual running script  #################
from optparse import OptionParser

def parse_options():
	parser = OptionParser()
	parser.add_option("-W", "--wigfile", dest="wigfile",
					  help="Directory where wig_snapshot.png are")
	parser.add_option("-o", "--output", dest="output",
					  help="14 columned TXT file of wanted genomic regions columns: Sample	Chr	Start	End	Whole	Ant	LinregPost	AplusP	Dnase1	Type	Location	Name	Zscore	Pvalue")
	parser.add_option("-n", "--names", dest="names",
					  help="Directory where insitu.png are")
	(options, args) = parser.parse_args()
	return options
            
options = parse_options()
parser = OptionParser()
if not options.wigfile:
    print("Wigfile option is missing\n")
    parser.print_help()
    exit(-1)
if not options.output:
    print("output file is missing\n")
    parser.print_help()
    exit(-1)
if not options.names:
    print("names file is missing\n")
    parser.print_help()
    exit(-1)

# Read in the peak list
PeakList_file = open(str(options.wigfile), 'r')
PeakList = PeakList_file.readlines()
PeakList_file.close()

#read in names file 

NamesList_file = open(str(options.names), 'r')
NamesList = NamesList_file.readlines()
NamesList_file.close()

#   C. Create output .txt file
outputfileName = str(options.output)
outputFile = open(str(outputfileName), 'w')

#   D. Take out the header of both random and enhancer ATAC files. it is a list
PeakListHeader = PeakList.pop(0)
PeakListHeader = PeakListHeader.strip('\n')
print(PeakListHeader)


print("files imported")

#Split the names and make them each dictionaries.
#Make List of all the list Names

NameListDict ={}
for line in NamesList:
    line2 = line.strip('\n')
    Nameheader = line
    NameListElement = line2.split('\t')
    for name in NameListElement:
        NameListDict[name] = [] #Make a dictionary such that each name is a key and the list is the value
        print(NameListDict.keys())
print("dictionary created")


#for each line separate by tab
#make a dictionary for each transcription factor 

PeakDictionary ={}

for line in PeakList:
    line2 = line.strip('\n')
    PeakListElement = line2.split('\t')
    PeakName = "-".join([PeakListElement[0], PeakListElement[1], PeakListElement[2]]) #make the peak name the chr_start_end
    PeakInfo = "\t".join([PeakListElement[0], PeakListElement[1], PeakListElement[2], PeakListElement[3], PeakListElement[4]])
    PeakDictionary[PeakName] = PeakInfo
    #PeakDictionary[PeakName] = line2 #Make a dictionary that has the peakname assigned to information calculated for that peak
    #print(PeakListElement)
    #print(PeakListElement)
    #print(str(PeakListElement[18]))
    # if the intersect field matches a listname add the peak number to that list 
    if  NameListDict.has_key(str(PeakListElement[5])):
        NameListDict[str(PeakListElement[5])].append(PeakName) # if there was a match then add the peak number to that list
        #print(NameListDict[str(PeakListElement[18])])

# Finally I need to write the output file

#first write out the header
#outputFile.write(PeakListHeader)
outputFile.write("chr\tstart\tend\tpositionalscore\tpvalue")
outputFile.write('\t')
outputFile.write(Nameheader)
outputFile.write('\n')


for peak in PeakDictionary:
    outputFile.write(PeakDictionary[peak]) # First write the peak info
    # then cycle through all the keys and ask if this peak is in that dictionary, if it is write true else write false then move on to next key
    counter = 0
    while counter < len(NameListElement) : 
        if peak in NameListDict[NameListElement[counter]]:
            outputFile.write("\tTRUE")
        else:
            outputFile.write("\tFALSE")
        counter += 1
    outputFile.write('\n')

#print(NameListDict['GT'])
```

##### S3 Files¶

In [ ]:

```
python 071317_PDFoutput_wigfile.py \
-W ~/020118_BrowserTraces_1kb \
-R ~/021218_AllSamplesUsed_Peak_Insitu_PDFINPUT.txt \
-S ~/InSitus
```

In [ ]:

```
#############################################################
######### 071317_PDFoutput_wigfile.py               #########
######### Take a directory of wig snapshots,        #########
#########  insitus, and data and compile it into    #########
#########  one pdf per gene                         #########
#############################################################

#!/usr/bin/env python


import os
from math import *
from optparse import OptionParser
import sys
import re
from pylab import figure, title, xlabel, ylabel, hist, axis, grid, savefig
import matplotlib.pyplot as plt
from matplotlib.backends.backend_pdf import PdfPages
import numpy as np
from scipy.stats import norm
from reportlab.lib.enums import TA_JUSTIFY
from reportlab.lib.pagesizes import letter
from reportlab.platypus import SimpleDocTemplate, Paragraph, Spacer, Image
from reportlab.lib.styles import getSampleStyleSheet, ParagraphStyle
from reportlab.lib.units import inch

def parse_options():
	parser = OptionParser()
	parser.add_option("-W", "--wigfileDest", dest="wigfileDest",
					  help="Directory where wig_snapshot.png are")
	parser.add_option("-R", "--regfile", dest="regfile",
					  help="14 columned TXT file of wanted genomic regions columns: Sample	Chr	Start	End	Whole	Ant	LinregPost	AplusP	Dnase1	Type	Location	Name	Zscore	Pvalue")
	parser.add_option("-S", "--insitu", dest="insitu",
					  help="Directory where insitu.png are")
	(options, args) = parser.parse_args()
	return options
            
options = parse_options()
parser = OptionParser()
if not options.wigfileDest:
    print("Wigfile option is missing\n")
    parser.print_help()
    exit(-1)
if not options.regfile:
    print("Region file option is missing\n")
    parser.print_help()
    exit(-1)
if not options.insitu:
    print("output file is missing\n")
    parser.print_help()
    exit(-1)

################# Data Import   #################

RegionList_file = open(str(options.regfile), 'r')
WigfileDirectory = str(options.wigfileDest)
InsituDirectory = str(options.insitu)


RegionList = RegionList_file.readlines()
RegionList_file.close()

#print(RegionList)

#Make a test regionList
regionList_test = RegionList[0:10]

enhInfoDict={}
enhLocDict={}
enhTypeDict={}
enhZScoreDict={}
enhPValueDict={}
enhNameList =[]


#for reportlab: Justifying the text for some reason. I probably don't need to do this
styles=getSampleStyleSheet()
styles.add(ParagraphStyle(name='Justify', alignment=TA_JUSTIFY))


with PdfPages('multipage_pdf.pdf') as pdf: # making a pdf called multipage_pdf.pdf
    for enhancer in RegionList:
        line2 = enhancer.strip('\n')
        enhListElement = line2.split('\t')
        enhNameList.append(enhListElement[10])
        enhInfoDict[enhListElement[10]] = line2 #Dictionary that returns the line without line ending
        enhLocDict[enhListElement[10]] = enhListElement[9] #Dictionary that returns Location
        enhTypeDict[enhListElement[10]] = enhListElement[8] #Dictionary that returns Type
        enhZScoreDict[enhListElement[10]] = enhListElement[11] #Dictionary that returns ZScore
        enhPValueDict[enhListElement[10]] = enhListElement[12] #Dictionary that returns Pvalue
        Wigname = "".join([str(enhListElement[10]), "_WigFileSnapshot.png"]) #making the wigname
        PDFTitle = str(enhListElement[10]) # Making the PDF title the name of the enhancer
        PDFName = "".join([PDFTitle, "_Report.pdf"]) #Making the PDFName for the PDF File name the name of the enhancer + .pdf
        #for reportlab: first specify the document format
        doc = SimpleDocTemplate(PDFName,pagesize=letter,
                            rightMargin=72,leftMargin=72,
                            topMargin=72,bottomMargin=18)
        #for reportlab: make a story variable that is a list that contains all images + text
        Story=[]
        ptext = '<font size=20>%s</font>' % PDFTitle #creating the title from the enhancer name
        Story.append(Paragraph(ptext, styles["Normal"])) #Adding the title to the story
        Story.append(Spacer(2, 12)) #adding a space of one line below the title
        for file in os.listdir(WigfileDirectory): #list the contents of the wigfile directory
            #print(file) #TEST
            if str(file) == Wigname:  #if the file is the same as the enhancer 
                WigSnapshotimage = str(file) #make a string of the file name
                WigSnapshotimage_Path = "".join([str(WigfileDirectory) , "/", WigSnapshotimage]) #add the path
                WigSnapshotimage_object = Image(WigSnapshotimage_Path, 5*inch, 4*inch) #make an image object
                #print(WigSnapshotimage_object) # TEST
                Story.append(WigSnapshotimage_object) #add it to the story
                Story.append(Spacer(1, 12)) # add a space 
        for insitu in os.listdir(InsituDirectory): #For each insitu in the insitu directory
            #print(insitu) #TEST
            insituName = insitu.split("_insit")
            insituName = insituName[0] #Obtain the insitu name 
            #print(insituName) #TEST
            if str(insituName) == str(enhListElement[10]): #if the insitu name is the same as the enhancer name
                Insituimage = str(insitu) #make the insitu file a string 
                Insituimage_Path = "".join([str(InsituDirectory) , "/", Insituimage]) #add it's path
                Insituimage_object = Image(Insituimage_Path, 5*inch, 4*inch) #make it into an image object
                Story.append(Insituimage_object) #append it to the PDF
        #Creating the string from the pvalue and Zscore
        DataString = "".join(["Location: ", str(enhListElement[9]), " Type: ", str(enhListElement[8]), " ZScore: ", str(enhListElement[11]), " PValue: ", str(enhListElement[12])])
        DataText = '<font size=12>%s</font>' % DataString #create the text with the enhancer data 
        Story.append(Paragraph(DataText, styles["Normal"])) #Adding the data text to the story
        doc.build(Story) #build the story list into the document
        print(Story) #TEST
```

## Figure 6¶

#### Cusanovich 2018¶

In [ ]:

```
####################################################################################################
##########   040418_postalign_RemoveDuplicates_SortedBam_PairedEnd_Sam_FilterFragments.sh ##########
##########   Input:  bam file from Cusanovich 2017                                        ##########
#########    Output: shifted bed file, merged bed files, wig file, 10Mnormalized wig file ##########
####################################################################################################

#bash 040418_postalign_RemoveDuplicates_SortedBam_PairedEnd_Sam_FilterFragments.sh 2> 040418_postalign_RemoveDuplicates_SortedBam_PairedEnd_Sam_FilterFragments_STERR.txt | cat > 040418_postalign_RemoveDuplicates_SortedBam_PairedEnd_Sam_FilterFragments_STOUT.txt &

#! /bin/bash

# bam file from Cusanovich 2018
Bowtie2SamFile[1]=SCatac_tha.bowtie.2to4.fixed.nodups.15.bam
Bowtie2SamFile[2]=SCatac_tha.bowtie.2to4.fixed.nodups.16.bam
Bowtie2SamFile[3]=SCatac_tha.bowtie.2to4.fixed.nodups.4.bam
Bowtie2SamFile[4]=SCatac_tha.bowtie.2to4.fixed.nodups.6.bam
Bowtie2SamFile[5]=SCatac_tha.bowtie.2to4.fixed.nodups.7.bam


#Core name that will be carried with the sample

filesetname[1]=SCatac_tha.bowtie.2to4.fixed.nodups.15
filesetname[2]=SCatac_tha.bowtie.2to4.fixed.nodups.16
filesetname[3]=SCatac_tha.bowtie.2to4.fixed.nodups.4
filesetname[4]=SCatac_tha.bowtie.2to4.fixed.nodups.6
filesetname[5]=SCatac_tha.bowtie.2to4.fixed.nodups.7
                         

for k in 1 2 3 4 5
do
    samfile=${Bowtie2SamFile[${k}]}
    echo 'sam file imported'
    filesetname=${filesetname[${k}]}
    samtools sort -n ${filesetname}.bam ${filesetname}.srt.temp 
    bedtools bamtobed -bedpe -i ${filesetname}.srt.temp.bam | perl -n -e '@A = split (/\t/, $_); $start = $A[1] +4 ; $end = $A[5] - 6 ; print "$A[0]\t$start\t$end\t$A[6]\t$A[7]\t$A[8]\t$[9]\n"' > 040418_${filesetname}_shifted.bed
    echo 'shifted bed PE file made'
    # did not filter based on the 130bp threshold..since they did not do that in their pipeline
    #perl -n -e '@A = split (/\t/, $_); $readsize = abs($A[2] - $A[1]); print $_' 011617_${filesetname}_shifted.bed > 011617_${filesetname}_shifted.bed
    #echo 'filtered based on 130 bp threshold'
   
done

#merge anterior (clusters 6 and 15) and posterior (4,7,16) beds together

cat 040418_SCatac_tha.bowtie.2to4.fixed.nodups.6_shifted.bed 040418_SCatac_tha.bowtie.2to4.fixed.nodups.15_shifted.bed > 040418_MERGED_ANTERIOR_SCatac_tha.bowtie.2to4.fixed.nodups_shifted.bed
cat 040418_SCatac_tha.bowtie.2to4.fixed.nodups.4_shifted.bed 040418_SCatac_tha.bowtie.2to4.fixed.nodups.7_shifted.bed 040418_SCatac_tha.bowtie.2to4.fixed.nodups.16_shifted.bed > 040418_MERGED_POSTERIOR_SCatac_tha.bowtie.2to4.fixed.nodups_shifted.bed
echo 'Merged Bed files'


mkdir Bedfiles_040418
mv 040418_MERGED_*.bed Bedfiles_040418
perl ~/scripts/xl-bed2wig-dirproc-HOA-2014-new-chrsz Bedfiles_040418
echo 'made wig files'

#This script will take the filtered bed files.. count the number of reads and then use that to noramlize the wig files to 1M filtered reads

MERGEDfilesetname[1]=040418_MERGED_ANTERIOR_SCatac_tha.bowtie.2to4.fixed.nodups_shifted
MERGEDfilesetname[2]=040418_MERGED_POSTERIOR_SCatac_tha.bowtie.2to4.fixed.nodups_shifted

for k in 1 2
do
  cd Bedfiles_040418
  filesetname=${MERGEDfilesetname[${k}]}
  NumberofReads=$(wc -l < "${filesetname}.bed")
  echo "$filesetname" > outputfile.txt
  export NumberofReads
  echo "$NumberofReads" > outputfile.txt
  echo "Normalizing to 1M reads"
  perl -n -e ' $Scale = 1000000/$ENV{NumberofReads} ; if (/^\d+/) {@A = split (/\s+/, $_); $norm = $A[1] * $Scale ; print "$A[0]\t $norm \n"} else { print "$_"}' ${filesetname}.wig > ${filesetname}1MNorm.wig
  echo "${filesetname} Normalized wig File made"

done
```

#### Linear regression Normalization of Anterior to Posterior¶

In [ ]:

```
# Script to generate wig signal around 1kb regions
perl /Users/jennahaines/'Box Sync'/Eisen_Lab/Scripts/wig_sig_around_bedfile_013018.pl \
/Users/jennahaines/'Box Sync'/Eisen_Lab/Experiments/ATAC-seq/Halves/ATAC-seq_Pools/040617_Analysis/061417_BrowserTraces/062317_RegressionNormalization/011618_PairedEndAnalysisforReviews/Dmel_1kb_windows.bed \
/Users/jennahaines/'Box Sync'/Eisen_Lab/Experiments/ATAC-seq/Halves/ATAC-seq_Pools/040617_Analysis/061417_BrowserTraces/062317_RegressionNormalization/011618_PairedEndAnalysisforReviews/SingleCellPeaks/032918_mixedbedfiles/NormalizedWigs/040418_1MNormWigs \
040518_SingleCell_1kb.txt
```

In [ ]:

```
setwd("~/Box Sync/Eisen_Lab/Experiments/ATAC-seq/Halves/ATAC-seq_Pools/040617_Analysis/061417_BrowserTraces/062317_RegressionNormalization/011618_PairedEndAnalysisforReviews/SingleCellPeaks/032918_mixedbedfiles/040518_SingleCell/")

merged_norm_1kb = read.delim2("040518_SingleCell_1kb.txt", sep = "\t", stringsAsFactors = FALSE) 
colnames(merged_norm_1kb)  = c("chr", "start", "end", "scATAC_Anterior", "scATAC_Posterior")

sciATAC_AvsP.lm = lm(as.numeric(merged_norm_1kb$scATAC_Anterior) ~ as.numeric(merged_norm_1kb$scATAC_Posterior))


# Call:
#   lm(formula = as.numeric(merged_norm_1kb$scATAC_Anterior) ~ as.numeric(merged_norm_1kb$scATAC_Posterior))
# 
# Coefficients:
#   (Intercept)  
# 1.2800  
# as.numeric(merged_norm_1kb$scATAC_Posterior)  
# 0.9255
```

In [ ]:

```
perl -n -e ' $Scale = 0.9255 ; $Yint = 0 ; if (/^\d+/) {@A = split (/\s+/, $_); $norm = ($A[1] - $Yint) / $Scale ; print "$A[0]\t $norm \n"} else { print "$_"}' 040418_MERGED_ANTERIOR_SCatac_tha.bowtie.2to4.fixed.nodups_shifted1MNorm.wig > 040418_MERGED_ANTERIOR_SCatac_tha.bowtie.2to4.fixed.nodups_shifted1MNorm_linregnorm.wig


#Sum wig signal around AP regions
perl wig_sig_around_bedfile_013018.pl \
012918_Redid_011618_ReviewsRevised_RevisedAPDV_enhPromRegions.bed \
040518_LinregNormalizedWigs \
040518_SingleCell_APregions.txt

# Summarize around random regions

perl wig_sig_around_bedfile_013018.pl \
013118_081517_RandomRegions_Excl_flybasegenes_Enhancers.bed \
~/RandomRegionNormalizedWigs \
040518_sciATAC_wig_sig_around_bedfile_randomregions.txt


# 040518_RandomDistributionScript_V5_forOverachievers.py wrote for this data 
python 040518_RandomDistributionScript_V5_forOverachievers.py \
-W 040518_sciATAC_wig_sig_around_bedfile_randomregions.txt \
-R 040518_SingleCell_APregions_matchback.txt \
-o 040518_sciATAC_Randomregv2_output_LinRegWhole.txt
```

### Figure 6B-E¶

In [ ]:

```
allSINGLECELLSamples <-read.delim2("040518_sciATAC_Randomregv2_output_LinRegWhole.txt", sep = "\t", stringsAsFactors = FALSE)

allPeaksFile <- read.delim2("021518_2reps_REppeaks_overlap_REVISEDGENELIST.txt", sep ="\t", stringsAsFactors = FALSE, header = FALSE)

allPeaks = allPeaksFile %>%
  select(V1, V2, V3, V6, V11, V12, V13, V17, V21)
colnames(allPeaks) = c("chr", "start", "end", "name", "chrPeak",	"startPeak",	"endPeak", "fold_enrichment", "Peakname")

allPeaksWhole <- allPeaks %>%
  dplyr::filter(grepl(pattern = "whole", Peakname)) %>%
  group_by(name) %>%
  arrange(as.numeric(fold_enrichment)) %>%
  dplyr::top_n(1, as.numeric(fold_enrichment))
allPeaksAnt <- allPeaks %>%
  dplyr::filter(grepl(pattern = "Anterior", Peakname)) %>%
  group_by(name) %>%
  arrange(as.numeric(fold_enrichment)) %>%
  dplyr::top_n(1, as.numeric(fold_enrichment))
allPeaksPost <- allPeaks %>%
  dplyr::filter(grepl(pattern = "Posterior", Peakname)) %>%
  group_by(name) %>%
  arrange(as.numeric(fold_enrichment)) %>%
  dplyr::top_n(1, as.numeric(fold_enrichment))
allPeaksNone <- allPeaks %>%
  dplyr::filter(Peakname == ".")

allSINGLECELLSamples$WholePeaks = as.numeric(allPeaksWhole$fold_enrichment)[match(allSINGLECELLSamples$Name, allPeaksWhole$name)]
allSINGLECELLSamples$AntPeaks = as.numeric(allPeaksAnt$fold_enrichment)[match(allSINGLECELLSamples$Name, allPeaksAnt$name)]
allSINGLECELLSamples$PostPeaks = as.numeric(allPeaksPost$fold_enrichment)[match(allSINGLECELLSamples$Name, allPeaksPost$name)]
allSINGLECELLSamples$NoPeak = as.character(allPeaksNone$fold_enrichment)[match(allSINGLECELLSamples$Name, allPeaksNone$name)]

allSamples_SINGLECELL_enhancers_ATACSkew_Peaks = allSINGLECELLSamples %>%
  filter(X011617.use == "yes") %>%
  filter(New.Location.Assignment %in%  c('Anterior', 'Posterior', 'Mostly Post')) %>%
  filter(Type == 'Enhancer') %>%
  mutate(Dotsize = 1.5) %>%
  distinct()

allSamples_SINGLECELL_enhancers_ATACSkew_Peaks$New.Location.Assignment <- factor(allSamples_SINGLECELL_enhancers_ATACSkew_Peaks$New.Location.Assignment,
                                                                                 levels = c('Anterior', 'Posterior', 'Mostly Post'),ordered = TRUE)
ggplot(allSamples_SINGLECELL_enhancers_ATACSkew_Peaks,
       aes(x = reorder(as.factor(Name),- as.numeric(ATACSkewScore)),
           y = as.numeric(ATACSkewScore),
           fill = New.Location.Assignment)) +
  geom_col(position = "stack", colour = "black") +
  ylim(-1, 1) +
  xlab("A-P Enhancers") +
  ylab("PositionalScore") +
  ggtitle("040118_sciATAC_AP_Enhancers_PositionalScore_Bargraph_Position_0118newgeneList_LINREGNORM") +
  scale_fill_manual(name = "Legand", values=c("darkorange1", "dodgerblue3", "dodgerblue3")) +
  theme(panel.border = element_blank(),
        panel.background = element_rect(fill= NA),
        axis.line = element_line(colour = "black"),
        axis.title.x = element_text(vjust = 0, size = 15),
        axis.title.y = element_text(vjust = 1, size = 15),
        axis.text.x = element_text(size = 7, angle = -90),
        axis.text.y  = element_text(size= 15),
        plot.title = element_text(size = 15),
        legend.text = element_text(size = 15),
        legend.title = element_text(size = 15),
        strip.text.x = element_text(size = 12),
        strip.text.y = element_text(size = 12)) +
  png('040118_sciATAC_APEnhancers_Bargraph_LinRegNorm.png', width = 5000, height = 2000, units = "px",  res=300)
dev.off()

SignificantPvalue = allSINGLECELLSamples %>%
  select(Name, Type, New.Location.Assignment, PValue, ATACSkewScore) %>%
  arrange(as.numeric(PValue))
######### ######### ######### AP Promoters Bargraphs 
#########  Filter out A vs P enhancers to calculate the positional score

allSamples_SINGLECELL_Promoters_ATACSkew_Peaks = allSINGLECELLSamples %>%
  filter(X011617.use == "yes") %>%
  filter(New.Location.Assignment %in%  c('Anterior', 'Mostly Ant', 'Posterior', 'Mostly Post')) %>%
  filter(Type == 'Promoter') %>%
  mutate(Dotsize = 1.5) %>%
  distinct()

allSamples_SINGLECELL_Promoters_ATACSkew_Peaks$New.Location.Assignment <- factor(allSamples_SINGLECELL_Promoters_ATACSkew_Peaks$New.Location.Assignment,
                                                                                 levels = c('Anterior', 'Mostly Ant', 'Posterior', 'Mostly Post'),ordered = TRUE)
# AP Promoters Bargraph
ggplot(allSamples_SINGLECELL_Promoters_ATACSkew_Peaks,
       aes(x = reorder(as.factor(Name),- as.numeric(ATACSkewScore)),
           y = as.numeric(ATACSkewScore),
           fill = New.Location.Assignment)) +
  geom_col(position = "stack", colour = "black") +
  ylim(-1, 1) +
  xlab("A-P Promoters") +
  ylab("PositionalScore") +
  ggtitle("040518_sciATAC_AP_Promoters_PositionalScore_Bargraph_Position_0118newgeneList_LINREGNORM") +
  scale_fill_manual(name = "Legand", values=c("darkorange1", "darkorange1", "dodgerblue3", "dodgerblue3")) +
  theme(panel.border = element_blank(),
        panel.background = element_rect(fill= NA),
        axis.line = element_line(colour = "black"),
        axis.title.x = element_text(vjust = 0, size = 15),
        axis.title.y = element_text(vjust = 1, size = 15),
        axis.text.x = element_text(size = 7, angle = -90),
        axis.text.y  = element_text(size= 15),
        plot.title = element_text(size = 15),
        legend.text = element_text(size = 15),
        legend.title = element_text(size = 15),
        strip.text.x = element_text(size = 12),
        strip.text.y = element_text(size = 12)) +
  png('040518_sciATAC_APPromoters_Bargraph_LinRegNorm.png', width = 5000, height = 2000, units = "px",  res=300)
dev.off()

############# scATAC vs Halves Positional Skew Score comparison

sci_vs_Halves_skewScore = allSINGLECELLSamples %>%
  mutate(MYDATA_Skewscore = (as.numeric(X011718_MERGED_2reps_081817_RemDUP_041217_Bowtie2_ME_JH_112315_20Ant_shifted_lessthan130_10MNorm_linregwhole) - as.numeric(X011718_MERGED_2reps_081817_RemDUP_041217_Bowtie2_ME_JH_112315_20Post_shifted_lessthan130_10MNorm_linregwhole)) / (as.numeric(X011718_MERGED_2reps_081817_RemDUP_041217_Bowtie2_ME_JH_112315_20Ant_shifted_lessthan130_10MNorm_linregwhole) + as.numeric(X011718_MERGED_2reps_081817_RemDUP_041217_Bowtie2_ME_JH_112315_20Post_shifted_lessthan130_10MNorm_linregwhole))) %>%
  select(Name, MYDATA_Skewscore, ATACSkewScore, New.Location.Assignment)

cor(sci_vs_Halves_skewScore$MYDATA_Skewscore, as.numeric(sci_vs_Halves_skewScore$ATACSkewScore))

png('040918_sci_vs_Halves_skewScore.png', width = 2000, height = 2000, units = "px",  res=300) 
ggplot(sci_vs_Halves_skewScore,
       aes(x = as.numeric(sci_vs_Halves_skewScore$MYDATA_Skewscore),y= as.numeric(sci_vs_Halves_skewScore$ATACSkewScore), fill = as.factor(New.Location.Assignment))) +
  geom_point(colour="grey5", alpha = 0.5) +
  ggtitle('040918_sci_vs_Halves_skewScore') +
  xlab('Halves ATACSkewScore') +
  ylab('Sc ATACSkewScore') +
  ylim(-1,1) + 
  xlim(-1,1) +
  coord_equal() +
  geom_abline(slope = 1, intercept=0, linetype = 'dotted') +
  theme(panel.border = element_rect(fill = NA),
        panel.background = element_rect(fill= NA),
        axis.title.x = element_text(vjust = 0, size = 12),
        axis.title.y = element_text(vjust = 1, size = 12),
        axis.text.x = element_text(size=15),
        axis.text.y  = element_text(size=15),
        plot.title = element_text(size = 12),
        legend.text = element_text(size = 10),
        legend.title = element_text(size = 10),
        strip.text.x = element_text(size = 15),
        strip.text.y = element_text(size = 15))
dev.off()
```
