# Supplementary material for: Patterns of chromatin accessibility along the anterior-posterior axis in the early Drosophila embryo
Source: PLoS Genet. 2018 May 4;14(5):e1007367. doi: 10.1371/journal.pgen.1007367 (PMC5955596; doi:10.1371/journal.pgen.1007367)
Supplement: S1 Text — (DOCX) [file pgen.1007367.s017.docx]

**Supplementary References**

90. Kvon EZ, Stampfel G, Yáñez-Cuna JO, Dickson BJ, Stark A. HOT regions function as patterned developmental enhancers and have a distinct cis-regulatory signature. Genes Dev. 2012 May 1;26(9):908–13.

91. Lin M-D, Fan S-J, Hsu W-S, Chou T-B. Drosophila decapping protein 1, dDcp1, is a component of the oskar mRNP complex and directs its posterior localization in the oocyte. Dev Cell. 2006 May;10(5):601–13.

92. Huang JD, Schwyter DH, Shirokawa JM, Courey AJ. The interplay between multiple enhancer and silencer elements defines the pattern of decapentaplegic expression. Genes Dev. 1993 Apr;7(4):694–704.

93. Hodar C, Zuñiga A, Pulgar R, Travisany D, Chacon C, Pino M, et al. Comparative gene expression analysis of Dtg, a novel target gene of Dpp signaling pathway in the early Drosophila melanogaster embryo. Gene. 2014 Feb 10;535(2):210–7.

94. Zeitlinger J, Zinzen RP, Stark A, Kellis M, Zhang H, Young RA, et al. Whole-genome ChIP–chip analysis of Dorsal, Twist, and Snail suggests integration of diverse patterning processes in the Drosophila embryo. Genes Dev. 2007 Feb 15;21(4):385–90.

95. Kirov N, Childs S, O’Connor M, Rushlow C. The Drosophila dorsal morphogen represses the tolloid gene by interacting with a silencer element. Mol Cell Biol. 1994 Jan;14(1):713–22.

96. Ozdemir A, Fisher-Aylor KI, Pepke S, Samanta M, Dunipace L, McCue K, et al. High resolution mapping of Twist to DNA in Drosophila embryos: Efficient functional analysis and evolutionary conservation. Genome Res. 2011 Apr;21(4):566–77.

97. Sandmann T, Girardot C, Brehme M, Tongprasit W, Stolc V, Furlong EEM. A core transcriptional network for early mesoderm development in Drosophila melanogaster. Genes Dev. 2007 Feb 15;21(4):436–49.

98. Stathopoulos A, Tam B, Ronshaugen M, Frasch M, Levine M. pyramus and thisbe: FGF genes that pattern the mesoderm of Drosophila embryos. Genes Dev. 2004 Mar 15;18(6):687–99.

99. Markstein M, Zinzen R, Markstein P, Yee K-P, Erives A, Stathopoulos A, et al. A regulatory code for neurogenic gene expression in the Drosophila embryo. Development. 2004 May;131(10):2387–94.

100. Ip YT, Park RE, Kosman D, Yazdanbakhsh K, Levine M. dorsal-twist interactions establish snail expression in the presumptive mesoderm of the Drosophila embryo. Genes Dev. 1992 Aug;6(8):1518–30.

101. Yin Z, Xu XL, Frasch M. Regulation of the twist target gene tinman by modular cis-regulatory elements during early mesoderm development. Development. 1997 Dec;124(24):4971–82.

102. Jiang J, Kosman D, Ip YT, Levine M. The dorsal morphogen gradient regulates the mesoderm determinant twist in early Drosophila embryos. Genes Dev. 1991 Oct;5(10):1881–91.
